# Terphenyl-Based Small-Molecule Inhibitors of Programmed Cell Death-1/Programmed Death-Ligand 1 Protein–Protein Interaction

**DOI:** 10.1021/acs.jmedchem.1c00957

**Published:** 2021-07-27

**Authors:** Damian Muszak, Ewa Surmiak, Jacek Plewka, Katarzyna Magiera-Mularz, Justyna Kocik-Krol, Bogdan Musielak, Dominik Sala, Radoslaw Kitel, Malgorzata Stec, Kazimierz Weglarczyk, Maciej Siedlar, Alexander Dömling, Lukasz Skalniak, Tad A. Holak

**Affiliations:** † Department of Organic Chemistry, Faculty of Chemistry, Jagiellonian University, Gronostajowa 2, 30-387 Krakow, Poland; ‡ Malopolska Centre of Biotechnology, Jagiellonian University, Gronostajowa 7a, 30-387 Kraków, Poland; § Department of Clinical Immunology, Institute of Pediatrics, Jagiellonian University Medical College, Wielicka 265, 30-663 Krakow, Poland; ∥ Department of Drug Design, University of Groningen, A. Deusinglaan 1, 9713 AV Groningen, The Netherlands

## Abstract

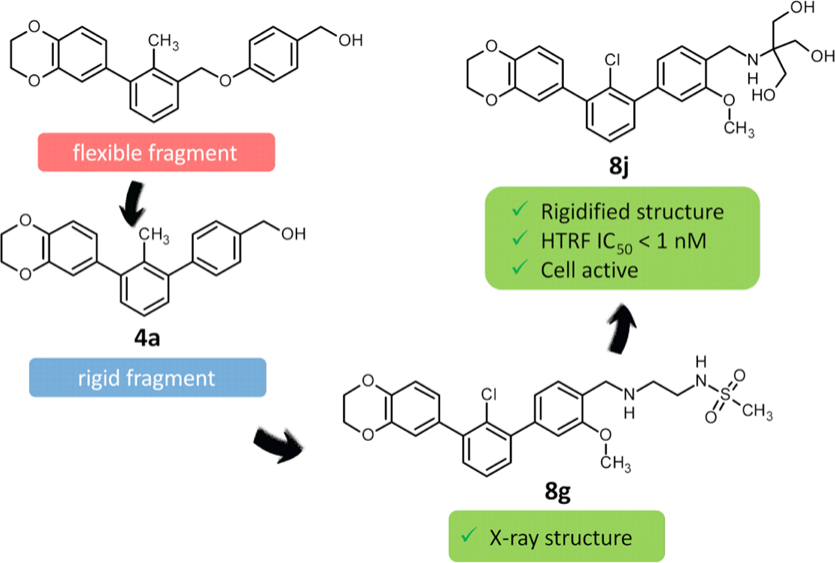

We describe a new class of potent PD-L1/PD-1 inhibitors based on a terphenyl scaffold that is derived from the rigidified biphenyl-inspired structure. Using *in silico* docking, we designed and then experimentally demonstrated the effectiveness of the terphenyl-based scaffolds in inhibiting PD-1/PD-L1 complex formation using various biophysical and biochemical techniques. We also present a high-resolution structure of the complex of PD-L1 with one of our most potent inhibitors to identify key PD-L1/inhibitor interactions at the molecular level. In addition, we show the efficacy of our most potent inhibitors in activating the antitumor response using primary human immune cells from healthy donors.

## Introduction

Cancer immunotherapy aims to stimulate the immune system’s ability to fight cancer as opposed to directly killing cancer cells with more traditional methods such as chemotherapy and radiation therapy.^1−6^ Inhibition of negative immune checkpoint regulators is now used in clinics around the world and was recognized with the 2018 Nobel Prize in Physiology or Medicine.^7^ One of the most important immune checkpoints (ICs) in cancer immunotherapy consists of programmed cell death protein 1 (PD-1, also known as CD279) with its ligand (PD-L1, known also as CD274 or B7-H1).^4^

The PD-1/PD-L1 axis is responsible for inhibiting excessive stimulation and normally aims at maintaining immune tolerance to self-antigens by negatively regulating the immune response.^8−10^ By blocking the PD-1/PD-L1 interaction, activation of T cells can be achieved for an improved antitumor response. Therefore, the immune checkpoint blockade (ICB) of PD-1 or PD-L1 at the tumor cell–T cell interface has become an attractive strategy for cancer immunotherapy.^11,12^

Traditionally, the inhibition of PD-1/PD-L1 interactions is achieved with monoclonal antibodies (mAbs) that target PD-1 (e.g., pembrolizumab, nivolumab, cemiplimab, etc.) or PD-L1 (e.g., atezolizumab, avelumab, and durvalumab).^3^ Despite their medical and commercial success, mAb-based immunotherapies suffer from several disadvantages, including high treatment costs, immune-related adverse events (irAE), and poor penetration of solid tumors.^13,14^ Small-molecule inhibitors (SMIs) are expected to overcome these limitations, with additional benefits such as oral bioavailability and lower production cost.^15,16^

Although the PD-1/PD-L1 interface is considered difficult to treat with SMIs due to its large, flat surface of interaction with no visible binding pockets, the number of patents and publications on anti-PD-L1 SMIs is constantly growing.^17,18^

A common feature of virtually all reported PD-1/PD-L1 SMI is that they target PD-L1 through a biphenyl-based scaffold originally developed by Bristol Myers Squibb (BMS).^19−21^ However, none of these molecules has progressed into clinical trials so far. As of now, only the peptide-derived compound **CA-170** from Curis and Aurigene is undergoing clinical trials as a small molecule that directly targets the PD-L1, PD-L2, and the V-domain Ig suppressor of T cell activation (VISTA) IC. However, it turned out that **CA-170** is not a classic PD-L1-blocker and alternative modes of its action have been postulated.^22,23^

Here, we present a new class of potent PD-L1 inhibitors based on a rigidified biphenyl structure. These compounds, which comprise the terphenyl scaffold, are effective in blocking PD-1/PD-L1 interactions in a variety of biophysical and cellular assays. We also provide a high-resolution structure of PD-L1 bound to the terphenyl small molecule to identify key PD-L1/ligand interactions on the PD-L1 interface.

## Results and Discussion

### Identification of Potent Terphenyl-Based PD-L1 Inhibitors

**Figure 1:**
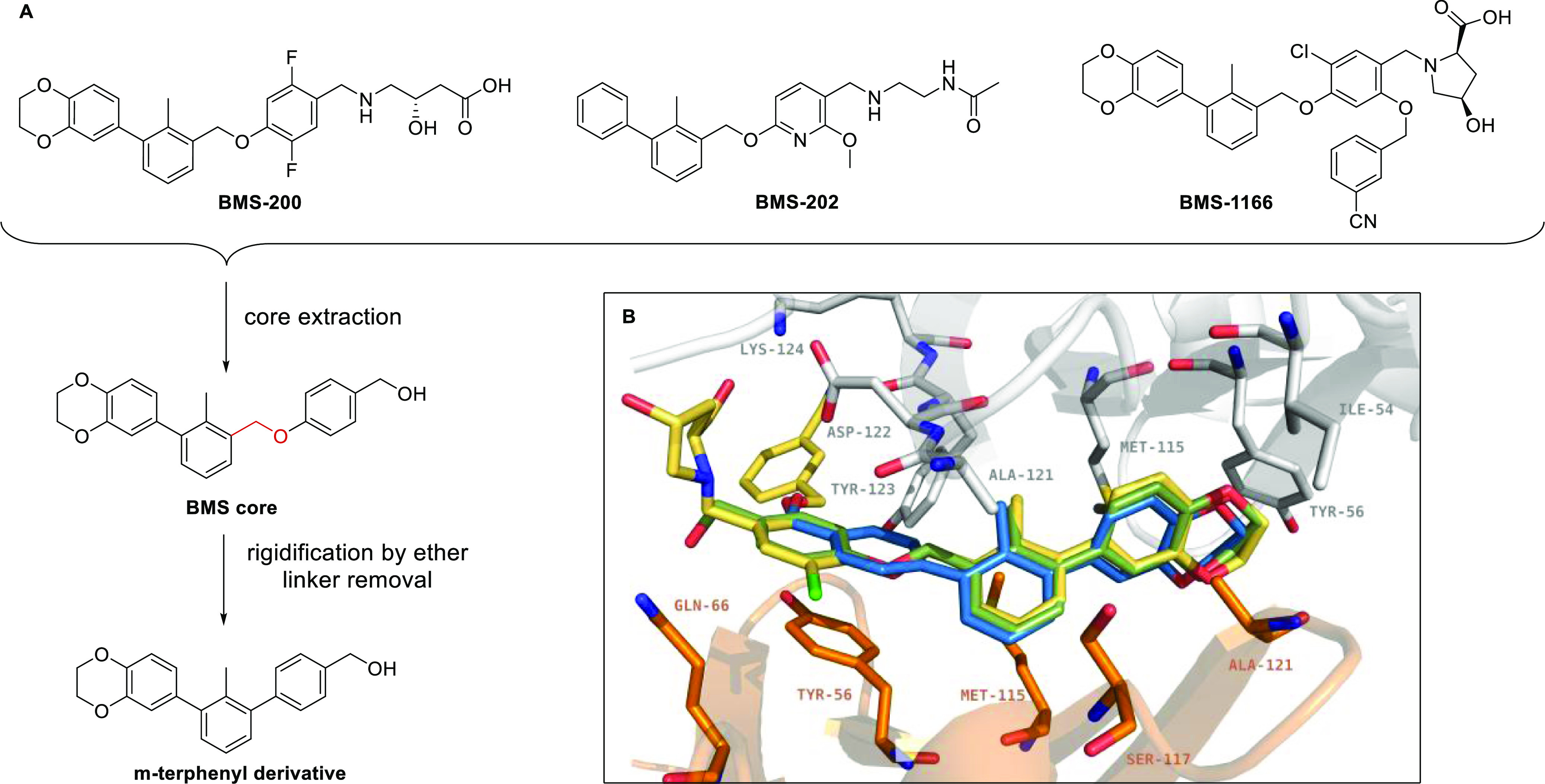
*In silico* comparison of the prototype *m*-terphenyl fragment and corresponding **BMS core** performed with AutoDock Vina integrated into PyRx software. (A) Structures used in molecular modeling: known PD-L1 inhibitors **BMS-200**, **BMS-202**, and **BMS-1166** molecules; **BMS core**—fragment extracted by comparison of the structures of several BMS molecules; ***m*-terphenyl derivative**—rigidified motif obtained by the ether linker removal. (B) Imposition of the modeled complexes of found ***m*-terphenyl derivative** (blue), **BMS core** (green), and whole **BMS-1166** structure (yellow) in complex with PD-L1 (subunit A—gray, subunit B—orange, PDB ID: 6R3K).

Rigidification of the scaffold is a well-known strategy for enhancing the selectivity and potency of SMIs that bind to protein targets.^24−27^ In our study, we focused on the effect of rigidifying the known PD-L1 inhibitors from the BMS family^19,21^ (Figure 1A). The core scaffold was extracted by comparison with several BMS molecules disclosed in patents^19,21^ (Figure 1A, **BMS core**). Rigidification was then implemented by removing the ether linker (highlighted in red), leading to the ***m*-terphenyl derivative** (Figure 1A). The rigidified terphenyl structure was tested *in silico* against the aromatic core of **BMS** molecules (Figure 1B) using AutoDock Vina^28^ integrated into PyRx software,^29^ with the dimeric structure of PD-L1 bound to **BMS-1166** (PDB ID: 6R3K). Molecular docking shows the high potency of the designed, rigidified core, with a stronger PD-L1 binding affinity of 1.0 kcal/mol (−11.7 kcal/mol) than the corresponding fragment of **BMS core** (Figure 1A, −10.7 kcal/mol). Moreover, the calculations suggest that the deletion of the ether linker fragment allows for a better stabilization of the complex, mainly by a stronger π–π stacking with _B_Tyr56 (Figure 1B). These promising results prompted us to structurally optimize the found *m*-terphenyl motif. All designed molecules were tested in a homogeneous time-resolved fluorescence (HTRF) assay to assess their activity as inhibitors of the PD-1/PD-L1 complex (Table S1).

**Scheme 1:**
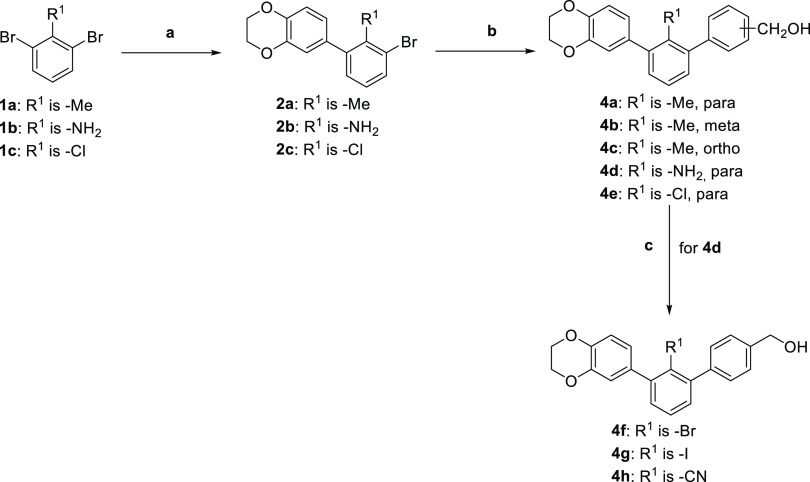
Synthesis Pathway Used in R^1^ and Hydroxymethyl Group Position Optimization of *m*-Terphenyls **4a–4h** Reagents and conditions: (a) **1a–c**, 4-benzodioxane-6-boronic acid, Pd(dppf)Cl_2_·DCM, K_2_CO_3_, dioxane/water 2:1, 80 °C, 3 h, 47–61%; (b) **2a–c**, (hydroxymethyl)phenylboronic acid, Pd(dppf)Cl_2_·DCM, K_2_CO_3_, dioxane/water 2:1, 80 °C, 3 h, 32–56%; (c) for **4f**: **4d** (R^1^ = NH_2_), HBr, NaNO_2_, CuBr_2_, 70 °C, 2 h, 9%; for **4g**: **4d** (R^1^ = NH_2_), HCl, NaNO_2_, KI, RT, 16 h, 28%; for **4h**: **4d** (R^1^ = NH_2_), HCl, NaNO_2_, CuCN, KCN, RT, 4 h, 21%.

In the first step of the process, the impact of the type of the C-2′-attached substituent (R^1^), as well as the position of the hydroxymethyl group, for the binding affinity was tested (Scheme 1, Table 1).

**Table 1:**
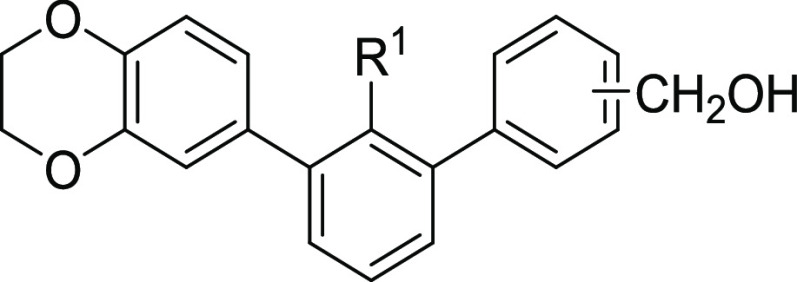
Inhibitory Activities of the Optimized Terphenyls Obtained in the HTRF Assay at the First Stage of Optimization of the R^1^ Substituent

name	R^1^	hydroxymethyl position	HTRF % of the undissociated complex at 5 μM	IC_50_ [μM]
**4a** (*m*-terphenyl)^a^	Me	para	42	5.52 ± 0.04
**4b**	Me	meta	88	
**4c**	Me	ortho	98	
**4d**	NH_2_	para	73	
**4e**	Cl	para	1	0.51 ± 0.01
**4f**	Br	para	15	0.95 ± 0.02
**4g**	I	para	56	
**4h**	CN	para	21	1.17 ± 0.12

a **4a** molecule is modeled terphenyl core fragment presented in Figure 1 and labeled there as ***m*-terphenyl derivative**.

To obtain the designed molecules, we applied a synthetic pathway based mainly on the Suzuki cross-coupling reaction. Appropriate 1,3-dibromoaryls **1a–c** were coupled into their 3-benzodioxane derivatives **2a–c**. Subsequently, biphenyls were taken into a second cross-coupling reaction with 2, 3, or 4-(hydroxymethyl)phenylboronic acid, leading to the corresponding ***m*-terphenyl derivatives 4a–e**. C-2′ nitrile and brominated and iodinated molecules (compounds **4f–h**) were synthesized by the Sandmeyer reaction, followed by amine-based diazonium salt formation. All compounds generated in this step had their potency for the PD-1/PD-L1 complex dissociation tested using the HTRF assay in the scouting mode at 5 μM since IC_50_ determination would not be possible for some compounds due to their low solubility at this stage. In this assay, a lower percentage of the undissociated complex reported in Table 1 indicates higher potency of the tested compound. Full IC_50_s of several compounds were determined (Table 1) and compared with the corresponding percentages of the undissociated complex, indicating a correlation between the two measurements. This approach was used for the activity estimation of all molecules presented in this article. The HTRF assay was also validated on the **BMS-1166** compound, yielding IC_50_ of 3.89 nM (Table 4) as compared to 1.40 nM reported in the literature.^19,30^ The results of the HTRF assay show clearly that the optimal substituent attached to C-2′ of the middle ring of terphenyl is chlorine with IC_50_ of approximately 500 nM (compound **4e**); however, comparable results have been obtained for brominated and cyano derivatives (compounds **4f** and **4h**, respectively). Introduction of a polar fragment (compound **4d**) in this region has a negative impact on the molecule affinity. Generally, the insertion of the halogen atom at the C-2′ position (the R^1^ substituent) is preferred and the activity of the tested fragments decreases with the growth of the halogen van der Waals radius (compounds **4e–g**).

**Scheme 2:**
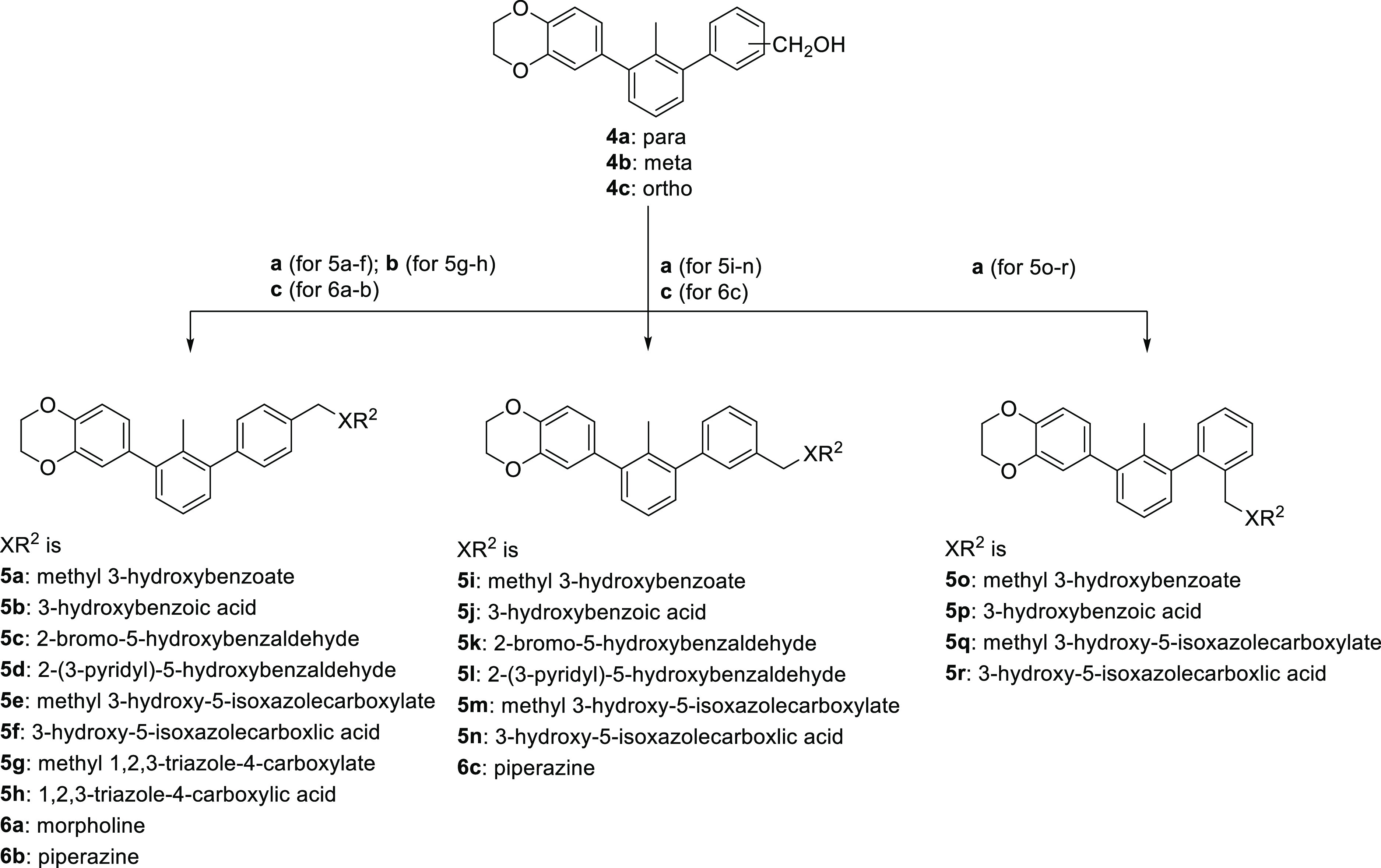
Synthesis Routes Leading to Elongated ***m*-Terphenyl Derivatives 5a–5r** and **6a–6c** Reagents and conditions: (a) (i) **4a–4c**, SOCl_2_, DCM, cat. DMF, RT, 2H; (ii) appropriate phenol, K_2_CO_3_/Cs_2_CO_3_, KI, DMF, 80 °C, 16 h, 42–85%; (iii) **5a**/**5e**/**5i**/**5m**/**5o**/**5q**, LiOH, dioxane/H_2_O, 2:1, RT, 16 h, 45–89% or **5c**/**5k**, 3-pyridinyl boronic acid, Pd(dppf)Cl_2_·DCM, K_2_CO_3_, dioxane/water 2:1, 80 °C, 5 h, 80–92%; (b) (i) **4a**, MsCl, DCM, Et_3_N, RT, 2H (ii) NaN_3_, DMF, RT, 6 days; (iii) methyl propiolate, sodium ascorbate, CuSO_4_, RT, 24 h, 72%; (iv) **5g**, LiOH, dioxane/H_2_O, 2:1, RT, 16 h, 61%; (c) (i): **4a** or **4b**, SOCl_2_, DCM, cat. DMF, RT, 2H (ii) appropriate amine, THF, RT, 48 h, 29–70%.

In parallel, we assessed the influence of further molecular elongation on both the aromatic and aliphatic fragments with different hydrogen bond donor–acceptor properties (Scheme 2, Table 2). Molecules **5a–r** were obtained following a nucleophilic substitution reaction of the previously prepared benzyl chloride derivatives. The corresponding alkyl chlorides were synthesized by the transformation of the 4-hydroxymethylterphenyl derivatives **4a–c** with SOCl_2_ and a catalytic amount of anhydrous dimethylformamide (DMF). Then, they were treated with an appropriate nucleophile, yielding ethers or tertiary amines (compounds **5a–r** and **6a–c**). The double substitution of the amines was prevented by using an excess of the nucleophile. Furthermore, the bulkiness of the terphenyl fragment additionally precluded double substitution.

**Table 2:**
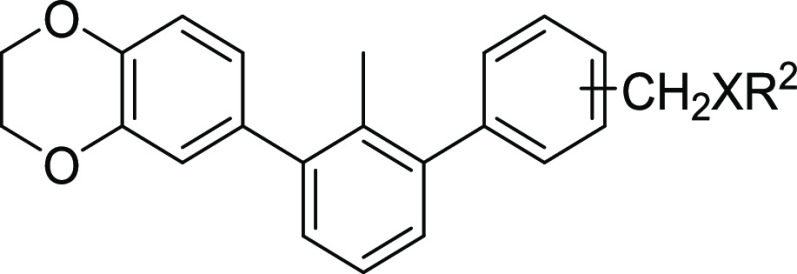

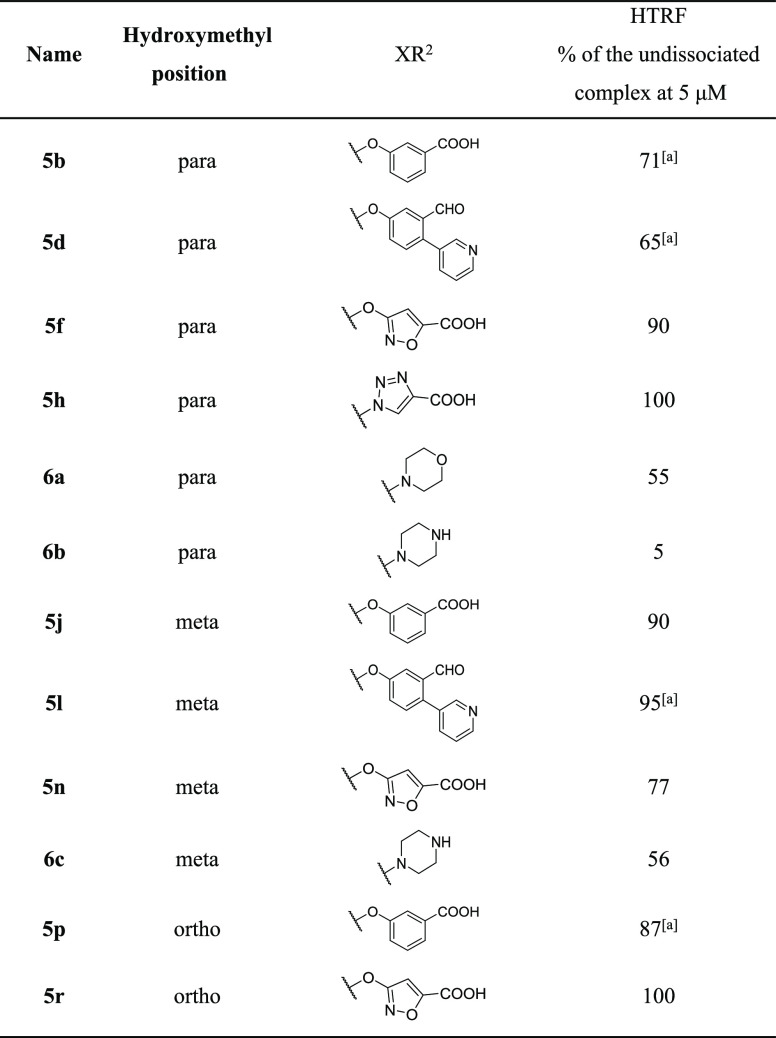
Inhibitory Activities of the Optimized Terphenyls Obtained in HTRF Assay in the First Stage of Optimization of the XR^2^ Substituent and Position of Molecule Extension

a Percentage of the undissociated protein complex was determined at 50 μM concentration of the inhibitor.

The results obtained from these steps clearly indicated that only the para elongation, using amine as a building block is beneficial for the interaction (compound **6b**, Table 2). Attempted meta extension of the motif causes a significant drop of the affinity from 5% of the undissociated complex for **6b** to about 56% for **6c**, tested at 5 μM concentrations. Furthermore, the introduction of an aromatic fragment in each position destabilizes the interaction.

**Scheme 3:**
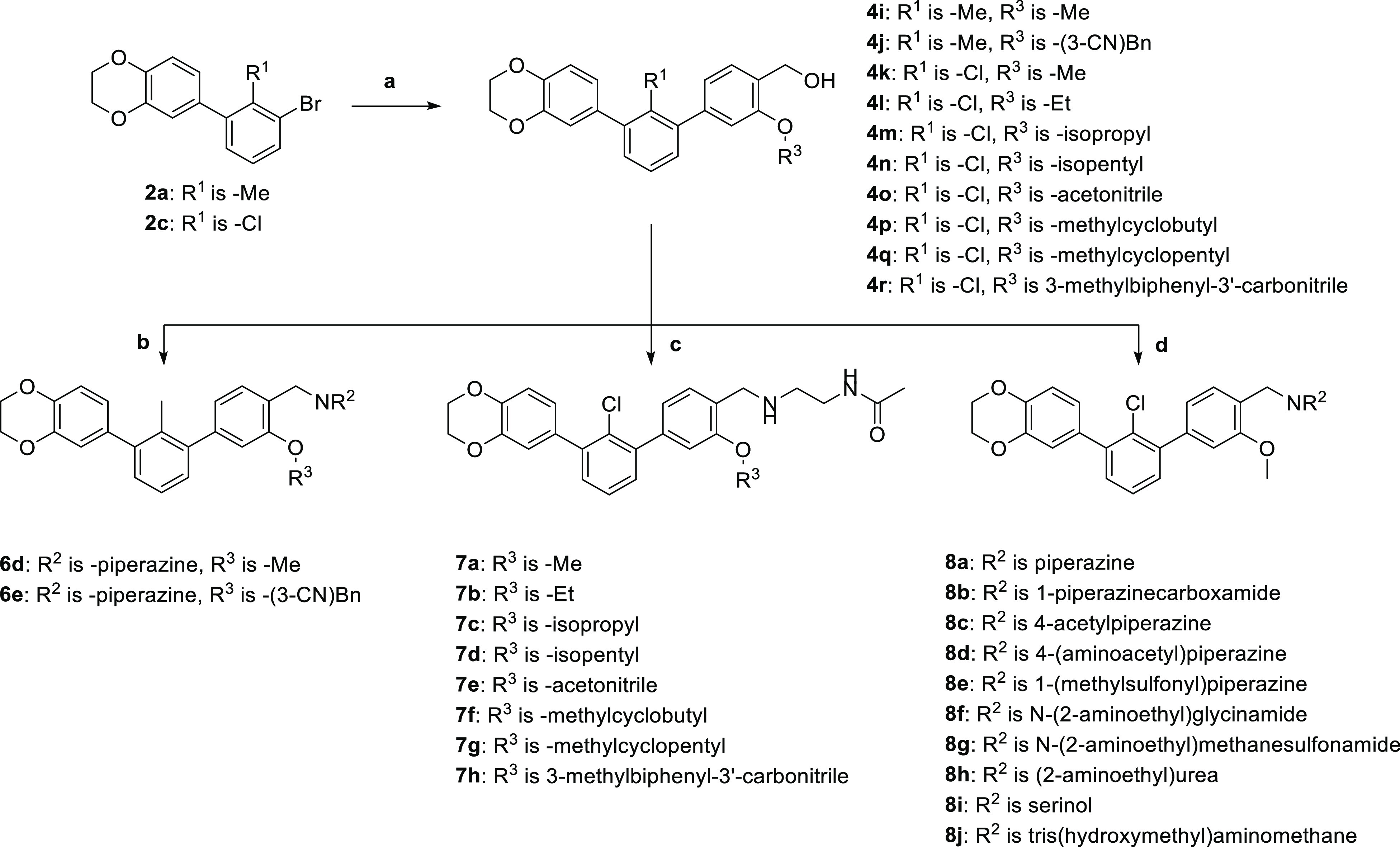
Synthetic Pathway Used during R^3^ Alkoxy Fragment and R^2^ Hydrophilic Tail Optimization of *m*-Terphenyls **6d–6e**, **7a**–**7h**, and **8a–8j** Reagents and conditions: (a) **2a** or **2c**, borane **3a–3i**, Pd(dppf)Cl_2_·DCM, K_2_CO_3_, dioxane/water 2:1, 80 °C, 3 h, 29–89%; (b) (i) **4i** or **4j**, SOCl_2_, DCM, cat. DMF, RT, 2H; (ii) appropriate amine, THF, RT, 48 h, 20–38%; (c) (i) **4k–4r**, SOCl_2_, DCM, cat. DMF, RT, 2H; (ii) appropriate amine, DIPEA, DMF, 80 °C, 16 h, 10–78% (d) (i) **4k**, SOCl_2_, DCM, cat. DMF, RT, 2H (ii) appropriate amine, DIPEA, DMF, 80 °C, 16 h, 33–89%.

In the next stage of the structure optimization, the type of the C3-attached ether substituent in the proximal phenyl ring of the terphenyl was considered. We synthesized a series of different alkoxy derivatives (Scheme 3, Table 3) implementing different-length alkyl and cycloalkyl substituents, as well as an acetonitrile fragment (compounds **6d–e** and **7a–h**).

**Table 3:**
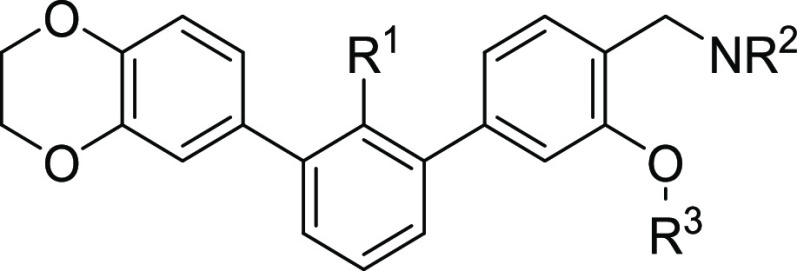

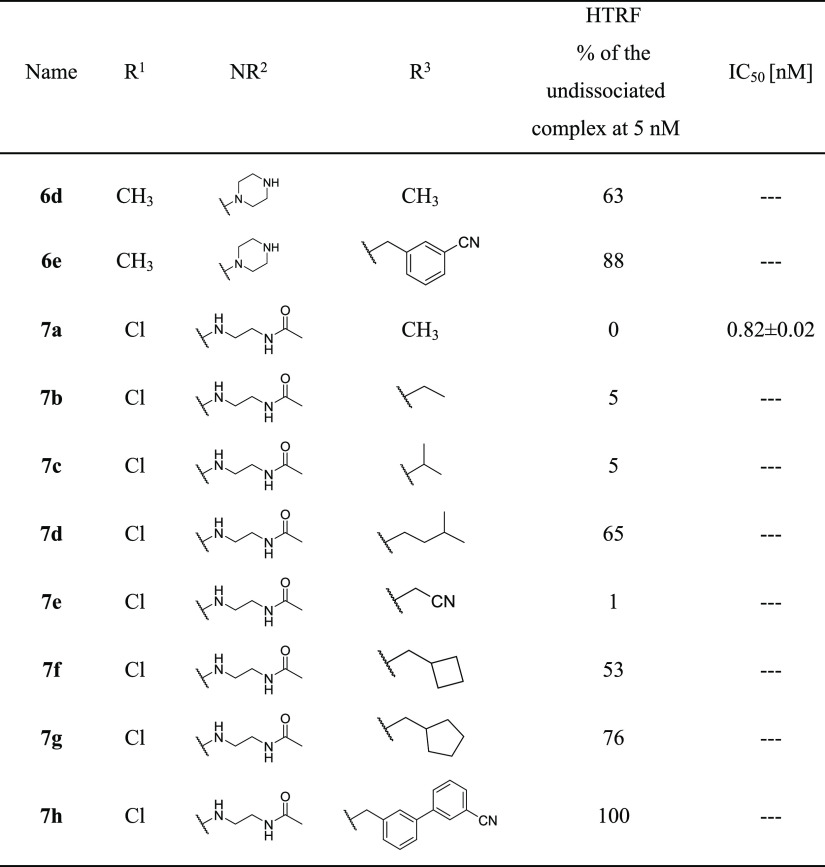
Inhibitory Activities of the Optimized Terphenyls Obtained in HTRF Assay in the Second Step of Optimization of the R^3^ Alkoxyl Substituent

To prepare the desired compounds, we carried out Williamson alkylation of the 4-bromo-2-hydroxybenzyl alcohol, leading to the appropriate alkoxy derivative. Then, the intermediates were transformed into the corresponding phenylboronic acid pinacol esters (compounds **3a–i**, **SI**) and coupled with 3-bromobiphenyls **2a** or **2c**. Hydroxymethyl groups of terphenyls were then transformed into the corresponding chlorides, which were used as an amine alkylating agent. Initially, we tested the piperazine-extended derivatives of the ethers **6d–e**, but we have observed precipitation during the HTRF assay. This forced us to change the amine to *N*-(2-aminoethyl)acetamide, significantly increasing the solubility in the aqueous buffer for compounds **7a–h**. Almost all molecules obtained in this stage of the optimization were characterized by high affinity to the PD-L1 protein and caused complete dissociation of the complex at 5 μM. Therefore, to differentiate potencies of the tested molecules, the inhibitor concentration was set to 5 nM in further assays.

Introduction of the methoxy fragment in **7a** has the greatest impact on binding to PD-L1, resulting in complete complex dissociation at 5 nM ligand concentration and a measured IC_50_ value of 0.82 nM. Further elongation of the ether substituent decreases the affinity to the target (compounds **7b–g**). Addition of an extra biphenyl motif in compound **7h** caused a drop in the inhibitory potency of the molecule. The acetonitrile derivative in molecule **7e** shows the activity comparable with the O-methylated **7a** molecule, causing 99% of the complex dissociation at 5 nM.

The last step of the SAR analysis was focused on the optimization of the hydrophilic tail (Scheme 3, Table 4). Maintaining all optimized molecule fragments, we synthesized and evaluated the inhibitory activities of the piperazine- (compounds **8a–e**), ethylenediamine- (compounds **7a**, **8f–h**), and 2-aminoethanol-containing (compounds **8i–j**) terphenyl derivatives.

**Table 4:**
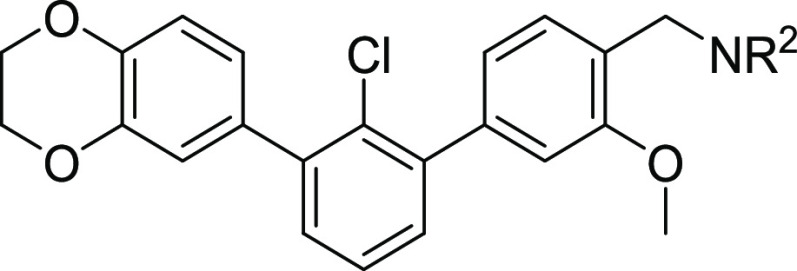

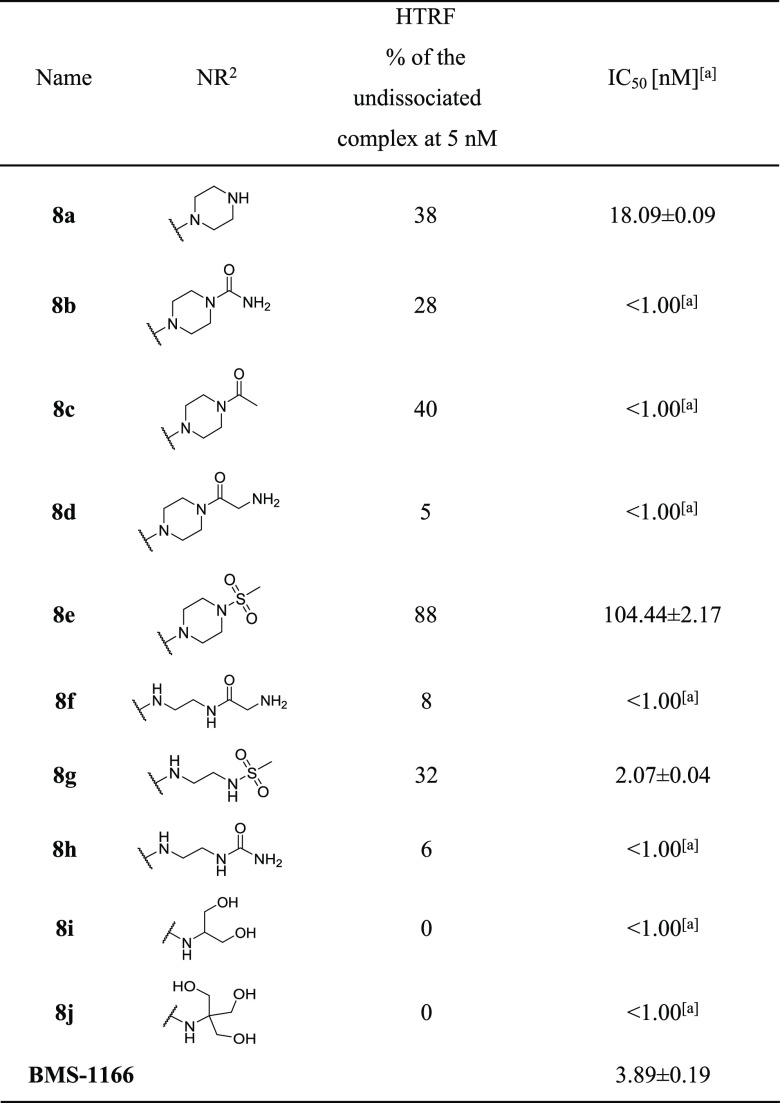
Inhibitory Activities of the Optimized Terphenyls Obtained in HTRF Assay in the Second Step of Optimization of Hydrophilic Tail NR^2^

a Limit of quantification of the used assay was achieved.

The results showed that the type of solubilizer used has a great impact on the affinity to the targeted protein. The piperazine derivative family (compounds **8a**–**8e**) was characterized by the lowest potencies in disrupting the PD-1/PD-L1 interaction, characterized by 88–5% undissociated complex at 5 nM ligand concentration. The best PD-L1 binder from this group is the glycine-containing **8d** molecule with 5% of the remaining complex. For **8b–d**, we could not precisely determine the values of the complex dissociation as they are beyond the quantification limits of the assay. Therefore, we assigned them the values below 1 nM, the level at which we can still confidently determine the assay results. The ethylenediamine derivatives (compounds **7a** and **8f–h**) were characterized by moderate to excellent potencies in the inhibition of the PD-1/PD-L1 interactions, with the percentage of the undissociated complex in 32–0% range at 5 nM inhibitor concentration. Determination of the half-maximal inhibitory concentration was successful only for **7a** and **8g**. The values of 0.82 and 2.07 nM, respectively, were obtained. For the rest of the tested molecules, the limit of the assay was exceeded. It has to be noted that HTRF results served only the purpose of the initial selection of “leads” for further cell-based assays and as such should not be considered as a sole determinant of the compound potency in the PD1-/PD-L1 complex formation inhibition.

### Terphenyl-Based Inhibitors Induce Dimerization of PD-L1

**Figure 2:**
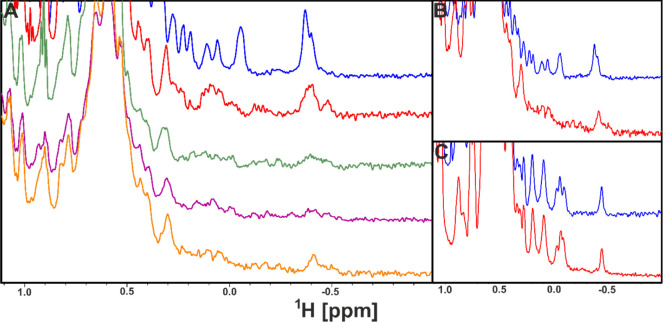
^1^H NMR spectra of PD-L1 with terphenyl-based inhibitors. (A) ^1^H NMR spectra of the aliphatic part of human PD-L1 protein (blue) with compound **4a** (red), **7a** (green), **7e** (purple), and **8j** (orange) in the molar ratio 1:1. (B,C) Aliphatic region of PD-L1 (blue) with **8j** (red): human PD-L1 (panel B) and mouse PD-L1 (panel C) in the molar ratio 1:1 and 1:10, respectively.

Representative molecules from the optimized group were tested for the interaction with PD-L1 using a ^1^H NMR titration experiment and showed protein oligomerization upon addition of the ligand (Figure 2).

After adding compounds **4a**, **7a**, **7e**, and **8j** to human PD-L1 (hPD-L1), we observed the broadening of the hPD-L1 proton signals in 1D NMR spectra, characteristic of oligomerization of hPD-L1, previously observed by us with the BMS compounds.^30^ We additionally compared the spectra of the interaction of **8j** with hPD-L1 (Figure 2B) and murine PD-L1 (mPD-L1) (Figure 2C). Only for human PD-L1, changes in NMR were observed upon addition of **8j** to the protein. For murine PD-L1, no interaction was observed between mPD-L1 and the compound, even with the over-titration of mPD-L1 with **8j** (1:10, molar ratio, respectively) as there was no change in the corresponding NMR spectra fingerprints (changes in the intensity of the peaks or their position compared to each other). The results are, therefore, consistent with our previous studies, in which we showed that the biphenyl moiety from anti-PD-L1 inhibitors binds to human PD-L1 and not to the murine analogue.^31^

### Lead Compounds Activate Jurkat Effector Cells in the ICB Assay

**Figure 3:**
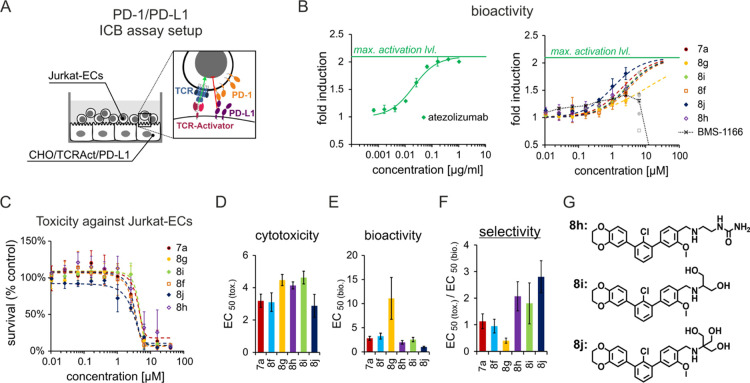
Terphenyl compound bioactivity and toxicity in the ICB assay, (A) schematic representation of the ICB assay. (B) Dose–response curves of the reactivation of Jurkat-ECs with PD-L1-blocking agents: atezolizumab (left panel) and terphenyl compounds (right panel). Graphs show fold induction of the luminescence signal relative to either untreated (for atezolizumab) or dimethyl sulfoxide (DMSO)-treated (for compounds) cells. Data points represent mean ± SD values from 3 to 5 independent experiments. Gray data points were removed from curve-fitting analysis due to the toxicity of the compounds observed at the 10 μM concentration. (C) Long-term (48 h) cytotoxicity of the tested compounds toward Jurkat-ECs. The graph shows Jurkat-EC survival relative to DMSO-treated control cells. Data points represent mean ± SD values from three independent experiments. (D) Half-maximal effective concentrations (EC_50_) of the cytotoxicity of the compounds, calculated from (C). (E) EC_50_ values of the bioactivity of the compounds in the ICB assay, calculated from (B). (F) Selectivity of the bioactivity of the compounds in the ICB assay over the cytotoxicity effect toward Jurkat-ECs. Error bars are combined errors of EC_50 (tox.)_ and EC_50 (bio.)_. (G) Chemical structures of the three most potent terphenyl compounds.

After a successful optimization of the terphenyl compounds, the activity of the optimized molecules **7a** and **8a–j** in a cellular context was assessed. To this end, we first performed the PD-1/PD-L1 ICB assay. In this assay, T-cell receptor (TCR)-mediated activation of Jurkat effector cells (Jurkat-ECs) is reduced by the presence of PD-L1, exposed at the surface of CHO/TCRAct/PD-L1 cells (Figure 3A). Upon the addition of PD-1/PD-L1 blockers, full activation of Jurkat-ECs is restored, as presented for a therapeutic anti-PD-L1 antibody, atezolizumab (Figure 3B).

Most of the compounds selected for the assay (molecules **7a**, and **8f–8j**) were able to increase the activation of Jurkat-ECs (Figure 3B). Only the piperazine-containing molecules do not show any changes after the treatment. The best activity, reflected by the lowest EC_50_ values (Figure 3E), was observed for **8j**, followed by **8h**, **8i**, **7a**, and **8f**. These compounds provided higher activation of the Jurkat-ECs than the previously published **BMS-1166** compound (Figure 3B). All active compounds were comparable in terms of toxicity toward Jurkat-ECs, with compounds **7a**, **8f**, and **8j** slightly less cytotoxic than **8g**, **8h**, and **8i** (Figure 3C,D). Importantly, none of the tested compounds was able to increase the activity of Jurkat-ECs where PD-L1 was absent (Figure S1). This provides evidence that the activity of the compounds is PD-L1-dependent and is not related to the unspecific activation of T cells. Compounds **8j**, **8h**, and **8i** (Figure 3G) showed the highest selectivity of the desired PD-1/PD-L1-blocking activity versus cytotoxicity (Figure 3F). Compounds **8h** and **8j**, which, apart from their bioactivity, showed dissociation of the protein complex in HTRF at 5 nM, were directed for further study.

### Lead Compound **8j** Activates Primary T Cells in the T-Cell Activation Assay

A standard ICB assay relies on artificial Jurkat-ECs, which are surrogates of T-cells. To verify the potential of the compounds to reactivate the PD-L1-blocked primary T cells, a modified assay termed T-cell activation (TCA) was carried out.^32^ In this assay, human primary peripheral blood mononuclear cells (PBMCs) are allowed to make contact with CHO/TCRAct/PD-L1 cells alone or in the presence of PD-1/PD-L1 blockers, and the activation of CD4^+^ and CD8^+^ T cell is monitored by flow cytometry.

**Figure 4:**
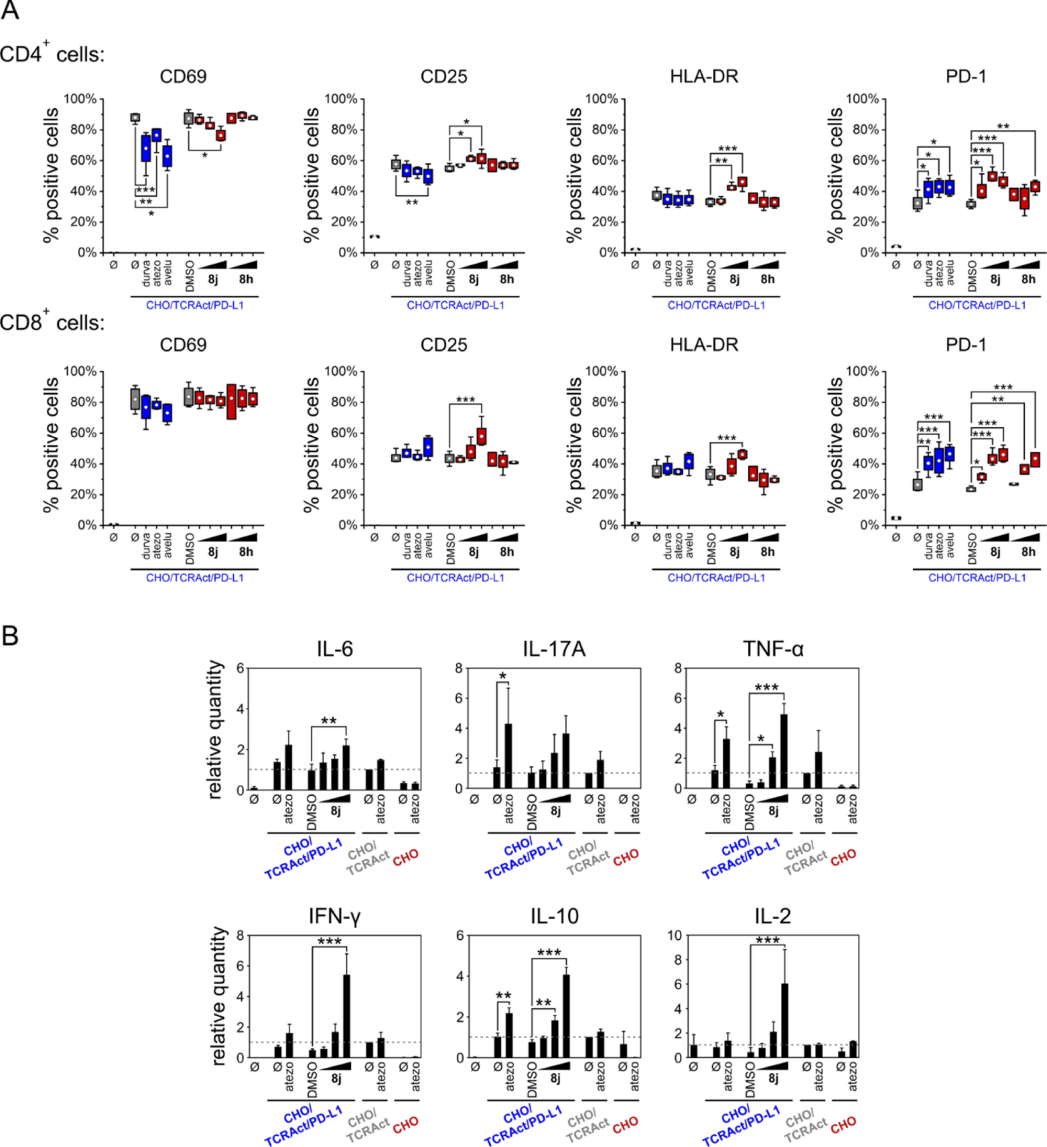
Effect of **8j** and **8h** on the reactivation of primary T-cells preblocked by PD-L1-overexpressing cells. (A) Expression of early (CD69), intermediate (CD25, HLA-DR), and late (PD-1) T-cell activation/exhaustion markers was determined by flow cytometry. PBMCs from healthy donors were exposed to CHO/TCRAct/PD-L1 cells for 2 days in the presence of durvalumab (durva), atezolizumab (atezo), avelumab (avelu), or increasing concentrations of **8j** and **8h** (concentrations: 0.1, 0.5, or 2.5 μM). *ø* indicates untreated cells. The graphs show fractions of positive cells and represents cumulative data from four donors. Statistical significance was analyzed with one-way ANOVA, followed by Fisher’s posthoc test: *p < 0.05, **p < 0.01, ***p < 0.01. (B) Accumulation of the indicated cytokines in culture media from the TCA assay. The graphs show mean ± standard error of the mean values from four independent donors. For each donor, the data were normalized to untreated coculture of PBMCs and CHO/TCRAct cells as a determinant of donor-dependent cytokine level independent of the PD-L1/PD-1 axis. Statistical significance was analyzed with one-way ANOVA, followed by the Fisher posthoc test: *p < 0.05, **p < 0.01, ***p < 0.001.

Following the addition of PBMCs to the CHO/TCRAct/PD-L1 cells, the activation of both CD4^+^ and CD8^+^ T cells was observed, as monitored by the expression of early (CD69), intermediate (CD25 and HLA-DR), and late (PD-1) activation/exhaustion markers (Figure 4A). This is due to the TCR-dependent activation of T-cells by CHO/TCRAct/PD-L1 cells. As reported before, the addition of PD-1/PD-L1-blockers in such a setup increases the expression of PD-1 on T-cells but rarely other markers.^32^ In the current experiment, this was confirmed for three therapeutic anti-PD-L1 antibodies, durvalumab, atezolizumab, and avelumab (Figure 4A). Also, for the tested compounds **8h** and **8j**, a significant increase in the numbers of the PD-1-positive cells was observed (Figure 4A). Surprisingly, for the antibodies, a decrease of CD69 and CD25 expression was observed for CD4^+^ cells, which has not been observed before in our studies. The addition of compound **8j**, but neither **8h** nor the therapeutic antibodies, increased the numbers of activated, CD25-positive, and HLA-DR-positive T-cells (Figure 4A). Altogether, the presented data clearly show the biological activity of **8j** in a cellular context.

In addition to the expression of surface markers, the accumulation of immune-related cytokines in the culture media collected in the TCA assay was monitored with the cytometric bead array (CBA) human Th1/Th2/Th17 kit. In the presence of atezolizumab, significantly increased levels of IL-17A, TNF-α, and IL-10 were observed (Figure 4B). Also, the levels of IL-6, IFN-γ, and IL-2 increased, but these increases were not statistically significant. Similarly, the treatment of PBMCs cocultured with the CHO/TCR-Act/PD-L1 cells with **8j** also increased the accumulation of all six cytokines, with significant increases observed for IL-6, TNF-α, IFN-γ, IL-10, and IL-2 (Figure 4B).

### Distal Terphenyl Ring Provides Rigidification That Fits into the PD-L1 Cleft

**Figure 5:**
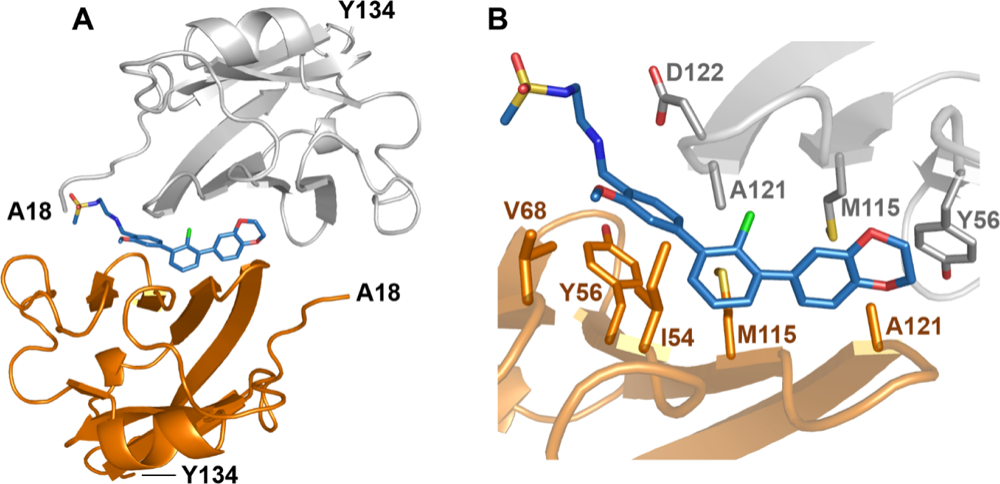
Cocrystal X-ray structure of **8g** bound to the Ig-like V-type domain of human PD-L1 (PDB: 7NLD). (A) Arrangement of the molecules in the crystal structure—two PD-L1 molecules (gray and orange) form a single pocket accommodating **8g** compound (blues). (B) Detailed interactions of 8g at the binding cleft of PD-L1. The inhibitor binds at a hydrophobic cavity formed upon PD-L1 dimerization. Color coding as in (A).

We were able to crystalize and solve the structure of the **8g** compound in complex with PD-L1 at the resolution of 2.3 Å that allowed us to decipher the molecular basis of the interactions between them (Figure 5 and Table S2). The asymmetric unit of the hPD-L1/**8g** complex contains six protein molecules organized into three dimers, with one inhibitor located at the center of the interface of each dimer. The electron density map describes each inhibitor molecule allowing the position of all moieties in the structure with the fully detailed density for the molecule located at the interface of the AB dimer. **8g** is mostly buried in the deep and elongated tunnel, which is composed of two PD-L1 monomers. The 1,4-benzodioxane group creates the standard interaction with _A_Tyr56 (the monomer molecules are annotated by subscripts A and B according to their chain arrangement in the crystal structure of the dimer), previously observed by us for the **BMS-202**, **BMS**-**1001**, and **BMS-1166** compounds.^30,33^ The central terphenyl moiety is stabilized by plenty of hydrophobic contacts with _B_Tyr56, _A_Met115, _B_Met115, _A_Ala121, and _B_Ala121. The chlorine substituent attached to this moiety forms an additional halogen bond with the backbone carbonyl of _A_Asp122, providing the rationale for the enhanced inhibitory effect of these halogenated inhibitors at the R^1^ position. The methoxy substituent at the meta position of the distal ring of the terphenyl is also additionally stabilized by hydrophobic interaction with _B_Ile54 and _B_Val68, pointing out the importance of this substituent. At the other side of the tunnel, the sulfonamide polar extension forms a hydrogen bond with the carbonyl of the _A_Asp122 side chain and is largely water-exposed.

Our structural data confirm that the terphenyl compounds with the rigidified biphenyl unit bind to PD-L1 at the interaction site that has been seen in the compounds with the biphenyl scaffold. Comparison of the PD-L1/**8g**, PD-L1/**BMS-1166**, PD-L1/the C2-symmetric inhibitor, and PD-L1/**ARB-272572** complexes shows that the crystallographic arrangement of the terphenyl core is close to the docking results (Figure S2).^33−35^

## Conclusions

In this paper, we have demonstrated a new group of small-molecule anti-PD-L1 inhibitors based on the concept of rigidifying the biphenyl unit by removing the ether linker from the known PD-L1 inhibitors of the BMS family. Such terphenyl-based scaffolds exhibit high inhibitory activity against the PD-1/PD-L1 complex both in common biophysical assays, such as HTRF and NMR, as well as cell-based assays. Our most potent compounds activated Jurkat-ECs to levels comparable to those of the control antibodies, despite being considerably smaller. Similar to other anti-PD-L1 inhibitors reported in the literature, our compounds dimerize human PD-L1, as presented here by NMR and X-ray crystallography studies. They are specific for human PD-L1 and do not cross-react with the murine PD-L1 analogue. Our most potent compound, **8j**, is characterized by an exceptionally high affinity to the molecular target (IC_50_ < 1 nM), as well as a proven potency in cell-based assays with an EC_50_ of ca. 1 μM. Most importantly, adding compound **8j** to the coculture of PBMCs and CHO/TCRAct/PD-L1 cells not only induced PD-1 expression but also increased the numbers of CD25-positive and HLA-DR-positive T cells, which was not observed even in PD-L1 therapeutic antibodies. Also, the accumulation of immune-related cytokines was increased in the presence of either **8j** or atezolizumab. This shows the potency of this compound toward primary human T cells and designates **8j** as an outstanding candidate for further optimization in the anti-PD-L1 treatments of cancer. The presented crystal structure provides an explanation for the enhanced inhibitory effect of terphenyl-based inhibitors. The introduced phenyl ring replaces the benzyl linker of **BMS-1166** and thus provides the rigidification of the structures in the conformation that fits perfectly into the PD-L1 cleft.

## Experimental Procedures

### Homogeneous Time-Resolved Fluorescence

HTRF was performed using the certified CisBio assay according to the manufacturer’s guidelines. The experiments were performed at 5 nM h-PD-L1 and 50 nM hPD-1 in the final formulation at 20 μL final volume in the well. To determine the half-maximal inhibitory concentration (IC_50_) of tested compounds, measurements were performed on two individual dilution series, unless states otherwise. After mixing all components according to the CisBio protocol, the plate was left for 2 h incubation at room temperature, followed by time-resolved fluorescence energy transfer (TR-FRET) measurement on Tecan Spark 20M. Collected data were background-subtracted on the negative control, normalized on the positive control, averaged, and fitted with normalized Hill’s equation to determine the IC_50_ value using *Mathematica 12*.

### Protein Nuclear Magnetic Resonance

NMR spectra were recorded in PBS pH 7.4 containing 10% (v/v) of D_2_O added to the samples to provide the lock signal. Water suppression was carried out using the WATERGATE sequence. All the spectra were recorded at 300 K using a Bruker AVANCE 600 MHz spectrometer with the cryo-platform. The spectra were recorded at the ligand/protein ratio of 1:1, unless stated otherwise. The samples were prepared by adding small amounts of a 50 mM ligand stock solution in DMSO to the protein solution (0.20 mL) of PD-L1 fragment at a concentration of 0.2 mM. Spectra were visualized using TopSpin 4.0.2.

### *In Silico* Molecular Docking

For molecular docking, we generated structures of **BMS core** and ***m*-terphenyl derivative** in the PDB format using Chem3D 17.1 and used AutoDock Vina1.1.2 integrated into PyRx with the dimeric structure of PD-L1 bound to **BMS-1166** (PDB ID: 6R3K).

### Protein Expression and Purification

*Escherichia coli* strain BL21 was transformed with a pET-21b plasmid carrying the PD-L1 gene (amino acids 18–134). The bacteria were cultured in LB at 37 °C until OD_600nm_ of 0.6 when the recombinant protein production was induced with 1 mM IPTG. The protein production was continued overnight. Inclusion bodies were collected by centrifugation, washed twice with 50 mM Tris-HCl pH 8.0 containing 200 mM NaCl, 10 mM ethylenediaminetetraacetic acid, 10 mM 2-mercaptoethanol, and 0.5% Triton X-100, followed by a single wash with the same buffer but with no Triton X-100. The washed inclusion bodies were resuspended overnight in 50 mM Tris-HCl pH 8.0, 6 M GuHCl, 200 mM NaCl, and 10 mM 2-mercaptoethanol and clarified with centrifugation. Refolding of PD-L1 was performed by dropwise dilution into 0.1 M Tris-HCl pH 8.0 containing 1 M l-arginine hydrochloride, 0.25 mM oxidized glutathione, and 0.25 mM reduced glutathione. The refolded protein was dialyzed 3 times against 10 mM Tris-HCl pH 8.0 containing 20 mM NaCl and purified by size exclusion chromatography using Superdex 75 and dialysis buffer. The quality of the refolded protein was evaluated by sodium dodecyl sulphate–polyacrylamide gel electrophoresis and NMR.

### PD-L1 Cocrystallization, Determination, and Refinement

Purified PD-L1 in 10 mM Tris-HCl pH 8.0, 20 mM NaCl buffer was concentrated to 5 mg/mL, mixed with the **8g** inhibitor in 1:3 molar ratio (protein/compound) and clarified. The supernatant was used for screening using commercially available buffer sets. Diffraction-quality crystals were obtained at room temperature from the condition containing 0.1 M sodium cocodylate pH 6.5, 25% PEG 4000. The crystal was flash-cooled in liquid nitrogen. The X-ray diffraction data were collected at the BL14.1 beamline operated by the Helmholtz-Zentrum Berlin (HZB) at the BESSY II (Berlin Adlershof, Germany).^36^ The data were indexed, integrated, and scaled using XDS, XSCALE, and Aimless.^37−39^ The initial phases were obtained by molecular replacement calculated in Phaser.^40^ The model building was performed in Coot and refinement was performed using Phenix or PDB-REDO server.^41−43^ Water molecules were added automatically and inspected manually. The coordinates and structure factors were deposited in the Protein Data Bank with accession number 7NLD.

### Cell Culture

CHO-K1 cells, CHO-K1 cells overexpressing TCR activator (CHO/TCRAct), PD-L1-expressing CHO/TCRAct cells (CHO/TCRAct/PD-L1), and Jurkat-EC T-cells overexpressing PD-1 and carrying a luciferase reporter gene under the control of the nuclear factor of activated T-cell response element (NFAT-RE) were obtained from Promega and cultured in RPMI-1640 medium (Biowest) supplemented with 10% fetal bovine serum (FBS, Biowest) and 200 mM l-glutamine (Biowest). G418 (250 μg/mL, InvivoGen) and Hygromycin B Gold (50 μg/mL, InvivoGen) were also added to the culture medium as selection antibiotics. The overexpression of PD-L1 and TCR ligand in CHO/TCRAct/PD-L1 cells and overexpression of PD-1 in Jurkat-ECs were confirmed by flow cytometry and western blot analysis. PCR tests for *Mycoplasma* sp. contamination^44^ were routinely performed, which indicated negative results for both cell lines.

### Cytotoxicity Assay

The cytotoxicity of compounds was verified toward Jurkat-ECs since these cells provide the readout in the ICB assay and present a much higher degree of susceptibility to the compounds’ toxicity than CHO-K1 cells (not shown). Jurkat-ECs were seeded on 96-well transparent plates in the presence of increasing concentrations of the compounds with DMSO-treated cells as a control. The concentration of DMSO was kept constant (0.1%). After 48 h of incubation, a tetrazolium reagent, Biolog redox dye MIX MB (Biolog), was added according to the manufacturer’s protocol, and the culture plates were incubated for an additional 2 h (37 °C, 5% CO_2_). The absorbance was measured using a Spark microplate reader (Tecan) at 590 nm with 750 nm as a reference. The data are presented as Jurkat-EC survival relative to DMSO-treated control cells. Data points represent mean ± SD values from three independent experiments. Half-maximal effective concentrations (EC_50_ values) were calculated from the dose–response curve using OriginPro 2020 (OriginLab) software.

### PD-1/PD-L1 ICB Assay

The functional assay of the blockade of PD-1/PD-L1 interaction in vitro (PD-1/PD-L1 Blockade Bioassay, Promega) was performed according to the manufacturer’s protocol. CHO/TCRAct/PD-L1 cells or CHO/TCRAct or CHO-K1 cells were seeded on 96-well white plates at the density of 10,000 cells/well and the next day overlaid with Jurkat T cells (20,000 cells/well) in the presence of increasing concentrations of the compounds with DMSO only as a control (the concentration of DMSO was kept constant at 0.1%) or atezolizumab, as a positive control, anti-PD-L1 monoclonal antibody (Selleckchem). The 2.5-fold dilutions of atezolizumab were prepared in the assay buffer (RPMI 1640 + 1% FBS) on the day of the assay. Activation of the Jurkat T cells, reflected by luciferase activity, was monitored by luminescence measurements after 6 h of incubation (37 °C, 5% CO_2_), and 20 min of additional incubation with the Bio-Glo assay reagent (Promega) at room temperature. The luminescence was read on a Spark microplate reader (Tecan). The data are presented as fold induction of the luminescence signal relative to either untreated (for atezolizumab) or DMSO-treated (for compound) cells. Data points represent mean ± SD values from three to five independent experiments. Half-maximal effective concentrations (EC_50_ values) were calculated from the Hill curve fitting to the experimental data using *Origin Pro 2020* software (OriginLab).

### Isolation of PBMCs and TCA Assay

Anticoagulant citrate dextrose-A-treated blood from healthy donors was purchased from the Regional Center of Blood Donation and Blood Therapy in Krakow, Poland. PBMCs were isolated from whole blood by density gradient centrifugation using Pancoll human separating solution (PAN-Biotech GmbH, Aidenbach, Germany). The separated cells were washed and resuspended in RPMI 1640 medium (Biowest) containing 10% FBS (Biowest). PBMCs were added at an amount of 3 × 10^5^ to CHO, CHO/TCRAct, or CHO/TCRAct/PD-L1 cells preseeded on 24-well plates in the presence of either anti-PD-L1 mAbs (at the final concentration 5 μg/mL): durvalumab (Selleckchem), atezolizumab (Selleckchem), or avelumab (MedChemExpress) or tested compounds at the concentrations 0.1, 0.5, and 2.5 μM. Untreated cells were used as controls for antibody treatment, while DMSO-treated cells [0.1% (v/v)] were used as controls for compound treatment. The cells were incubated for 48 h (37 °C, 5% CO_2_) and then detached from the plates with TrypLe Select Enzyme (Thermo Fisher). PBMCs were stained for 20 min at room temperature with the antibodies: anti-CD4-FITC, anti-CD8-BV510, anti-CD69-APC, anti-CD25-PE, anti-HLA-DR-PerCP, and anti-PD1-PECy7 (Becton Dickinson Biosciences, BD). Following two washing steps, the cells were analyzed with a FACSCanto II cytometer. Data analysis was carried out with FlowJo software, followed by statistical significance calculations done with *Origin Pro 2020* software (OriginLab). Statistical significance was analyzed with one-way analysis of variance (ANOVA), followed by the Fisher’s posthoc test: *p < 0.05, **p < 0.01, ***p < 0.01.

### Analysis of Cytokine Accumulation

The concentration of the cytokines (IL-2, IL-4, IL-6, IL-10, IL-17A, IFN-γ, and TNFα) in supernatants was determined using the CBA Human Th1/Th2/Th17 kit (BD Biosciences), with data acquisition using the FACSCanto flow cytometer. The CBA beads were discriminated in FL-4 and FL-5 channels, while the concentration of specified cytokines was determined by the intensity of FL-2 fluorescence, using the respective standard reference curve and FCAP Array software (BD Biosciences). IL-4 was below the detection level and is not presented in the manuscript. Statistical significance was analyzed with one-way ANOVA using the *Origin Pro 2020* software, followed by the Fisher’s posthoc test: *p < 0.05, **p < 0.01, ***p < 0.01.

### Syntheses

All syntheses were performed according to the procedures summarized in Schemes 1-3 and as described in the Supporting Information. Reagents were obtained from commercial suppliers (Sigma-Aldrich, ABCR, Acros Organics, Appollo Scientific, AK Scientific) and used without further purification unless otherwise noted. The anhydrous solvents were purchased from Sigma-Aldrich or Alfa Aesar. Nuclear magnetic resonance spectra were recorded at 300K on a Bruker AVANCE 600 spectrometer {^1^H NMR (600 MHz) and ^13^C NMR (151 MHz)}. Chemical shifts for ^1^H NMR and ^13^C NMR were reported in parts per million (δ) referenced to the appropriate deuterated solvent. Coupling constants were reported in hertz (Hz). The following abbreviations were used for spin multiplicity: s = singlet, s_broad_ = broad singlet, d = doublet, t = triplet, q = quartet, quin = quintet, dd = double of doublets, ddd = double doublet of doublets, and m = multiplet. IR absorption spectra were recorded on a Nicolet IR200 spectrometer using the attenuated total reflection (ATR) technique. Thin-layer chromatography (TLC) was performed on Sigma-Aldrich precoated silica gel plates (0.20 mm thick, particle size 25 mm). Spots were visualized by UV light at 254 and 365 nm. Column chromatography was performed using silica gel 60 (40–63 mm, Sigma-Aldrich). Flash chromatography was performed on a Reveleris X2 flash chromatography, using Grace Reveleris silica flash cartridges. The liquid chromatography–mass spectrometry (LC–MS) measurements were performed on an ultraperformance liquid chromatography (UPLC)–tandem mass spectrometry system consisting of a Waters ACQUITY UPLC (Waters Corporation, Milford, MA, USA) coupled to a Waters TQD mass spectrometer (electrospray ionization mode ESI-tandem quadrupole). Chromatographic separations were carried out using the Acquity UPLC BEH (bridged ethyl hybrid) C_18_ column, 2.1 × 100 mm and 1.7 μm particle size, equipped with an Acquity UPLC BEH C18 VanGuard precolumn; 2.1 × 5 mm and 1.7 μm particle size. The column was maintained at 40 °C and eluted under gradient conditions using 95–0% of eluent A over 10 min at a flow rate of 0.3 mL min^–1^. Eluent A: water/formic acid (0.1%, v/v); eluent B: acetonitrile/formic acid (0.1%, v/v). The purity of all final compounds determined using chromatographic LC–MS was >95%. HRMS was carried out by the Laboratory for Forensic Chemistry Faculty of Chemistry, Jagiellonian University, with a microOTOF-QII (Bruker) spectrometer using the ESI ionization technique.

### General Procedure for Preparation of 3-Bromobiphenyl Intermediates (**2a–2c**)

A three-necked round-bottom flask was charged with appropriate halide **1a**–**1c**, 1,4-benzodioxane-6-boronic acid, K_2_CO_3_, and dioxane/water mixture (2:1, 7/14 mL for 1 mmol) under an argon atmosphere. The mixture was deoxygenated by rinsing with argon for half an hour; then, the Pd(dppf)Cl_2_ dichloromethane complex was added. The reaction mixture was heated at 80 °C, using preheated bath, for 3 h and after this time, the progression of the reaction was controlled using TLC analysis (SiO_2_, hexane/ethyl acetate, 4:1). When the reaction was complete, water was added and the extraction with ethyl acetate followed. The organic phases were combined, dried over anhydrous MgSO_4_, and evaporated. The crude product was purified by flash chromatography (SiO_2_, hexane/ethyl acetate), giving the final products **2a**–**2c** with 47–61% yield.

#### 6-(3-Bromo-2-methylphenyl)-1,4-benzodioxane (**2a**)

2,6-Dibromotoluene (**1a**) (1.93 mL, 14.0 mmol, 1.4 equiv), 1,4-benzodioxane-6-boronic acid (1.79 g, 10.0 mmol, 1.0 equiv), K_2_CO_3_ (5.53 g, 40.0 mmol, 4.0 equiv), Pd(dppf)Cl_2_ complex with dichloromethane (0.41 g, 0.5 mmol, 0.05 equiv). Product **2a** was obtained as a colorless oil with 52.7% (1.61 g) yield. Compound **2a** was previously described in patent application.^45^

**R**_**f**_ = 0.51 (SiO_2_, hexane/ethyl acetate 2:1); ^**1**^**H NMR** (600 MHz, CDCl_3_): δ [ppm] 7.52 (dd, *J* = 8.0, 1.2 Hz, 1H), 7.14 (dd, *J* = 7.6, 1.1 Hz, 1H), 7.05 (t, *J* = 7.7 Hz, 1H), 6.90 (d, *J* = 8.2 Hz; 1H), 6.79 (d, *J* = 2.1 Hz, 1H), 6.74 (dd, *J* = 8.2, 2.2 Hz, 1H), 4.31 (s, 4H), 2.33 (s, 3H); ^**13**^**C NMR** (151 MHz, CDCl_3_): δ [ppm] 143.6, 143.2, 143.0, 135.7, 135.2, 131.5, 129.2, 126.8, 126.3, 122.5, 118.2, 117.1, 64.6, 21.2; **IR (ATR)** [cm^–1^]: 2927, 2874, 1582, 1505, 1457, 1303, 1276, 1240, 1066, 993, 897, 878, 820, 789, 780; **LC–MS (DAD/ESI)**: *t*_R_ = 9.03 compound did not ionize.

#### 6-(2-Amino-3-bromophenyl)-1,4-benzodioxane (**2b**)

2,6-Dibromoaniline (**1b**) (5.27 g, 21.0 mmol, 1.4 equiv), 1,4-benzodioxane-6-boronic acid (2.70 g, 15.0 mmol, 1.0 equiv), K_2_CO_3_ (8.29 g, 60.0 mmol, 4.0 equiv), Pd(PPh_3_)_4_ (0.87 g, 0.75 mmol, 0.05 equiv). Product **2b** was obtained as a colorless solid with 61.0% (2.80 g) yield. Product **2b** was crystallized from cyclohexane.

**R**_**f**_ = 0.36 (SiO_2_, hexane/ethyl acetate 4:1); ^**1**^**H NMR** (600 MHz, CDCl_3_): δ [ppm] 7.39 (dd, *J* = 8.0, 1.5 Hz, 1H), 7.02 (dd, *J* = 7.5, 1.4 Hz, 1H), 6.95–6.93 (m, 2H), 6.89 (dd, *J* = 8.3, 2.0 Hz, 1H), 6.65 (t, *J* = 7.3 Hz, 1H), 4.30 (s, 4H); ^**13**^**C NMR** (151 MHz, CDCl_3_): δ [ppm] 143.9, 143.3, 141.6, 132.4, 131.7, 129.6, 122.2, 119.0, 118.0, 117.9, 109.9, 64.6, 64.6; **IR (ATR)** [cm^–1^]: 3481,3389, 2932, 2876, 1603, 1504, 1462, 1308, 1239, 1055, 898, 881, 821, 780, 737; **LC–MS (DAD/ESI)**: t_r_ = 7.77 min, calcd for C_14_H_12_BrNO_2_ (*m*/*z*) [M + H]^+^ 306.01; found, [M + H]^+^ 306.10; **HRMS (ESI)**: calcd for C_14_H_12_BrNO_2_ (*m*/*z*) [M + H]^+^ 306.0124; found, [M + H]^+^ 306.0123.

#### 6-(3-Bromo-2-chlorophenyl)-1,4-benzodioxane (**2c**)

1,3-Dibromo-2-chlorobenzene (**1c**) (5.16 g, 19.09 mmol, 1.1 equiv), 1,4-benzodioxane-6-boronic acid (3.12 g, 17.4 mmol, 1.0 equiv), K_2_CO_3_ (9.62 g, 69.6 mmol, 4.0 equiv), Pd(dppf)Cl_2_ complex with dichloromethane (0.71 g, 0.87 mmol, 0.05 equiv). Product **2c** was obtained as a salmon solid with 46.8% (2.65 g) yield.

**R**_**f**_ = 0.38 (SiO_2_, hexane/ethyl acetate 4:1); ^**1**^**H NMR** (600 MHz, CDCl_3_): δ [ppm] 7.59 (dd, *J* = 8.0, 1.6 Hz, 1H), 7.24 (dd, *J* = 7.6, 1.6 Hz, 1H), 7.13 (t, *J* = 7.8 Hz, 1H), 6.93–6.91 (m, 2H), 6.87 (dd, *J* = 8.3, 2.1 Hz, 1H), 4.30 (s, 4H); ^**13**^**C NMR** (151 MHz, CDCl_3_): δ [ppm] 143.5, 143.1, 142.3, 132.9, 132.9, 132.6, 130.2, 127.4, 123.8, 122.5, 118.3, 117.0, 64.5, 64.4; **IR (ATR)** [cm^–1^]: 2978, 2931, 2875, 1506, 1447, 1312; **LC–MS (DAD/ESI**^+^): *t*_R_ = 8.68 compound did not ionize.

### General Procedure for the Suzuki Reaction (**4a–4e** and **4i–4r**)

A three-necked round-bottom flask was charged with appropriate halide **2a**–**2c**, borane **3a**–**3i** (for synthesis of compounds **3a**–**3i**, see Supporting Information), K_2_CO_3_, and dioxane/water mixture (2:1, 5/10 mL for 1 mmol) under an argon atmosphere. The mixture was deoxygenated by rinsing with argon for half an hour, and then, the Pd(dppf)Cl_2_ dichloromethane complex was added. The reaction mixture was heated at 80 °C, using preheated bath, for 5 h. After this time, the progression of the reaction was controlled by TLC. When the reaction was complete, water was added and the extraction with ethyl acetate followed. The organic phases were combined, dried over anhydrous MgSO_4_, and evaporated. The crude product was purified by flash chromatography (SiO_2_, hexane/ethyl acetate), giving the final products **4a–e** and **4i–r** with 29–89% yield.

#### (3′-(Benzo-1,4-dioxan-6-yl)-2′-methyl-[1,1′-biphenyl]-4-yl)methanol (**4a**)

6-(3-Bromo-2-methylphenyl)-1,4-benzodioxane (**2a**) (2.98 g, 9.78 mmol, 1.0 equiv), 4-(hydroxymethyl)phenylboronic acid (2.03 g, 13.30 mmol, 1.2 equiv), K_2_CO_3_ (4.90 g, 35.56 mmol, 3.2 equiv), Pd(dppf)Cl_2_ complex with dichloromethane (0.36 g, 0.45 mmol, 0.04 equiv). Product **4a** was obtained as a colorless solid with 56.4% (1.62 g) yield.

**R**_**f**_ = 0.23 (SiO_2_, hexane/ethyl acetate 2:1); ^**1**^**H NMR** (600 MHz, DMSO-*d*_6_): δ [ppm] 7.39 (d, *J* = 8.3 Hz, 2H), 7.33 (dt, *J* = 8.1, 1.8 Hz, 2H), 7.27 (t, *J* = 7.6 Hz, 1H), 7.17–7.15 (m, 2H), 6.92 (d, *J* = 8.2 Hz, 1H), 6.86 (d, *J* = 2.1 Hz, 1H), 6.82 (dd, *J* = 8.2, 2.1 Hz, 1H), 5.22 (t, *J* = 5.8 Hz, 1H), 4.65 (d, *J* = 5.7 Hz, 2H), 4.28 (s, 4H), 2.07 (s, 3H); ^**13**^**C NMR** (151 MHz, DMSO-*d*_6_): δ [ppm] 143.4, 143.0, 142.8, 142.3, 141.7, 140.7, 135.4, 132.8, 129.4, 129.1, 129.0, 126.8, 126.0, 122.6, 118.2, 117.2, 64.6, 64.5, 63.2, 19.1; **IR (ATR)** [cm^–1^]: 3315, 2930, 1578, 1508, 1318; **LC–MS (DAD/ESI)**: *t*_R_ = 7.36 min, calcd for C_22_H_20_O_3_ [*m*/*z*] [M + H]^+^ 333.15, [M-H_2_O + H]^+^ 315.14; found, [M-H_2_O + H]^+^ 315.28; **HRMS (ESI)**: calcd for C_22_H_20_O_3_ [*m*/*z*] [M + Na]^+^ 355.1305; found, [M + Na]^+^ 355.1301.

#### (3′-(Benzo-1,4-dioxan-6-yl)-2′-methyl-[1,1′-biphenyl]-3-yl)methanol (**4b**)

6-(3-Bromo-2-methylphenyl)-1,4-benzodioxane (**2a**) (2.98 g, 9.78 mmol, 1.0 equiv), 3-(hydroxymethyl)phenylboronic acid (2.03 g, 13.30 mmol, 1.2 equiv), K_2_CO_3_ (4.90 g, 35.56 mmol, 3.2 equiv), Pd(dppf)Cl_2_ complex with dichloromethane (0.36 g, 0.45 mmol, 0.04 equiv). Product **4b** was obtained as a colorless solid with 50.1% (1.48 g) yield.

**R**_**f**_ = 0.24 (SiO_2_, hexane/ethyl acetate 2:1); ^**1**^**H NMR** (600 MHz, CDCl_3_): δ [ppm] 7.43–7.40 (m, 1H), 7.36 (m, 2H), 7.29 (dt, *J* = 7.5, 1.2 Hz, 1H), 7.26–7.24 (m, 1H), 7.22–7.19 (m, 2H), 6.91 (d, *J* = 8,2 Hz, 1H), 6.88 (d, *J* = 2.0, 1H), 6.83 (dd, *J* = 8.2, 2.1 Hz, 1H), 4.75 (s, 2H), 4.30 (s, 4H), 2.12 (s, 3H), 1.76 (s_broad_, 1H); ^**13**^**C NMR** (151 MHz, CDCl_3_): δ [ppm] 143.1, 142.8, 142.6, 142.6, 142.3, 140.7, 135.8, 133.1, 129.1, 128.8, 128.7, 128.3, 128.0, 125.4, 125.3, 122.6, 118.2, 116.9, 65.4, 64.4, 18.8; **IR (ATR)** [cm^–1^]: 3360, 2875, 1578, 1506, 1069; **LC–MS (DAD/ESI)**: *t*_R_ = 7.48 min, calcd for C_22_H_20_O_3_ [*m*/*z*] [M + H]^+^ 333.15, [M-H_2_O + H]^+^ 315.14; found, [M-H_2_O + H]^+^ 315.21; **HRMS (ESI)**: calcd for C_22_H_20_O_3_ [*m*/*z*] [M + Na]^+^ 355.1305; found, [M + Na]^+^ 355.1304.

#### (3′-(Benzo-1,4-dioxan-6-yl)-2′-methyl-[1,1′-biphenyl]-2-yl)methanol (**4c**)

6-(3-Bromo-2-methylphenyl)-1,4-benzodioxane (**2a**) (2.98 g, 9.78 mmol, 1.0 equiv), 2-(hydroxymethyl)phenylboronic acid (2.03 g, 13.30 mmol, 1.2 equiv), K_2_CO_3_ (4.90 g, 35.56 mmol, 3.2 equiv), Pd(dppf)Cl_2_ complex with dichloromethane (0.36 g, 0.45 mmol, 0.04 equiv). Product **4c** was obtained as a colorless solid with 41.9% (1.24 g) yield.

**R**_**f**_ = 0.25 (SiO_2_, hexane/ethyl acetate 2:1); ^**1**^**H NMR** (600 MHz, CDCl_3_): δ [ppm] 7.59 (dd, *J* = 7.6, 0.7 Hz, 1H), 7.43 (dt, *J* = 7.5, 1.4 Hz, 1H), 7.38 (dt, *J* = 7.5, 1.4 Hz, 1H), 7.28–7.25 (m, 2H), 7.22 (dd, *J* = 7.5, 1.3 Hz, 1H), 7.14 (dd, *J* = 6.6, 2.4 Hz, 1H), 6.93 (d, *J* = 8.2 Hz, 1H), 6.90 (d, *J* = 2.0 Hz, 1H), 6.84 (dd, *J* = 8.2, 2.1 Hz, 1H), 4.49 (dd, *J* = 6.0, 1.2 Hz, 2H), 4.33 (s, 4H), 1.97 (s, 3H), 1.57 (t, *J* = 5.9 Hz, 1H); ^**13**^**C NMR** (151 MHz, CDCl_3_): δ [ppm] 143.1, 142.6, 142.2, 140.9, 140.8, 138.5, 135.6, 133.5, 129.5, 129.3, 128.3, 127.7, 127.6, 127.5, 125.3, 122.5, 118.2, 116.9, 64.4, 63.2, 18.2; **IR (ATR)** [cm^–1^]: 3559, 3533, 2933, 1581, 1505, 1317, 1066; **LC–MS (DAD/ESI)**: *t*_R_ = 7.58 min, calcd for C_22_H_20_O_3_ [*m*/*z*] [M-H_2_O + H]^+^ 315.14; found, [M-H_2_O + H]^+^ 315.21; **HRMS (ESI)**: calcd for C_22_H_20_O_3_: [M + Na]^+^ 355.1305; found, [M + Na]^+^ 355.1305.

#### (2′-Amino-3′-(benzo-1,4-dioxan-6-yl)-[1,1′-biphenyl]-4-yl)methanol (**4d**)

6-(2-Amino-3-bromophenyl)-1,4-benzodioxane (**2b**) (6.12 g, 20.00 mmol, 1.0 equiv), 4-(hydroxymethyl)phenylboronic acid (3.04 g, 20.00 mmol, 1.2 equiv), K_2_CO_3_ (9.22 g, 66.68 mmol, 4.2 equiv), Pd(dppf)Cl_2_ complex with dichloromethane (0.68 g, 0.83 mmol, 0.04 equiv). Product **4d** was obtained as a colorless solid with 32.0% (1.78 g) yield.

**R**_**f**_ = 0.19 (SiO_2_, hexane/ethyl acetate 1:1); ^**1**^**H NMR** (600 MHz, CDCl_3_): δ [ppm] 7.53 (m, 2H), 7.47 (d, *J* = 8.2 Hz, 2H), 7.12 (m, 2H), 7.05 (d, *J* = 2.00 Hz, 1H), 7.00 (dd, *J* = 8.3, 2.0 Hz, 1H), 6.97 (d, *J* = 8.2 Hz, 1H), 6.88 (t, *J* = 7.5 Hz, 1H), 4.75 (s, 2H), 4.31 (s, 4H), 3.90 (s_broad_, 2H), 2.23 (s_broad_, 1H); ^**13**^**C NMR** (151 MHz, CDCl_3_): δ [ppm] 143.7, 142.9, 141.0, 139.9, 139.2, 133.0, 129.8, 129.6, 129.5, 127.5, 127.5, 127.5, 122.4, 118.1, 117.6, 65.1, 64.5, 64.4; **IR (ATR)** [cm^–1^]: 3466, 3429, 3372, 3350, 3247, 1507, 1317, 1284; **LC–MS (DAD/ESI)**: *t*_R_ = 6.64 min, calcd for C_21_H_19_NO_3_ [*m*/*z*] [M + H]^+^ 334.14; found, [M + H]^+^ 334.22; **HRMS (ESI)**: calcd for C_21_H_19_NO_3_ [*m*/*z*] [M + Na]^+^ 356.1257; found, [M + Na]^+^ 356.1259.

#### (3′-(Benzo-1,4-dioxan-6-yl)-2′-chloro-[1,1′-biphenyl]-4-yl)methanol (**4e**)

6-(3-Bromo-2-chlorophenyl)-1,4-benzodioxane (**2c**) (0.19 g, 0.59 mmol, 1.0 equiv), 4-(hydroxymethyl)phenylboronic acid (0.11 g, 0.71 mmol, 1.2 equiv), K_2_CO_3_ (0.24 g, 1.77 mmol, 4.2 equiv), Pd(dppf)Cl_2_ complex with dichloromethane (0.048 g, 0.06 mmol, 0.1 equiv). Product **4e** was obtained as a colorless solid with 32.6% (0.068 g) yield.

**R**_**f**_ = 0.50 (SiO_2_, hexane/ethyl acetate 1:2); ^**1**^**H NMR** (600 MHz, CDCl_3_): δ [ppm] 7.48 (m, 4H), 7.36–7.30 (m, 3H), 7.03 (d, *J* = 1.9 Hz, 1H), 6.98–6.95 (m, 2H), 4.79 (s, 2H), 4.33 (s, 4H), 1.75 (s, 1H);^**13**^**C NMR** (151 MHz, CDCl_3_): δ [ppm] 143.2, 143.0, 141.3, 141.1, 140.1, 139.6, 133.4, 131.0, 130.5, 130.2, 129.8, 126.7, 126.4, 122.8, 118.6, 116.9, 65.2, 64.5, 64.4; **IR (ATR)** [cm^–1^]: 3317, 1507, 1455, 1247, 795; **LC–MS (DAD/ESI)**: *t*_R_ = 7.29 min, calcd for C_21_H_17_ClO_3_ [*m*/*z*] [M + H]^+^ 353.09, [M-H_2_O + H]^+^ 335.08; found, [M-H_2_O + H]^+^ 335.15; **HRMS (ESI)**: calcd for C_21_H_17_ClO_3_ [*m*/*z*] [M + Na]^+^ 375.0758; found, [M + Na]^+^ 375.0761.

#### (3′-(Benzo-1,4-dioxan-6-yl)-3-methoxy-2′-methyl-[1,1′-biphenyl]-4-yl)methanol (**4i**)

6-(3-Bromo-2-methylphenyl)-1,4-benzodioxane (**2a**) (1.50 g, 4.92 mmol, 1.0 equiv), (2-methoxy-4-(4,4,5,5-tetramethyl-1,3,2-dioxaborolan-2-yl)phenyl)methanol (**3a**) (1.30 g, 4.92 mmol, 1.0 equiv), K_2_CO_3_ (2.72 g, 19.66 mmol, 4.0 equiv), Pd(dppf)Cl_2_ complex with dichloromethane (0.20 g, 0.25 mmol, 0.05 equiv). Product **4i** was obtained as a colorless solid with 68.7% (1.22 g) yield.

**R**_**f**_ = 0.38 (SiO_2_, hexane/ethyl acetate 1:1); ^**1**^**H NMR** (600 MHz, CDCl_3_): δ [ppm] 7.34 (d, *J* = 7.5 Hz, 1H), 7.30–7.28 (m, 1H), 7.26–7.23 (m, 2H), 6.96 (dd, *J* = 7.5, 1.4 Hz, 1H), 6.94 (d, *J* = 8.2 Hz, 1H), 6.91 (m, 2H), 6.86 (dd, *J* = 8.2, 2.1 Hz, 1H), 4.77 (s, 2H), 4.33 (s, 4H), 3.91 (s, 3H), 2.20 (s, 3H); ^**13**^**C NMR** (151 MHz, CDCl_3_): δ [ppm] 157.1, 143.5, 143.1, 142.7, 142.6, 142.4, 135.7, 133.1, 129.2, 128.7, 128.5, 127.5, 125.3, 122.5, 121.6, 118.2, 116.9, 111.6, 64.5, 64.4, 62.0, 55.4, 18.8; **IR (ATR)** [cm^–1^]: 3547, 2936, 1505, 1302, 1229; **LC–MS (DAD/ESI)**: *t*_R_ = 7.58 min, calcd for C_23_H_22_O_4_ [*m*/*z*] [M + H]^+^ 363.15; found, [M + H]^+^ 363.26; **HRMS (ESI)**: calcd for C_23_H_22_O_4_ [*m*/*z*] [M + Na]^+^ 385.1410; found, [M + Na]^+^ 385.1411.

#### 3-(((3′-(Benzo-1,4-dioxan-6-yl)-4-(hydroxymethyl)-2′-methyl-[1,1′-biphenyl]-3-yl)oxy)methyl)benzonitrile (**4j**)

6-(3-Bromo-2-methylphenyl)-1,4-benzodioxane (**2a**) (0.60 g, 2.0 mmol, 1.0 equiv), 3-((2-(hydroxymethyl)-5-(4,4,5,5-tetramethyl-1,3,2-dioxaborolan-2-yl)phenoxy)methyl)benzonitrile (**3i**) (0.86 g, 2.4 mmol, 1.2 equiv), K_2_CO_3_ (1.09 g, 7.9 mmol, 4.0 equiv), Pd(PPh_3_)_4_ (0.11 g, 0.1 mmol, 0.05 equiv). Product **4j** was obtained as a bluish oil with 29.1% (0.266 g) yield.

**R**_**f**_ = 0.45 (SiO_2_, hexane/ethyl acetate 1:1); ^**1**^**H NMR** (600 MHz, DMSO-*d*_6_): δ [ppm] 7.93 (s, 1H), 7.81 (t, *J* = 7.9 Hz, 2H), 7.62 (t, *J* = 7.8 Hz, 1H), 7.45 (d, *J* = 7.6 Hz, 1H), 7.27 (t, *J* = 7.6 Hz, 1H), 7.15 (dd, *J* = 10.7, 3.5 Hz, 2H), 6.99 (d, *J* = 1.3 Hz, 1H), 6.95 (dd, *J* = 7.6, 1.3 Hz, 1H), 6.91 (d, *J* = 8.2 Hz, 1H), 6.84 (d, *J* = 2.1 Hz, 1H), 6.80 (dd, *J* = 8.2, 2.1 Hz, 1H), 5.24 (s, 2H), 5.07 (t, *J* = 5.6 Hz, 1H), 4.62 (d, *J* = 5.6 Hz, 1H), 4.28 (s, 4H), 1.99 (s, 3H); ^**13**^**C NMR** (151 MHz, DMSO-*d*_6_): δ [ppm] 154.3, 143.0, 142.5, 142.3, 141.8, 141.4, 139.2, 134.9, 132.4, 132.1, 131.5, 130.7, 129.7, 129.4, 128.7, 128.4, 127.2, 125.5, 122.1, 121.3, 118.8, 117.7, 116.8, 112.9, 111.4, 67.9, 64.1, 57.9, 18.6; **IR (ATR)** [cm^–1^]: 3390, 2924, 2873, 2230, 1574, 1505, 1459, 1402, 1301, 1278, 1221, 1066, 894, 791; **LC–MS (DAD/ESI)**: *t*_R_ = 8.16 min, calcd for C_30_H_25_NO_4_ (*m*/*z*) [M-H_2_O + H]^+^ 446.18; found, [M-H_2_O + H]^+^ 446.34; **HRMS (ESI)**: calcd for C_30_H_25_NO_4_ (*m*/*z*) [M + Na]^+^ 486.1676; found, [M + Na]^+^ 486.1676.

#### (3′-(Benzo-1,4-dioxan-6-yl)-2′-chloro-3-methoxy-[1,1′-biphenyl]-4-yl)methanol (**4k**)

6-(3-Bromo-2-chlorophenyl)-1,4-benzodioxane (**2c**) (0.46 g, 1.41 mmol, 1.0 equiv), (2-methoxy-4-(4,4,5,5-tetramethyl-1,3,2-dioxaborolan-2-yl)phenyl)methanol (**3a**) (0.37 g, 1.41 mmol, 1.00 equiv), K_2_CO_3_ (0.78 g, 5.63 mmol, 4.0 equiv), Pd(dppf)Cl_2_ complex with dichloromethane (0.06 g, 0.07 mmol, 0.05 equiv). Product **4k** was obtained as a colorless solid with 70.0% (0.377 g) yield.

**R**_**f**_ = 0.38 (SiO_2_, hexane/ethyl acetate 1:1); ^**1**^**H NMR** (600 MHz, CDCl_3_): δ [ppm] 7.35 (d, *J* = 7.4 Hz, 1H), 7.33–7.28 (m, 3H), 7.02 (dd, *J* = 7.5, 1.5 Hz, 1H), 7.00 (m, 2H), 6.99–6.92 (m, 2H), 4.74 (d, *J* = 6.4 Hz, 2H), 4.31 (s, 4H), 3.89 (s, 3H), 2.30 (t, *J* = 6.5 Hz, 1H); ^**13**^**C NMR** (151 MHz, CDCl_3_): δ [ppm] 156.9, 143.2, 143.0, 141.4, 141.1, 141.1, 133.3, 131.0, 130.6, 130.1, 128.4, 128.3, 126.4, 122.8, 121.8, 118.6, 116.9, 111.9, 64.5, 64.4, 62.0, 55.4; **IR (ATR)** [cm^–1^]: 3530, 2936, 1506, 1460, 1303, 1227; **LC–MS** (DAD/ESI): *t*_R_ = 7.43 min, calcd for C_22_H_19_ClO_4_ [*m*/*z*] [M + H]^+^ 383.10, [M-H_2_O + H]^+^ 365.09; found, [M-H_2_O + H]^+^ 365.19; **HRMS (ESI)**: calcd for C_22_H_19_ClO_4_ [*m*/*z*] [M + Na]^+^ 405.0864; found, [M + Na]^+^ 405.0863.

#### (3′-(Benzo-1,4-dioxan-6-yl)-2′-chloro-3-ethoxy-[1,1′-biphenyl]-4-yl)methanol (**4l**)

6-(3-Bromo-2-chlorophenyl)-1,4-benzodioxane (**2c**) (0.50 g, 1.53 mmol, 1.2 equiv), (2-ethoxy-4-(4,4,5,5-tetramethyl-1,3,2-dioxaborolan-2-yl)phenyl)methanol (**3b**) (0.35 g, 1.27 mmol, 1.0 equiv), K_2_CO_3_ (0.70 g, 5.10 mmol, 4.0 equiv), Pd(dppf)Cl_2_ complex with dichloromethane (0.05 g, 0.06 mmol, 0.05 equiv). Product **4l** was obtained as a colorless solid with 37.9% (0.191 g) yield.

**R**_**f**_ = 0.40 (SiO_2_, hexane/ethyl acetate 1:1); ^**1**^**H NMR** (600 MHz, CDCl_3_): δ [ppm] 7.38 (m, 1H), 7.35–7.31 (m, 3H), 7.05–7.03 (m, 2H), 7.02 (m, 1H), 6.99–6.95 (m, 2H), 4.79 (s, 2H), 4.33 (s, 4H), 4.16 (q, *J* = 7.0 Hz, 2H), 2.58 (s_broad_, 1H), 1.48 (t, *J* = 7.0 Hz, 3H); ^**13**^**C NMR** (151 MHz, CDCl_3_): δ [ppm] 156.3, 143.2, 143.0, 141.4, 141.1, 140.9, 133.3, 131.0, 130.5, 130.1, 128.4, 128.4, 126.4, 122.8, 121.7, 118.6, 116.9, 112.8, 64.5, 64.4, 63.7, 62.1, 14.9; **IR (ATR)** [cm^–1^]: 3219, 2972, 1509, 1399, 1279, 1070, 1052, 1044; **LC–MS (DAD/ESI)**: *t*_R_ = 7.92 min, calcd for C_23_H_21_ClO_4_ [*m*/*z*] [M + H]^+^ 397.12 [M-H_2_O + H]^+^ 379.11; found, [M-H_2_O + H]^+^ 379.21; **HRMS (ESI)**: calcd for C_23_H_21_ClO_4_ [*m*/*z*] [M + Na]^+^ 419.1021; found, [M + Na]^+^ 419.1021.

#### (3′-(Benzo-1,4-dioxan-6-yl)-2′-chloro-3-isopropoxy-[1,1′-biphenyl]-4-yl)methanol (**4m**)

6-(3-Bromo-2-chlorophenyl)-1,4-benzodioxane (**2c**) (0.33 g, 1.03 mmol, 1.0 equiv), (2-izopropoxy-4-(4,4,5,5-tetramethyl-1,3,2-dioxaborolan-2-yl)phenyl)methanol (**3c**) (0.36 g, 1.23 mmol, 1.2 equiv), K_2_CO_3_ (0.42 g, 3.08 mmol, 3.0 equiv), Pd(dppf)Cl_2_ complex with dichloromethane (0.08 g, 0.10 mmol, 0.1 equiv). Product **4m** was obtained as a colorless oil with 60.8% (0.256 g) yield.

**R**_**f**_ = 0.4 (SiO_2_, hexane/ethyl acetate 1:1); ^**1**^**H NMR** (600 MHz, CDCl_3_): δ [ppm] 7.34–7.32 (m, 4H), 7.01–7.00 (m, 2H), 6.98 (dd, *J* = 7.6, 1.5 Hz, 1H), 6.96–6.92 (m, 2H), 4.72 (s, 2H), 4.66 (hept, *J* = 6.0 Hz, 1H), 4.31 (s, 4H), 1.39 (d, *J* = 6.0 Hz, 6H); ^**13**^**C NMR** (151 MHz, CDCl_3_): δ [ppm] 155.3, 143.3, 143.1, 141.5, 141.2, 140.9, 133.4, 131.1, 130.6, 130.2, 129.3, 128.7, 126.5, 122.9, 121.6, 118.7, 117.0, 114.3, 70.4, 64.6, 64.5, 62.6, 22.4; **IR (ATR)** [cm^–1^]: 3419, 3053, 2976, 2931, 2874, 1583, 1507, 1456, 1302, 1280, 1248, 1225, 1068, 797; **LC–MS (DAD/ESI)**: *t*_R_ = 8.23 min, calcd for C_24_H_23_ClNO_4_ (*m*/*z*) [M-H_2_O + H]^+^ 393.13; found, [M-H_2_O + H]^+^ 393.23; **HRMS (ESI)**: calcd for C_24_H_23_ClO_4_ (*m*/*z*) [M + Na]^+^ 433.1177; found, [M + Na]^+^ 433.1177.

#### (3′-(Benzo-1,4-dioxan-6-yl)-2′-chloro-3-isopentyloxy-[1,1′-biphenyl]-4-yl)methanol (**4n**)

6-(3-Bromo-2-chlorophenyl)-1,4-benzodioxane (**2c**) (0.48 g, 1.47 mmol, 1.0 equiv), (2-izopentyloxy-4-(4,4,5,5-tetramethyl-1,3,2-dioxaborolan-2-yl)phenyl)methanol (**3d**) (0.66 g, 2.06 mmol, 1.4 equiv), K_2_CO_3_ (0.61 g, 4.41 mmol, 3.0 equiv), Pd(dppf)Cl_2_ complex with dichloromethane (0.12 g, 0.15 mmol, 0.1 equiv). Product **4n** was obtained as a colorless oil with 55.8% (0.361) yield.

**R**_**f**_ = 0.16 (SiO_2_, hexane/ethyl acetate 4:1); ^**1**^**H NMR** (600 MHz, CDCl_3_): δ [ppm] 7.35 (m, 4H), 7.02–6.98 (m, 3H), 6.96–6.92 (m, 2H), 4.75 (s, 2H), 4.31 (s, 4H), 4.08 (t, *J* = 6.6 Hz, 2 H), 2.39 (s_broad_, 1H), 1.89–1.79 (m, 1H), 1.73 (q, *J* = 6.6 Hz, 2H), 0.97 (d, *J* = 6.6 Hz, 6H); ^**13**^**C NMR** (151 MHz, CDCl_3_): δ [ppm] 156.6,143.3, 143.1, 141.6, 141.2, 141.1, 1335., 131.1, 130.7, 130.2, 128.5, 128.4, 126.5, 122.9, 121.8, 118.7, 117.0, 112.8, 66.7, 64.6, 64.6, 62.4, 38.2, 25.4, 227; **IR (ATR)** [cm^–1^]: 3376, 3055, 2955, 2930, 2871, 1583, 1507, 1456, 1303, 1280, 1248, 1225, 1068, 797; **LC–MS (DAD/ESI)**: *t*_R_ = 9.16 min, calcd for C_26_H_27_ClO_4_ (*m*/*z*) [M-H_2_O + H]^+^ 421.16; found, [M-H_2_O + H]^+^ 421.16; **HRMS (ESI)**: calcd for C_26_H_27_ClO_4_ (*m*/*z*) [M + Na]^+^ 461.1490; found, [M + Na]^+^ 461.1490.

#### 2-((3′-(Benzo-1,4-dioxan-6-yl)-2′-chloro-4-(hydroxymethyl)-[1,1′-biphenyl]-3-yl)oxy)acetonitrile (**4o**)

6-(3-Bromo-2-chlorophenyl)-1,4-benzodioxane (**2c**) (0.40 g, 1.24 mmol, 1.0 equiv), 2-(2-hydroxymethyl)-5-(4,4,5,5-tetramethyl-1,3,2-dioxaborolan-2-yl)phenoxy)acetonitrile (**3e**) (0.50 g, 1.72 mmol, 1.4 equiv), K_2_CO_3_ (0.51 g, 3.72 mmol, 3.0 equiv), Pd(dppf)Cl_2_ complex with dichloromethane (0.10 g, 0.12 mmol, 0.1 equiv). Product **4o** was obtained as a colorless solid with 56.0% (0.283 g) yield.

**R**_**f**_ = 0.24 (SiO_2_, hexane/ethyl acetate 1:1); ^**1**^**H NMR** (600 MHz, CDCl_3_): δ [ppm] 7.49 (d, *J* = 7.7 Hz, 1H), 7.36–7.32 (m, 2H), 7.29 (dd, *J* = 6.5, 2.8 Hz, 1H), 7.19 (dd, *J* = 7.7, 1.4 Hz, 1H), 7.06 (d, *J* = 1.3 Hz, 1H), 7.00–6.99 (m, 1H), 6.96–6.91 (m, 2H), 4.86 (s, 2H), 4.78 (s, 2H), 4.31 (s, 4H), 1.90 (s_broad_, 1H); ^**13**^**C NMR** (151 MHz, CDCl_3_): δ [ppm] 153.8, 143.4, 143.2, 141.4, 141.3, 133.2, 131.0, 130.2, 129.5, 129.2, 126.7, 124.5, 122.9, 118.7, 117.0, 115.0, 113.4, 64.6, 64.5, 60.8, 54.0; **IR (ATR)** [cm^–1^]: 3524, 3053, 2985, 2944, 2886, 1586, 1510, 1400, 1321, 1283, 1244, 1069, 1048, 890, 787; **LC–MS (DAD/ESI)**: *t*_R_ = 6.99 min, calcd for C_23_H_18_ClNO_4_ (*m*/*z*) [M-H_2_O + H]^+^ 390.09; found, [M-H_2_O + H]^+^ 390.18; **HRMS (ESI)**: calcd for C_23_H_28_ClNO_4_ (*m*/*z*) [M + Na]^+^ 430.0816; found, [M + Na]^+^ 430.0817.

#### (3′-(Benzo-1,4-dioxan-6-yl)-2′-chloro-3-cyclobutylmethoxy-[1,1′-biphenyl]-4-yl)methanol (**4p**)

6-(3-Bromo-2-chlorophenyl)-1,4-benzodioxane (**2c**) (0.49 g, 1.50 mmol, 1.0 equiv), (2-(cyclobutylmethoxy)-4-(4,4,5,5-tetramethyl-1,3,2-dioxaborolan-2-yl)phenyl)methanol (**3f**) (0.67 g, 2.10 mmol, 1.4 equiv), K_2_CO_3_ (0.62 g, 4.48 mmol, 3.0 equiv), Pd(dppf)Cl_2_ complex with dichloromethane (0.12 g, 0.15 mmol, 0.1 equiv). Product **4p** was obtained as a colorless solid with 64.8% (0.425 g) yield.

**R**_**f**_ = 0.16 (SiO_2_, hexane/ethyl acetate 4:1); **IR (ATR)** [cm^–1^]: 3414, 2976, 2935, 2869, 1583, 1507, 1456, 1303, 1280, 1249, 1226, 1069, 1046, 797, 746; ^**1**^**H NMR** (600 MHz, CDCl_3_): δ [ppm] 7.34–7.27 (m, 4H), 7.02–6.98 (m, 3H), 6.96–6.92 (m, 2H), 4.75 (s, 2H), 4.31 (s, 4H), 4.02 (d, *J* = 6.6. Hz, 2H), 2.8702.78 (m, 1H), 2.19–2.12 (m, 2H), 2.02–1.85 (m, 4H); ^**13**^**C NMR** (151 MHz, CDCl_3_): δ [ppm] 156.7, 143.3, 143.1, 141.5, 141.2, 141.1, 133.5, 131.1, 130.7, 130.2, 128.6, 128.4, 126.5, 1229., 121.8, 118.7, 117.0, 113.0, 72.2, 64.6, 64.6, 62.5, 34.7, 25.0, 18.8; **LC–MS (DAD/ESI)**: *t*_R_ = 9.00 min, calcd for C_26_H_25_ClNO_4_ (*m*/*z*) [M-H_2_O + H]^+^ 419.14; found, [M-H_2_O + H]^+^ 419.24; **HRMS (ESI)**: calcd for C_26_H_25_ClO_4_ (*m*/*z*) [M + Na]^+^ 459.1334; found, [M + Na]^+^ 459.1333.

#### (3′-(Benzo-1,4-dioxan-6-yl)-2′-chloro-3-cyclopentylmethoxy-[1,1′-biphenyl]-4-yl)methanol (**4q**)

6-(3-Bromo-2-chlorophenyl)-1,4-benzodioxane (**2c**) (0.29 g, 0.89 mmol, 1.0 equiv), (2-(cyclobutylmethoxy)-4-(4,4,5,5-tetramethyl-1,3,2-dioxaborolan-2-yl)phenyl)methanol (**3g**) (0.42 g, 1.26 mmol, 1.4 equiv), K_2_CO_3_ (0.37 g, 2.68 mmol, 3.0 equiv), Pd(dppf)Cl_2_ complex with dichloromethane (0.07 g, 0.09 mmol, 0.1 equiv). Product **4q** was obtained as a colorless solid with 62.4% (0.251 g) yield.

**R**_**f**_ = 0.16 (SiO_2_, hexane/ethyl acetate 4:1); ^**1**^**H NMR** (600 MHz, CDCl_3_): δ [ppm] 7.35 (m, 4H), 7.02–6.97 (m, 3H), 6.96–6.91 (m, 2H), 4.75 (s, 2H), 4.31 (s, 4H), 3.93 (d, *J* = 6.9 Hz, 1H), 2.45–2.38 (m, 1H), 1.90–1.83 (m, 2H), 1.69–1.57 (m, 4H), 1.41–1.33 (m, 2H); ^**13**^**C NMR** (151 MHz, CDCl_3_): δ [ppm] 156.7, 143.3, 143.1, 141.6, 141.2, 141.1, 133.5, 131.1, 130.6, 128.5, 128.4, 126.5, 122.9, 121.7, 118.7, 117.0, 112.8, 72.4, 64.6, 64.6, 62.5, 39.2, 29.7, 25.6; **IR (ATR)** [cm^–1^]: 3390, 3054, 2952, 2868, 1583, 1507, 1456, 1303, 1280, 1249, 1225, 1069, 1040, 797, 747; **LC–MS (DAD/ESI)**: *t*_R_ = 9.38 min, calcd for C_27_H_27_ClO_4_ (*m*/*z*) [M-H_2_O + H]^+^ 433.16; found, [M-H_2_O + H]^+^ 433.25; **HRMS (ESI)**: calcd for C_27_H_27_ClO_4_ (*m*/*z*) [M + Na]^+^ 473.1490; found, [M + Na]^+^ 473.1488.

#### 3′-(((3′-(Benzo-1,4-dioxan-6-yl)-2′-chloro-4-(hydroxymethyl)-[1,1′-biphenyl]-3-yl)oxy)methyl)-[1,1′-biphenyl]-3-carbonitrile (**4r**)

6-(3-Bromo-2-chlorophenyl)-1,4-benzodioxane (**2c**) (0.28 g, 0.86 mmol, 1.0 equiv), 3′-((2-(hydroxymethyl)-5-(4,4,5,5-tetramethyl-1,3,2-dioxaborolan-2-yl)phenoxy)methyl)-[1,1′-biphenyl]-3-carbonitrile (**3h**) (0.38 g, 0.86 mmol, 1.0 equiv), K_2_CO_3_ (0.48 g, 3.45 mmol, 4.0 equiv), Pd(dppf)Cl_2_ complex with dichloromethane (0.03 g, 0.04 mmol, 0.05 equiv). Product **4r** was obtained as a colorless oil with 88.8% (0.427 g) yield.

**R**_**f**_ = 0.37 (SiO_2_, hexane/ethyl acetate 1:1); ^**1**^**H NMR** (600 MHz, CDCl_3_): δ [ppm] 7.86 (t, *J* = 1.6 Hz, 1H), 7.80 (m, 1H), 7.64 (dt, *J* = 7.7, 1.3 Hz, 1H), 7.62 (s, 1H), 7.56–7.48 (m, 4H), 7.41 (d, *J* = 7.6 Hz, 1H), 7.33–7.26 (m, 3H), 7.10 (d, *J* = 1.4 Hz, 1H), 7.06 (dd, *J* = 7.6, 1.5 Hz, 1H), 6.98 (t, *J* = 1.2 Hz, 1H), 6.92 (m, 2H), 5.22 (s, 2H), 4.81 (s, 2H), 4.30 (s, 4H), 2.27 (s_broad_, 1H); ^**13**^**C NMR** (151 MHz, CDCl_3_): δ [ppm] 155.8, 143.2, 143.0, 142.1, 141.2, 141.1, 141.0, 139.4, 137.7, 133.2, 131.6, 130.9, 130.8, 130.6, 130.1, 129.7, 129.6, 128.8, 128.6, 127.4, 126.9, 126.4, 126.2, 122.8, 122.4, 118.8, 118.6, 116.9, 113.4, 113.1, 70.0, 64.5, 64.4, 61.8; **IR (ATR)** [cm^–1^]: 3334, 3059, 2931, 2229, 1585, 1507, 1397, 1246, 781; **LC–MS (DAD/ESI)**: *t*_R_ = 8.83 min, calcd for C_35_H_26_ClNO_4_ [*m*/*z*] [M + H]^+^ 560.16 [M-H_2_O + H]^+^ 542.16; found, [M-H_2_O + H]^+^ 542.32; **HRMS (ESI)**: calcd for C_35_H_26_ClNO_4_ (*m*/*z*) [M + Na]^+^ 582.1443; found, [M + Na]^+^ 582.1440.

### Synthesis of C2′ Halogenated and C2′-Cyano Derivatives by the Sandmeyer Reaction (**4f–h**)

#### (3′-(Benzo-1,4-dioxan-6-yl)-2′-bromo-[1,1′-biphenyl]-4-yl)methanol (**4f**)

In a three-necked round-bottom flask equipped with a thermometer, (2′-amino-3′-(benzo-1,4-dioxan-6-yl)-[1,1′-biphenyl]-4-yl)methanol (**4d**) (0.22 g, 0.63 mmol, 1 equiv) was added and dissolved in 1.2 mL of 48% HBr. The mixture was cooled in an ice bath and a solution of NaNO_2_ (0.09 g, 1.35 mmol, 2.0 equiv) in 2 mL of water was added dropwise, maintaining the temperature below 5 °C, and the mixture was stirred for 5 min and a solution of CuBr_2_ (0.37 g, 2.59 mmol, 4 equiv) in 1.8 mL of 48% HBr was added dropwise, maintaining the temperature below 5 °C. The mixture was heated in 70 °C for 2 h. After this time, the solution was transferred into a separatory funnel, 5 mL of water was added, and the aqueous phase was extracted with ethyl acetate (3 × 15 mL). The organic phases were collected, dried over anhydrous MgSO_4_, and the solvent was evaporated. The product was purified by flash chromatography (SiO_2_, hexane/ethyl acetate, 1:2), giving the final product **4f** with 9.1% (0.02 g) yield.

**R**_**f**_ = 0.60 (SiO_2_, hexane/ethyl acetate 1:2); ^**1**^**H NMR** (600 MHz, CDCl_3_): δ [ppm] 7.37–2.28 (m, 5H), 7.21–7.16 (m, 2H), 6.88 (m, 1H), 6.86–6.80 (m, 2H), 4.69 (s, 2H), 4.23 (s, 4H), 1.55 (s_broad_, 1H); ^**13**^**C NMR** (151 MHz, CDCl_3_): δ [ppm] 143.5, 143.2, 143.1, 142.9, 141.6, 140.1, 135.4, 130.3, 123.0, 129.8, 126.9, 126.6, 123.3, 122.8, 118.5, 116.8, 65.2, 64.5, 64.4; **IR (ATR)** [cm^–1^]: 3291, 2927, 1505, 1454, 1251, 1070; **LC–MS (DAD/ESI**^+^): *t*_R_ = 7.35 min, calcd for C_21_H_17_BrO_3_ [M-H_2_O + H]^+^ 379.03; found, [M-H_2_O + H]^+^ 379.14; **HRMS (ESI**^+^): calcd for C_21_H_17_BrO_3_ [*m*/*z*] [M + Na]^+^ 419.0253, [M + 2 + Na]^+^ 421.0233; found, [M + Na]^+^ 419.0251, [M + 2 + Na]^+^ 421.0233.

#### (3′-(Benzo-1,4-dioxan-6-yl)-2′-iodo-[1,1′-biphenyl]-4-yl)methanol (**4g**)

In a three-necked round-bottom flask equipped with a thermometer, (2′-amino-3′-(benzo-1,4-dioxan-6-yl)-[1,1′-biphenyl]-4-yl)methanol (**4d**) (0.21 g, 0.62 mmol, 1 equiv) was added and dissolved in 2 mL of dioxane/water (3/1, v/v) mixture. After dissolution, 2 mL of 10% HCl was added and the mixture was cooled in an ice bath; then, a solution of NaNO_2_ (0.05 g, 0.62 mmol, 1.0 equiv) in 2 mL of water was added dropwise, maintaining the temperature below 5 °C. The mixture was stirred for 1 h, and a solution of KI (0.42 g, 2.47 mmol, 4 equiv) in 4 mL of water was added dropwise, maintaining the temperature below 5 °C; then, the solution was stirred overnight at room temperature. After this time, the solution was transferred into a separatory funnel, 5 mL of water was added, and the aqueous phase was extracted with ethyl acetate (3 × 15 mL). The organic phases were collected, dried over anhydrous MgSO_4_, and the solvent was evaporated. The product was purified by flash chromatography (SiO_2_, hexane/ethyl acetate, 1:2), giving the final product **4g** with 27.6% (0.08 g) yield.

**R**_**f**_ = 0.47 (SiO_2_, hexane/ethyl acetate 1:2); ^**1**^**H NMR** (600 MHz, CDCl_3_): δ [ppm] 7.44 (d, *J* = 8.2 Hz, 2H), 7.37–7.35 (m, 3H), 7.23 (dd, *J* = 7.6, 1.7 Hz, 1H), 7.21 (dd, *J* = 7.5, 1.7 Hz, 1H), 6.91 (d, *J* = 8.3 Hz, 1H), 6.89 (d, *J* = 2.1 Hz, 1H), 6.84 (dd, *J* = 8.3, 2.1 Hz, 1H), 4.77 (d, *J* = 2.6 Hz, 2H), 4.31 (s, 4H), 1.72 (s_broad_, 1H); ^**13**^**C NMR** (151 MHz, CDCl_3_): δ [ppm] 147.7, 147.5, 145.1, 143.1, 142.8, 140.2, 139.0, 129.7, 128.8, 128.5, 127.6, 126.5, 122.7, 118.4, 116.7, 104.0, 65.2, 64.5, 64.4; **IR (ATR)** [cm^–1^]: 3309, 2955, 1505, 1451, 1252, 1071; **LC–MS (DAD/ESI**^+^): *t*_R_ = 7.47 min, calcd for C_21_H_17_IO_3_ [M-H_2_O + H]^+^ 427.02; found, [M-H_2_O + H]^+^ 427.13; **HRMS (ESI**^+^): calcd for C_21_H_17_IO_3_ [*m*/*z*] [M + Na]^+^ 467.0115; found, [M + Na]^+^ 467.0115.

#### 3-(3′-(Benzo-1,4-dioxan-6-yl)-4′-(hydroxymethyl)-[1,1′-biphenyl]-2-carbonitrile (**4h**)

In a three-necked round-bottom flask equipped with a thermometer, (2′-amino-3′-(benzo-1,4-dioxan-6-yl)-[1,1′-biphenyl]-4-yl)methanol (**4d**) (0.21 g, 0.62 mmol, 1 equiv) was added and dissolved in 6 mL of dioxane/water (2/1, v/v) mixture. After dissolution, 0.2 mL of conc. HCl was added and the mixture was cooled in an ice bath; then, a solution of NaNO_2_ (0.07 g, 0.96 mmol, 1.5 equiv) in 2 mL of water was added dropwise, maintaining the temperature below 5 °C. The mixture was stirred for 5 min and then added to a previously prepared solution of CuCN (0.08 g, 0.86 mmol, 1.4 equiv) and KCN (0.16 g, 2.37 mmol, 3.8 equiv) in 4 mL of water. The solution was stirred for 4 h. After this time, 3 mL of 2 M NaOH was added and the mixture was transferred into a separatory funnel. The aqueous phase was extracted with ethyl acetate (3 × 15 mL). The organic phases were collected, dried over anhydrous MgSO_4_, and the solvent was evaporated. The product was purified by flash chromatography (SiO_2_, hexane/ethyl acetate, 1:2), giving the final product **4h** with 20.6% (0.04 g) yield.

**R**_**f**_ = 0.44 (SiO_2_, hexane/ethyl acetate 1:2); ^**1**^**H NMR** (600 MHz, CDCl_3_): δ [ppm] 7.63 (t, *J* = 7.8 Hz, 1H), 7.58 (m, 2H), 7.50 (d, *J* = 8.2 Hz, 2H), 7.43–7.41 (m, 2H), 7.10 (d, *J* = 2.1 Hz, 1H), 7.08 (dd, *J* = 8.3, 2.2 Hz, 1H), 6.97 (d, *J* = 8.3 Hz, 1H), 4.77 (s, 2H), 4,31 (s, 4H), 1.82 (s_broad_, 1H); ^**13**^**C NMR** (151 MHz, CDCl_3_): δ [ppm] 146.6, 146.4, 144.2, 143.5, 141.4, 138.1, 132.3, 131.9, 129.3, 128.8, 128.5, 127.1, 122.3, 118.1, 118.0, 117.5, 110.3, 65.0, 64.5, 64.4; **IR (ATR)** [cm^–1^]: 3418, 2931, 2224, 1513, 1461, 1288; **LC–MS (DAD/ESI**^+^): *t*_R_ = 6.60 min, calcd for C_22_H_17_NO_3_ [*m*/*z*] [M]^+^ 343.12; found, [M]^+^ 343.25; **HRMS (ESI**^+^): calcd for C_22_H_17_NO_3_ [*m*/*z*] [M + Na]^+^ 366.1101; found, [M + Na]^+^ 366.1100.

### General Procedure for Williamson Ether Synthesis (**5a**, **5c**, **5e**, **5i**, **5k**, **5m**, **5o**, and **5q**)

Appropriate alcohol **4a–c** (1 equiv) was added in a round-bottom flask with anhydrous dichloromethane under an argon atmosphere and cooled in an ice bath; thionyl chloride (3 equiv) was added dropwise, and then, anhydrous DMF (0.2 mL per 2 mmol alcohol) was added. The reaction mixture was stirred approximately 2 h at room temperature and controlled by TLC (SiO_2_, hexane/ethyl acetate, 4/1). When all alcohol disappeared, the reaction mixture was poured onto saturated NaHCO_3_ and the aqueous phase extracted three times with dichloromethane. The organic phases were collected, dried over anhydrous MgSO_4_, and evaporated. The crude product was dissolved in anhydrous DMF; then, K_2_CO_3_ or Cs_2_CO_3_ (3 equiv), appropriate nucleophile (1.0–1.5 equiv), and KI (0.1 equiv) were added. The reaction mixture was heated at 80 °C overnight. After this time, the mixture was transferred into a separatory funnel with water and extracted three times with ethyl acetate. The organic phases were collected, dried over anhydrous MgSO_4_, and evaporated. The products were separated by column chromatography (SiO_2_, hexane/ethyl acetate, or CH_2_Cl_2_).

#### Methyl 3-((3′-(Benzo-1,4-dioxan-6-yl)-2′-methyl-[1,1′-biphenyl]-4-yl)methoxy)benzoate (**5a**)

(3′-(Benzo-1,4-dioxan-6-yl)-2′-methyl-[1,1′-biphenyl]-4-yl)methanol (**4a**) (0.30 g, 0.90 mmol, 1.0 equiv), SOCl_2_ (0.20 mL, 2.70 mmol, 3.0 equiv), K_2_CO_3_ (0.37 g, 2.70 mmol, 3.0 equiv), methyl 3-hydroxybenzoate (0.20 g, 1.35 mmol, 1.5 equiv), KI (0.015 g, 0.09 mmol, 0.1 equiv). Product **5a** was obtained as a colorless solid with 77.7% (0.326 g) yield.

**R**_**f**_ = 0.50 (SiO_2_, hexane/ethyl acetate 2:1); ^**1**^**H NMR** (600 MHz, CDCl_3_): δ [ppm] 7.62 (m, 1H), 7.58 (dt, *J* = 7.7, 1.0 Hz, 1H), 7.41 (d, *J* = 8.1 Hz, 2H), 7.30 (d, *J* = 8.1 Hz, 2H), 7.28 (t, *J* = 7.9 Hz, 1H), 7.18–7.11 (m, 4H), 6.82 (d, *J* = 8.2 Hz, 1H), 6.80 (d, *J* = 2.0 Hz, 1H), 6.74 (dd, *J* = 8.2, 2.1 Hz, 1H), 5.06 (s, 2H), 4.20 (s, 4H), 3.83 (s, 3H), 2.06 (s, 3H); ^**13**^**C NMR** (151 MHz, CDCl_3_): δ [ppm] 167.0, 158.8, 143.1, 142.6, 142.4, 142.4, 135.8, 135.0, 133.1, 131.6, 129.7, 129.5, 129.2, 128.9, 127.5, 125.4, 122.6, 122.4, 120.3, 118.2, 116.9, 115.1, 70.1, 64.5, 64.5, 52.2, 18.8; **IR (ATR)** [cm^–1^]: 2952, 1712, 1301, 1278, 1234; **LC–MS** (DAD/ESI): *t*_R_ = 9.80 min, calcd for C_30_H_26_O_5_ [*m*/*z*] [M + H]^+^ 467.19; found, [M + H]^+^ 467.41; **HRMS (ESI)**: calcd for C_30_H_26_O_5_ [*m*/*z*] [M + Na]^+^ 489.1672; found, [M + Na]^+^ 489.1672.

#### 2-Bromo-5-((3′-(benzo-1,4-dioxan-6-yl)-2′-methyl-[1,1′-biphenyl]-4-yl)methoxy)benzaldehydem (**5c**)

(3′-(Benzo-1,4-dioxan-6-yl)-2′-methyl-[1,1′-biphenyl]-4-yl)methanol (**4a**) (1.20 g, 3.62 mmol, 1.0 equiv), SOCl_2_ (0.79 mL, 10.86 mmol, 3.0 equiv), K_2_CO_3_ (1.50 g, 10.86 mmol, 3.0 equiv), 2-bromo-5-hydroxybenzaldehyde (1.09 g, 5.43 mmol, 1.5 equiv), KI (0.06 g, 0.36 mmol, 0.1 equiv). Product **5c** was obtained as a colorless solid with 84.9% (1.58 g) yield.

**R**_**f**_ = 0.55 (SiO_2_, hexane/ethyl acetate 2:1); ^**1**^**H NMR** (600 MHz, CDCl_3_): δ [ppm] 10.33 (s, 1H), 7.55 (m, 2H), 7.48 (d, *J* = 8.1 Hz, 2H), 7.40 (d, *J* = 8.1 Hz, 2H), 7.27–7.25 (m, 1H), 7.23–7.17 (m, 2H), 7.15 (dd, *J* = 8.8, 3.2 Hz, 1H), 6.91 (d, *J* = 8.2 Hz, 1H), 6.88 (d, *J* = 2.0 Hz, 1H), 6.84 (dd, *J* = 8.2, 2.1 Hz, 1H), 5.14 (s, 2H), 4.31 (s, 4H), 2.13 (s, 3H); ^**13**^**C NMR** (151 MHz, CDCl_3_): δ [ppm] 191.8, 158.4, 143.0, 142.6, 142.6, 142.4, 142.2, 137.5, 134.7, 134.3, 134.0, 133.1, 129.7, 129.2, 128.8, 127.4, 125.3, 123.8, 122.5, 118.3, 118.2, 116.9, 113.8, 70.4, 64.4, 64.4, 18.8; **IR (ATR)** [cm^–1^]: 3052, 2953, 2874, 2754, 1688, 1508, 1305, 1167, 993; **LC–MS (DAD/ESI)**: *t*_R_ = 10.14 min, calcd for C_29_H_23_BrO_4_ [*m*/*z*] [M + H]^+^ 515.08; found, [M + H]^+^ 515.18; **HRMS (ESI)**: calcd for C_29_H_23_BrO_4_ [*m*/*z*] [M + Na]^+^ 537.0672; found, [M + Na]^+^ 537.0679.

#### Methyl 3-((3′-(Benzo-1,4-dioxan-6-yl)-2′-methyl-[1,1′-biphenyl]-4-yl)methoxy)isoxazole-5-carboxylate (**5e**)

(3′-(Benzo-1,4-dioxan-6-yl)-2′-methyl-[1,1′-biphenyl]-4-yl)methanol (**4a**) (0.30 g, 0.90 mmol, 1.0 equiv), SOCl_2_ (0.20 mL, 2.70 mmol, 3.0 equiv), K_2_CO_3_ (0.37 g, 2.70 mmol, 3.0 equiv), methyl 3-hydroxyisoxazole-5-carboxylate (0.13 g, 0.90 mmol, 1.0 equiv), KI (0.015 g, 0.09 mmol, 0.1 equiv). Product **5e** was obtained as a colorless solid with 69.2% (0.285 g) yield.

**R**_**f**_ = 0.34 (SiO_2_, hexane/ethyl acetate 2:1); ^**1**^**H NMR** (600 MHz, CDCl_3_): δ [ppm] 7.54 (d, *J* = 8.1 Hz, 2H), 7.43 (d, *J* = 8.1 Hz, 2H), 7.31–7.22 (m, 3H), 6.94 (d, *J* = 8.2 Hz, 1H), 6.92 (d, *J* = 2.0 Hz, 1H), 6.86 (dd, *J* = 8.2, 2.1 Hz, 1H), 6.63 (s, 1H), 5.41 (s, 2H), 4.33 (s, 4H), 3.99 (s, 3H), 2.17 (s, 3H); ^**13**^**C NMR** (151 MHz, CDCl_3_): δ [ppm] 171.4, 160.4, 157.1, 143.1, 142.6, 142.4, 142.2, 135.7, 133.7, 133.1, 129.7, 129.3, 128.8, 128.2, 125.4, 122.6, 118.2, 116.9, 101.0, 72.1, 64.5, 64.5, 52.9, 18.8; **IR (ATR)** [cm^–1^]: 3144, 1747, 1607, 1504, 1313; **LC–MS (DAD/ESI)**: *t*_R_ = 8.98 min, decomposition during ionization; **HRMS (ESI)**: calcd for C_27_H_23_NO_6_ [*m*/*z*] [M + Na]^+^ 480.1418; found, [M + Na]^+^ 480.1418.

#### Methyl 3-((3′-(Benzo-1,4-dioxan-6-yl)-2′-methyl-[1,1′-biphenyl]-3-yl)methoxy)benzoate (**5i**)

(3′-(Benzo-1,4-dioxan-6-yl)-2′-methyl-[1,1′-biphenyl]-3-yl)methanol (**4b**) (0.30 g, 0.90 mmol, 1.0 equiv), SOCl_2_ (0.20 mL, 2.70 mmol, 3.0 equiv), K_2_CO_3_ (0.37 g, 2.70 mmol, 3.0 equiv), methyl 3-hydroxybenzoate (0.20 g, 1.35 mmol, 1.5 equiv), KI (0.015 g, 0.09 mmol, 0.1 equiv). Product **5i** was obtained as colorless solid with 58.8% (0.247 g) yield.

**R**_**f**_ = 0.47 (SiO_2_, CH_2_Cl_2_); ^**1**^**H NMR** (600 MHz, CDCl_3_): δ [ppm] 7.22 (m, 1H), 7.70 (dt, *J* = 7.7, 1.0 Hz, 1H), 7.49–7.46 (m, 3H), 7.40–7.37 (m, 2H), 7.32–7.25 (m, 3H), 7.23 (ddd, *J* = 8.2, 2.6, 0.9, 1H), 6.95 (d, *J* = 8.2 Hz, 1H), 6.93 (d, *J* = 2.0, 1H), 6.87 (dd, *J* = 8.2, 2.1 Hz, 1H), 5.20 (s, 2H), 4.33 (s, 4H), 3.95 (s, 3H), 2.17 (s, 3H); ^**13**^**C NMR** (151 MHz, CDCl_3_): δ [ppm] 166.9, 158.8, 143.1, 142.9, 142.6, 142.5, 142.4, 136.5, 135.8, 133.1, 131.5, 129.5, 129.2, 129.2, 128.8, 128.6, 128.4, 126.0, 125.4, 122.6, 122.3, 120.3, 118.2, 116.9, 115.2, 70.2, 64.5, 52.2, 18.8; **IR (ATR)** [cm^–1^]: 2950, 1722, 1507, 1279; **LC–MS (DAD/ESI)**: *t*_R_ = 9.81 min, calcd for C_30_H_26_O_5_ [*m*/*z*] [M + H]^+^ 467.19; found, [M + H]^+^ 467.28; **HRMS (ESI)**: calcd for C_30_H_26_O_5_ [*m*/*z*] [M + Na]^+^ 489.1672; found, [M + Na]^+^ 489.1671.

#### 2-Bromo-5-((3′-(benzo-1,4-dioxan-6-yl)-2′-methyl-[1,1′-biphenyl]-3-yl)methoxy)benzaldehyde (**5k**)

(3′-(Benzo-1,4-dioxan-6-yl)-2′-methyl-[1,1′-biphenyl]-3-yl)methanol (**4b**) (0.60 g, 1.81 mmol, 1.0 equiv), SOCl_2_ (0.40 mL, 5.43 mmol, 3.0 equiv), K_2_CO_3_ (0.75 g, 5.43 mmol, 3.0 equiv), 2-bromo-5-hydroxybenzaldehyde (0.55 g, 2.72 mmol, 1.5 equiv), KI (0.03 g, 0.18 mmol, 0.1 equiv). Product **5k** was obtained as a colorless solid with 78.2% (0.73 g) yield.

**R**_**f**_ = 0.65 (SiO_2_, CH_2_Cl_2_); ^**1**^**H NMR** (600 MHz, CDCl_3_): δ [ppm] 10.34 (s, 1H), 7.57–7.55 (m, 2H), 7.49–7.45 (m, 2H), 7.43 (m, 1H), 7.37 (dt, *J* = 7.5, 1.4 Hz, 1H), 7.31–7.28 (m, 1H), 7.26–7.23 (m, 2H), 7.14 (dd, *J* = 8.8, 3.2 Hz, 1H), 6.94 (d, *J* = 8.2 Hz, 1H), 6.91 (d, *J* = 2.0 Hz, 1H), 6.86 (dd, *J* = 8.2, 2.1 Hz, 1H), 5.17 (s, 2H), 4.33 (s, 4H), 2.15 (s, 3H); ^**13**^**C NMR** (151 MHz, CDCl_3_): δ [ppm] 191.7, 158.4, 143.1, 143.0, 142.6, 142.4, 142.3, 135.8, 135.7, 134.7, 134.0, 133.1, 129.4, 129.3, 128.8, 128.5, 128.5, 125.9, 125.4, 123.8, 122.6, 118.3, 118.2, 116.9, 113.9, 70.5, 64.5, 18.8; **IR (ATR)** [cm^–1^]: 2976, 2928, 2873, 1692, 1507, 1467, 1278, 1229, 1069; **LC–MS (DAD/ESI)**: *t*_R_ = 10.13 min, calcd for C_29_H_23_BrO_4_ [*m*/*z*] [M + H]^+^ 515.08; found, [M + H]^+^ 515.22; **HRMS (ESI)**: calcd for C_29_H_23_BrO_4_ [*m*/*z*] [M + Na]^+^ 537.0672; found, [M + Na]^+^ 537.0672.

#### Methyl 3-((3′-(Benzo-1,4-dioxan-6-yl)-2′-methyl-[1,1′-biphenyl]-3-yl)methoxy)isoxazole-5-carboxylate (**5m**)

(3′-(Benzo-1,4-dioxan-6-yl)-2′-methyl-[1,1′-biphenyl]-3-yl)methanol (**4b**) (0.30 g, 0.90 mmol, 1.0 equiv), SOCl_2_ (0.20 mL, 2.70 mmol, 3.0 equiv), K_2_CO_3_ (0.37 g, 2.70 mmol, 3.0 equiv), methyl 3-hydroxyisoxazole-5-carboxylate (0.13 g, 0.90 mmol, 1.0 equiv), KI (0.015 g, 0.09 mmol, 0.1 equiv). Product **5m** was obtained as a colorless solid with 47.8% (0.197 g) yield.

**R**_**f**_ = 0.42 (SiO_2_, hexane/ethyl acetate 2:1); ^**1**^**H NMR** (600 MHz, CDCl_3_): δ [ppm] 7.47–7.44 (m, 2H), 7.42 (m, 1H), 7.37 (dt, *J* = 7.4, 1.6 Hz, 1H), 7.28–7.25 (m, 1H), 7.23–7.20 (m, 2H), 6.91 (d, *J* = 8.2 Hz, 1H), 6.88 (d, *J* = 2.0 Hz, 1H), 6.83 (dd, *J* = 8.2, 2.1 Hz, 1H), 6.58 (s, 1H), 5.37 (s, 2H), 4.30 (s, 4H), 3.95 (s, 3H), 2.12 (s, 3H); ^**13**^**C NMR** (151 MHz, CDCl_3_): δ [ppm] 171.3, 160.4, 157.1, 143.1, 143.0, 142.6, 142.4, 142.2, 135.7, 135.1, 133.1, 129.7, 129.3, 129.3, 128.8, 128.4, 126.7, 125.4, 122.6, 118.2, 116.9, 101.0, 72.1, 64.5, 52.9, 18.8; **IR (ATR)** [cm^–1^]: 3140, 1743, 1506, 1303; **LC–MS (DAD/ESI)**: *t*_R_ = 9.07 min, calcd for C_27_H_23_NO_6_ [*m*/*z*] [M + H]^+^ 458.16; found, [M + H]^+^ 458.37; **HRMS (ESI)**: calcd for C_27_H_23_NO_6_ [*m*/*z*] [M + Na]^+^ 480.1418; found, [M + Na]^+^ 480.1418.

#### Methyl 3-((3′-(Benzo-1,4-dioxan-6-yl)-2′-methyl-[1,1′-biphenyl]-2-yl)methoxy)benzoate (**5o**)

(3′-(Benzo-1,4-dioxan-6-yl)-2′-methyl-[1,1′-biphenyl]-2-yl)methanol (**4c**) (0.30 g, 0.90 mmol, 1.0 equiv), SOCl_2_ (0.20 mL, 2.70 mmol, 3.0 equiv), K_2_CO_3_ (0.37 g, 2.70 mmol, 3.0 equiv), methyl 2-hydroxybenzoate (0.20 g, 1.35 mmol, 1.5 equiv), KI (0.015 g, 0.09 mmol, 0.1 equiv). Product **5o** was obtained as a colorless solid with 49.0% (0.206 g) yield.

**R**_**f**_ = 0.58 (SiO_2_, CH_2_Cl_2_); ^**1**^**H NMR** (600 MHz, CDCl_3_): δ [ppm] 7.65 (m, 2H), 7.53 (m, 1H), 7.46–7.42 (m, 2H), 7.34–7.30 (m, 2H), 7.28–7.24 (m, 2H), 7.21 (dd, *J* = 7.0, 1.8 Hz, 1H), 7.04 (dd, *J* = 8.2, 2.6 Hz, 1H), 6.94 (d, *J* = 8.2 Hz, 1H), 6.88 (d, *J* = 2.0 Hz, 1H), 6.81 (dd, *J* = 8.2, 2.0 Hz, 1H), 4.92 (s, 2H), 4.32 (s, 4H), 3.93 (s, 3H), 2.05 (s, 3H); ^**13**^**C NMR** (151 MHz, CDCl_3_): δ [ppm] 166.9, 158.7, 143.1, 142.6, 142.2, 141.7, 140.5, 135.6, 134.3, 133.6, 131.4, 123.0, 129.4, 129.4, 128.5, 128.3, 128.0, 127.6, 125.2, 122.5, 122.2, 120.1, 118.2, 116.9, 115.3, 68.3, 64.4, 52.2, 18.3; **IR (ATR)** [cm^–1^]: 2949, 1722, 1507, 1278; **LC–MS (DAD/ESI)**: *t*_R_ = 9.75 min, calcd for C_30_H_26_O_5_ [*m*/*z*] [M + H]^+^ 467.19, [M-OCH_3_]^+^ 435.16; found, [M-OCH_3_]^+^ 435.31; **HRMS (ESI)**: calcd for C_30_H_26_O_5_ [*m*/*z*] [M + Na]^+^ 489.1672; found, [M + Na]^+^ 489.1672.

#### Methyl 3-((3′-(Benzo-1,4-dioxan-6-yl)-2′-methyl-[1,1′-biphenyl]-2-yl)methoxy)isoxazole-5-carboxylate (**5q**)

(3′-(Benzo-1,4-dioxan-6-yl)-2′-methyl-[1,1′-biphenyl]-2-yl)methanol (**4c**) (0.30 g, 0.90 mmol, 1.0 equiv), SOCl_2_ (0.20 mL, 2.70 mmol, 3.0 equiv), K_2_CO_3_ (0.37 g, 2.70 mmol, 3.0 equiv), methyl 3-hydroxyisoxazole-5-carboxylate (0.13 g, 0.90 mmol, 1.0 equiv), KI (0.015 g, 0.09 mmol, 0.1 equiv). Product **5q** was obtained as a colorless solid with 42.1% (0.173 g) yield.

**R**_**f**_ = 0.35 (SiO_2_, hexane/ethyl acetate 2:1); ^**1**^**H NMR** (600 MHz, CDCl_3_): δ [ppm] 7.50 (m, 1H), 7.35–7.31 (m, 2H), 7.19–7.17 (m, 1H), 7.13–7.10 (m, 2H), 7.02 (dd, *J* = 6.4, 2.6 Hz, 1H), 6.81 (d, *J* = 8.2 Hz, 1H), 6.75 (d, *J* = 2.0 Hz, 1H), 6.69 (dd, *J* = 8.2, 2.1 Hz, 1H), 6.38 (s, 1H), 5.05 (d, *J* = 11.7 Hz, 1H), 5.00 (d, *J* = 11.7 Hz, 1H), 4.20 (s, 4H), 3.84 (s, 3H), 1.86 (s, 3H); ^**13**^**C NMR** (151 MHz, CDCl_3_): δ [ppm] 171.2, 160.2, 157.1, 143.1, 142.6, 142.4, 142.2, 140.2, 135.5, 133.5, 133.1, 130.1, 129.5, 129.2, 128.7, 128.4, 127.6, 125.2, 122.5, 118.2, 116.8, 100.8, 70.3, 64.4, 52.8, 18.2; **IR (ATR)** [cm^–1^]: 3140, 2953, 1743, 1505, 1288; **LC–MS (DAD/ESI)**: *t*_R_ = 9.00 min, calcd for C_27_H_23_NO_6_ [*m*/*z*] [M + H]^+^ 458.16; found, [M + H]^+^ 458.30; **HRMS (ESI)**: calcd for C_27_H_23_NO_6_ [*m*/*z*] [M + Na]^+^ 480.1418; found, [M + Na]^+^ 480.1418.

#### Methyl 1-((3′-(Benzo-1,4-dioxan-6-yl)-2′-methyl-[1,1′-biphenyl]-4-yl)methyl)-1*H*-1,2,3-triazole-4-carboxylate (**5g**)

(3′-(Benzo-1,4-dioxan-6-yl)-2′-methyl-[1,1′-biphenyl]-4-yl)methanol (**4a**) (0.33 g, 1.0, 1.0 equiv) was added in a round-bottom flask with anhydrous dichloromethane and triethylamine (0.21 mL, 1.5 mmol, 1.5 equiv) and cooled in an ice bath; then, mesyl chloride (0.12 mL, 1.5 mmol, 1.5 equiv) was added dropwise. The reaction mixture was stirred approximately 2 h at room temperature and controlled by TLC. When all substrate disappeared, the reaction mixture was poured onto water and extracted with dichloromethane. The organic phases were collected, dried over anhydrous MgSO_4_, and evaporated. The crude product was dissolved in anhydrous DMF, and NaN_3_ (0.097 g, 1.5 mmol, 1.5 equiv) was added. The reaction mixture was stirred at room temperature for 6 days. After this time, water was added and the reaction mixture was extracted with ethyl acetate. The organic layers were collected, washed with brine, dried over anhydrous MgSO_4_, and evaporated. Crude azide was roughly purified with flash chromatography on a silica pad (SiO_2_, hexane/ethyl acetate 4:1). The obtained azide (0.25 g, 0.69 mmol, 1.0 equiv) was added to a round-bottom flask, and then, methyl propiolate (0.041 mL, 0.69 mmol, 1.0 equiv) in a THF/water mixture (1:2), sodium ascorbate (0.068 g, 0.32 mmol, 0.5 equiv), and copper(II) sulfate pentahydrate (0.034 g, 0.14 mmol, 0.2 equiv) were added. The reaction mixture was stirred at room temperature for 24 h. After this time, water was added, and the reaction mixture was extracted with ethyl acetate. The organic layers were collected, washed with brine, dried over anhydrous MgSO_4_, and evaporated. The crude product was crystallized from the cyclohexane/ethyl acetate (4:1), giving the final product **5g** with 72.1% (0.220 g)

**R**_**f**_ = 0.88 (SiO_2_, ethyl acetate/methanol 1:1); ^**1**^**H NMR (600 MHz, CDCl**_**3**_**)**: δ [ppm] 8.00 (s, 1H), 7.32 (d, *J* = 8.2 Hz, 2H), 7.27 (d, *J* = 8.2 Hz, 2H), 7.19 (t, *J* = 7.5 Hz, 1H), 7.15 (dd, *J* = 7.6, 1.6 Hz, 1H), 7.09 (dd, *J* = 7.3, 1.6 Hz, 1H), 6.83 (d, *J* = 8.2 Hz, 1H), 6.79 (d, *J* = 2.0 Hz, 1H), 6.74 (dd, *J* = 8.2, 2.1 Hz, 1H), 5.56 (s, 2H), 4.23 (s, 4H), 3.87 (s, 3H), 2.03 (s, 3H); ^**13**^**C NMR (151 MHz, CDCl**_**3**_**)**: δ [ppm] 161.3, 143.7, 143.2, 142.8, 142.6, 141.8, 140.5, 135.7, 133.1, 132.2, 130.4, 129.6, 128.8, 128.2, 127.6, 125.6, 122.6, 118.3, 117.0, 64.6, 64.6, 54.4, 52.4, 18.9; **IR (ATR)** [cm^–1^]: 3142, 2999, 1726, 1509, 1456, 1509, 1438, 1360, 1318, 1244, 1068, 1047, 901, 813, 780; **LC–MS (DAD/ESI)**: *t*_R_ = 7.78 min, calcd for C_26_H_23_N_3_O_4_ [*m*/*z*] [M + H]^+^ 442.18; found, [M + H]^+^ 442.30; **HRMS (ESI)**: calcd for C_26_H_23_N_3_O_4_ (*m*/*z*) [M + Na]^+^ 464.1581; found, [M + Na]^+^ 464.1581.

### General Procedure of Ester Saponification (**5b**, **5f**, **5h**, **5j**, **5n**, **5p**, and **5r**)

The appropriate ester was added in a one-necked round-bottom flask and dissolved in a dioxane/water (2:1, 10:5 mL per 1 mmol of ester) mixture. After complete dissolution, LiOH monohydrate was added and the mixture was stirred at room temperature overnight. The progression of the reaction was controlled using TLC analysis (SiO_2_, ethyl acetate/methanol, or dichloromethane (DCM)/methanol). After completion of the reaction, the mixture was transferred into a separatory funnel, 7 mL of water was added, and then extracted once with a small amount of ethyl acetate. The aqueous phase was acidified to pH ≈ 4 and extracted three times with ethyl acetate. The organic phases obtained from extraction after acidification were collected, washed with brine, dried over anhydrous MgSO_4_, and the solvent was evaporated. Further purification of the product was not required.

#### 3-((3′-(Benzo-1,4-dioxan-6-yl)-2′-methyl-[1,1′-biphenyl]-4-yl)methoxy)benzoic Acid (**5b**)

Methyl 3-((3′-(benzo-1,4-dioxan-6-yl)-2′-methyl-[1,1′-biphenyl]-4-yl)methoxy)benzoate (**5a**) (0.093 g, 0.20 mmol, 1.0 equiv), LiOH·H_2_O (0.016 g, 0.40 mmol, 2.0 equiv). Product **5b** was obtained as a colorless solid with 88.8% (0.080 g) yield.

**R**_**f**_ = 0.44 (SiO_2_, DCM/MeOH, 9:1); ^**1**^**H NMR** (600 MHz, DMSO-*d*_6_): δ [ppm] 7.59–7.57 (m, 2H), 7.53 (d, *J* = 8.0 Hz, 2H), 7.43 (t, *J* = 7.8 Hz, 1H), 7.39 (d, *J* = 8.0 Hz, 2H), 7.30 (dd, *J* = 8.1, 2.4 Hz, 1H), 7.28–7.26 (m, 1H), 7.17 (d, *J* = 7.6 Hz, 2H), 6.91 (d, *J* = 8,2 Hz, 1H), 6.86 (d, *J* = 2.0, 1H), 6.81 (dd, *J* = 8.2, 2.0 Hz, 1H), 5.21 (s, 2H), 4.27 (s, 4H), 2.07 (s, 3H); ^**13**^**C NMR** (151 MHz, DMSO-*d*_6_): δ [ppm] 167.6, 158.9, 143.4, 143.0, 142.4, 142.3, 141.9, 135.9, 135.3, 132.8, 132.7, 130.3, 129.7, 129.3, 129.0, 128.1, 126.0, 122.6, 122.3, 120.2, 118.2, 117.2, 115.3, 69.7, 64.6, 64.6, 19.1; **IR (ATR)** [cm^–1^]: 2981, 2572, 1683, 1506, 1454, 1314, 1245; **LC–MS (DAD/ESI)**: *t*_R_ = 8.67 min, calcd for C_29_H_24_O_5_ [*m*/*z*] [M – H]^−^ 451.16; found, [M – H]^−^ 451.19; **HRMS (ESI)**: calcd for C_29_H_24_O_5_ [*m*/*z*] [M + Na]^+^ 475.1516; found, [M + Na]^+^ 475.1517.

### 3-((3′-(Benzo-1,4-dioxan-6-yl)-2′-methyl-[1,1′-biphenyl]-4-yl)methoxy)isoxazole-5-carboxylic Acid (**5f**)

Methyl 3-((3′-(benzo-1,4-dioxan-6-yl)-2′-methyl-[1,1′-biphenyl]-4-yl)methoxy)isoxazole-5-carboxylate (**5e**) (0.090 g, 0.20 mmol, 1.0 equiv), LiOH·H_2_O (0.016 g, 0.40 mmol, 2.0 equiv). Product **5f** was obtained as a colorless solid with 65.2% (0.058 g) yield.

**R**_**f**_ = 0.14 (SiO_2_, ethyl acetate/MeOH, 3:1); ^**1**^**H NMR** (600 MHz, DMSO-*d*_6_): δ [ppm] 7.56 (d, *J* = 8.0 Hz, 2H), 7.42 (d, *J* = 8.0 Hz, 2H), 7.29 (t, *J* = 7.6 Hz, 1H), 7.18 (d, *J* = 7.7 Hz, 2H), 7.00 (s, 1H), 6.92 (d, *J* = 8.2 Hz, 1H), 6.86 (d, *J* = 2.0 Hz, 1H), 6.82 (dd, *J* = 8.2, 2.0 Hz, 1H), 2.17 (s, 2H), 4.28 (s, 4H), 2.07 (s, 3H) ^**13**^**C NMR** (151 MHz, DMSO-*d*_6_): δ [ppm] 171.8, 162.2, 158.0, 143.4, 143.0, 142.5, 142.4, 142.3, 135.3, 134.6, 132.8, 129.8, 129.4, 129.0, 128.9, 126.1, 122.6, 118.2, 117.3, 100.7, 71.9, 64.6, 64.6, 19.1; **IR (ATR)** [cm^–1^]: 2929, 2606, 1727, 1497, 1274; **LC–MS (DAD/ESI)**: *t*_R_ = 7.89 min, calcd for C_26_H_21_NO_6_ [*m*/*z*] [M – H]^−^ 442.13; found, [M – H]^−^ 442.15; **HRMS (ESI)**: calcd for C_26_H_21_NO_6_ [*m*/*z*] [M + Na]^+^ 466.1261; found, [M + Na]^+^ 466.1263.

#### 1-((3′-(Benzo-1,4-dioxan-6-yl)-2′-methyl-[1,1′-biphenyl]-4-yl)methyl)-1*H*-1,2,3-triazole-4-carboxylic Acid (**5h**)

Methyl 1-((3′-(benzo-1,4-dioxan-6-yl)-2′-methyl-[1,1′-biphenyl]-4-yl)methyl)-1H-1,2,3-triazole-4-carboxylate (**5g**) (0.150 g, 0.34 mmol, 1.0 equiv), LiOH·H_2_O (0.042 g, 1.00 mmol, 3.0 equiv). Product **5h** was obtained as a colorless solid with 61.4% (0.089 g) yield.

**R**_**f**_ = 0.39 (SiO_2_, ethyl acetate/methanol 1:1); ^**1**^**H NMR** (600 MHz, CDCl_3_): δ [ppm] 8.85 (s, 1H), 7.42–7.37 (m, 4H), 7.26 (t, *J* = 7.6 Hz, 1H), 7.17–7.13 (m, 2H), 6.91 (d, *J* = 8.2 Hz, 1H), 6.84 (d, *J* = 2.0 Hz, 1H), 6.80 (dd, *J* = 8.2, 2.0 Hz, 1H), 5.71 (s, 2H), 4.27 (s, 4H), 2.04 (s, 3H); ^**13**^**C NMR** (151 MHz, CDCl_3_): δ [ppm] 162.1, 143.4, 134.0, 142.3, 142.2, 140.4, 135.2, 134.7, 132.8, 130.1, 129.6, 129.4, 129.0, 128.3, 126.1, 122.6, 118.2, 117.2, 64.6, 53.2, 19.1; **IR (ATR)** [cm^–1^]: 3115, 2977, 2874, 2559, 1687, 1506, 1439, 1229, 1071, 1053, 899, 783; **LC–MS (DAD/ESI)**: *t*_R_ = 6.99 min, calcd for C_25_H_21_N_3_O_4_ [*m*/*z*] [M + H]^+^ 428.16; found, [M + H]^+^ 428.28; **HRMS (ESI)**: calcd for C_25_H_21_N_3_O_4_ (*m*/*z*) [M + Na]^+^ 450.1424; found, [M + Na]^+^ 450.1422.

#### 3-((3′-(Benzo-1,4-dioxan-6-yl)-2′-methyl-[1,1′-biphenyl]-3-yl)methoxy)benzoic Acid (**5j**)

Methyl 3-((3′-(benzo-1,4-dioxan-6-yl)-2′-methyl-[1,1′-biphenyl]-3-yl)methoxy)benzoate (**5i**) (0.093 g, 0.20 mmol, 1.0 equiv), LiOH·H_2_O (0.016 g, 0.40 mmol, 2.0 equiv). Product **5j** was obtained as a colorless solid with 58.8% (0.053 g) yield.

**R**_**f**_ = 0.58 (SiO_2_, DCM/MeOH, 9:1); ^**1**^**H NMR** (600 MHz, CDCl_3_): δ [ppm] 7.66–7.64 (m, 2H), 7.38–7.34 (m, 3H), 7.30 (t, *J* = 8.2 Hz, 1H), 7.25 (dt, *J* = 6.9, 1.7 Hz, 1H), 7.19–7.13 (m, 4H), 6.82 (d, *J* = 8.2 Hz, 1H), 6.80 (d, *J* = 2.0 Hz, 1H), 6.75 (dd, *J* = 8.2, 2.0 Hz, 1H), 5.09 (s, 2H), 4.21 (s, 4H), 2.04 (s, 3H); ^**13**^**C NMR** (151 MHz, CDCl_3_): δ [ppm] 171.7, 158.8, 143.1, 142.9, 142.6, 142.5, 142.4, 136.4, 135.8, 133.1, 130.7, 129.6, 129.2, 129.2, 128.8, 128.6, 128.5, 126.0, 125.4, 123.0, 122.6, 121.3, 118.2, 116.9, 115.6, 70.2, 64.5, 18.8; **IR (ATR)** [cm^–1^]: 2922, 2565, 1679, 1506, 1455, 1302, 1247; **LC–MS (DAD/ESI)**: *t*_R_ = 8.68 min, calcd for C_29_H_24_O_5_ [*m*/*z*] [M – H]^−^ 451.16; found, [M – H]^−^ 451.12; **HRMS (ESI)**: calcd for C_29_H_24_O_5_ [*m*/*z*] [M + Na]^+^ 475.1516; found, [M + Na]^+^ 475.1516.

#### 3-((3′-(Benzo-1,4-dioxan-6-yl)-2′-methyl-[1,1′-biphenyl]-3-yl)methoxy)isoxazole-5-carboxylic Acid (**5n**)

Methyl 3-((3′-(benzo-1,4-dioxan-6-yl)-2′-methyl-[1,1′-biphenyl]-3-yl)methoxy)isoxazole-5-carboxylate (**5m**) (0.090 g, 0.20 mmol, 1.0 equiv), LiOH·H_2_O (0.016 g, 0.40 mmol, 2.0 equiv). Product **5n** was obtained as a colorless solid with 45.1% (0.040 g) yield.

**R**_**f**_ = 0.31 (SiO_2_, ethyl acetate/MeOH, 3:1); ^**1**^**H NMR** (600 MHz, CDCl_3_): δ [ppm] 7.47–7.45 (m, 2H), 7.43 (m, 1H), 7.37 (dt, *J* = 7.4, 1.4 Hz, 1H), 7.28–7.25 (m, 1H), 7.23–7.20 (m, 2H), 6.91 (d, *J* = 8.2 Hz, 1H), 6.88 (d, *J* = 2.0 Hz, 1H), 6.83 (dd, *J* = 8.2, 2.1 Hz, 1H), 6.68 (s, 1H), 5.38 (s, 2H), 4.31 (s, 4H), 2.12 (s, 3H); ^**13**^**C NMR** (151 MHz, CDCl_3_): δ [ppm] 171.4, 159.7, 159.3, 143.1, 143.0, 142.6, 142.4, 142.2, 135.7, 135.0, 133.1, 129.8, 129.3, 129.3, 128.8, 128.5, 126.7, 125.4, 122.6, 118.2, 116.9, 102.2, 72.3, 64.5, 64.5, 18.8; **IR (ATR)** [cm^–1^]: 3147, 2954, 2614, 360, 1736, 1505; **LC–MS (DAD/ESI)**: *t*_R_ = 7.79 min, calcd for C_26_H_21_NO_6_ [*m*/*z*] [M – H]^−^ 442.13; found, [M – H]^−^ 442.15; **HRMS (ESI)**: calcd for C_26_H_21_NO_6_ [*m*/*z*] [M + Na]^+^ 466.1261; found, [M + Na]^+^ 466.1260.

#### 3-((3′-(Benzo-1,4-dioxan-6-yl)-2′-methyl-[1,1′-biphenyl]-2-yl)methoxy)benzoic Acid (**5p**)

Methyl 3-((3′-(benzo-1,4-dioxan-6-yl)-2′-methyl-[1,1′-biphenyl]-2-yl)methoxy)benzoate (**5o**) (0.093 g, 0.20 mmol, 1.0 equiv), LiOH·H_2_O (0.016 g, 0.40 mmol, 2.0 equiv). Product **5p** was obtained as a colorless solid with 58.8% (0.053 g) yield.

**R**_**f**_ = 0.70 (SiO_2_, DCM/MeOH, 9:1); ^**1**^**H NMR** (600 MHz, DMSO-*d*_6_): δ [ppm] 12.96 (s, 1H), 7.61 (m, 1H), 7.50 (d, *J* = 7.7 Hz, 1H), 7.43 (m, 2H), 7.35 (t, *J* = 7.9 Hz, 1H), 7.31 (m, 1H), 7.27–7.21 (m, 2H), 7.14 (t, *J* = 7.5 Hz, 2H), 7.06 (dd, *J* = 8.2, 2.1 Hz, 1H), 6.88 (d, *J* = 8.2 Hz, 1H), 6.77 (d, *J* = 2.0 Hz, 1H), 6.72 (dd, *J* = 8.3, 2.0 Hz, 1H), 4.89 (d, *J* = 11.5 Hz, 1H), 4.85 (d, *J* = 11.5 Hz, 1H), 4.27 (s, 4H), 1.92 (s, 3H); ^**13**^**C NMR** (151 MHz, DMSO-*d*_6_): δ [ppm] 167.5, 158.7, 143.4, 142.9, 142.0, 142.0, 140.8, 135.2, 134.5, 133.4, 132.6, 130.2, 130.1, 129.5, 129.4, 128.6, 128.5, 128,00, 125.7, 122.5, 122.2, 119.9, 118.1, 117.2, 115.2, 68.3, 66.8, 64.6, 18.6; **IR (ATR)** [cm^–1^]: 2876, 2647, 1690, 1507, 1279, 1244; **LC–MS (DAD/ESI)**: *t*_R_ = 8.61 min, calcd for C_29_H_24_O_5_ [*m*/*z*] [M – H]^−^ 451.16; found, [M – H]^−^ 451.12 **HRMS (ESI)**: calcd for C_29_H_24_O_5_ [*m*/*z*] [M + Na]^+^ 475.1516; found, [M + Na]^+^ 475.1516.

#### 3-((3′-(Benzo-1,4-dioxan-6-yl)-2′-methyl-[1,1′-biphenyl]-2-yl)methoxy)isoxazole-5-carboxylic Acid (**5r**)

Methyl 3-((3′-(benzo-1,4-dioxan-6-yl)-2′-methyl-[1,1′-biphenyl]-2-yl)methoxy)isoxazole-5-carboxylate (**5q**) (0.090 g, 0.20 mmol, 1.0 equiv), LiOH·H_2_O (0.016 g, 0.40 mmol, 2.0 equiv). Product **5r** was obtained as a colorless solid with 55.3% (0.049 g) yield.

**R**_**f**_ = 0.45 (SiO_2_, ethyl acetate/MeOH, 1:3); ^**1**^**H NMR** (600 MHz, CDCl_3_): δ [ppm] 7.21 (m, 1H), 7.46–7.41 (m, 2H), 7.26–7.23 (m, 2H), 7.16 (dd, *J* = 7.6, 0.8 Hz, 1H), 7.09 (dd, *J* = 7.4, 0.9 Hz, 1H), 6.90 (d, *J* = 8.2 Hz, 1H), 6.82 (d, *J* = 2.0 Hz, 1H), 6.76 (dd, *J* = 8.3, 2.0 Hz, 1H), 6.17 (s, 1H), 4.97 (d, *J* = 11.8 Hz, 1H), 4.93 (d, *J* = 11.8 Hz, 1H), 4.27 (s, 4H), 1.88 (s, 3H); ^**13**^**C NMR** (151 MHz, CDCl_3_): δ [ppm] 171.2, 170.6, 159.5, 143.4, 143.0, 142.0, 140.6, 135.1, 133.9, 133.3, 130.1, 129.5, 128.9, 128.6, 128.0, 125.7, 122.6, 118.1, 117.3, 95.6, 69.4, 64.6, 18.5; **IR (ATR)** [cm^–1^]: 3396, 2931, 1595, 1505, 1359; **LC–MS (DAD/ESI)**: *t*_R_ = 7.78 min, calcd for C_26_H_21_NO_6_ [*m*/*z*] [M – H]^−^ 442.13; found, [M – H]^−^ 442.15; **HRMS (ESI)**: calcd for C_26_H_21_NO_6_ [M + Na]^+^ 466.1261; found, [M + Na]^+^ 466.1262.

### General Procedure for 2-Pyridinebenzaldehyde Preparation (**5d** and **5l**)

A three-neck round-bottom flask was charged with appropriate halide **5c** or **5k**, borane 3-pyridinylboronic acid, K_2_CO_3_, and a dioxane/water mixture (2:1, 5/10 mL for 1 mmol) under an argon atmosphere. The mixture was deoxygenated by rinsing with argon for half an hour; then, the Pd(dppf)Cl_2_ dichloromethane complex was added. The reaction mixture was heated at 80 °C, using preheated bath, for 5 h. After this time, the progression of the reaction was controlled by TLC. When the reaction was complete, water was added and the extraction with ethyl acetate followed. The organic phases were combined, dried over anhydrous MgSO_4_, and evaporated. The crude product was purified by flash chromatography (SiO_2_, hexane/ethyl acetate), giving the final products **5d** or **5l** with 93 and 80% yields, respectively.

#### 5-((3′-(Benzo-1,4-dioxan-6-yl)-2′-methyl-[1,1′-biphenyl]-4-yl)methoxy)-2-(pyridin-3-yl)benzaldehyde (**5d**)

2-Bromo-5-((3′-(benzo-1,4-dioxan-6-yl)-2′-methyl-[1,1′-biphenyl]-4-yl)methoxy)benzaldehyde (**5c**) (0.20 g, 0.40 mmol, 1.0 equiv), 3-pyridinylboronic acid (0.072 g, 0.60 mmol, 1.5 equiv), K_2_CO_3_ (0.16 g, 1.20 mmol, 3.0 equiv), Pd(dppf)Cl_2_ complex with dichloromethane (0.016 g, 0.02 mmol, 0.05 equiv). Product **5d** was obtained as a colorless solid with 92.6% (0.190 g) yield.

**R**_**f**_ = 0.37 (SiO_2_, ethyl acetate); ^**1**^**H NMR** (600 MHz, CDCl_3_): δ [ppm] 9.95 (s, 1H), 8.68 (dd, *J* = 4.9, 1.5 Hz, 1H), 8.66 (m, 1H), 7.71–7.69 (m, 2H), 7.53 (d, *J* = 8.0 Hz, 2H), 7.42–7.38 (m, 4H), 7.35 (dd, *J* = 8.4, 2.7 Hz, 1H), 7.27 (m, 1H), 7.24–7.21 (m, 2H), 6.91 (d, *J* = 8.2 Hz, 1H), 6.89 (d, *J* = 2.0 Hz, 1H), 6.83 (dd, *J* = 8.2, 2.0 Hz, 1H), 5.23 (s, 2H), 4.31 (s, 4H), 2.15 (s, 3H); ^**13**^**C NMR** (151 MHz, CDCl_3_): δ [ppm] 191.1, 159.1, 150.3, 149.1, 143.0, 142.6, 142.4, 142.3, 137.4, 135.7, 135.1, 134.8, 134.6, 133.4, 133.1, 132.4, 129.7, 129.2, 128.8, 127.4, 125.3, 123.2, 122.5, 122.2, 118.2, 116.9, 112.0, 70.3, 64.4, 64.4, 18.8; **IR (ATR)** [cm^–1^]: 3049, 2997, 2876, 1684, 1504, 1320, 1169; **LC–MS (DAD/ESI)**: *t*_R_ = 8.56 min, calcd for C_34_H_27_NO_4_ [*m*/*z*] [M + H]^+^ 514.19; found, [M + H]^+^ 514.33; **HRMS (ESI)**: calcd for C_34_H_27_NO_4_: [M + Na]^+^ 536.1832; found, [M + Na]^+^ 536.1830.

#### 5-((3′-(Benzo-1,4-dioxan-6-yl)-2′-methyl-[1,1′-biphenyl]-3-yl)methoxy)-2-(yridine-3-yl)benzaldehyde (**5l**)

2-Bromo-5-((3′-(benzo-1,4-dioxan-6-yl)-2′-methyl-[1,1′-biphenyl]-3-yl)methoxy)benzaldehyde (**5k**) (0.20 g, 0.40 mmol, 1.0 equiv), 3-pyridinylboronic acid (0.072 g, 0.60 mmol, 1.5 equiv), K_2_CO_3_ (0.160 g, 1.20 mmol, 3.0 equiv), Pd(dppf)Cl_2_ complex with dichloromethane (0.016 g, 0.02 mmol, 0.05 equiv). Product **5l** was obtained as a colorless solid with 79.9% (0.164 g) yield.

**R**_**f**_ = 0.51 (SiO_2_, ethyl acetate); ^**1**^**H NMR** (600 MHz, CDCl_3_): δ [ppm] 9.96 (s, 1H), 8.70 (dd, *J* = 4.9, 1.6 Hz, 1H), 8.67 (d, *J* = 1.7 Hz, 1H), 7.72–7.70 (m, 1H), 7.69 (d, *J* = 2.7 Hz, 1H), 7.51–7.46 (m, 3H), 7.43 (dd, *J* = 4.9, 0.7 Hz, 1H), 7.40–7.37 (m, 2H), 7.35 (dd, *J* = 8.5, 2.7 Hz, 1H), 7.31–7.28 (m, 1H), 7.26–7.24 (m, 2H), 6.94 (d, *J* = 8,2 Hz, 1H), 6.91 (d, *J* = 2.0 Hz, 1H), 6.86 (dd, *J* = 8.2, 2.1 Hz, 1H), 5.26 (s, 2H), 4.33 (s, 4H), 2.15 (s, 3H); ^**13**^**C NMR** (151 MHz, CDCl_3_): δ [ppm] 191.1, 159.0, 150.3, 149.0, 143.1, 143.0, 142.6, 142.4, 137.4, 136.0, 135.7, 135.0, 134.8, 133.5, 133.1, 132.4, 129.3, 129.3, 128.8, 128.6, 128.5, 126.0, 125.4, 123.2, 122.6, 122.2, 118.2, 116.9, 112.1, 70.4, 64.5, 64.5, 18.8; **IR (ATR)** [cm^–1^]: 2974, 2927, 2875, 1688, 1506, 1279, 1227, 1069; **LC–MS (DAD/ESI)**: *t*_R_ = 8.59 min, calcd for C_34_H_27_NO_4_ [*m*/*z*] [M + H]^+^ 514.19; found, [M + H]^+^ 514.33; **HRMS (ESI)**: [M + Na]^+^ 536.1832; found, [M + Na]^+^ 536.1834.

### General Procedure for the Amine Formation (**6a–e**, **7a–h**, and **8a–j**)

Appropriate alcohol **4a–h** was added in a round-bottom flask with anhydrous dichloromethane and few drops of anhydrous DMF (0.2 mL for 2 mmol alcohol) and cooled in an ice bath; then, thionyl chloride (5 equiv) was added dropwise. The reaction mixture was stirred approximately 2 h at room temperature and controlled by TLC. When all substrates disappeared, the reaction mixture was poured onto saturated NaHCO_3_ and extracted with dichloromethane. The organic phases were collected, dried over anhydrous MgSO_4_, and evaporated. The crude product was used without further purification in the next step.

Method A. Crude alkyl chloride was dissolved in anhydrous acetonitrile or THF, and excess of appropriate amine dissolved in anhydrous acetonitrile or THF was added (if the hydrochloride of amine was used, an equimolar amount of triethylamine was added to the reaction mixture). The reaction mixture was stirred at room temperature for 48 h and controlled by TLC. After completion of the reaction, the mixture was poured into saturated NaHCO_3_ and extracted with ethyl acetate. The organic phases were collected, dried over anhydrous MgSO_4_, and evaporated. The crude products were purified by flash column chromatography (SiO_2_, hexane/ethyl acetate).

Method B. Crude alkyl chloride was dissolved in anhydrous THF, and excess of Boc-protected piperazine dissolved in anhydrous THF was added. The reaction mixture was stirred at room temperature 48 h and controlled by TLC. After the reaction was complete, the solvent was evaporated and the crude Boc-protected product was purified by flash chromatography (SiO_2_, hexane/ethyl acetate 1:1). The purified Boc-protected product was dissolved in anhydrous dioxane, and HCl in dioxane was added (10 equiv, 4 M). The reaction mixture was stirred at room temperature overnight and then poured into saturated NaHCO_3_ and extracted with ethyl acetate. The organic phases were collected, dried over anhydrous MgSO_4_, and evaporated. The products were purified by column chromatography (SiO_2_, pure methanol, or methanol/7 M NH_3_ in methanol).

Method C. Crude alkyl chloride was dissolved in anhydrous THF and an appropriate amine dissolved in anhydrous THF was added (if the hydrochloride of amine was used, an equimolar amount of triethylamine was added to the reaction mixture). The reaction mixture was stirred at 50 °C overnight. After completion of the reaction, it was poured into saturated NaHCO_3_ and extracted with ethyl acetate. The organic phases were collected, dried over anhydrous MgSO_4_, and evaporated. The crude products were purified by column chromatography (SiO_2_, DCM/methanol, methanol, or methanol/7 M NH_3_ in methanol).

Method D. Crude alkyl chloride was dissolved in anhydrous DMF, and an appropriate amine dissolved in anhydrous DMF was added (if the hydrochloride of the amine was used, equimolar amount of triethyl amine was added to the reaction mixture) together with diisopropylethylamine (DIPEA). The reaction mixture was stirred at 80 °C overnight. After completion of the reaction, it was poured into saturated NaHCO_3_ and extracted with ethyl acetate. The organic phases were collected, dried over anhydrous MgSO_4_, and evaporated. The crude products were purified by column chromatography (SiO_2_, ethyl acetate, DCM/methanol, or CHCl_3_/7 M NH_3_ in methanol or methanol/7 M NH_3_ in methanol).

Method E. Crude alkyl chloride was dissolved in anhydrous DMF, and an appropriate amine dissolved in anhydrous DMF was added (if the hydrochloride of the amine was used, an equimolar amount of triethylamine was added to the reaction mixture) together with DIPEA. The reaction mixture was stirred at 80 °C overnight. After completion of the reaction, it was poured into saturated NaHCO_3_, extracted three times with ethyl acetate, and the crude Boc-protected product was purified by flash chromatography (SiO_2_, hexane/ethyl acetate 1:1). The purified Boc-protected product was dissolved in anhydrous dioxane, and HCl in dioxane was added (10 equiv, 4 M). The reaction mixture was stirred at room temperature overnight, then poured into saturated NaHCO_3_, and extracted three times with ethyl acetate. The organic phases were collected, dried over anhydrous MgSO_4_, and evaporated. The products were purified by column chromatography (SiO_2_, DCM/methanol, or methanol/7 M NH_3_ in methanol).

#### 4-((3′-(Benzo-1,4-dioxan-6-yl)-2′-methyl-[1,1′-biphenyl]-4-yl)methyl)morpholine (**6a**)

Method A. (3′-(Benzo-1,4-dioxan-6-yl)-2′-methyl-[1,1′-biphenyl]-4-yl)methanol (**4a**) (0.33 g, 1.0 mmol, 1.0 equiv), thionyl chloride (0.36 mL, 5.0 mmol, 5.0 equiv), morpholine (0.35 mL, 5.0 mmol, 5.0 equiv). The crude product was purified by flash chromatography (SiO_2_, hexane/ethyl acetate 2:1), giving the final compound **6a** as a colorless oil with 70.1% (0.281 g) yield.

**R**_**f**_ = 0.36 (SiO_2_, ethyl acetate/hexane 1:1); ^**1**^**H NMR** (600 MHz, DMSO-*d*_6_): δ [ppm] 7.37 (d, *J* = 8.1 Hz, 2H), 7.32 (d, *J* = 8.1 Hz, 2H), 7.27 (t, *J* = 7.6 Hz, 1H), 7.16 (d, *J* = 7.6 Hz, 2H), 6.91 (d, *J* = 8.2 Hz, 1H), 6.85 (d, *J* = 2.1 Hz, 1H), 6.81 (dd, *J* = 8.2, 2.1 Hz, 1H), 4.28 (s, 4H), 3.62–3.57 (m, 4H), 3.51 (s, 2H), 2.43–2.32 (m, 4H), 2.06 (s, 3H); ^**13**^**C NMR** (151 MHz, DMSO-*d*_6_): δ [ppm] 142.9, 142.5, 142.1, 141.8, 140.5, 136.5, 134.9, 132.3, 29.0, 128.7, 128.5, 125.5, 122.1, 117.7, 116.8, 66.2, 64.1, 64.1, 62.2, 53.2, 18.7; **IR (ATR)** [cm^–1^]: 2957, 2923, 2842, 1582, 1506, 1455, 1317, 1292, 1242, 1228, 1114, 1070, 1006, 898, 881, 845, 791; **LC–MS (DAD/ESI)**: *t*_R_ = 5.65 min, calcd for C_26_H_27_NO_3_ (*m*/*z*) [M + H]^+^ 402.27; found, [M + H]^+^ 402.21; **HRMS (ESI)**: calcd for C_26_H_27_NO_3_ (*m*/*z*) [M + H]^+^ 402.2064; found, [M + H]^+^ 402.2062.

#### 4-((3′-(Benzo-1,4-dioxan-6-yl)-2′-methyl-[1,1′-biphenyl]-4-yl)methyl)piperazine (**6b**)

Method B. (3′-(Benzo-1,4-dioxan-6-yl)-2′-methyl-[1,1′-biphenyl]-4-yl)methanol (**4a**) (0.24 g, 0.72 mmol, 1.0 equiv), thionyl chloride (0.26 mL, 3.6 mmol, 5.0 equiv), 1-Boc-piperazine (0.67 g, 3.6 mmol, 5.0 equiv). Product **6b** was obtained as a light-yellow oil with 66.3% (0.191 g) yield.

**R**_**f**_ = 0.09 (SiO_2_, methanol); **IR (ATR)** [cm^–1^]: 3298, 2933, 2811, 1578, 1507, 1463, 1414, 1317, 1279, 1245, 1123, 1069, 899, 790; ^**1**^**H NMR (**600 MHz, DMSO-*d*_6_): δ [ppm] 7.36 (d, *J* = 8.0 Hz, 2H), 7.31 (d, *J* = 8.2 Hz, 2H), 7.27 (t, *J* = 7.4 Hz, 1H), 7.16 (dd, *J* = 7.6, 2.0 Hz, 2H), 6.92 (d, *J* = 8.2 Hz, 1H), 6.86 (d, *J* = 2.1 Hz, 1H), 6.82 (dd, *J* = 8.2, 2.1 Hz, 1H), 4.28 (s, 4H), 3.47 (s, 2H), 3.17 (s, 1H), 2.71–2.66 (m, 4H), 2.35–2.27 (m, 4H), 2.07 (s, 3H); ^**13**^**C NMR** (151 MHz, DMSO-*d*_6_): δ [ppm] 142.9, 142.5, 142.2, 141.8, 140.3, 137.0, 134.9, 132.3, 128.9, 128.7, 128.6, 125.5, 122.1, 117.7, 116.8, 64.1, 62.6, 54.2, 45.6, 18.7; **LC–MS (DAD/ESI)**: *t*_R_ = 5.01 min, calcd for C_26_H_28_N_2_O_2_ [*m*/*z*] [M + H]^+^ 401.22; found, [M + H]^+^ 401.30; **HRMS (ESI)**: calcd for C_26_H_28_N_2_O_2_ (*m*/*z*) [M + H]^+^ 401.2223; found, [M + H]^+^ 401.2226.

#### 1-((3′-(Benzo-1,4-dioxan-6-yl)-2′-methyl-[1,1′-biphenyl]-3-yl)methyl)piperazine (**6c**)

Method B. (3′-(Benzo-1,4-dioxan-6-yl)-2′-methyl-[1,1′-biphenyl]-3-yl)methanol (**4b**) (0.20 g, 0.60 mmol, 1.0 equiv), SOCl_2_ (0.22 mL, 3.0 mmol, 5.0 equiv), 1-Boc-piperazine (0.56 g, 3.0 mmol, 5.0 equiv). Product **6c** was obtained as a colorless solid with 29.1% (0.070 g) yield.

**R**_**f**_ = 0.11 (SiO_2_, MeOH); ^**1**^**H NMR** (600 MHz, CDCl_3_): δ [ppm] 7.25 (t, *J* = 7.5 Hz, 1H), 7.23 (s, 1H), 7.19 (d, *J* = 7.6 Hz, 1H), 7.16–7.13 (m, 2H), 7.10 (m, 2H), 6.80 (d, *J* = 8.2 Hz, 1H), 6.79 (d, *J* = 2.0 Hz, 1H), 6.73 (dd, *J* = 8.2, 2.0 Hz, 1H), 4.17 (s, 4H), 3.45 (s, 2H), 2.80 (m, 5H), 2.36 (s_broad_, 4H), 2.03 (s, 3H); ^**13**^**C NMR** (151 MHz, CDCl_3_): δ [ppm] 143.1, 142.8, 142.6, 142.3, 137.8, 135.9, 133.1, 130.3, 129.0, 128.8, 128.1, 128.0, 127.7, 125.3, 122.6, 118.2, 116.9, 64.5, 64.4, 63.6, 54.1, 45.8, 18.9; **IR (ATR)** [cm^–1^]: 3319, 2934, 1507, 1279, 1069; **LC–MS (DAD/ESI)**: *t*_R_ = 5.11 min, calcd for C_26_H_28_N_2_O_2_ [*m*/*z*] [M + H]^+^ 401.22; found, [M + H]^+^ 401.34; **HRMS (ESI)**: calcd for C_26_H_28_N_2_O_2_ [*m*/*z*] [M + H]^+^ 401.2223; found, [M + H]^+^ 401.2222.

#### 1-((3′-(Benzo-1,4-dioxan-6-yl)-3-methoxy-2′-methyl-[1,1′-biphenyl]-4-yl)methyl)piperazine (**6d**)

Method B. (3′-(Benzo-1,4-dioxan-6-yl)-3-methoxy-2′-methyl-[1,1′-biphenyl]-4-yl)methanol (**4i**) (0.20 g, 0.5 mmol, 1.0 equiv), thionyl chloride (0.19 mL, 2.7 mmol, 5.0 equiv), 1-Boc-piperazine (0.51 g, 2.7 mmol, 5.0 equiv). Product **6d** was obtained as a light-yellow oil with 19.9% (0.047 g) yield.

**R**_**f**_ = 0.08 (SiO_2_, MeOH); ^**1**^**H NMR** (600 MHz, DMSO-*d*_6_): δ [ppm] 7.35 (d, *J* = 7.6 Hz, 1H), 7.27 (t, *J* = 7.5 Hz, 1H), 7.19 (d, *J* = 6.6 Hz, 1H), 7.16 (dd, *J* = 7.5, 1.2 Hz, 1H), 6.95–6.89 (m, 3H), 6.88 (d, *J* = 2.1 Hz, 1H), 6.82 (dd, *J* = 8.2, 2.1 Hz, 1H), 4.28 (s, 4H), 3.80 (s, 3H), 3.45 (s, 2H), 3.17 (s, 1H), 2.74–2.60 (s_broad_, 4H), 2.40–2.27 (s_broad_, 4H), 2.09 (s, 3H); ^**13**^**C NMR** (151 MHz, DMSO-*d*_6_): δ [ppm] 157.4, 143.4, 143.0, 142.3, 142.0, 135.4, 132.9, 129.9, 129.1, 129.0, 125.9, 125.0, 122.6, 121.3, 118.2, 117.2, 112.3, 64.6, 56.6, 55.9, 54.9, 46.2, 19.2; **IR (ATR)** [cm^–1^]: 3322, 2934, 2829, 1577, 1507, 1463, 1399, 1301, 1280, 1227, 1068, 872, 792, 742; **LC–MS (DAD/ESI)**: *t*_R_ = 4.72 min, calcd for C_27_H_30_N_2_O_3_ [*m*/*z*] [M + H]^+^ 431.34; found, [M + H]^+^ 431.23; **HRMS (ESI)**: calcd for C_27_H_30_N_2_O_3_ (*m*/*z*) [M + H]^+^ 431.2329; found, [M + H]^+^ 431.2329.

#### 3-(((3′-(Benzo-1,4-dioxan-6-yl)-2′-methyl-4-(piperazin-1-ylmethyl)-[1,1′-biphenyl]-3-yl)oxy)methyl)benzonitrile (**6e**)

Method B. 3-(((3′-(Benzo-1,4-dioxan-6-yl)-4-(hydroxymethyl)-2′-methyl-[1,1′-biphenyl]-3-yl)oxy)methyl)benzonitrile (**4j**) (0.24 g, 0.5 mmol, 1.0 equiv), thionyl chloride (0.14 mL, 2.0 mmol, 5.0 equiv), 1-Boc-piperazine (0.37 g, 2.0 mmol, 5.0 equiv). Product **6e** was obtained as a colorless solid with 38.4% (0.104 g) yield.

**R**_**f**_ = 0.12 (SiO_2_, methanol^**1**^**H NMR** (600 MHz, DMSO-*d*_6_): δ [ppm] 7.94 (s, 1H), 7.84 (d, *J* = 7.9 Hz, 1H), 7.79 (d, *J* = 7.7 Hz, 1H), 7.62 (t, *J* = 7.8 Hz, 1H), 7.35 (d, *J* = 7.6 Hz, 1H), 7.26 (t, *J* = 7.3 Hz, 1H), 7.16 (d, *J* = 7.7 Hz, 2H), 7.02 (d, *J* = 1.4 Hz, 1H), 6.94–6.90 (m, 2H), 6.84 (d, *J* = 2.1 Hz, 1H), 6.81 (dd, *J* = 8.2, 2.1 Hz, 1H), 5.25 (s, 2H), 4.28 (s, 4H), 3.51 (s, 2H), 2.73–2.67 (m, 4H), 2.39–2.32 (m, 4H), 2.00 (s, 3H); ^**13**^**C NMR** (151 MHz, DMSO-*d*_6_): δ [ppm] 155.6, 143.0, 142.5, 142.2., 141.8, 141.7, 139.3, 134.9, 132.4, 132.0, 131.5, 130.6, 130.2, 129.7, 128.7, 128.4, 125.5, 125.5, 122.1, 121.3, 118.7, 117.7, 116.8, 113.6, 111.4, 68.0, 64.1, 56.7, 54.5, 45.7, 18.6); **IR (ATR)** [cm^–1^]: 2932, 2875, 2814, 2230, 1576, 1506, 1461, 1402, 1301, 1244, 1225, 1068, 1001, 792; **LC–MS (DAD/ESI)**: *t*_R_ = 5.93 min, calcd for C_34_H_33_N_3_O_3_ (*m*/*z*) [M + H]^+^ 532.26; found, [M + H]^+^ 532.41; **HRMS (ESI)**: calcd for C_34_H_33_N_3_O_3_ (*m*/*z*) [M + H]^+^ 532.2595; found, [M + H]^+^ 532.2594.

#### *N*-(2-(((3′-(Benzo-1,4-dioxan-6-yl)-2′-chloro-3-methoxy-[1,1′-biphenyl]-4-yl)methyl)amino)ethyl)acetamide (**7a**)

Method C. (3′-(Benzo-1,4-dioxan-6-yl)-2′-chloro-3-methoxy-[1,1′-biphenyl]-4-yl)methanol (**4k**) (0.15 g, 0.39 mmol, 1.0 equiv), SOCl_2_ (0.1 mL, 1.95 mmol, 5.0 equiv) *N*-(2-aminoethyl)acetamide (0.19 mL, 1.95 mmol, 5.0 equiv). Product **7a** was obtained as a brownish oil with 28.0% (0.051 g) yield.

**R**_**f**_ = 0.26 (SiO_2_, MeOH); ^**1**^**H NMR** (600 MHz, DMSO-*d*_6_): δ [ppm] 7.33 (m, 4H), 7.00–6.98 (m, 3H), 6.95–6.91 (m, 2H), 6.33 (s, 1H), 4.29 (s, 4H), 3.86 (s, 3H), 3.81 (s, 2H), 3.36 (m, 2H), 2.78 (t, *J* = 5.8 Hz, 2H), 2.00 (s_broad_, 1H), 1.98 (s, 3H); ^**13**^**C NMR** (151 MHz, DMSO-*d*_6_): δ [ppm] 170.3, 157.1, 143.2, 143.0, 141.4, 141.1, 140.5, 133.3, 130.9, 130.5, 130.1, 129.5, 127.2, 126.4, 122.8, 121.6, 118.6, 116.9, 112.0, 64.5, 64.4, 55.4, 48.7, 47.9, 39.1, 23.3; **IR (ATR)** [cm^–1^]: 3294, 2934, 1652, 1507, 1457, 1302, 1068, 732; **LC–MS (DAD/ESI)**: *t*_R_ = 5.43 min, calcd for C_26_H_27_ClN_2_O_4_ [*m*/*z*] [M + H]^+^ 467.17; found, [M + H]^+^ 467.28; **HRMS (ESI)**: calcd for C_26_H_27_ClN_2_O_4_ [*m*/*z*] [M + H]^+^ 467.1732; found, [M + H]^+^ 467.1732.

#### *N*-(2-(((3′-(Benzo-1,4-dioxan-6-yl)-2′-chloro-3-ethoxy-[1,1′-biphenyl]-4-yl)methyl)amino)ethyl)acetamide (**7b**)

Method D. (3′-(Benzo-1,4-dioxan-6-yl)-2′-chloro-3-ethoxy-[1,1′-biphenyl]-4-yl)methanol (**4l**0.15 g, 0.27 mmol, 1.0 equiv), SOCl_2_ (0.14 mL, 1.95 mmol, 5.0 equiv), *N*-(2-aminoethyl)acetamide (0.080 g, 0.78 mmol, 2.0 equiv), DIPEA (0.094 mL, 0.54 mmol, 2.0 equiv). Product **7b** was obtained as a brownish oil with 48.7% (0.091 g) yield.

**R**_**f**_ = 0.29 (SiO_2_, MeOH); ^**1**^**H NMR** (600 MHz, CDCl_3_): δ [ppm] 7.25–7.10 (m, 4H), 6.92–6.89 (m, 3H), 6.87–6.84 (m, 2H), 6.14 (s_broad_, 1H), 4.22 (s, 4H), 4.02 (q, *J* = 7.0 Hz, 2H), 3.75 (s, 2H), 3.29 (m, 2H), 2.70 (m, 2H), 1.90 (s, 3H), 1.35 (t, *J* = 7.0 Hz, 3H); ^**13**^**C NMR** (151 MHz, CDCl_3_): δ [ppm] 170.2, 156.4, 143.2, 143.0, 141.4, 141.1, 140.4, 133.3, 131.0, 130.5, 130.1, 129.5, 126.9, 126.4, 122.8, 121.5, 118.6, 116.9, 112.9, 64.5, 64.4, 63.6, 48.8, 47.8, 39.1, 23.4, 15.0; **IR (ATR)** [cm^–1^]: 3293, 3066, 2979, 2878, 1652, 1507, 1456, 1303, 1250, 1068; **LC–MS (DAD/ESI)**: *t*_R_ = 5.69 min, calcd for C_27_H_29_ClN_2_O_4_ [*m*/*z*] [M + H]^+^ 481.18; found, [M + H]^+^ 481.30; **HRMS (ESI)**: calcd for C_27_H_29_ClN_2_O_4_ [*m*/*z*] [M + H]^+^ 481.1889; found, [M + H]^+^ 481.1889.

#### *N*-(2-(((3′-(Benzo-1,4-dioxan-6-yl)-2′-chloro-3-isopropoxy-[1,1′-biphenyl]-4-yl)methyl)amino)ethyl)acetamide (**7c**)

Method D. (3′-(Benzo-1,4-dioxan-6-yl)-2′-chloro-3-isopropoxy-[1,1′-biphenyl]-4-yl)methanol (**4m**) (0.21 g, 0.53 mmol, 1.0 equiv), SOCl_2_ (0.12 mL, 1.59 mmol, 3.0 equiv), *N*-(2-aminoethyl)acetamide (0.21 mL, 2.24 mmol, 4.0 equiv), DIPEA (0.20 mL, 1.12 mmol, 2.0 equiv). Product **7c** was obtained as a colorless oil with 36.6% (0.096 g) yield.

**R**_**f**_ = 0.30 (SiO_2_, ethyl acetate/methanol 1:1;^**1**^**H NMR** (600 MHz, CDCl_3_): δ [ppm] 7.34–7.26 (m, 4H), 7.01–6.98 (m, 2H), 6.97–6.91 (m, 3H), 6.15 (s_broad_, 1H), 4.65–4.58 (m, 1H), 4.31 (s, 4H), 3.81 (s, 2H), 3.37 (q, *J* = 5.8 Hz, 2H), 2.78 (t, *J* = 5.8 Hz, 2H), 1.99 (s, 3H), 1.36 (d, *J* = 6.0 Hz, 6H); ^**13**^**C NMR** (151 MHz, CDCl_3_): δ [ppm] 170.3, 155.5, 143.3, 143.1, 141.6, 141.2, 140.5, 139.5, 131.5, 130.6, 130.3, 129.9, 128.0, 126.5, 122.9, 121.4, 118.7, 117.0, 114.5, 70.2, 64.6, 64.6, 49.1, 47.8, 39.1, 23.5, 22.4); **IR (ATR)** [cm^–1^]: 3295, 3057, 2976, 2931, 2874, 1652, 1507, 1456, 1302, 1280, 1249, 1226, 1113, 1069, 796, 734; **LC–MS (DAD/ESI)**: *t*_R_ = 5.86 min, calcd for C_28_H_31_ClN_2_O_4_ (*m*/*z*) [M + H]^+^ 495.32; found, [M + H]^+^ 495.20; **HRMS (ESI)**: calcd for C_28_H_31_ClN_2_O_4_ (*m*/*z*) [M + H]^+^ 495.2045; found, [M + H]^+^ 495.2045.

#### *N*-(2-(((3′-(Benzo-1,4-dioxan-6-yl)-2′-chloro-3-isopentyloxy-[1,1′-biphenyl]-4-yl)methyl)amino)ethyl)acetamide (**7d**)

Method D. (3′-(Benzo-1,4-dioxan-6-yl)-2′-chloro-3-isopentyloxy-[1,1′-biphenyl]-4-yl)methanol (**4n**) (0.15 g, 0.34 mmol, 1.0 equiv), SOCl_2_ (0.12 mL, 1.70 mmol, 5.0 equiv), *N*-(2-aminoethyl)acetamide (0.097 mL, 1.02 mmol, 3.0 equiv), DIPEA (0.12 mL, 0.68 mmol, 2.0 equiv). Product **7d** was obtained as a colorless oil 73.7% (0.131 g) yield.

**R**_**f**_ = 0.30 (SiO_2_, ethyl acetate/methanol 1:1); ^**1**^**H NMR** (600 MHz, CDCl_3_): δ [ppm] 7.34–7.26 (m, 4H), 7.00–6.97 (m, 3H), 6.95–6.91 (m, 2H), 6.23 (s, 1H), 4.31 (s, 1H), 4.04 (7, *J* = 6.6 Hz, 2H), 3.83 (s, 2H), 3.37 (q, *J* = 5.5 Hz, 2H), 2.79 (t, *J* = 5.9 Hz, 2H), 1.99 (s, 3H), 1.82 (sept, *J* = 6.7 Hz, 1H), 0.97 (d, *J* = 6.6 Hz, 6H); ^**13**^**C NMR** (151 MHz, CDCl_3_): δ [ppm] 170.4, 156.7, 143.3, 143.1, 141.5, 141.2, 140.7, 133.4, 131.1, 130.6, 130.2, 129.6, 126.9, 126.5, 122.9, 121.6, 118.7, 117.0,113.0, 66.6, 64.6, 64.5, 48.9, 47.9, 39.1, 38.2, 25.4, 23.5, 22.8; **IR (ATR)** [cm^–1^]: 3292, 3062, 2956, 2931, 2872, 1652, 1507, 1456, 1302, 1280, 1249, 1226, 1069, 796, 733; **LC–MS (DAD/ESI)**: *t*_R_ = 6.62 min, calcd for C_30_H_35_ClN_2_O_4_ (*m*/*z*) [M + H]^+^ 523.24; found, [M + H]^+^ 523.37; **HRMS (ESI)**: calcd for C_30_H_35_ClN_2_O_4_ (*m*/*z*) [M + H]^+^ 523.2358; found, [M + H]^+^ 523.2358.

#### *N*-(2-(((3′-(Benzo-1,4-dioxan-6-yl)-2′-chloro-3-(cyanomethoxy)-[1,1′-biphenyl]-4-yl)methyl)amino)ethyl)acetamide (**7e**)

Method D. 2-((3′-(Benzo-1,4-dioxan-6-yl)-2′-chloro-4-(hydroxymethyl)-[1,1′-biphenyl]-3-yl)oxy)acetonitrile (**4o**) (0.22 g, 0.54 mmol, 1.0 equiv), SOCl_2_ (0.12 mL, 1.62 mmol, 3.0 equiv), *N*-(2-aminoethyl)acetamide (0.21 mL, 2.24 mmol, 4.0 equiv), DIPEA (0.20 mL, 1.12 mmol, 2.0 equiv). Product **7e** was obtained as a colorless oil with 9.8% (0.026 g) yield.

**R**_**f**_ = 0.24 (SiO_2_, ethyl acetate/methanol 1:1); ^**1**^**H NMR** (600 MHz, CDCl_3_): δ [ppm] 7.37 (d, 7.7 Hz, 1H), 7.35–7.31 (m, 2H), 7.28 (dd, *J* = 6.7, 2.7 Hz, 1H), 7.15 (dd, *J* = 7.6, 1.5 Hz, 1H), 7.05 (d, *J* = 1.4 Hz, 1H), 6.99–6.98 (m, 1H), 6.95–6.91 (m, 2H), 6.09 (s_broad_, 1H), 4.86 (s, 2H), 4.30 (s, 4H), 3.85 (s, 2H), 3.38 (q, *J* = 5.5 Hz, 2H), 2.81 (t, *J* = 5.6 Hz, 2H), 1.99 (s, 3H); ^**13**^**C NMR** (151 MHz, CDCl_3_): δ [ppm] 170.3, 154.1, 143.3, 143.0, 141.2, 140.8, 140.5, 133.1, 130.9, 130.1, 128.5, 126.5, 124.3, 122.8, 118.5, 116.9, 115.1, 113.6, 64.5, 64.4, 53.9, 48.1, 48.0, 39.1, 23.4; **IR (ATR)** [cm^–1^]: 3292, 3059, 2932, 1652, 1582, 1508, 1457, 1319, 1303, 1281,1251, 1068, 798, 737; **LC–MS (DAD/ESI)**: *t*_R_ = 5.29 min, calcd for C_27_H_26_ClN_3_O_4_ (*m*/*z*) [M + H]^+^ 492.17; found, [M + H]^+^ 492.27; **HRMS (ESI)**: calcd for C_27_H_26_ClN_2_O_4_ (*m*/*z*) [M + H]^+^ 492.1685; found, [M + H]^+^ 492.1683.

#### *N*-(2-(((3′-(Benzo-1,4-dioxan-6-yl)-2′-chloro-3-(cyclobutylmethoxy)-[1,1′-biphenyl]-4-yl)methyl)amino)ethyl)acetamide (**7f**)

Method D. (3′-(Benzo-1,4-dioxan-6-yl)-2′-chloro-3-cyclobutylmethoxy-[1,1′-biphenyl]-4-yl)methanol (**4p**) (0.36 g, 0.83 mmol, 1.0 equiv), SOCl_2_ (0.30 mL, 4.16 mmol, 5.0 equiv), *N*-(2-aminoethyl)acetamide (0.23 mL, 2.49 mmol, 3.0 equiv), DIPEA (0.29 mL, 1.66 mmol, 2.0 equiv). Product **7f** was obtained as a colorless oil with 29.6% (0.128 g) yield.

**R**_**f**_ = 0.30 (SiO_2_, ethyl acetate/methanol 1:1); ^**1**^**H NMR** (600 MHz, DMSO-*d*_6_): δ [ppm] 7.82 (t, *J* = 5.5 Hz, 1H), 7.43 (t, *J* = 7.6 Hz, 1H), 7.38–7.36 (m, 2H), 7.35 (dd, *J* = 7.5, 1.8 Hz, 1H), 7.00 (d, *J* = 1.5 Hz, 1H), 6.97 (dd, *J* = 7.6, 1.6 Hz, 1H), 6.95–6.93 (m, 2H), 6.90 (dd, *J* = 8.3, 2.1 Hz, 1H), 4.29 (s, 4H), 3.99 (d, *J* = 6.3 Hz, 2H), 3.72 (s, 2H), 3.15 (q, *J* = 6.3 Hz, 2H), 2.78–2.71 (m, 1H), 2.58 (t, *J* = 6.5 Hz, 2H), 2.11–2.03 (m, 2H), 1.94–1.85 (m, 4H), 1.79)s, 3H); ^**13**^**C NMR** (151 MHz, DMSO-*d*_6_): δ [ppm] 169.14, 156.1, 143.1, 142.8, 141.1, 140.4, 139.0, 132.5, 130.4, 130.3, 129.9, 128.5, 128.5, 126.9, 122.4, 121.0, 118.0, 116.7, 112.7, 79.2, 71.3, 64.1, 64.1, 48.5, 47.3, 34.1, 24.2, 22.6, 18.1; **IR (ATR)** [cm^–1^]: 3292, 3062, 2975, 2934, 2867, 1652, 1507, 1456, 1302, 1280, 1249, 1226, 1068, 796, 747; **LC–MS (DAD/ESI)**: *t*_R_ = 6.37 min, calcd for C_30_H_33_ClN_2_O_4_ (*m*/*z*) [M + H]^+^ 521.22; found, [M + H]^+^ 521.38; **HRMS (ESI)**: calcd for C_30_H_33_ClN_2_O_4_ (*m*/*z*) [M + H]^+^ 521.2202; found, [M + H]^+^ 521.2202.

#### *N*-(2-(((3′-(Benzo-1,4-dioxan-6-yl)-2′-chloro-3-(cyclopentylmethoxy)-[1,1′-biphenyl]-4-yl)methyl)amino)ethyl)acetamide (**7g**)

Method D. (3′-(Benzo-1,4-dioxan-6-yl)-2′-chloro-3-cyclopentylmethoxy-[1,1′-biphenyl]-4-yl)methanol (**4q**) (0.21 g, 0.51 mmol, 1.0 equiv), SOCl_2_ (0.18 mL, 2.55 mmol, 5.0 equiv), *N*-(2-aminoethyl)acetamide (0.18 mL, 1.02 mmol, 3.0 equiv), DIPEA (0.15 mL, 1.53 mmol, 2.0 equiv). Product **7g** was obtained as a colorless oil with 70.9% (0.193 g) yield.

**R**_**f**_ = 0.30 (SiO_2_, ethyl acetate/methanol 1:1); ^**1**^**H NMR (600 MHz, CDCl**_**3**_**)**: δ [ppm] 7.33–7.27 (m, 4H), 7.00–6.95 (m, 3H), 6.95–6.91 (m, 2H), 6.18 (s, 1H), 4.31 (s, 4H), 3.89 (d, *J* = 6.9 Hz, 2H0, 3.84 (s, 2H), 3.37 (q, *J* = 5.5 Hz, 2H), 2.79 (t, *J* = 5.8 Hz, 2H), 2.44–2.35 (m, 1H), 1.88–1.81 (m, 2H), 1.68–1.56 (m, 4H), 1.39–1.33 (m, 2H); ^**13**^**C NMR (151 MHz, CDCl**_**3**_**)**: δ [ppm] 156.7, 143.2, 143.0, 141.4, 140.6, 133.3, 131.0, 130.5, 130.1, 129.6, 122.8, 121.4, 118.6, 116.9, 112.8, 72.2, 64.5, 64.4, 49.0, 47.8, 39.1, 39.0, 29.5, 25.5, 23.3; **IR (ATR)** [cm^–1^]: 3292, 3060, 2947, 2867, 1652, 1507, 1456, 1303, 1280, 1249, 1226, 1068, 796, 748; **LC–MS (DAD/ESI)**: *t*_R_ = 6.88 min, calcd for C_31_H_35_ClN_2_O_4_ (*m*/*z*) [M + H]^+^ 535.24; found, [M + H]^+^ 535.41; **HRMS (ESI)**: calcd for C_31_H_35_ClN_2_O_4_ (*m*/*z*) [M + H]^+^ 535.2358; found, [M + H]^+^ 535.2358.

#### *N*-(2-(((3′-(Benzo-1,4-dioxan-6-yl)-2′-chloro-3-((3′-cyano-[1,1′-biphenyl]-3-yl)methoxy)-[1,1′-biphenyl]-4-yl)methyl)amino)ethyl)acetamide (**7h**)

Method D. 3′-(((3′-(Benzo-1,4-dioxan-6-yl)-2′-chloro-4-(hydroxymethyl)-[1,1′-biphenyl]-3-yl)oxy)methyl)-[1,1′-biphenyl]-3-carbonitrile (**4r**) (0.15 g, 0.27 mmol, 1.0 equiv), SOCl_2_ (0.10 mL, 1.35 mmol, 5.0 equiv), *N*-(2-aminoethyl)acetamide (0.077 mL, 0.81 mmol, 3.0 equiv), DIPEA (0.094 mL, 0.54 mmol, 2.0 equiv). Product **7h** was obtained as a brownish oil with 78.5% (0.136 g) yield.

**R**_**f**_ = 0.26 (SiO_2_, DCM/methanol, 9:1 ^**1**^**H NMR** (600 MHz, CDCl_3_): δ [ppm] 7.77 (t, *J* = 1.5 Hz, 1H), 7.72 (dt, *J* = 7.9, 1.2 Hz, 1H), 7.54 (m, 2H), 7.47–7.39 (m, 4H), 7.29 (d, *J* = 7.7 Hz, 1H), 7.23–7.18 (m, 2H), 7.16–7.13 (m, 1H), 7.02 (d, *J* = 1.3 Hz, 1H), 6.93 (dd, *J* = 7.6, 1.4 Hz, 1H), 6.88 (m, 1H), 6.83–6.80 (m, 3H), 5.13 (s, 2H), 4.42 (s_broad_, 1H), 4.20 (s, 4H), 3.92 (s, 2H), 3.30 (m, 2H), 2.82 (t, *J* = 5.5 Hz, 2H), 1.80 (s, 3H); ^**13**^**C NMR** (151 MHz, CDCl_3_): δ [ppm] 170.9, 156.2, 143.3, 143.0, 142.0, 141.6, 141.2, 140.9, 139.3, 137.7, 133.1, 131.6, 131.0, 130.8, 130.8, 130.7, 130.4, 130.0, 129.8, 129.6, 127.4, 126.9, 126.5, 126.2, 124.1, 122.7, 122.3, 118.8, 118.5, 116.9, 113.7, 113.0, 70.1, 64.5, 64.4, 47.9, 47.9, 38.0, 23.1); **IR (ATR)** [cm^–1^]: 3293, 3062, 2932, 2229, 1652, 1507, 1456, 1303, 1249, 1068; **LC–MS (DAD/ESI)**: *t*_R_ = 6.68 min, calcd for C_39_H_34_ClN_3_O_4_ (*m*/*z*) [M + H]^+^ 644.23; found, [M + H]^+^ 644.42; **HRMS (ESI)**: calcd for C_39_H_34_ClN_3_O_4_ (*m*/*z*) [M + H]^+^ 644.2311; found, [M + H]^+^ 644.2311.

#### 1-((3′-(Benzo-1,4-dioxan-6-yl)-2′-chloro-3-methoxy-[1,1′-biphenyl]-4-yl)methyl)piperazine (**8a**)

Method B. (3′-(Benzo-1,4-dioxan-6-yl)-2′-chloro-3-methoxy-[1,1′-biphenyl]-4-yl)methanol (**4k**) (0.15 g, 0.39 mmol, 1.0 equiv), SOCl_2_ (0.14 mL, 1.95 mmol, 5.0 equiv), 1-Boc-piperazine (0.36 g, 1.95 mmol, 5.0 equiv). Product **8a** was obtained as a colorless solid with 33.3% (0.058 g) yield.

**R**_**f**_ = 0.10 (SiO_2_, MeOH); ^**1**^**H NMR** (600 MHz, DMSO-*d*_6_): δ [ppm] 7.43 (m, 1H), 7.38 (m, 2H), 7.35 (dd, *J* = 7.5, 1.5 Hz, 1H), 7.02 (s, 1H), 6.99 (d, *J* = 7.6 Hz, 1H), 6.95–6.93 (m, 2H), 6.90 (dd, *J* = 8.3, 1.8 Hz, 1H), 4.29 (s, 4H), 3.80 (s, 3H), 3.46 (s, 2H), 2.70 (t, *J* = 4.3 Hz, 4H), 2.35 (m, 5H); ^**13**^**C NMR** (151 MHz, DMSO-*d*_6_): δ [ppm] 157.3, 143.6, 143.3, 141.5, 140.9, 139.6, 133.0, 130.9, 130.8, 130.4, 129.8, 127.4, 125.9, 122.9, 121.6, 118.5, 117.2, 112.5, 64.6, 64.6, 56.5, 55.9, 54.9, 46.2; **IR (ATR)** [cm^–1^]: 3306, 2931, 1507, 1457, 796; **LC–MS (DAD/ESI)**: *t*_R_ = 4.71 min, calcd for C_26_H_27_ClN_2_O_3_ [*m*/*z*] [M + H]^+^ 451.18; found, [M + H]^+^ 451.32; **HRMS (ESI)**: calcd for C_26_H_27_ClN_2_O_3_ [M + H]^+^ 451.1783; found, [M + H]^+^ 451.1783.

#### 4-((3′-(Benzo-1,4-dioxan-6-yl)-2′-chloro-3-methoxy-[1,1′-biphenyl]-4-yl)methyl)piperazine-1-carboxamide (**8b**)

Method C. (3′-(Benzo-1,4-dioxan-6-yl)-2′-chloro-3-methoxy-[1,1′-biphenyl]-4-yl)methanol (**4k**) (0.15 g, 0.39 mmol, 1.0 equiv), SOCl_2_ (0.14 mL, 1.95 mmol, 5.0 equiv), piperazine-1-carboxamide hydrochloride (0.13 g, 0.78 mmol, 2.0 equiv), triethylamine (0.11 mL, 0.78 mmol, 2.00 equiv). Product **8b** was obtained as a brownish solid with 36.3% (0.070 g) yield.

**R**_**f**_ = 0.38 (SiO_2_, DCM/MeOH, 9:1); ^**1**^**H NMR** (600 MHz, CDCl_3_): δ [ppm] 7.31 (d, *J* = 7.7 Hz, 1H), 7.24–7.17 (m, 3H), 6.93 (dd, *J* = 7.7, 1.5 Hz, 1H), 6.91 (d, *J* = 1.9 Hz, 1H), 6.89 (d, *J* = 1.5 Hz, 1H), 6.86–6.83 (m, 2H), 4.71 (s, 2H), 4.20 (s, 4H), 3.74 (s, 3H), 3.54 (s, 2H), 3.34 (t, *J* = 5.0 Hz, 4H), 2.45 (t, *J* = 5.1 Hz, 4H); ^**13**^**C NMR** (151 MHz, CDCl_3_): δ [ppm] 158.3, 157.2, 143.2, 143.0, 141.5, 141.1, 140.2, 133.4, 130.9, 130.5, 130.2, 130.0, 126.4, 125.0, 122.8, 121.6, 118.6, 116.9, 112.1, 64.5, 64.4, 56.0, 55.6, 52.8, 44.1; **IR (ATR)** [cm^–1^]: 3352, 2937, 1586, 1507, 1459, 1301, 1228, 1068; **LC–MS (DAD/ESI)**: *t*_R_ = 5.22 min, calcd C_27_H_28_ClN_3_O_4_ [*m*/*z*] [M + H]^+^ 494.18; found, [M + H]^+^ 494.33; **HRMS (ESI)**: calcd for C_27_H_28_ClN_3_O_4_ [*m*/*z*] [M + H]^+^ 494.1841; found, [M + H]^+^ 494.1841.

#### 1-(4-((3′-(Benzo-1,4-dioxan-6-yl)-2′-chloro-3-methoxy-[1,1′-biphenyl]-4-yl)methyl)piperazin-1-yl)ethan-1-one (**8c**)

Method D. (3′-(Benzo-1,4-dioxan-6-yl)-2′-chloro-3-methoxy-[1,1′-biphenyl]-4-yl)methanol (**4k**) (0.15 g, 0.39 mmol, 1.0 equiv), SOCl_2_ (0.14 mL, 1.95 mmol, 5.0 equiv), 1-acetylpiperazine (0.10 g, 0.78 mmol, 2.0 equiv), DIPEA (0.14 mL, 0.78 mmol, 2.0 equiv). Product **8c** was obtained as a brownish oil with 76.3% (0.147 g) yield.

**R**_**f**_ = 0.46 (SiO_2_, DCM/MeOH, 9:1); ^**1**^**H NMR** (600 MHz, CDCl_3_): δ [ppm] 7.30 (d, *J* = 7.7 Hz, 1H), 7.23–7.17 (m, 3H), 6.92 (dd, *J* = 7.6, 1.5 Hz, 1H), 6.91 (d, *J* = 1.9 Hz, 1H), 6.88 (d, *J* = 1.5 Hz, 1H), 6.85–6.82 (m, 2H), 4.18 (s, 4H), 3.74 (s, 3H), 3.56 (t, *J* = 4.9 Hz, 2H), 3.54 (s, 2H), 3.39 (t, *J* = 4.8 Hz, 2H), 2.44 (m, 4H), 1.99 (s, 3H); ^**13**^**C NMR** (151 MHz, CDCl_3_): δ [ppm] 169.0, 157.3, 143.2, 143.0, 141.5, 141.1, 140.3, 133.3, 130.9, 130.5, 130.2, 130.1, 126.4, 124.7, 122.8, 121.6, 118.6, 116.9, 112.1, 64.4, 64.4, 55.8, 55.5, 53.1, 52.7, 46.4, 41.5, 21.4; **IR (ATR)** [cm^–1^]: 3436, 2933, 1644, 1507, 1458, 1247, 1228; **LC–MS (DAD/ESI)**: *t*_R_ = 5.38 min, calcd for C_28_H_29_ClN_2_O_4_ [*m*/*z*] [M + H]^+^ 493.19; found, [M + H]^+^ 493.33; **HRMS (ESI)**: calcd for C_28_H_29_ClN_2_O_4_ [M + H]^+^ 493.1888; found, [M + H]^+^ 493.1889.

#### 2-Amino-1-(4-((3′-(benzo-1,4-dioxan-6-yl)-2′-chloro-3-methoxy-[1,1′-biphenyl]-4-yl)methyl)piperazin-1-yl)ethan-1-one (**8d**)

Method E. (3′-(Benzo-1,4-dioxan-6-yl)-2′-chloro-3-methoxy-[1,1′-biphenyl]-4-yl)methanol (**4k**) (0.15 g, 0.39 mmol, 1.0 equiv), SOCl_2_ (0.14 mL, 1.95 mmol, 5.0 equiv), *tert*-butyl(2-oxo-2-(piperazin-1-yl)ethyl)carbamate (0.19 g, 0.78 mmol, 2.0 equiv), DIPEA (0.14 mL, 0.78 mmol, 2.0 equiv). Product **8d** was obtained as a brownish solid with 57.3% (0.114 g) yield.

**R**_**f**_ = 0.26 (SiO_2_, methanol/7 M NH_3_ in methanol, 20:1); ^**1**^**H NMR** (600 MHz, CDCl_3_): δ [ppm] 7.30 (d, *J* = 7.7 Hz, 1H), 7.25–7.19 (m, 3H), 6.94–6.92 (m, 2H), 6.89 (m, 1H), 6.87–6.83 (m, 2H), 4.22 (s, 4H), 3.75 (s, 3H), 3.59 (m, 2H), 3.54 (s, 2H), 3.48 (s_broad_, 2H), 3,34 (m, 2H), 3.05 (s_broad_, 2H), 2.45 (m, 4H); ^**13**^**C NMR** (151 MHz, CDCl_3_): δ [ppm] 170.2, 157.2, 143.2, 143.0, 141.5, 141.1, 140.3, 133.3, 130.9, 130.5, 130.2, 130.0, 126.3, 124.8, 122.8, 121.6, 118.6, 116.9, 112.1, 64.5, 64.4, 55.9, 55.6, 52.9, 52.8, 44.3, 42.7, 42.2; **IR (ATR)** [cm^–1^]: 3371, 2935, 1651, 1507, 1459, 1246, 1229, 732; **LC–MS (DAD/ESI)**: *t*_R_ = 4.50 min, calcd C_28_H_30_ClN_3_O_4_ [M + H]^+^ 508.20; found, [M + H]^+^ 508.35; **HRMS (ESI**^+^**)**: calcd for C_28_H_30_ClN_3_O_4_ [M + H]^+^ 508.1998; found, [M + H]^+^ 508.1998.

#### 1-((3′-(Benzo-1,4-dioxan-6-yl)-2′-chloro-3-methoxy-[1,1′-biphenyl]-4-yl)methyl)-4-(methylsulfonyl)piperazine (**8e**)

Method D. (3′-(Benzo-1,4-dioxan-6-yl)-2′-chloro-3-methoxy-[1,1′-biphenyl]-4-yl)methanol (**4k**) (0.15 g, 0.39 mmol, 1.0 equiv), SOCl_2_ (0.14 mL, 1.95 mmol, 5.0 equiv), 1-(methylsulfonyl)piperazine (0.13 g, 0.78 mmol, 2.0 equiv), DIPEA (0.14 mL, 0.78 mmol, 2.0 equiv). Product **8e** was obtained as a brownish solid with 88.9% (0.183 g) yield.

**R**_**f**_ = 0.27 (SiO_2_, EtOAc); ^**1**^**H NMR** (600 MHz, CDCl_3_): δ [ppm] 7.39 (d, *J* = 7.7 Hz, 1H), 7.36–7.31 (m, 3H), 7.03 (dd, *J* = 7.6, 1.5 Hz, 1H), 7.02 (d, *J* = 1.8 Hz, 1H), 7.00 (d, *J* = 1.4 Hz, 1H), 6.97–6.94 (m, 2H), 4.32 (s, 4H), 3.86 (s, 3H), 3.68 (s, 2H), 3.30 (m, 4H), 2.80 (s, 3H), 2.69 (t, *J* = 4.2 Hz, 4H); ^**13**^**C NMR** (151 MHz, CDCl_3_): δ [ppm] 157.3, 143.2, 143.0, 141.4, 141.1, 140.4, 133.3, 130.9, 130.5, 130.1, 130.0, 126.4, 124.7, 122.8, 121.6, 118.6, 116.9, 112.2, 64.5, 64.4, 55.7, 55.6, 52.3, 46.0, 34.1; **IR (ATR)** [cm^–1^]: 2929, 1507, 1323, 1160; **LC–MS (DAD/ESI)**: *t*_R_ = 5.81 min, calcd for C_27_H_29_ClN_2_O_5_S [*m*/*z*] [M + H]^+^ 529.16; found, [M + H]^+^ 529.29; **HRMS (ESI)**: calcd for C_27_H_29_ClN_2_O_5_S [M + Na]^+^ 551.1378; found, [M + Na]^+^ 551.1378.

#### 2-Amino-*N*-(2-(((3′-(benzo-1,4-dioxan-6-yl)-2′-chloro-3-methoxy-[1,1′-biphenyl]-4-yl)methyl)amino)ethyl)acetamide (**8f**)

Method E. (3′-(Benzo-1,4-dioxan-6-yl)-2′-chloro-3-methoxy-[1,1′-biphenyl]-4-yl)methanol (**4k**) (0.15 g, 0.39 mmol, 1.0 equiv), SOCl_2_ (0.14 mL, 1.95 mmol, 5.0 equiv), *tert*-butyl (2-((2-aminoethyl)amino)-2-oxoethyl)carbamate (0.17 g, 0.78 mmol, 2.0 equiv), DIPEA (0.14 mL, 0.78 mmol, 2.0 equiv). Product **8f** was obtained as a brownish solid with 43.3% (0.081 g) yield.

**R**_**f**_ = 0.29 (SiO_2_, methanol/7M NH_3_ in methanol, 20:1); **IR (ATR)** [cm^–1^]: 3306, 2934, 1650, 1581, 1507, 1459, 1068; ^**1**^**H NMR** (600 MHz, CDCl_3_): δ [ppm] 7.47 (s, 1H), 7.22 (m, 4H), 6.91–6.85 (m, 5H), 4.22 (s, 4H), 3.77 (s, 5H), 3.38–3.38 (m, 4H), 2.74 (s, 2H); ^**13**^**C NMR** (151 MHz, CDCl_3_): δ [ppm] 172.8, 157.1, 143.2, 143.0, 141.4, 141.1, 140.4, 133.3, 130.9, 130.5, 130.1, 129.5, 126.4, 122.8, 121.6, 118.6, 116.8, 112.0, 64.5, 64.4, 55.4, 48.5, 48.0, 44.9, 38.6; **LC–MS (DAD/ESI)**: *t*_R_ = 4.25 min, calcd C_26_H_28_ClN_3_O_4_ [*m*/*z*] [M + H]^+^ 482.18; found, [M + H]^+^ 482.30; **HRMS (ESI)**: calcd for C_26_H_28_ClN_3_O_4_ [*m*/*z*] [M + H]^+^ 482.1841; found, [M + H]^+^ 482.1840.

#### *N*-(2-(((3′-(Benzo-1,4-dioxan-6-yl)-2′-chloro-3-methoxy-[1,1′-biphenyl]-4-yl)methyl)amino)ethyl)methanesulfonamide (**8g**)

Method C. (3′-(Benzo-1,4-dioxan-6-yl)-2′-chloro-3-methoxy-[1,1′-biphenyl]-4-yl)methanol (**4k**) (0.15 g, 0.39 mmol, 1.0 equiv), SOCl_2_ (0.14 mL, 1.95 mmol, 5.0 equiv), *N*-(2-aminoethyl)methanesulfonamide (0.27 g, 1.95 mmol, 5.0 equiv). Product **8g** was obtained as a brownish oil with 36.2% (0.071 g) yield.

**R**_**f**_ = 0.28 (SiO_2_, DCM/methanol, 1:1); ^**1**^**H NMR** (600 MHz, CDCl_3_): δ [ppm] 7.26–7.21 (m, 3H), 7.19 (dd, *J* = 6.6, 2.3 Hz, 1H), 6.94–6.91 (m, 3H), 6.87–6.84 (m, 2H), 4.85 (s_broad_, 2H), 4.22 (s, 4H), 3.83 (s, 2H), 3.81 (s, 3H), 3.22 (t, *J* = 5.7 Hz, 2H), 2.87 (s, 3H), 2.84 (t, *J* = 5.6 Hz, 2H); ^**13**^**C NMR** (151 MHz, CDCl_3_): δ [ppm] 157.2, 143.2, 143.0, 141.4, 141.1, 141.1, 133.2, 130.9, 130.7, 130.1, 126.4, 124.5, 122.8, 121.8, 118.6, 116.9, 112.2, 64.5, 64.4, 55.6, 48.1, 47.6, 41.7, 40.0; **IR (ATR)** [cm^–1^]: 3320, 2932, 1507, 1457, 1318, 1303, 1151, 797; **LC–MS (DAD/ESI)**: *t*_R_ = 5.50 min, calcd C_25_H_27_ClN_2_O_5_S [*m*/*z*] [M + H]^+^ 503.14; found, [M + H]^+^ 503.30; **HRMS (ESI)**: calcd for C_25_H_27_ClN_2_O_5_S [*m*/*z*] [M + H]^+^ 503.1402; found, [M + H]^+^ 503.1402.

#### 1-(2-(((3′-(Benzo-1,4-dioxan-6-yl)-2′-chloro-3-methoxy-[1,1′-biphenyl]-4-yl)methyl)amino)ethyl)urea (**8h**)

Method D. (3′-(Benzo-1,4-dioxan-6-yl)-2′-chloro-3-methoxy-[1,1′-biphenyl]-4-yl)methanol (**4k**) (0.15 g, 0.39 mmol, 1.0 equiv), SOCl_2_ (0.142 mL, 1.95 mmol, 5.0 equiv), (2-aminoethyl)urea hydrochloride (0.11 g, 0.78 mmol, 2.0 equiv), triethylamine (0.054 mL, 0.39 mmol, 1.00 equiv), DIPEA (0.094 mL, 0.54 mmol, 2.0 equiv). Product **8h** was obtained as a brownish oil with 54.9% (0.100 g) yield.

**R**_**f**_ = 0.18 (SiO_2_, CHCl_3_/7M NH_3_ in methanol, 10:1); ^**1**^**H NMR** (600 MHz, CDCl_3_): δ [ppm] 7.33–7.27 (m, 3H), 7.24 (d, *J* = 7.5 Hz, 1H), 6.99–6.96 (m, 3H), 6.94–6.91 (m, 2H), 5.70 (s_broad_, 1H), 4.93 (s_broad_, 2H), 4.29 (s, 4H), 3.84 (s, 3H), 3.80 (s, 2H), 3.27 (m, 2H), 2.77 (t, *J* = 5.5 Hz, 2H), 2.19 (s_broad_, 1H); ^**13**^**C NMR** (151 MHz, CDCl_3_): δ [ppm] 159.7, 157.1, 143.2, 143.0, 141.4, 141.1, 140.6, 133.3, 130.9, 130.5, 130.1, 129.5, 126.9, 126.4, 122.8, 121.6, 118.6, 116.9, 112.0, 64.5, 64.4, 55.4, 48.9, 48.8, 40.3; **IR (ATR)** [cm^–1^]: 3335, 2933, 1658, 1582, 1507, 1457, 1303, 1168; **LC–MS (DAD/ESI)**: *t*_R_ = 5.23 min, calcd C_25_H_26_ClN_3_O_4_ [*m*/*z*] [M + H]^+^ 468.17; found, [M + H]^+^ 468.27; **HRMS (ESI)**: calcd for C_25_H_26_ClN_3_O_4_ [*m*/*z*] [M + H]^+^ 468.1685; found, [M + H]^+^ 468.1687.

#### 2-(((3′-(Benzo-1,4-dioxan-6-yl)-2′-chloro-3-methoxy-[1,1′-biphenyl]-4-yl)methyl)amino)propane-1,3-diol (**8i**)

Method D. (3′-(Benzo-1,4-dioxan-6-yl)-2′-chloro-3-methoxy-[1,1′-biphenyl]-4-yl)methanol (**4k**) (0.15 g, 0.39 mmol, 1.0 equiv), SOCl_2_ (0.14 mL, 1.95 mmol, 5.0 equiv), serinol (0.071 g, 0.78 mmol, 2.0 equiv), DIPEA (0.094 mL, 0.54 mmol, 2.0 equiv). Product **8i** was obtained as a brownish oil with 68.0% (0.121 g) yield.

**R**_**f**_ = 0.32 (SiO_2_, CHCl_3_/methanol, 1:2); ^**1**^**H NMR** (600 MHz, CDCl_3_): δ [ppm] 7.26 (d, *J* = 7.6 Hz, 1H), 7.23–7.21 (m, 2H), 7.18 (m, 1H), 6.93–6.90 (m, 3H), 6.86–6.83 (m, 2H), 4.84 (s_broad_, 2H), 4.22 (s, 4H), 3.98 (s, 2H), 3.80 (s, 3H), 3.72 (m, 2H), 3.61 (m, 2H), 2.86 (m, 1H), 1.84 (s, 1H); ^**13**^**C NMR** (151 MHz, CDCl_3_): δ [ppm] 157.2, 143.2, 143.0, 141.8, 141.1, 141.1, 133.2, 130.9, 130.7, 130.3, 130.1, 126.4, 123.3, 122.8, 122.0, 118.6, 116.9, 112.2, 77.1, 64.5, 64.4, 60.7, 59.6, 55.6, 46.1, 24.0; **IR (ATR)** [cm^–1^]: 3304, 2935, 2246, 1507, 1458, 1303, 1246, 1069, 732; **LC–MS (DAD/ESI)**: *t*_R_ = 5.27 min, calcd C_25_H_26_ClNO_5_ [*m*/*z*] [M + H]^+^ 456.16; found, [M + H]^+^ 456.24; **HRMS (ESI)**: calcd for C_25_H_26_ClNO_5_ [*m*/*z*] [M + H]^+^ 456.1572; found, [M + H]^+^ 456.1573.

#### 2-(((3′-(Benzo-1,4-dioxan-6-yl)-2′-chloro-3-methoxy-[1,1′-biphenyl]-4-yl)methyl)amino)-2-(hydroxymethyl)propane-1,3-diol (**8j**)

Method D. (3′-(Benzo-1,4-dioxan-6-yl)-2′-chloro-3-methoxy-[1,1′-biphenyl]-4-yl)methanol (**4k**) (0.15 g, 0.39 mmol, 1.0 equiv), SOCl_2_ (0.14 mL, 1.95 mmol, 5.0 equiv), tris(hydroxymethyl)aminomethane (0.094 g, 0.78 mmol, 2.0 equiv), DIPEA (0.094 mL, 0.54 mmol, 2.0 equiv). Product **8j** was obtained as a brownish oil with 77.7% (0.147 g) yield.

**R**_**f**_ = 0.26 (SiO_2_, CHCl_3_/methanol, 1:2); ^**1**^**H NMR** (600 MHz, CDCl_3_): δ [ppm] 7.23 (d, *J* = 7.8 Hz, 1H), 7.21–7.17 (m, 3H), 6.90 (m, 3H), 6.85–6.82 (m, 2H), 4.20 (s, 4H), 3.78 (s, 3H), 3.68 (s, 2H), 3.52 (s, 6H), 3.13 (s_broad_, 3H); ^**13**^**C NMR** (151 MHz, CDCl_3_): δ [ppm] 156.9, 143.2, 143.0, 141.3, 141.1, 140.9, 133.3, 130.9, 130.6, 130.1, 129.8, 127.1, 126.4, 122.8, 122.0, 118.6, 116.9, 112.2, 64.5, 64.4, 62.9, 60.4, 55.6, 40.9; **IR (ATR)** [cm^–1^]: 3343, 2935, 1507, 1457, 1068, 1042, 796; **LC–MS (DAD/ESI)**: *t*_R_ = 5.18 min, calcd C_26_H_28_ClNO_6_ [*m*/*z*] [M + H]^+^ 486.17, found, [M + H]^+^ 486.29; **HRMS (ESI)**: calcd for C_26_H_28_ClNO_6_ [*m*/*z*] [M + H]^+^ 486.1678; found, [M + H]^+^ 486.1.

## Supporting Information Available

Experimental procedures for substrate preparation (**S1**, **S2a–i**, and **3a–3i**); results of the HTRF assay; data collection and refinement statistics (molecular replacement) for the PD-L1 cocrystal structure with **8j**; results of the ICB assay; comparison of the terphenyl-based with biphenyl-based inhibitor structures in complex with PD-L1; and spectral data and purity of target compounds (PDF)
Molecular formula strings (CSV)

## Accession Codes

7NLD; authors will release the atomic coordinates and experimental data upon article publication.

## Abbreviations

DMSO: dimethyl sulfoxide
EC_50_: half-maximal effective concentration
FBS: fetal bovine serum
GuHCl: guanidinium chloride
HTRF: homogeneous time-resolved fluorescence
ICB: immune checkpoint blockade
IPTG: isopropyl β-d-1-thiogalactopyranoside
irAE: immune-related adverse events
Jurkat ECs: Jurkat effector cells
LB: Luria–Bertani broth Miller
mAbs: monoclonal antibodies
NFAT-RE: nuclear factor of activated T-cell response element
PBMCs: peripheral blood mononuclear cells
PD-1: programmed cell death protein 1
PD-L1: programmed cell death ligand 1 m-murine, h-human
SMI: small-molecule inhibitors
TCA: T-cell activation
TCR: T-cell receptor
TCRAct: TCR activator
TPSA: topological polar surface area
TR-FRET: time-resolved fluorescence energy transfer
