# Supplementary material for: Terphenyl-Based Small-Molecule Inhibitors of Programmed Cell Death-1/Programmed Death-Ligand 1 Protein–Protein Interaction
Source: J Med Chem. 2021 Jul 27;64(15):11614–36. doi: 10.1021/acs.jmedchem.1c00957 (PMC8365601; doi:10.1021/acs.jmedchem.1c00957)
Supplement: Supplementary file 1 — jm1c00957_si_001.pdf [file jm1c00957_si_001.pdf]

Supporting Information

Terphenyl-based Small-Molecule Inhibitors of Programmed Cell Death-1/Programmed Death-Ligand 1 Protein−Protein Interaction

Damian Muszak[1], Ewa Surmiak[1,*], Jacek Plewka[2], Katarzyna Magiera-Mularz[1], Justyna Kocik-Krol[1], Bogdan Musielak[1], Dominik Sala[1], Radoslaw Kitel[1], Malgorzata Stec[3], Kazimierz Weglarczyk[3], Maciej Siedlar[3], Alexander Dömling[4], Lukasz Skalniak[1], Tad A. Holak[1,*]

[1]Department of Organic Chemistry, Faculty of Chemistry, Jagiellonian University, Gronostajowa 2, 30-387 Krakow, Poland

[2]Malopolska Centre of Biotechnology, Jagiellonian University, Gronostajowa 7a, 30-387 Kraków, Poland

[3]Department of Clinical Immunology, Institute of Pediatrics, Jagiellonian University Medical College, Wielicka 265, 30-663 Krakow, Poland

[4]University of Groningen, Department of Drug Design, A. Deusinglaan 1, 9713 AV Groningen, The Netherlands

*corresponding author: ewa.surmiak@uj.edu.pl; tadholak@uj.edu.pl

CONTENT

1. PREPARATION OF PHENYLBORONIC ACID PINACOL ESTERS 2
2. SUPPRORTING FIGURES AND TABLES 13
Table S1. Structures and HTRF data for obtained compounds 13
Table S2. Data collection and refinement statistics (molecular replacement) 18
Figure S1.Results of the Immune Checkpoint Blockade (ICB) assay 19
Figure S2. Comparison of the terphenyl-based with biphenyl-based inhibitor structures 23
3. COPIES OF NMR SPECTRA 24
4. COPIES OF LCMS OF FINAL COMPOUNDS 104
6. REFERENCES 116

1. PREPARATION OF PHENYLBORONIC ACID PINACOL ESTERS

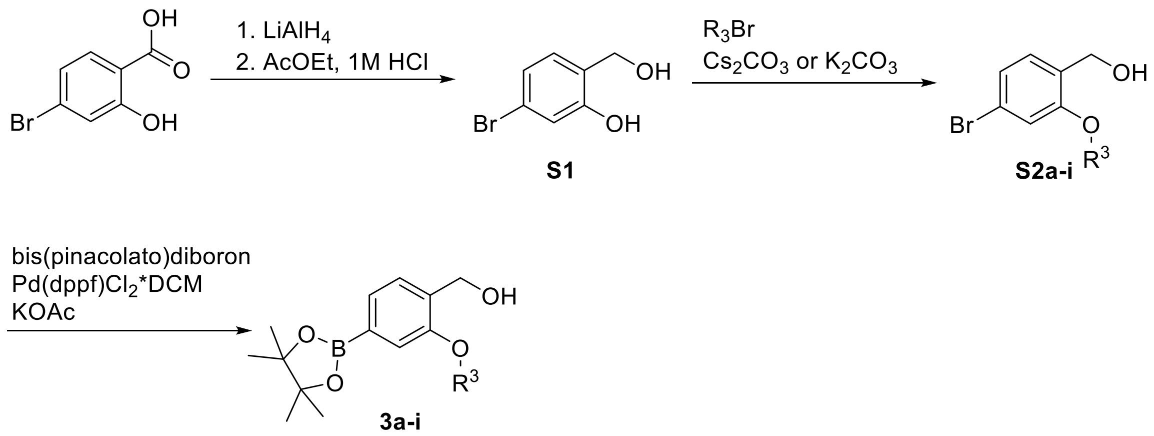

1.1. Preparation of 4-bromo-2-hydroxymethylbenzyl alcohol (S1):

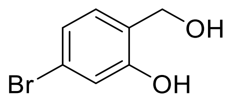

$LiAlH_4$ (2.28 g, 60.0. mmol, 4.0 equiv) in anhydrous diethyl ether was placed in a round-bottom flask and cooled down in ice bath. Then 4-bromo-2-hydroxybenzoic acid (3.26 g, 15.0 mmol, 1 equiv) dissolved in anhydrous diethyl ether was added dropwise to this mixture. The reaction was stirred at RT overnight, after this time it was quenched with ethyl acetate/water and then acidified with 1 M HCl, followed by extraction with ethyl acetate. Organic phases were combined, dried over anhydrous $MgSO_4$ and evaporated. The crude product was purified by flash chromatography (SiO_2, hexane/ethyl acetate 1:2) giving final product **S1** with 73.9% (2.25 g) yield. Compound **S1** was previously described in a patent application.[1]

R_f = 0.53 (SiO_2, hexane/ethyl acetate 1:2); **^{1}H NMR** (600 MHz, DMSO-d_6) δ [ppm]: 9.85 (s, 1H), 7.24 (d, J = 8.1 Hz, 1H), 6.97 (dd, J = 8.0, 2.0 Hz, 1H), 6.94 (d, J = 1.9 Hz, 1H), 4.44 (s, 2H); **^{13}C NMR** (151 MHz, DMSO-d_6) δ [ppm]: 155.7, 129.4, 128.8, 121.9, 119.8, 117.5, 58.2; **IR (ATR)** [cm^{-1}]: 3419, 3142, 2360, 1588, 1405, 1256; **LC-MS (DAD/ESI):** t_R = 4.11 min, calcd for C_7H_7Br (*m/z*): $[M-H]^-$ 200.96, found: $[M-H]^-$ 200.90.

1.2. General procedure for Williamson alkylation leading to alkoxy intermediates S2a-i:

5-Bromo-2-(hydroxymethyl)phenol (**S1**), appropriate alkyl halide, Cs_2CO_3 or K_2CO_3 and anhydrous DMF were placed in a round-bottom flask. The reaction mixture was heated at 85ºC for 16 h. When it was completed water was added and extraction with ethyl acetate follows. Organic phases were combined, dried over anhydrous $MgSO_4$ and evaporated. Crude products

were purified by flash chromatography (SiO_2, hexane/ethyl acetate) giving final products **S1a-i** with 91-43% yield.

(4-Bromo-2-methoxyphenyl)methanol (S2a)

Compound **S2a** was purchased from Sigma-Aldrich.

(4-Bromo-2-ethoxyphenyl)methanol (S2b)

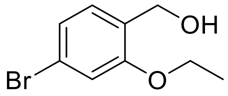

5-Bromo-2-(hydroxymethyl)phenol (**S1**) (0.50 g, 2.46 mmol, 1.0 equiv), bromoethane (0.82 ml, 2.71 mmol, 1.1 equiv), Cs_2CO_3 (1.20 g, 3.69 mmol, 1.5 equiv). Product **S2b** was obtained as colourless solid with 91.0% (0.52 g) yield. Compound **S2b** was previously characterized in patent application.[2]

R_f = 0.46 (SiO_2, hexane/ethyl acetate 1:1); **^{1}H NMR** (600 MHz, $CDCl_3$) δ [ppm]: 7.07 (d, *J* = 7.9 Hz, 1H), 6.99 (dd, *J* = 8.0, 1.7 Hz, 1H), 6.92 (d, *J* = 1.6 Hz, 1H), 4.56 (s, 2H), 4.00 (q, *J* = 7.0 Hz, 2H), 2.13 (s_{broad}, 1H), 1.37 (t, *J* = 7.0 Hz, 3H); **^{13}C NMR** (151 MHz, $CDCl_3$) δ [ppm]: 157.3, 129.7, 128.3, 123.5, 121.9, 114.7, 64.0, 61.6, 14.8; **IR (ATR)** [cm^{-1}]: 3486, 2985, 1414, 1351, 1242, 1145; **LC-MS (DAD/ESI):** t_R = 5.96 min, calcd for $C_9H_{11}BrO_2$ (*m/z*): $[M-H_2O+H]^+$ 213.00, found: $[M-H_2O+H]^+$ 212.99; **HRMS (ESI):** Calcd for $C_9H_{11}BrO_2$ (*m/z*): $[M+Na]^+$ 252.9835, found: $[M+Na]^+$ 252.9835

(4-Bromo-2-isopropoxyphenyl)methanol (S2c)

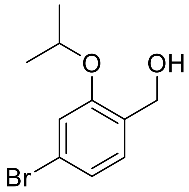

5-Bromo-2-(hydroxymethyl)phenol (**S1**) (0.69 g, 3.40 mmol, 1.0 equiv), 2-bromopropane (0.64 ml, 6.80 mmol, 2.0 equiv), Cs_2CO_3 (2.21 g, 6.80 mmol, 2.0 equiv). Product **S2c** was obtained as light yellow oil with 62.0% (0.52 g) yield. Compound **S2c** was previously described in literature.[3]

R_f = 0.24 (SiO_2, hexane/ethyl acetate 4:1); **^{1}H NMR** (600 MHz, $CDCl_3$) δ [ppm]: 7.14 (d, *J* = 8.0 Hz, 1H), 7.05 (dd, *J* = 8.0, 1.8 Hz, 1H), 7.01 (d, *J* = 1.7 Hz, 1H), 4.61 (s, 2H), 4.60-4.56 (m, 1H), 1.37 (d, *J* = 6.1 Hz, 6H); **^{13}C NMR** (151 MHz, $CDCl_3$) δ [ppm]: 156.5, 130.0, 129.2, 123.5, 12.0, 116.1, 70.9, 62.0, 22.2; **IR (ATR)** [cm^{-1}]: 3343, 2977, 2930, 1592, 1484, 1402, 1238, 1122, 1109,

1042, 960, 879; **LC-MS (DAD/ESI)**: t_r = 6.48 min, calcd for $C_{10}H_{13}BrO_2$ (*m/z*): $[M-H_2O+H]^+$ 227.01, found: $[M-H_2O+H]^+$ 227.02; **HRMS (ESI):** Calcd for $C_{10}H_{13}BrO_2$ (*m/z*): $[M+Na]^+$ 266.9991, found: $[M+Na]^+$ 266.9991

(4-Bromo-2-isopentyloxyphenyl)methanol (S2d)

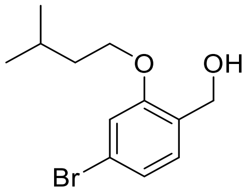

5-Bromo-2-(hydroxymethyl)phenol (**S1**) (0.43 g, 2.12 mmol, 1.0 equiv), 1-bromo3-methylbutane (0.51 ml, 4.24 mmol, 2.0 equiv), Cs_2CO_3 (1.38 g, 4.24 mmol, 2.0 equiv). Product **S2d** was obtained as yellow oil with 51.6% (0.30 g) yield.

R_f = 0.29 (SiO_2, hexane/ethyl acetate 4:1); **^{1}H NMR** (600 MHz, $CDCl_3$) δ [ppm]: 7.15 (d, *J* = 8.0 Hz, 1H), 7.07 (dd, *J* = 7.9, 1.8 Hz, 1H), 7.01 (d, *J* = 1.8 Hz, 1H), 4.63 (s, 2H), 4.02 (t, *J* = 6.6 Hz, 2H), 1.87-1.77 (m, 1H), 1.71 (q, *J* = 6.7 Hz, 2H), 0.98 (d, *J* = 6.6 Hz, 6H); **^{13}C NMR** (151 MHz, $CDCl_3$) δ [ppm]: 157.5, 129.7, 128.3, 123.5, 122.0, 114.6, 66.8, 61.8, 37.8, 25.2, 22.5; **IR (ATR)** [cm^{-1}]: 3335, 2956, 2931, 2871, 1593, 1490, 1404, 1386, 1241, 1044, 1016, 835, 806; **LC-MS (DAD/ESI)**: t_r = 7.88 min, calcd for $C_{12}H_{17}BrO_2$ (*m/z*): $[M-H_2O+H]^+$ 255.04, found: $[M-H_2O+H]^+$ 255.13; **HRMS (ESI):** Calcd for $C_{12}H_{17}BrO_2$ (*m/z*): $[M+Na]^+$ 295.0304, found: $[M+Na]^+$ 295.0305

2-(5-Bromo-2-(hydroxymethyl)phenoxy)acetonitrile (S2e)

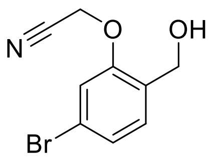

5-Bromo-2-(hydroxymethyl)phenol (**S1**) (0.50 g, 2.46 mmol, 1.0 equiv), bromoacetonitrile (0.34 ml, 4.92 mmol, 2.0 equiv), Cs_2CO_3 (0.68 g, 4.92 mmol, 2.0 equiv). Product **S2e** was obtained as light yellow solid with 42.7% (0.25 g) yield.

R_f = 0.32 (SiO_2, hexane/ethyl acetate 4:1); **^{1}H NMR** (600 MHz, $CDCl_3$) δ [ppm]: 7.31 (d, *J* = 8.1 Hz, 1H), 7.25 (dd, *J* = 8.1, 1.6 Hz, 1H), 7.08 (d, *J* = 1.7 Hz, 1H), 4.82 (s, 2H), 4.67 (s, 2H); **^{13}C NMR** (151 MHz, $CDCl_3$) δ [ppm]: 154.6, 130.5, 129.5, 126.5, 122.0, 115.5, 114.6, 60.3, 54.1; **IR (ATR)** [cm^{-1}]: 372, 3109, 3074, 2983, 2927, 2876, 2071, 1597, 1487, 1409, 1222, 1049, 851; **LC-MS (DAD/ESI)**: t_r = 4.72 min, calcd for $C_9H_8BrNO_2$ (*m/z*): $[M-H_2O+H]^+$ 223.97, found: [M-

$H_2O+H]^+$ 224.03; **HRMS (ESI):** Calcd for $C_9H_8BrNO_2$ (*m/z*): $[M+Na]^+$ 263.9631, found: $[M+Na]^+$ 263.9633

(4-Bromo-2-(cyclobutylmethoxy))methanol (S2f)

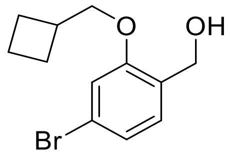

5-Bromo-2-(hydroxymethyl)phenol (**S1**) (1.00 g, 4.90 mmol, 1.0 equiv), (bromomethyl)cyclobutane (1.10 ml, 9.80 mmol, 2.0 equiv), K_2CO_3 (1.35 g, 9.80 mmol, 2.0 equiv). Product **S2f** was obtained as light yellow oil with 59.4% (0.79 g) yield.

R_f = 0.32 (SiO_2, hexane/ethyl acetate 4:1); **^{1}H NMR** (600 MHz, $CDCl_3$) δ [ppm]: 7.14 (d, J = 8.0, 1H), 7.07 (dd, J = 7.9, 1.8 Hz, 1H), 7.00 (d, J = 1.8 Hz, 1H), 4.63 (s, 2H), 3.96 (d, J = 6.6 Hz, 2H), 2.83-2.75 (m, 1H), 2.18-2.12 (m, 2H), 2.04-1.92 (m, 2H), 1.90-1.83 (m, 2H); **^{13}C NMR** (151 MHz, $CDCl_3$) δ [ppm]: 157.7, 129.8, 128.5, 123.6, 122.1, 114.9, 72.4, 61.8, 34.5, 24.9, 18.7; **IR (ATR)** [cm^{-1}]: 3323, 2975, 2935, 2864, 1593, 1488, 1403, 1237, 1045, 1013, 873; **LC-MS (DAD/ESI)**: t_r = 7.59 min, calcd for $C_{12}H_{15}BrN_2$ (*m/z*): $[M-H_2O+H]^+$ 253.07, found: $[M-H_2O+H]^+$ 253.02; **HRMS (ESI):** Calcd for $C_{12}H_{15}BrO_2$ (*m/z*): $[M+Na]^+$ 293.0148, found: $[M+Na]^+$ 293.0148

(4-Bromo-2-(cyclopentylmethoxy))methanol (S2g)

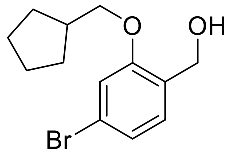

5-Bromo-2-(hydroxymethyl)phenol (**S1**) (0.62 g, 3.07 mmol, 1.0 equiv), (bromomethyl)cyclopentane (0.55 ml, 4.29 mmol, 1.4 equiv), Cs_2CO_3 (2.00 g, 6.13 mmol, 2.0 equiv). Product **S2g** was obtained as light yellow oil with 62.8% (0.55 g) yield.

R_f = 0.32 (SiO_2, hexane/ethyl acetate 4:1); **^{1}H NMR** (600 MHz, $CDCl_3$) δ [ppm]: 7.14 (d, J = 7.9 Hz, 1H), 7.07 (dd, J = 7.9, 1.8 Hz, 1H), 7.00 (d, J = 1.8 Hz, 1H), 4.64 (s, 2H), 3.88 (d, J = 6.9 Hz, 2H), 2.44-2.33 (m, 1H), 1.90-1.83 (m, 2H), 1.69-1.56 (m, 4H), 1.39-132 (m, 2H); **^{13}C NMR** (151 MHz, $CDCl_3$) δ [ppm]: 157.7, 129.8, 128.4, 123.6, 122.1, 114.8, 72.7, 61.9, 39.1, 29.6, 25.6; **IR (ATR)** [cm^{-1}]: 3335, 2952, 2866, 1593, 1489, 1404, 1238, 1028, 875; **LC-MS (DAD/ESI)**: t_r = 8.14 min, calcd for $C_{13}H_{17}BrO_2$ (*m/z*): $[M-H_2O+H]^+$ 267.04, found: $[M-H_2O+H]^+$ 267.16; **HRMS (ESI):** Calcd for $C_{13}H_{17}BrO_2$ (*m/z*): $[M+Na]^+$ 307.0304, found: $[M+Na]^+$ 307.0305

3'-((5-bromo-2-(hydroxymethyl)phenoxy)methyl)-[1,1'-biphenyl]-3-carbonitrile (S2h)

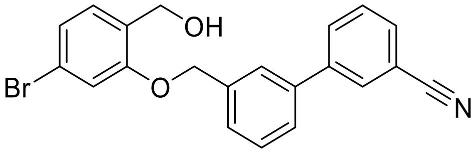

5-Bromo-2-(hydroxymethyl)phenol (**S1**) (0.45 g, 2.2 mmol, 1.0 equiv), 3'-(chloromethyl)-[1,1'-biphenyl]-3-carbonitrile (0.51 g, 2.2 mmol, 1.0 equiv), Cs_2CO_3 (1.43 g, 4.4 mmol, 2 equiv). Product **S2h** was obtained as light-yellow oil with 44.4% (0.38 g) yield.

R_f = 0.48 (SiO_2, hexane/ethyl acetate 1:1); **^{1}H NMR** (600 MHz, $CDCl_3$) δ [ppm]: 7.84 (t, *J* = 1.5 Hz, 1H), 7.80 (m, 1H), 7.62 (dt, *J* = 7.7, 1.3 Hz, 1H), 7.59 (s_{broad}, 1H), 7.55-7.49 (m, 3H), 7.45 (d, *J* = 7.3 Hz, 1H), 7.20 (d, *J* = 8.4 Hz, 1H), 7.11-7.09 (m, 2H), 5.12 (s, 2H), 4.68 (s, 2H), 2.34 (s, 1H); **^{13}C NMR** (151 MHz, $CDCl_3$) δ [ppm]: 156.8, 141.9, 139.5, 137.2, 131.6, 131.0, 130.7, 129.8, 129.8, 129.7, 128.7, 127.4, 127.1, 126.1, 124.2, 121.9, 118.8, 115.2, 113.0, 70.2, 61.0; **IR (ATR)** [cm^{-1}]:3273, 3059, 2920, 2231, 1592, 1491, 1243, 1053, 774; **LC-MS (DAD/ESI)**: t_r = 7.83 min, calcd for $C_{21}H_{16}BrNO_2$ (*m/z*): $[M-H_2O+H]^+$ 376.04, found: $[M-H_2O+H]^+$ 376.15; **HRMS (ESI):** Calcd for $C_{21}H_{16}BrNO_2$ (*m/z*): $[M+Na]^+$ 416.0257, found: $[M+Na]^+$ 416.0255

3-((5-Bromo-2-(hydroxymethyl)phenoxy)methyl)benzonitrile (S2i)

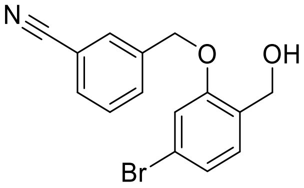

5-Bromo-2-(hydroxymethyl)phenol (**S1**) (1.0 g, 5.0 mmol, 1.0 equiv), 3-cyanobenzyl bromide (1.15 g, 5,9 mmol, 1.2 equiv), K_2CO_3 (1.03 g, 1.03 mmol, 1.5 equiv) Product **S2i** was obtained as colourless solid with 68.4% (1.09 g) yield. The product was recrystallized from cyclohexane/ethyl acetate mixture.

R_f = 0.64 (SiO_2, hexane/ethyl acetate 1:1); **^{1}H NMR** (600 MHz, DMSO-d_6) δ [ppm]: 7.91 (s, 1H), 7.81 (d, *J* = 12.3, 7.9 Hz, 1H), 7.63 (t, *J* = 7.8 Hz, 1H), 7.34 (d, *J* = 8.0 Hz, 1H), 7.23 (d, *J* = 1.7 Hz, 1H), 7.17 (dd, *J* = 8.0, 1.7 Hz, 1H), 5.22 (s, 2H), 5.14 (t, *J* = 5.6 Hz, 1H), 4.51 (d, 5.6 Hz, 2H); **^{13}C NMR** (151 MHz, DMSO-d_6) δ [ppm]: 155.5, 138.6, 132.1, 131.7, 130.7, 130.4, 129.8, 128.8, 123.4, 119.9, 118.7, 114.7, 111.5, 68.3; **IR (ATR)** [cm^{-1}]: 3288, 2922, 2230, 1594, 1489, 1400, 1054; **LC-MS (DAD/ESI)**: t_r = 6.56 min, calcd for $C_{15}H_{12}BrNO_2$ (*m/z*): $[M-H_2O+H]^+$ 300.00, found: $[M-H_2O+H]^+$ 300.06; **HRMS (ESI):** Calcd for $C_{15}H_{12}BrNO_2$ (*m/z*): $[M+Na]^+$ 339.9944, found: $[M+Na]^+$ 339.9941.

1.3. General procedure for Miyaura borylation (3a-i)

Bis(pinacolato)diboron, anhydrous potassium acetate, appropriate bromoaryl **S2a-i,** and anhydrous dioxane were placed in a two-neck round-bottom flask under argon atmosphere. The mixture was deoxygenated by rinsing with argon for half an hour, then Pd(dppf)Cl_2 dichloromethane complex was added. The reaction mixture was heated at 80°C, using a preheated bath, for three hours and after this time the progression of reaction was controlled using TLC analysis (SiO_2, hexane/ethyl acetate, 4:1). If the conversion was completed water was added and extraction with ethyl acetate followed. Organic phases were combined, dried over anhydrous $MgSO_4$ and evaporated. Crude products were purified by column chromatography (SiO_2, hexane/ethyl acetate) giving final boranes **3a-i** with 64.0-99.0% yield.

(2-Methoxy-4-(4,4,5,5-tetramethyl-1,3,2-dioxaborolan-2-yl)phenyl)methanol (3a)

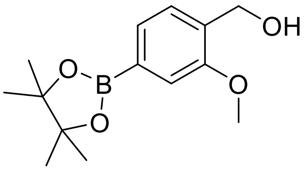

(4-Bromo-2-methoxyphenyl)methanol (**S2a**) (2.17 g, 10.0 mmol, 1.0 equiv), bis(pinacoloato)diboran (2.79 g, 11.0 mmol, 1.1 equiv), anhydrous potassium acetate (2.94 g, 30.0 mmol, 3.0 equiv), Pd(dppf)Cl_2 complex with dichloromethane (0.41 g, 0.5 mmol, 0.05 equiv). Product **3a** was obtained as colourless solid with quantitative (2.61 g) yield. Product **3a** was crystallized from a cyclohexane/ethyl acetate mixture. The compound was previously described in the literature.[4]

$\mathbf{R_f}$ **=** 0.46 (SiO_2, hexane/ethyl acetate 1:1); ^{1}**H NMR** (600 MHz, $CDCl_3$) δ [ppm]: 7.42 (d, J = 7.3 Hz, 1H), 7.30-7.28 (m, 2H), 4.70 (s, 2H), 3.91 (s, 4H), 1.35 (s, 12H); ^{13}**C NMR** (151 MHz, $CDCl_3$) δ [ppm]: 157.0, 132.4, 128.1, 127.7, 115.7, 84.0, 62.3, 55.5, 25.0; **IR (ATR)** [cm^{-1}]: 3287, 2979, 1402, 1353; **HRMS (ESI):** Calcd for $C_{14}H_{21}BO_4$ (*m/z*): $[M+Na]^+$ 287.1425, found: $[M+Na]^+$ 287.1429

(2-Ethoxy-4-(4,4,5,5-tetramethyl-1,3,2-dioxaborolan-2-yl)phenyl)methanol (3b)

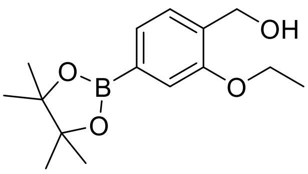

(4-Bromo-2-ethoxyphenyl)methanol (**S2b**) (0.42 g, 1.81 mmol, 1.0 equiv), bis(pinacoloato)diboran (0.55 g, 2.17 mmol, 1.2 equiv), anhydrous potassium acetate (0.53 g,

5.43 mmol, 3.0 equiv), Pd(dppf)Cl_2 complex with dichloromethane (0.07 g, 0.09 mmol, 0.05 equiv). Product **3b** was obtained as colourless oil with 81.0% (0.41 g) yield. Compounds was previously described in patent application.[5]

R_f = 0.46 (SiO_2, hexane/ethyl acetate 1:1); **^{1}H NMR** (600 MHz, $CDCl_3$) δ [ppm]: 7.39 (m, 1H), 7.28 (m, 2H), 4.70 (s, 2H), 4.16 (q, J = 7.0 Hz, 2H), 2.44 (s_{broad}, 1H), 1.44 (t, J = 7.0 Hz, 3H), 1.34 (s, 12H); **^{13}C NMR** (151 MHz, $CDCl_3$) δ [ppm]: 156.4, 132.3, 128.0, 127.5, 116.6, 83.9, 63.6, 62.6, 24.9, 15.0; **IR (ATR)** [cm^{-1}]: 3486, 2985, 1414, 1351, 1242, 1145; **HRMS (ESI):** Calcd for $C_{15}H_{23}BO_4$ (*m/z*): $[M+Na]^+$ 301.1582, found: $[M+Na]^+$ 301.1582

(2-Isopropoxy-4-(4,4,5,5-tetramethyl-1,3,2-dioxaborolan-2-yl)phenyl)methanol (3c)

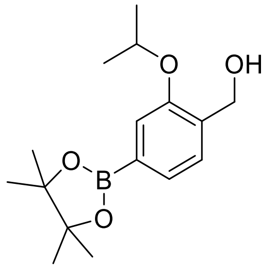

(4-Bromo-2-isopropoxyphenyl)methanol (**S2c**) (0.51 g, 2.10 mmol, 1.0 equiv), bis(pinacoloato)diboran (0.59 g, 2.31 mmol, 1.1 equiv), anhydrous potassium acetate (0.62 g, 6.30 mmol, 3.0 equiv), Pd(dppf)Cl_2 complex with dichloromethane (0.05 g, 0.06 mmol, 0.03 equiv). Product **3c** was obtained as colourless oil with 64.0% (0.39 g) yield.

R_f = 0.19 (SiO_2, hexane/ethyl acetate 4:1); **^{1}H NMR** (600 MHz, $CDCl_3$) δ [ppm]: 7.30 (d, J = 7.3 Hz, 1H), 7.23 (s, 1H), 7.19 (d, H = 7.3 Hz, 1H), 4.67 (hept, J = 6.0 Hz, 1H), 4.60 (s, 2H), 1.29 (d, J = 6.1 Hz, 6H), 1.27 (s, 12H); **^{13}C NMR** (151 MHz, $CDCl_3$) δ [ppm]: 155.5, 133.4,128.3, 127.5, 118.3, 84.0, 70.3, 62.8, 25.0, 22.4; **HRMS (ESI):** Calcd for $C_{16}H_{25}BO_4$ (*m/z*): $[M+Na]^+$ 315.1738, found: $[M+Na]^+$ 315.1741

(2-Isopentoxy-4-(4,4,5,5-tetramethyl-1,3,2-dioxaborolan-2-yl)phenyl)methanol (3d)

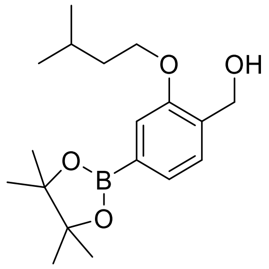

(4-Bromo-2-isopentoxyphenyl)methanol (**S2d**) (0.29 g, 1.09 mmol, 1.0 equiv), bis(pinacoloato)diboran (0.30 g, 1.30 mmol, 1.1 equiv), anhydrous potassium acetate (0.32 g,

3.27 mmol, 3.0 equiv), Pd(dppf)Cl_2 complex with dichloromethane (0.03 g, 0.03 mmol, 0.03 equiv). Product **3d** was obtained as colourless oil with 68.4% (0.35 g) yield.

R_f = 0.25 (SiO_2, hexane/ethyl acetate 4:1); **^{1}H NMR** (600 MHz, $CDCl_3$) δ [ppm]: 7.40 (d, J = 7.3 Hz, 1H), 7.29-7.27 (m, 2H), 4.70 (s, 2H) 4.10 (t, J = 6.5 Hz, 2H), 1.84 (sept, J = 6.8 Hz, 1H), 1.71 (q, J = 6.6 Hz, 2H), 1.35 (s, 12H), 0.98 (d, J = 6.6 Hz, 6H); **^{13}C NMR** (151 MHz, $CDCl_3$) δ [ppm]: 156.6, 132.4, 128.0, 127.6, 116.5, 84.0, 66.5, 62.7, 38.3, 25.4, 25.0, 22.7; **IR (ATR)** [cm^{-1}]: 3445, 2977, 2930, 1410, 1353, 1235, 1145; **HRMS (ESI):** Calcd for $C_{18}H_{29}BO_4$ (*m/z*): $[M+Na]^+$ 343.2051, found: $[M+Na]^+$ 343.2052

2-(2-Hydroxymethyl)-5-(4,4,5,5-tetramethyl-1,3,2-dioxaborolan-2-yl)phenoxy)acetonitrile (3e)

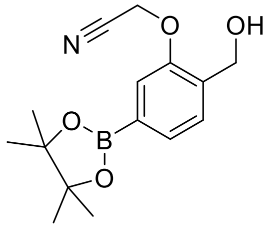

2-(5-Bromo-2-(hydroxymethyl)phenoxy)acetonitrile (**S2e**) (0.49 g, 2.04 mmol, 1.0 equiv), bis(pinacoloato)diboran (0.57 g, 2.24 mmol, 1.1 equiv), anhydrous potassium acetate (0.60 g, 6.12 mmol, 3.0 equiv), Pd(dppf)Cl_2 complex with dichloromethane (0.05 g, 0.06 mmol, 0.03 equiv). Product **3e** was obtained as light-yellow solid with 96.6% (0.57 g) yield.

R_f = 0.30 (SiO_2, hexane/ethyl acetate 4:1); **^{1}H NMR** (600 MHz, $CDCl_3$) δ [ppm]: 7.56 (d, J = 7.3 Hz, 1H), 7.43 (d, J = 7.3 Hz, 1H), 7.31 (s, 1H), 4.88 (s, 2H), 4.75 (s, 2H), 1.35 (s, 12 H); **^{13}C NMR** (151 MHz, $CDCl_3$) δ [ppm]: 153.8, 133.4, 130.1, 128.7, 117.0, 115.2, 84.3, 61.0, 53.9, 25.0; **IR (ATR)** [cm^{-1}]: 3320, 2983, 2937, 1410, 1351, 1237, 1143, 1050, 852; **HRMS (ESI):** Calcd for $C_{15}H_{20}BNO_4$ (*m/z*): $[M+Na]^+$ 312.1378, found: $[M+Na]^+$ 312.1381.

(2-(Cyclobutylmethoxy)-4-(4,4,5,5-tetramethyl-1,3,2-dioxaborolan-2-yl)phenyl)methanol (3f)

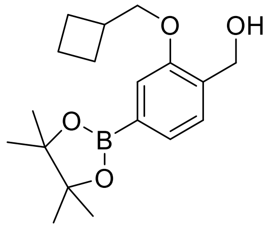

(4-Bromo-2-(cyclobutylmethoxy))methanol (**S2f**) (0.78 g, 2.9 mmol, 1.0 equiv), bis(pinacoloato)diboran (0.81 g, 3.19 mmol, 1.1 equiv), anhydrous potassium acetate (0.85 g, 8.70 mmol, 3.0 equiv), Pd(dppf)Cl_2 complex with dichloromethane (0.07 g, 0.09 mmol, 0.03 equiv). Product **3f** was obtained as colourless oil with 71.6% (0.66 g) yield.

R_f = 0.26 (SiO_2, hexane/ethyl acetate 4:1); **^{1}H NMR** (600 MHz, $CDCl_3$) δ [ppm]: 7.40 (d, J = 7.4 Hz, 1H), 7.28-7.26 (m, 2H), 4.70 (s, 2H), 4.06 (d, J = 6.6 Hz, 2H), 2.84-2.76 (m, 1H), 2.19-2.11 (m, 2H), 2.03-1.84 (m, 4H), 1.35 (s, 12H); **^{13}C NMR** (151 MHz, $CDCl_3$) δ [ppm]: 156.8, 132.5, 128.1, 127.6, 116.7, 84.0, 72.2, 62.8, 34.8, 29.8, 25.0, 25.0, 18.8; **IR (ATR)** [cm^{-1}]: 3417, 2977, 2933, 2861, 1409, 1354, 1233, 1145, 855; **HRMS (ESI):** Calcd for $C_{18}H_{27}BO_4$ (m/z): $[M+Na]^+$ 341.1895, found: $[M+Na]^+$ 341.1899

(2-(Cyclopentylmethoxy)-4-(4,4,5,5-tetramethyl-1,3,2-dioxaborolan-2-yl)phenyl)methanol (3g)

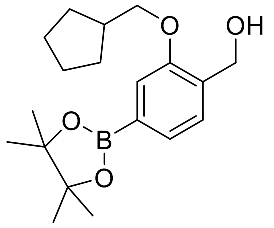

(4-Bromo-2-(cyclopentylmethoxy))methanol (**S2g**) (0.50 g, 1.75 mmol, 1.0 equiv), bis(pinacoloato)diboran (0.49 g, 1,93 mmol, 1.1 equiv), anhydrous potassium acetate (0.52 g, 5.25 mmol, 3.0 equiv), Pd(dppf)Cl_2 complex with dichloromethane (0.04 g, 0.05 mmol, 0.03 equiv). Product **3g** was obtained as colourless oil with 97.5% (0.55 g) yield.

R_f = 0.26 (SiO_2, hexane/ethyl acetate 4:1); **^{1}H NMR** (600 MHz, $CDCl_3$) δ [ppm]: 7.39 (d, J = 7.3 Hz, 1H), 7.28-7.25 (m, 2H), 4.70 (s, 2H), 3.96 (d, J = 6.9 Hz, 2H), 2.41-2.36 (m, 1H), 1.89-1.82 (m, 2H), 1.68-1.58 (m, 4H), 1.39-1.35 (m, 2H), 1.34 (s, 12H); **^{13}C NMR** (151 MHz, $CDCl_3$) δ [ppm]: 156.3, 132.4, 128.0, 127.6, 116.5, 84.0, 72.3, 62.8, 39.3, 29.7, 25.6, 25.0; **IR (ATR)** [cm^{-1}]: 3435, 2956, 2866, 1410, 1354, 1238, 1146, 1051, 855; **HRMS (ESI):** Calcd for $C_{19}H_{29}BO_4$ (m/z): $[M+Na]^+$ 355.2051, found: $[M+Na]^+$ 355.2053

3'-((2-(Hydroxymethyl)-5-(4,4,5,5-tetramethyl-1,3,2-dioxaborolan-2-yl)phenoxy)methyl)-[1,1'-biphenyl]-3-carbonitrile (3h)

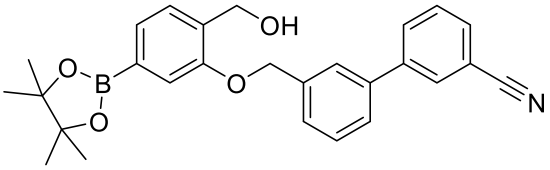

3'-((5-Bromo-2-(hydroxymethyl)phenoxy)methyl)-[1,1'-biphenyl]-3-carbonitrile (**S2h**) (0.50 g, 1.27 mmol, 1.0 equiv), bis(pinacoloato)diboran (0.48 g, 1.90 mmol, 1.5 equiv), anhydrous potassium acetate (0.37 g, 3.81 mmol, 3.0 equiv), Pd(dppf)Cl_2 complex with dichloromethane (0.05 g, 0.06 mmol, 0.05 equiv). Product **3h** was obtained as colourless solid with 92.0% (0.52 g) yield.

R_f = 0.46 (SiO_2, hexane/ethyl acetate 1:1); **^{1}H NMR** (600 MHz, $CDCl_3$) δ [ppm]: 7.87 (t, *J* = 1.6 Hz, 1H), 7.81 (m, 1H), 7.65-7.63 (m, 2H), 7.56-7.48 (m, 4H), 7.47 (d, *J* = 7.3 Hz, 1H), 7.43 (s, 1H), 7.35 (d, *J* = 7.3 Hz, 1H), 5.21 (s, 2H), 4.76 (s, 2H), 2.38 (s_{broad}, 1H), 1.35 (s, 12H); **^{13}C NMR** (151 MHz, $CDCl_3$) δ [ppm]: 155.9, 142.1, 139.3, 138.0, 132.7, 131.6, 130.9, 130.7, 129.7, 129.6, 128.1, 128.0, 127.5, 126.9, 126.3, 118.8, 116.9, 113.1, 84.0, 69.9, 62.0, 24.9; **IR (ATR)** [cm^{-1}]: 3471, 2977, 2230, 1408, 1352, 1144; **HRMS (ESI)**: Calcd for $C_{27}H_{28}BNO_4$ (*m/z*): $[M+Na]^+$ 464.2004, found: $[M+Na]^+$ 464.2008

3-((2-(Hydroxymethyl)-5-(4,4,5,5-tetramethyl-1,3,2-dioxaborolan-2-yl)phenoxy)methyl)benzonitrile (3i)

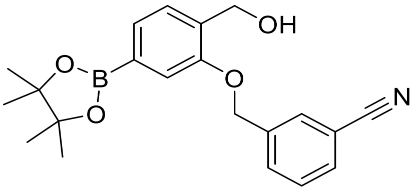

3-((5-Bromo-2-(hydroxymethyl)phenoxy)methyl)benzonitrile (**S2i**) **(**0.95 g, 3.0 mmol, 1.0 equiv), bis(pinacoloato)diboran (0.84 g, 3.3 mmol, 1.1 equiv), anhydrous potassium acetate (0.88 g, 9.0 mmol, 3.0 eq.), Pd(dppf)Cl_2 complex with dichloromethane (0.12 g, 0.15 mmol, 0.05 equiv). Product **3i** was obtained as colourless solid with 76.1% (0.83 g) yield. Product **3i** was crystallized from cyclohexane/ethyl acetate mixture.

R_f = 0.63 (SiO_2, hexane/ethyl acetate 1:1); **^{1}H NMR** (600 MHz, DMSO-d_6) δ [ppm]: 7.93 (s, 1H), 7.83 (d, *J* = 13.5, 7.8 Hz, 1H), 7.63 (t, *J* = 7.8 Hz, 1H), 7.46 (d, *J* = 7.4 Hz, 1H), 7.33 (d, *J* = 7.4 Hz, 1H), 7.24 (s, 1H), 5.20 (s, 2H), 5.30 (t, *J* = 5.5 Hz, 1H), 4.60 (d, *J* = 5.0 Hz, 2H), 1.30 (s, 12H); **^{13}C NMR** (151 MHz, DMSO-d_6) δ [ppm]: 154.3, 139.1, 134.5, 132.0, 131.5, 130.6, 129.7,

127.4, 126.5, 118.8, 116.1, 111.4, 83.7, 67.9, 58.0, 24.7; **IR (ATR)** [cm^{-1}]: 3435, 2980, 2227, 1571, 1509, 1405, 1351, 1238, 1141, 1064, 968, 924, 853, 785, 683; **HRMS (ESI):** Calcd for $C_{21}H_{24}BNO_4$ (*m/z*): $[M+Na]^+$ 388.1691, found: $[M+Na]^+$ 388.1696

2. SUPPRORTING FIGURES AND TABLES

Table S1. Structures and HTRF data for obtained compounds

No	Structure	% of undissociated complex at 5 μM	IC_{50} Determined [μM]	IC_{50} Plot
**4a**	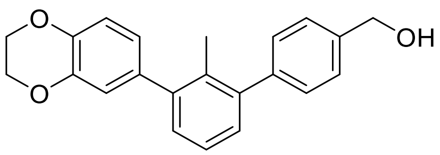	42	**5.52±0.04**	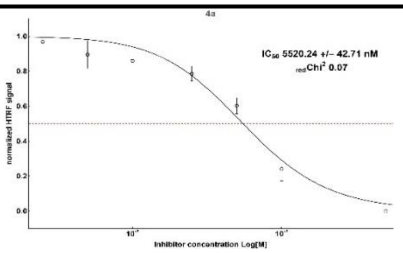
**4b**	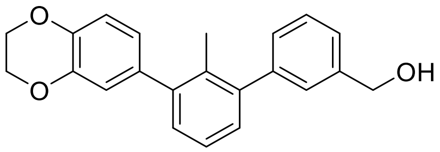	88	**-**	**--**
**4c**	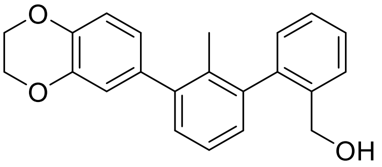	98	**-**	**-**
**4d**	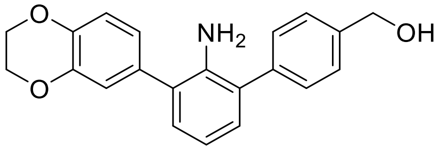	73	**-**	**-**
**4e**	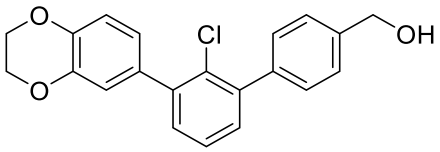	1	**0.51±0.01**	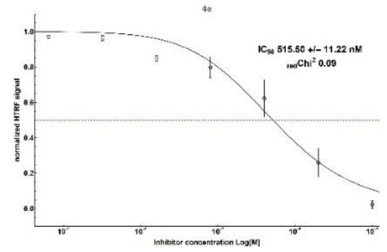
**4f**	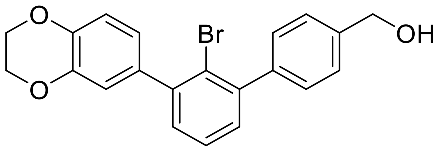	15	**0.95±0.02**	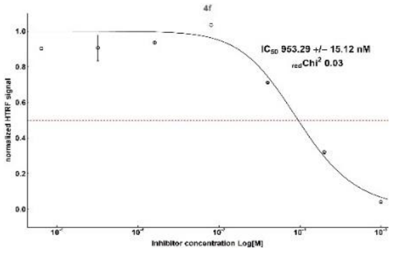
**4g**	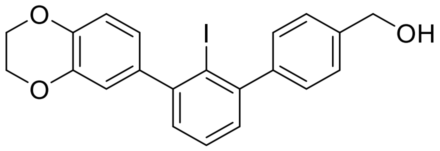	56	**-**	**-**

**4h**	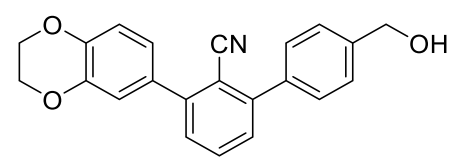	21	**1.17±0.12**	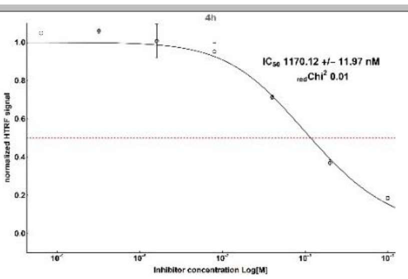
**5b**	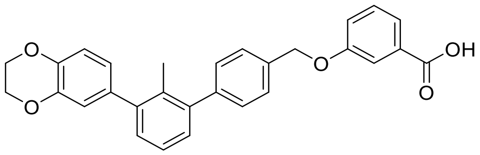	71 at 50 µM	**-**	**-**
**5d**	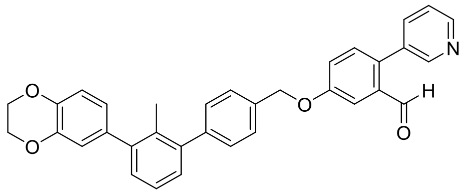	65 at 50 µM	**-**	**-**
**5f**	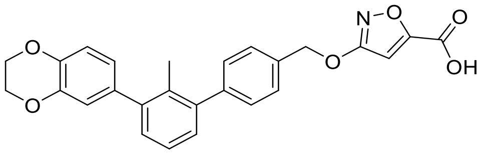	90	**-**	**-**
**5h**	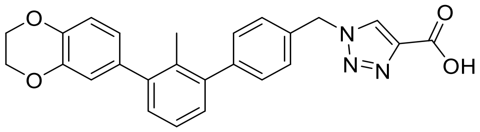	100	**-**	**-**
**5j**	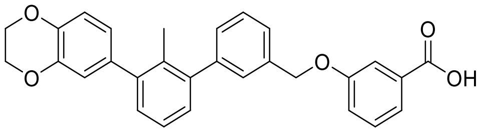	90	**-**	**-**
**5l**	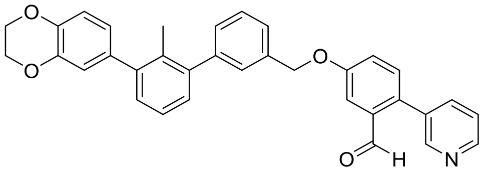	95 at 50 µM	**-**	**-**
**5n**	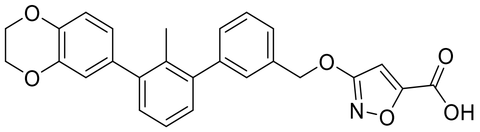	77	**-**	**-**
**5p**	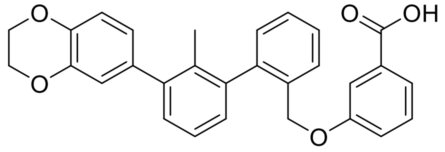	87 at 50 µM	**-**	**-**
**5r**	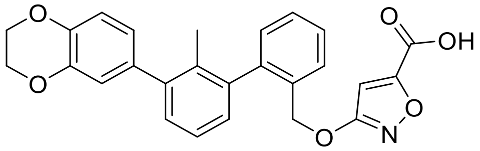	100	**-**	**-**

**6a**	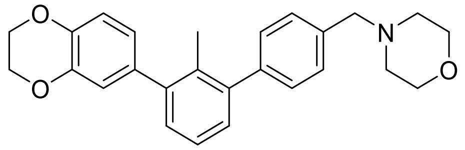	55	-	-
**6b**	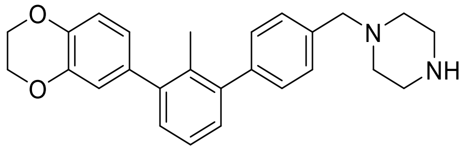	5	-	-
**6c**	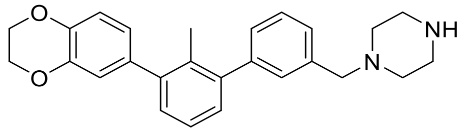	56	-	-
		**% of undissociated complex at 5 nM**	**Determined [nM]**	
**6d**	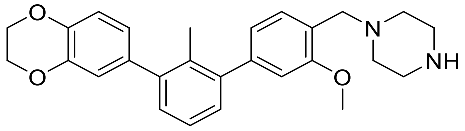	63	-	-
**6e**	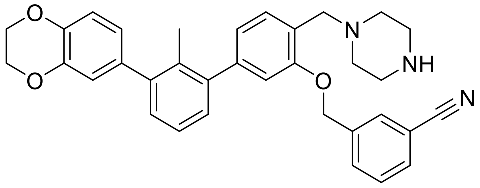	88	-	-
**7a**	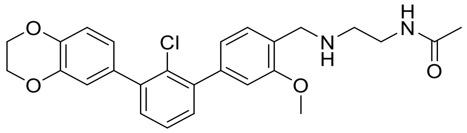	0	**0.82±0.02**	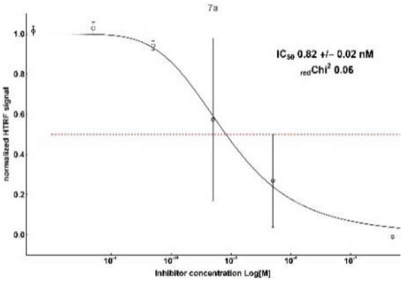
**7b**	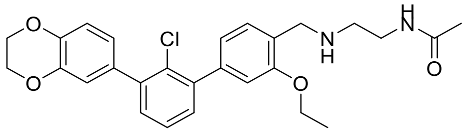	5	-	-
**7c**	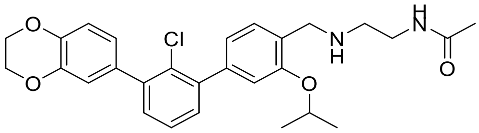	5	-	-
**7d**	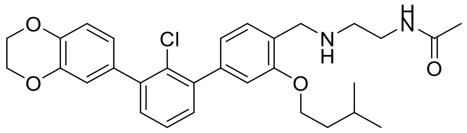	65	-	-
**7e**	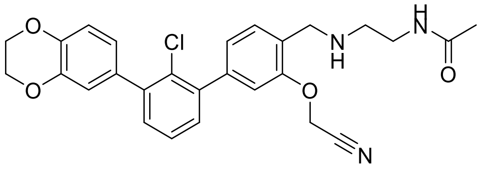	1	-	-

**7f**	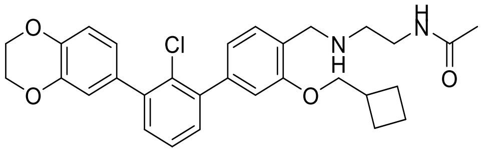	53	-	-
**7g**	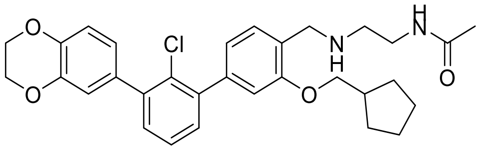	76	-	-
**7h**	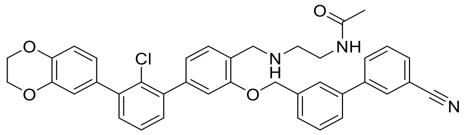	100	-	-
**8a**	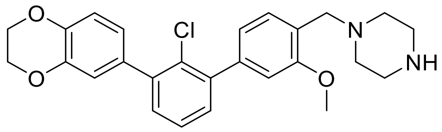	38	**18.09±0.09**	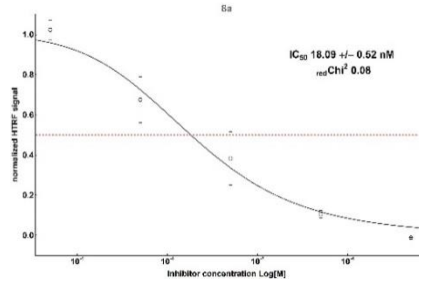
**8b**	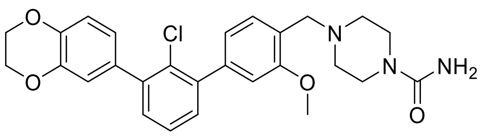	28	-	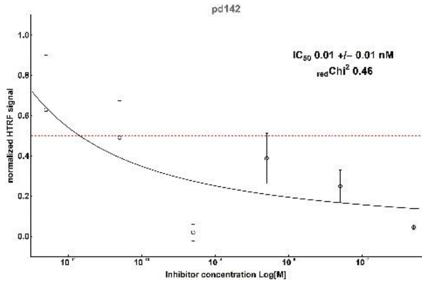
**8c**	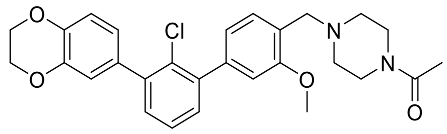	40	-	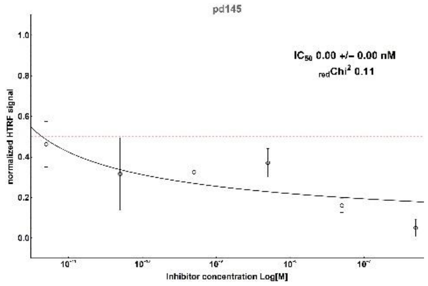
**8d**	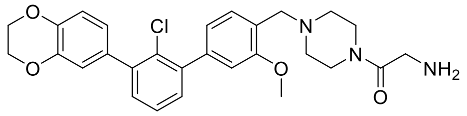	5	-	-
**8e**	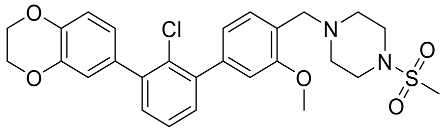	88	**104.44±2.17**	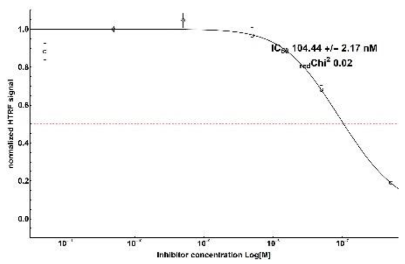

**8f**	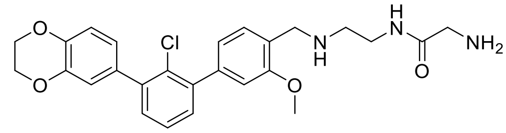	8	**-**	**-**
**8g**	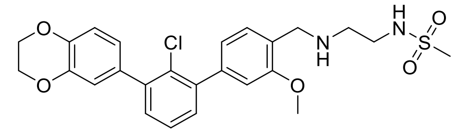	32	**2.07±0.04**	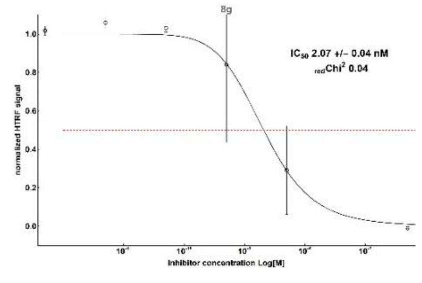
**8h**	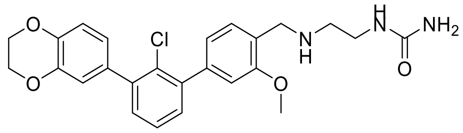	6	**-**	**-**
**8i**	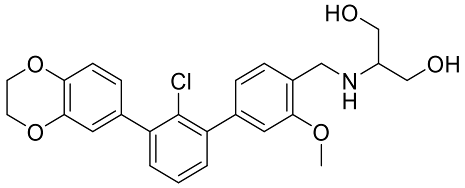	0	**-**	**-**
**8j**	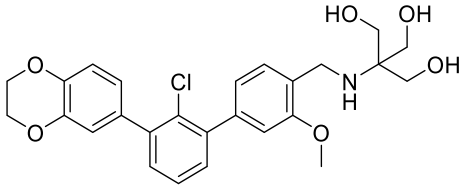	0	**-**	**-**
**BMS-1166**		53	**3.89±0.19**	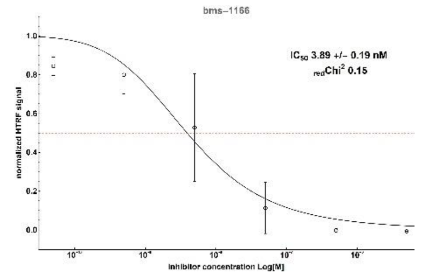

Table S2. Data collection and refinement statistics (molecular replacement)

**Data collection**	
Wavelength (Å)	0.9184
Space group	P 1 21 1
Cell dimensions	
a, b, c (Å)	67.09 75.25 74.35
α, β, γ (o)	90.0 96.29 90.0
Resolution range (Å)	47.01 - 2.30 (2.42- 2.30)
Rmerge	0.119 (3.196)
I/σI	15.00 (1.0)
Completeness (%)	99.0 (98.7)
Redundancy	13.4 (13.0)
Total reflections	217995 (21254)
CC1/2	1.0 (0.77)
**Refinement statistics**	
No. reflections	32189 (3143)
*R*work/*R*free	0.246/0.290
	(0.425/0.471)
Wilson B-factor	53.15
No. atoms	5360
Protein	5216
Water	42
Ramachandran favoured (%)	91.94
Ramachandran allowed (%)	7.18
Ramachandran outliers (%)	0.88
B-factors	56.60
Protein	56.24
Water	52.59
R.m.s deviations	
Bond lengths (Å)	0.011
Bond angles (o)	1.11

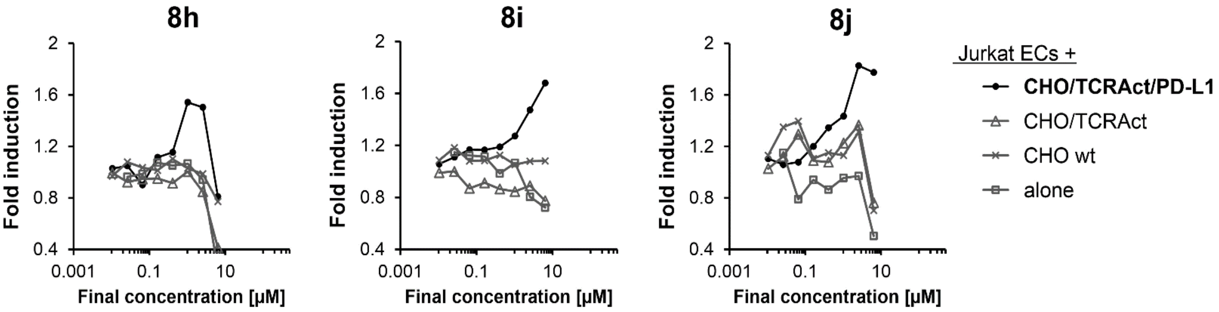

Figure S1. Terphenyl compounds **8h**, **8i**, and **8j** bioactivity requires the presence of PD-L1 in the ICB assay. Results of the Immune Checkpoint Blockade (ICB) assay, where **8h**, **8i**, and **8j** were used to restore the activation of effector Jurkat-ECs. Jurkat-ECs were either kept alone, or contacted with one of the following cell lines: wild type CHO K1 cells (CHO wt), CHO K1 cells expressing TCR Activator (CHO/TCRAct), or CHO K1 cells expressing TCR Activator and PD-1 receptor (CHO/TCRAct/PD-1). The cells were incubated with the indicated concentrations of the compounds for 6 hours and luminescence was measured as an indicator of TCR-dependent activation of Jurkat-ECs. The graphs present fold induction of the luminescence signal relative to DMSO-treated cells. Increased activation of Jurkat-ECs is observed only when the cells were contacted with PD-L1-expressing CHO/TCRAct/PD-L1 cells.

A

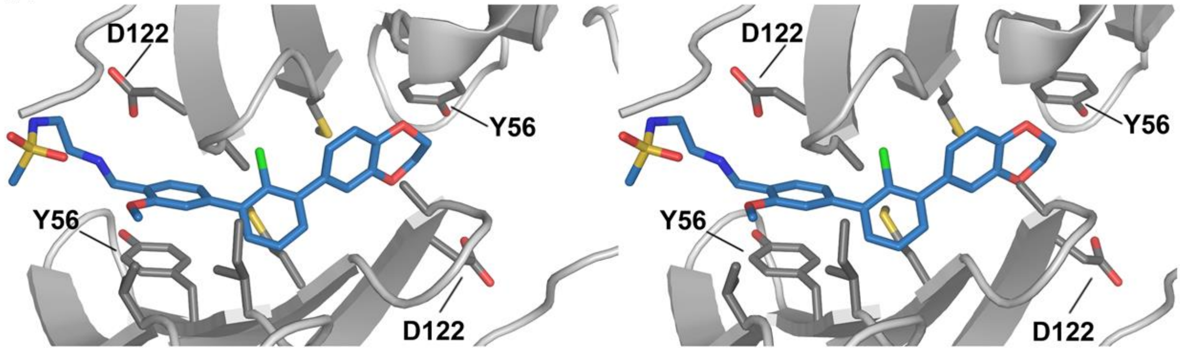
D122
Y56
Y56
D122
D122
Y56
Y56
D122

B

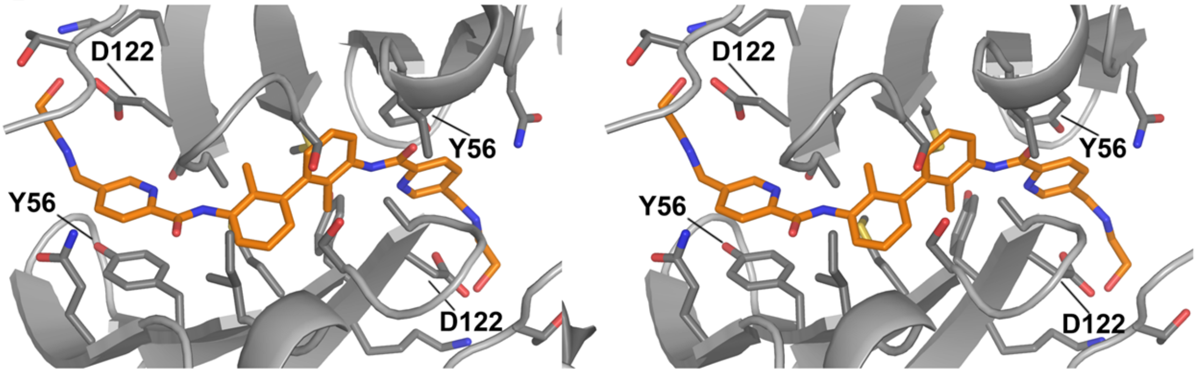
D122
Y56
Y56
D122
D122
Y56
Y56
D122

C

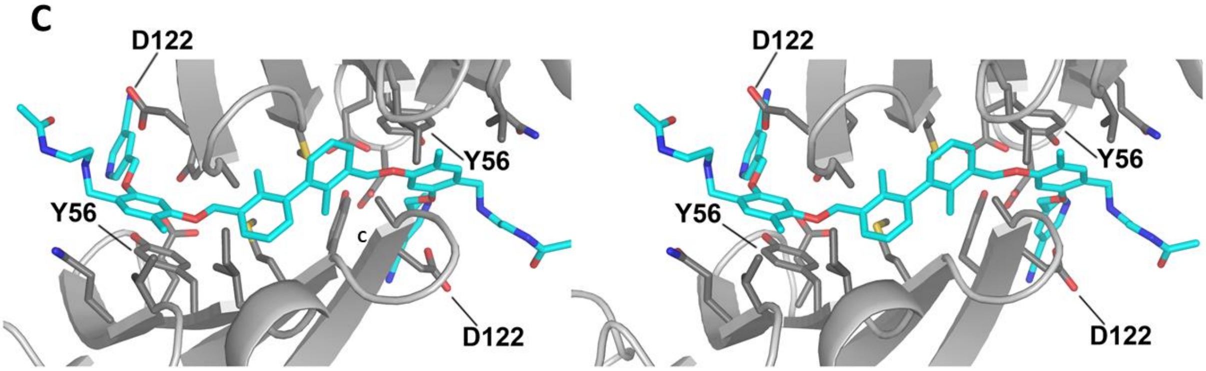
D122
Y56
Y56
D122
D122
Y56
Y56
D122

D

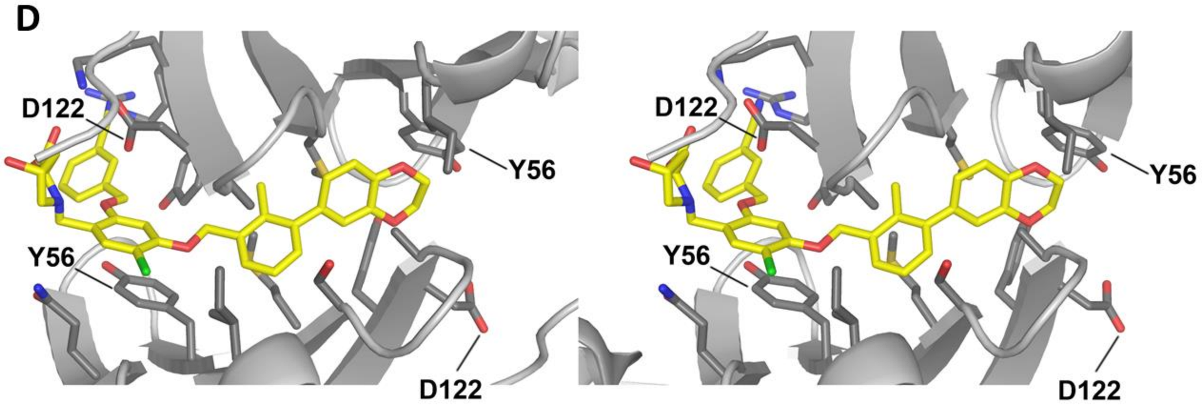
D122
Y56
Y56
D122
D122
Y56
Y56
D122

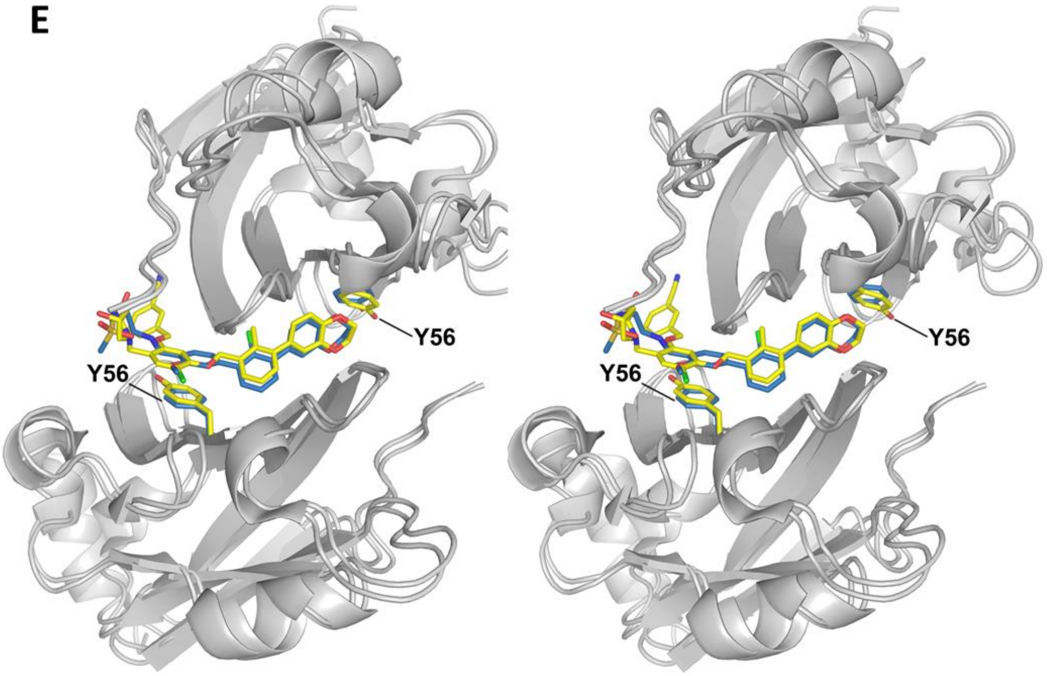
E
Y56
Y56
Y56
Y56

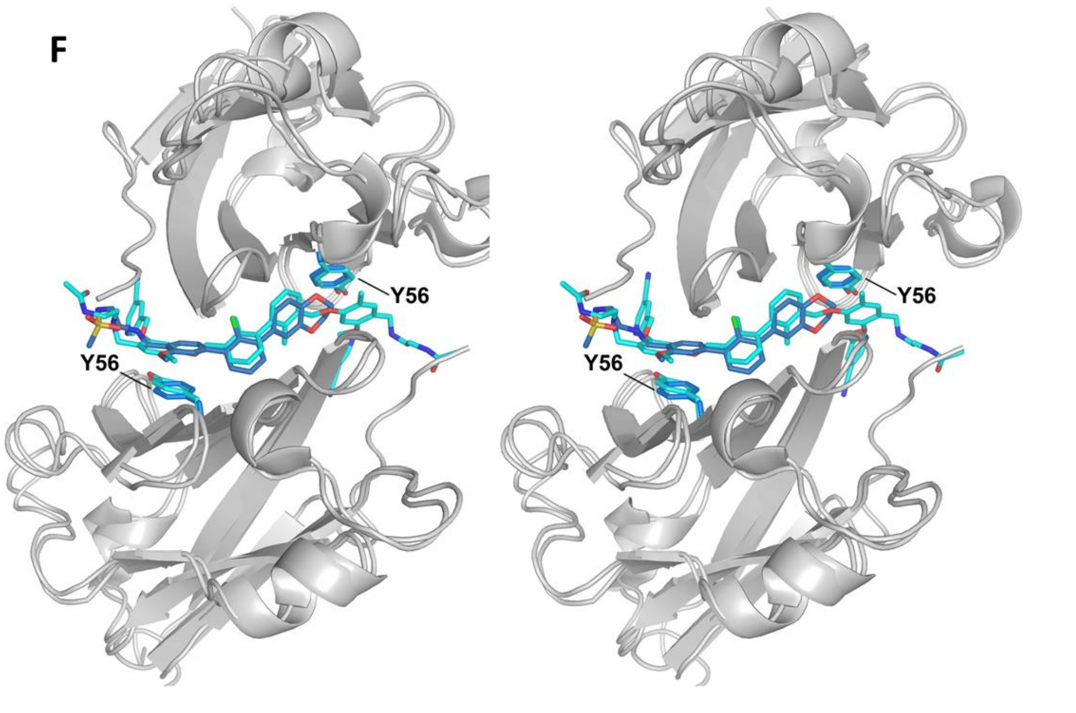
F
Y56
Y56
Y56
Y56

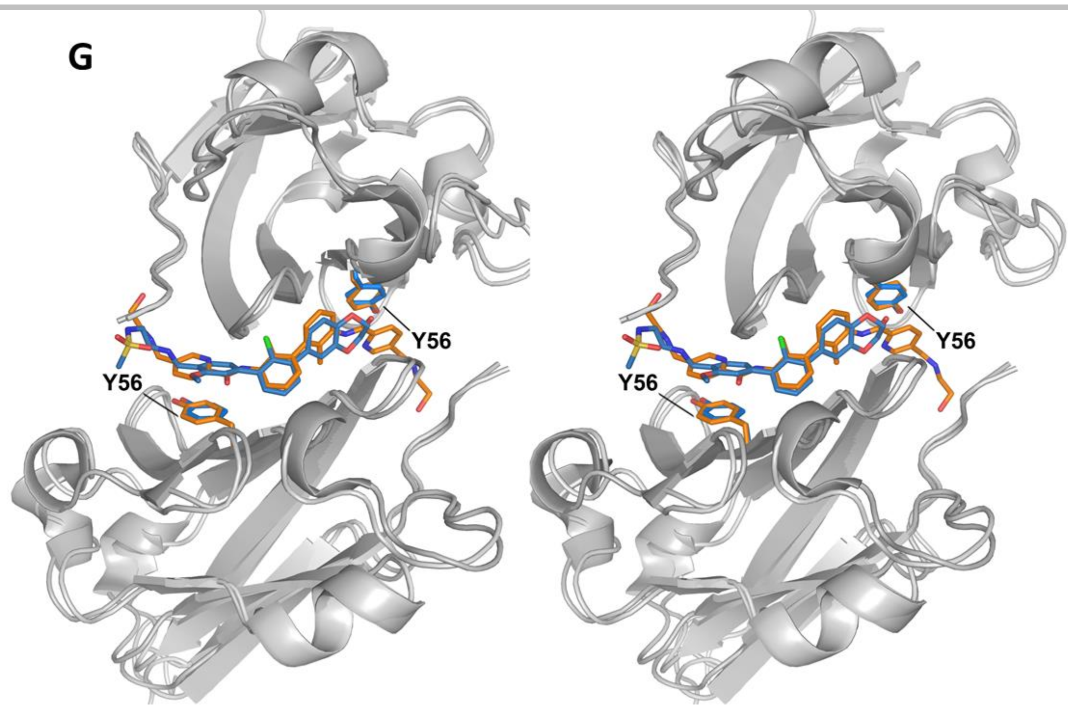

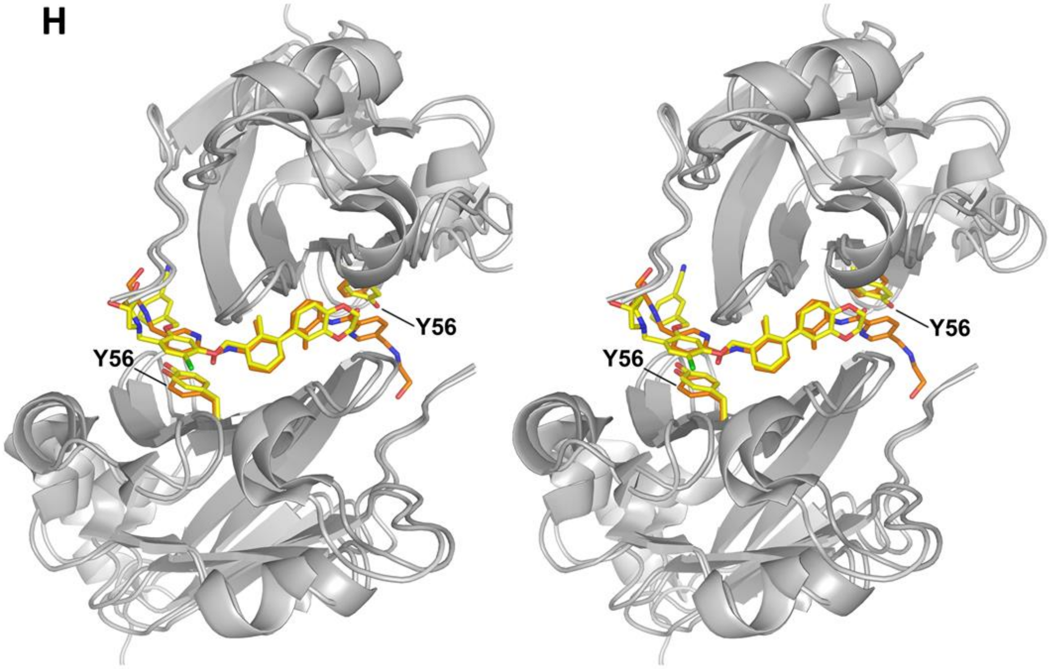

Figure S2. Comparison of the terphenyl-based with biphenyl-based inhibitor structures. (A-D) The stereoviews of detailed interactions of **8g** (blue), **ARB-272572 - Compound A** (orange), the C2-symmetric inhibitor (cyan) and **BMS-1166** (yellow) with the PD-L1 molecules. (E) Superposition of the **8g**/PD-L1 (blue) and **BMS-1166**/PD-L1 (yellow) structures (stereoview). (F) Superposition of the **8g**/PD-L1 (blue) and the C2-symmetric inhibitor/PD-L1 (cyan) structures (stereoview). (G) Superposition of the **8g**/PD-L1 (blue) and **ARB-272572**/PD-L1 (orange) structures (stereoview). (H) Superposition of the **BMS-1166**/PD-L1 (yellow) and **ARB-272572**/PD-L1 (orange) structures (stereoview).

3. COPIES OF NMR SPECTRA

2a. 6-(3-Bromo-2-methylphenyl)-1,4-benzodioxane

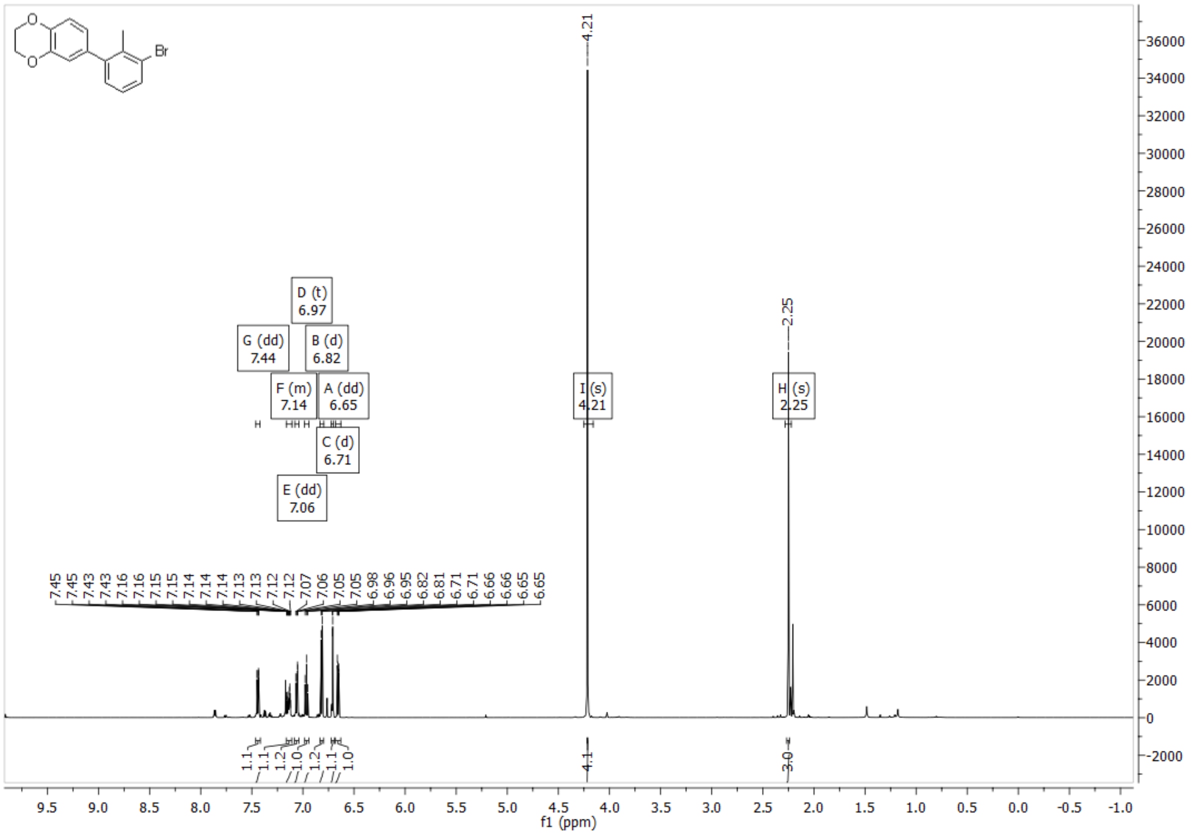

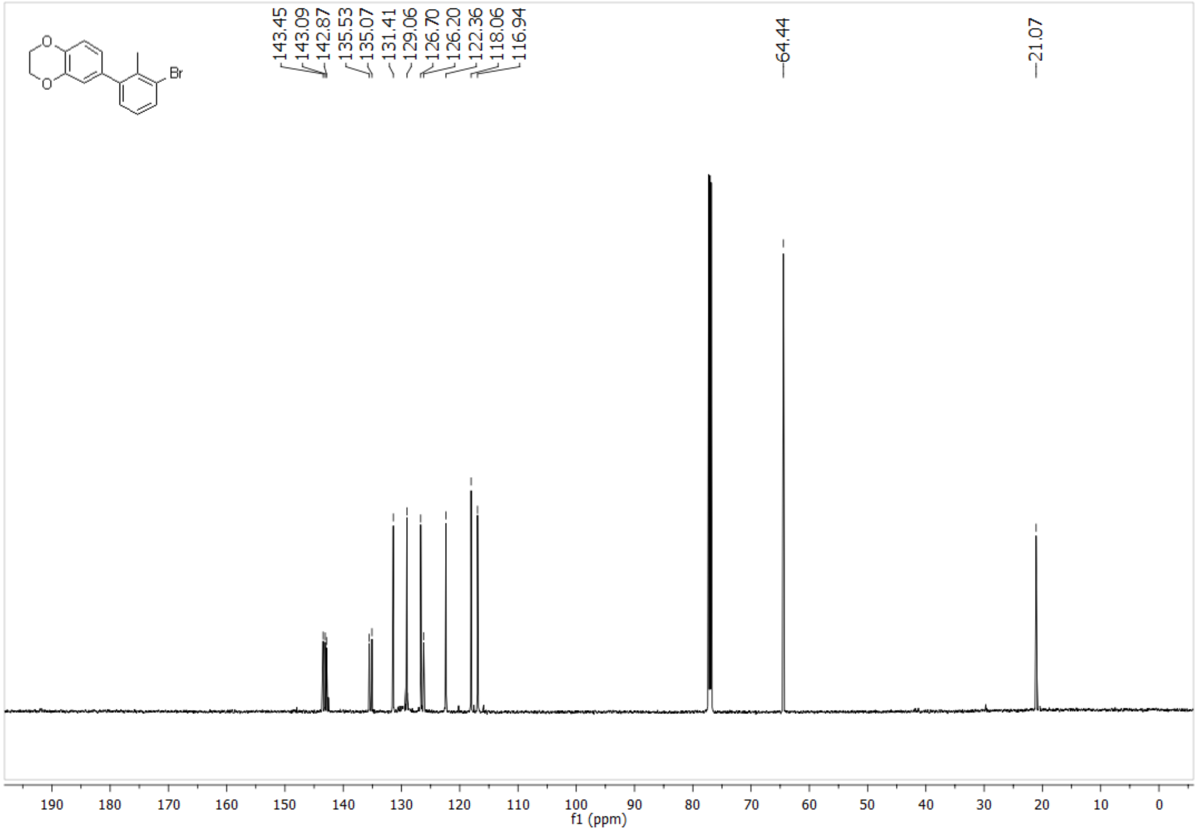

2b. 6-(2-Amino-3-bromophenyl)-1,4-benzodioxane

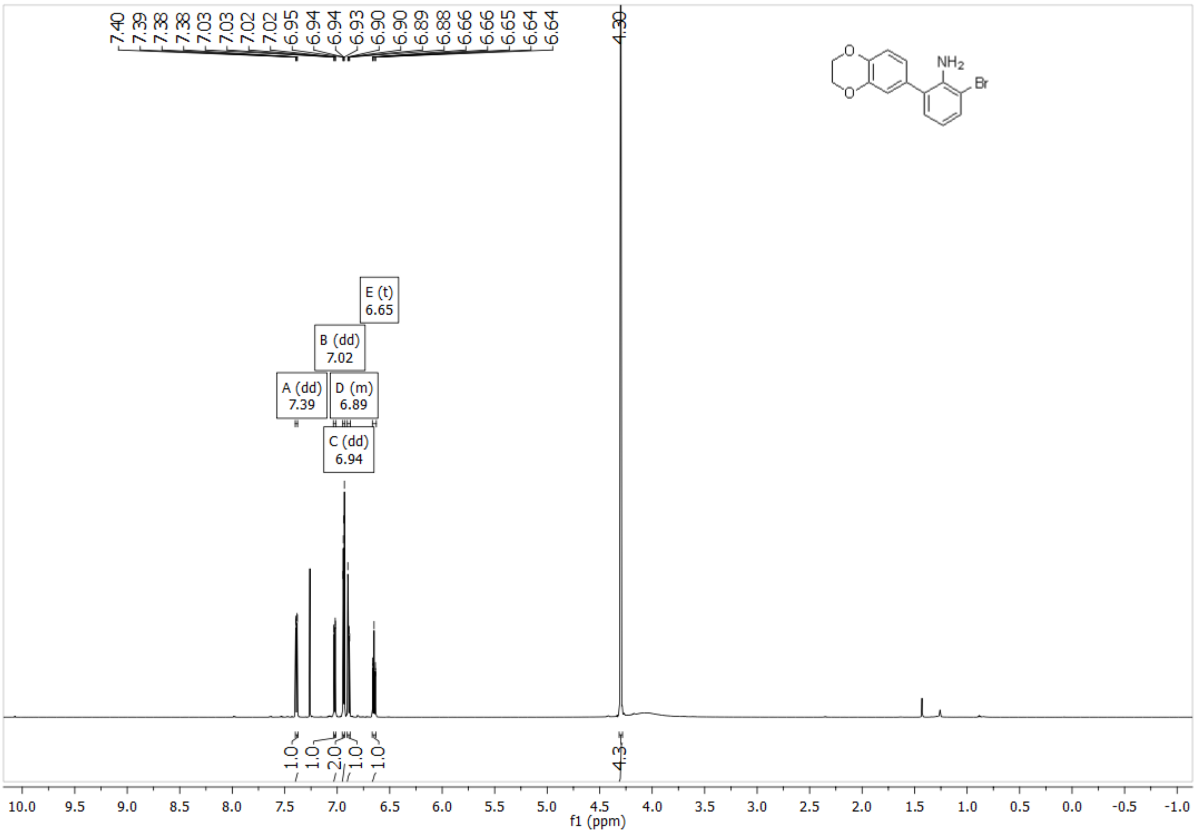

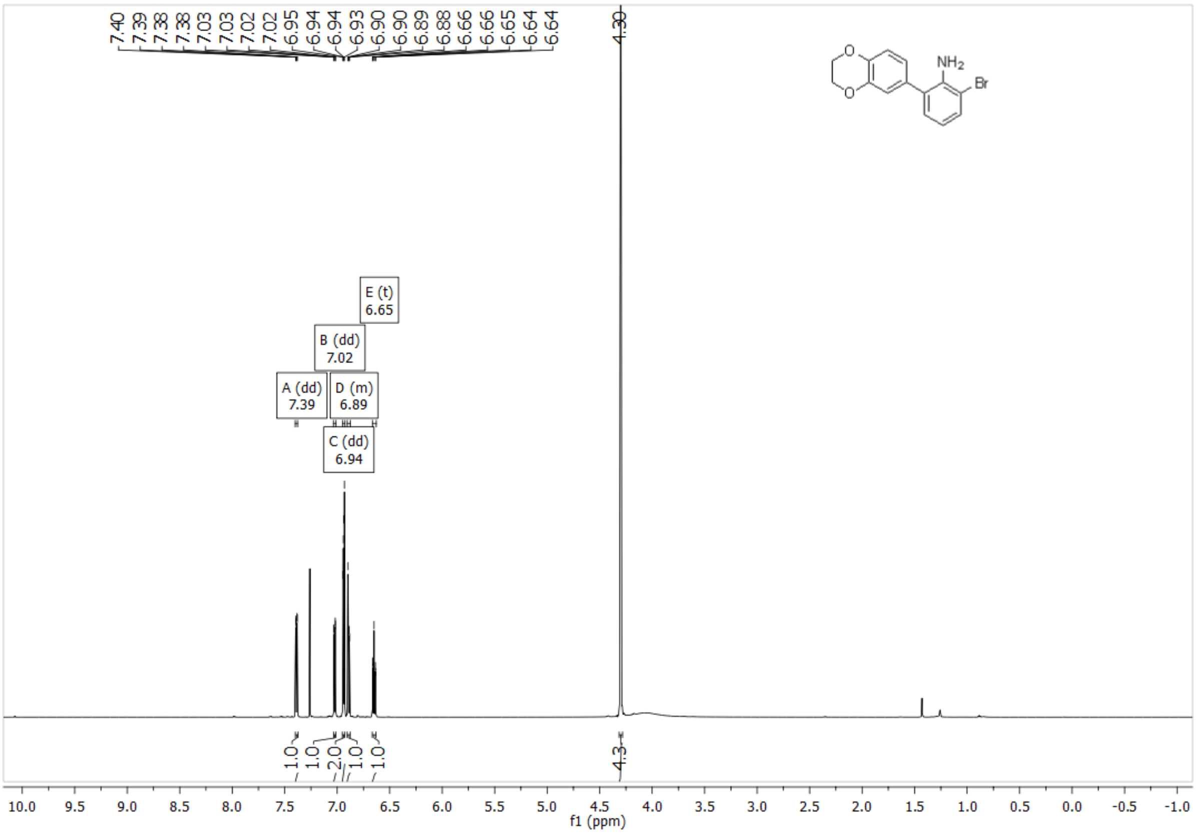

2c. 6-(3-Bromo-2-chlorophenyl)-1,4-benzodioxane

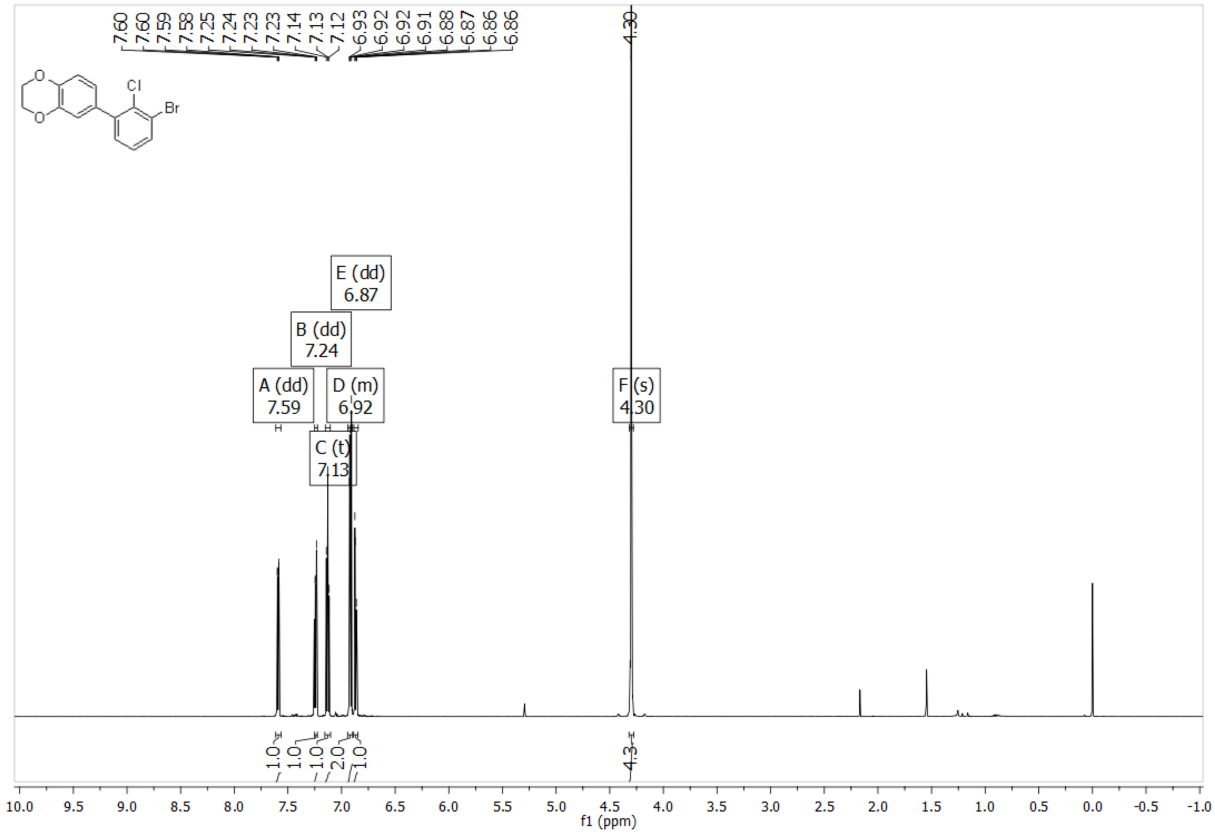

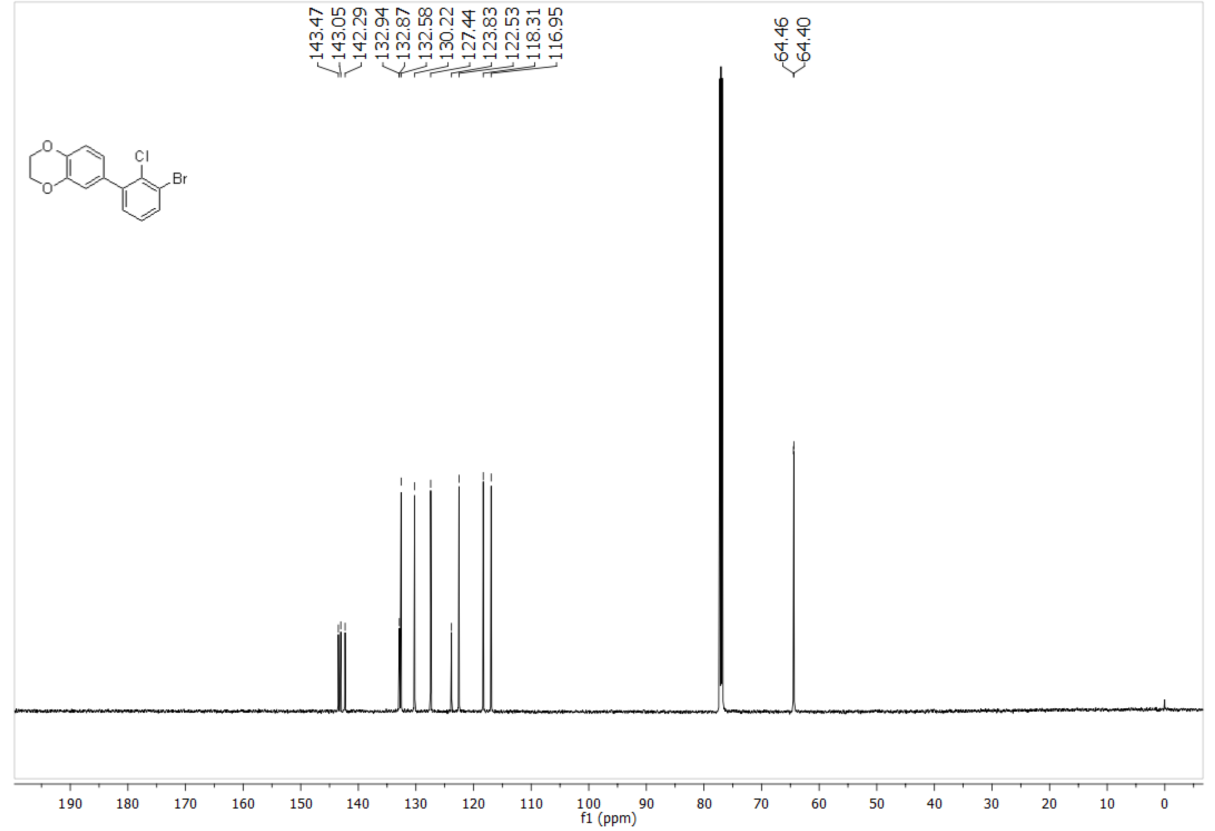

I-O. 4-bromo-2-hydroxymethylbenzyl alcohol

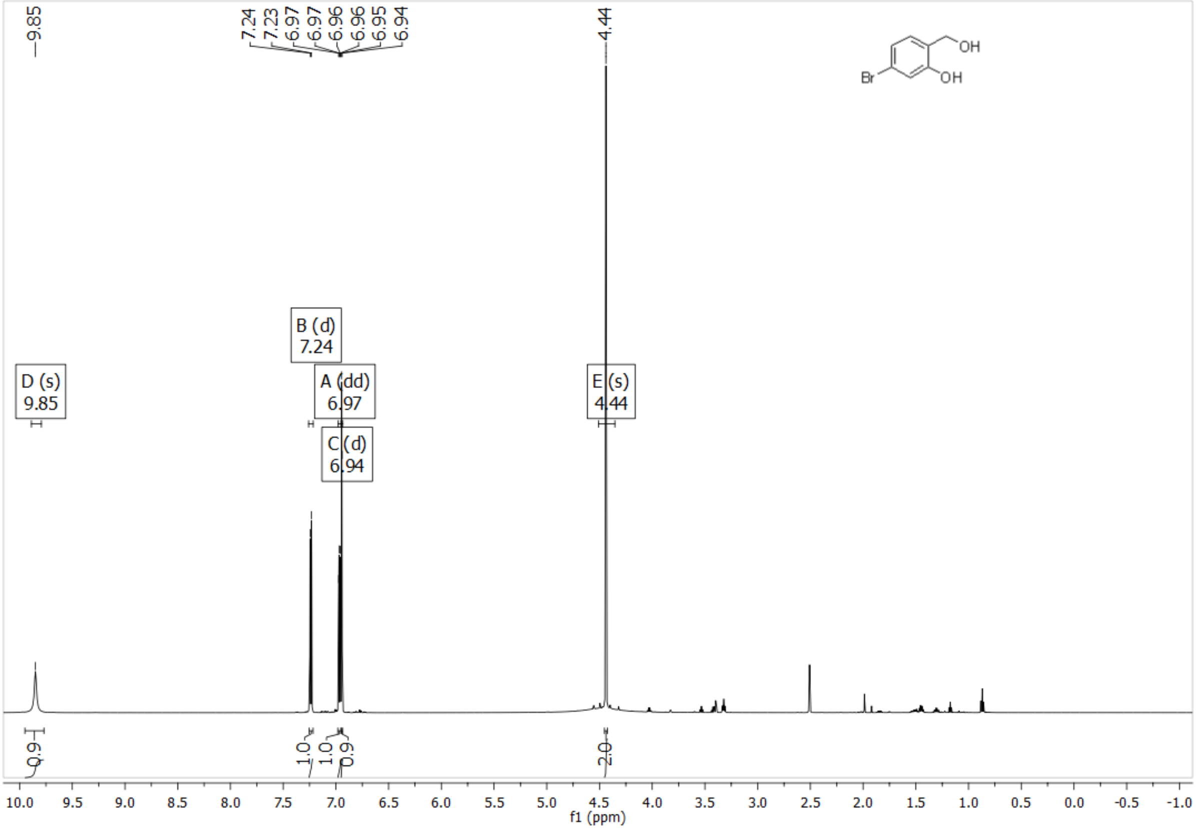

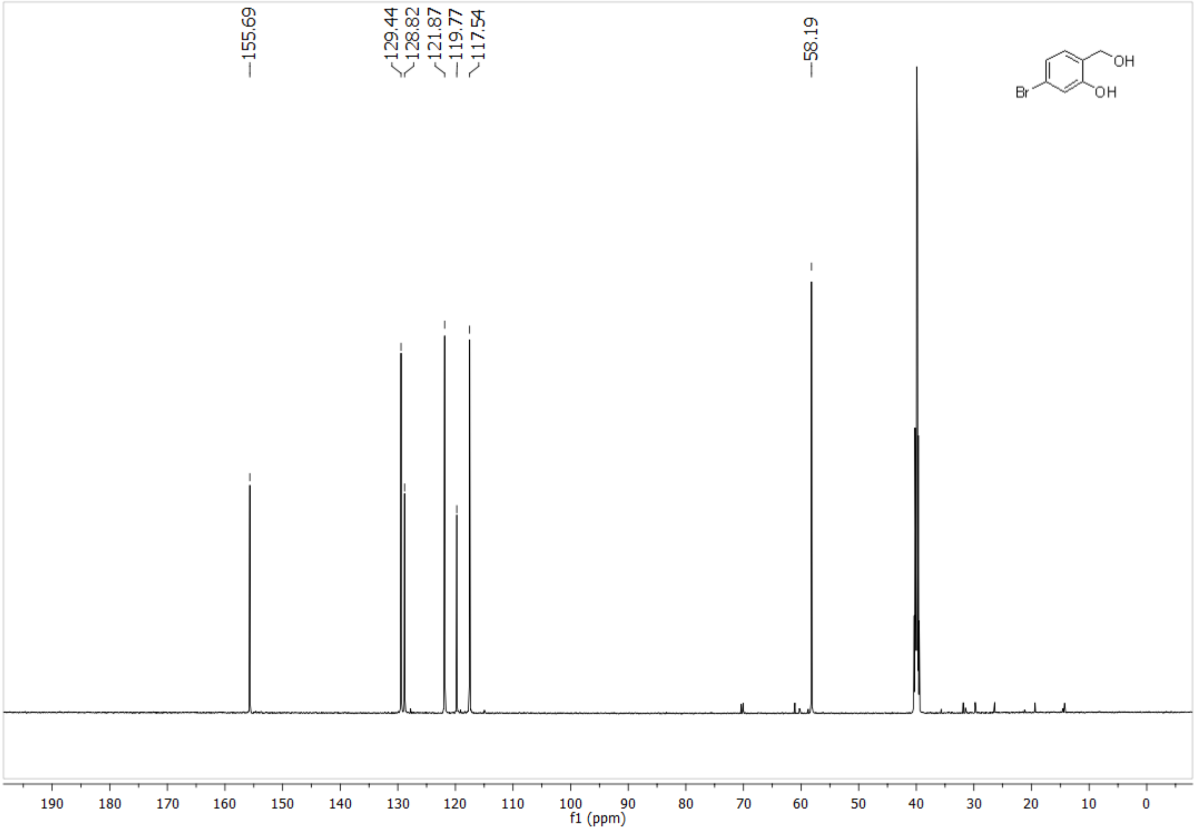

Ib. (4-Bromo-2-ethoxyphenyl)methanol

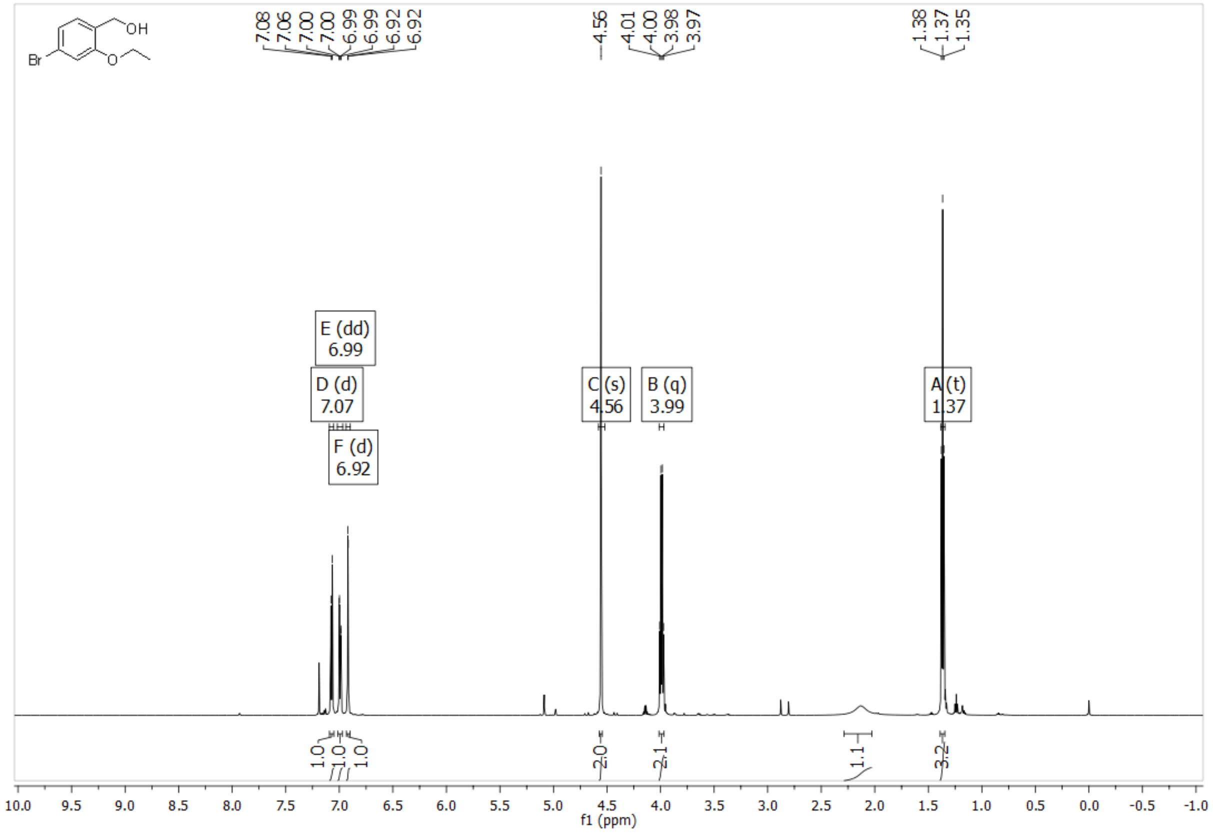

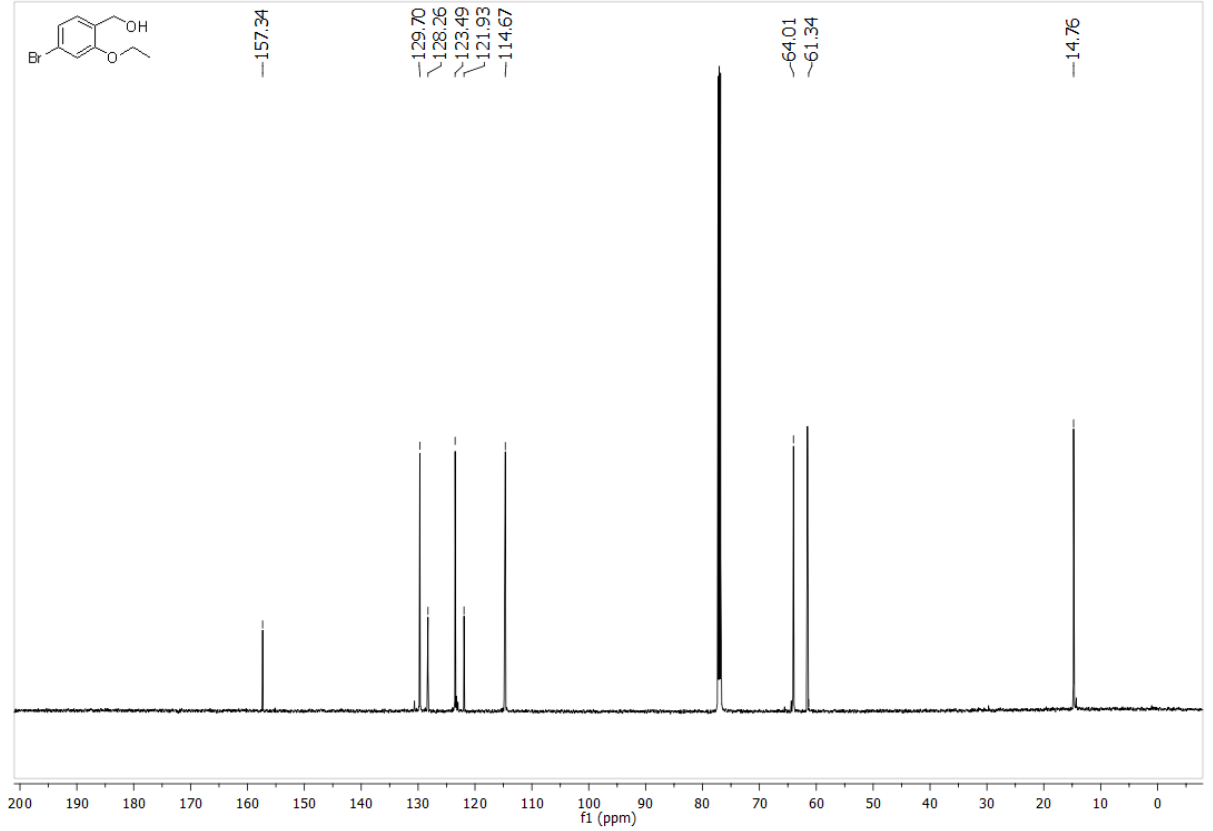

Ic. (4-Bromo-2-isopropoxyphenyl)methanol

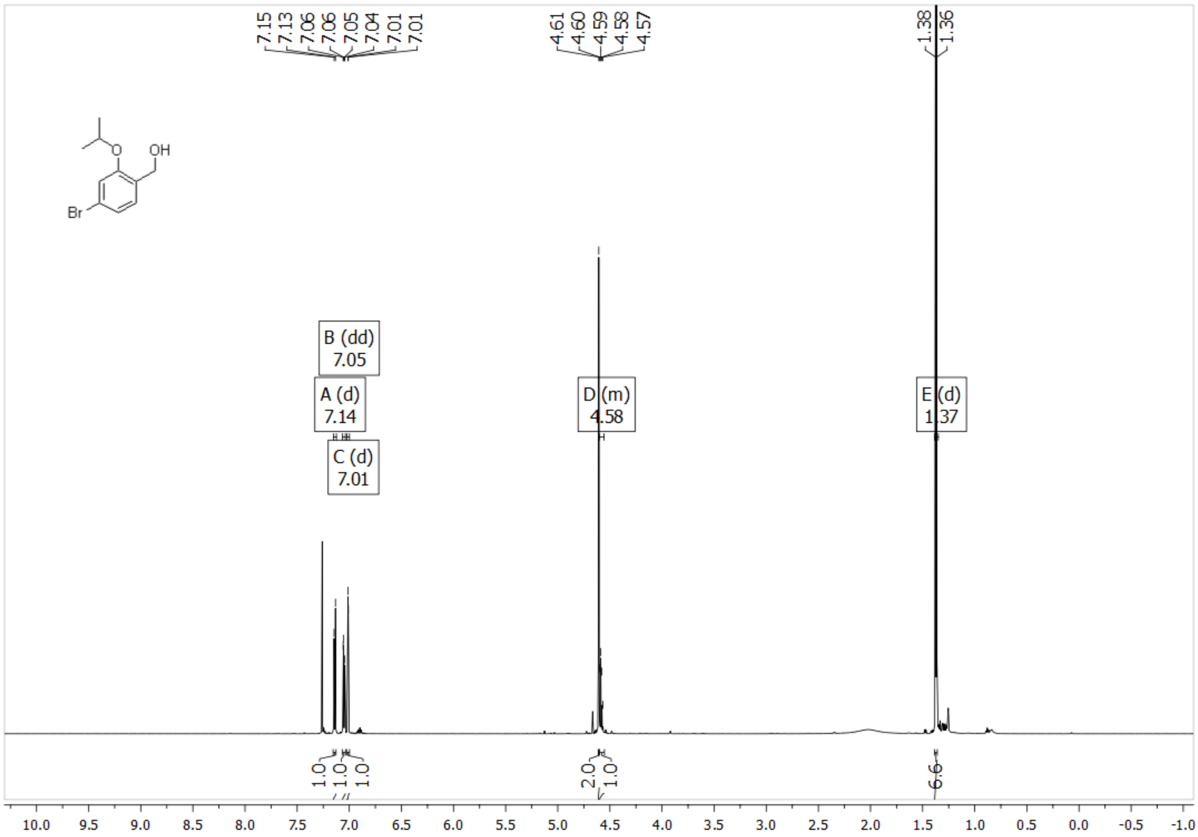

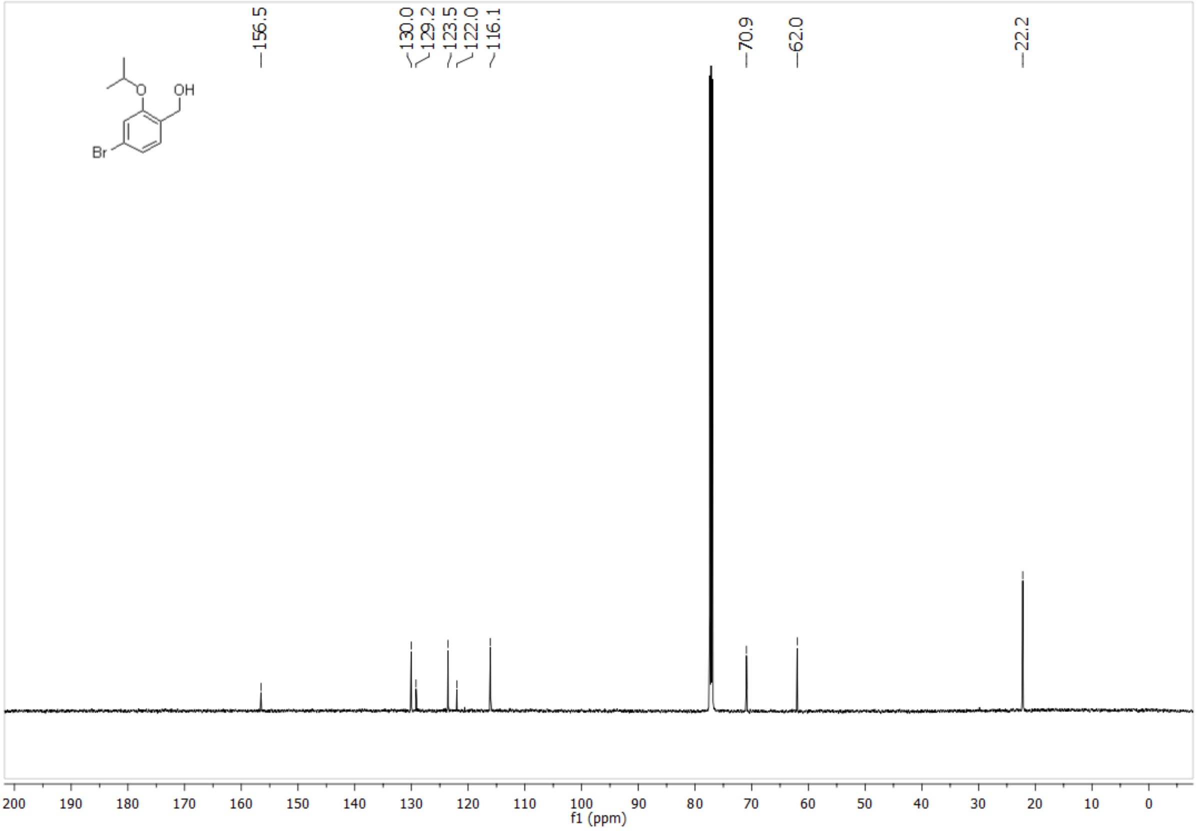

Id. (4-Bromo-2-isopentyloxyphenyl)methanol

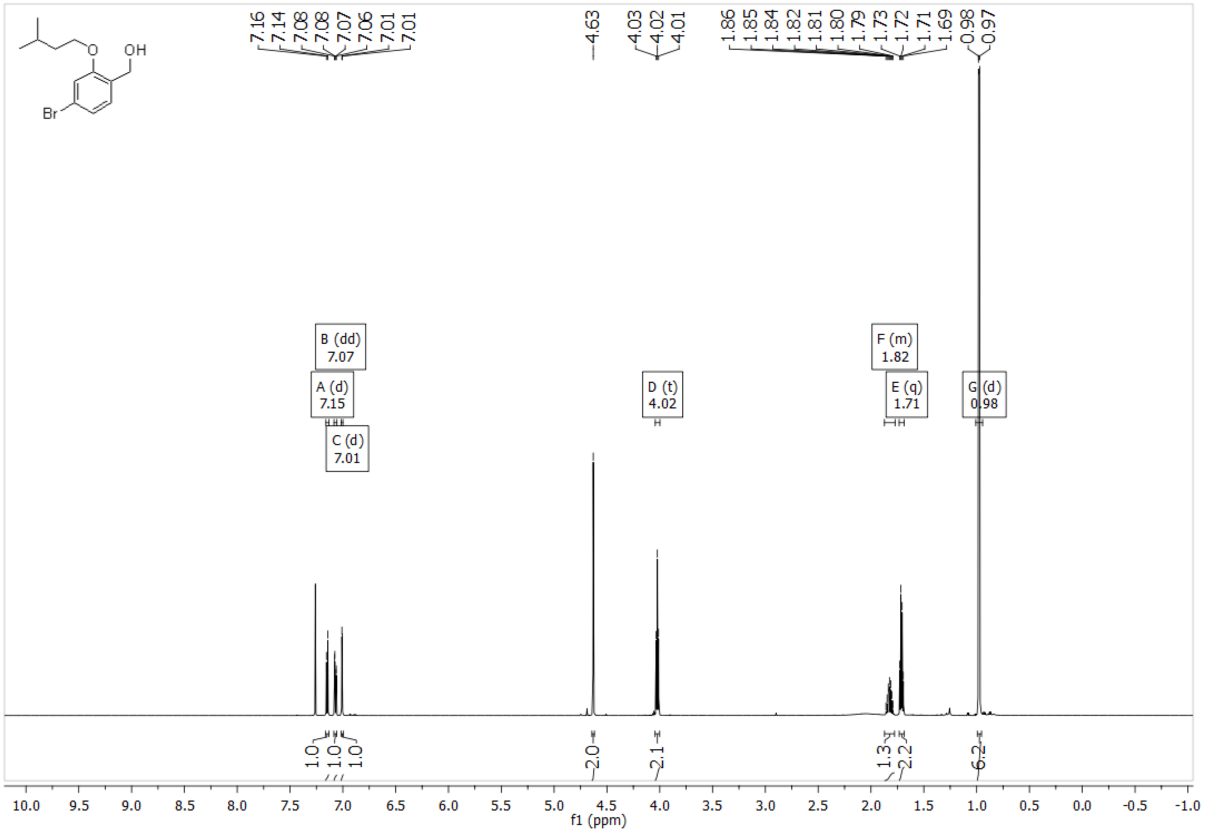

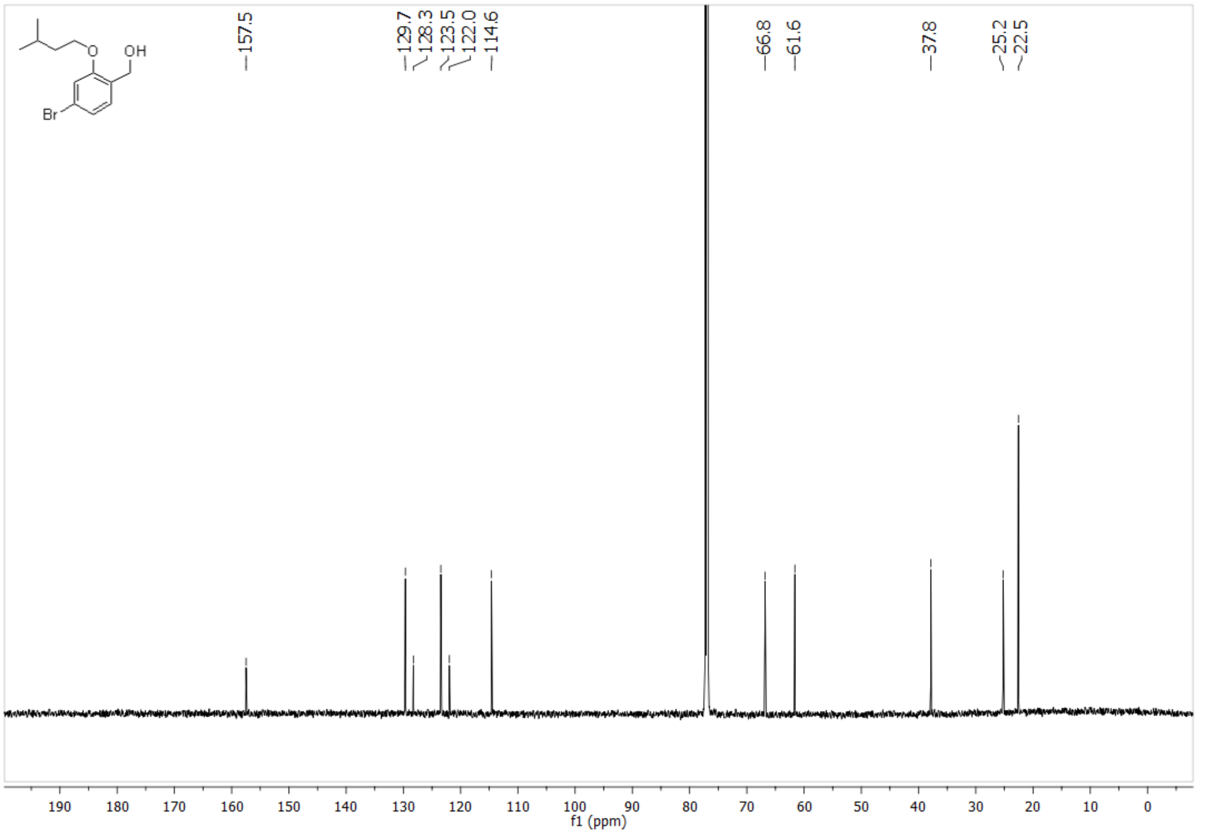

Ie. 2-(5-Bromo-2-(hydroxymethyl)phenoxy)acetonitrile

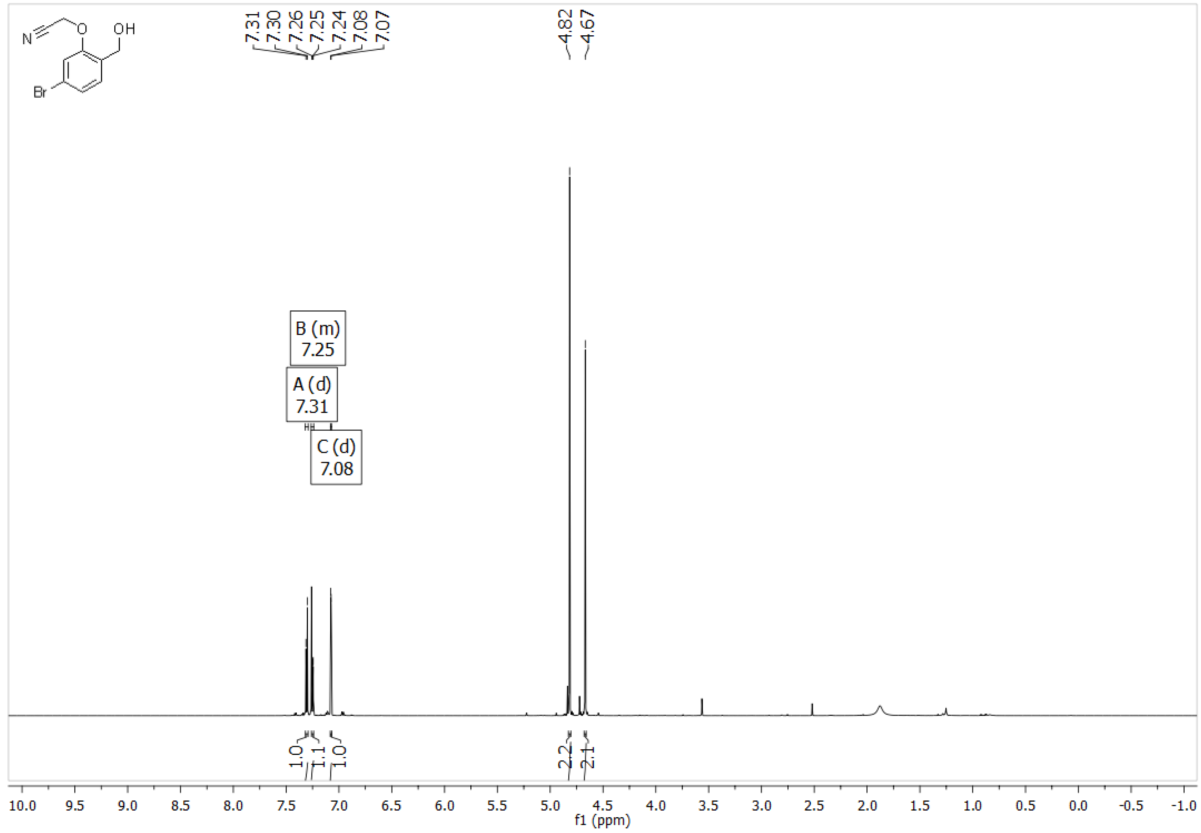

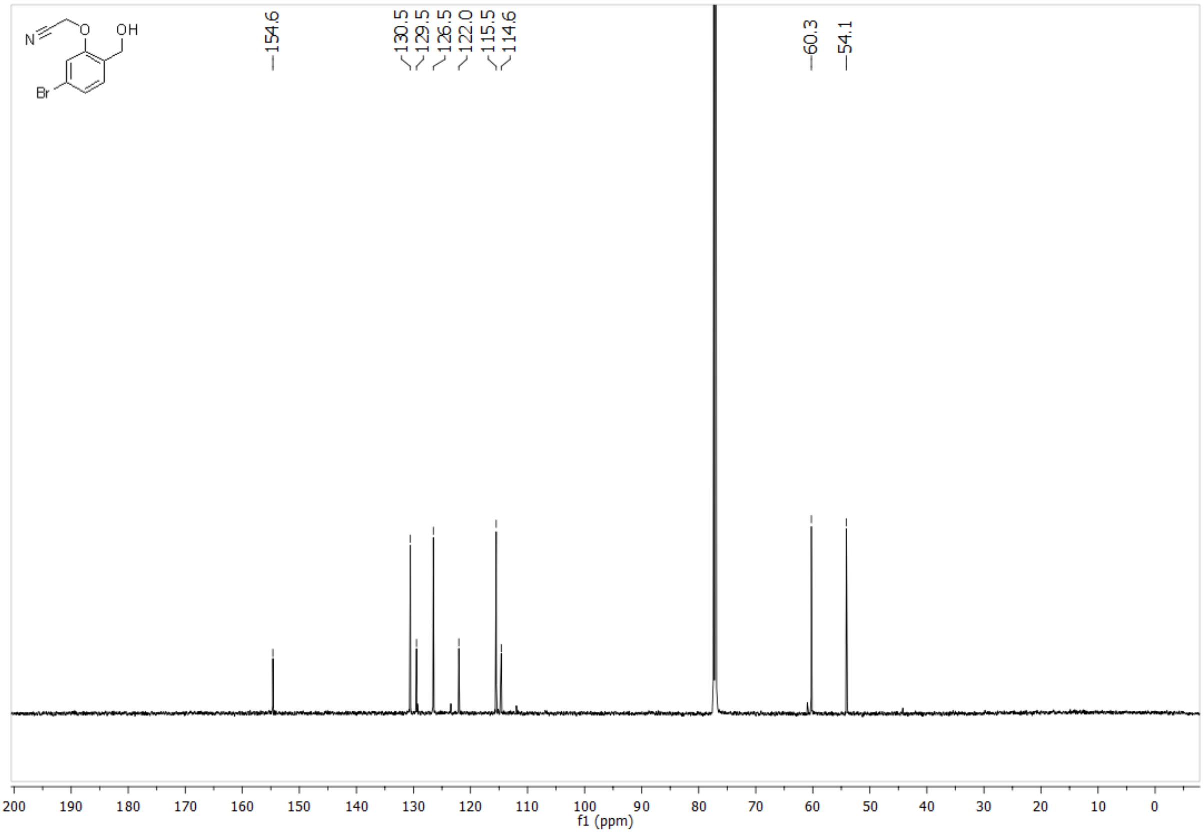

If. (4-Bromo-2-(cyclobutylmethoxy))methanol

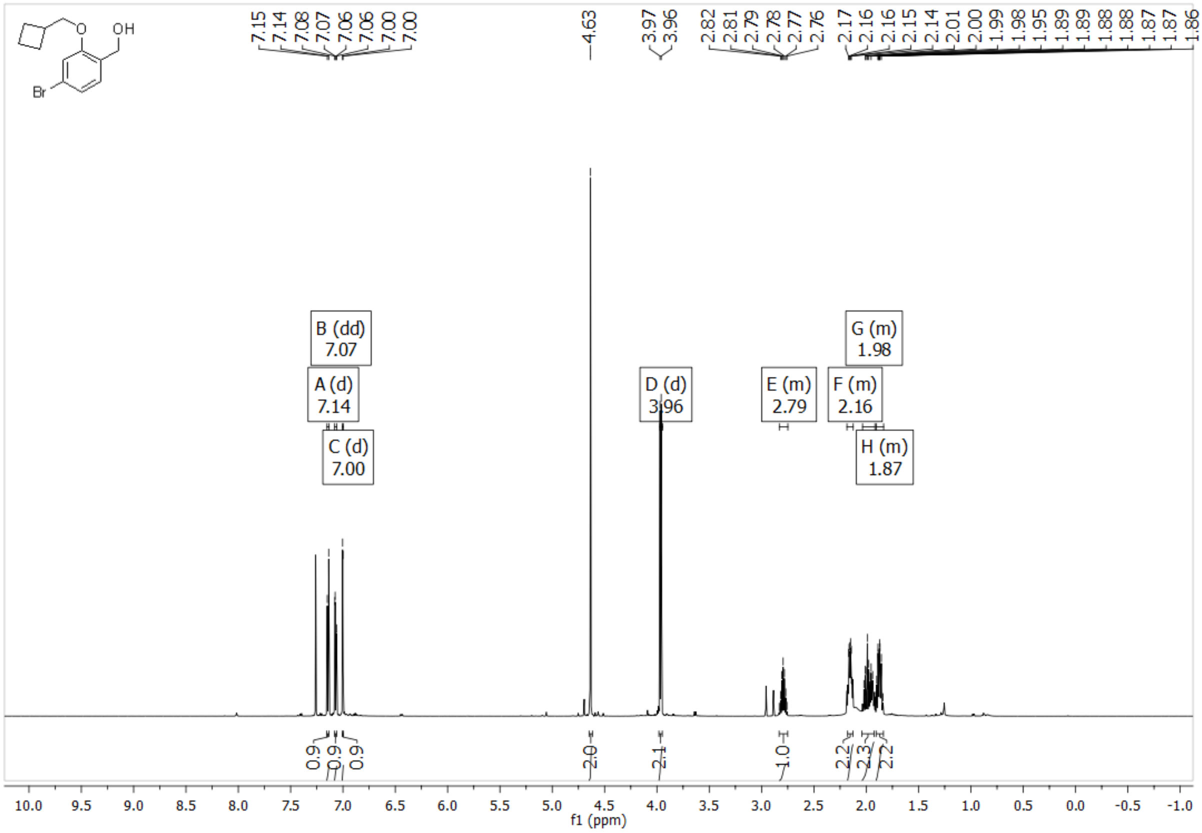

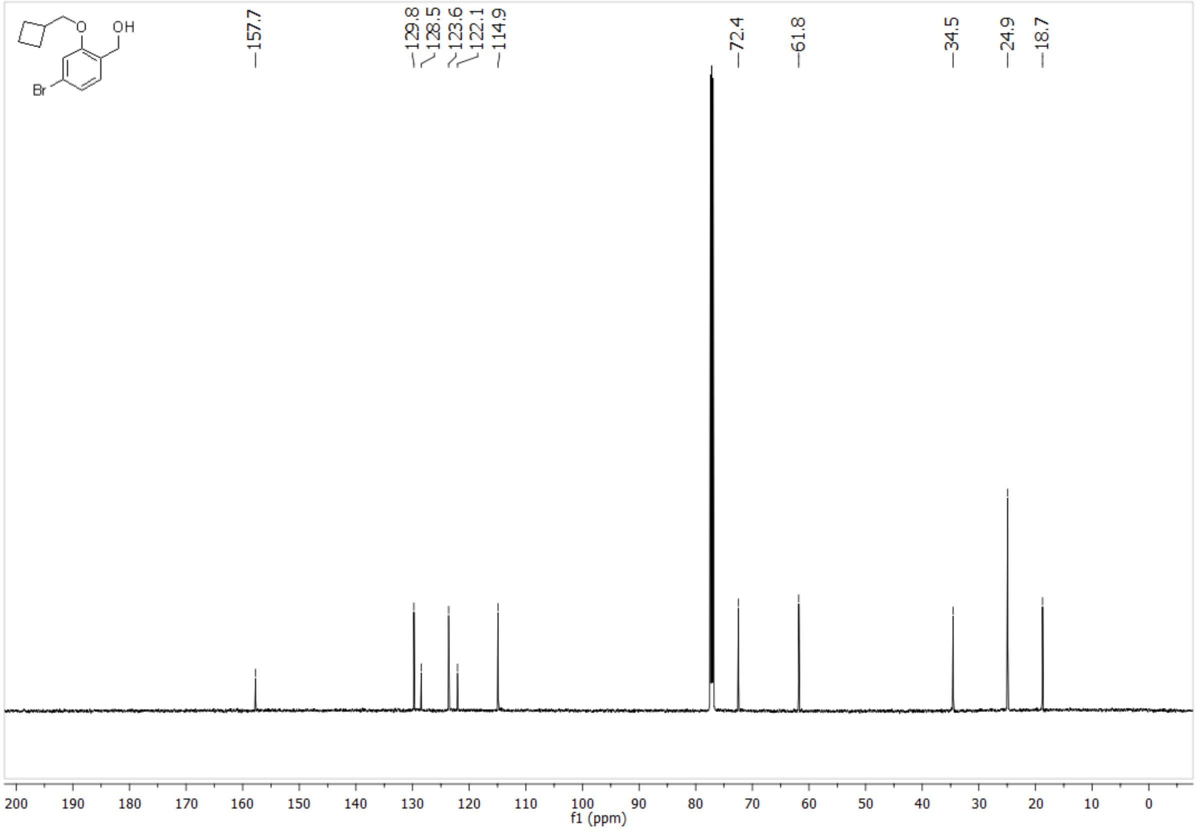

Ig. (4-Bromo-2-(cyclopentylmethoxy))methanol

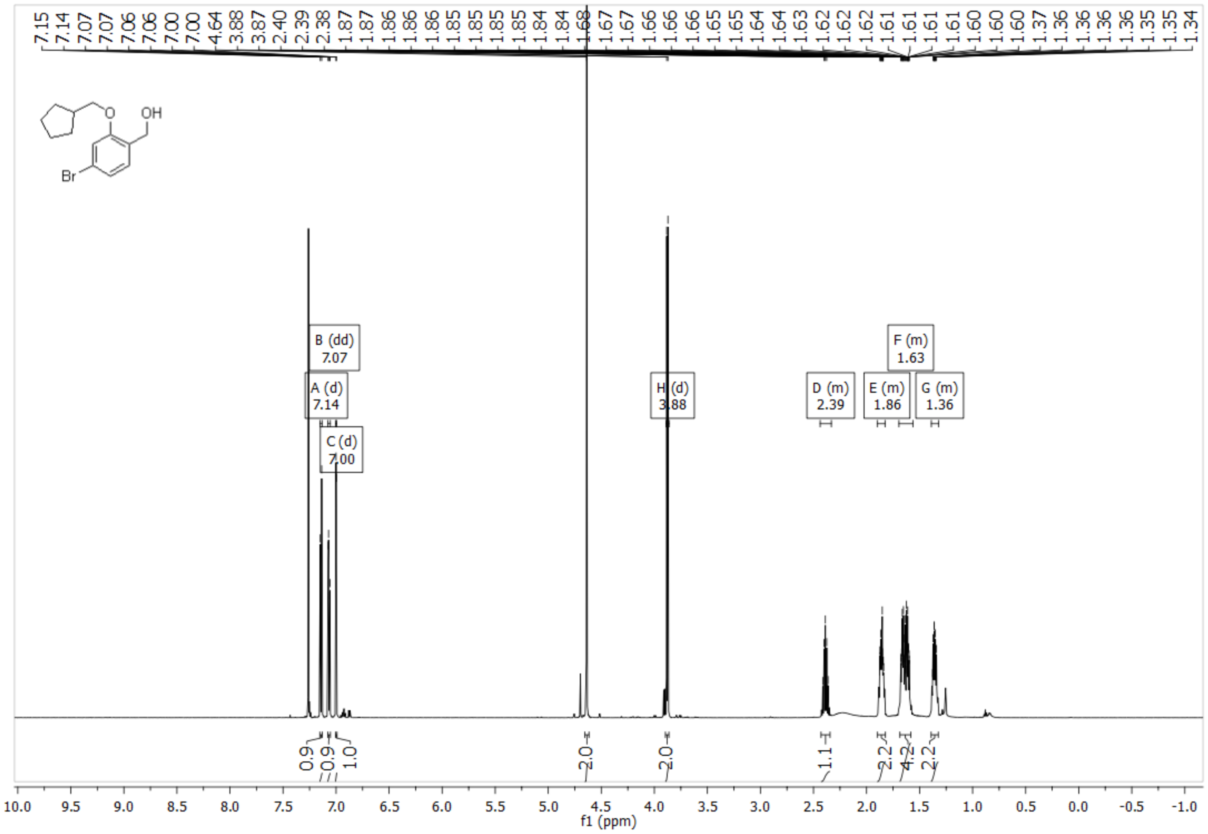

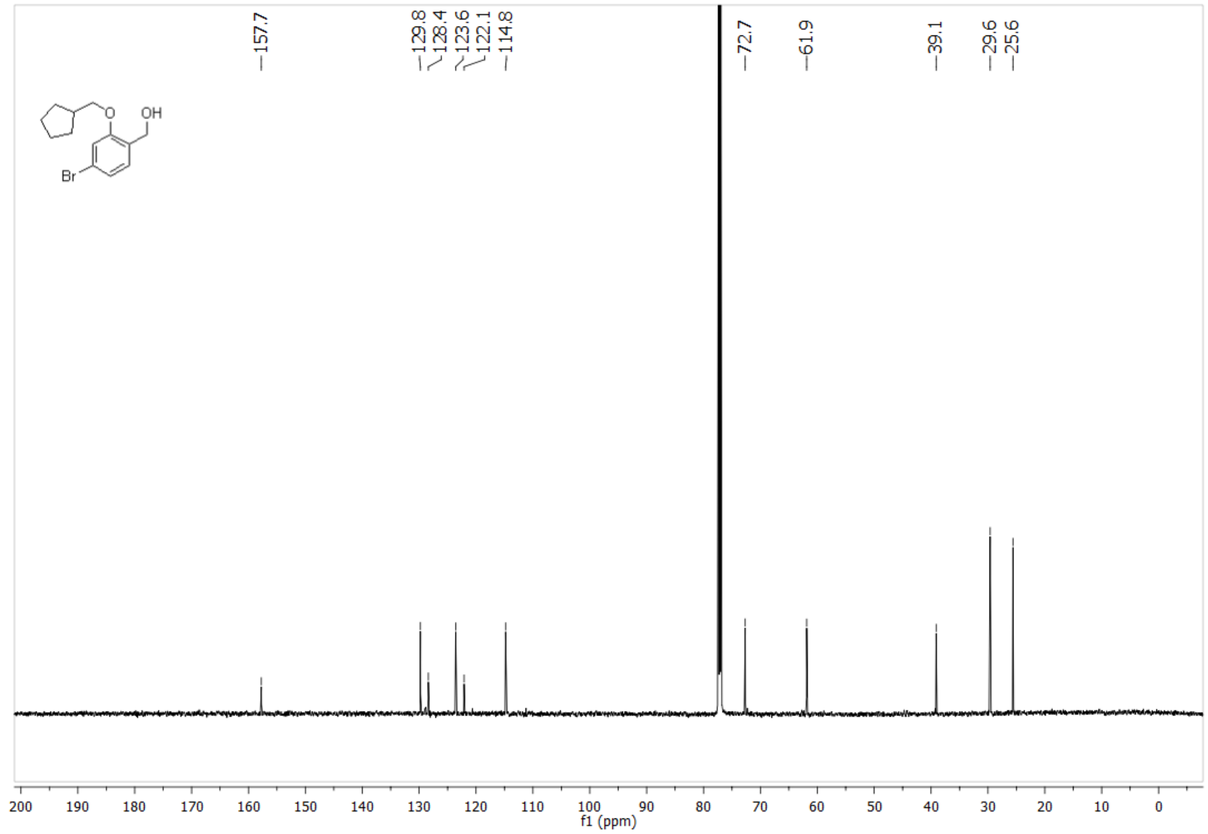

Ih. 3'-((5-bromo-2-(hydroxymethyl)phenoxy)methyl)-[1,1'-biphenyl]-3-carbonitrile

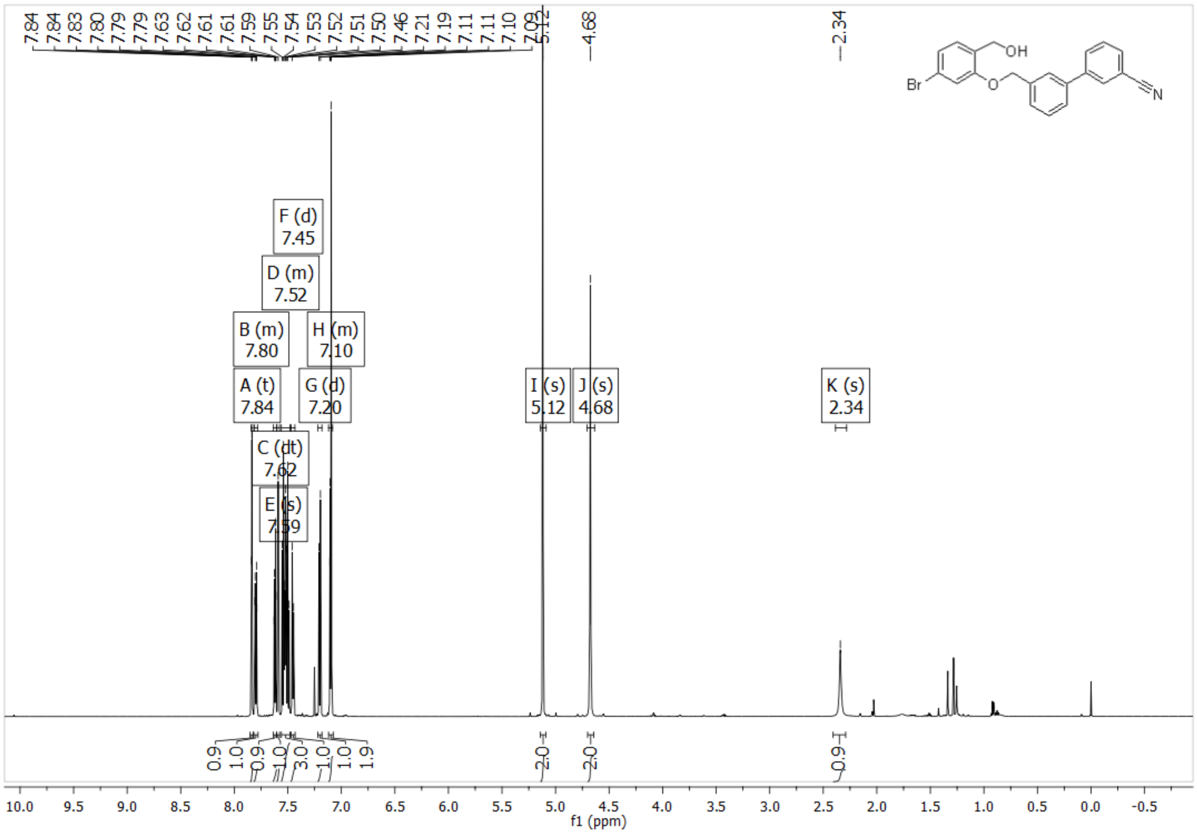

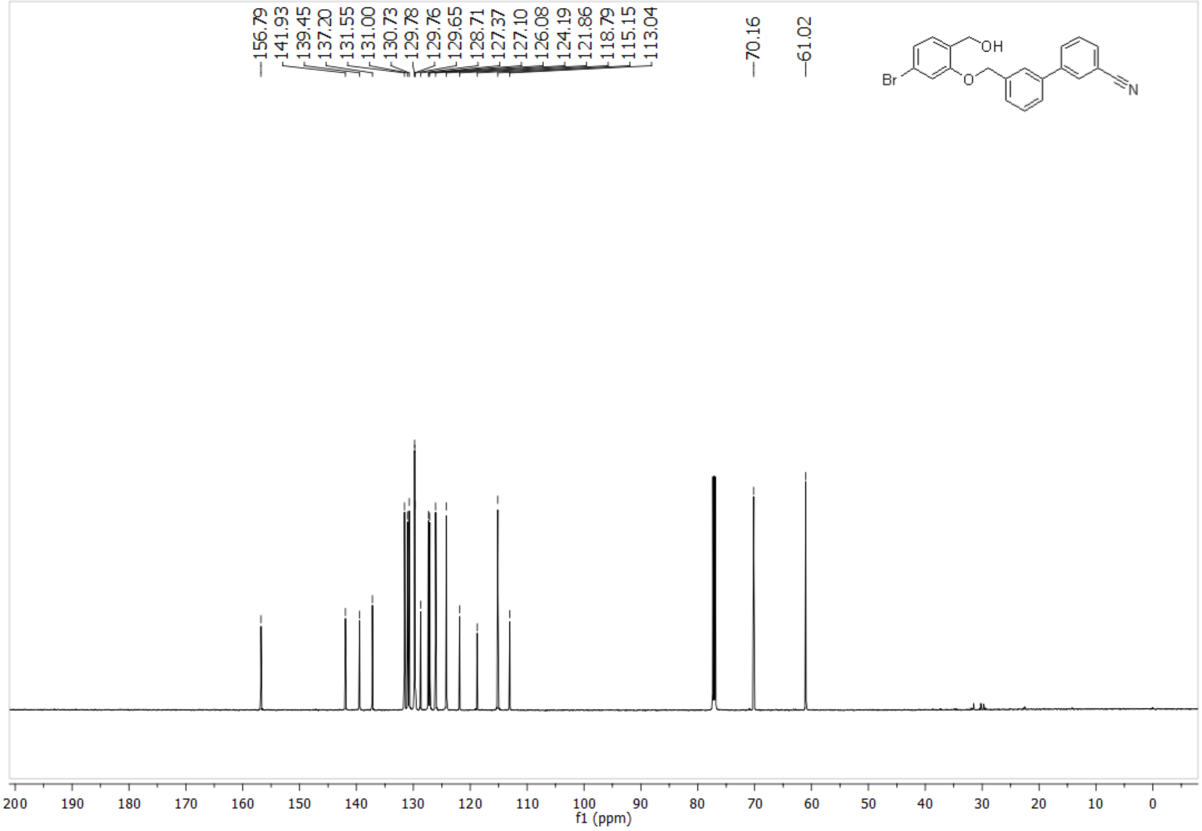

Ii. 3-((5-Bromo-2-(hydroxymethyl)phenoxy)methyl)benzonitrile

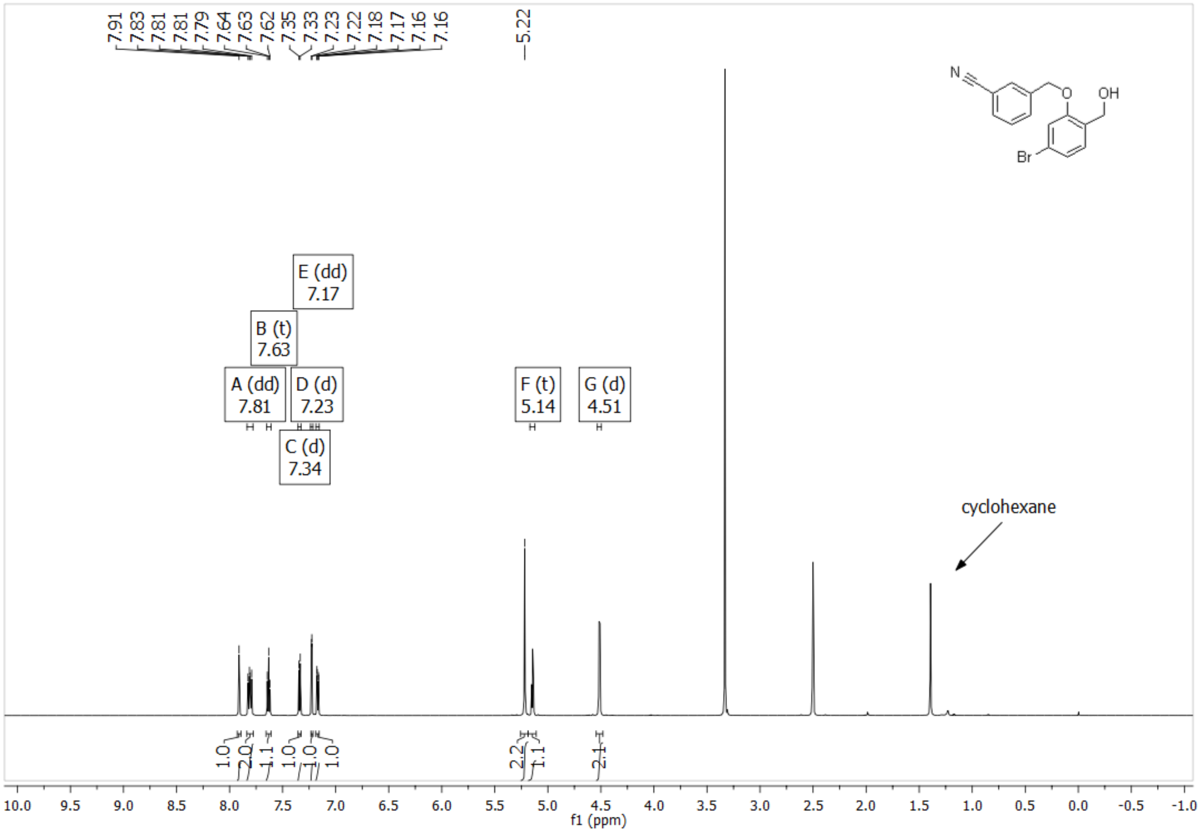

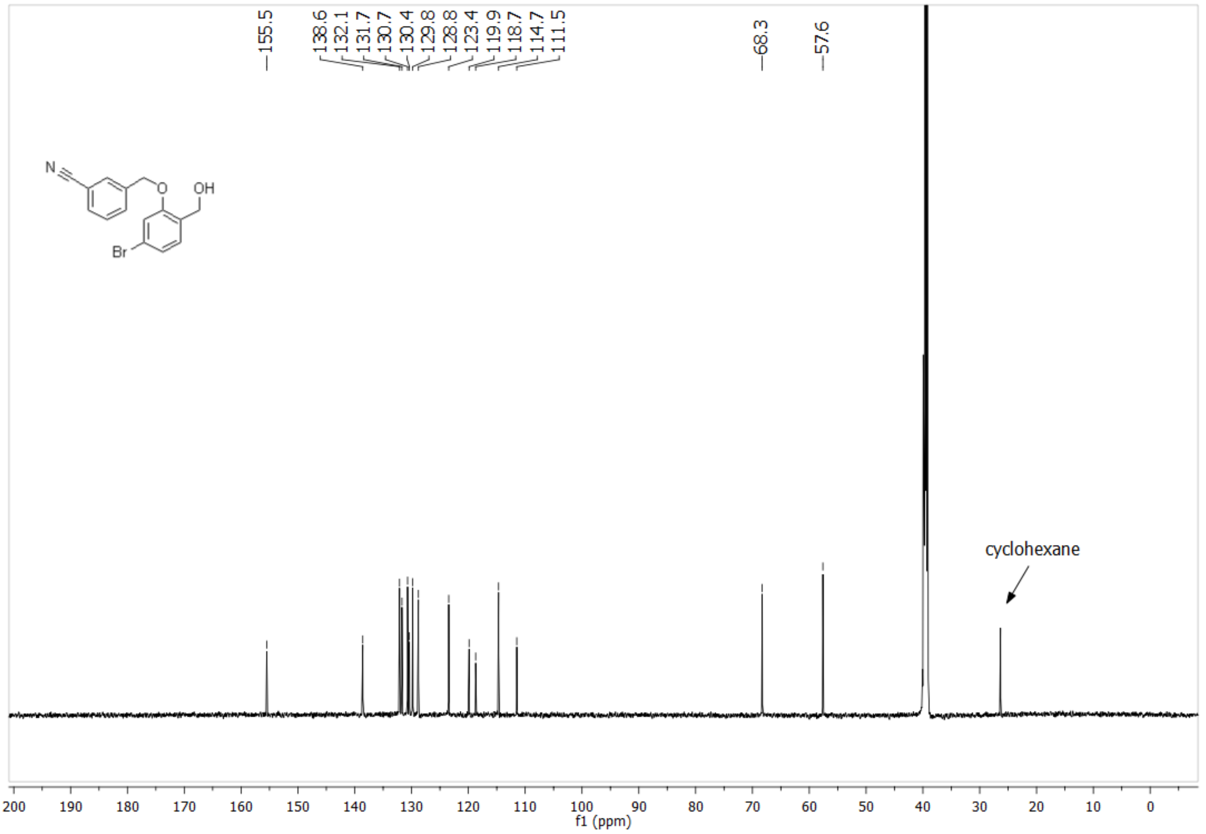

3a. (2-Methoxy-4-(4,4,5,5-tetramethyl-1,3,2-dioxaborolan-2-yl)phenyl)methanol

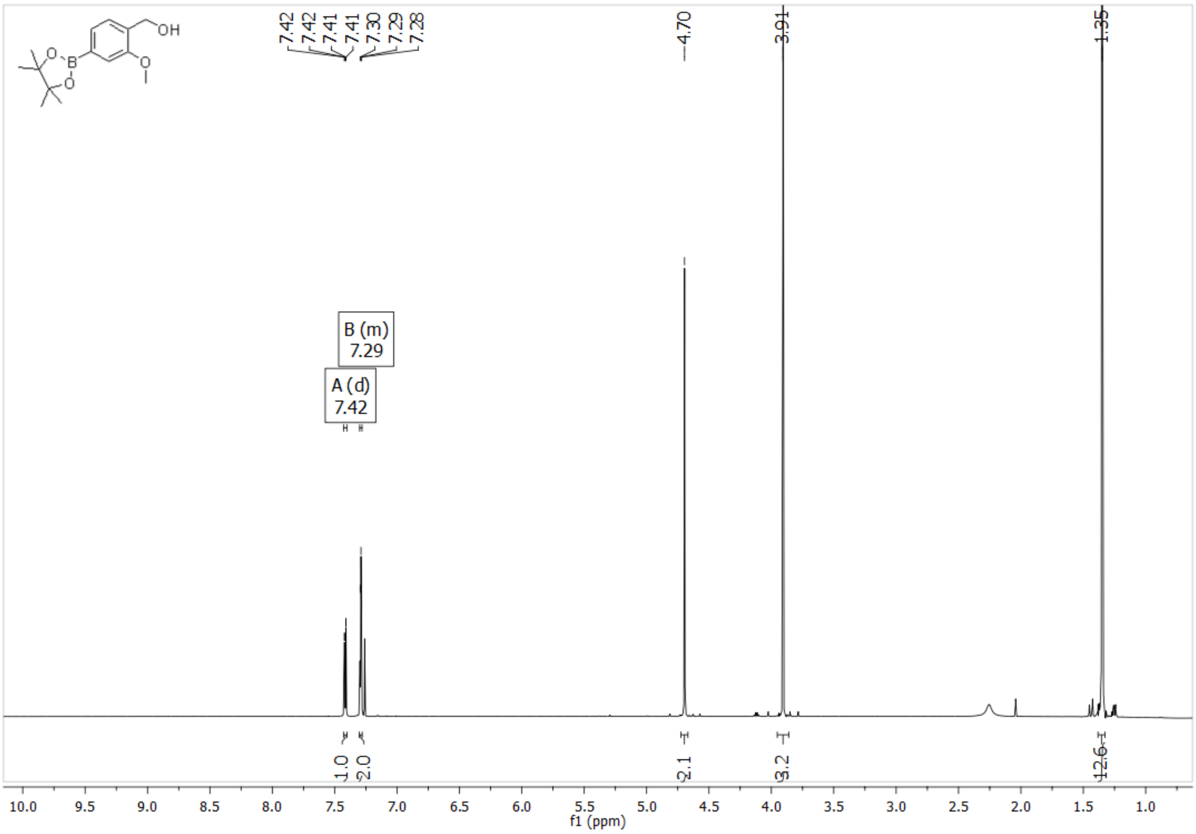

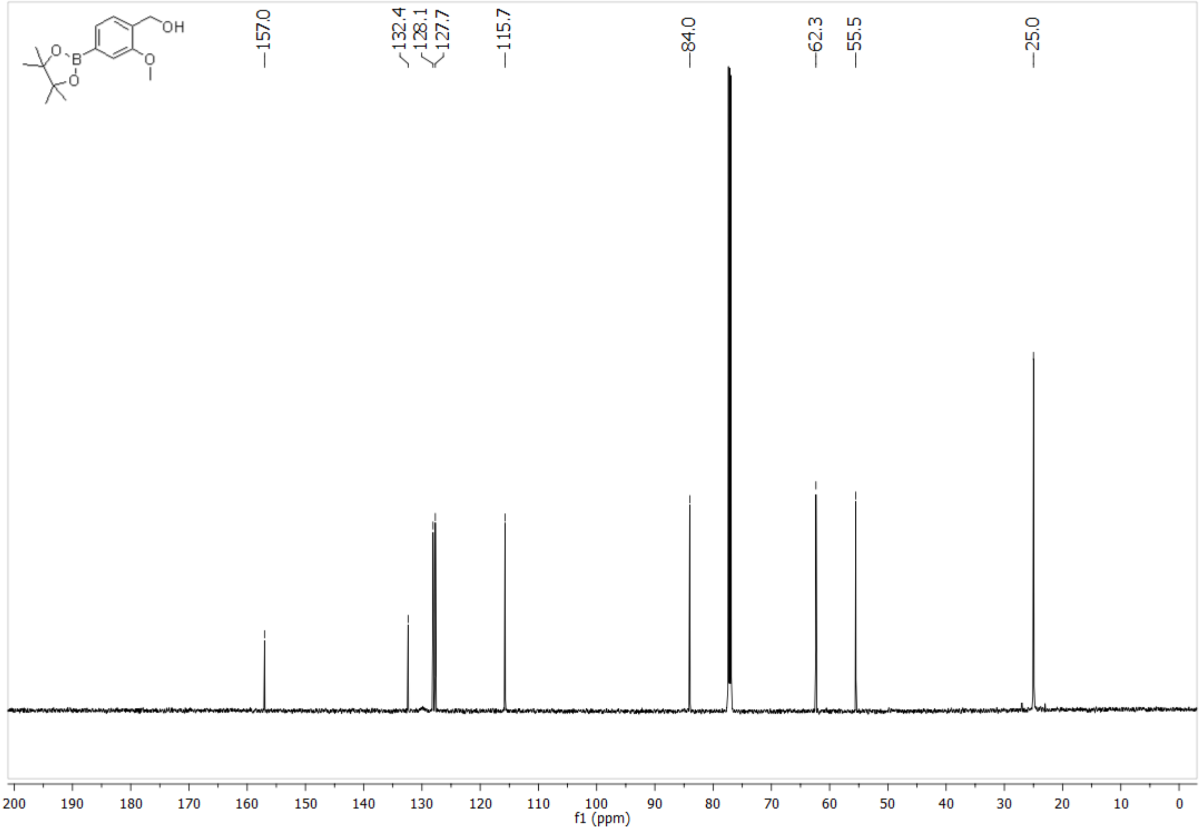

3b. (2-Ethoxy-4-(4,4,5,5-tetramethyl-1,3,2-dioxaborolan-2-yl)phenyl)methanol

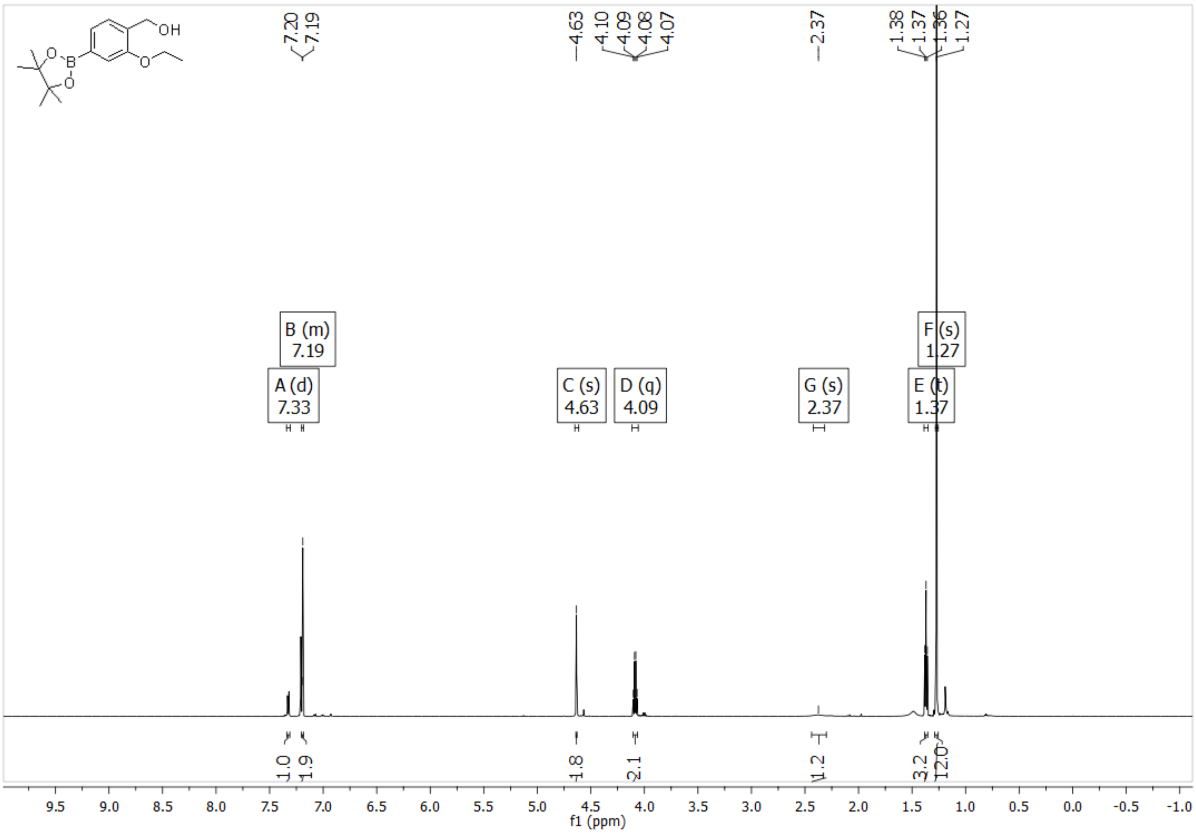

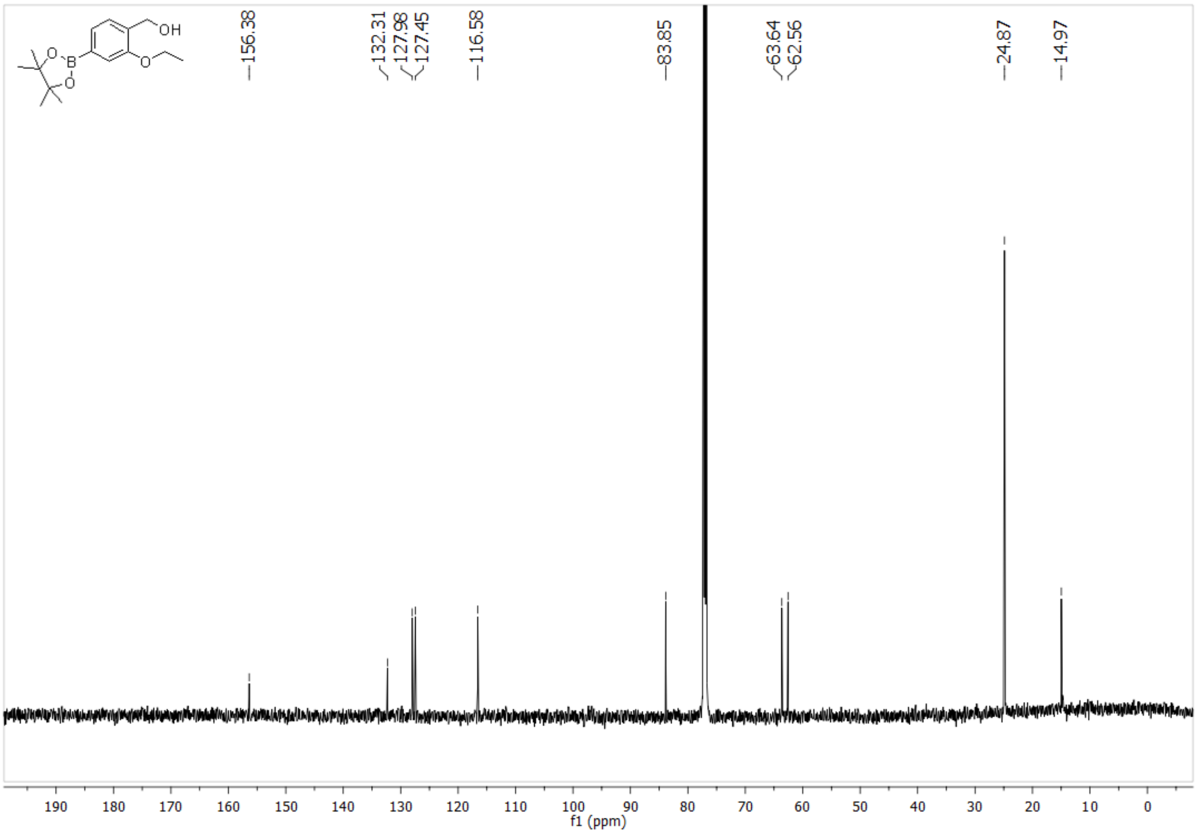

3c. (2-Isopropoxy-4-(4,4,5,5-tetramethyl-1,3,2-dioxaborolan-2-yl)phenyl)methanol

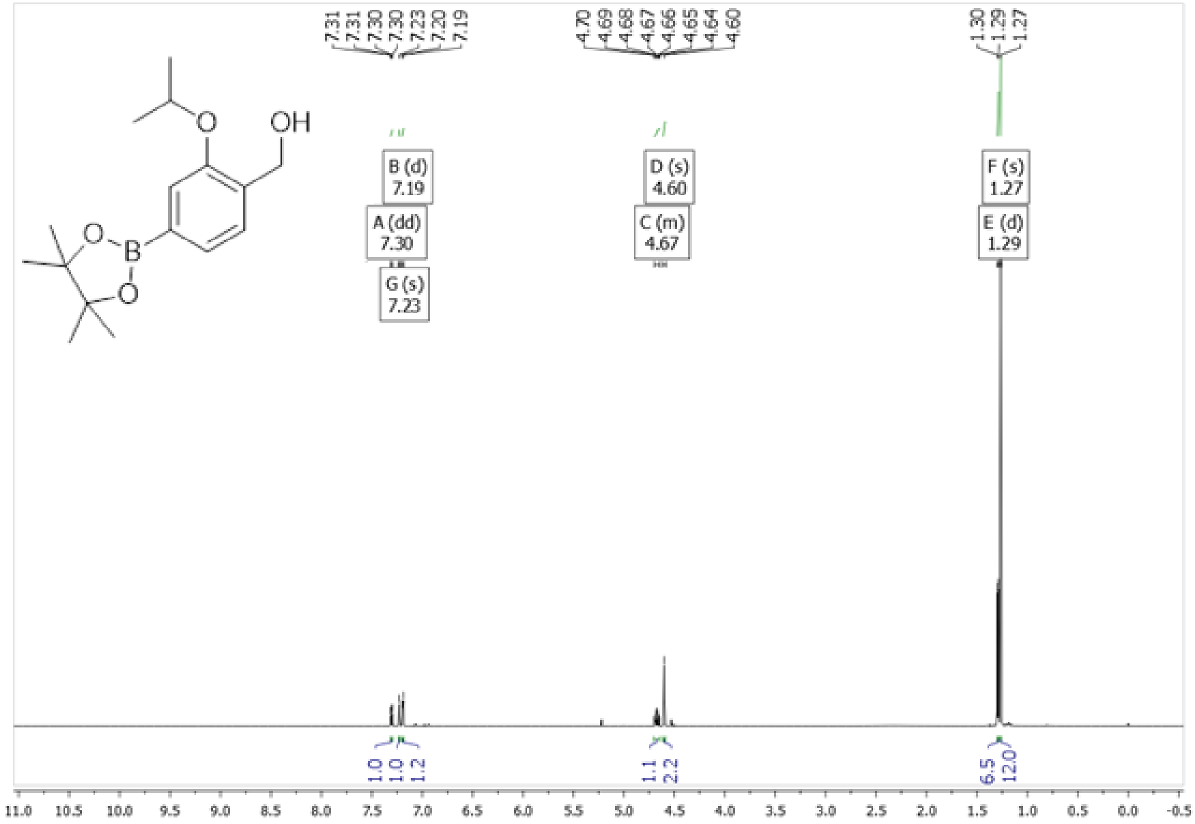

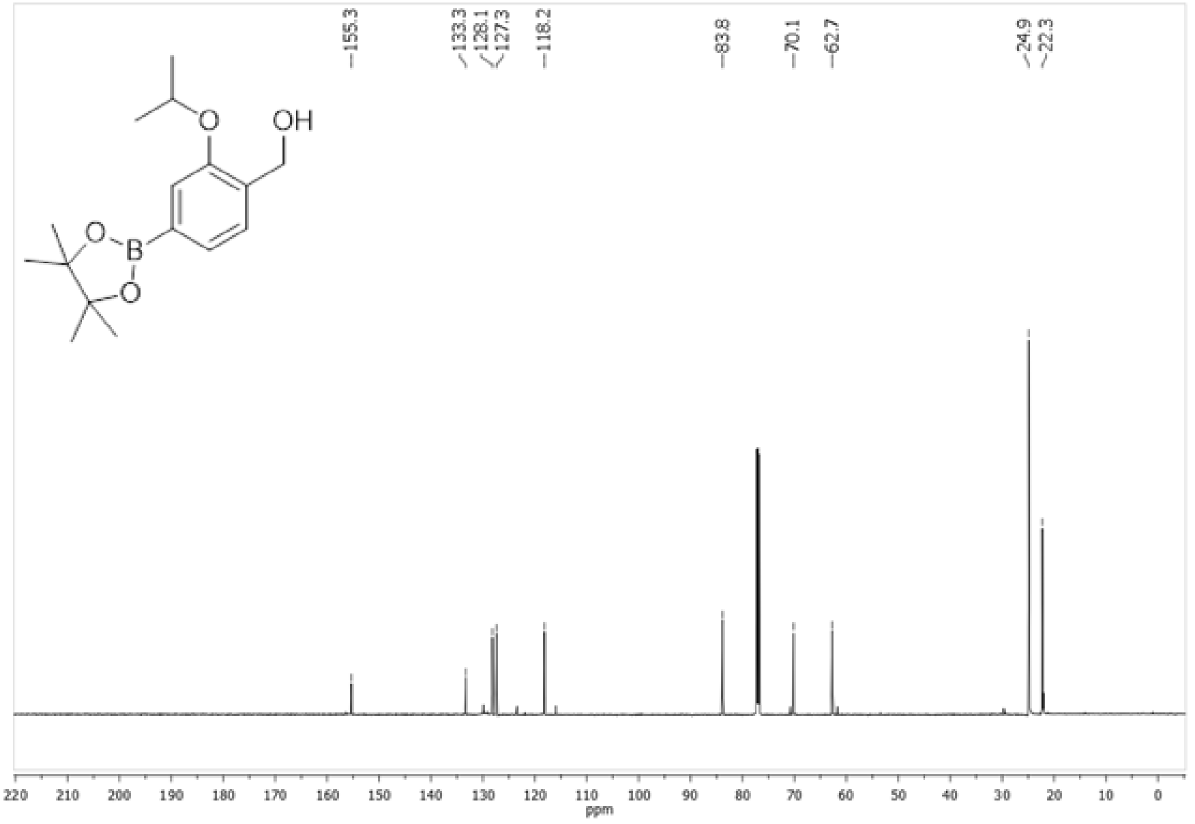

3d. (2-Isopentoxy-4-(4,4,5,5-tetramethyl-1,3,2-dioxaborolan-2-yl)phenyl)methanol

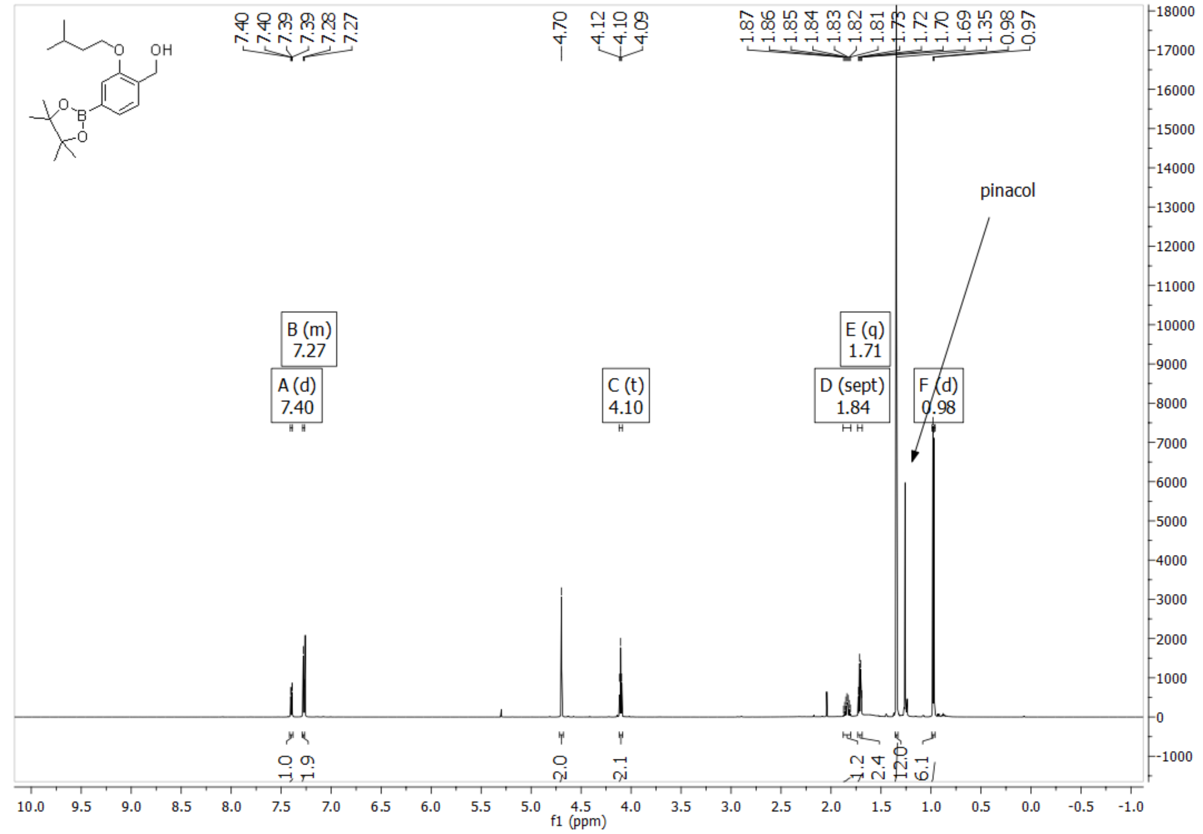

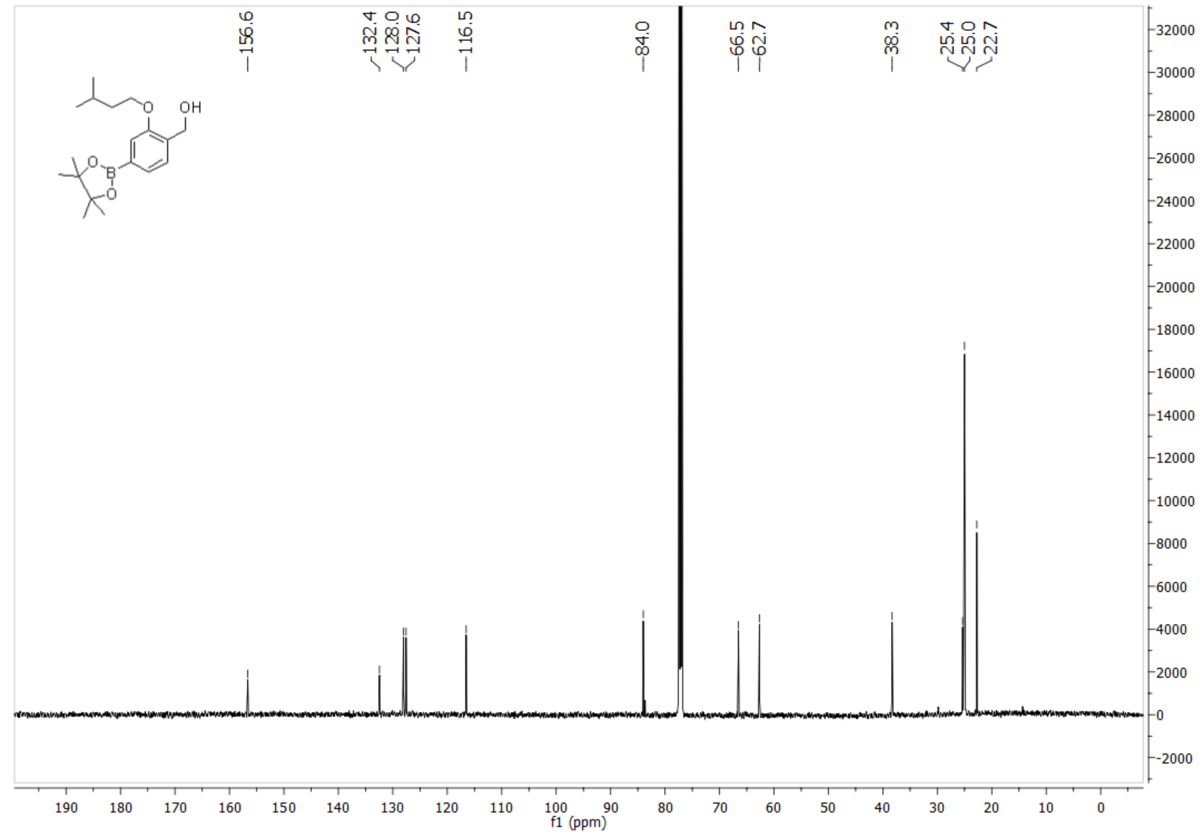

3e. 2-(2-Hydroxymethyl)-5-(4,4,5,5-tetramethyl-1,3,2-dioxaborolan-2-yl)phenoxy)acetonitrile

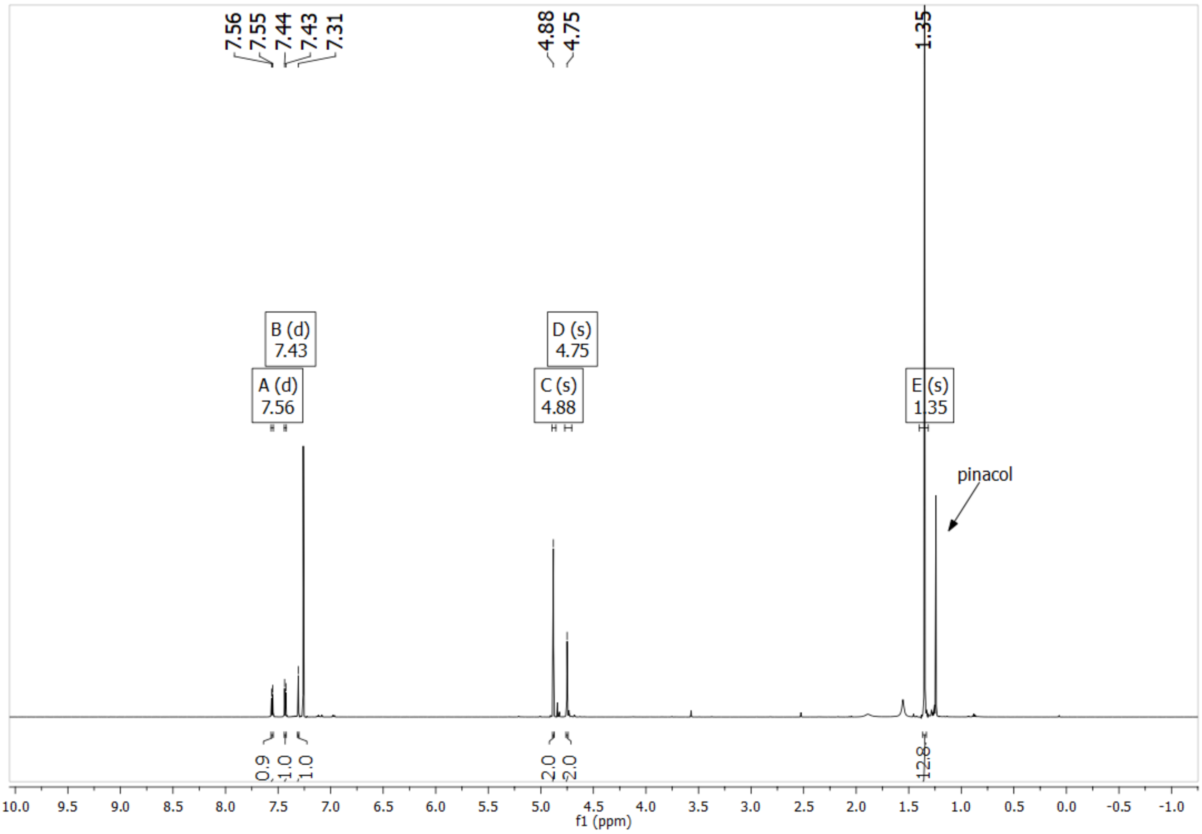

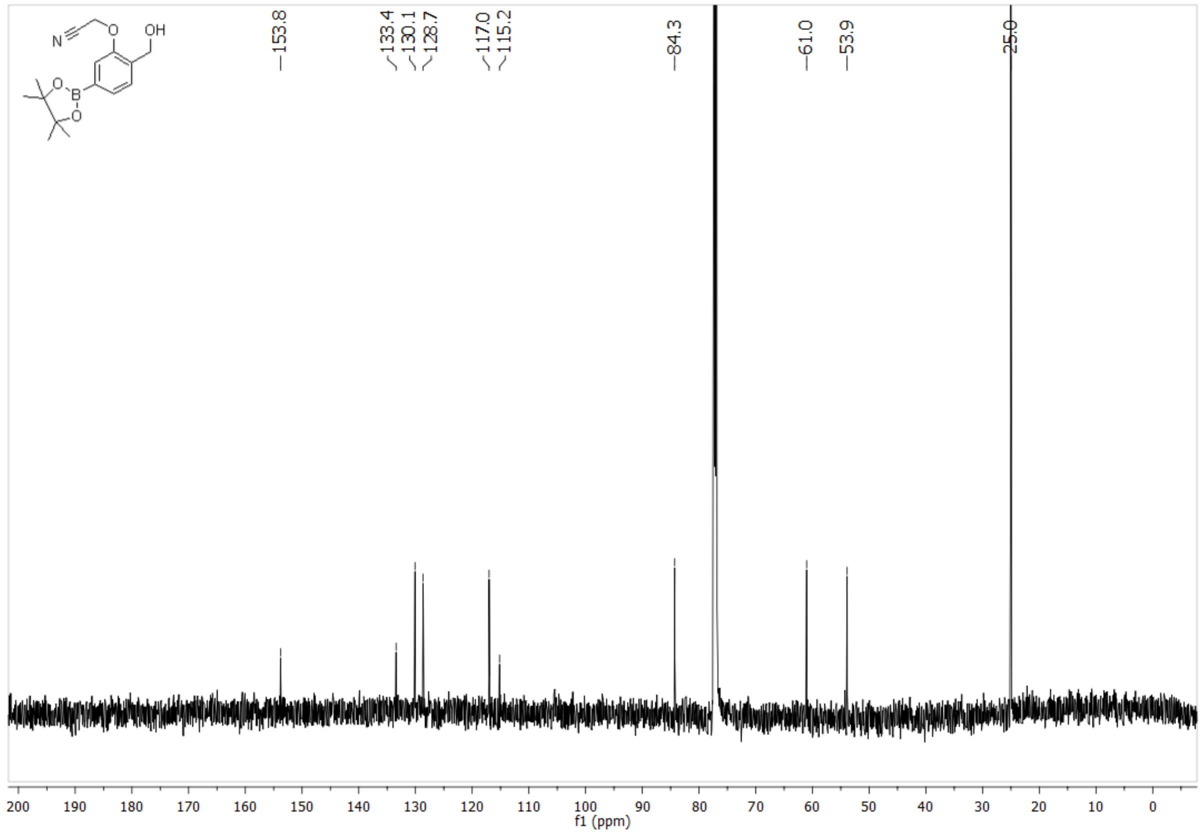

3f. (2-(Cyclobutylmethoxy)-4-(4,4,5,5-tetramethyl-1,3,2-dioxaborolan-2-yl)phenyl)methanol

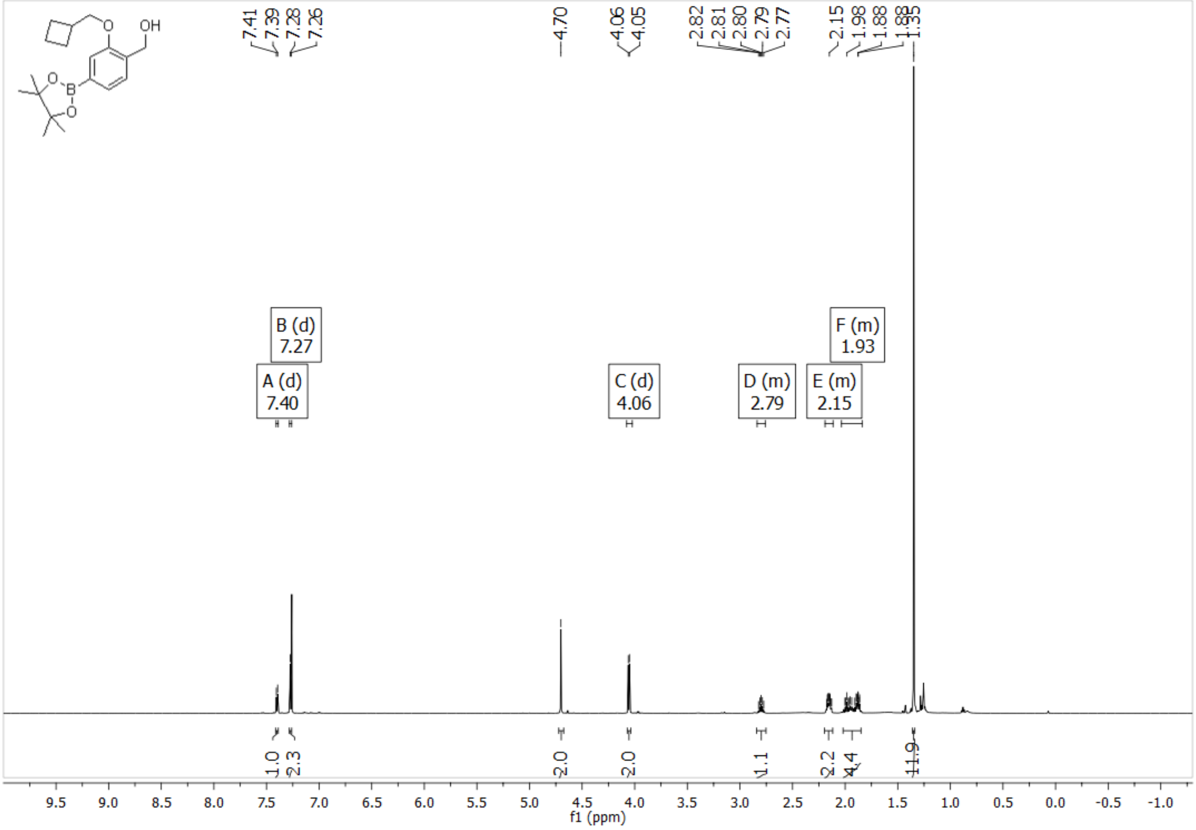

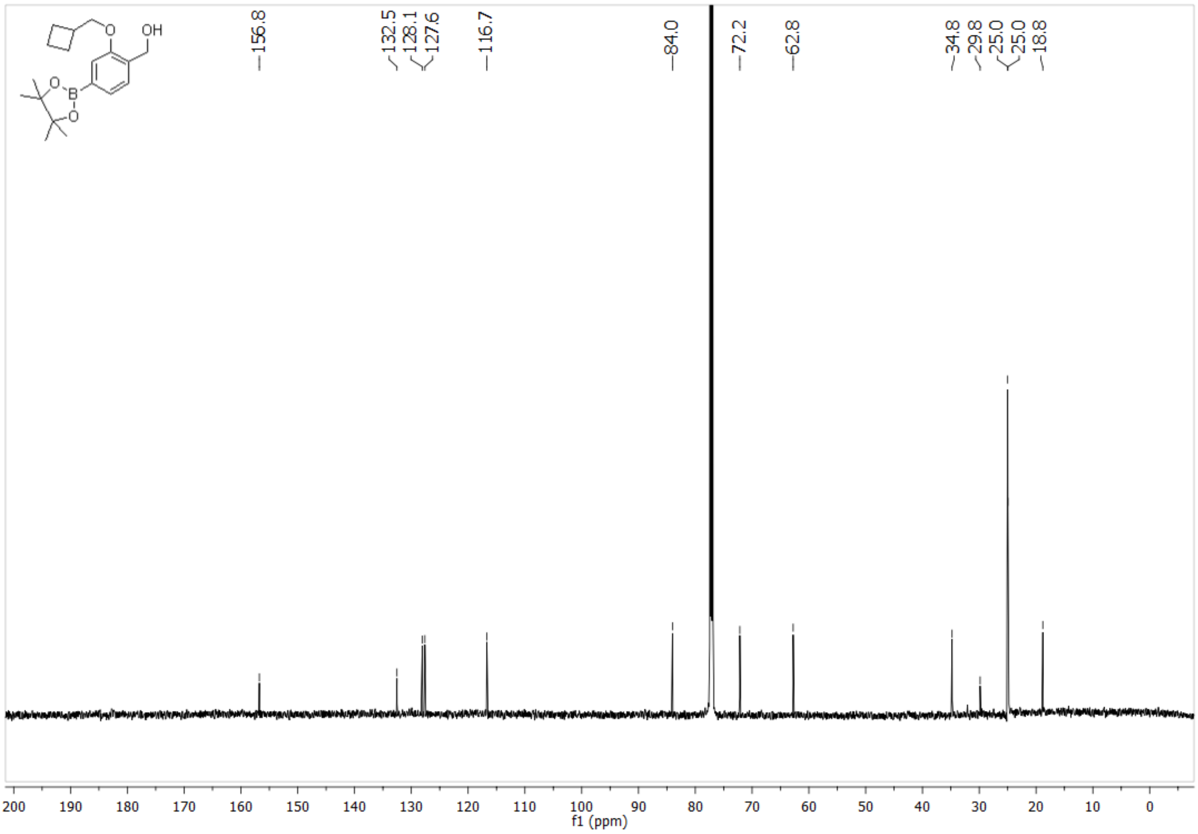

3g. (2-(Cyclopentylmethoxy)-4-(4,4,5,5-tetramethyl-1,3,2-dioxaborolan-2-yl)phenyl)methanol

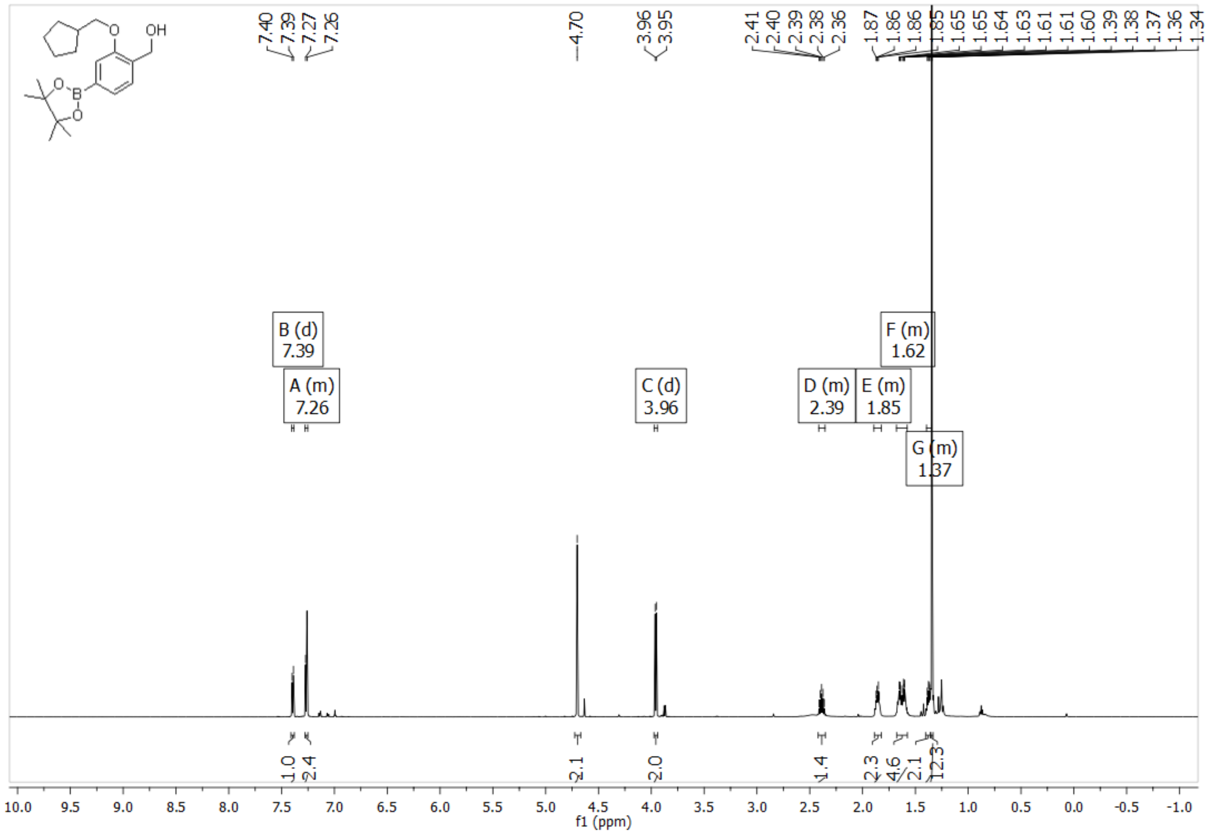

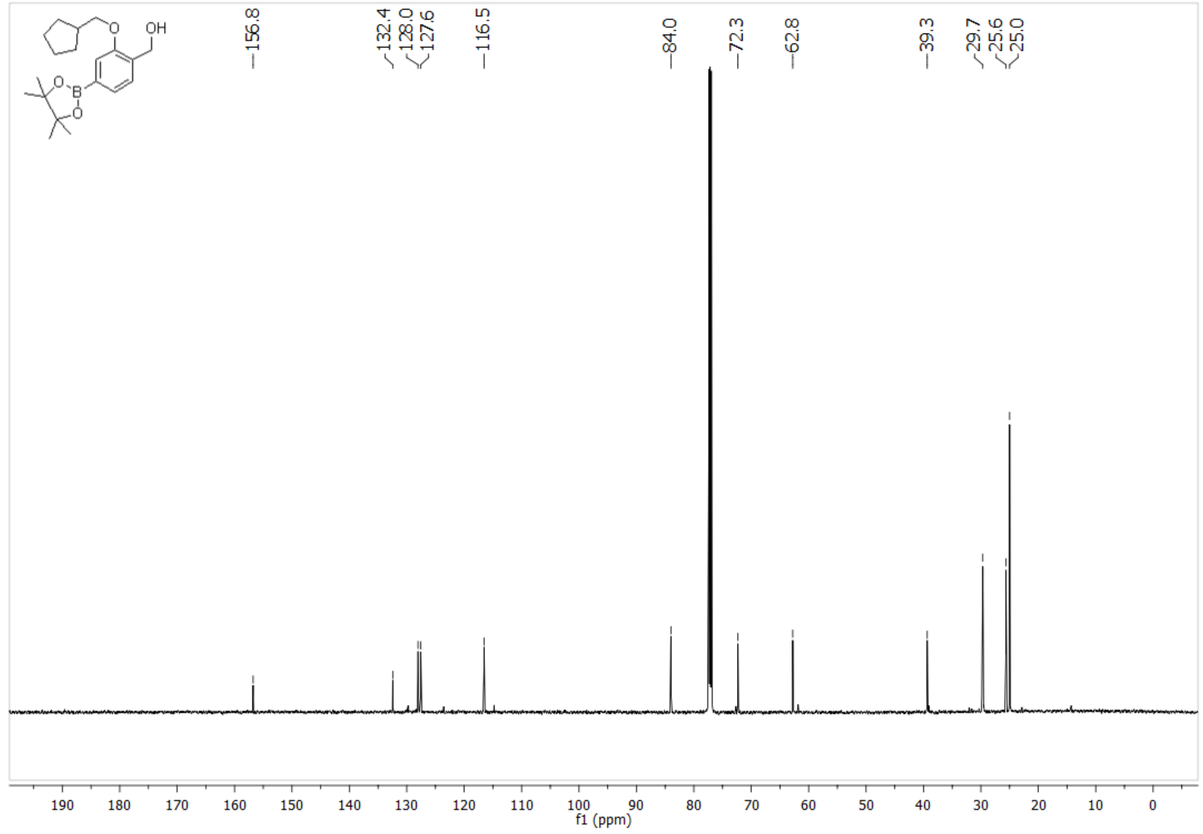

3h. 3'-((2-(Hydroxymethyl)-5-(4,4,5,5-tetramethyl-1,3,2-dioxaborolan-2-yl)phenoxy)methyl)-[1,1'-biphenyl]-3-carbonitrile

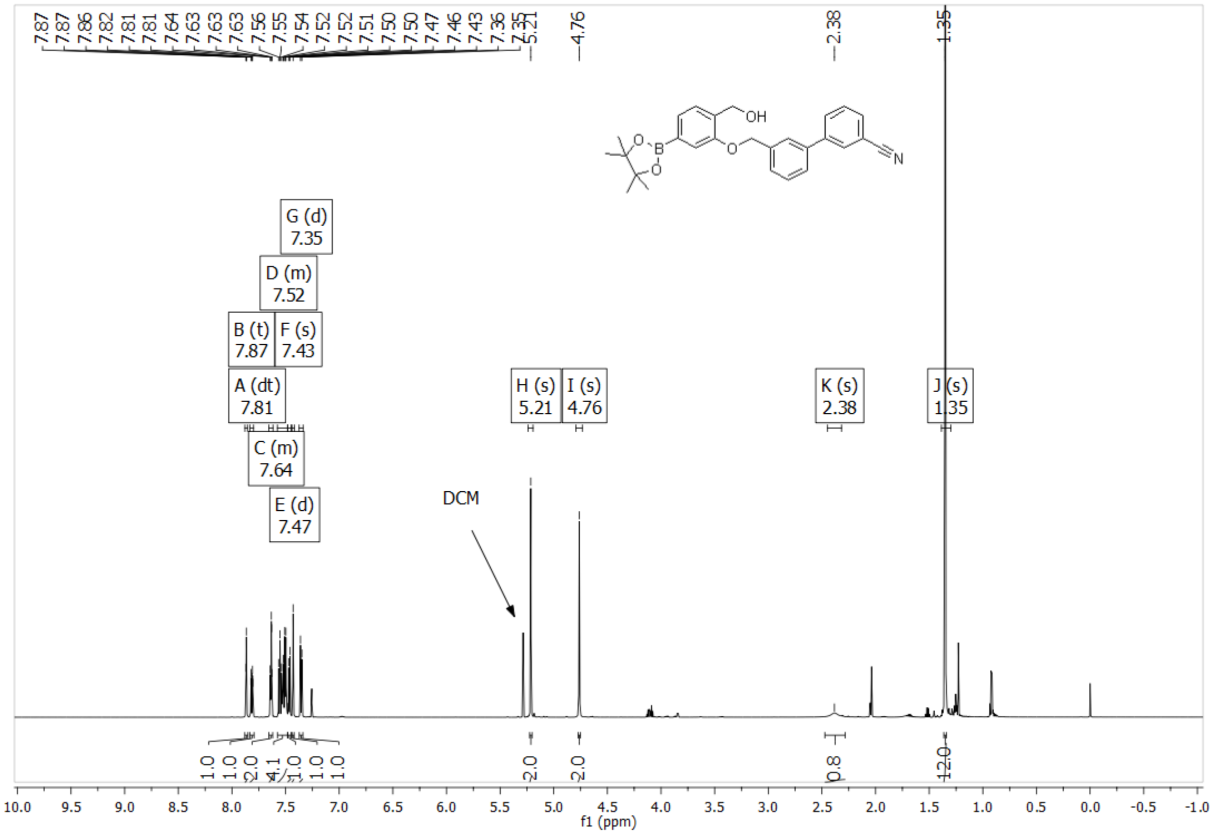

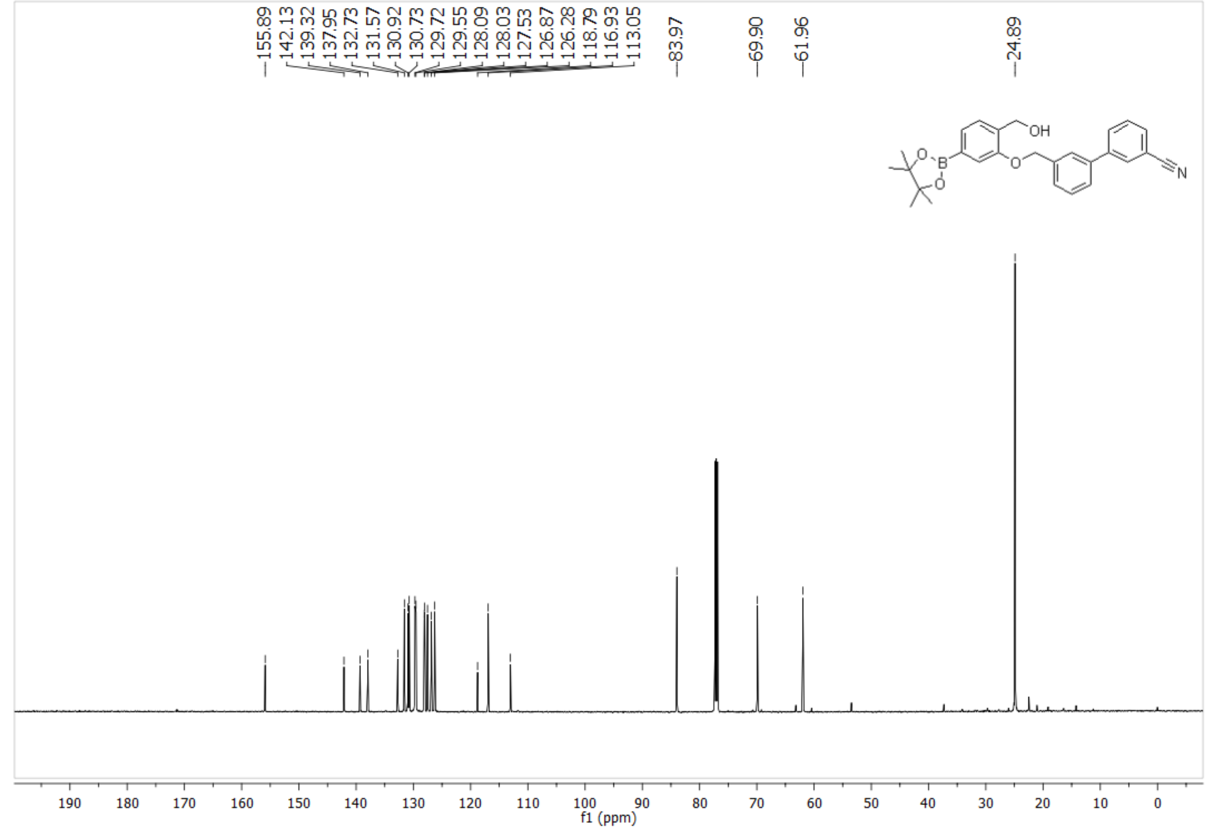

3i. 3-((2-(Hydroxymethyl)-5-(4,4,5,5-tetramethyl-1,3,2-dioxaborolan-2-yl)phenoxy)methyl)benzonitrile

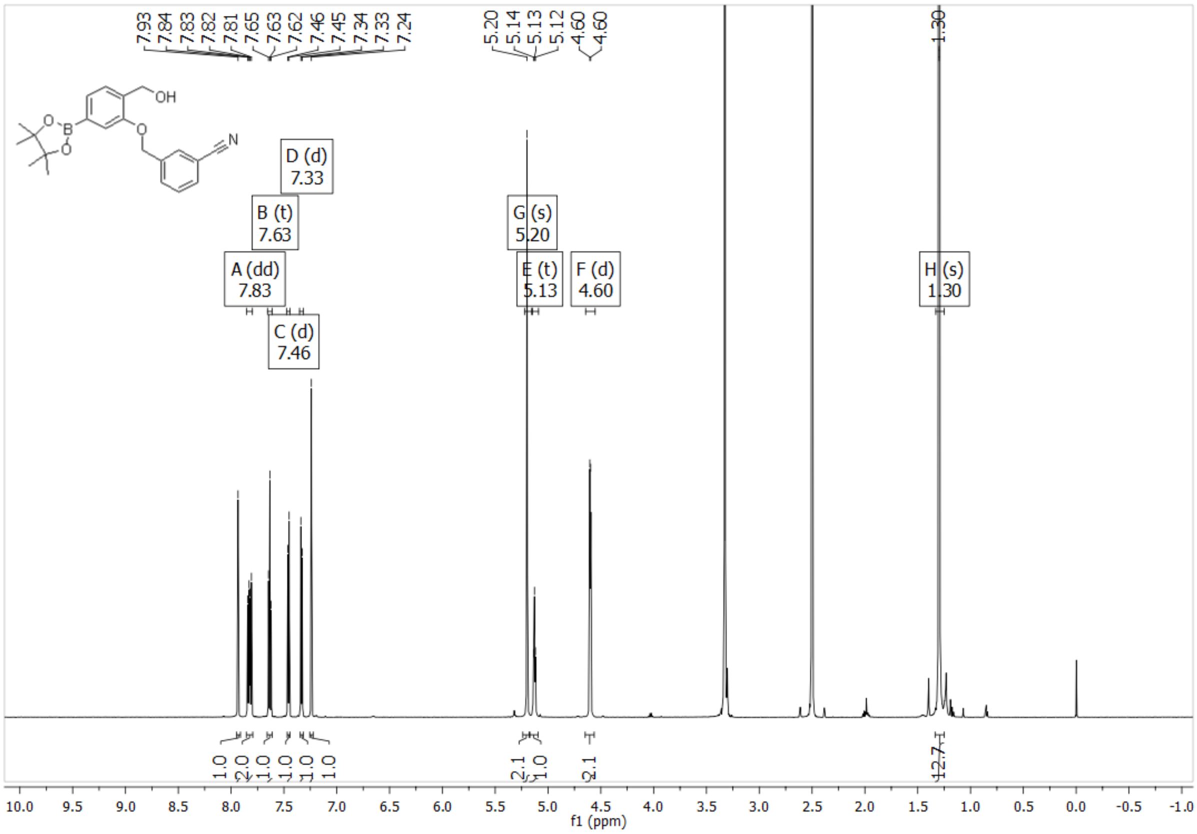

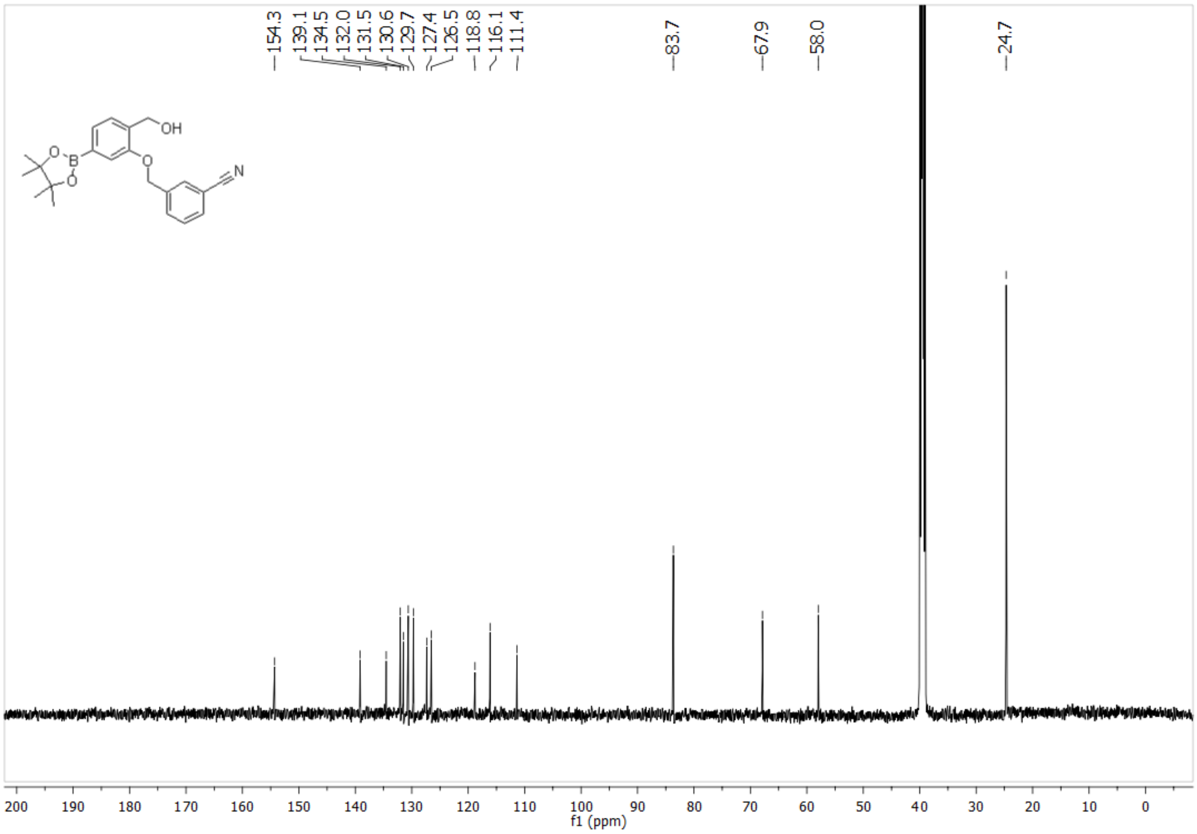

4a. (3'-(Benzo-1,4-dioxan-6-yl)-2'-methyl-[1,1'-biphenyl]-4-yl)methanol

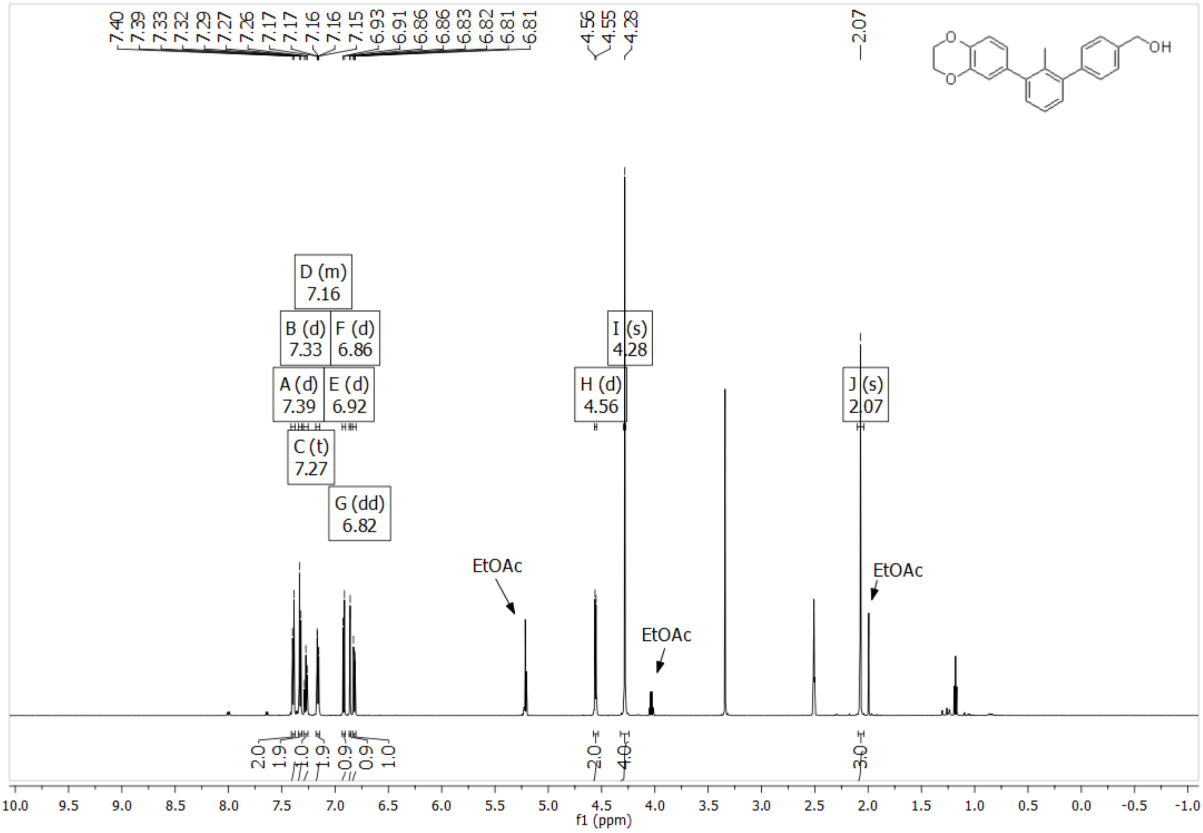

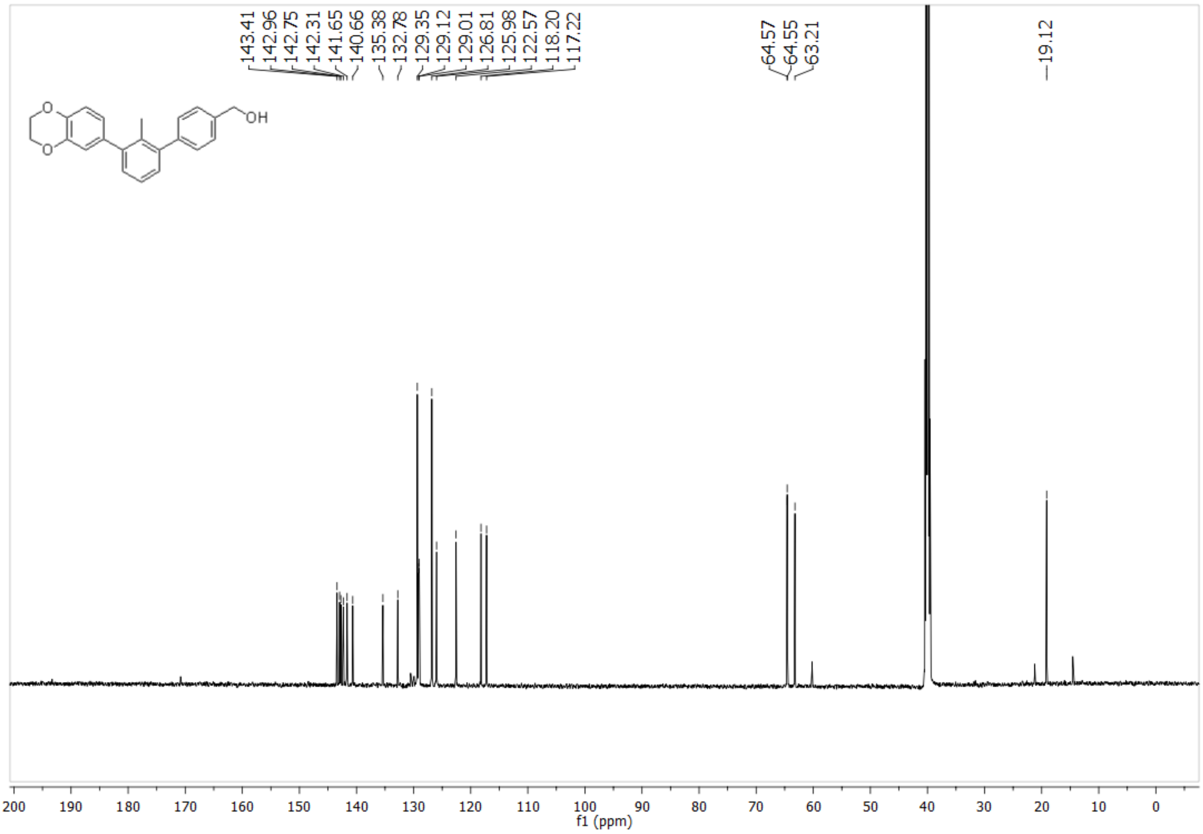

4b. (3'-(Benzo-1,4-dioxan-6-yl)-2'-methyl-[1,1'-biphenyl]-3-yl)methanol

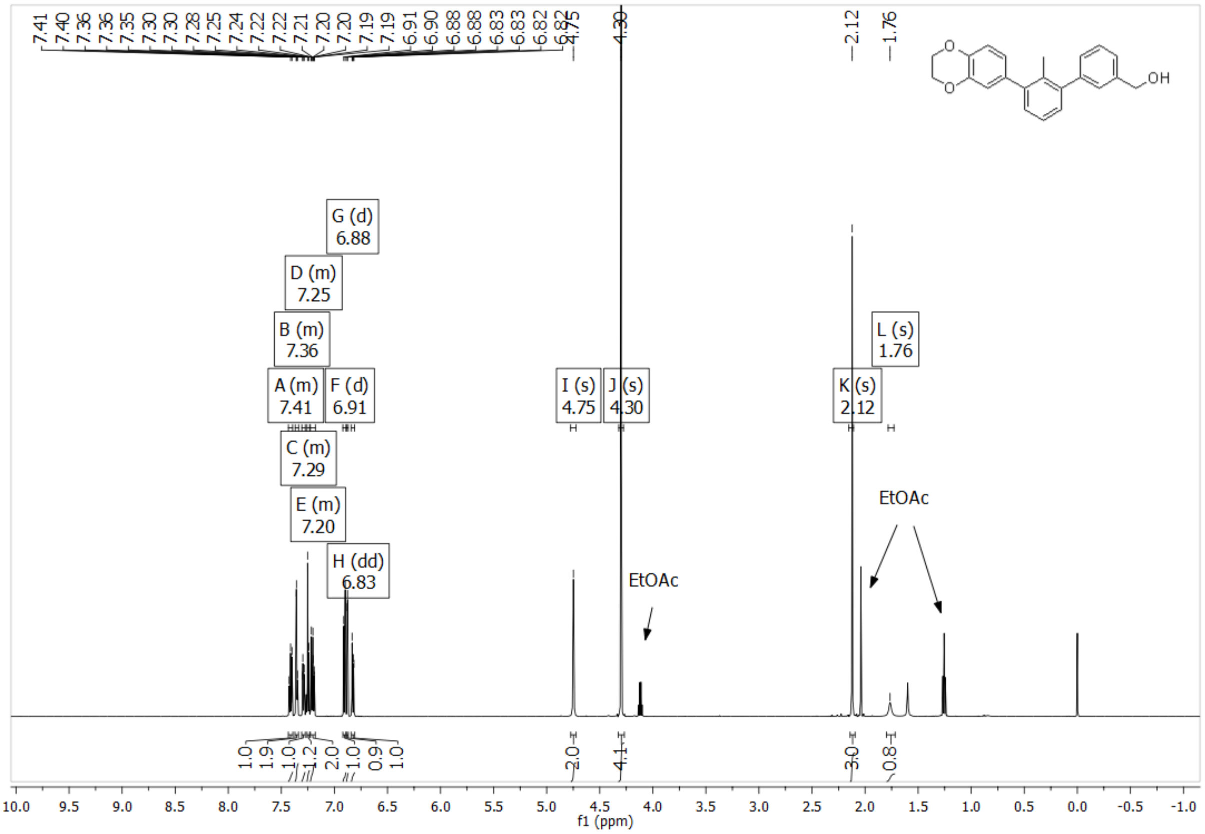

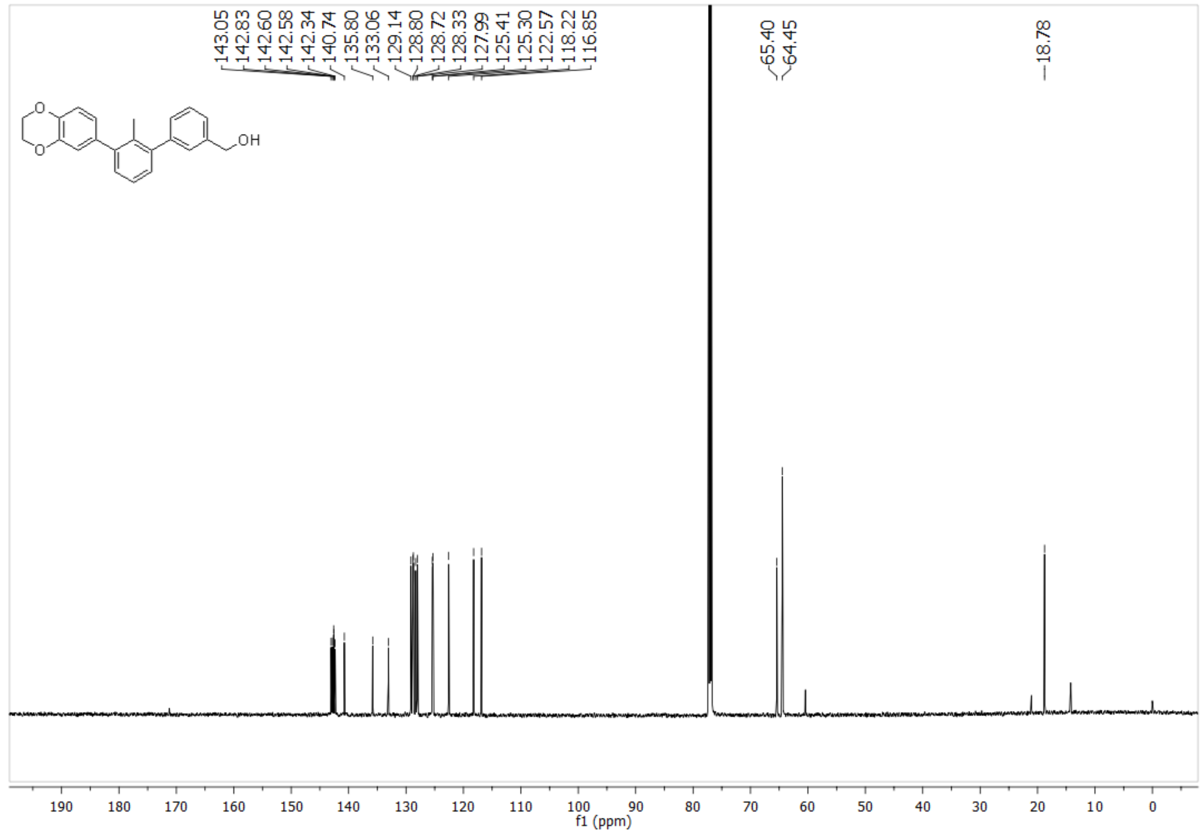

4c. (3'-(Benzo-1,4-dioxan-6-yl)-2'-methyl-[1,1'-biphenyl]-2-yl)methanol

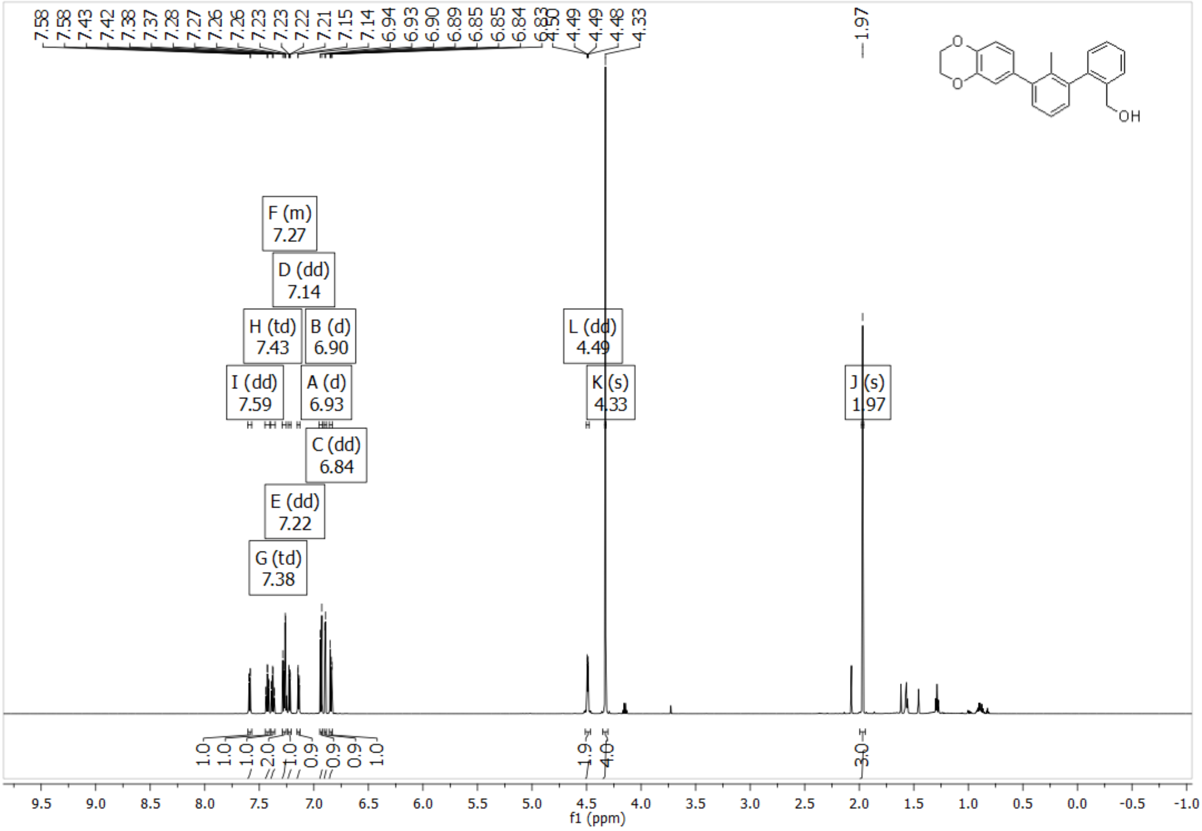

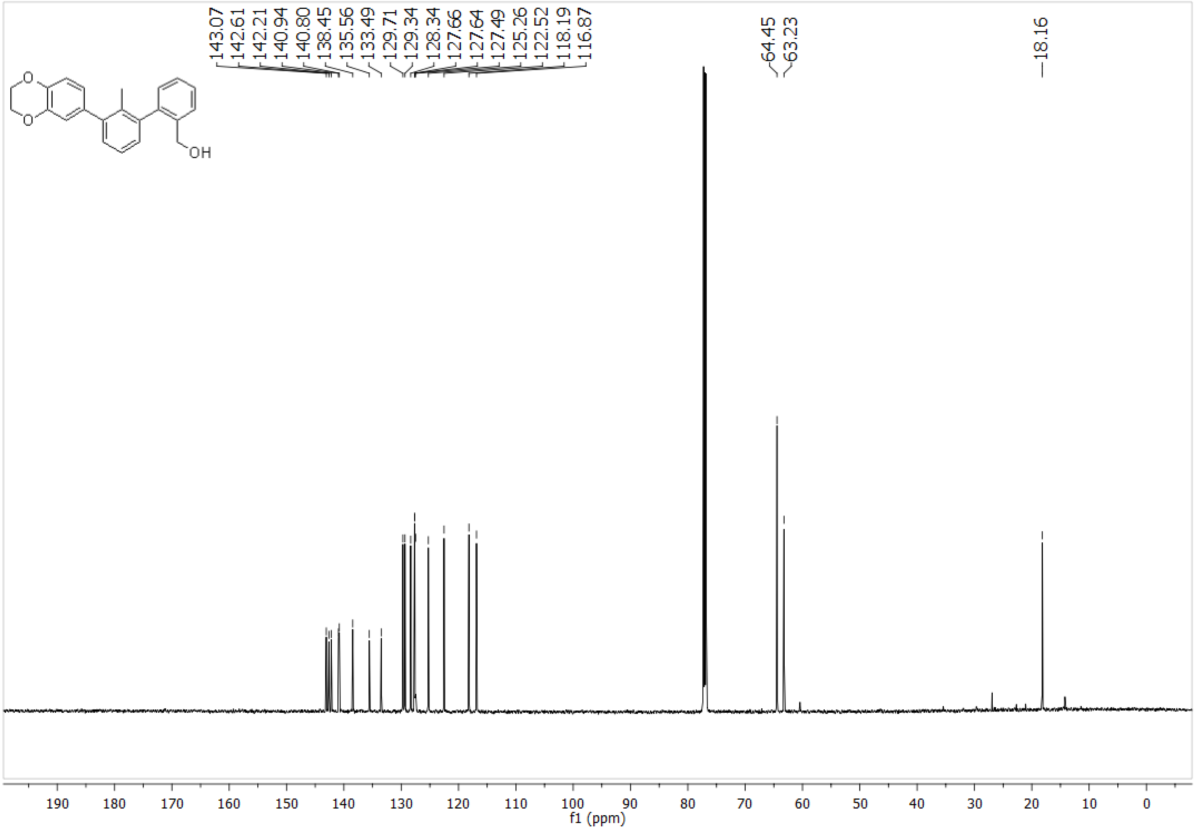

4d. (2'-Amino-3'-(benzo-1,4-dioxan-6-yl)-[1,1'-biphenyl]-4-yl)methanol

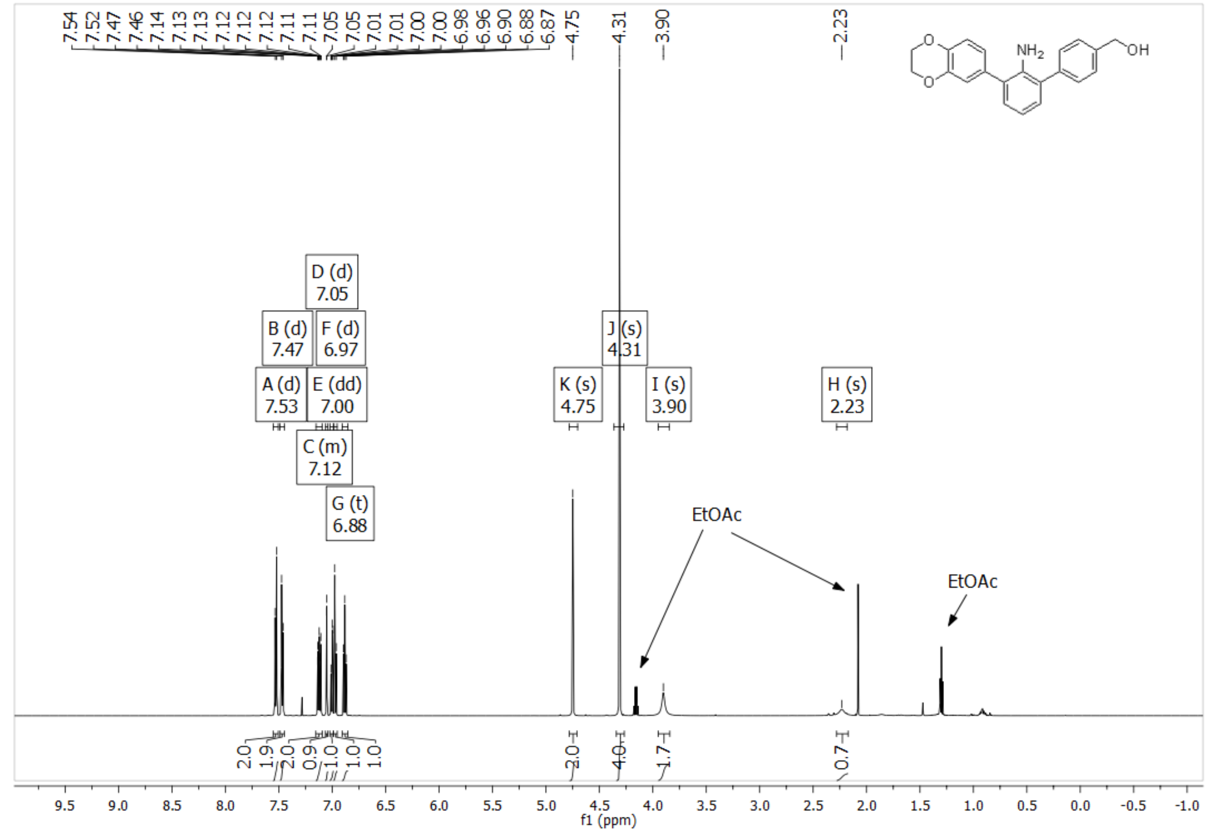

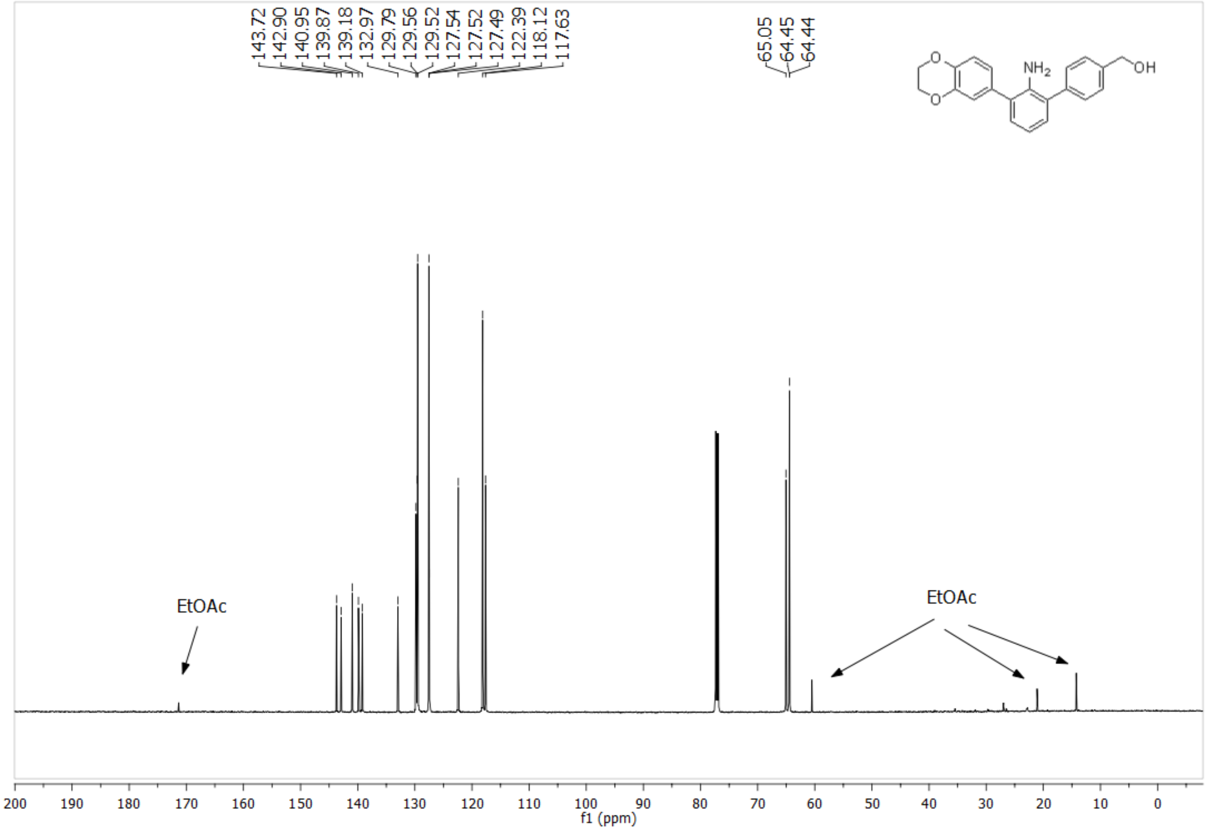

4e. (3'-(Benzo-1,4-dioxan-6-yl)-2'-chloro-[1,1'-biphenyl]-4-yl)methanol

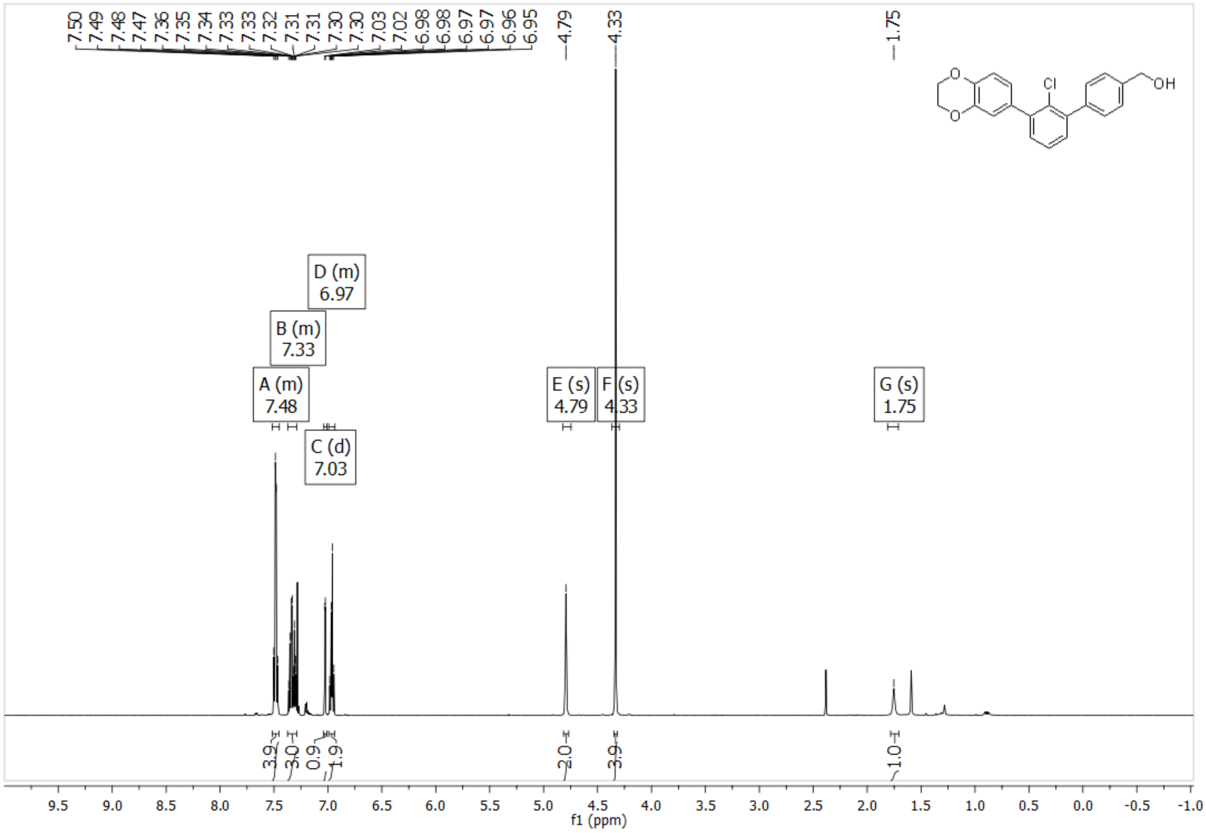

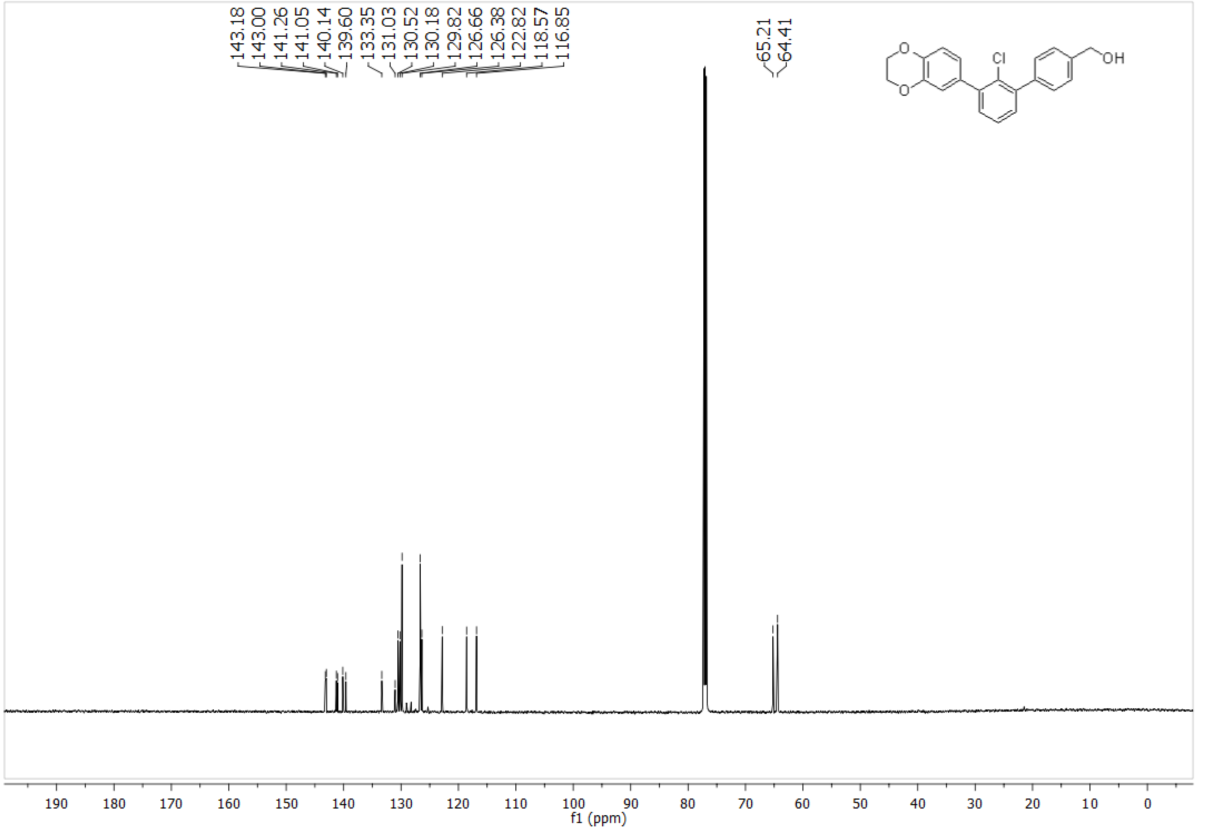

4f. (2'-Bromo-3'-(benzo-1,4-dioxan-6-yl)-[1,1'-biphenyl]-4-yl)methanol

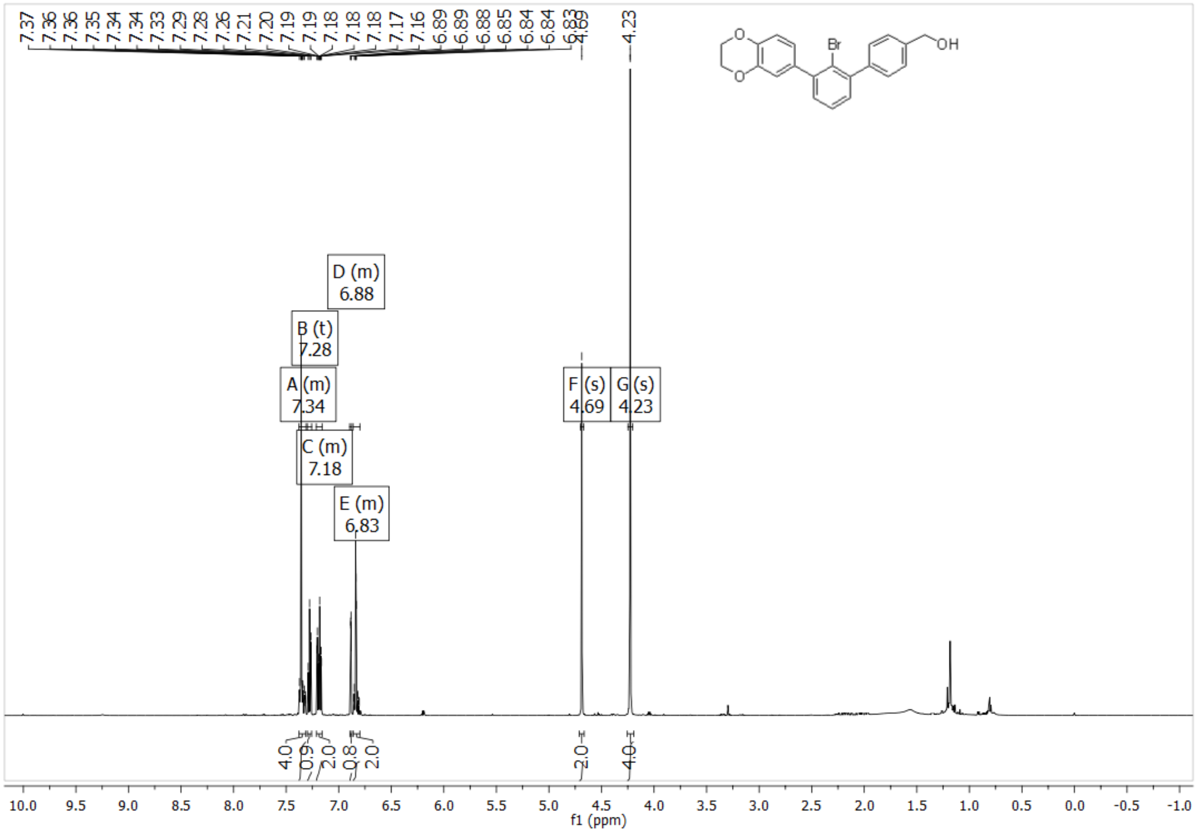

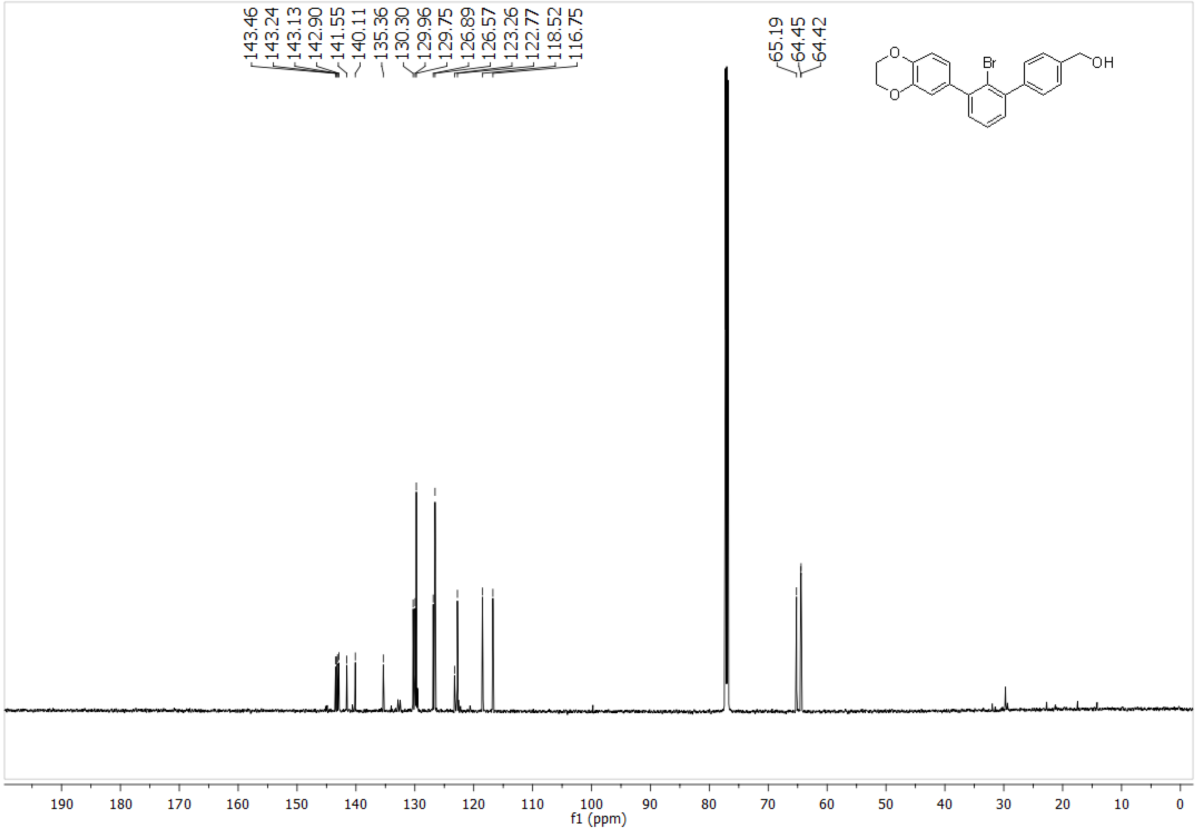

4g. (3'-(Benzo-1,4-dioxan-6-yl)-2'-iodo-[1,1'-biphenyl]-4-yl)methanol

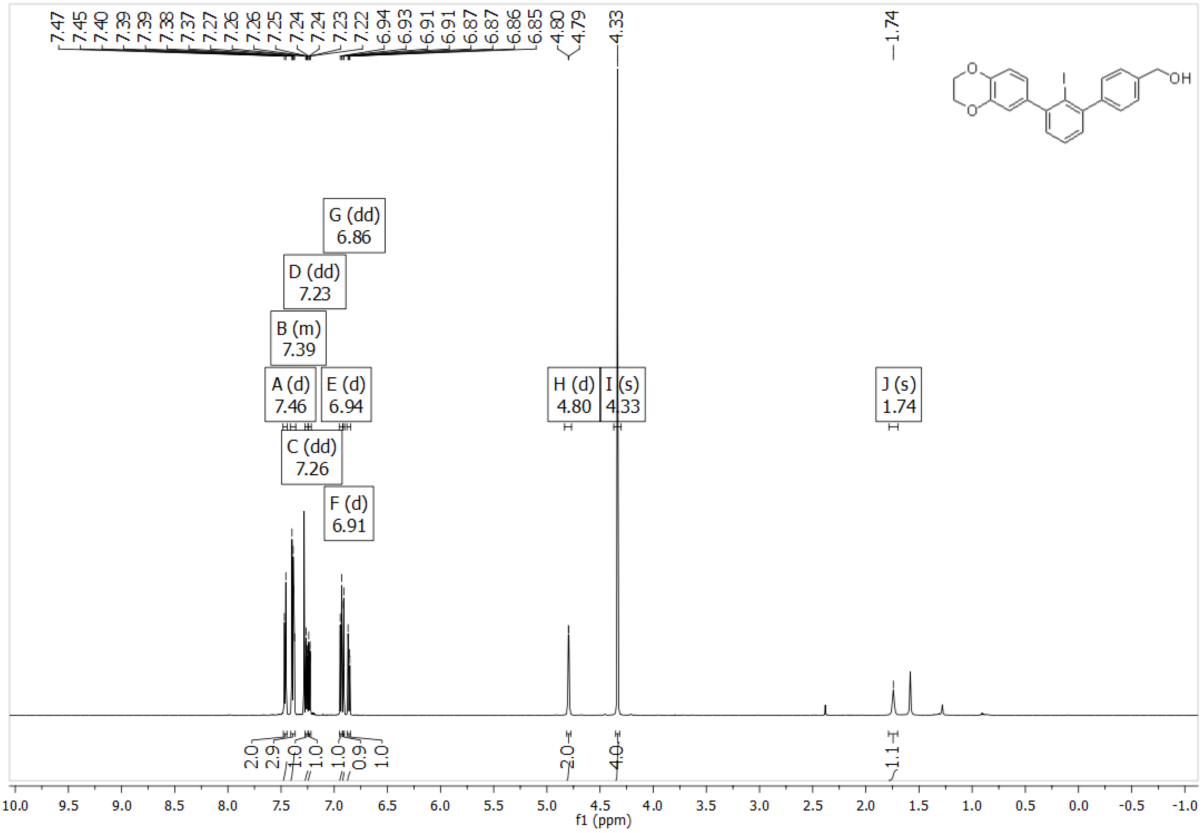

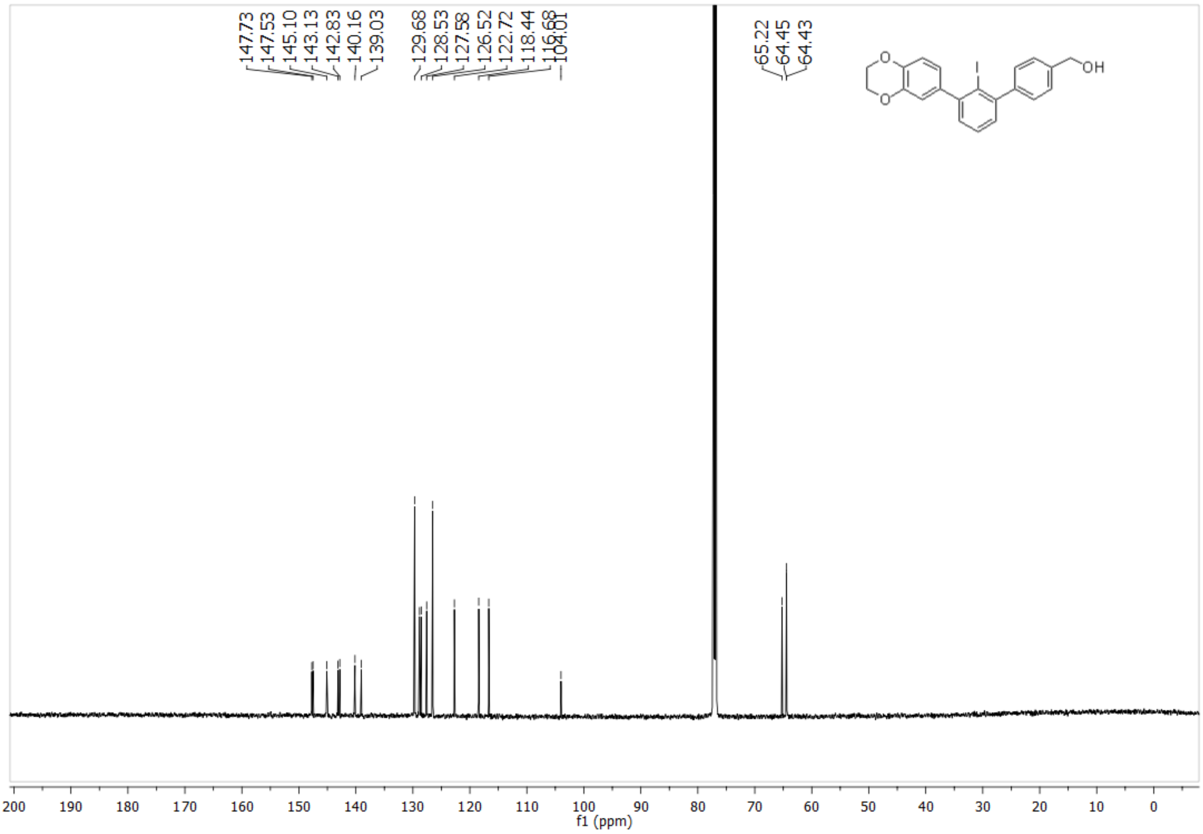

4h. 3-(3'-(Benzo-1,4-dioxan-6-yl)-4'-(hydroxymethyl)-[1,1'-biphenyl]-2-carbonitrile

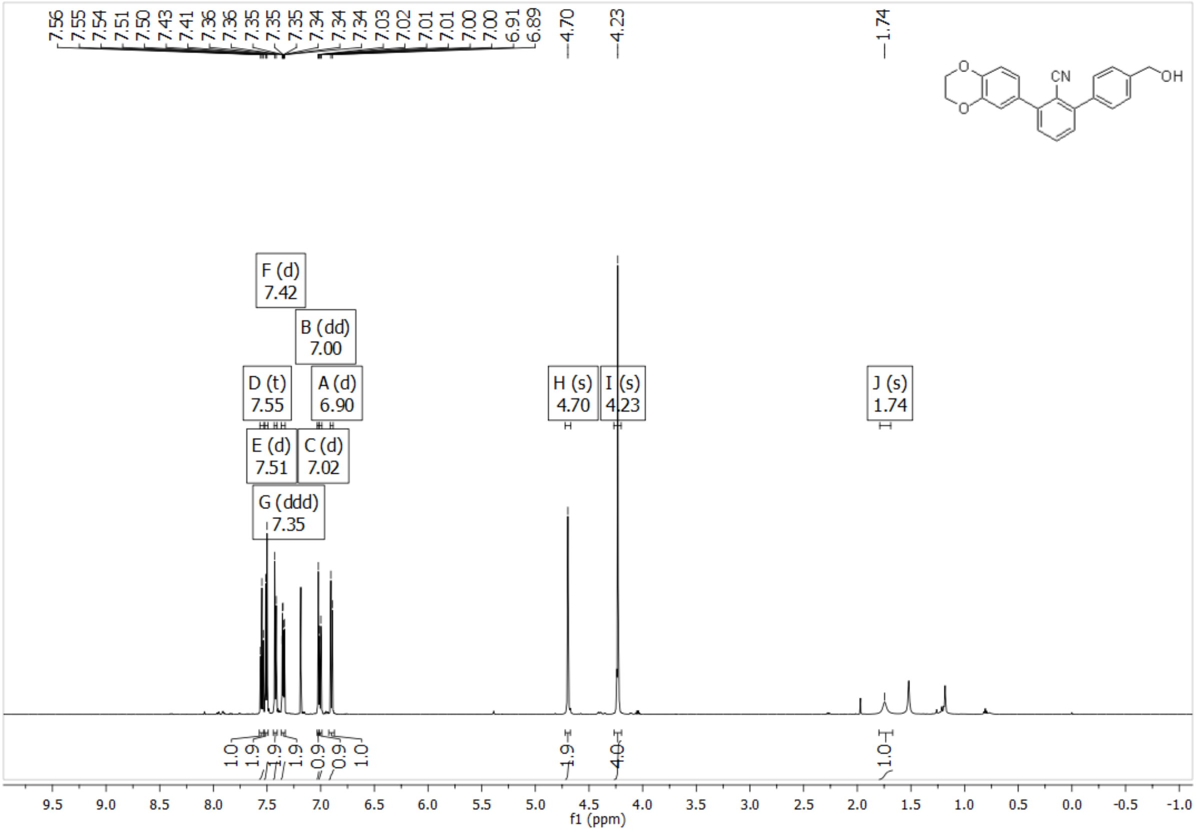

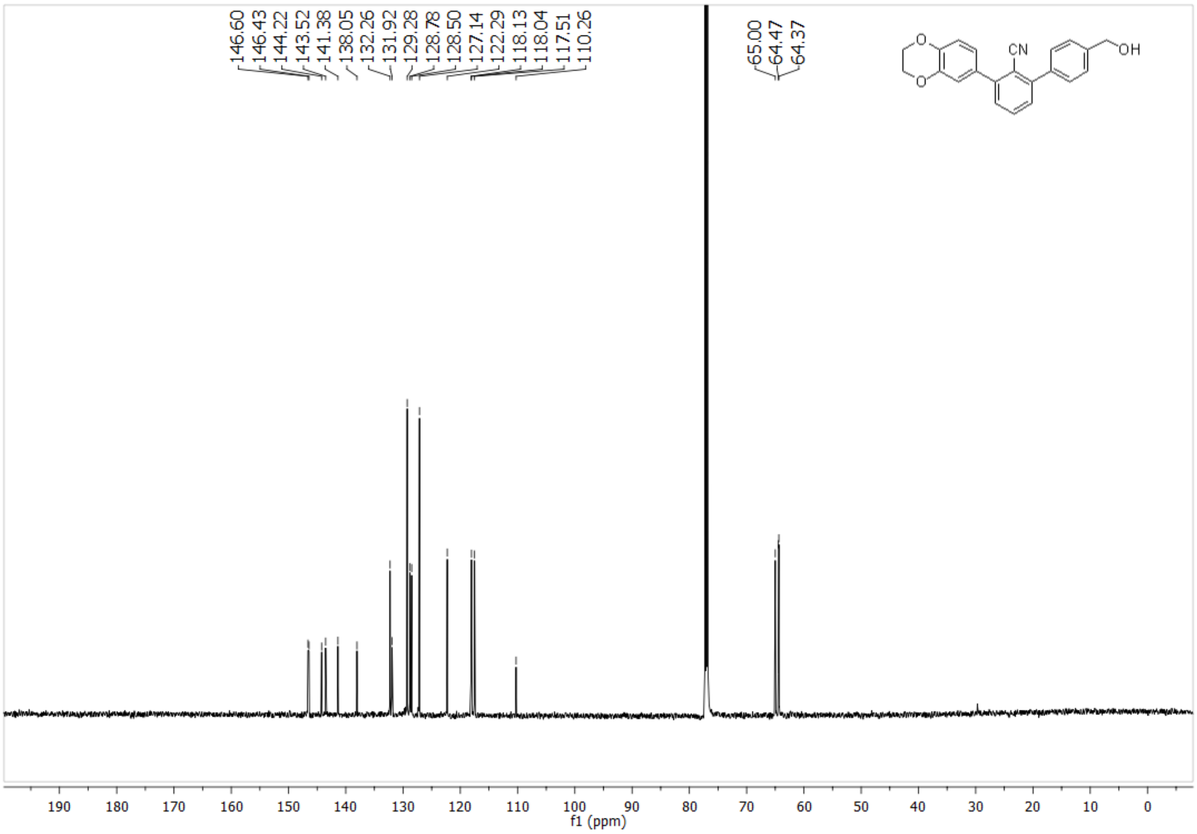

4i. (3'-(Benzo-1,4-dioxan-6-yl)-3-methoxy-2'-methyl-[1,1'-biphenyl]-4-yl)methanol

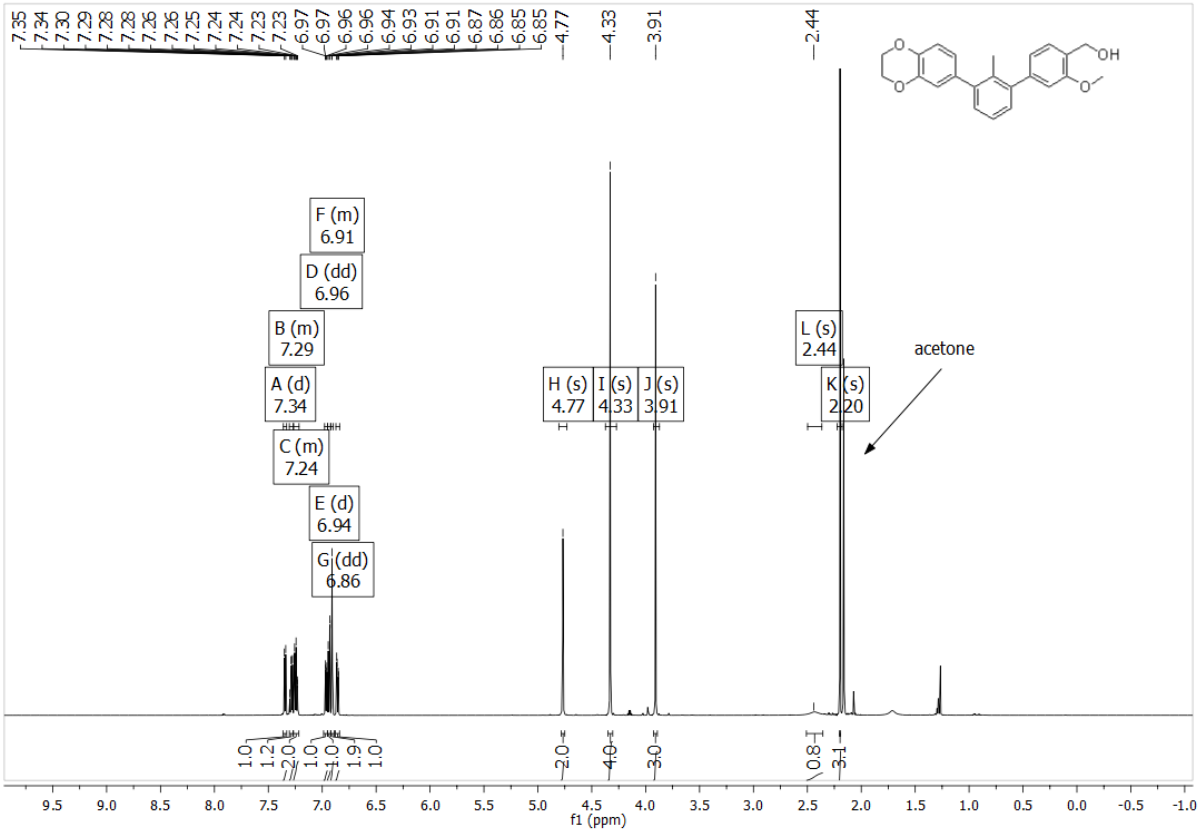

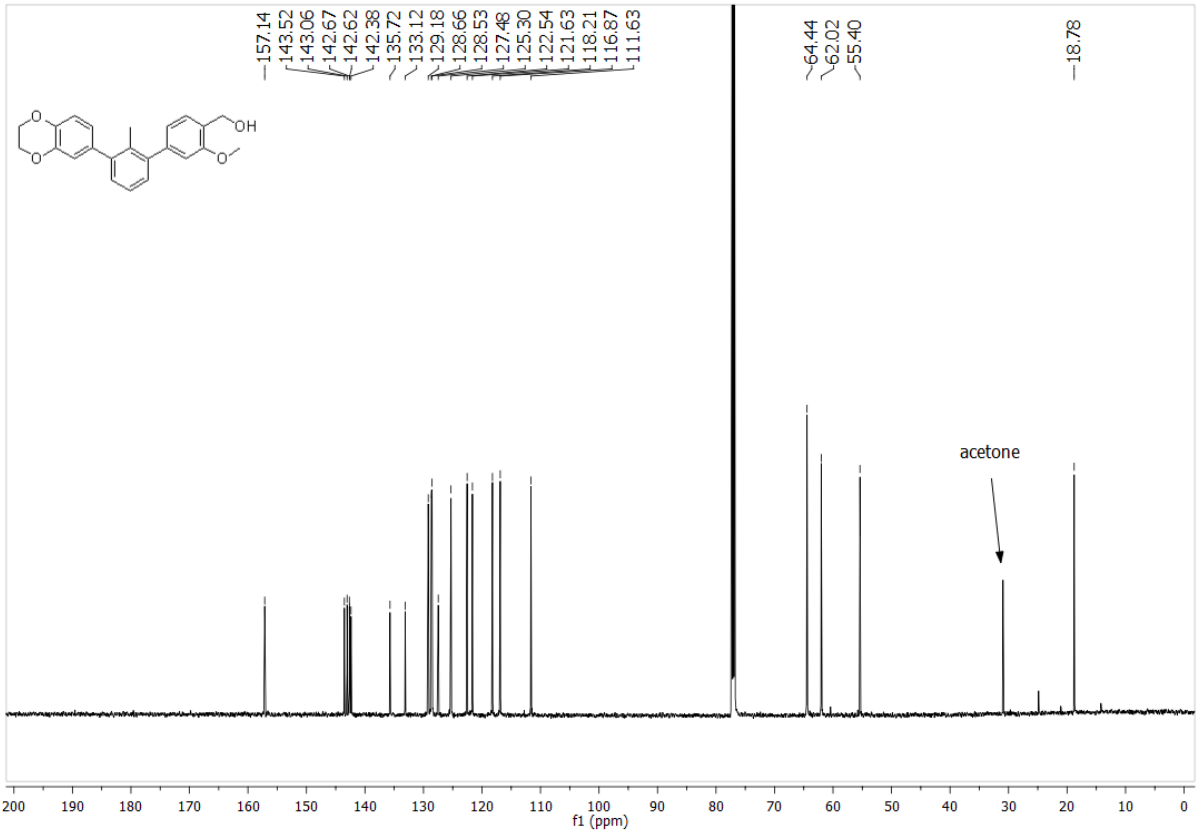

4j. 3-(((3'-(Benzo-1,4-dioxan-6-yl)-4-(hydroxymethyl)-2'-methyl-[1,1'-biphenyl]-3-yl)oxy)methyl)benzonitrile

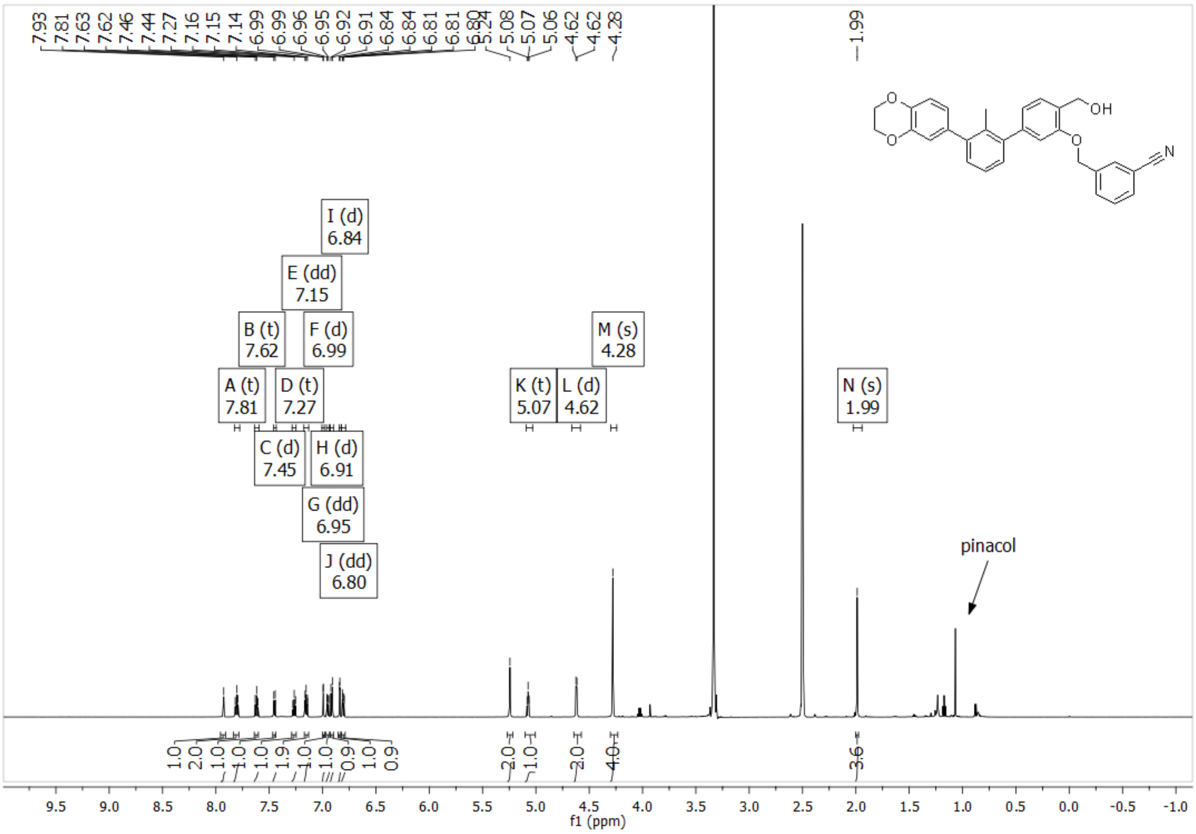

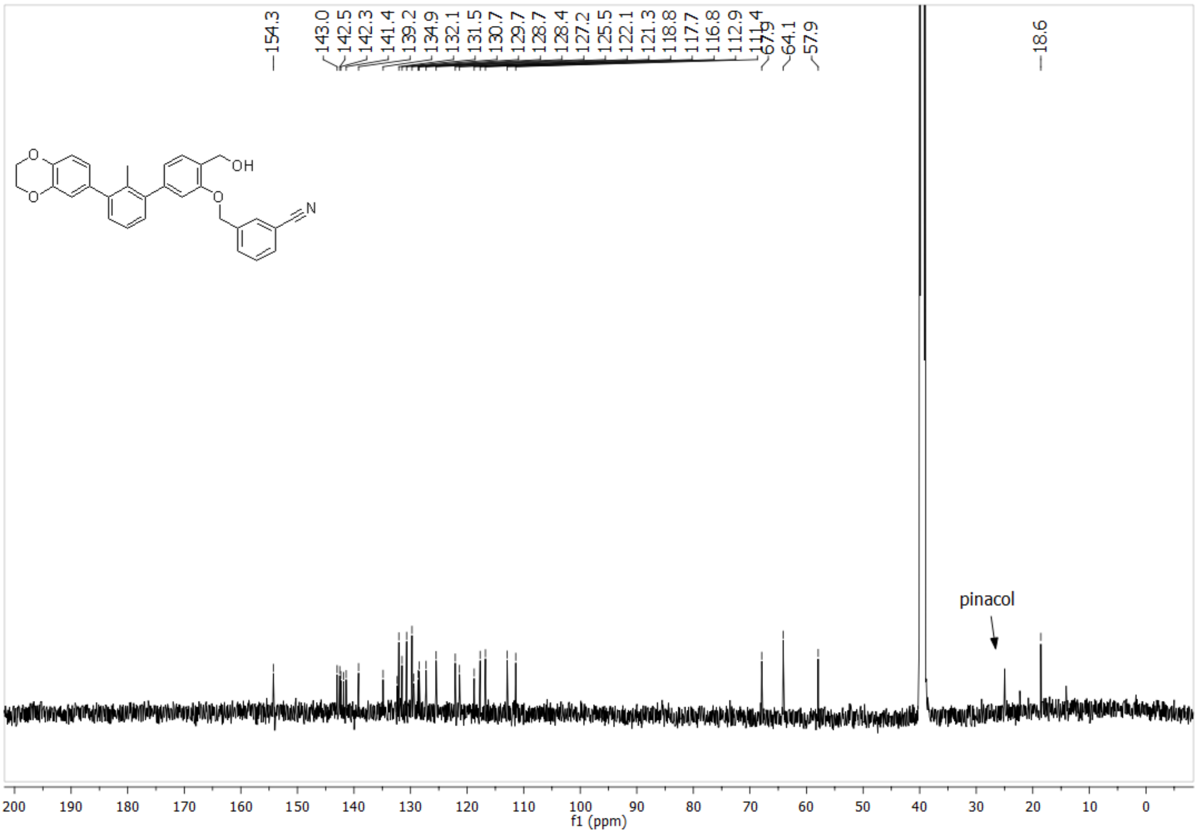

4k. (2'-Chloro-3'-(benzo-1,4-dioxan-6-yl)-3-methoxy-[1,1'-biphenyl]-4-yl)methanol

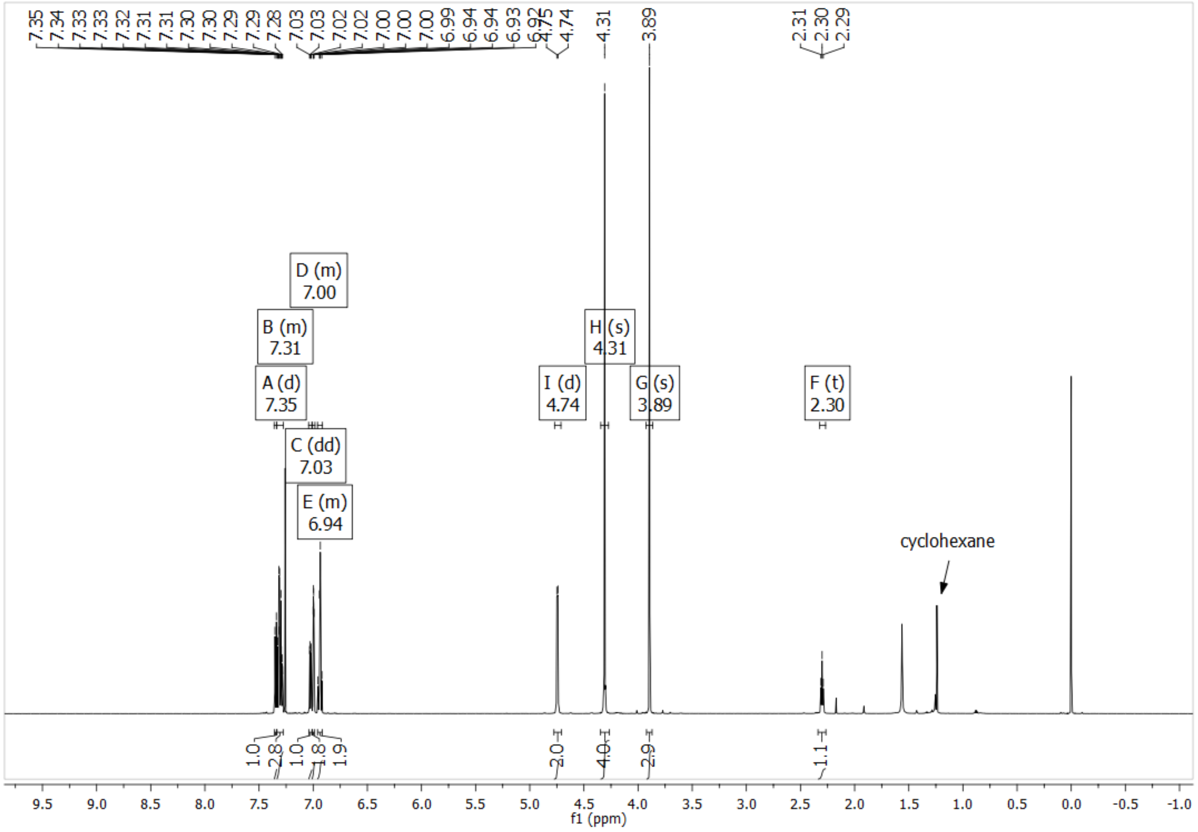

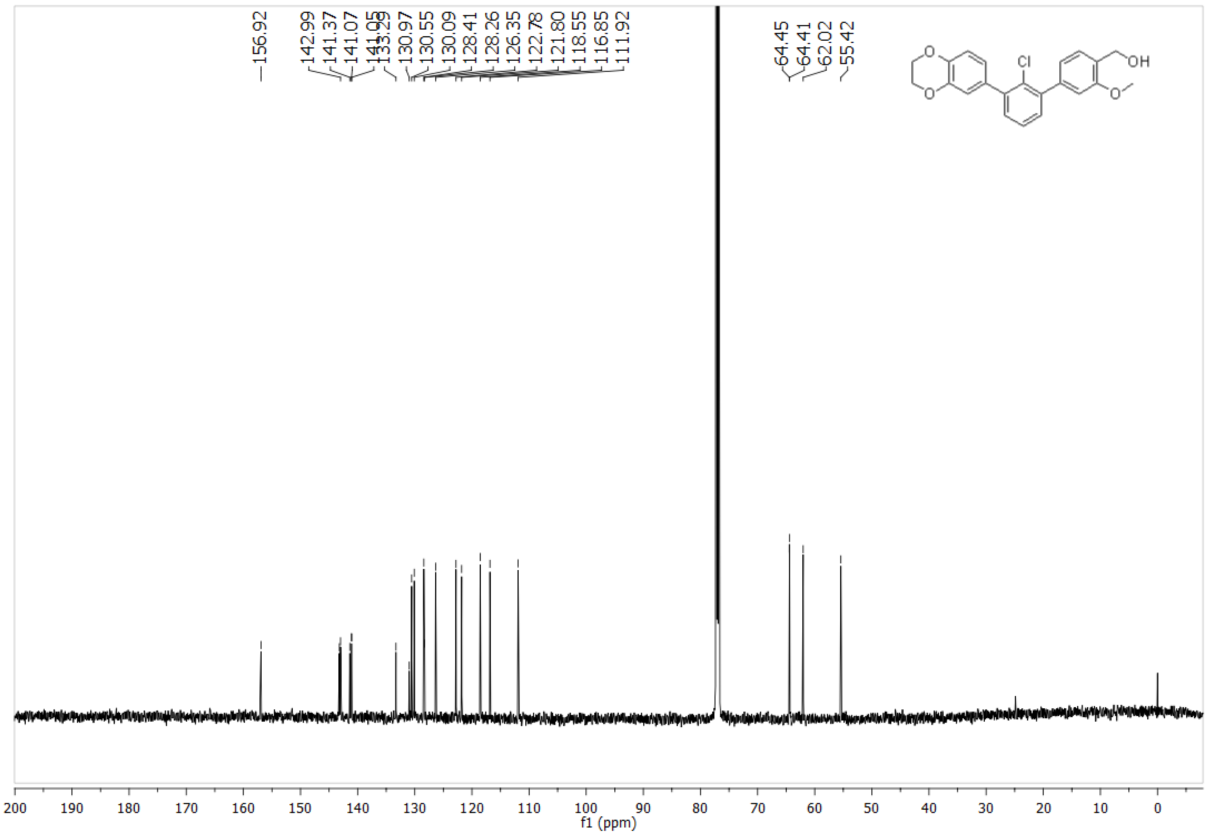

4l. (2'-Chloro-3'-(benzo-1,4-dioxan-6-yl)-3-ethoxy-[1,1'-biphenyl]-4-yl)methanol

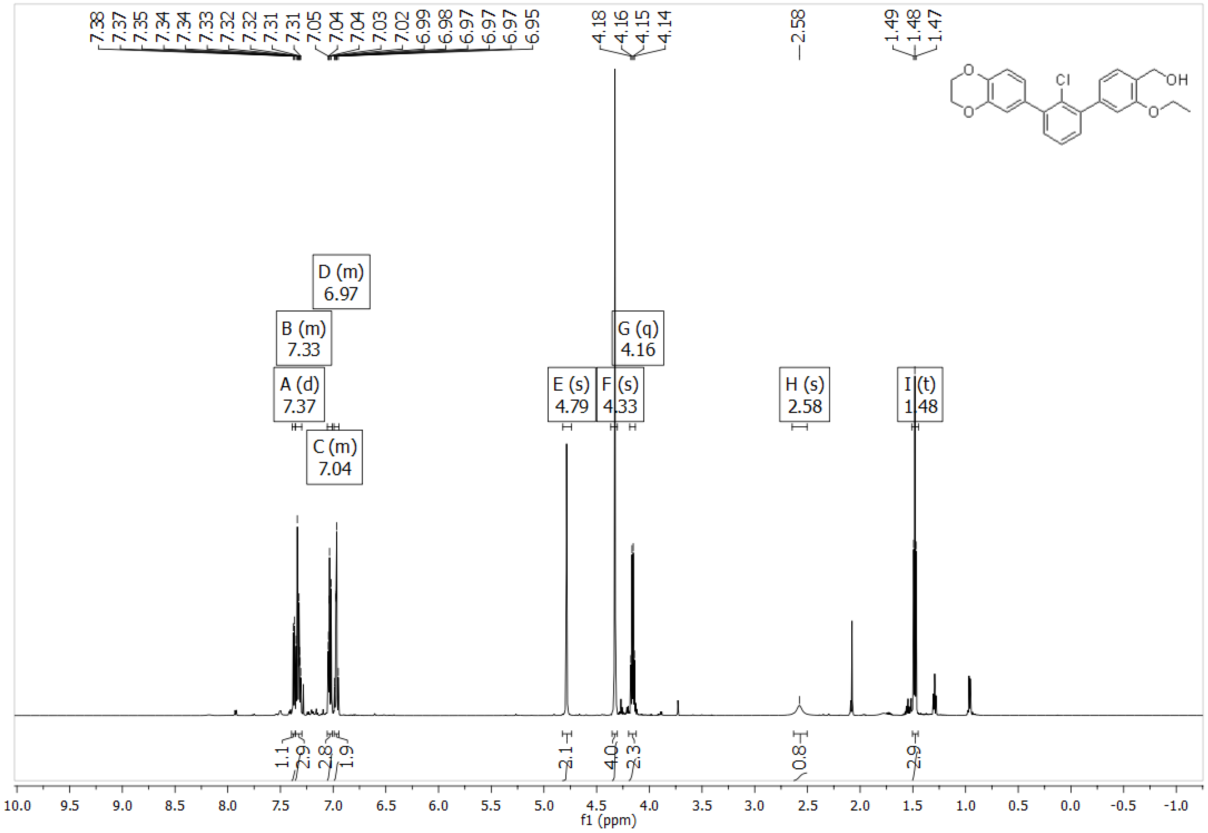

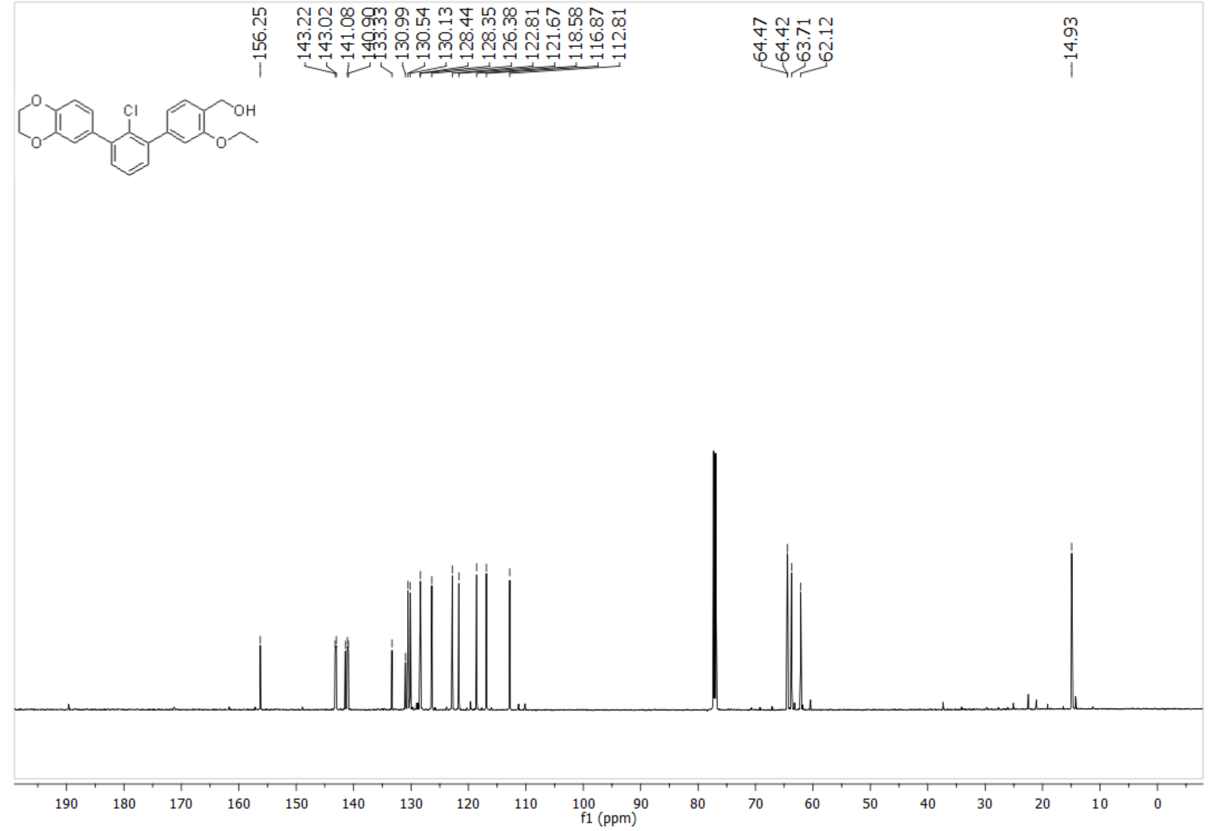

4m. (3'-(Benzo-1,4-dioxan-6-yl)-2’-chloro-3-isopropoxy-[1,1'-biphenyl]-4-yl)methanol

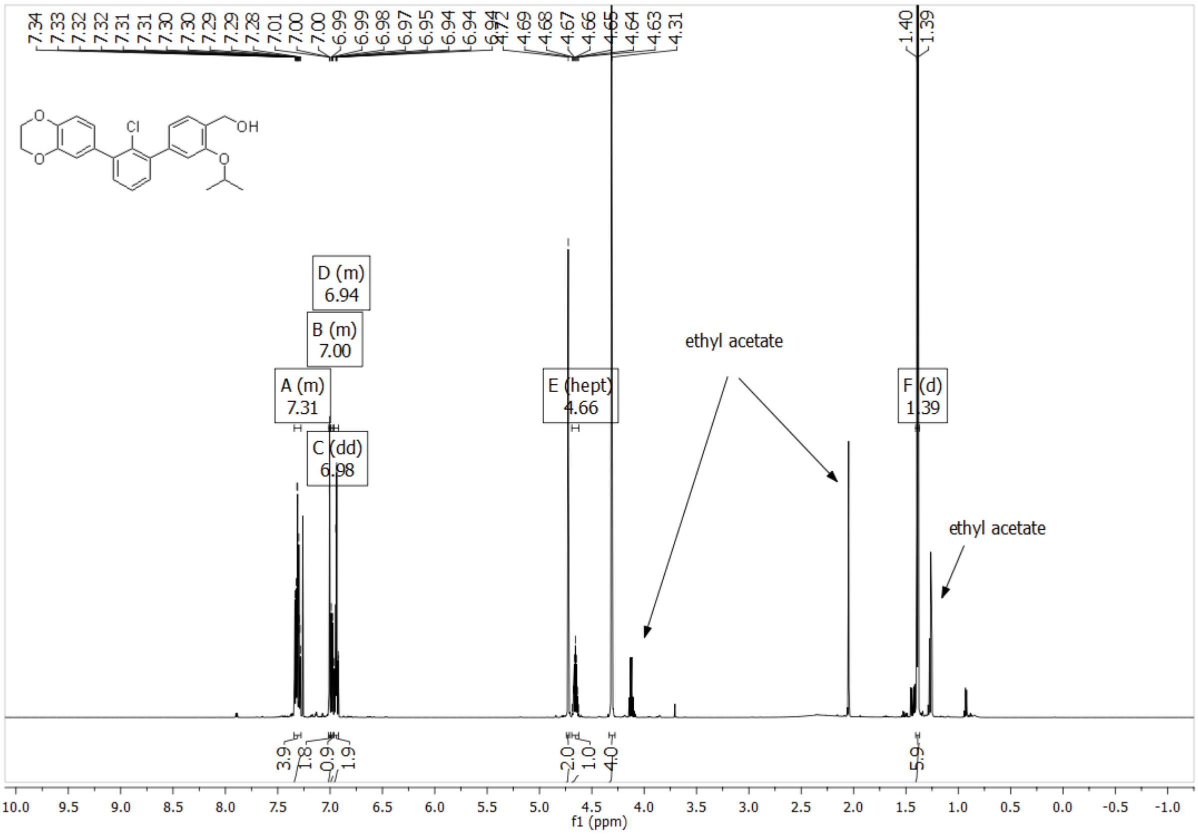

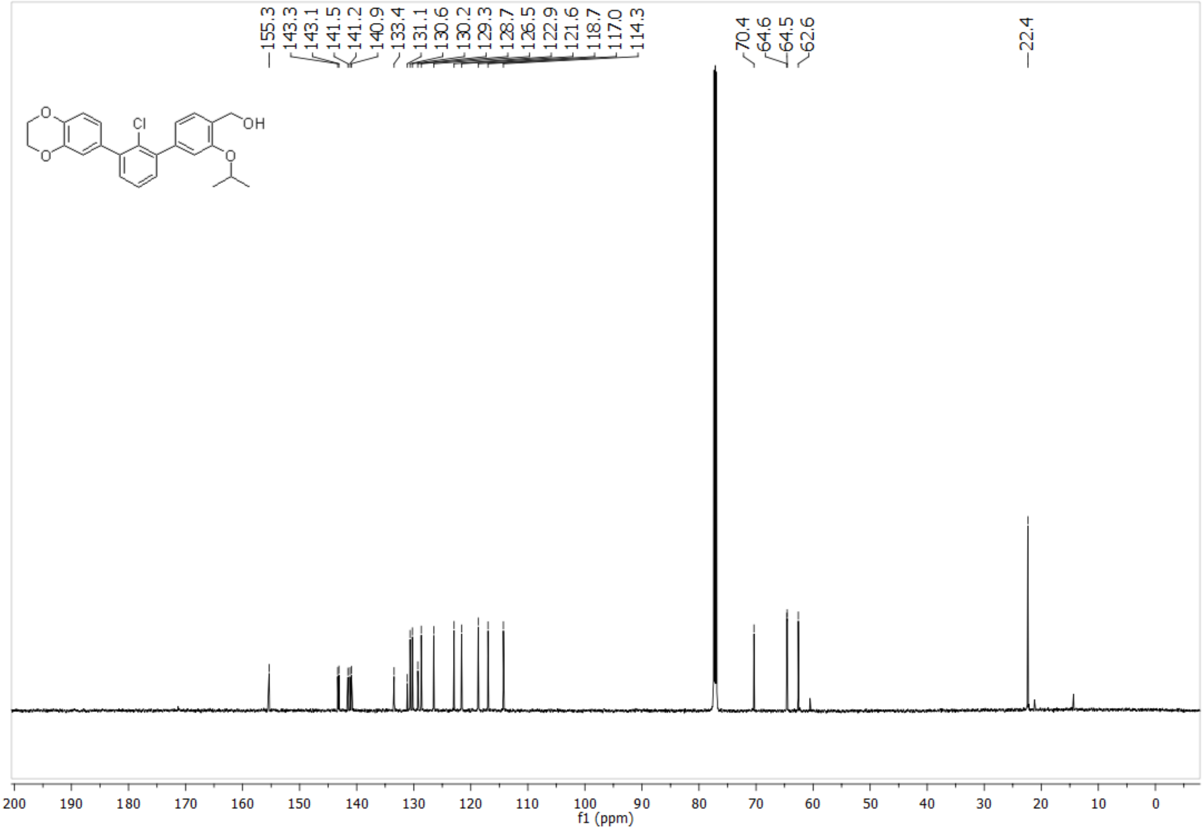

4n. (3'-(benzo-1,4-dioxan-6-yl)-2’-chloro-3-isopentyloxy-[1,1'-biphenyl]-4-yl)methanol

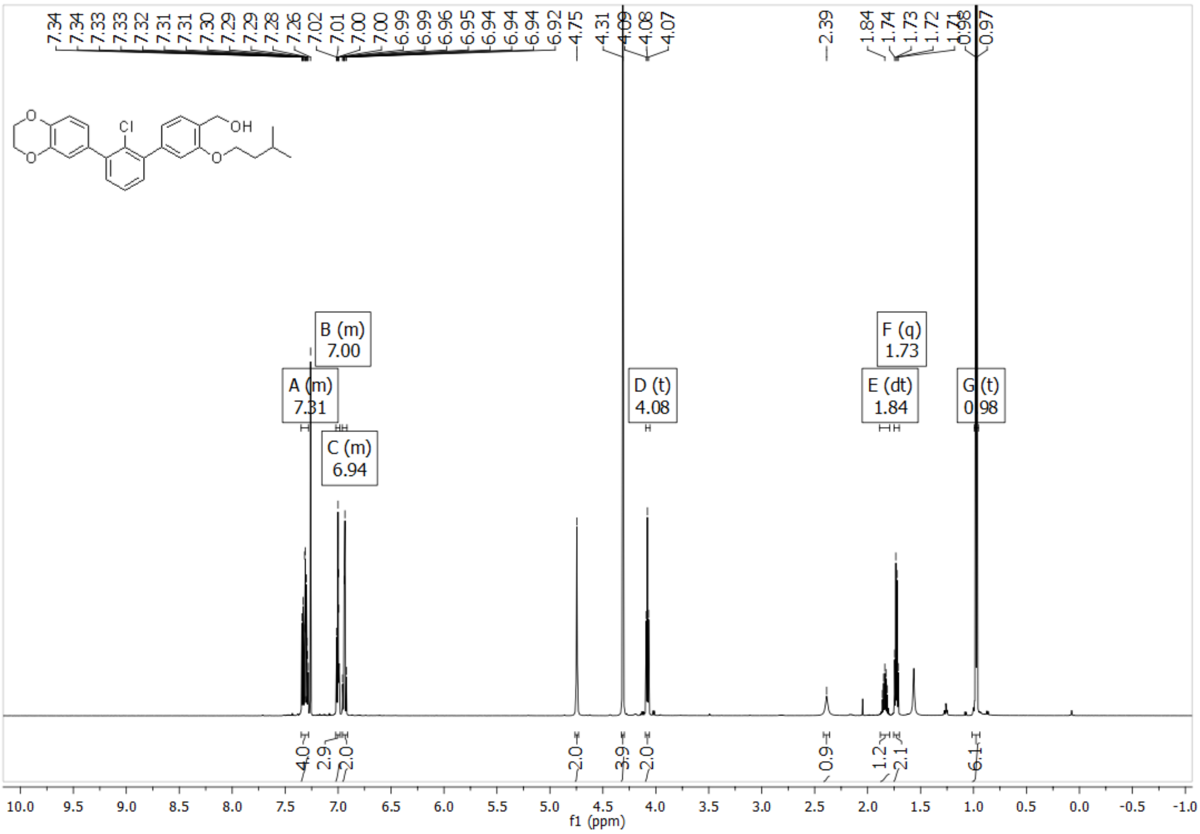

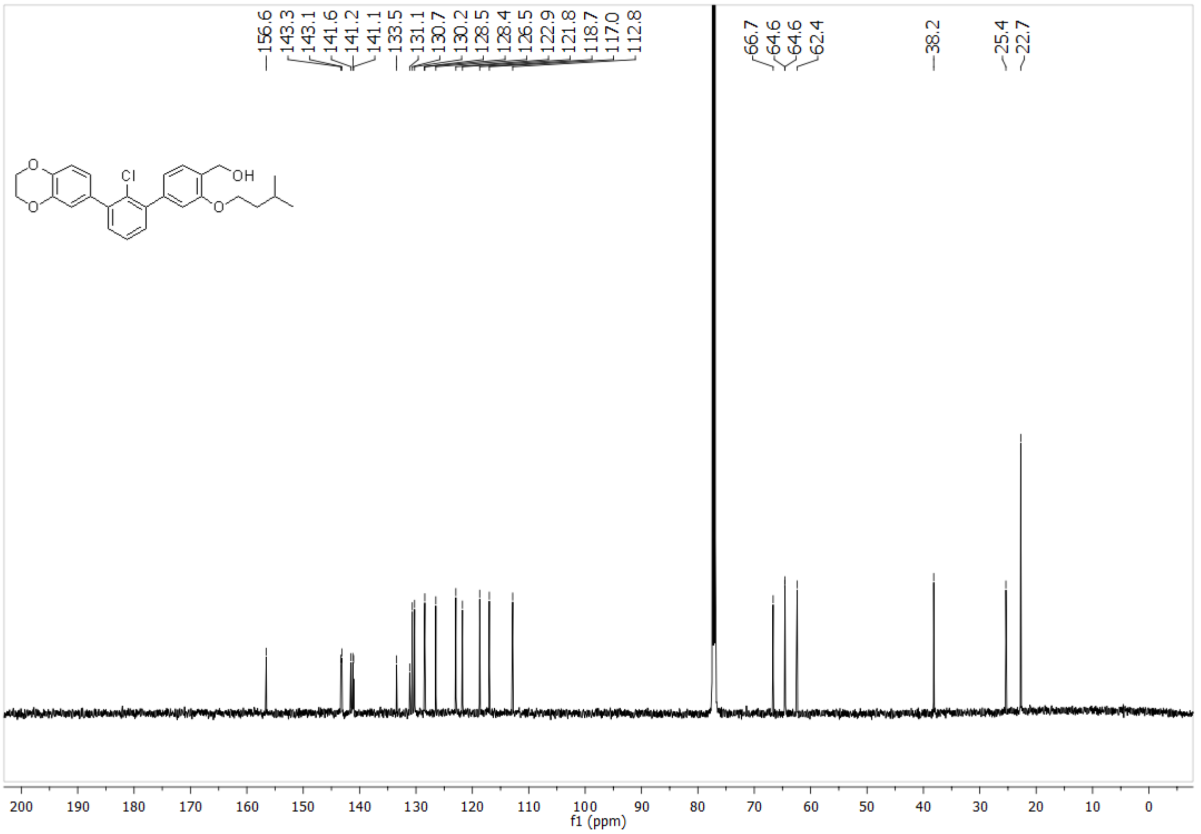

4o. 2-((3'-(Benzo-1,4-dioxan-6-yl)-2'-chloro-4-(hydroxymethyl)-[1,1'-biphenyl]-3-yl)oxy)acetonitrile

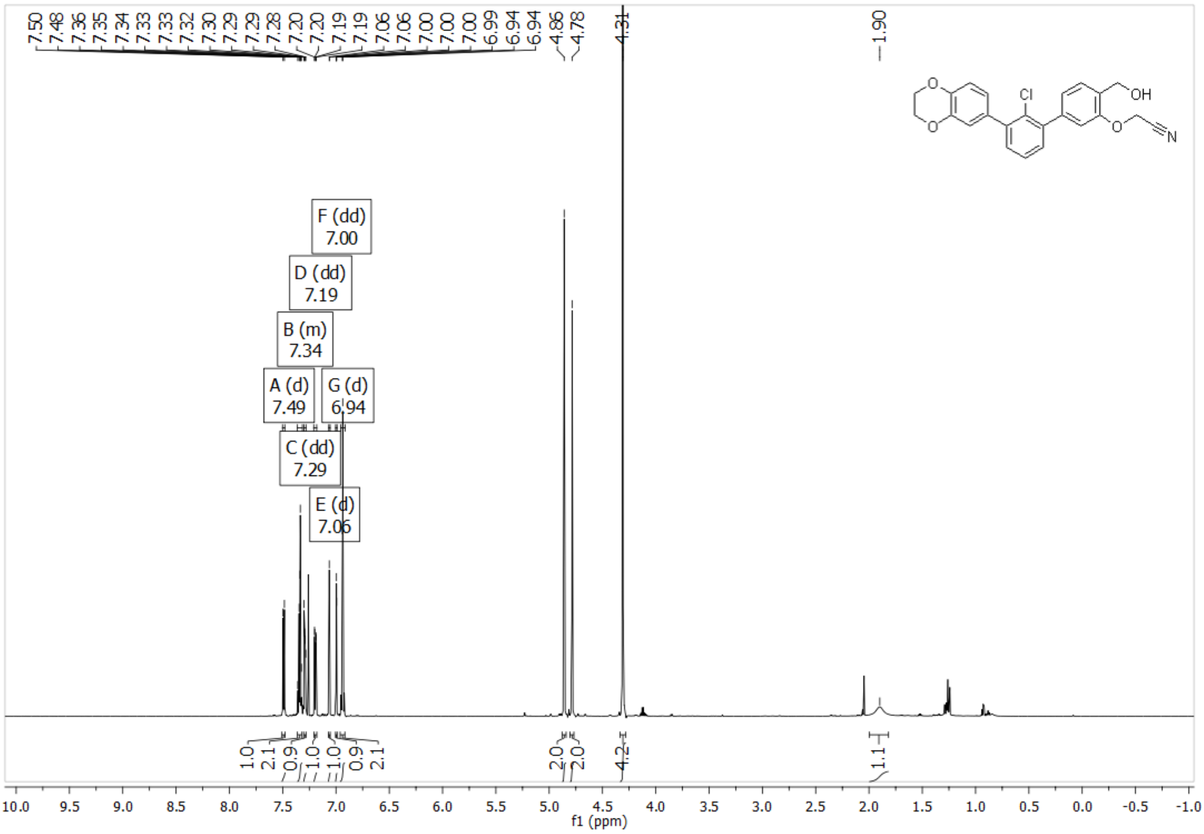

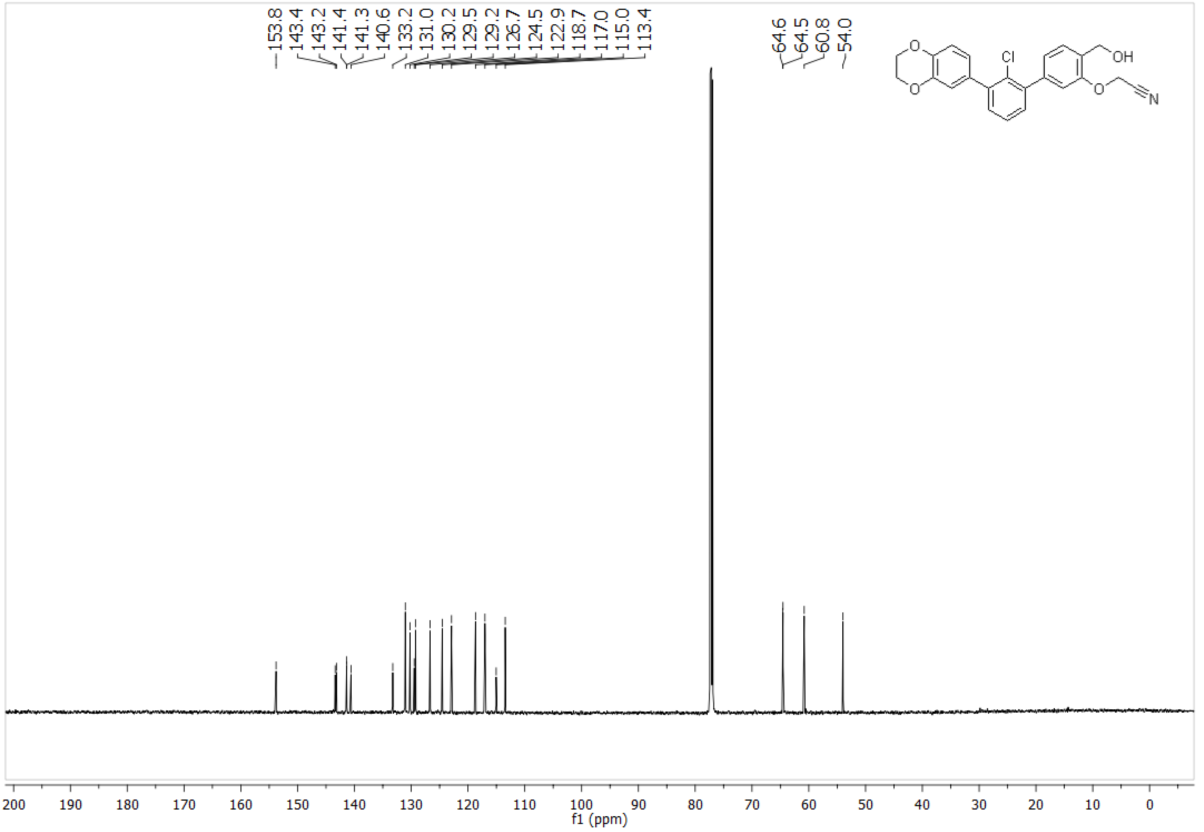

4p.(3'-(benzo-1,4-dioxan-6-yl)-2'-chloro-3-cyclobutylmethoxy-[1,1'-biphenyl]-4-yl)methanol

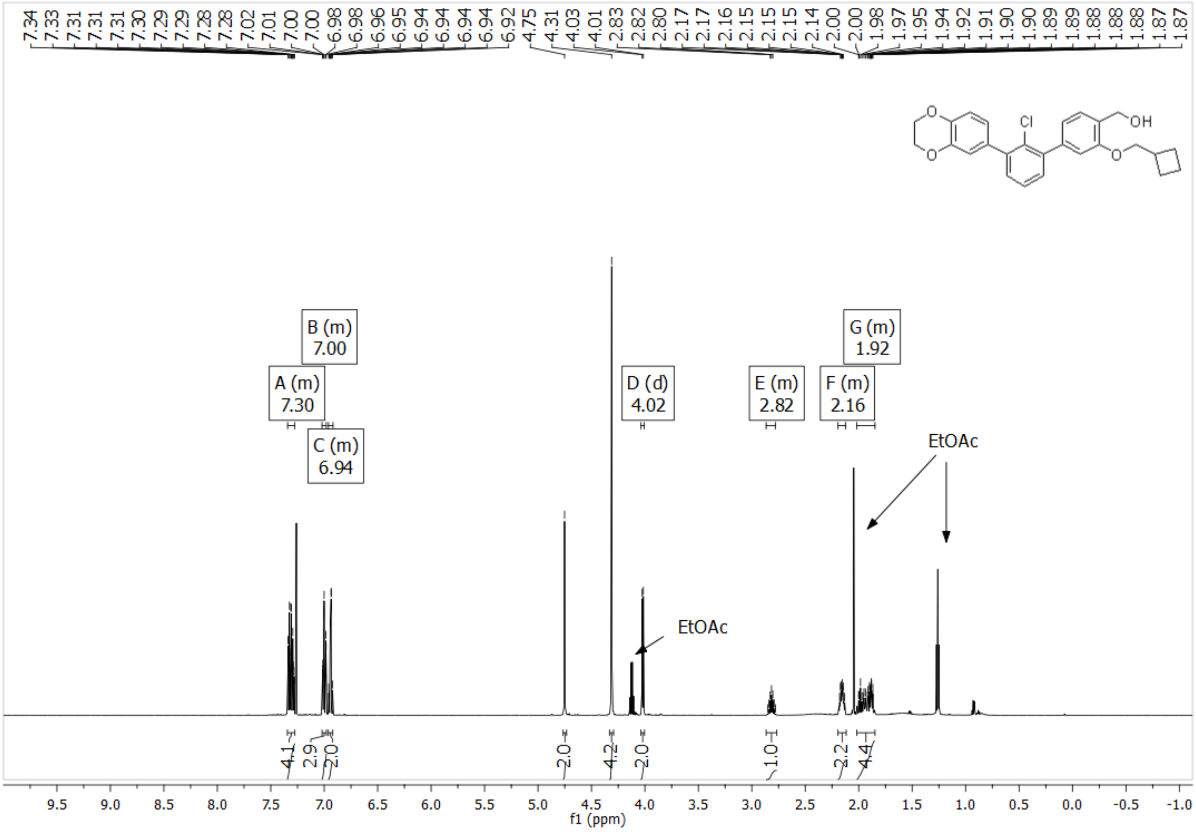

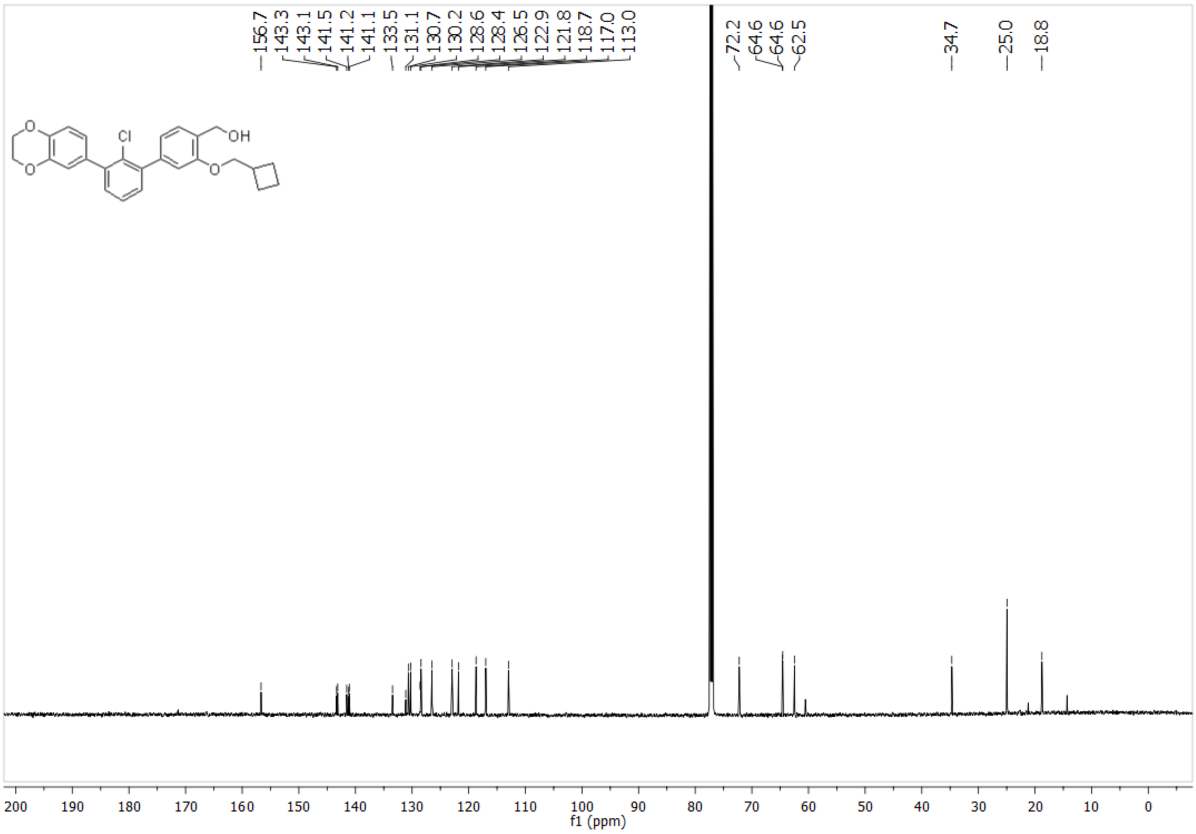

4q. (3'-(Benzo-1,4-dioxan-6-yl)-2’-chloro-3-cyclopentylmethoxy-[1,1'-biphenyl]-4-yl)methanol

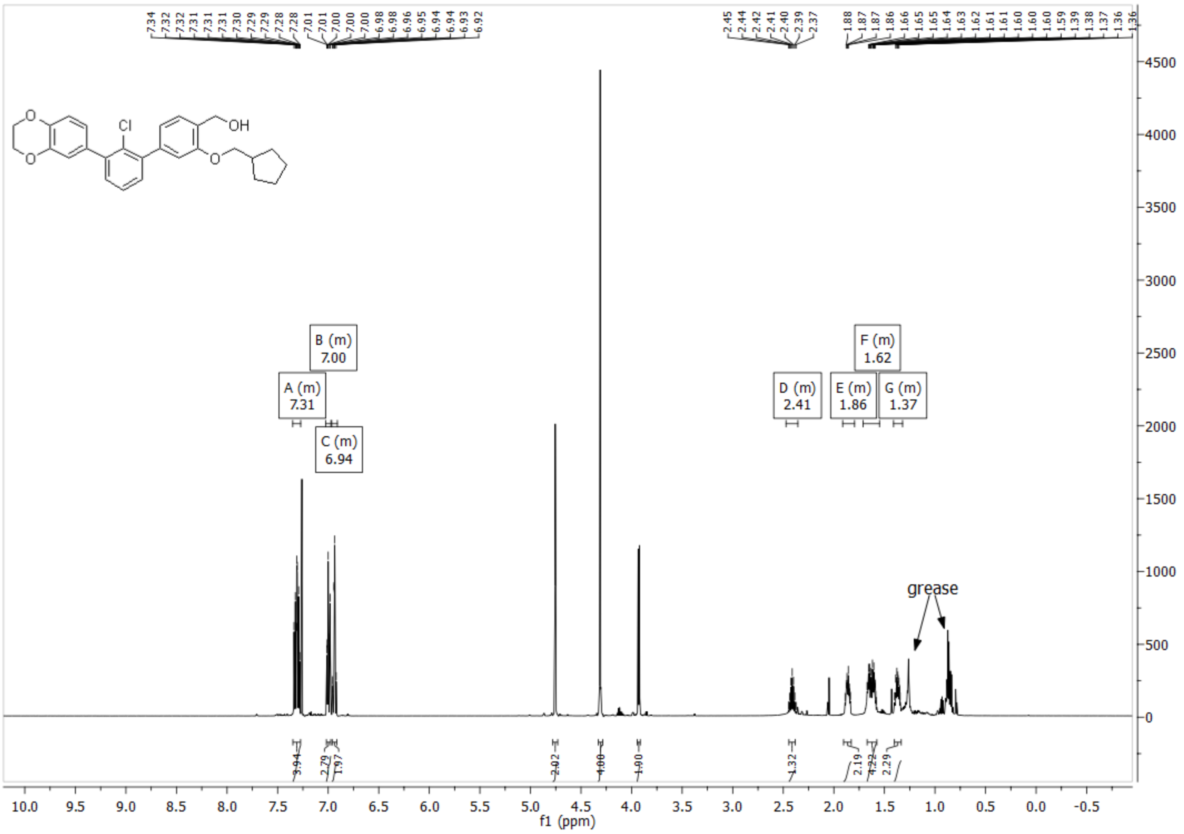

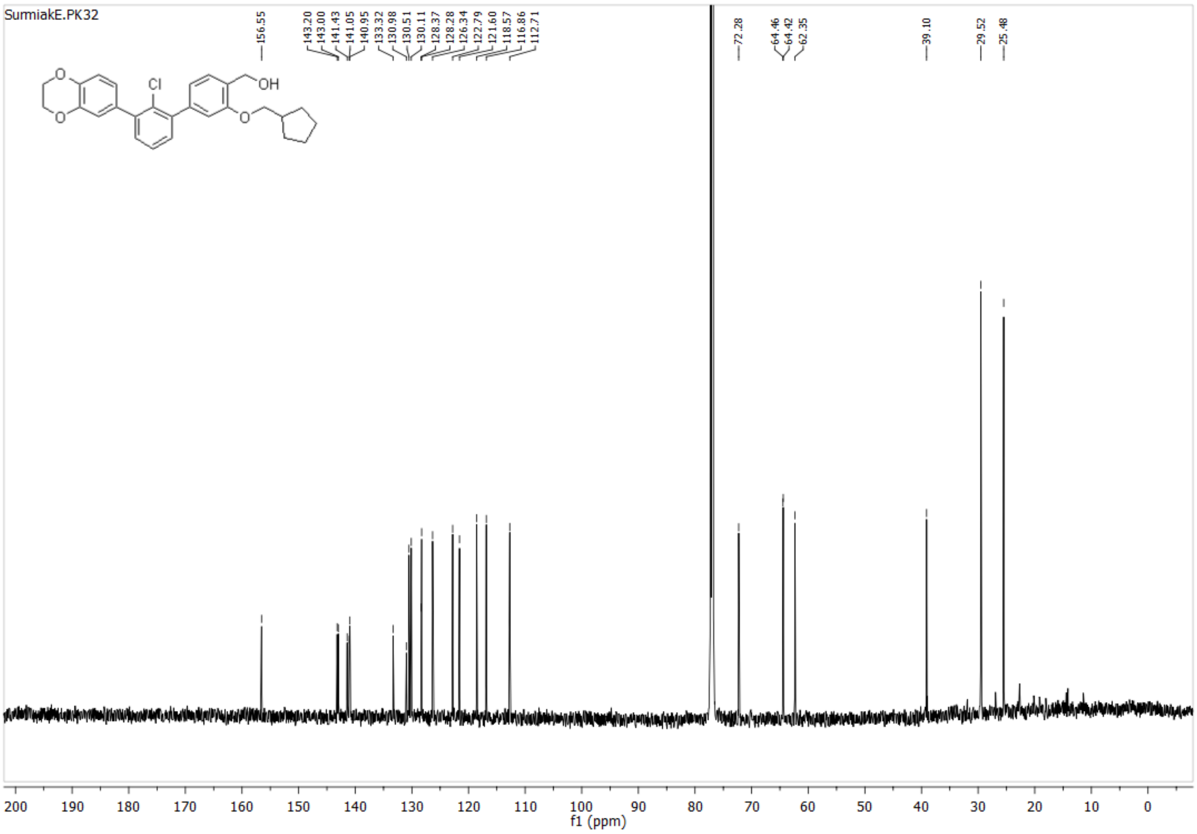

4r. 3'-(((2'-Chloro-3'-(benzo-1,4-dioxan-6-yl)-4-(hydroxymethyl)-[1,1'-biphenyl]-3-yl)oxy)methyl)-[1,1'-biphenyl]-3-carbonitrile

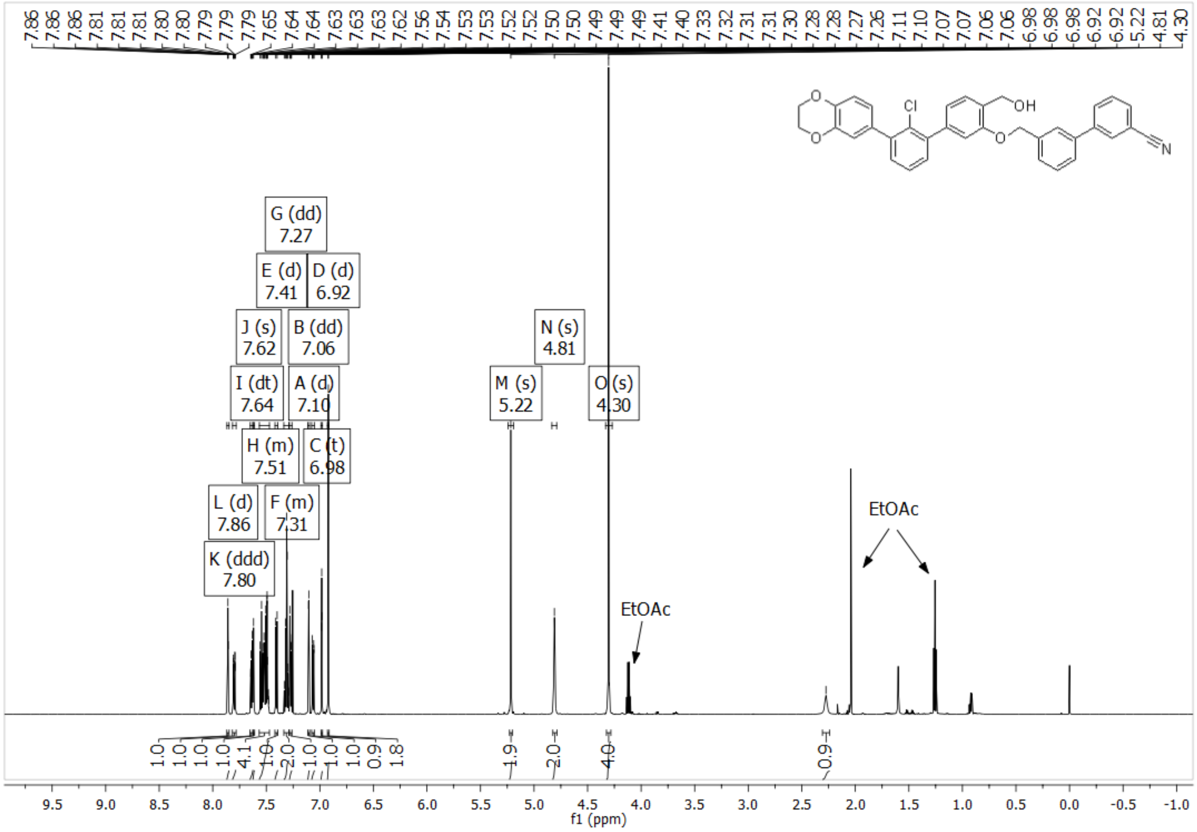

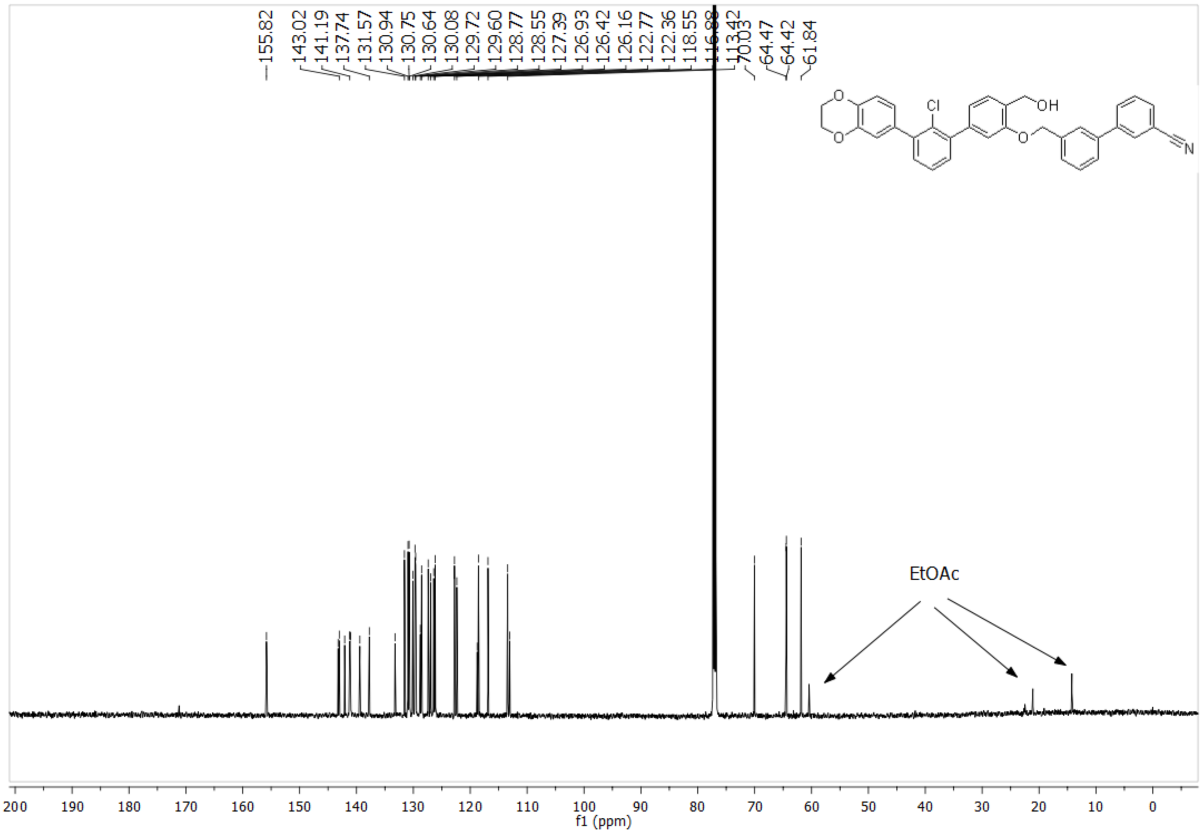

5a. methyl 3-((3'-(benzo-1,4-dioxan-6-yl)-2'-methyl-[1,1'-biphenyl]-4-yl)methoxy)benzoate

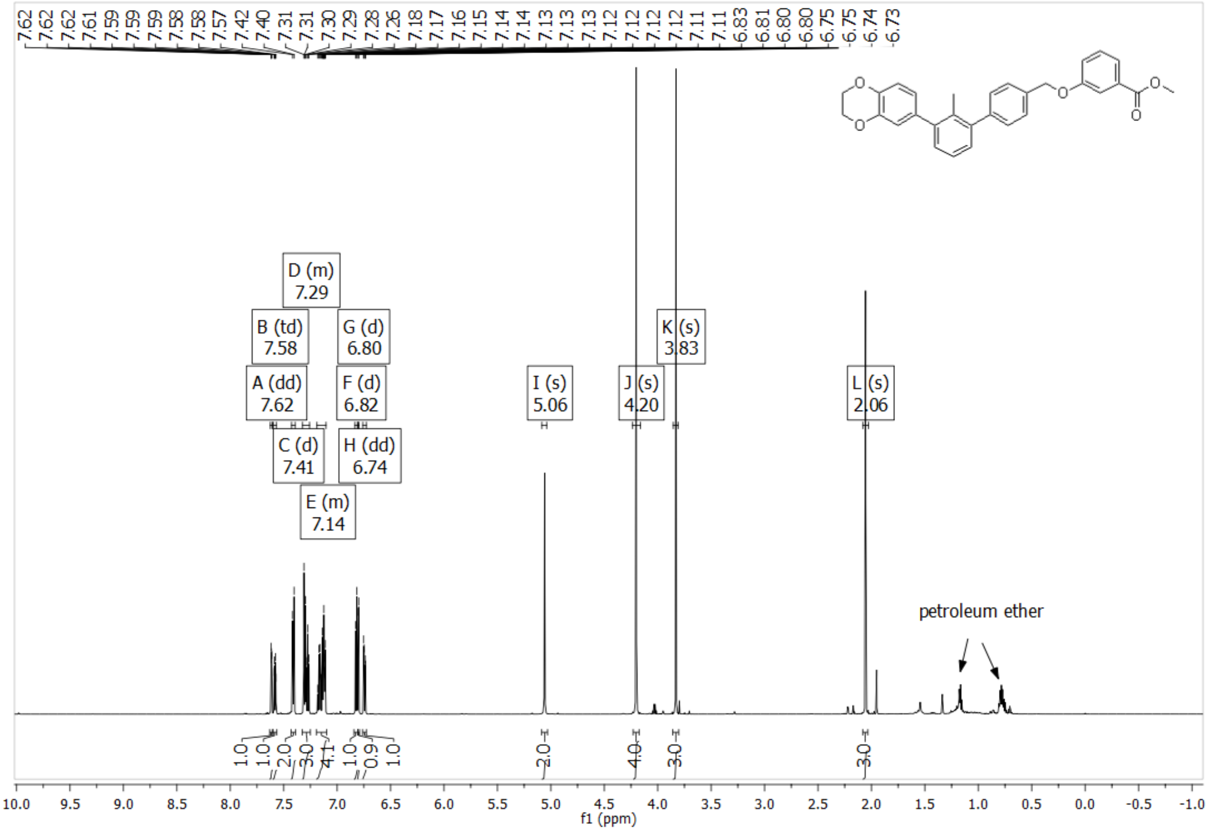

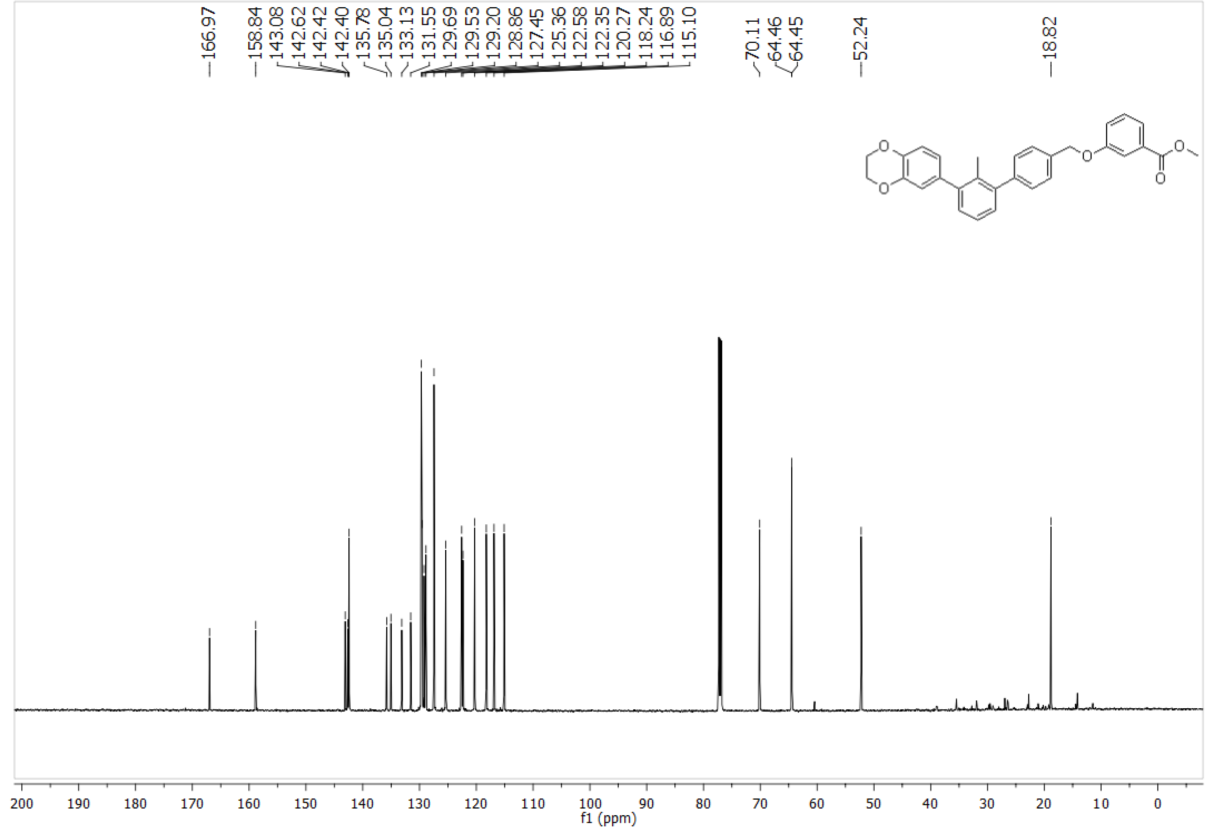

5b. 3-((3'-(benzo-1,4-dioxan-6-yl)-2'-methyl-[1,1'-biphenyl]-4-yl)methoxy)benzoic acid

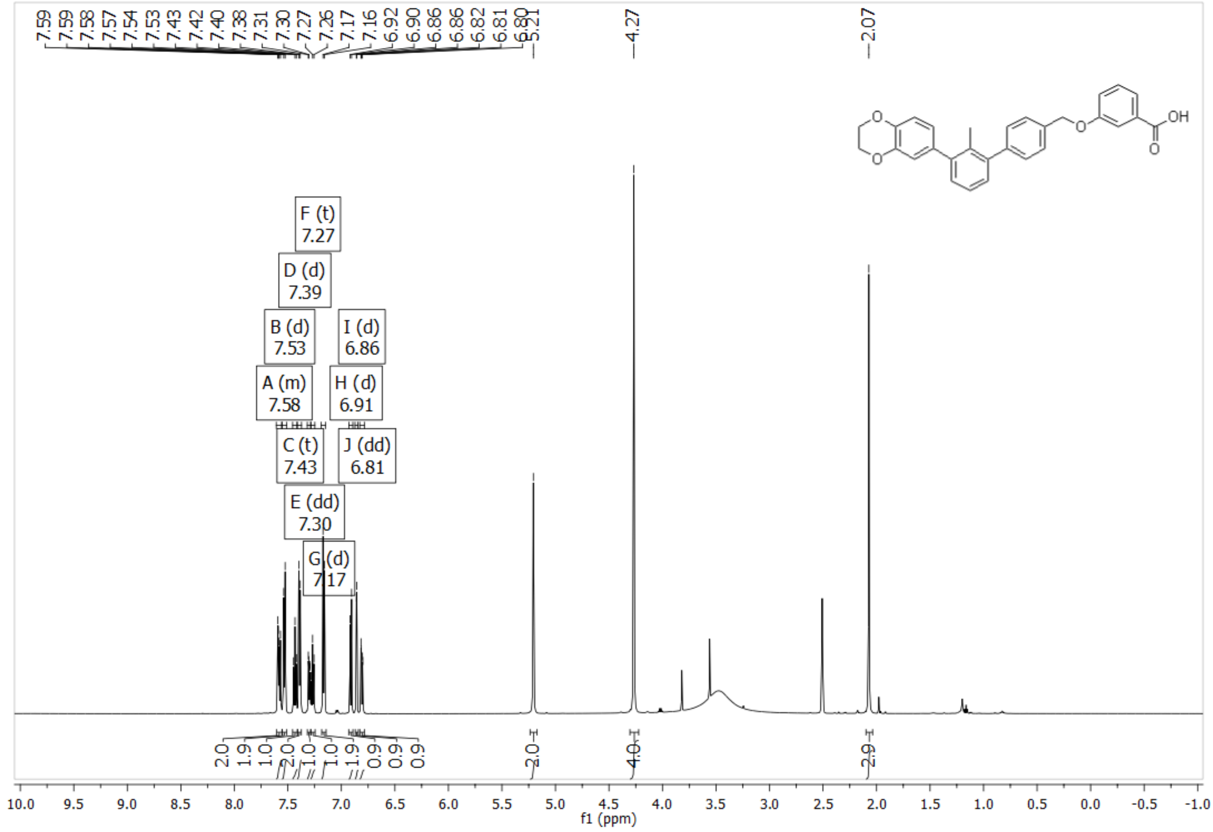

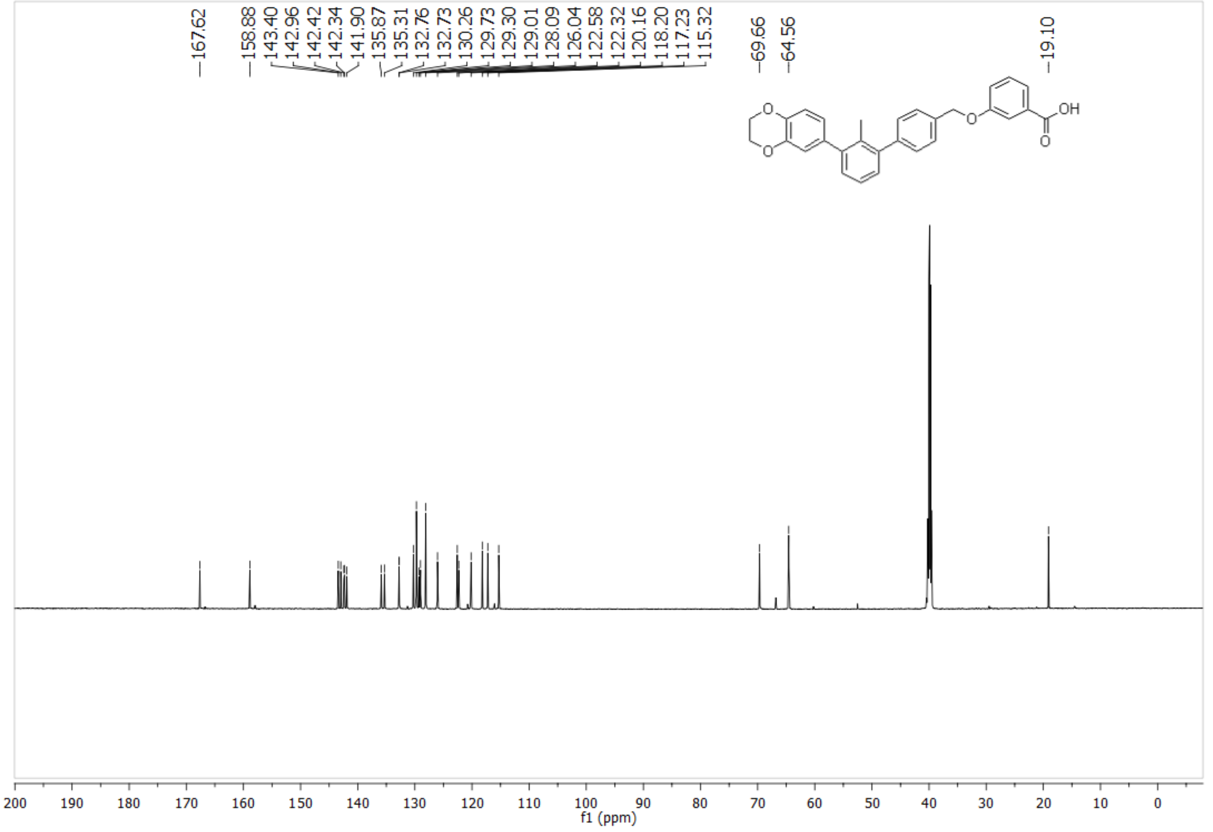

5c. 2-Bromo-5-((3'-(benzo-1,4-dioxan-6-yl)-2'-methyl-[1,1'-biphenyl]-4-yl)methoxy)benzaldehyde

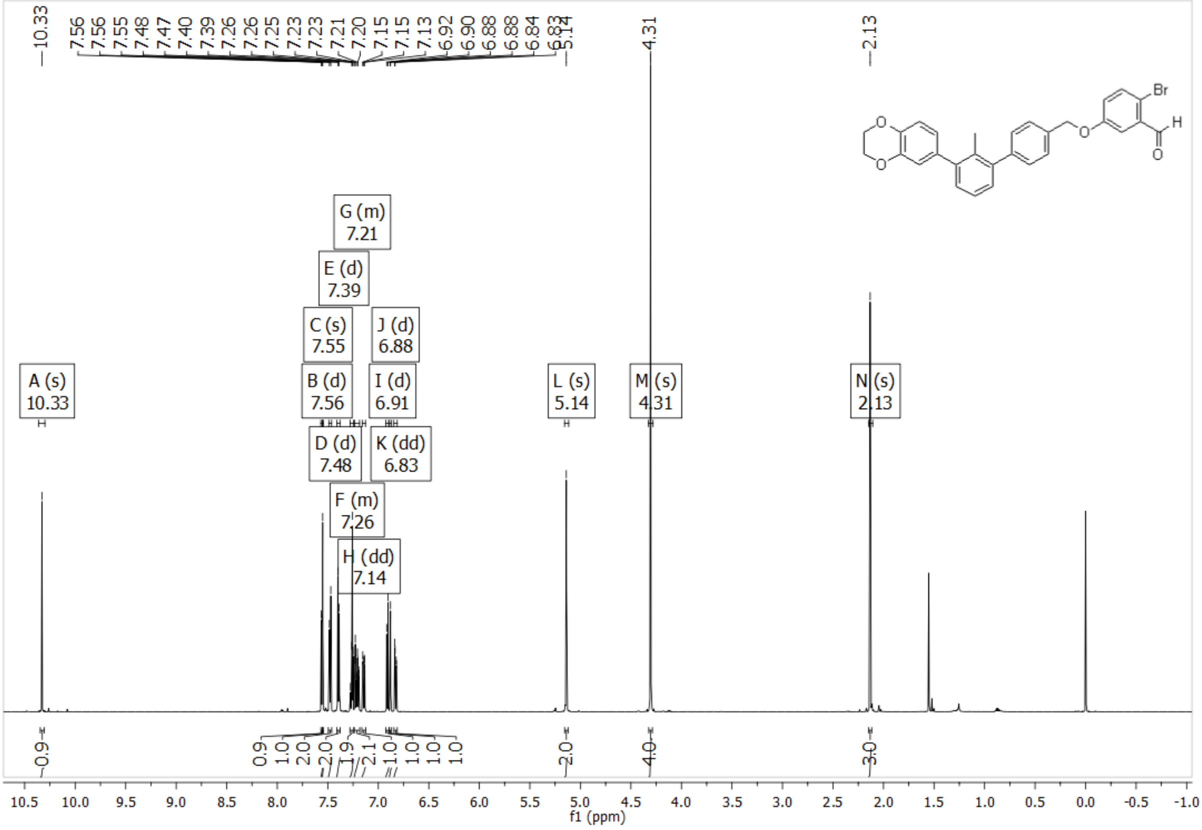

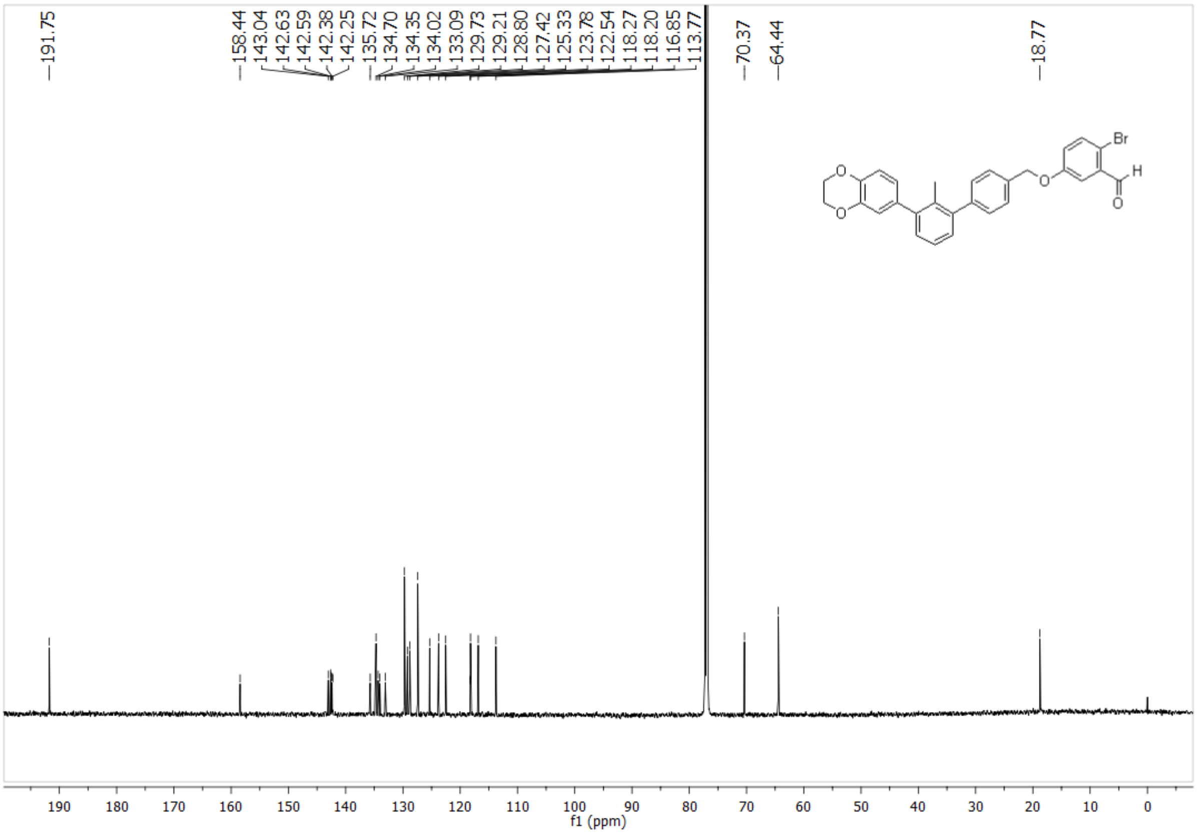

5d. 5-((3'-(benzo-1,4-dioxan-6-yl)-2'-methyl-[1,1'-biphenyl]-4-yl)methoxy)-2-(pyridin-3-yl)benzaldehyde

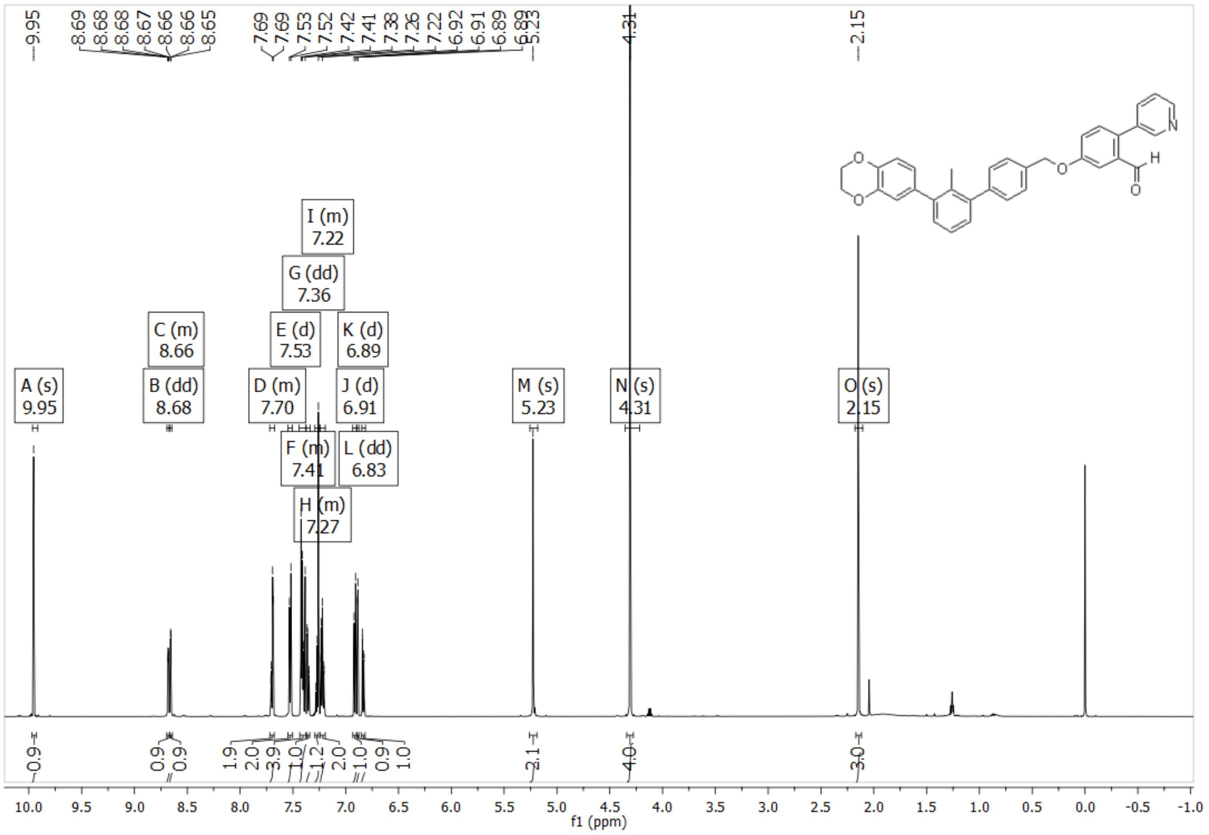

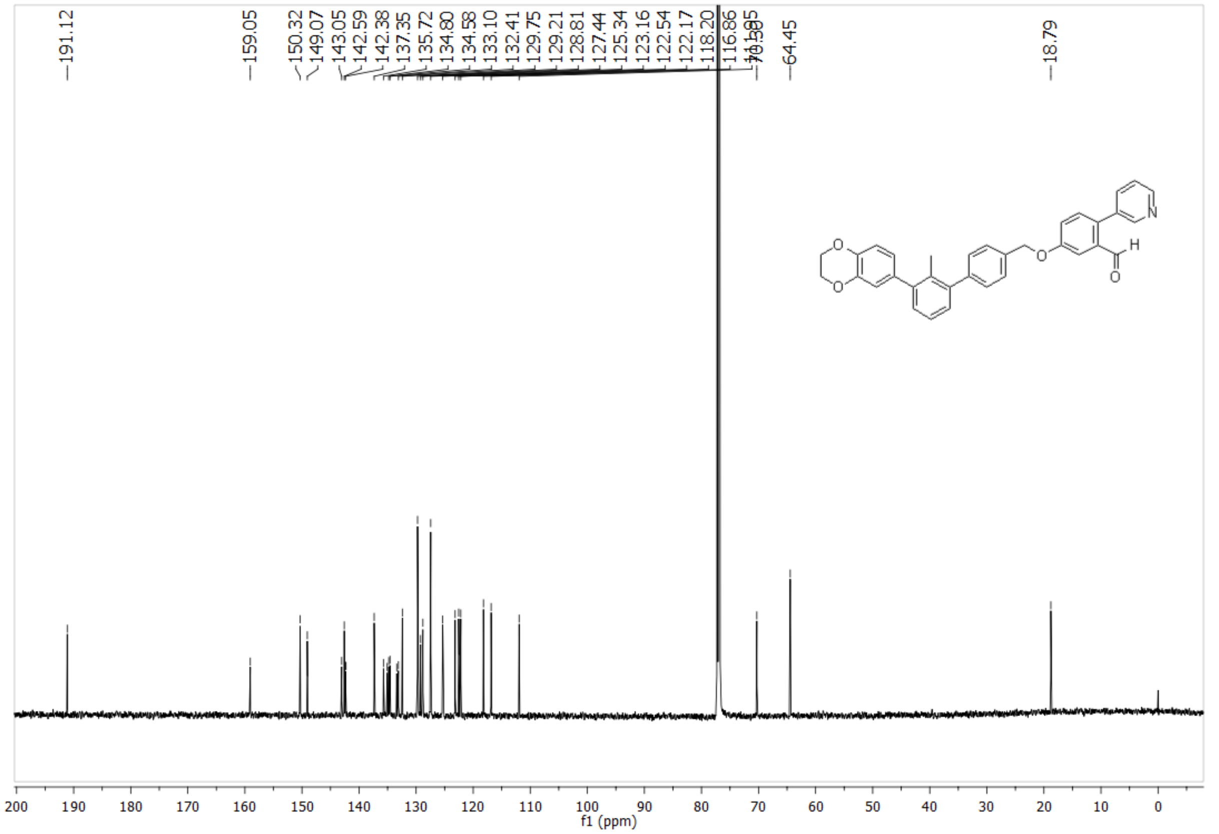

5e. methyl 3-((3'-(benzo-1,4-dioxan-6-yl)-2'-methyl-[1,1'-biphenyl]-4-yl)methoxy)isoxazole-5-carboxylate

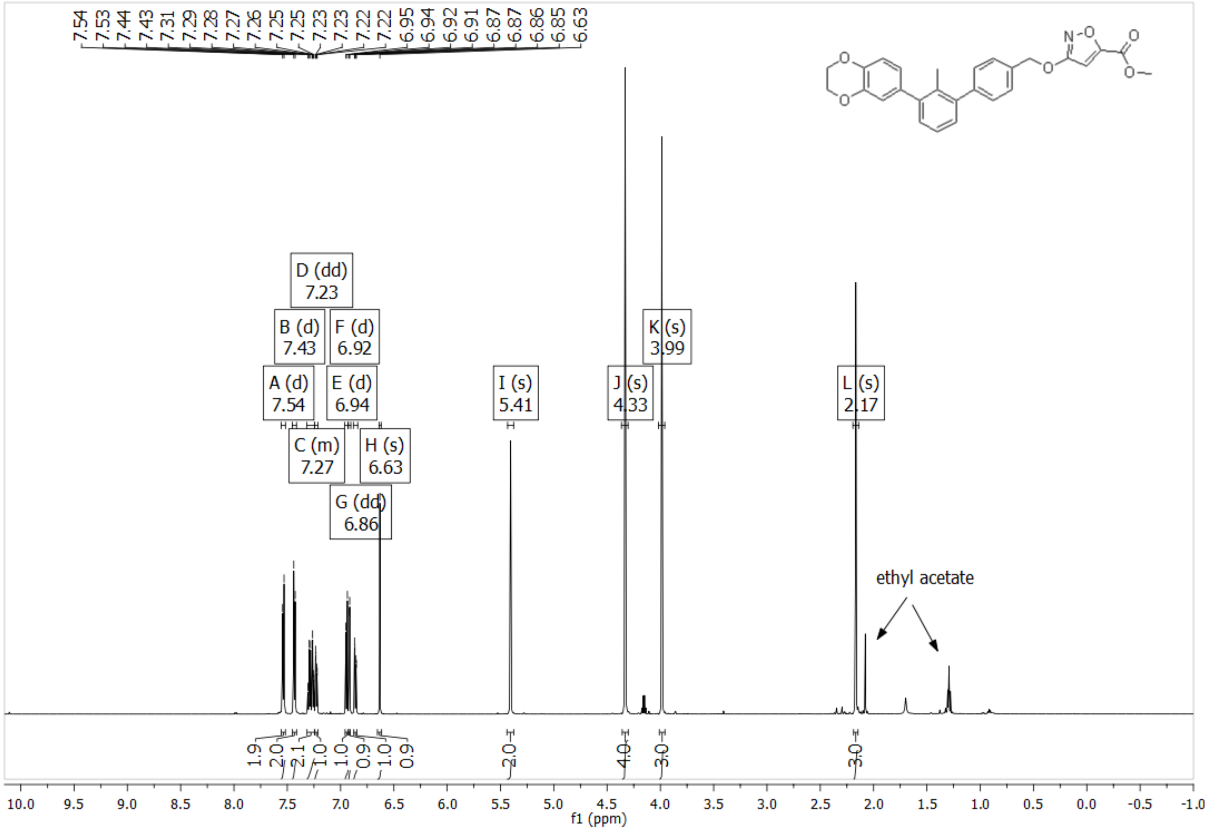

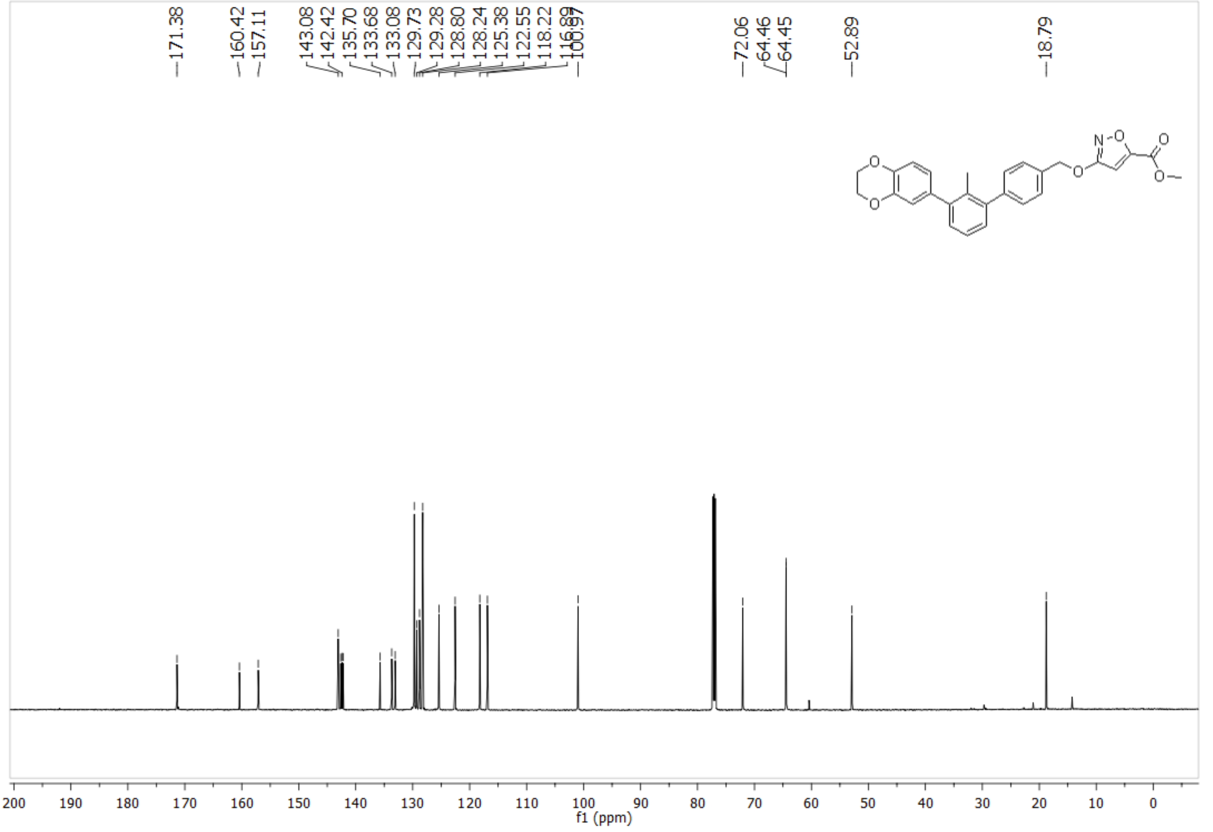

5f. 3-((3'-(benzo-1,4-dioxan-6-yl)-2'-methyl-[1,1'-biphenyl]-4-yl)methoxy)isoxazole-5-carboxylic acid

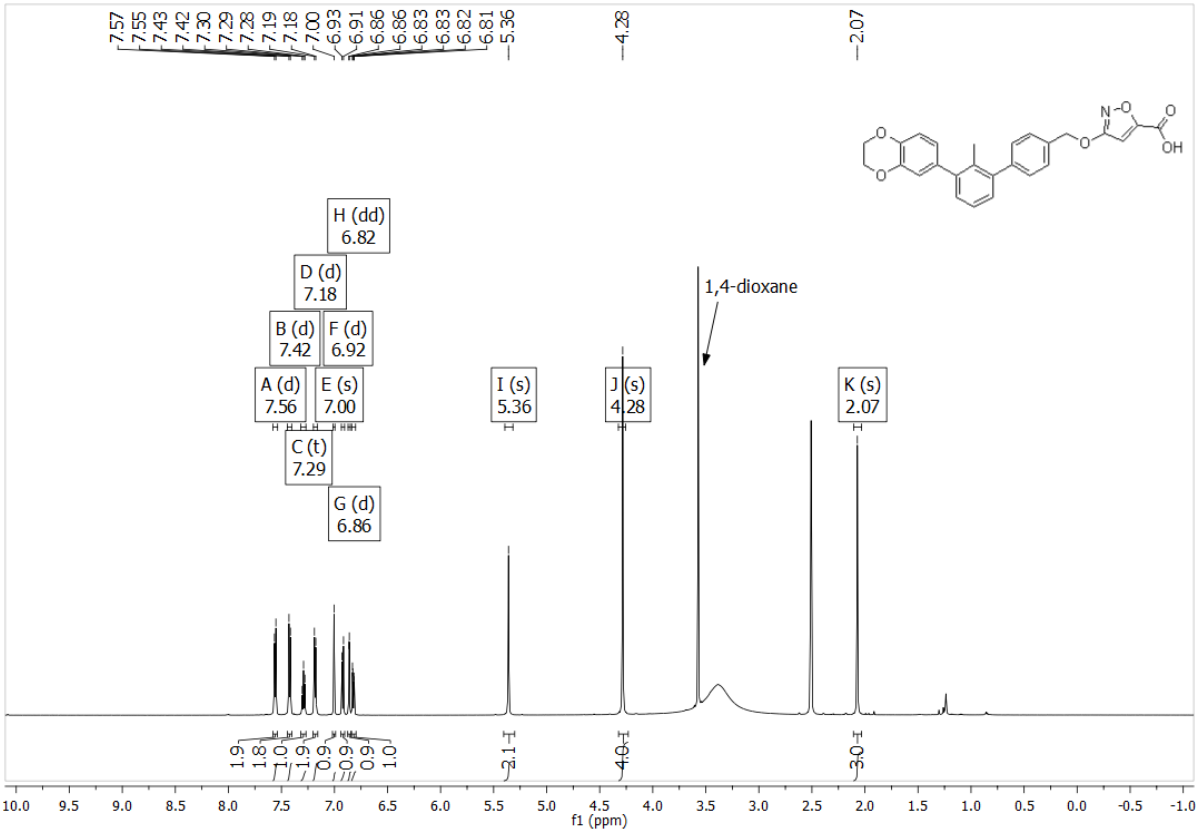

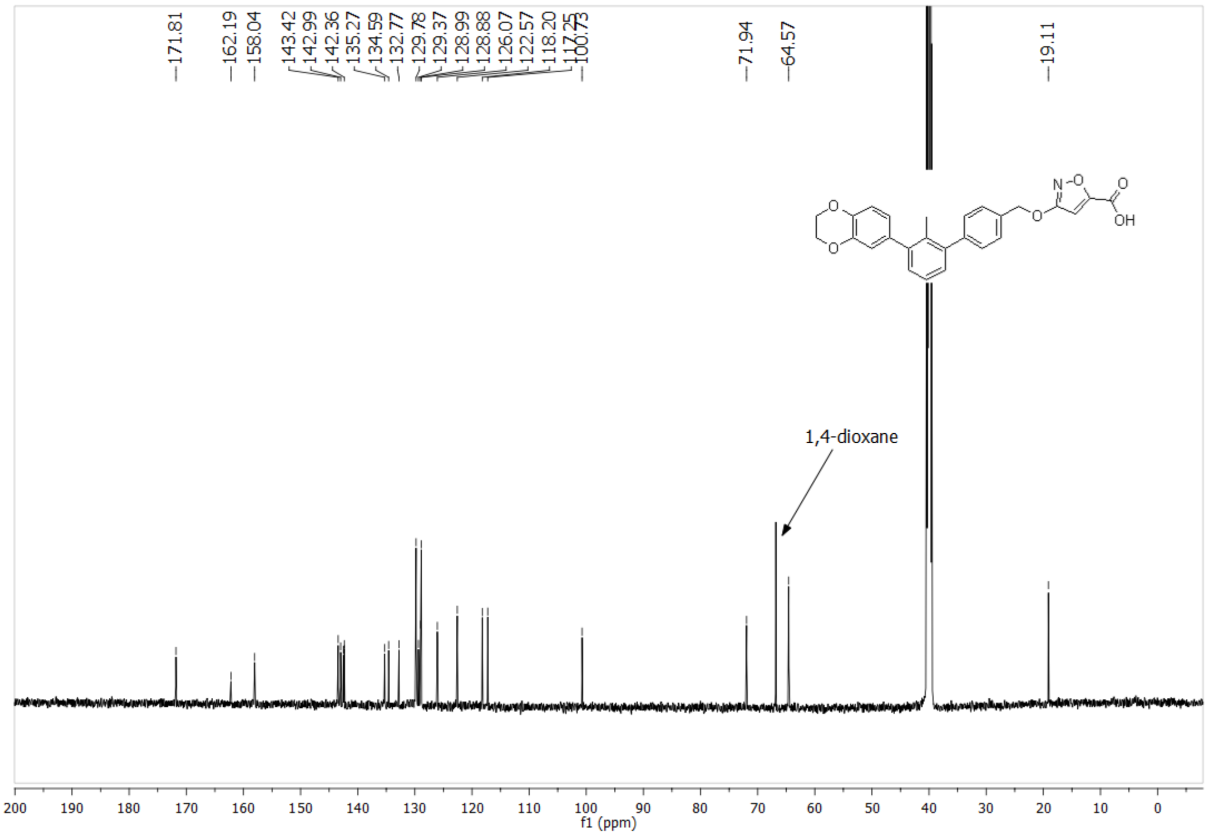

5g. Methyl 1-((3'-(benzo-1,4-dioxan-6-yl)-2'-methyl-[1,1'-biphenyl]-4-yl)methyl)-1H-1,2,3-triazole-4-carboxylate

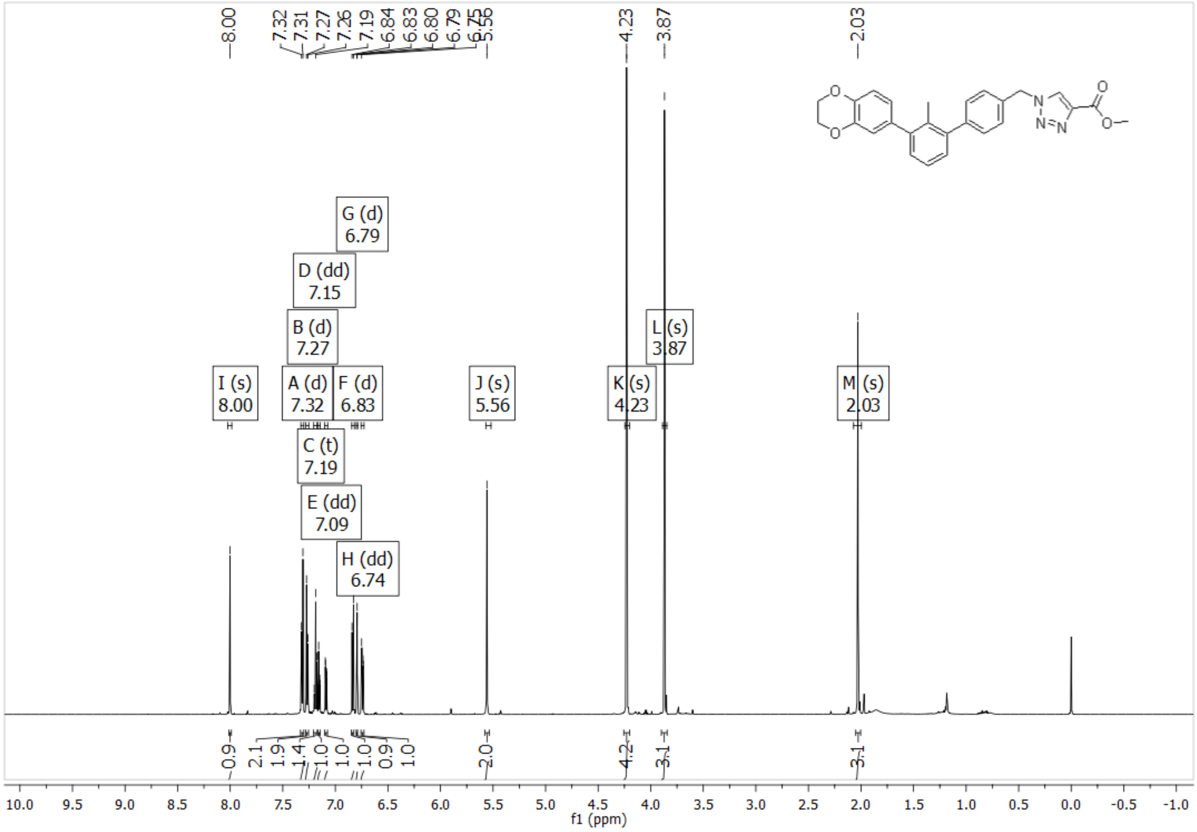

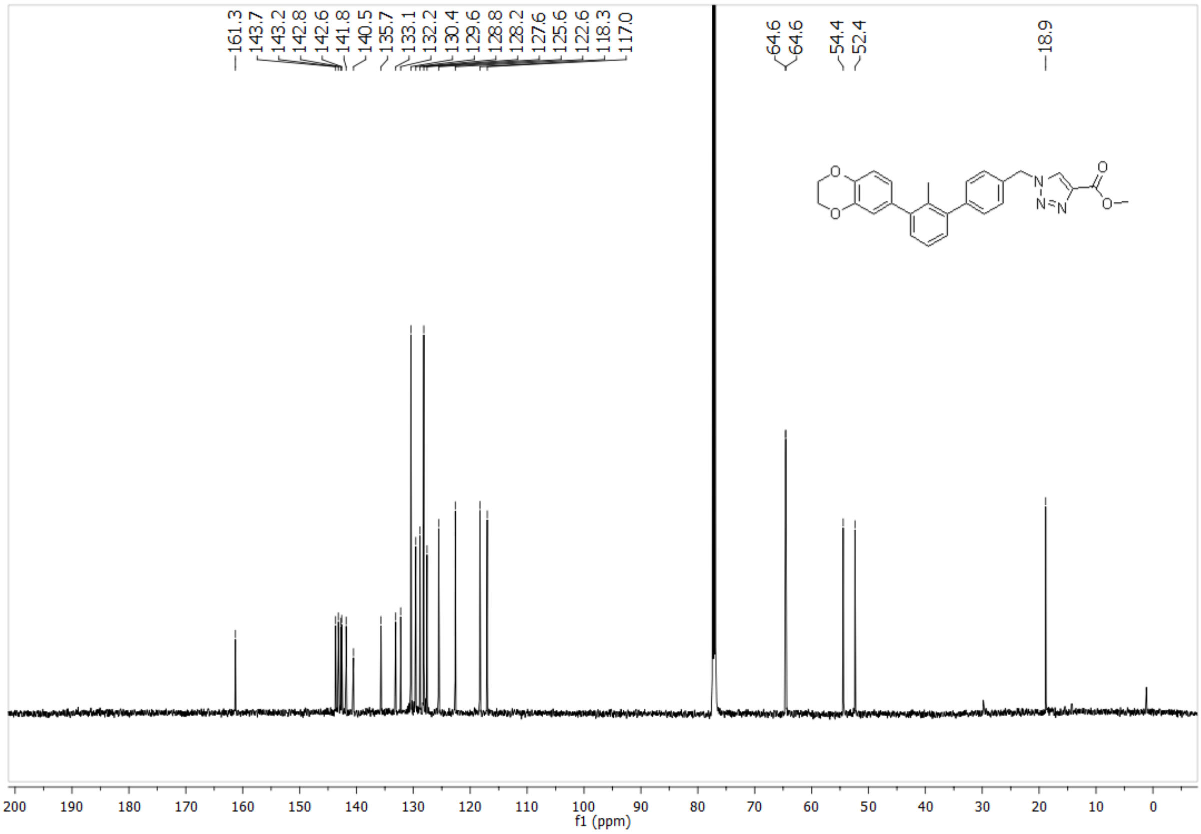

5h. 1-((3'-(benzo-1,4-dioxan-6-yl)-2'-methyl-[1,1'-biphenyl]-4-yl)methyl)-1H-1,2,3-triazole-4-carboxylic acid

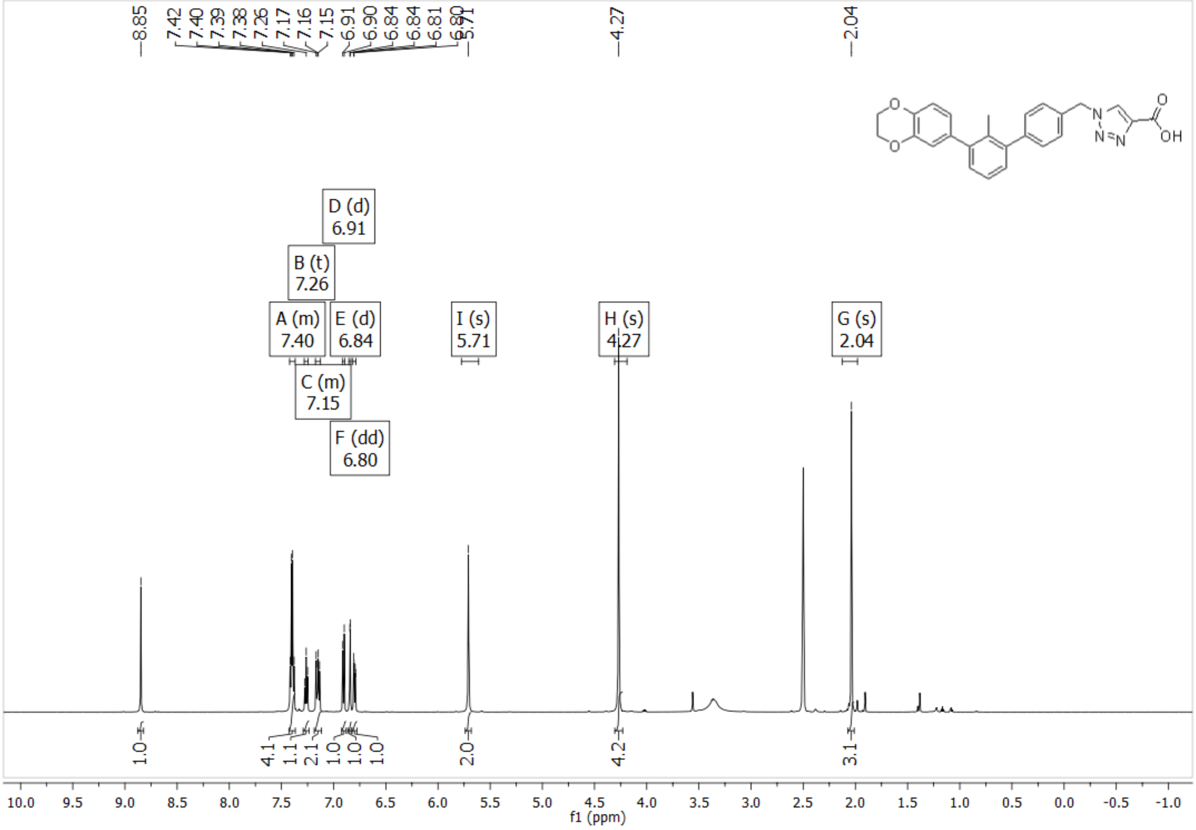

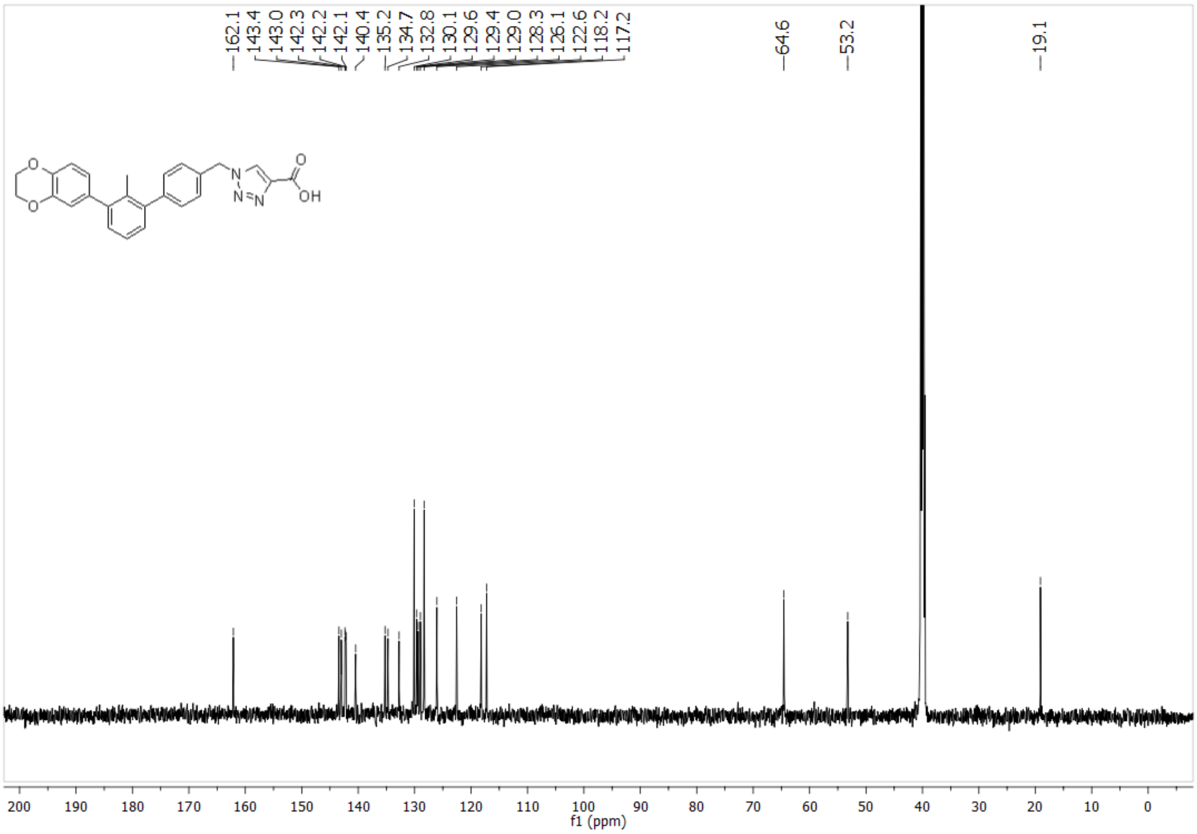

5i. methyl 3-((3'-(benzo-1,4-dioxan-6-yl)-2'-methyl-[1,1'-biphenyl]-3-yl)methoxy)benzoate

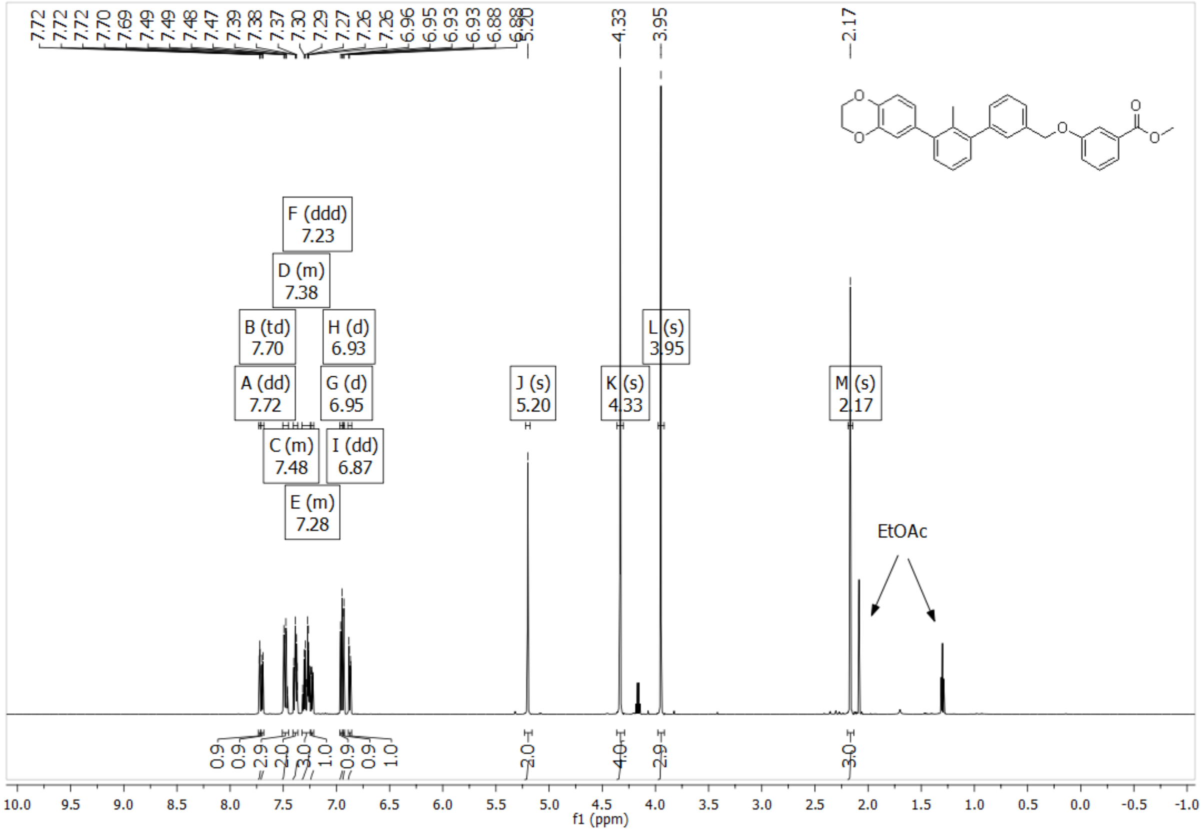

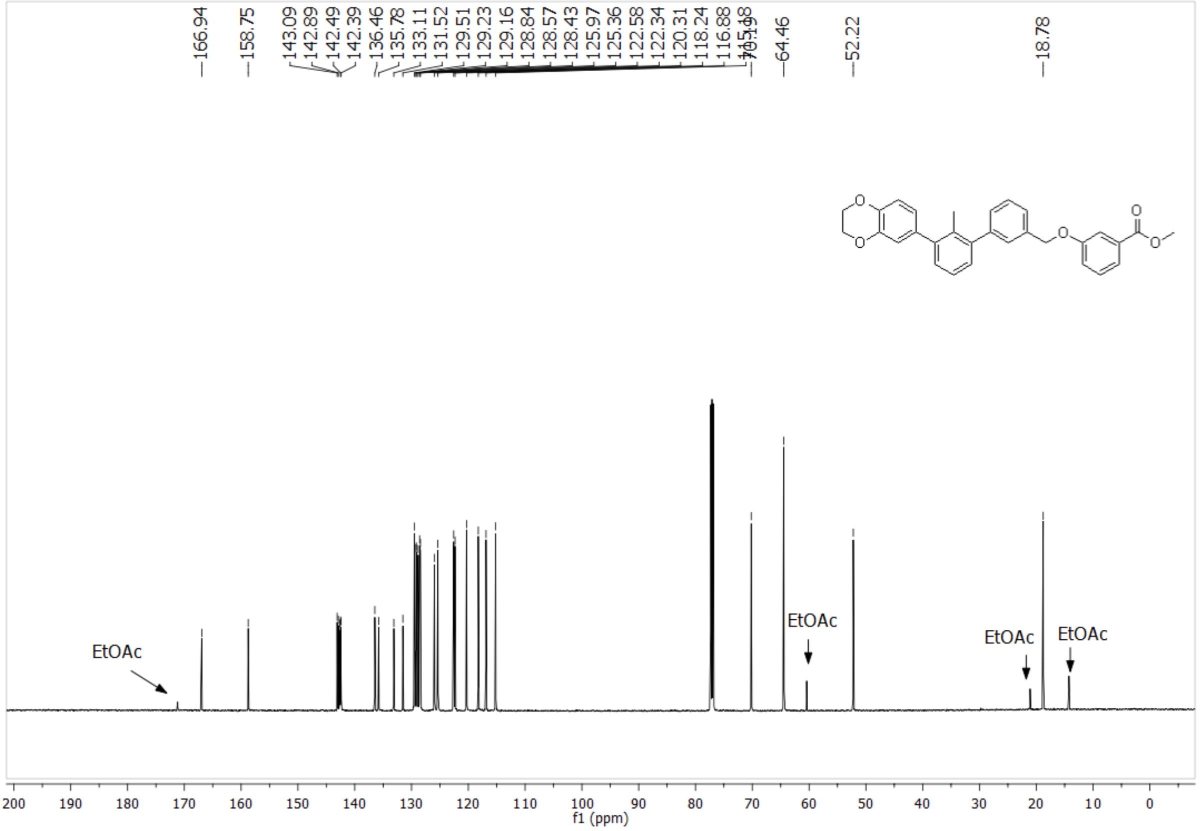

5j. 3-((3'-(benzo-1,4-dioxan-6-yl)-2'-methyl-[1,1'-biphenyl]-3-yl)methoxy)benzoic acid

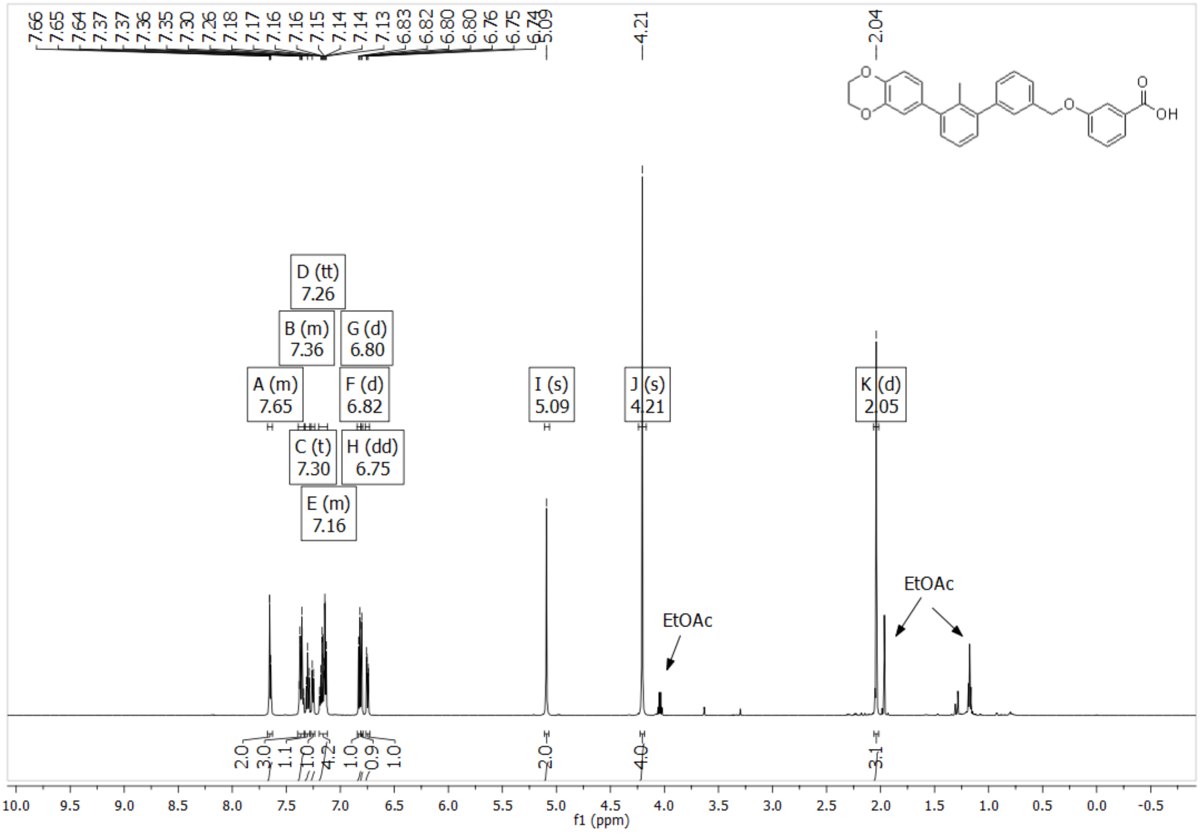

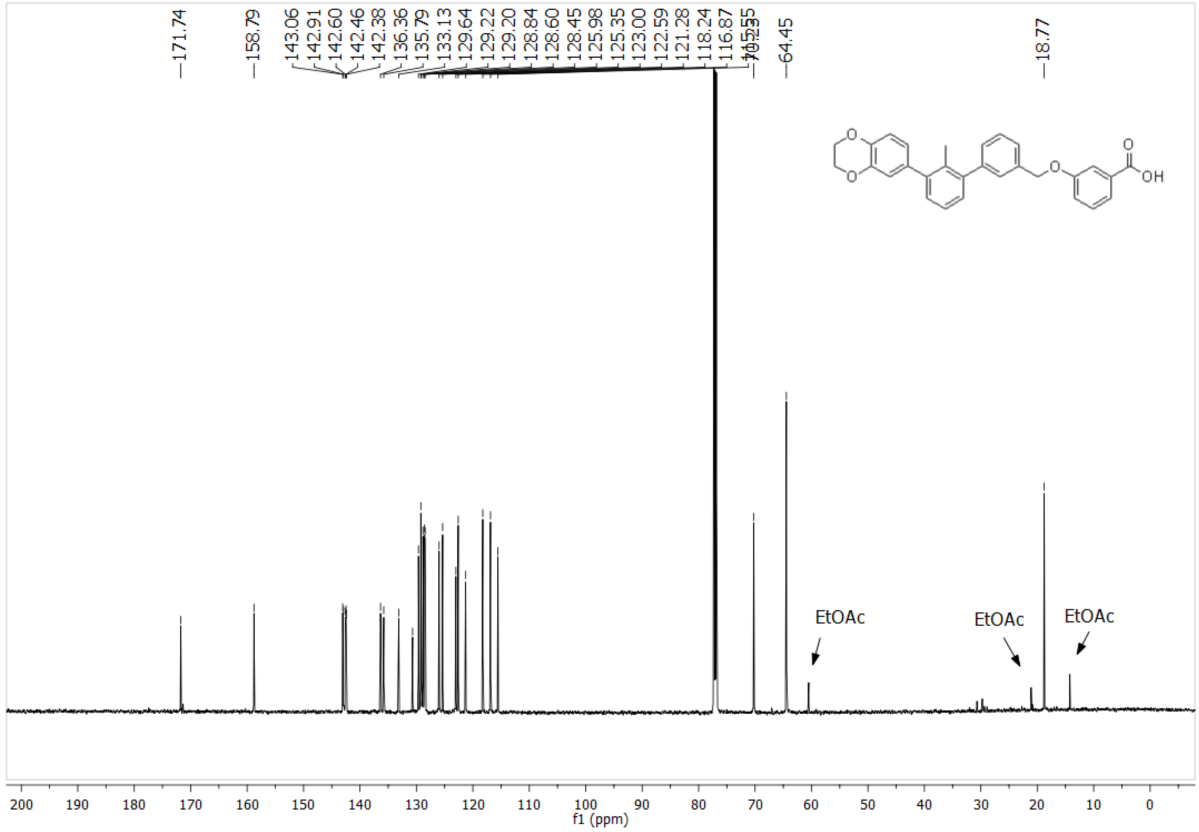

5k. 2-bromo-5-((3'-(benzo-1,4-dioxan-6-yl)-2'-methyl-[1,1'-biphenyl]-3-yl)methoxy)benzaldehyde

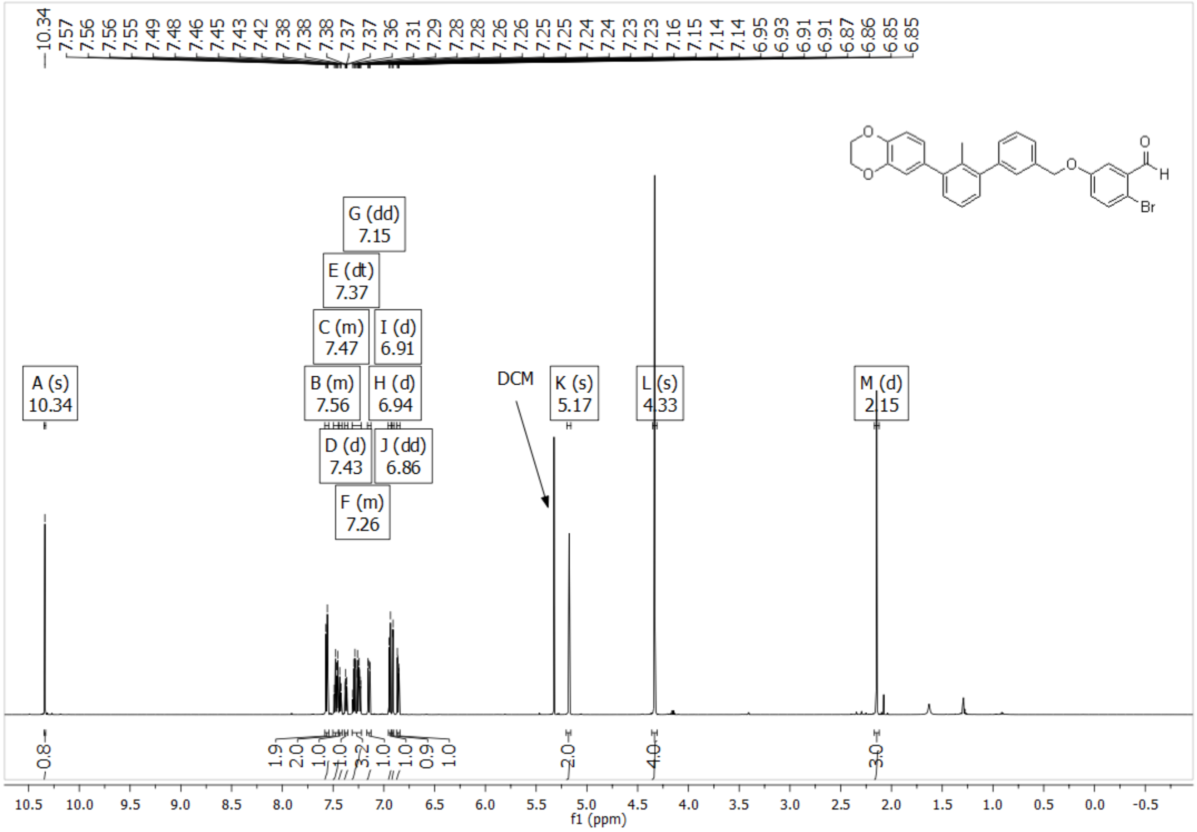

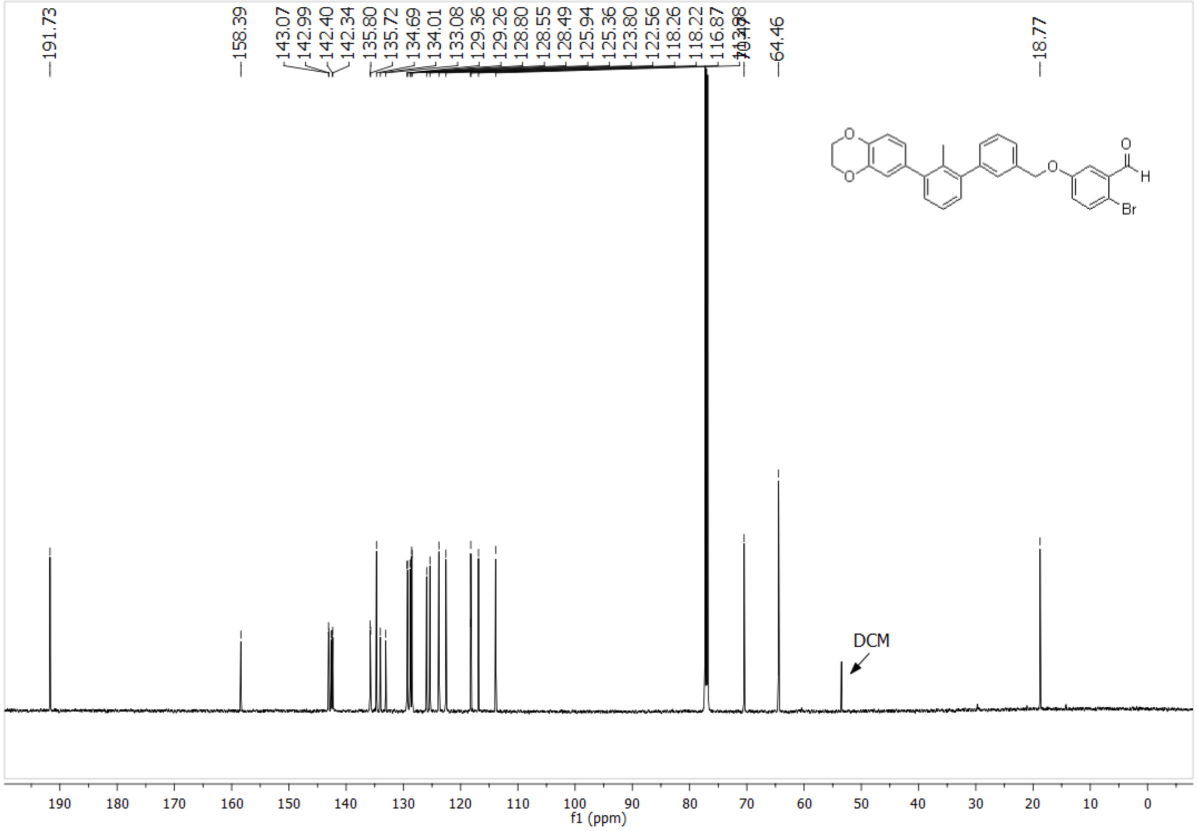

5l. 5-((3'-(benzo-1,4-dioxan-6-yl)-2'-methyl-[1,1'-biphenyl]-3-yl)methoxy)-2-(pyridin-3-yl)benzaldehyde

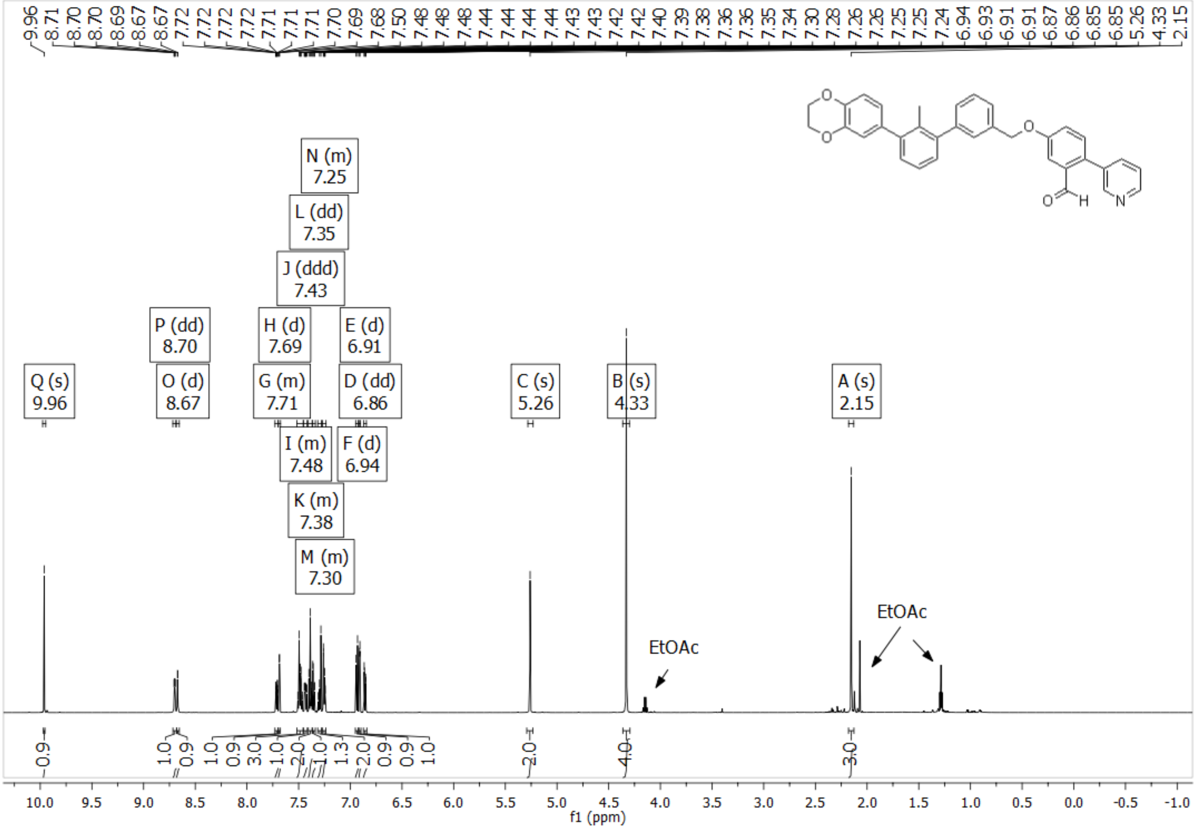

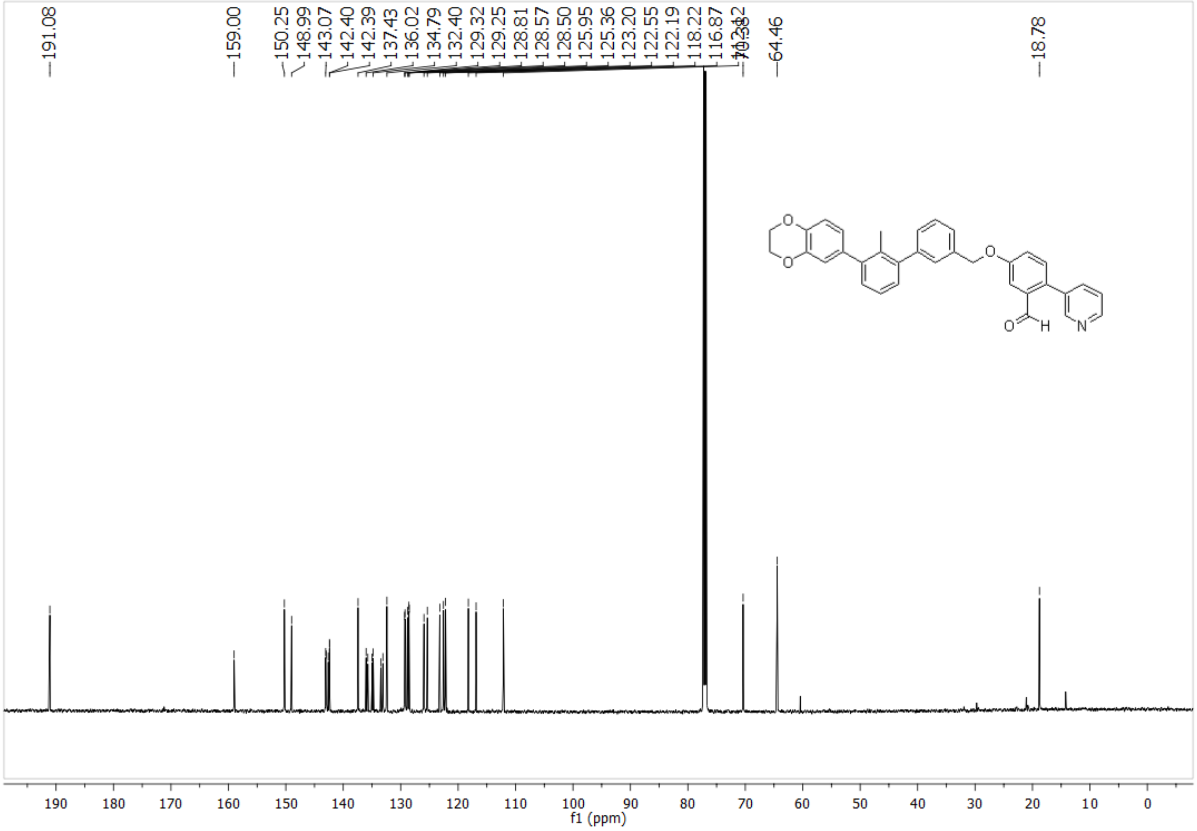

5m. methyl 3-((3'-(benzo-1,4-dioxan-6-yl)-2'-methyl-[1,1'-biphenyl]-3-yl)methoxy)isoxazole-5-carboxylate

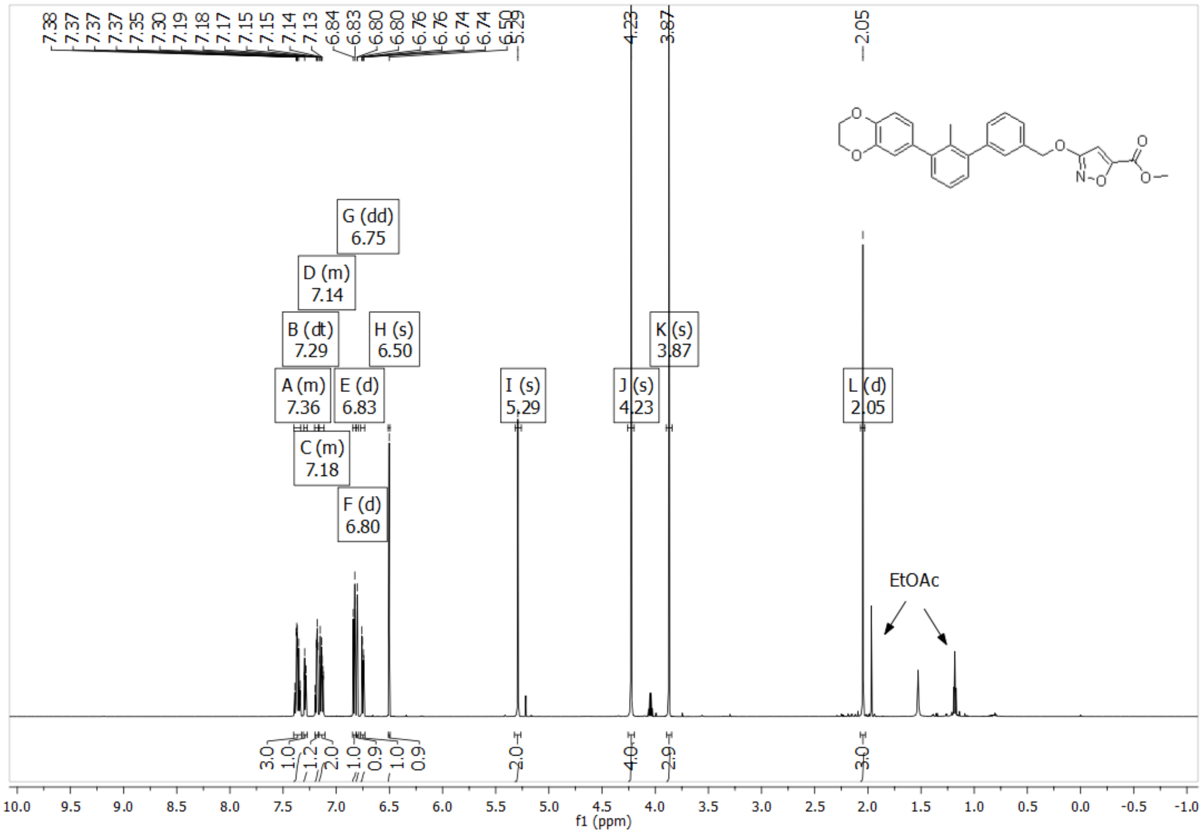

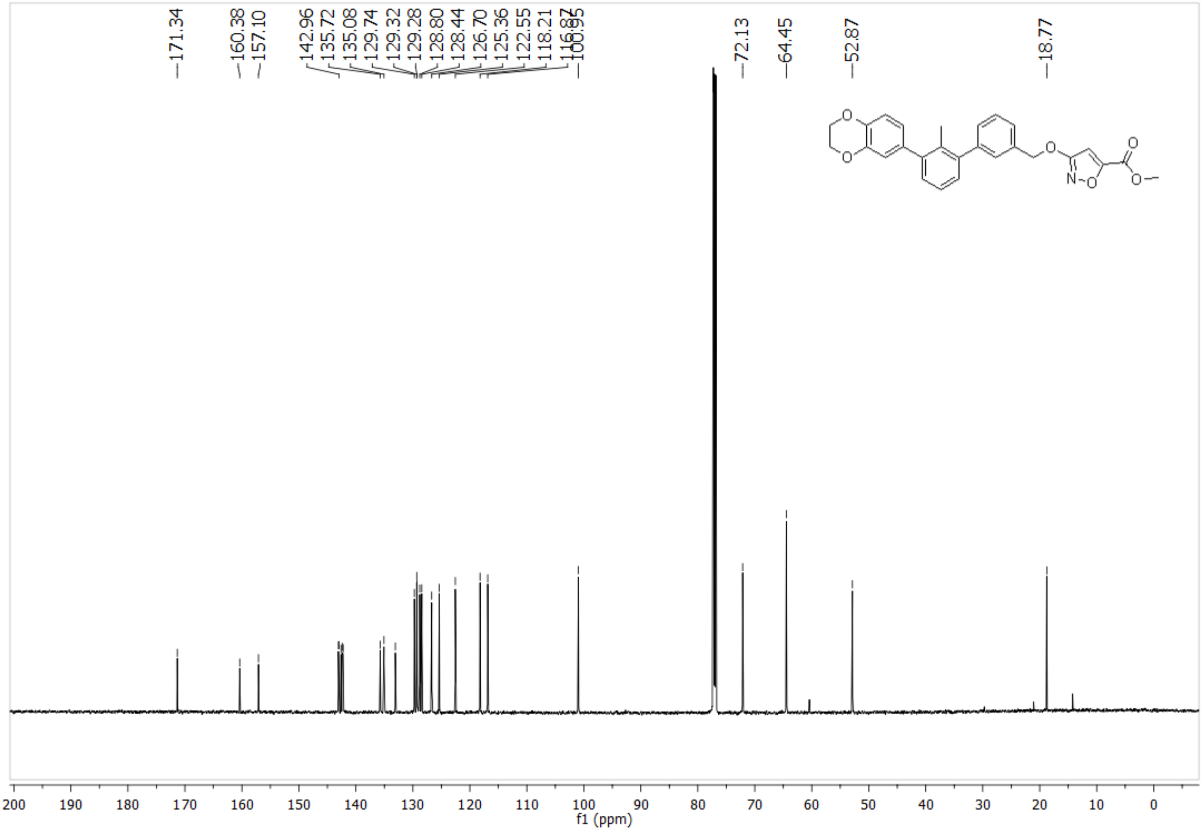

5n. 3-((3'-(benzo-1,4-dioxan-6-yl)-2'-methyl-[1,1'-biphenyl]-3-yl)methoxy)isoxazole-5-carboxylic acid

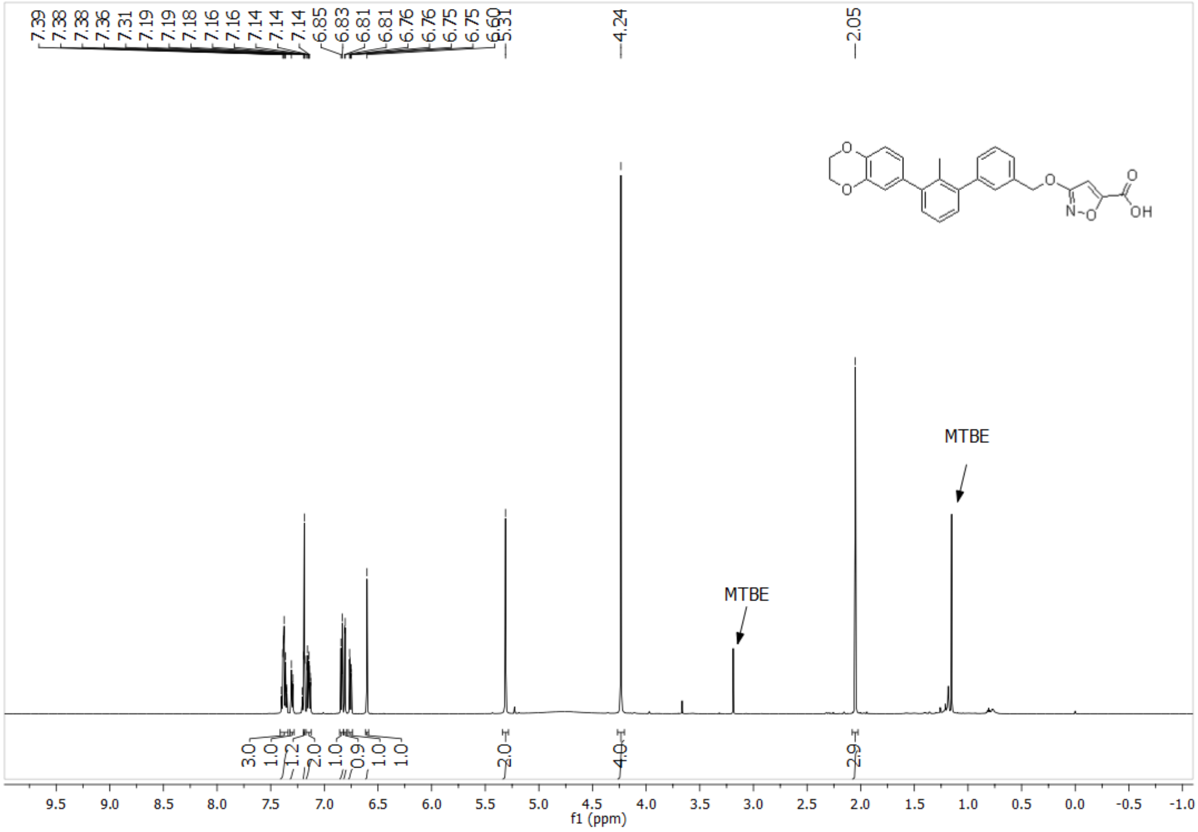

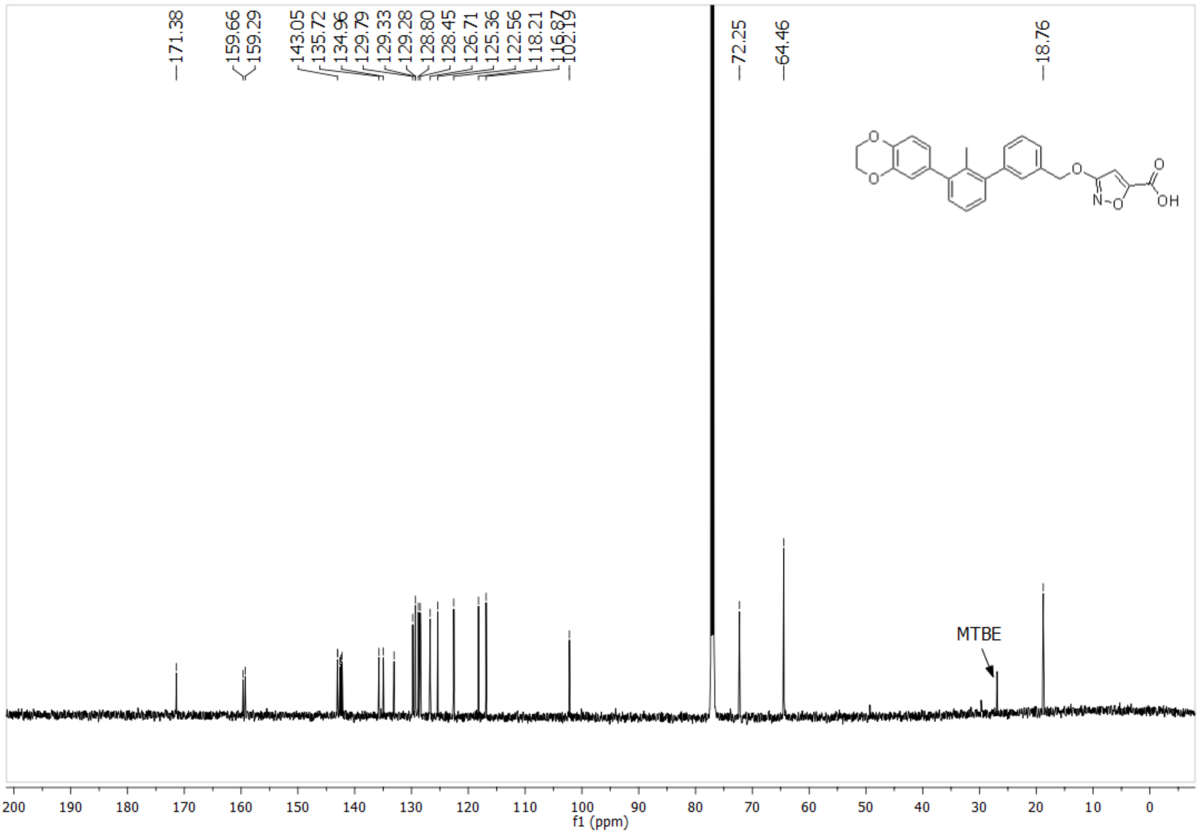

5o. methyl 3-((3'-(benzo-1,4-dioxan-6-yl)-2'-methyl-[1,1'-biphenyl]-2-yl)methoxy)benzoate

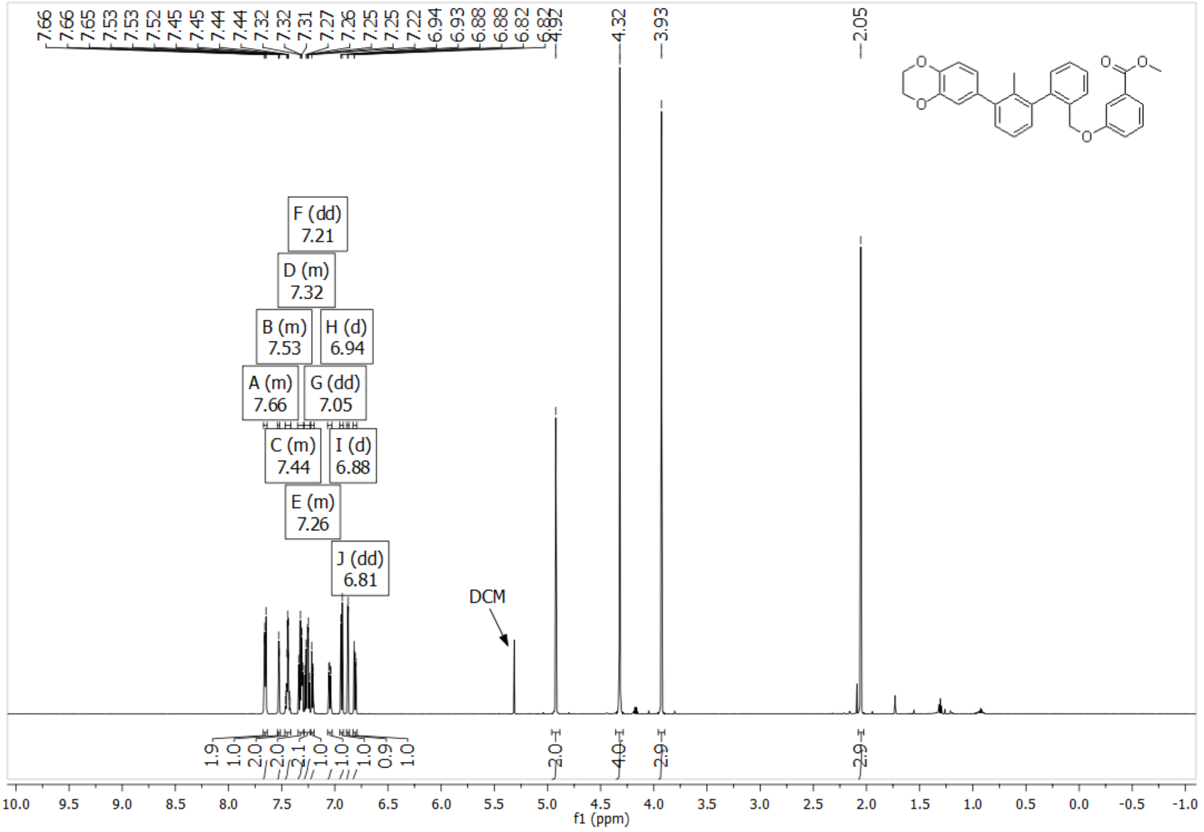

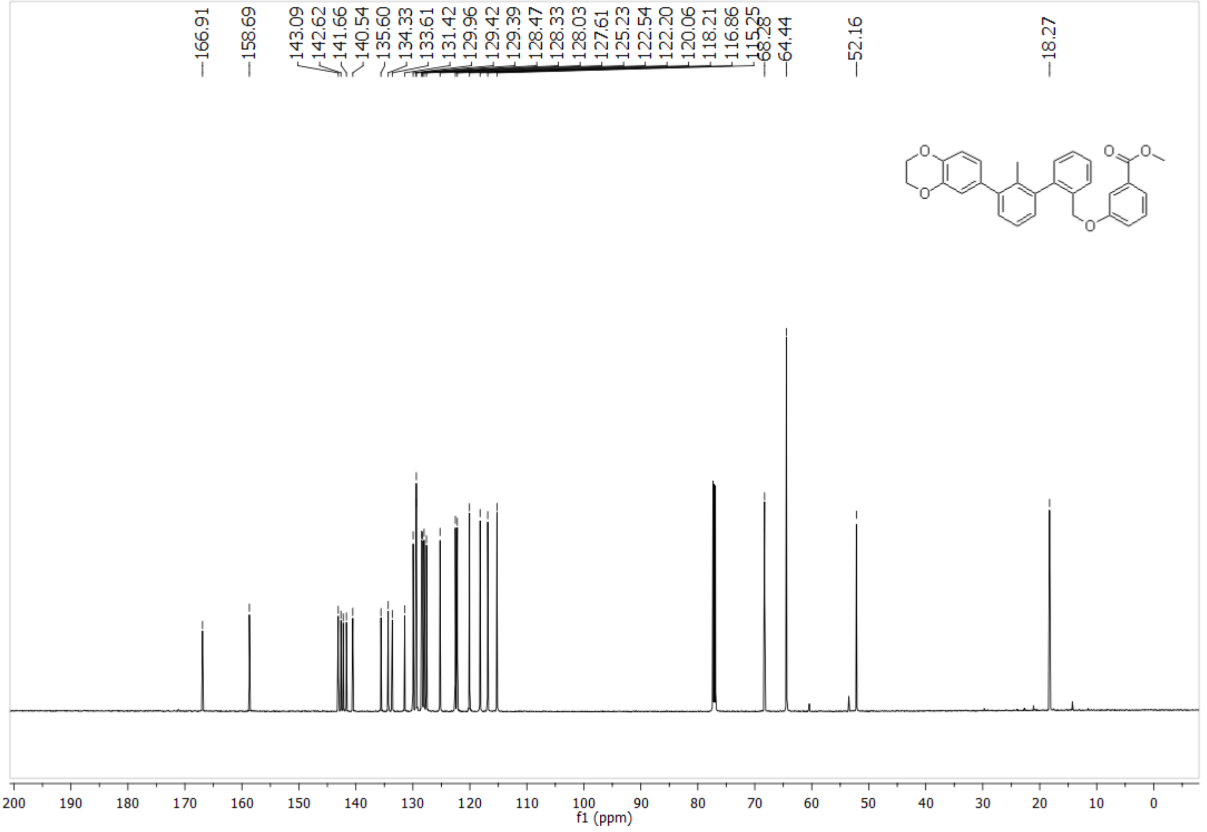

5p. 3-((3'-(benzo-1,4-dioxan-6-yl)-2'-methyl-[1,1'-biphenyl]-2-yl)methoxy)benzoic acid

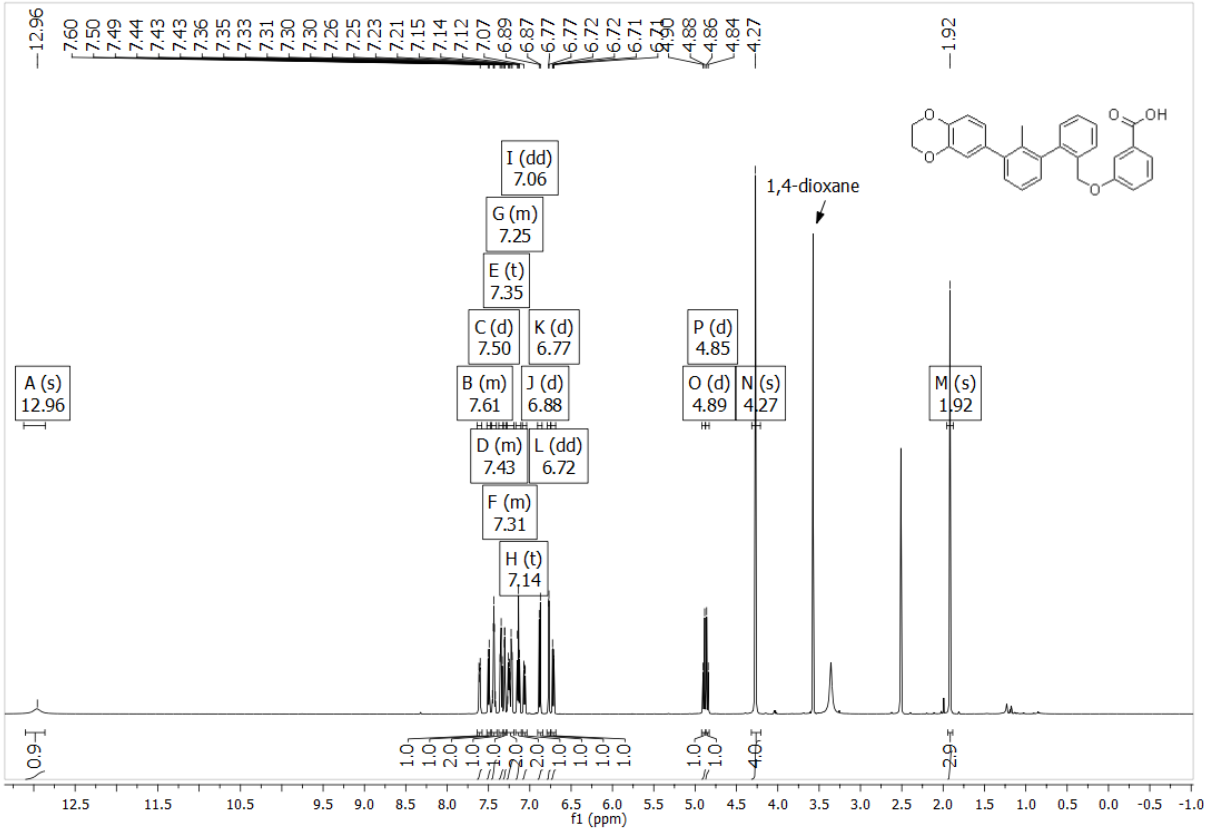

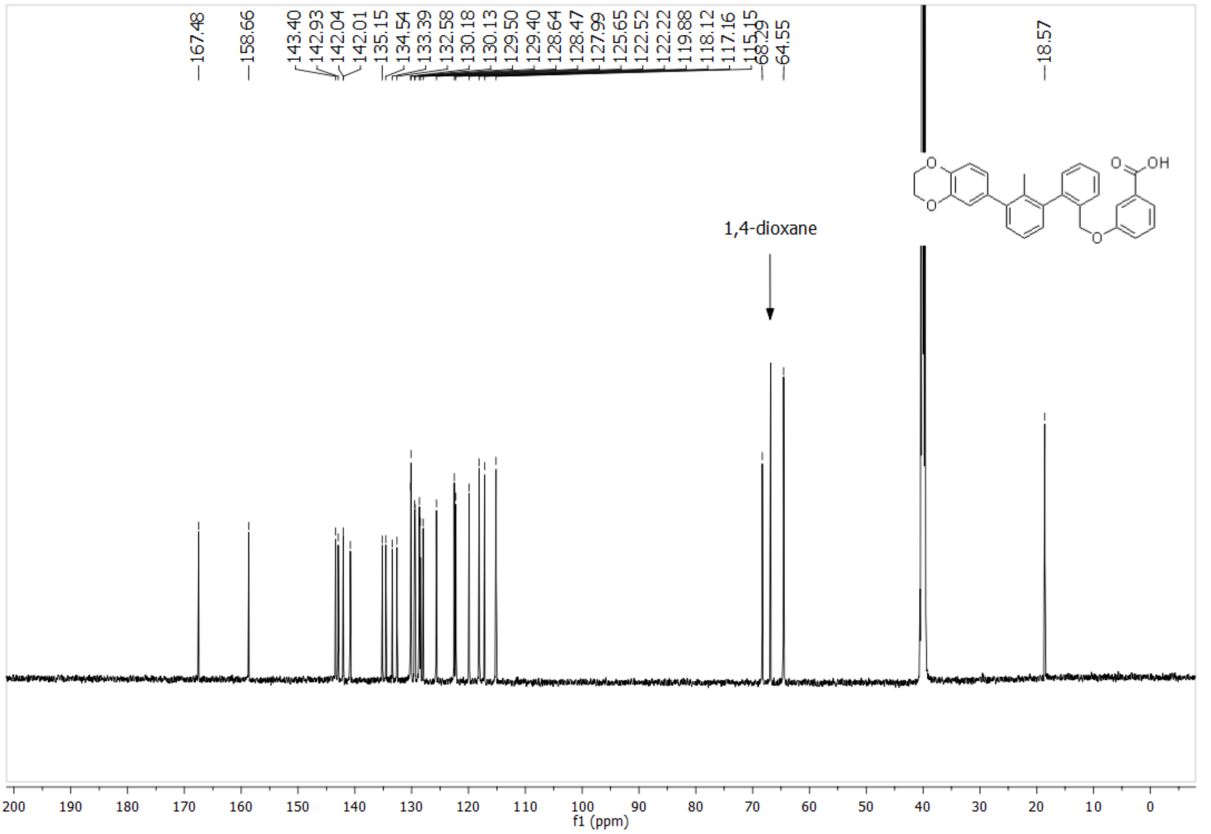

5q. methyl 3-((3'-(benzo-1,4-dioxan-6-yl)-2'-methyl-[1,1'-biphenyl]-2-yl)methoxy)isoxazole-5-carboxylate

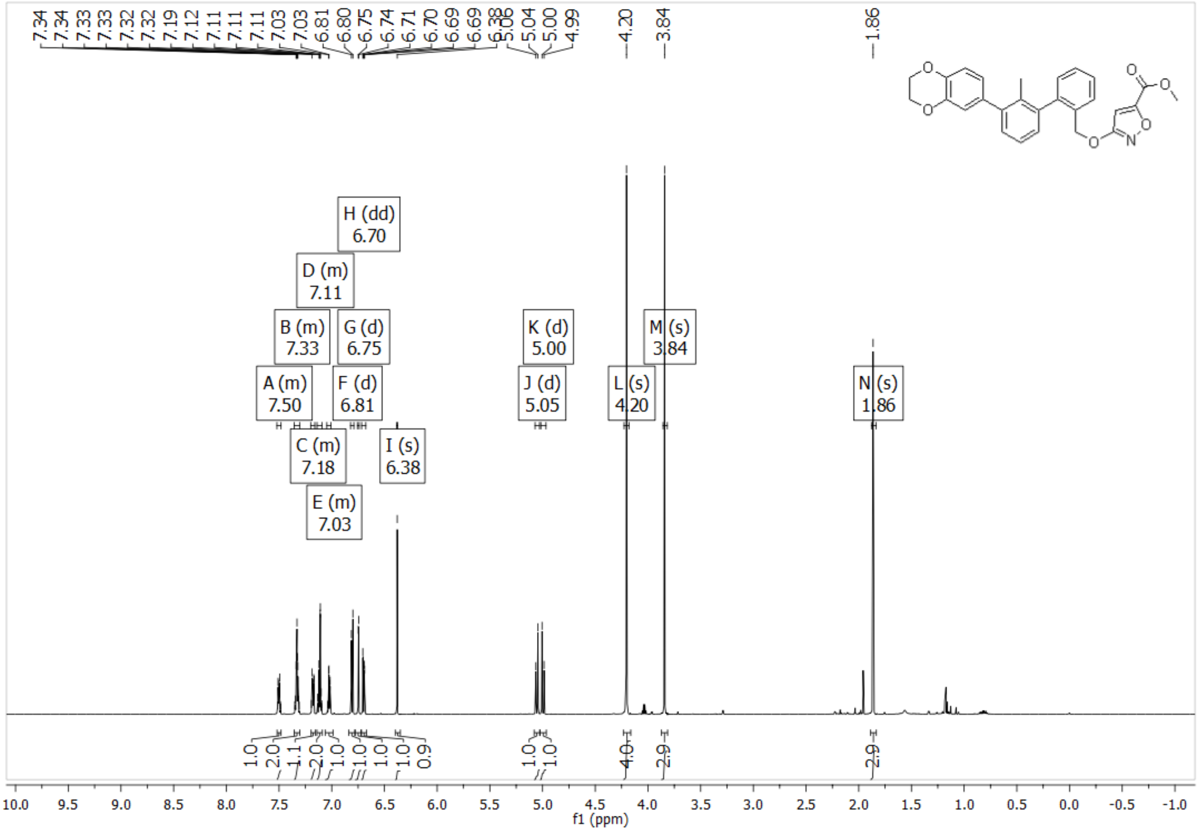

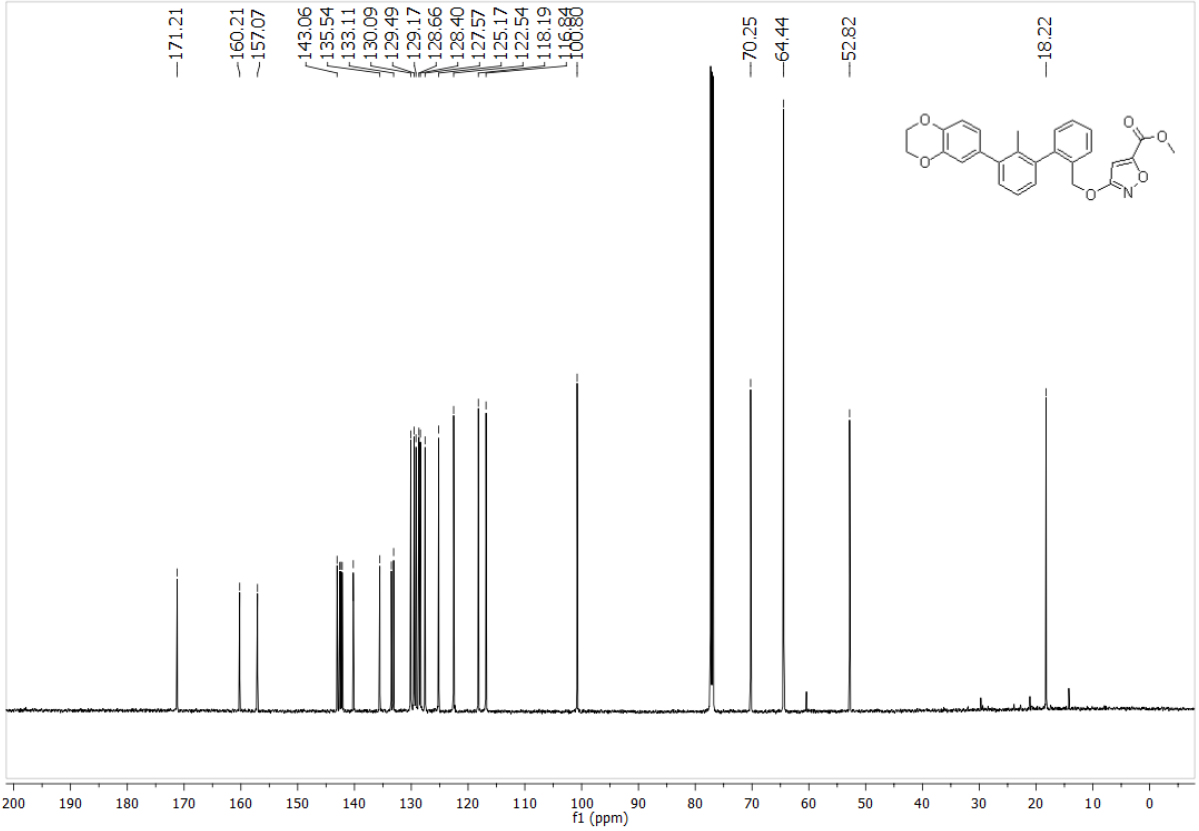

5r. 3-((3'-(benzo-1,4-dioxan-6-yl)-2'-methyl-[1,1'-biphenyl]-2-yl)methoxy)isoxazole-5-carboxylic acid

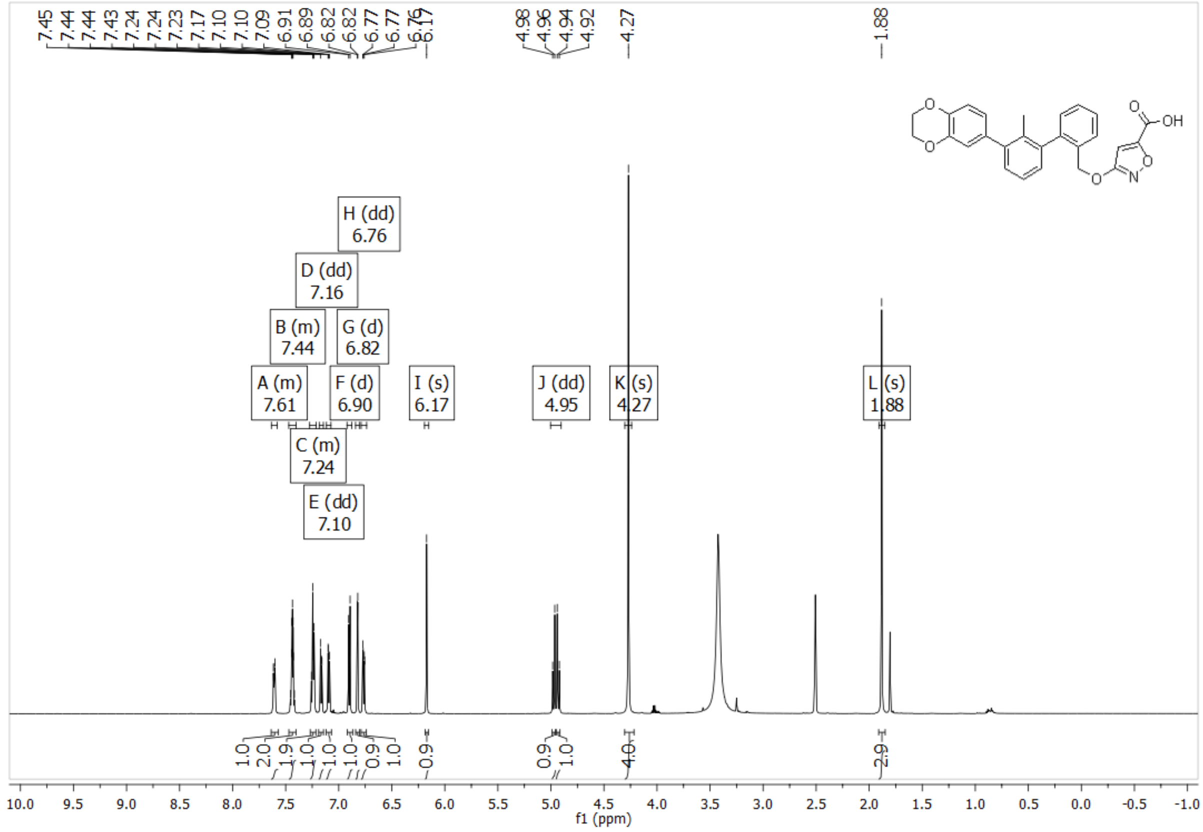

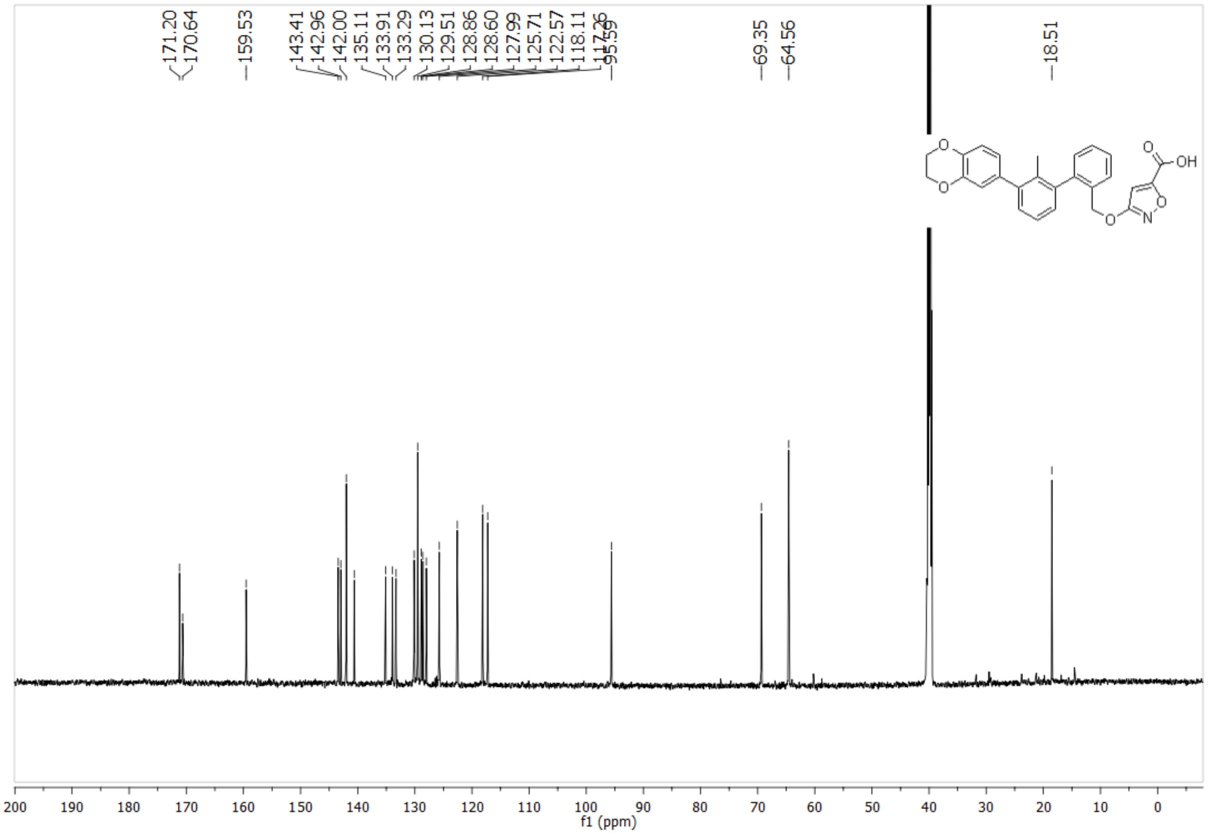

6a. 4-((3'-(benzo-1,4-dioxan-6-yl)-2'-methyl-[1,1'-biphenyl]-4-yl)methyl)morpholine

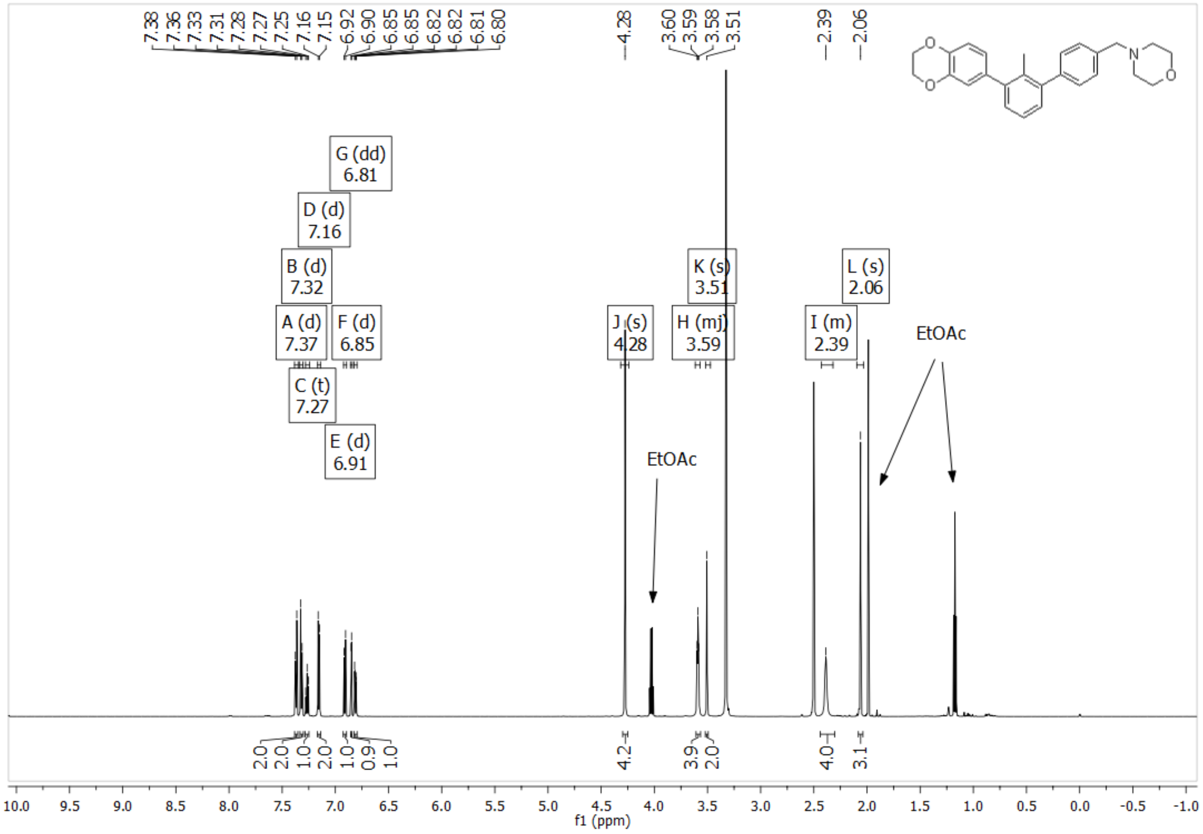

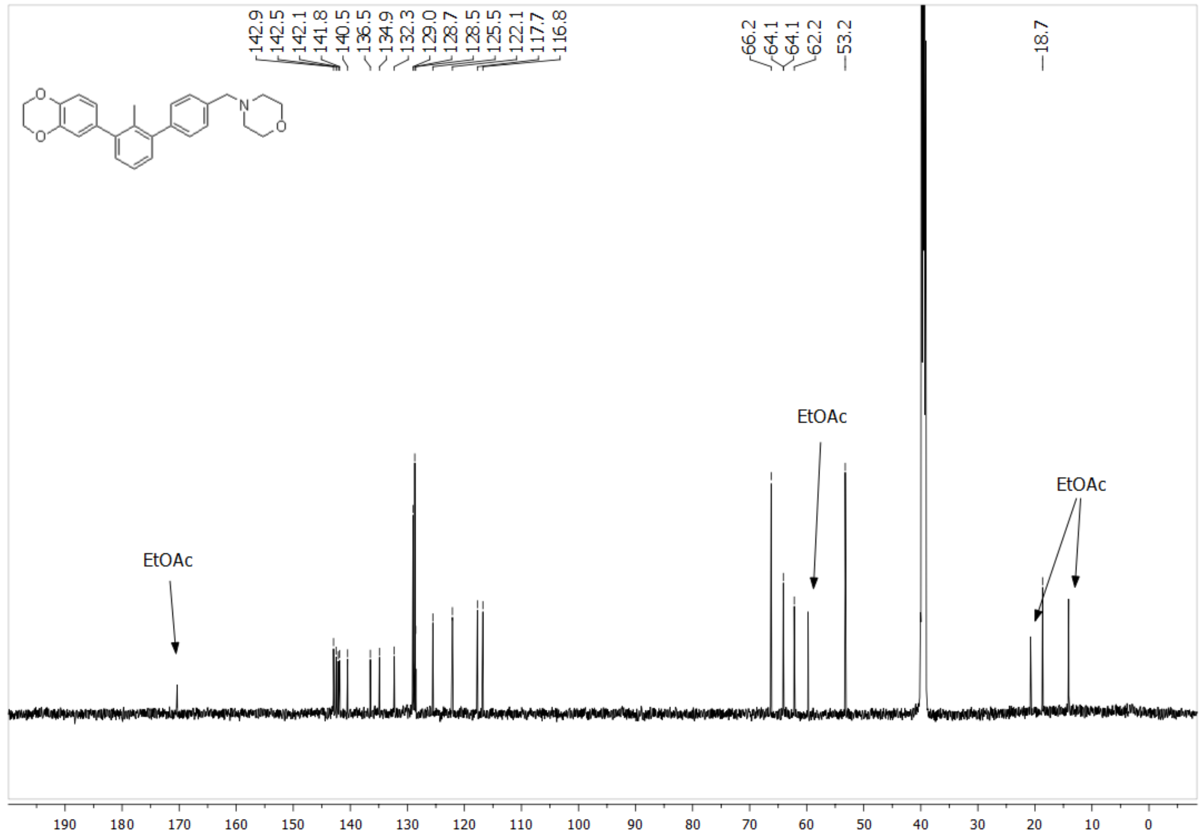

6b. 4-((3'-(benzo-1,4-dioxan-6-yl)-2'-methyl-[1,1'-biphenyl]-4-yl)methyl)piperazine

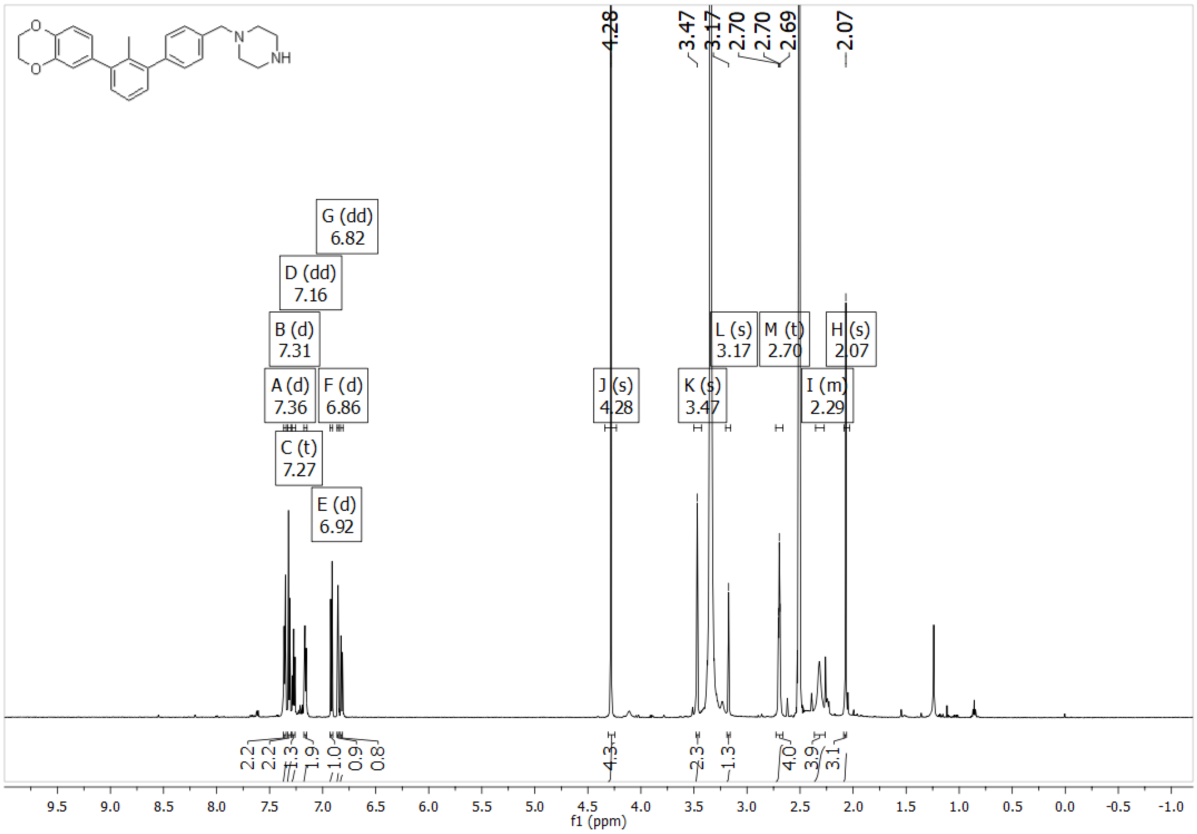

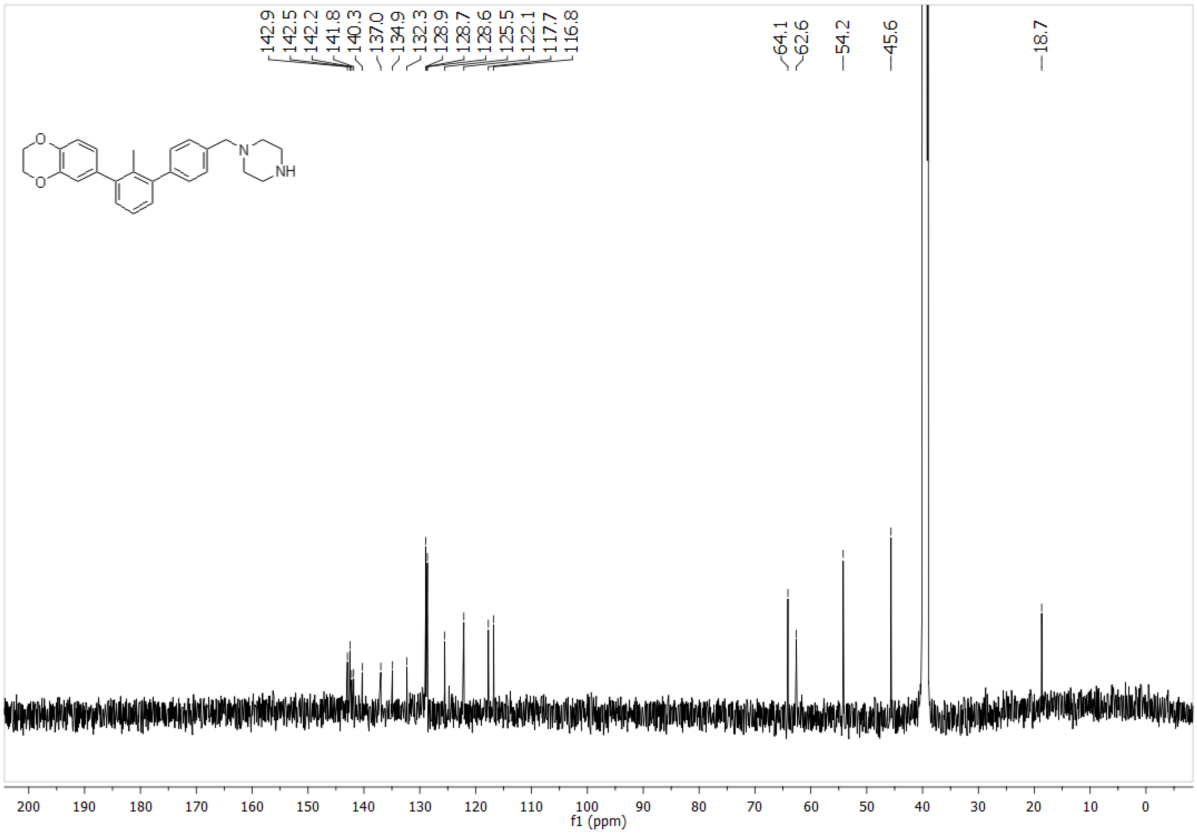

6c. 1-((3'-(benzo-1,4-dioxan-6-yl)-2'-methyl-[1,1'-biphenyl]-3-yl)methyl)piperazine

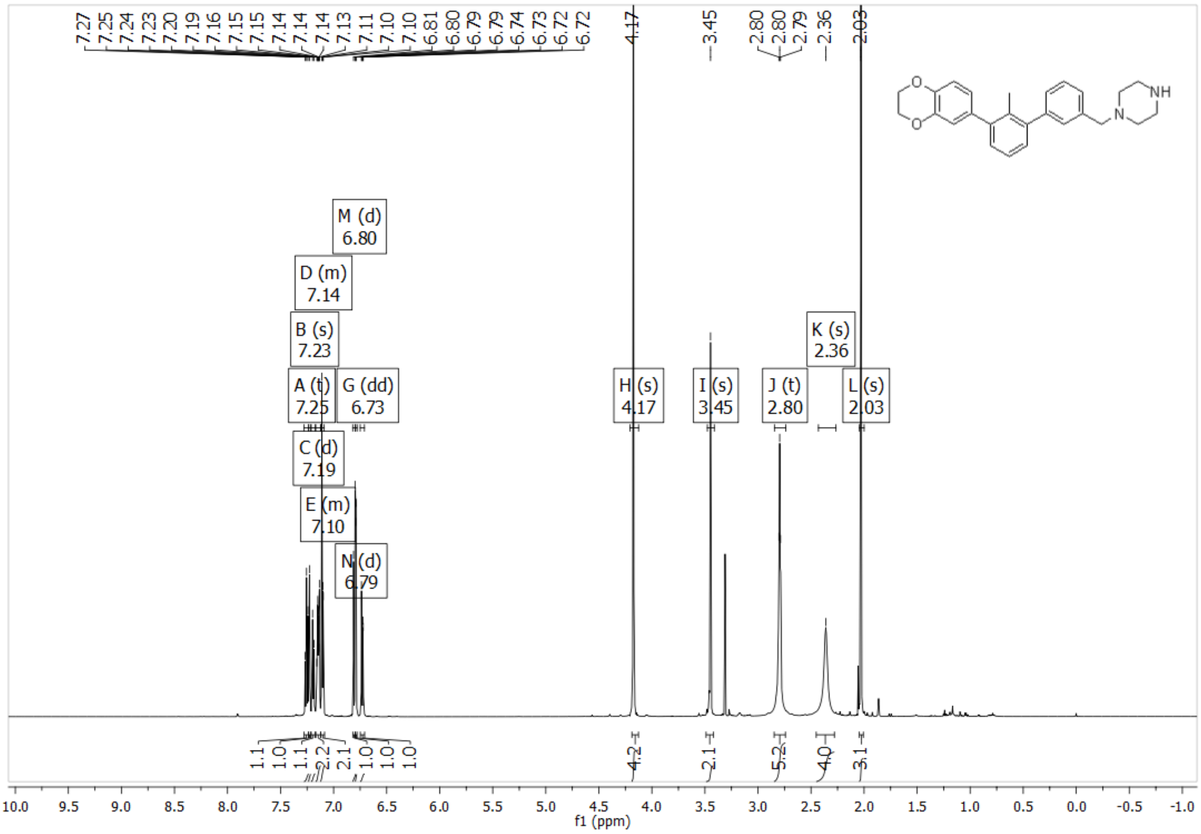

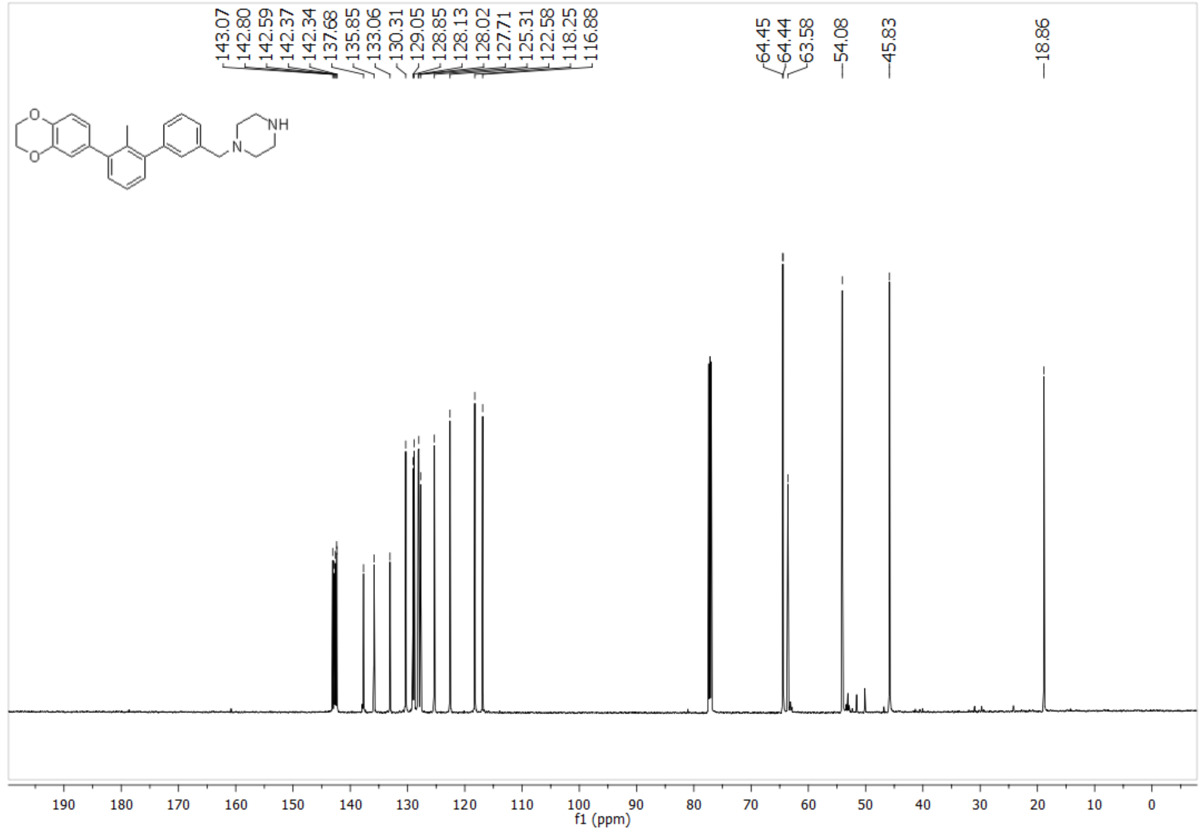

6d. 1-((3'-(benzo-1,4-dioxan-6-yl)-3-methoxy-2'-methyl-[1,1'-biphenyl]-4-yl)methyl)piperazine

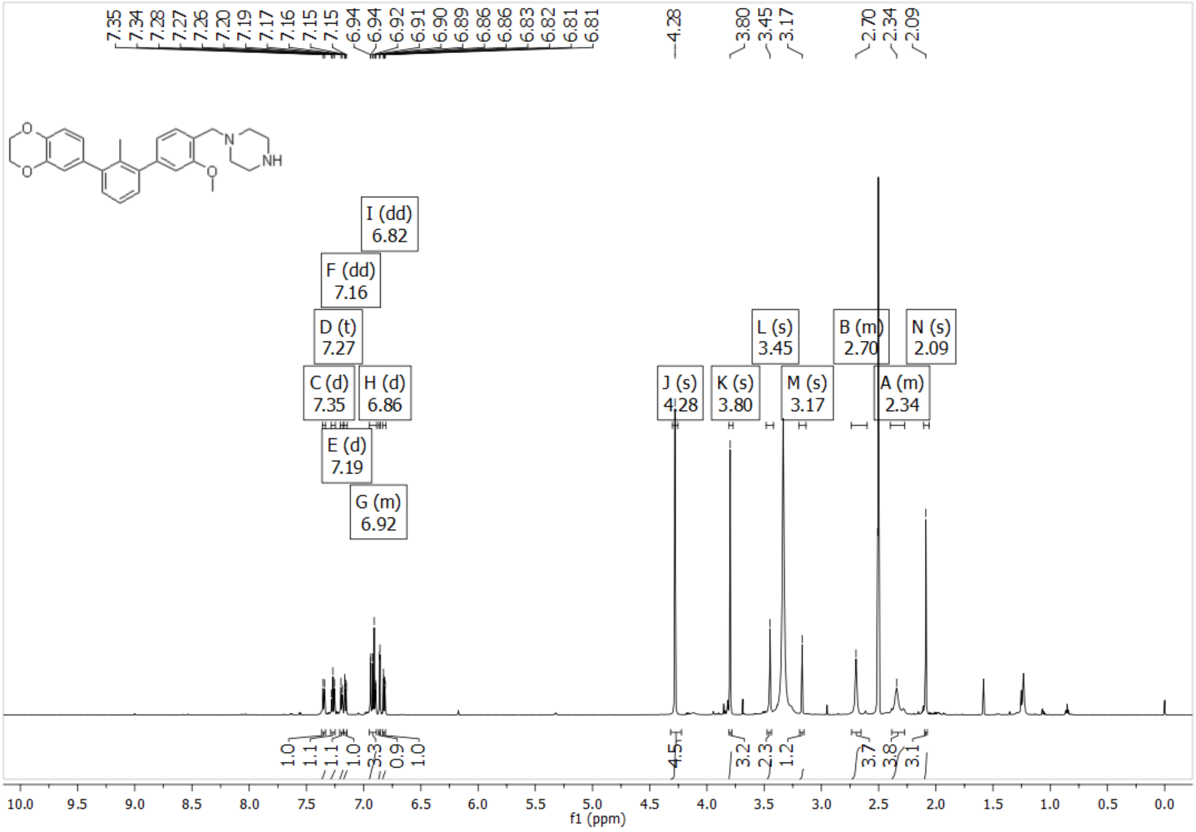

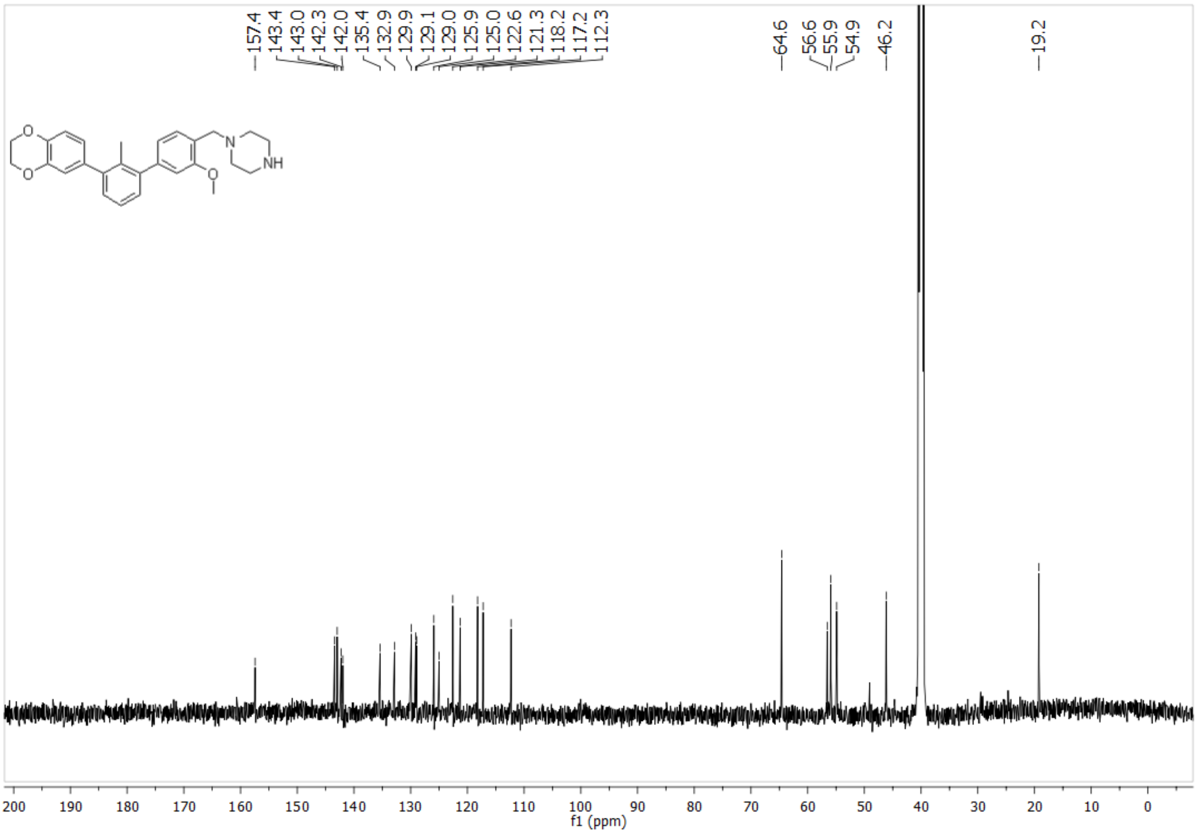

6e. 3-(((3'-(2,3-benzo-1,4-dioxan-6-yl)-2'-methyl-4-(piperazin-1-ylmethyl)-[1,1'-biphenyl]-3-yl)oxy)methyl)benzonitrile

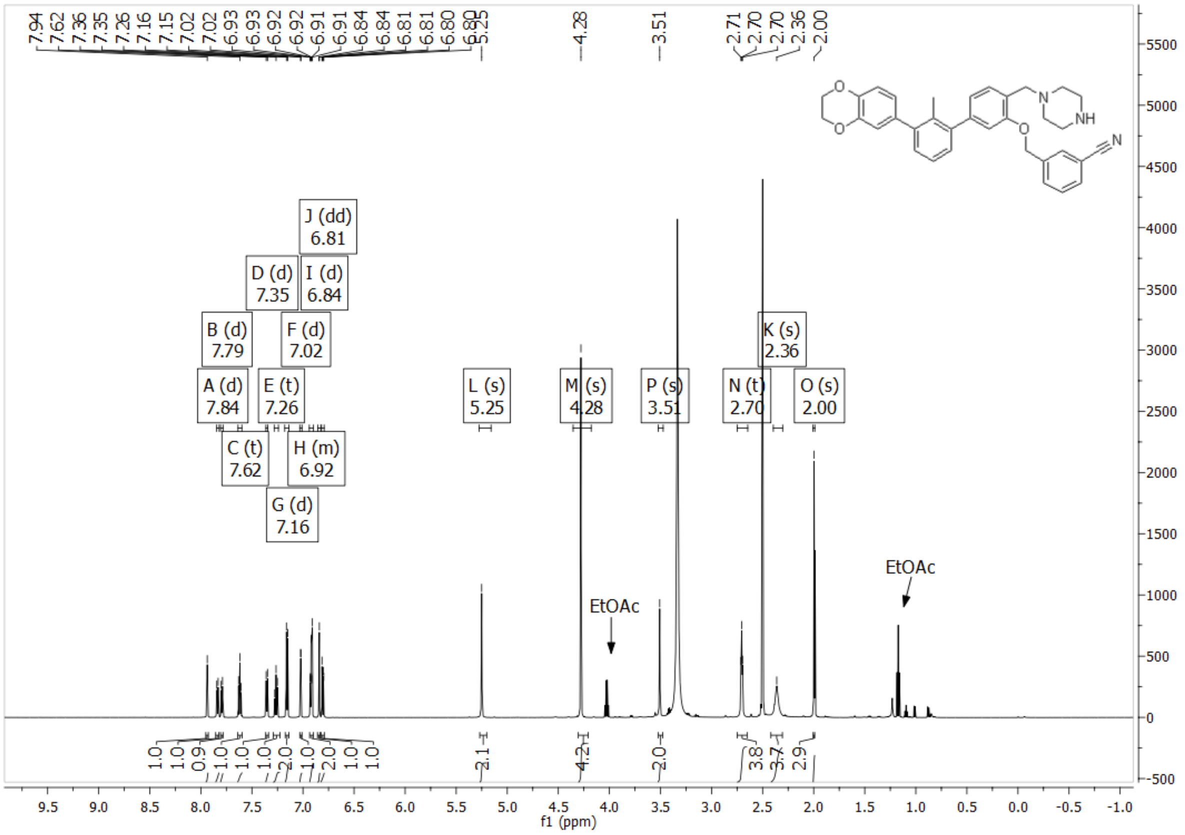

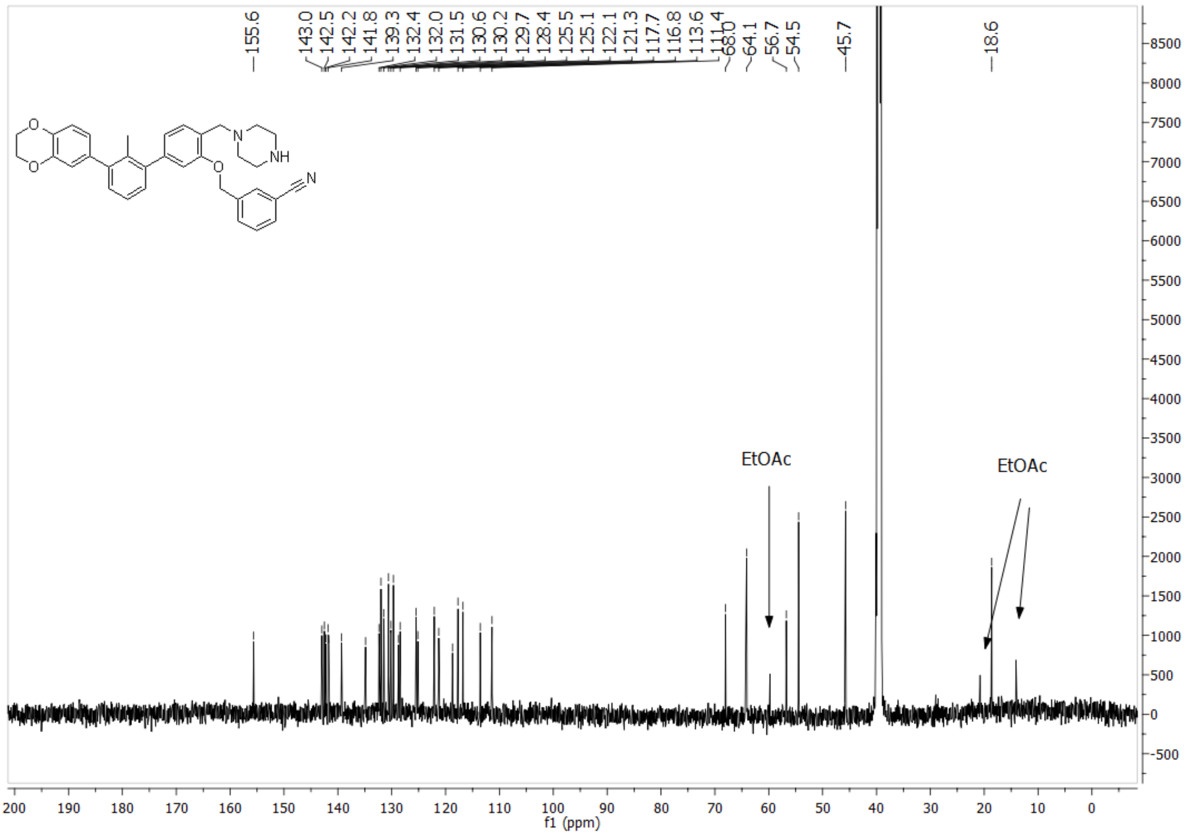

7a. N-(2-(((2'-chloro-3'-(benzo-1,4-dioxan-6-yl)-3-methoxy-[1,1'-biphenyl]-4-yl)methyl)amino)ethyl)acetamide

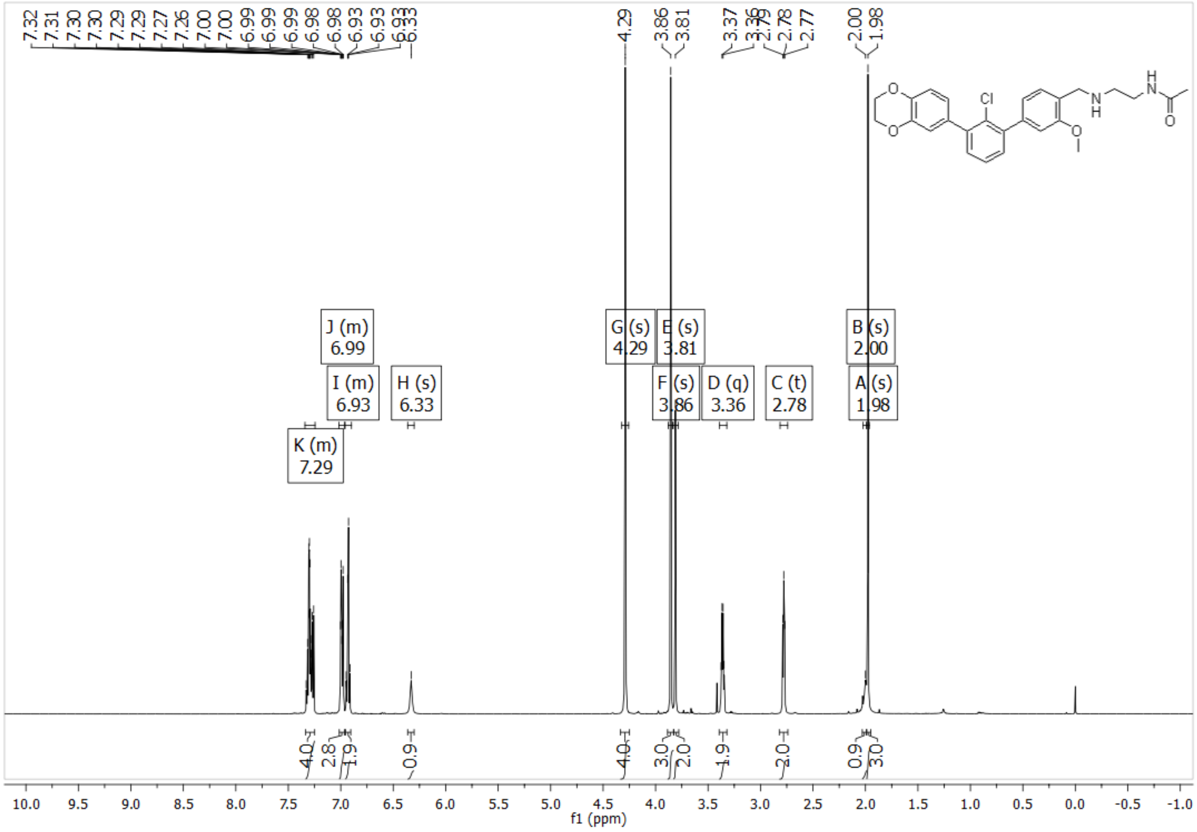

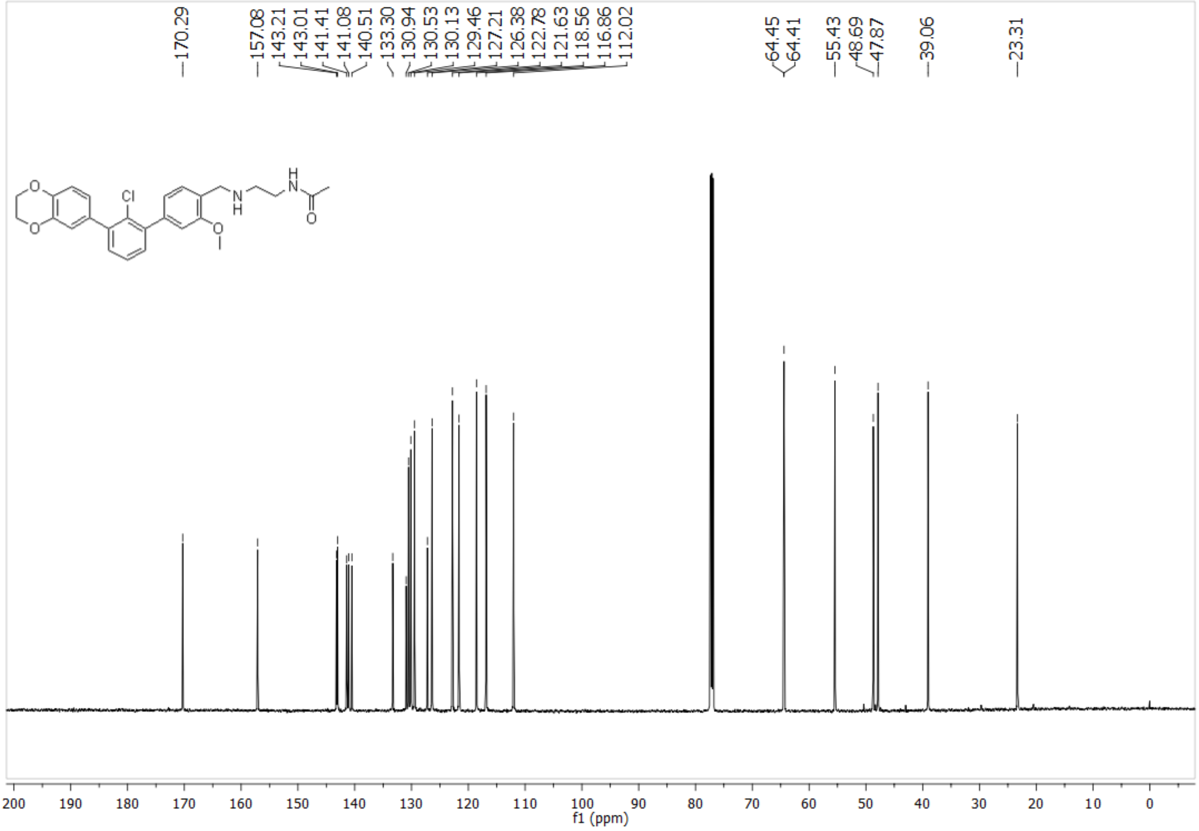

7b. N-(2-((((benzo-1,4-dioxan-6-yl)-2'-chloro-3'-3-ethoxy-[1,1'-biphenyl]-4-yl)methyl)amino)ethyl)acetamide

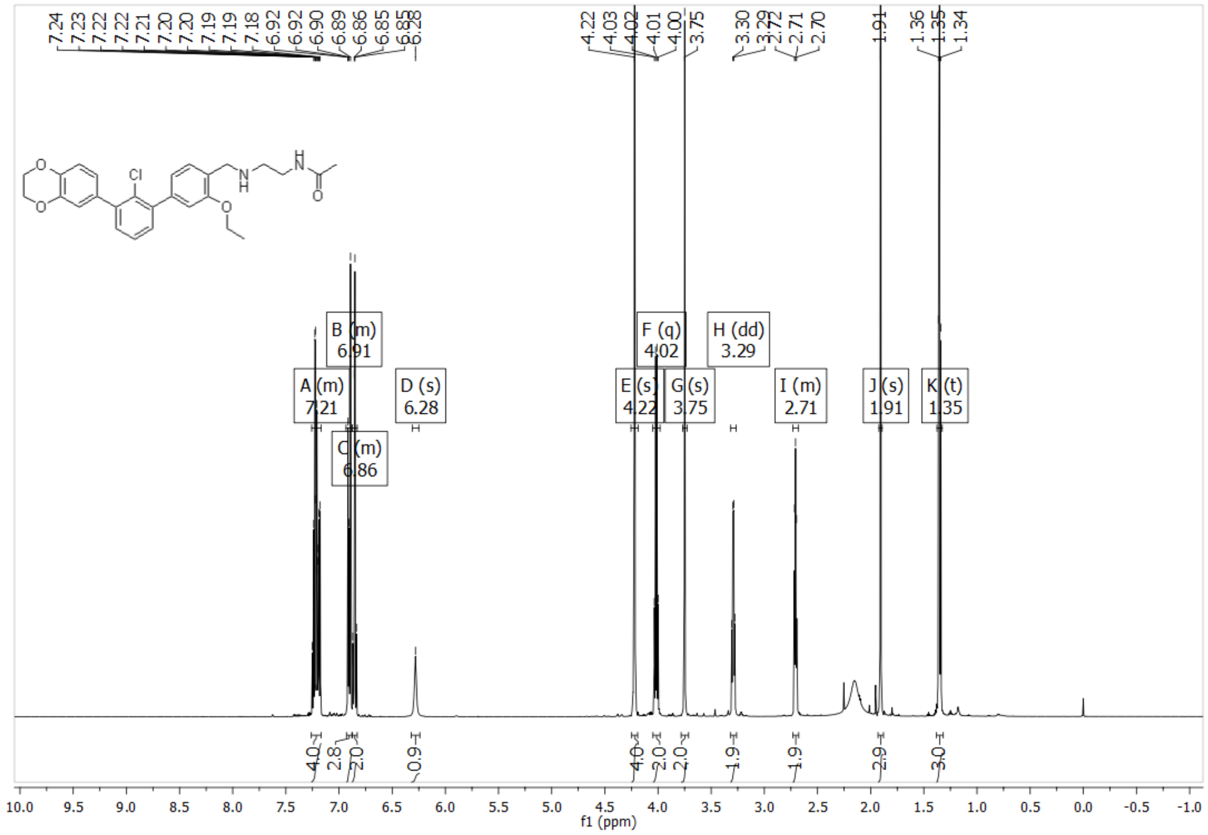

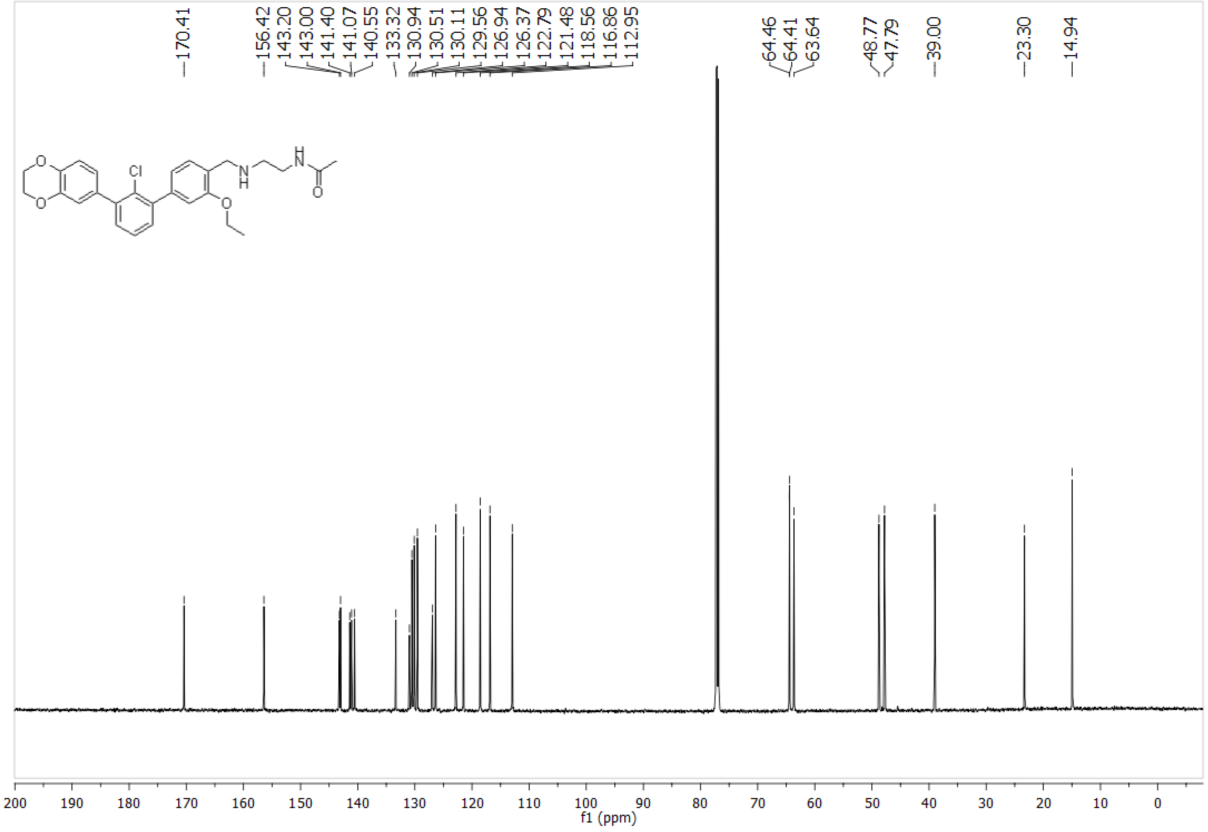

7c. N-(2-(((2'-chloro-3-((3'-cyano-[1,1'-biphenyl]-3-yl)isopropoxy)-3'-(benzo-1,4-dioxan-6-yl)-[1,1'-biphenyl]-4-yl)methyl)amino)ethyl)acetamide

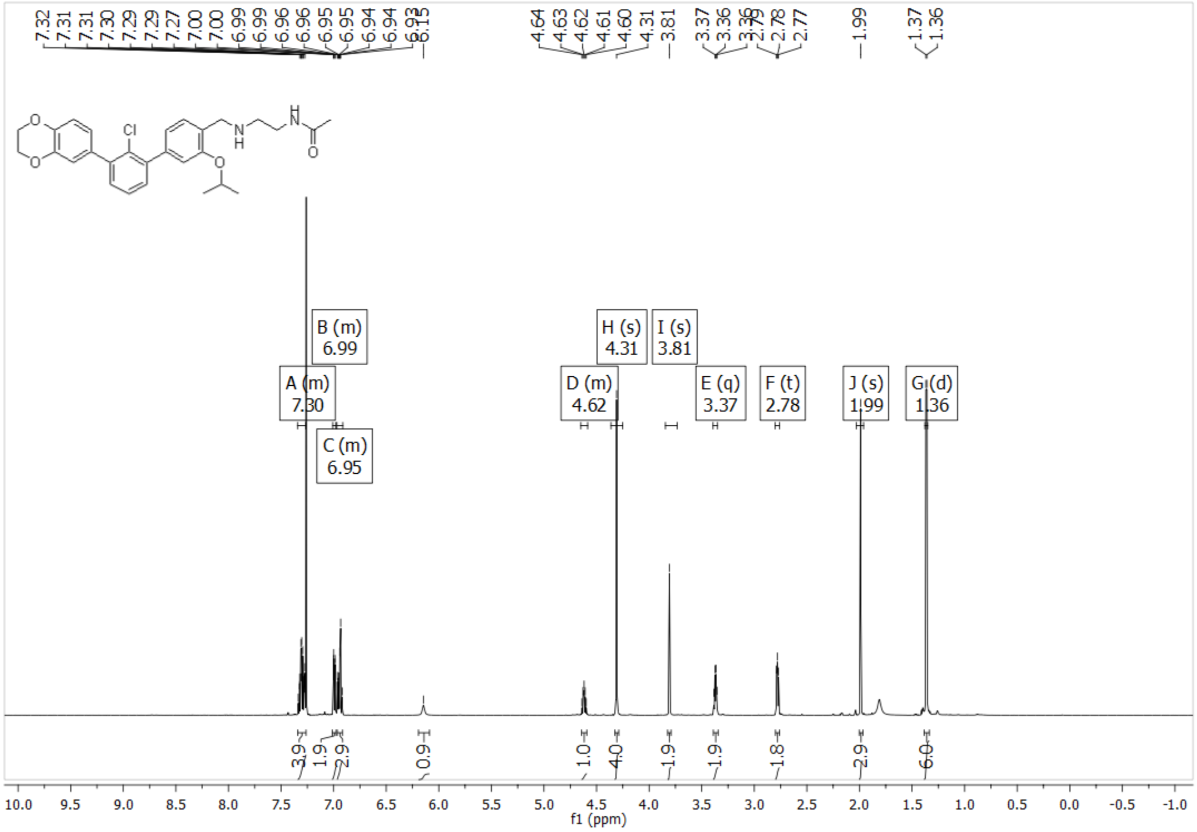

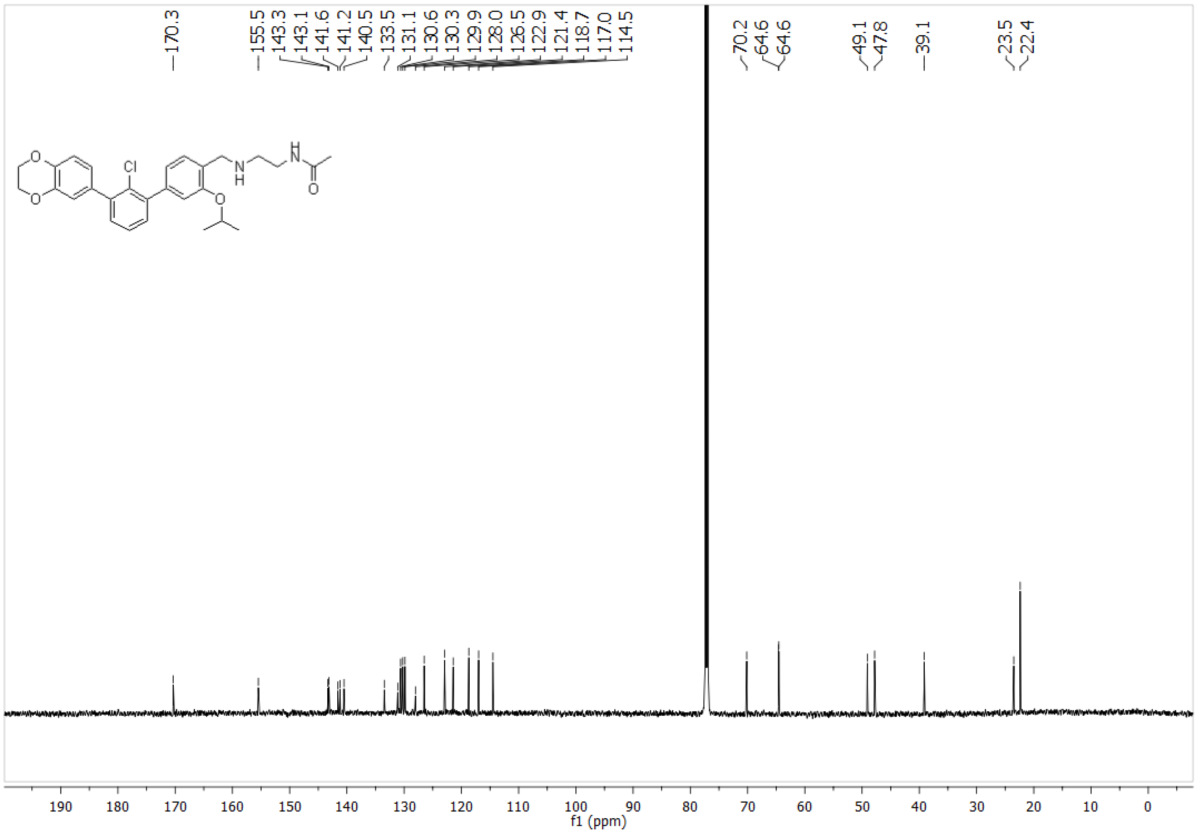

7d. N-(2-(((2'-chloro-3-((3'-cyano-[1,1'-biphenyl]-3-yl)isopentyloxy)-3'-(benzo-1,4-dioxan-6-yl)-[1,1'-biphenyl]-4-yl)methyl)amino)ethyl)acetamide

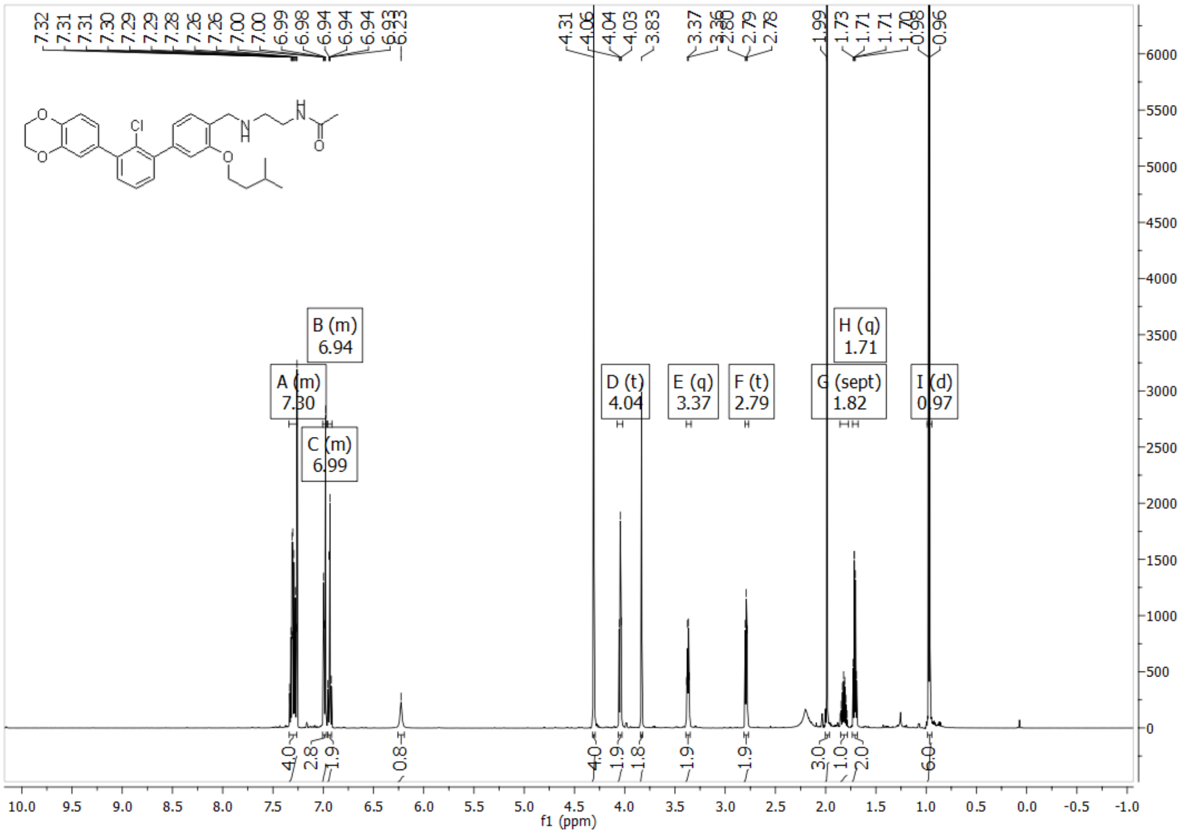

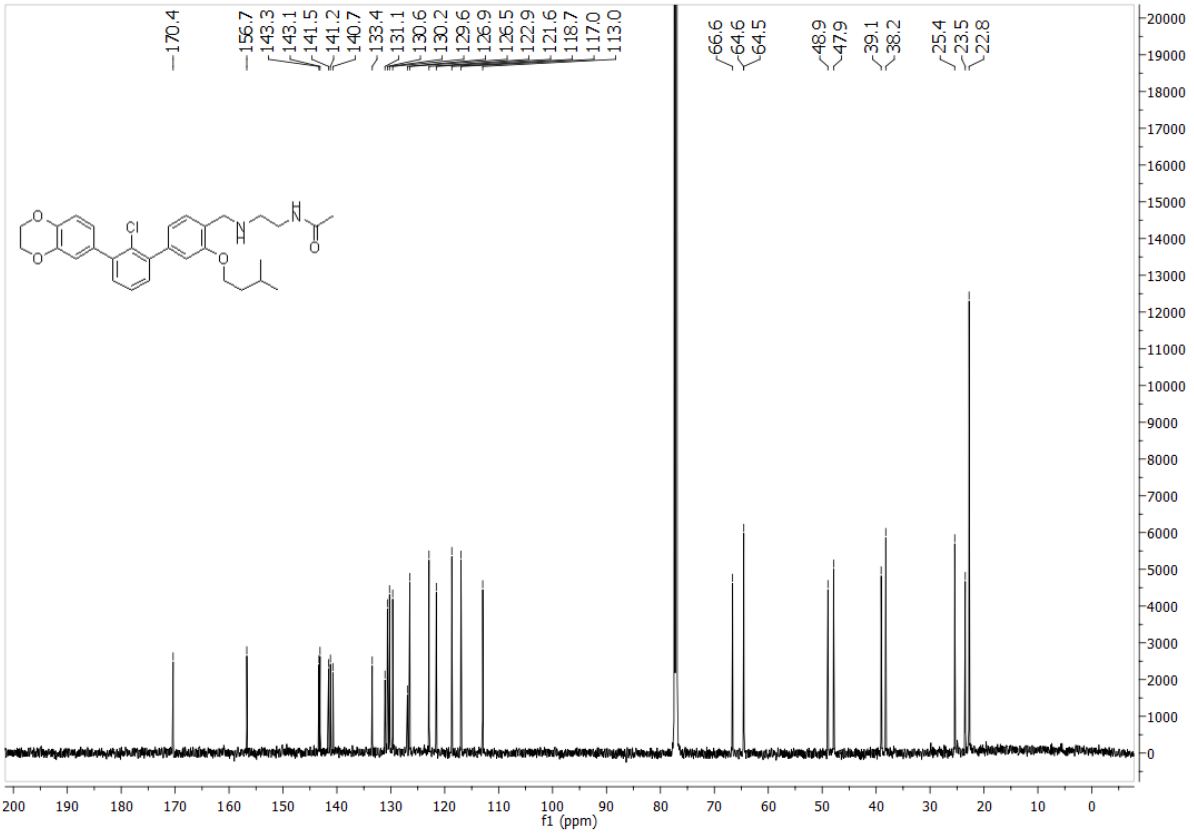

7e. N-(2-(((3'-(benzo-1,4-dioxan-6-yl)-2'-chloro-3-(cyanomethoxy)-[1,1'-biphenyl]-4-yl)methyl)amino)ethyl)acetamide

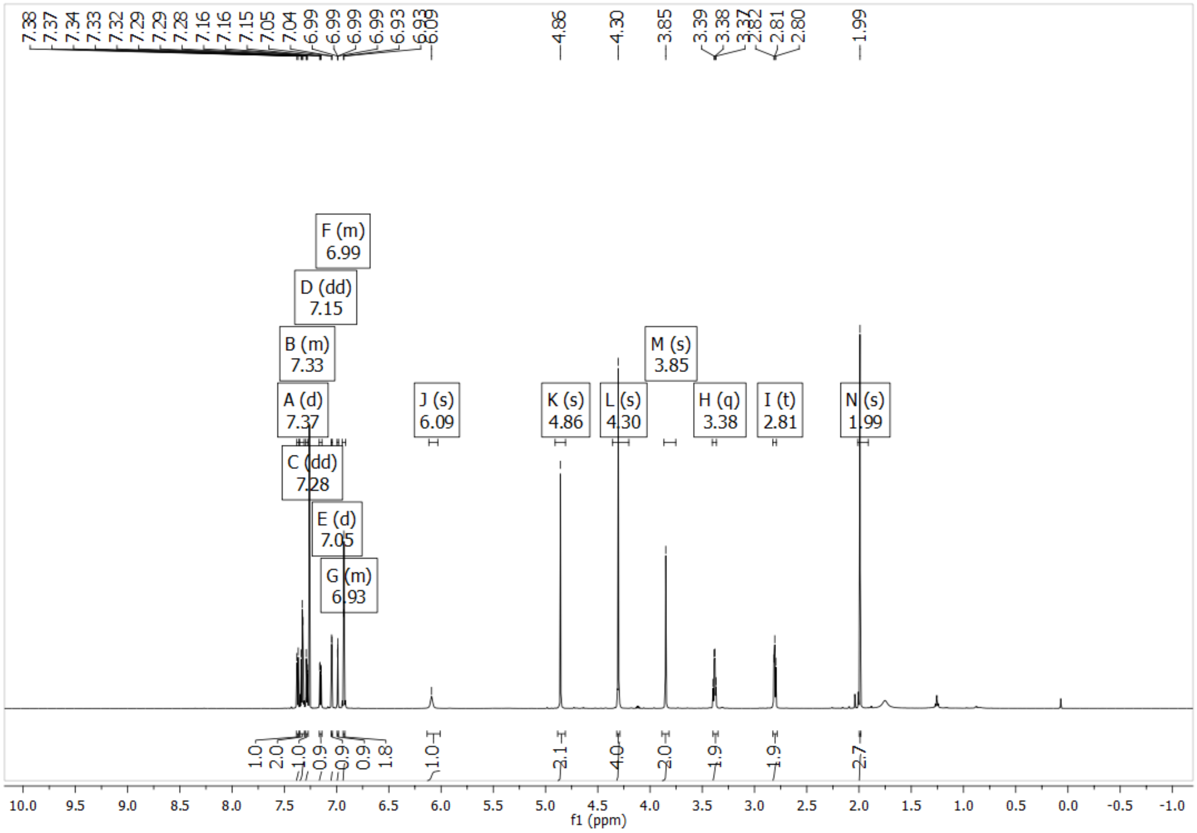

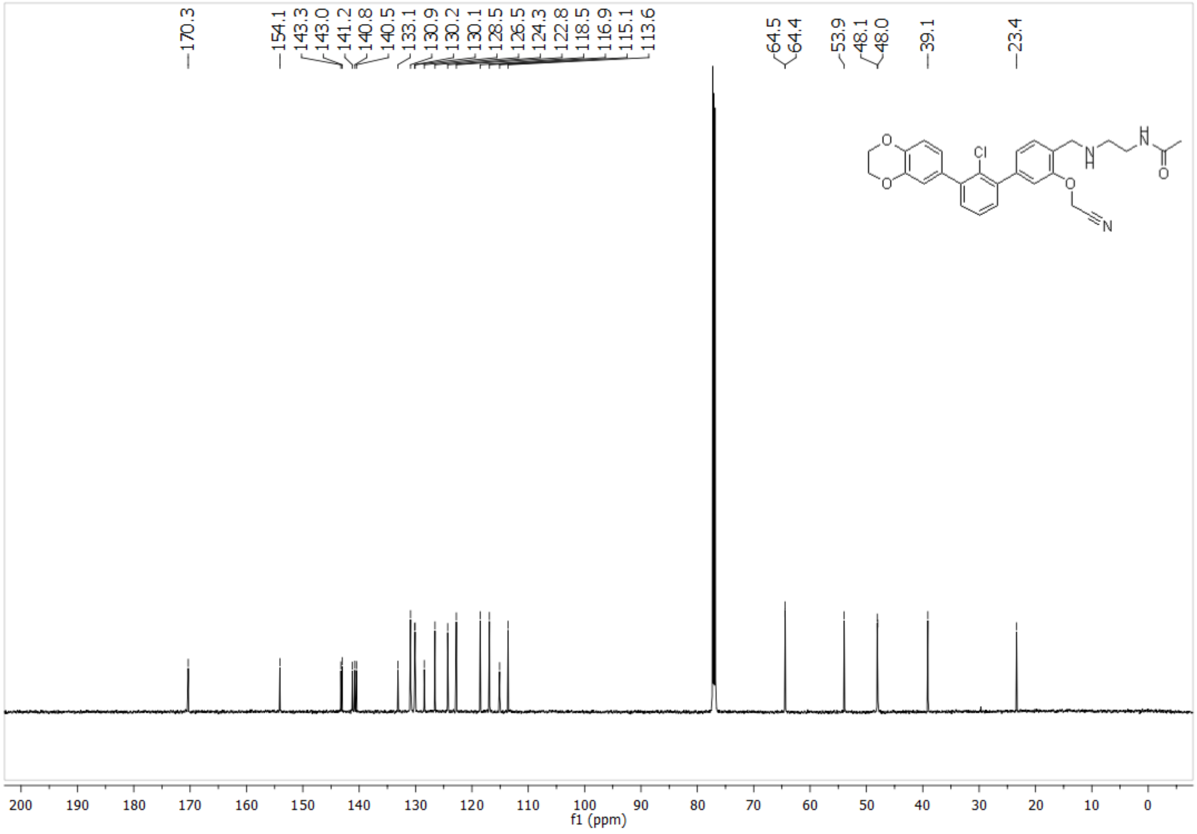

7f. N-(2-(((3'-(benzo-1,4-dioxan-6-yl)-2'-chloro-3-(cyclobutylmethoxy)-[1,1'-biphenyl]-4-yl)methyl)amino)ethyl)acetamide

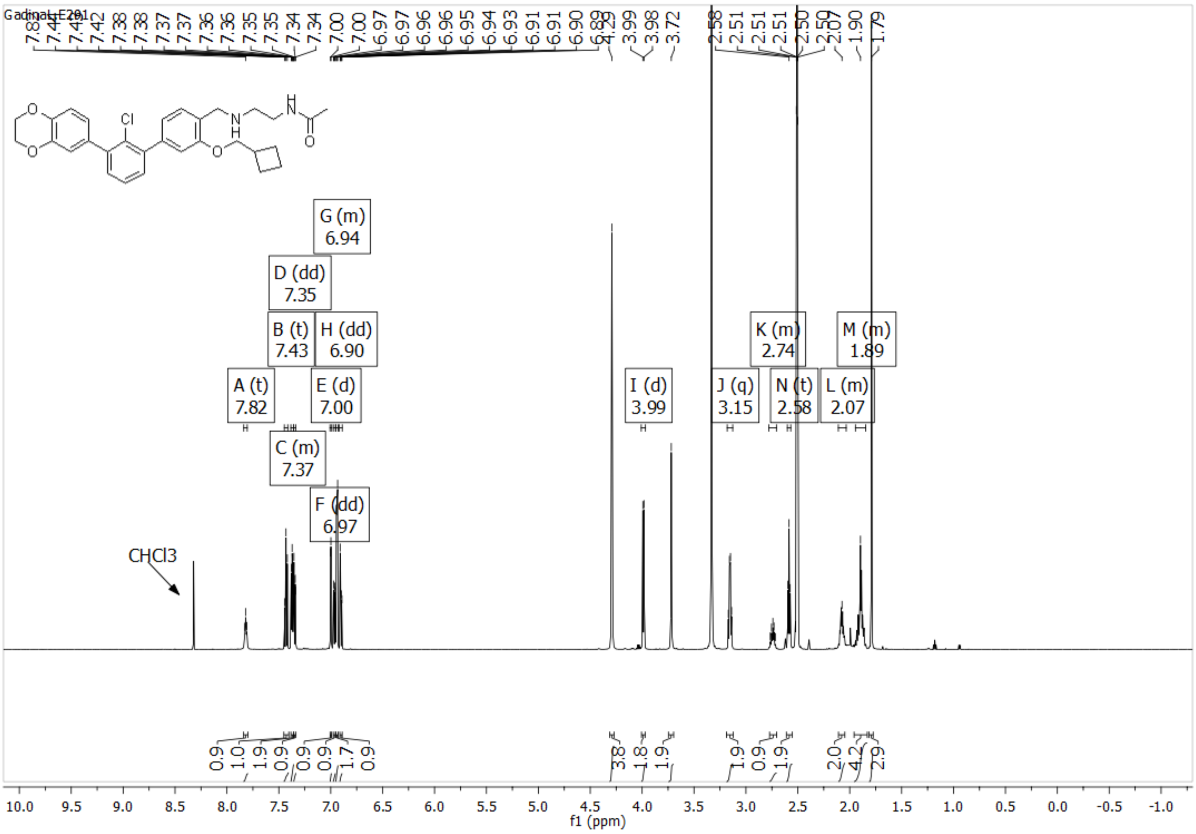

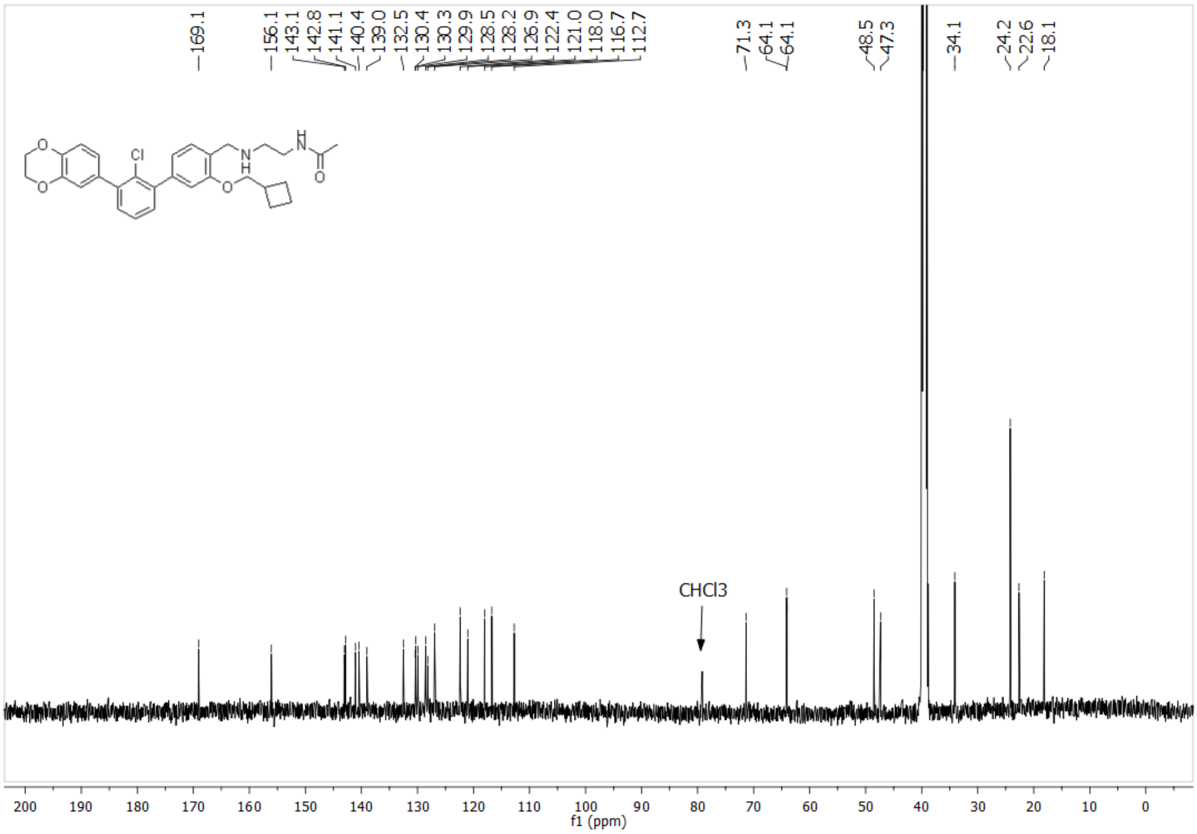

7g. N-(2-(((3'-(benzo-1,4-dioxan-6-yl)-2'-chloro-3-(cyclopentylmethoxy)-[1,1'-biphenyl]-4-yl)methyl)amino)ethyl)acetamide

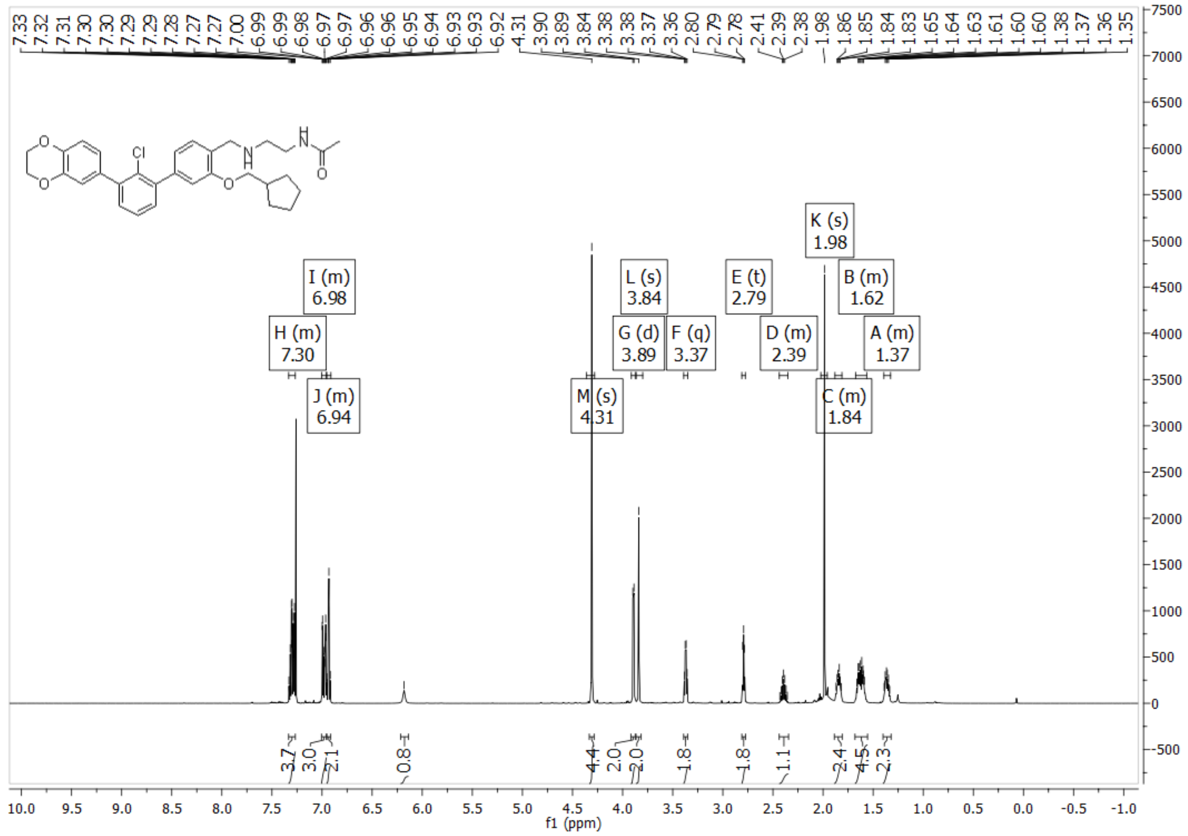

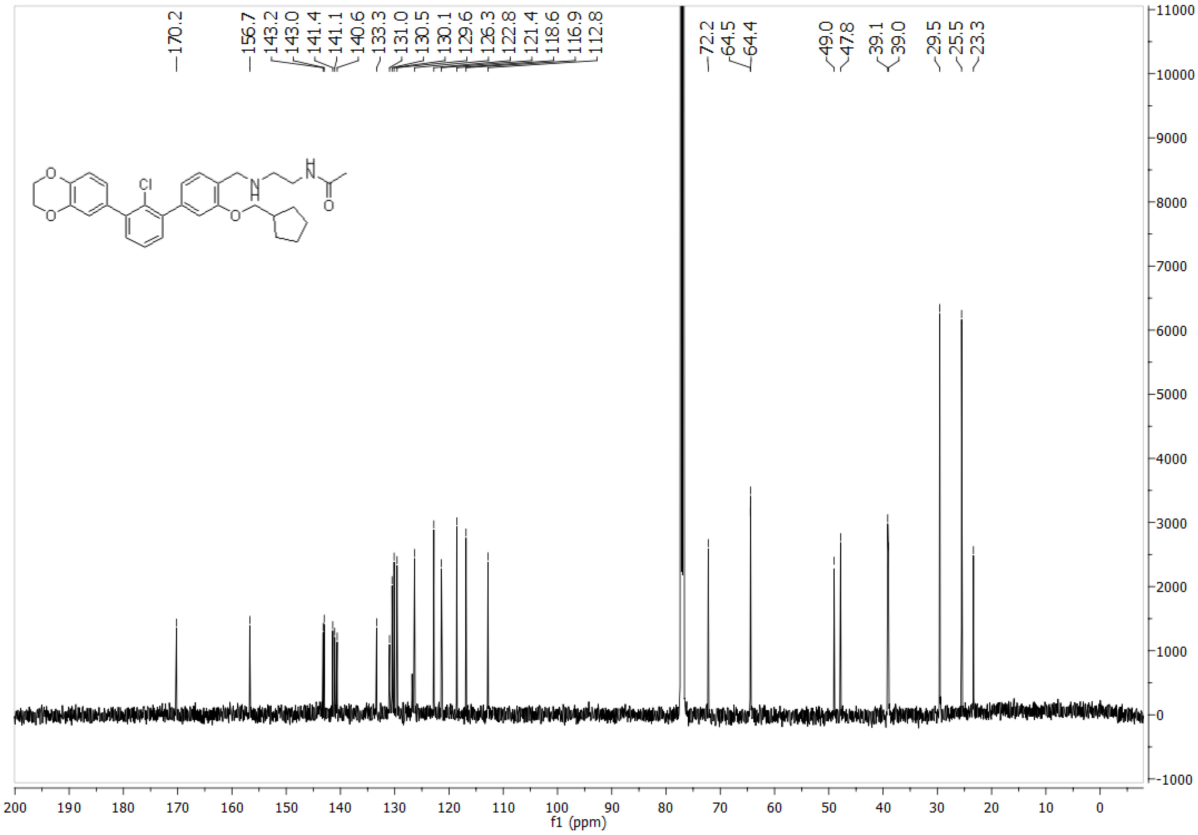

7h. N-(2-(((3'-(benzo-1,4-dioxan-6-yl)-2'-chloro-3-((3'-cyano-[1,1'-biphenyl]-3-yl)methoxy)-[1,1'-biphenyl]-4-yl)methyl)amino)ethyl)acetamide

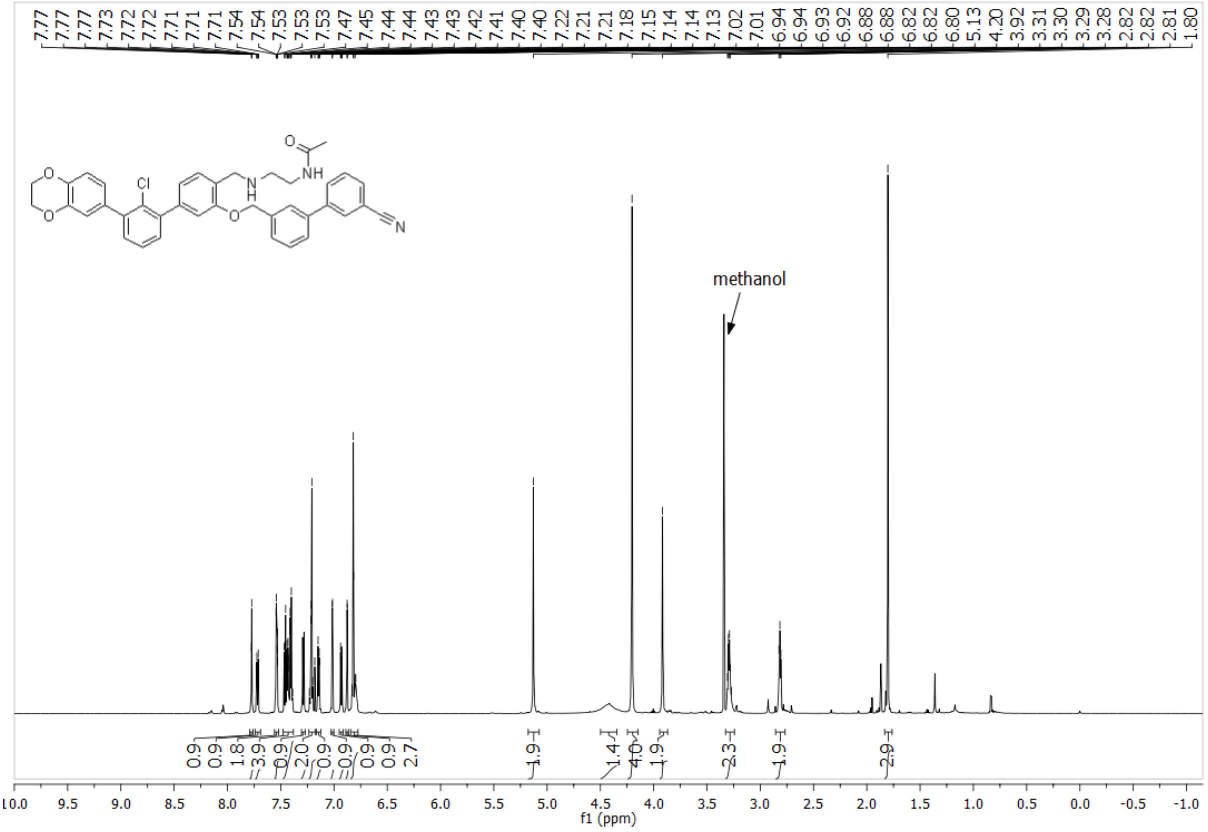

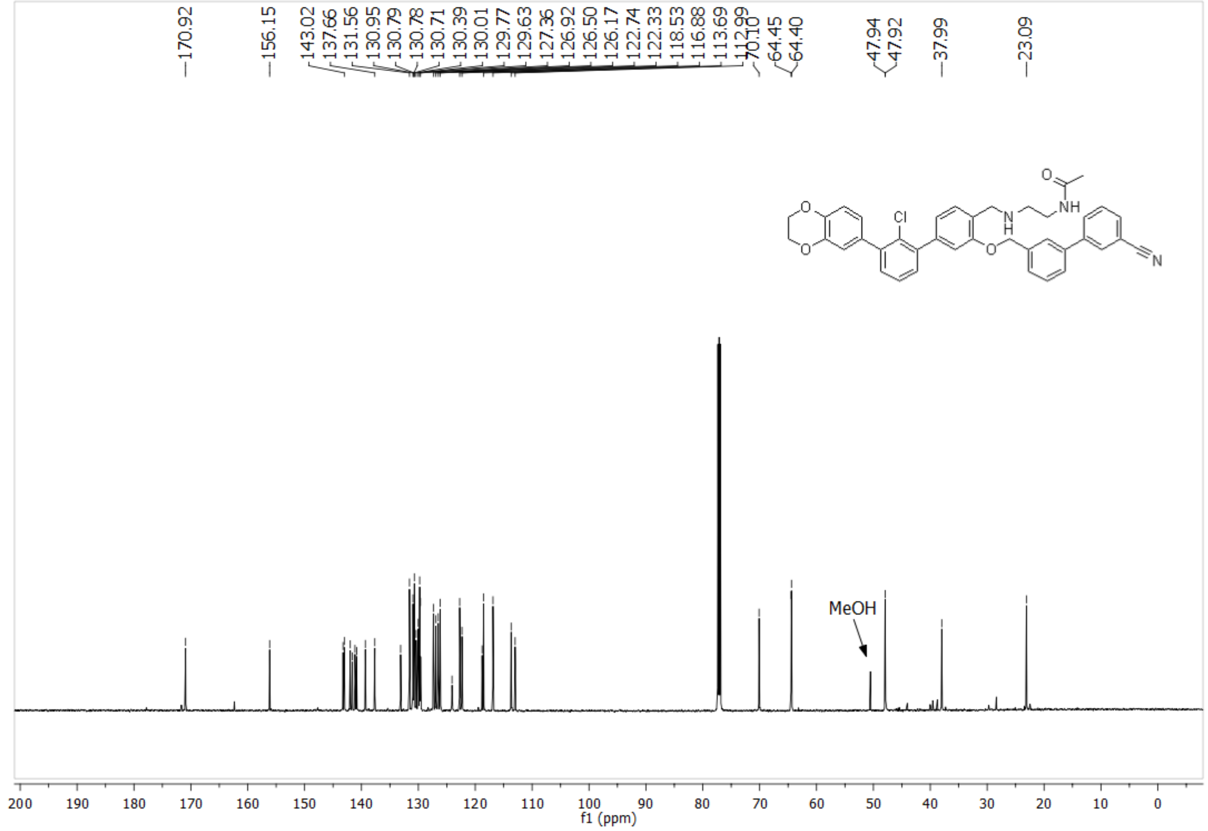

8a. 1-((2'-chloro-3'-(benzo-1,4-dioxan-6-yl)-3-methoxy-[1,1'-biphenyl]-4-yl)methyl)piperazine

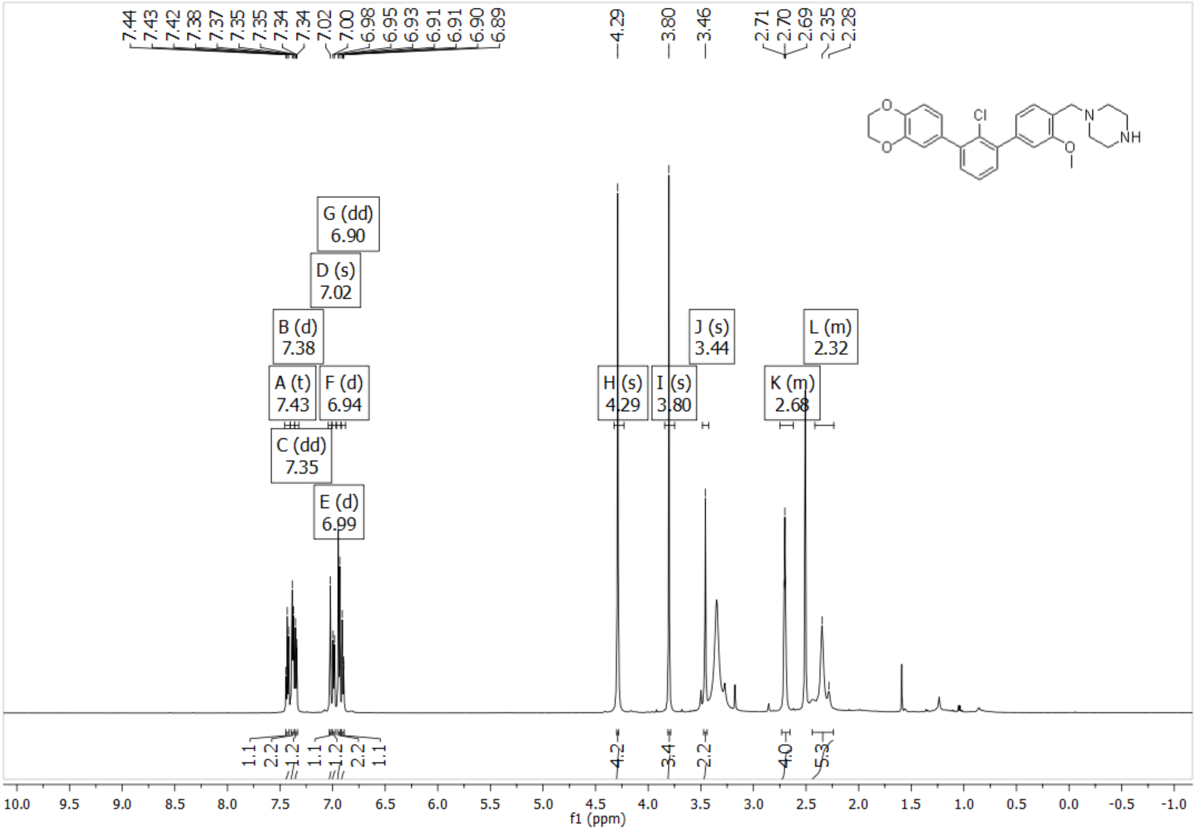

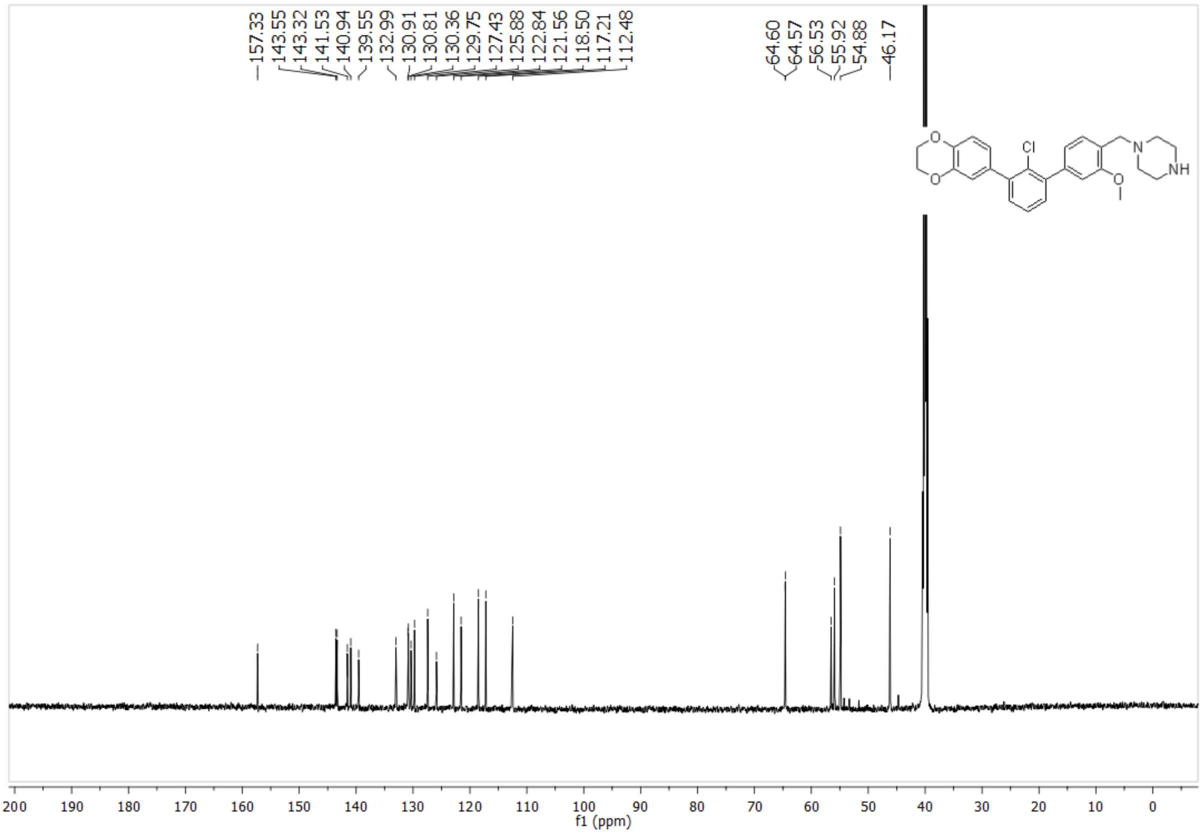

8b. 4-((2'-chloro-3'-(benzo-1,4-dioxan-6-yl)-3-methoxy-[1,1'-biphenyl]-4-yl)methyl)piperazine-1-carboxamide

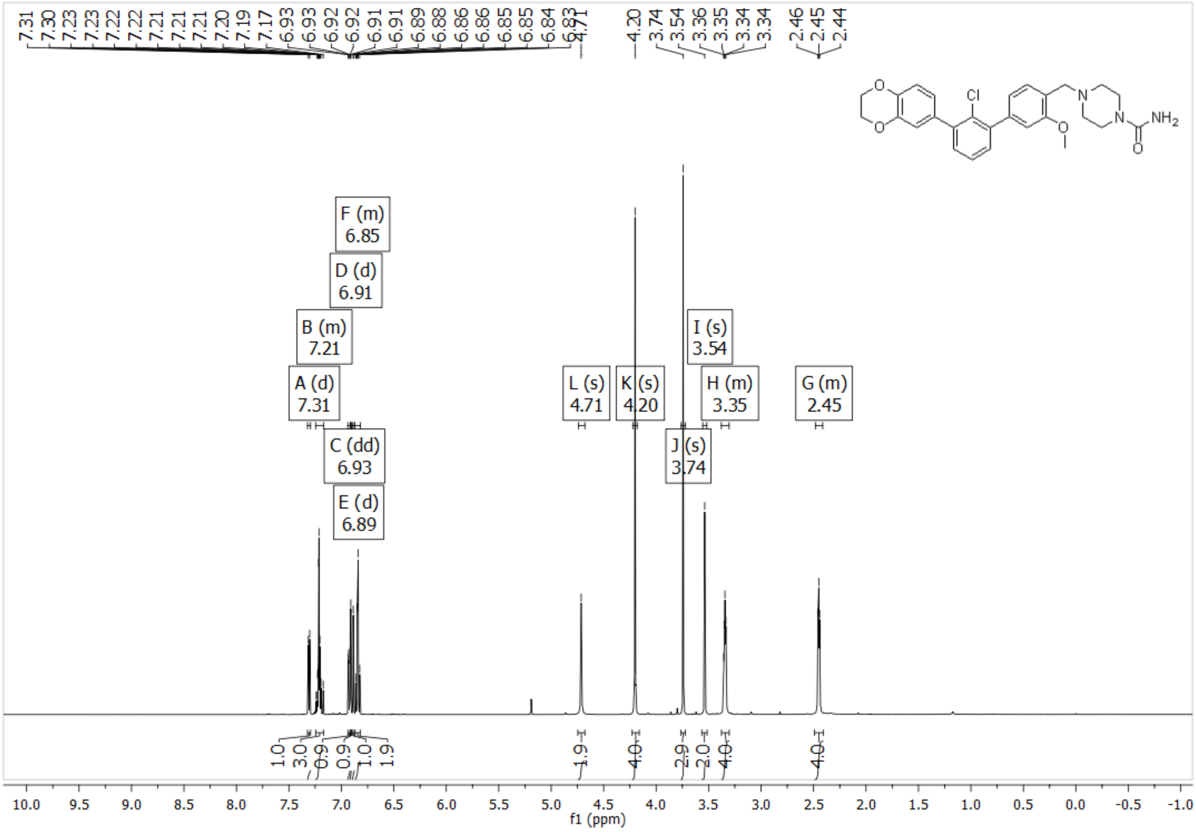

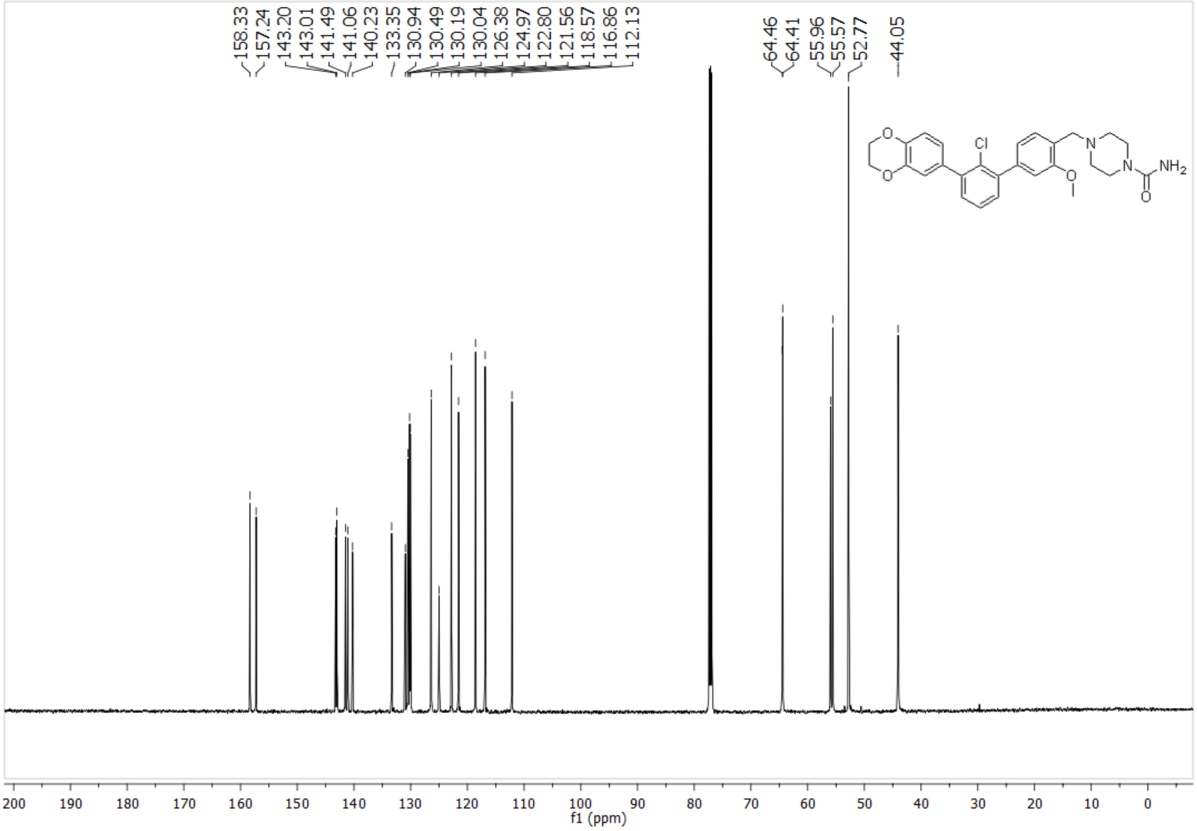

8c. 1-(4-((2'-Chloro-3'-(benzo-1,4-dioxan-6-yl)-3-methoxy-[1,1'-biphenyl]-4-yl)methyl)piperazin-1-yl)ethan-1-one

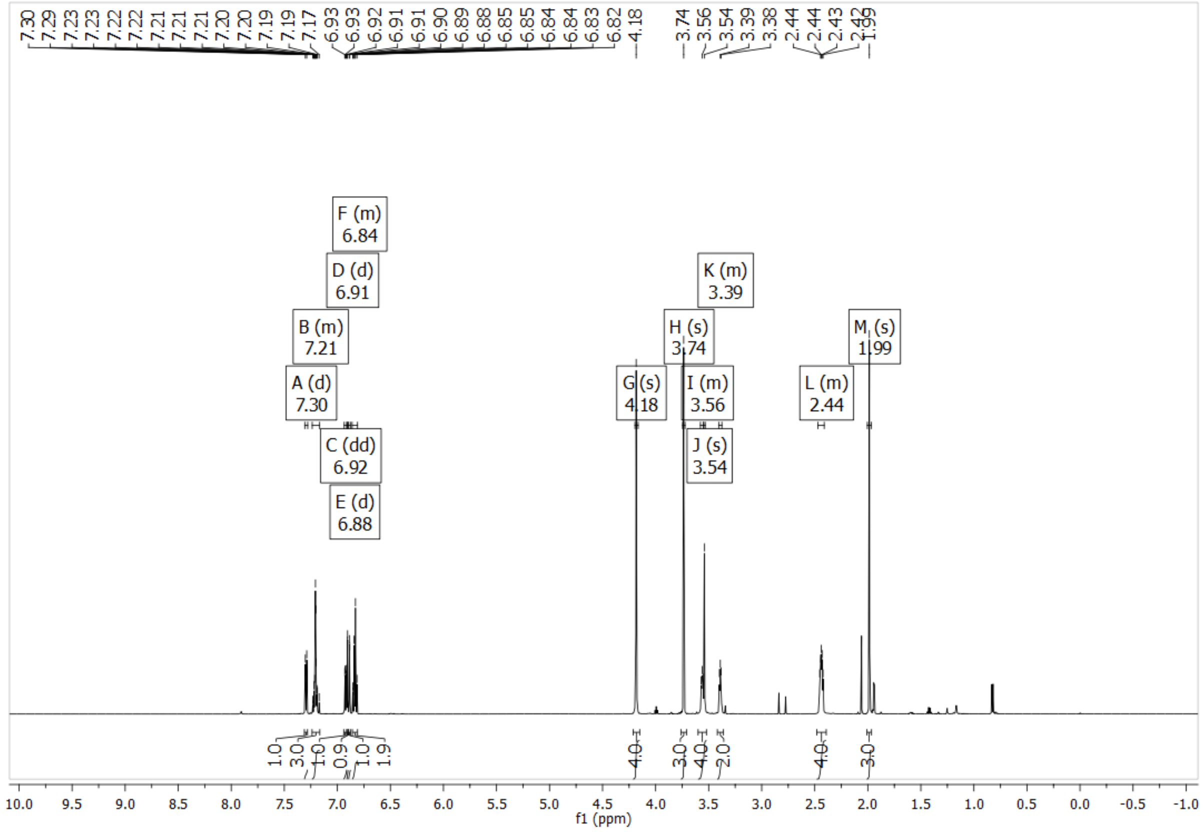

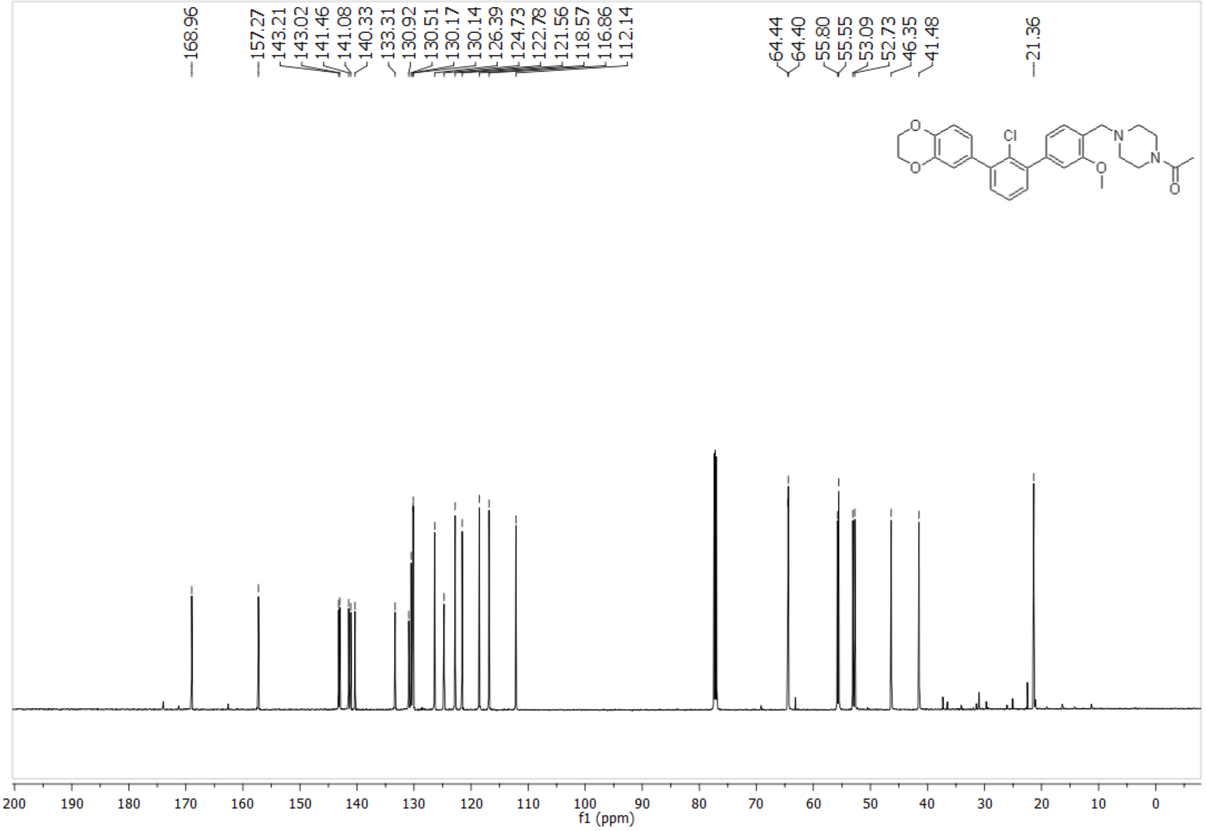

8d. 2-amino-1-(4-((2'-chloro-3'-(benzo-1,4-dioxan-6-yl)-3-methoxy-[1,1'-biphenyl]-4-yl)methyl)piperazin-1-yl)ethan-1-one

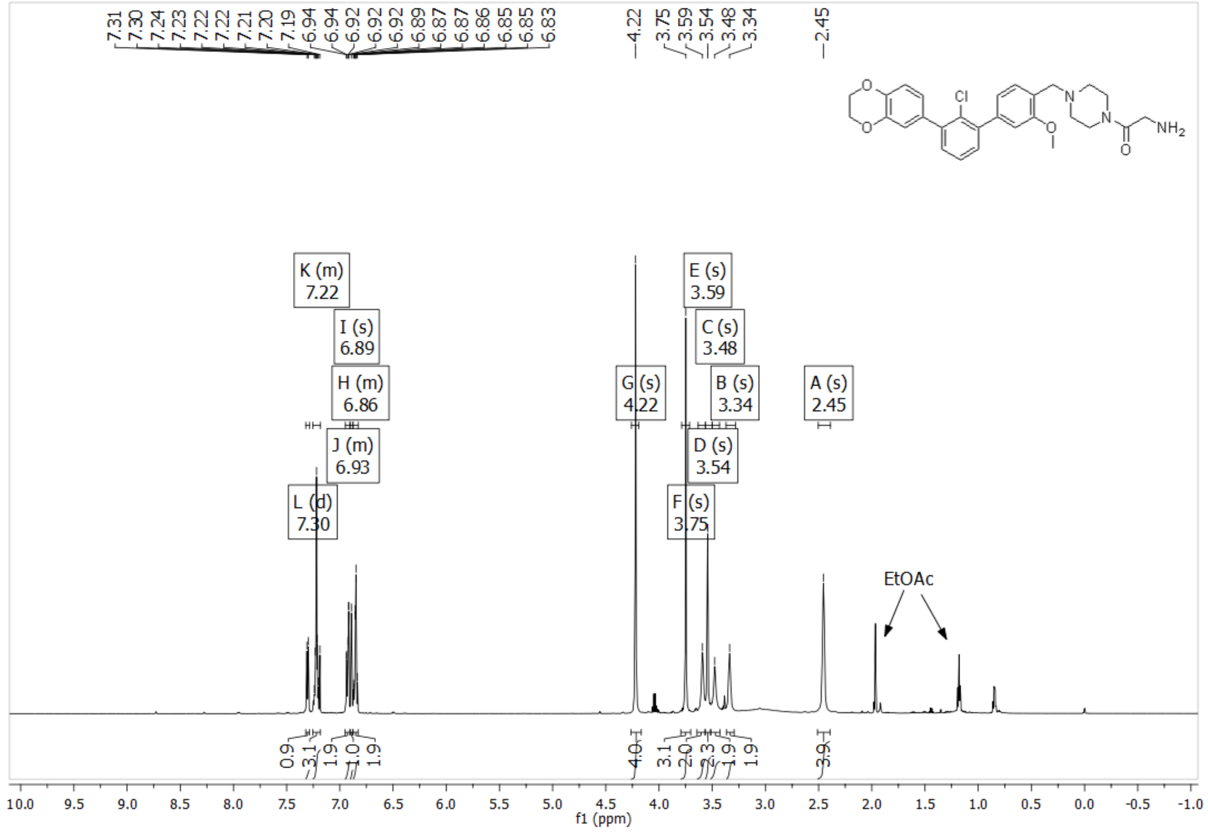

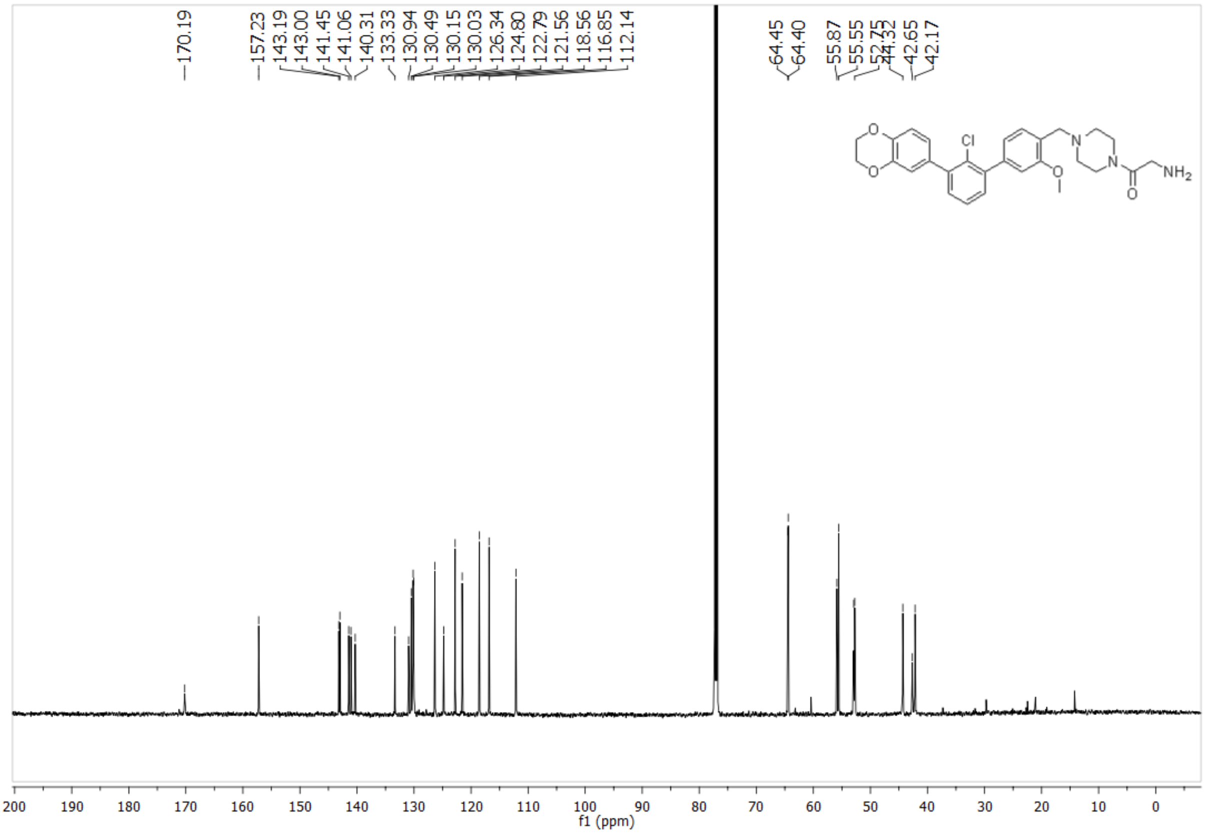

8e. 1-((2'-chloro-3'-(benzo-1,4-dioxan-6-yl)-3-methoxy-[1,1'-biphenyl]-4-yl)methyl)-4-(methylsulfonyl)piperazine

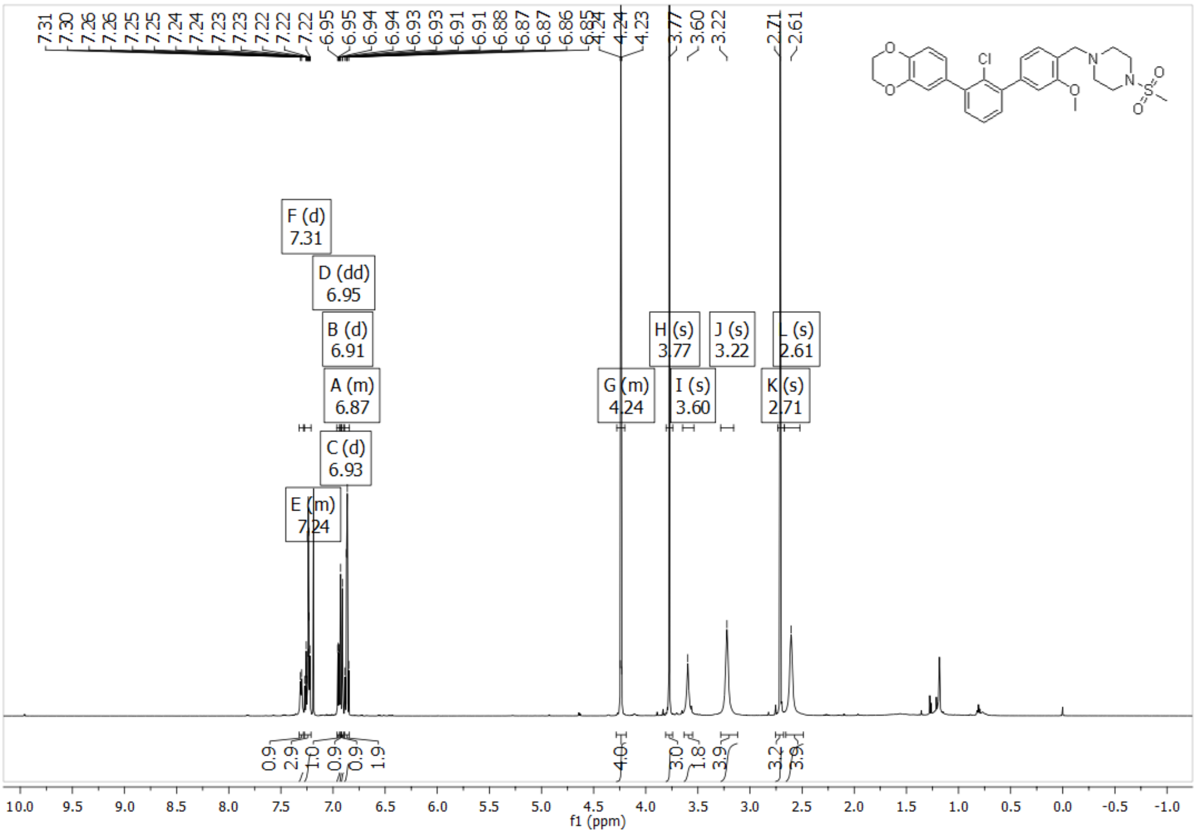

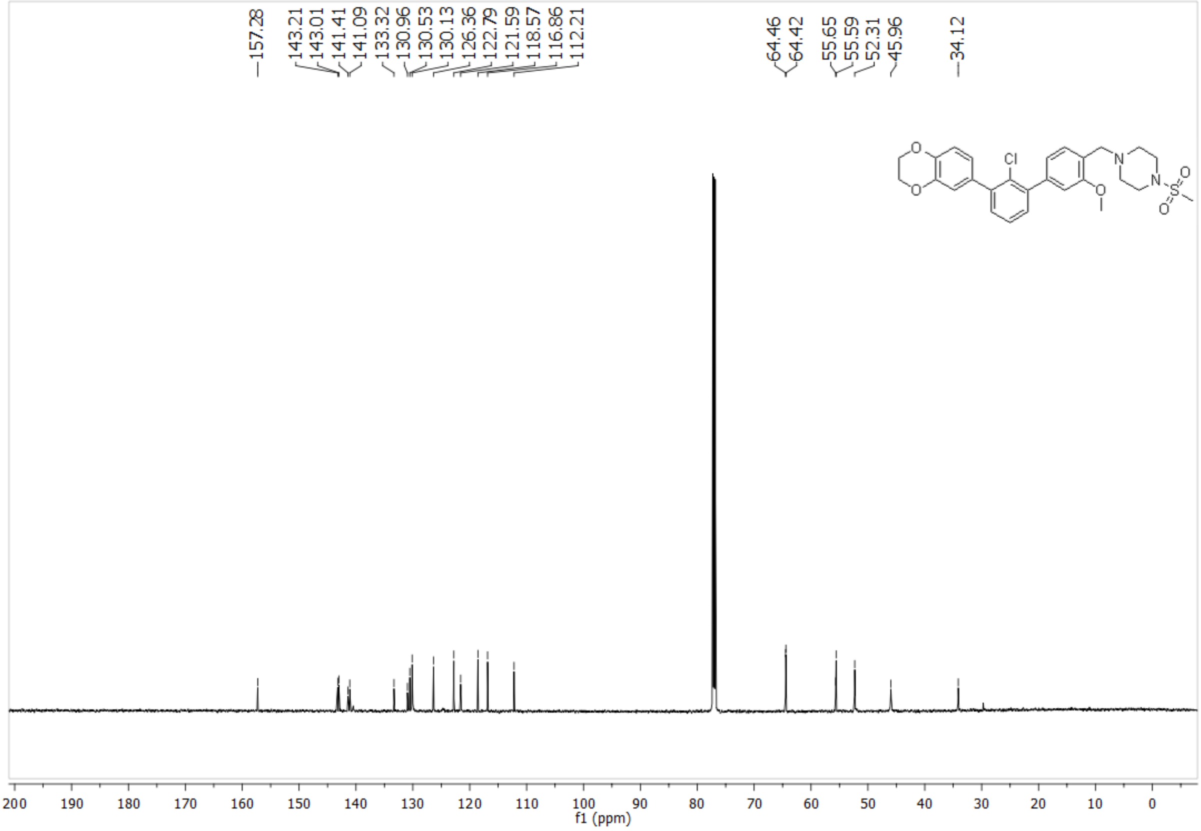

8f. 2-amino-N-(2-(((benzo-1,4-dioxan-6-yl)-3-methoxy-[1,1'-biphenyl]-4-yl)methyl)amino)ethyl)acetamide

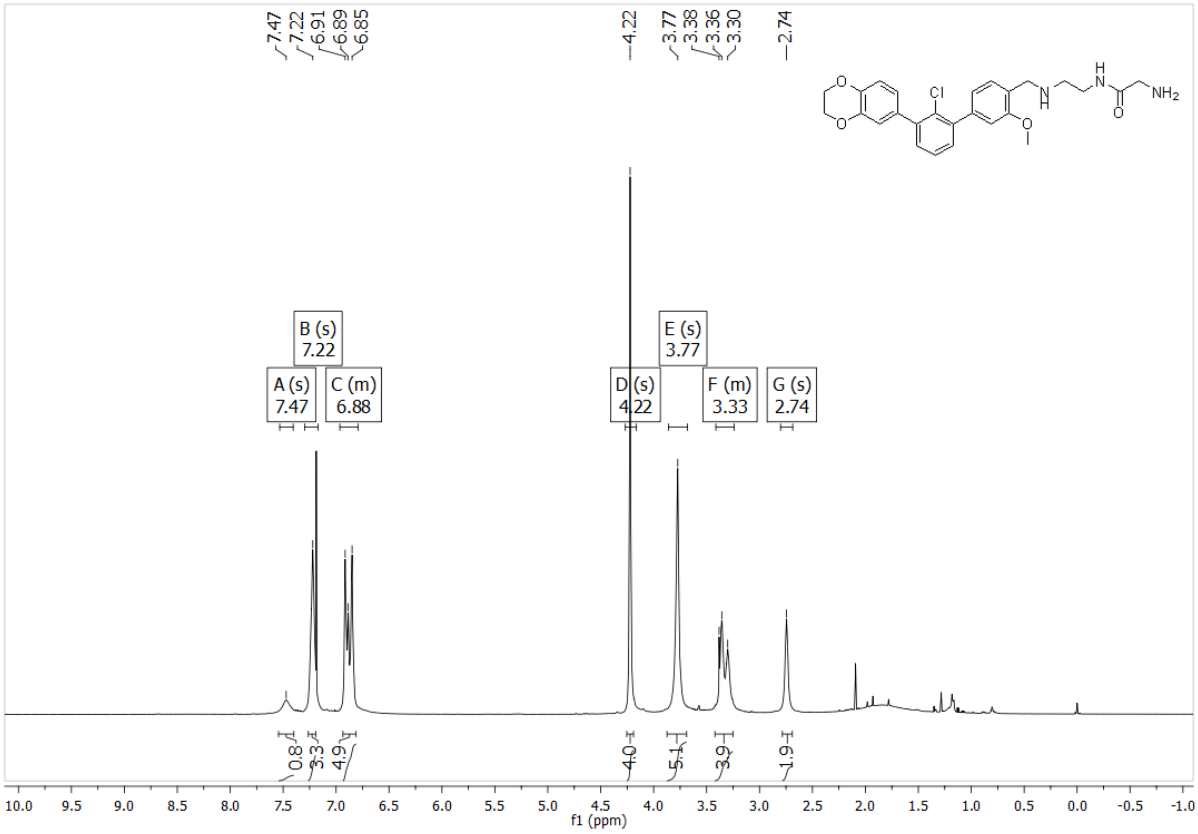

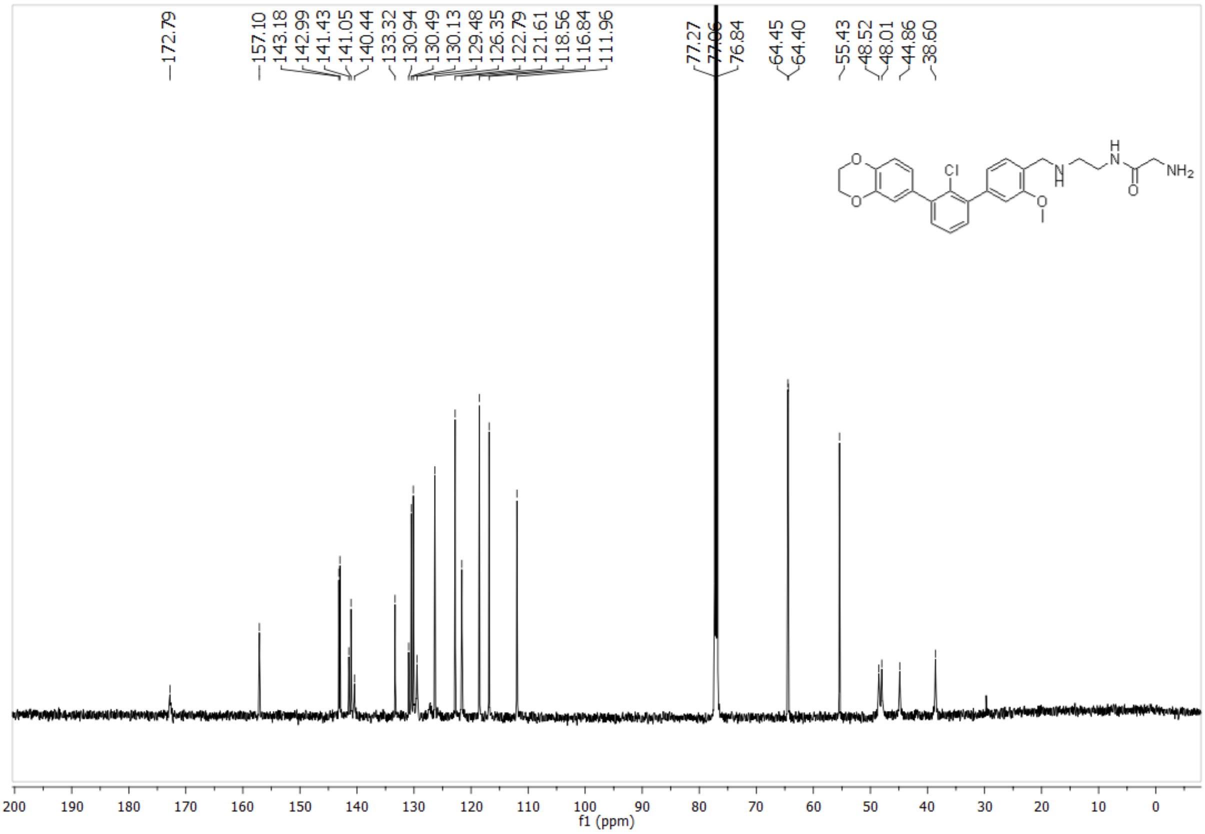

8g. N-(2-(((2'-chloro-3'-(benzo-1,4-dioxan-6-yl)-3-methoxy-[1,1'-biphenyl]-4-yl)methyl)amino)ethyl)methanesulfonamide

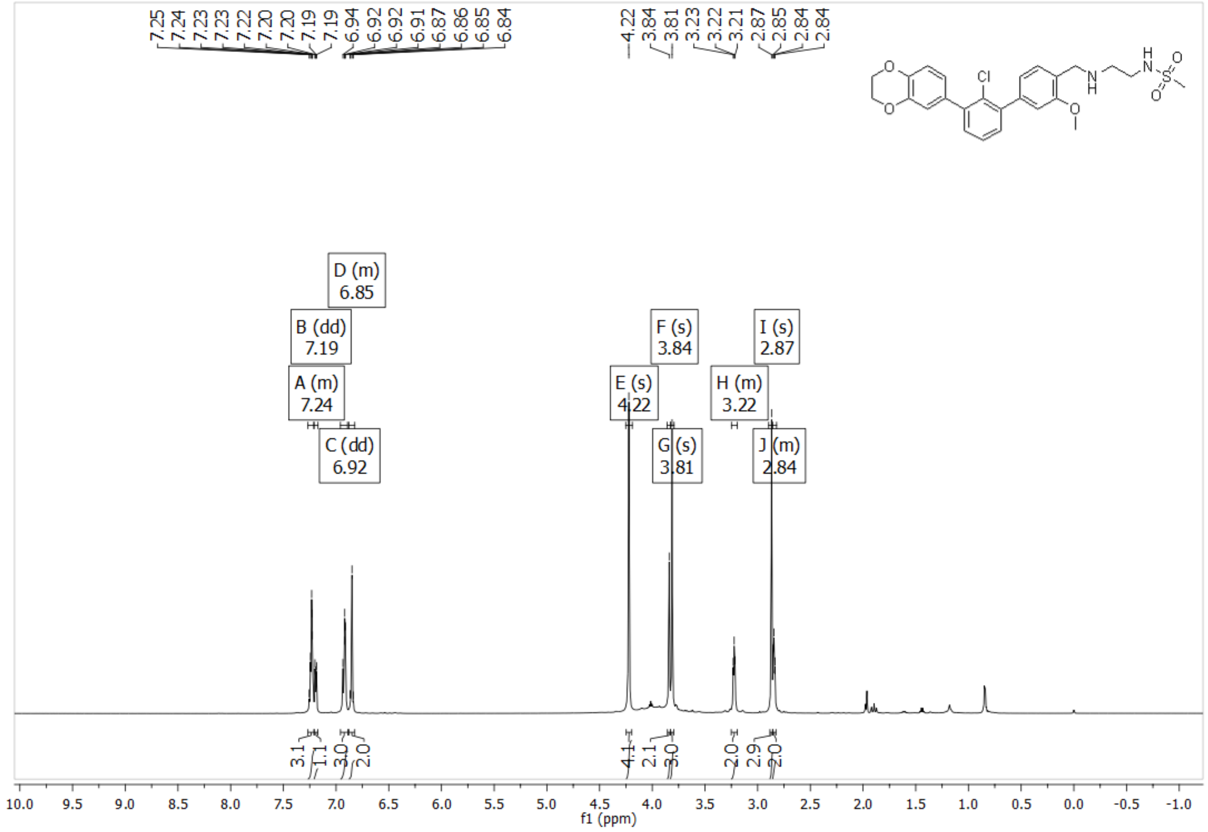

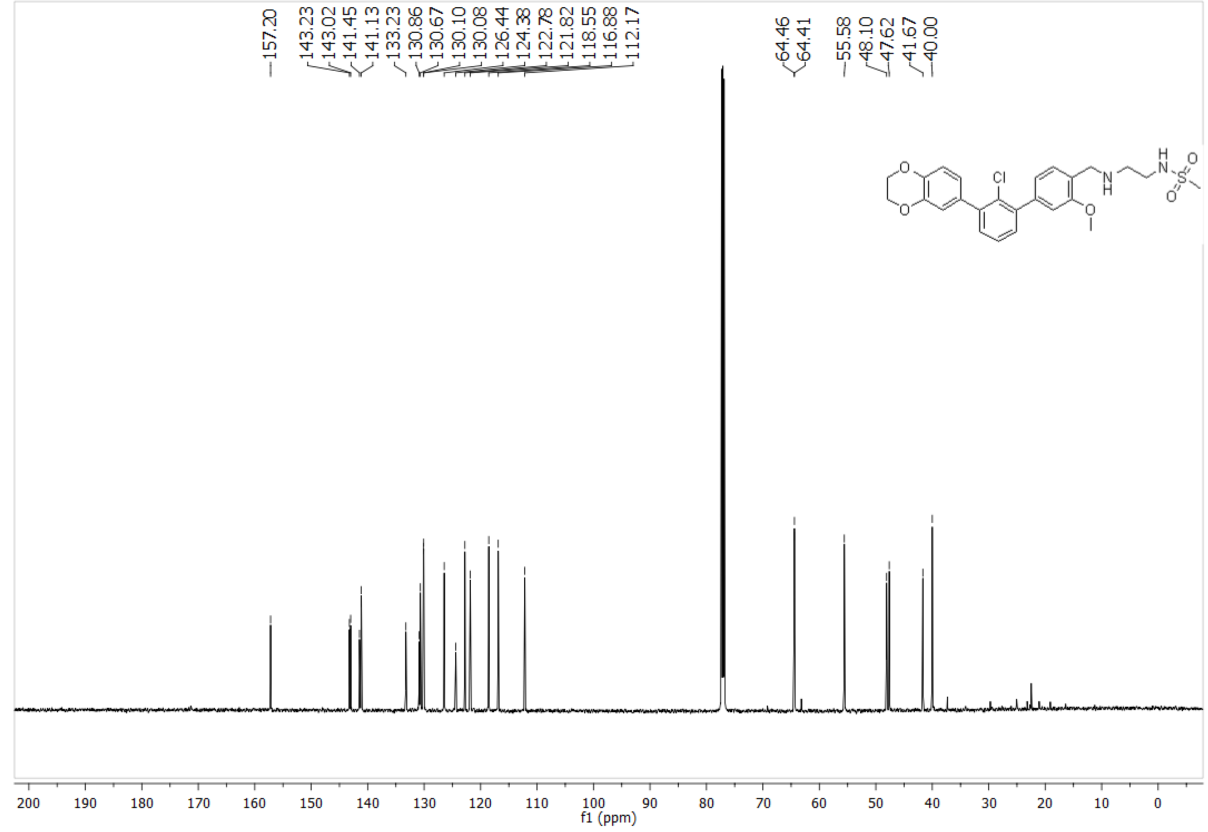

8h. 1-(2-(((2'-Chloro-3'-(2,3-dihydrobenzo[b][1,4]dioxin-6-yl)-3-methoxy-[1,1'-biphenyl]-4-yl)methyl)amino)ethyl)urea

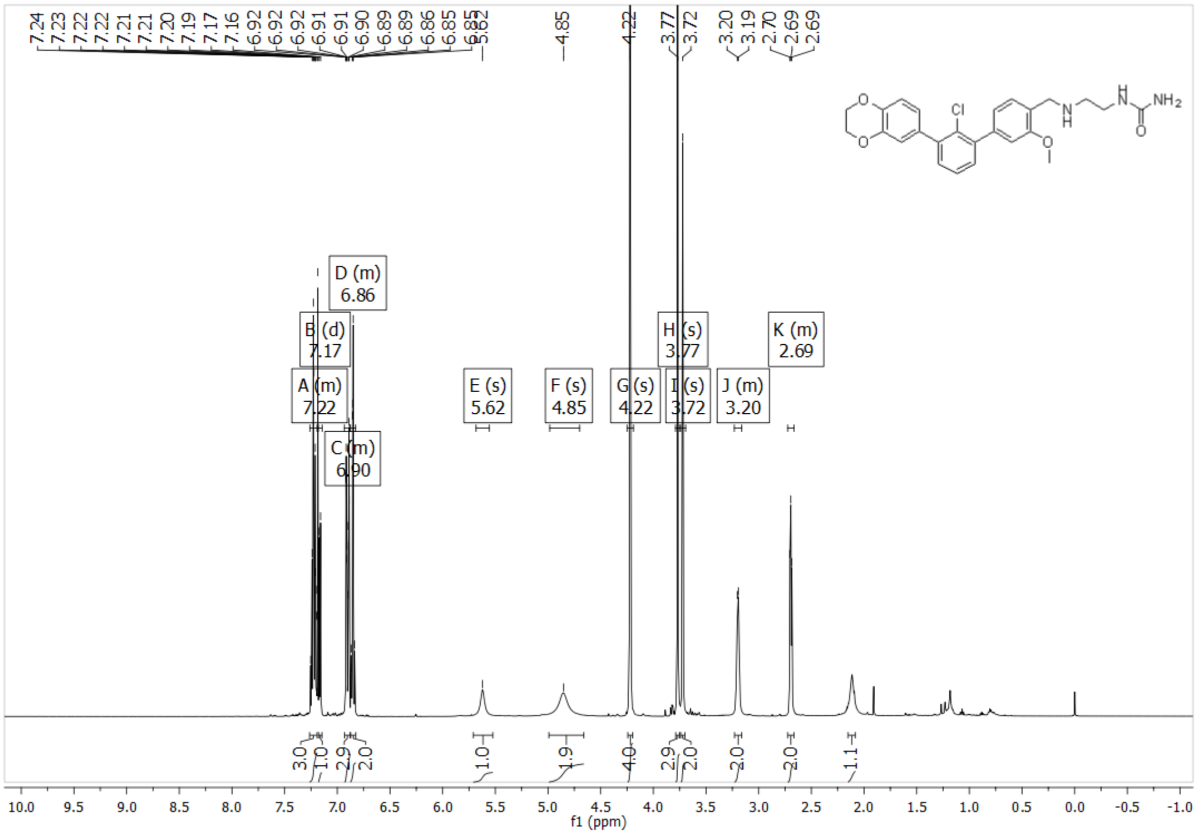

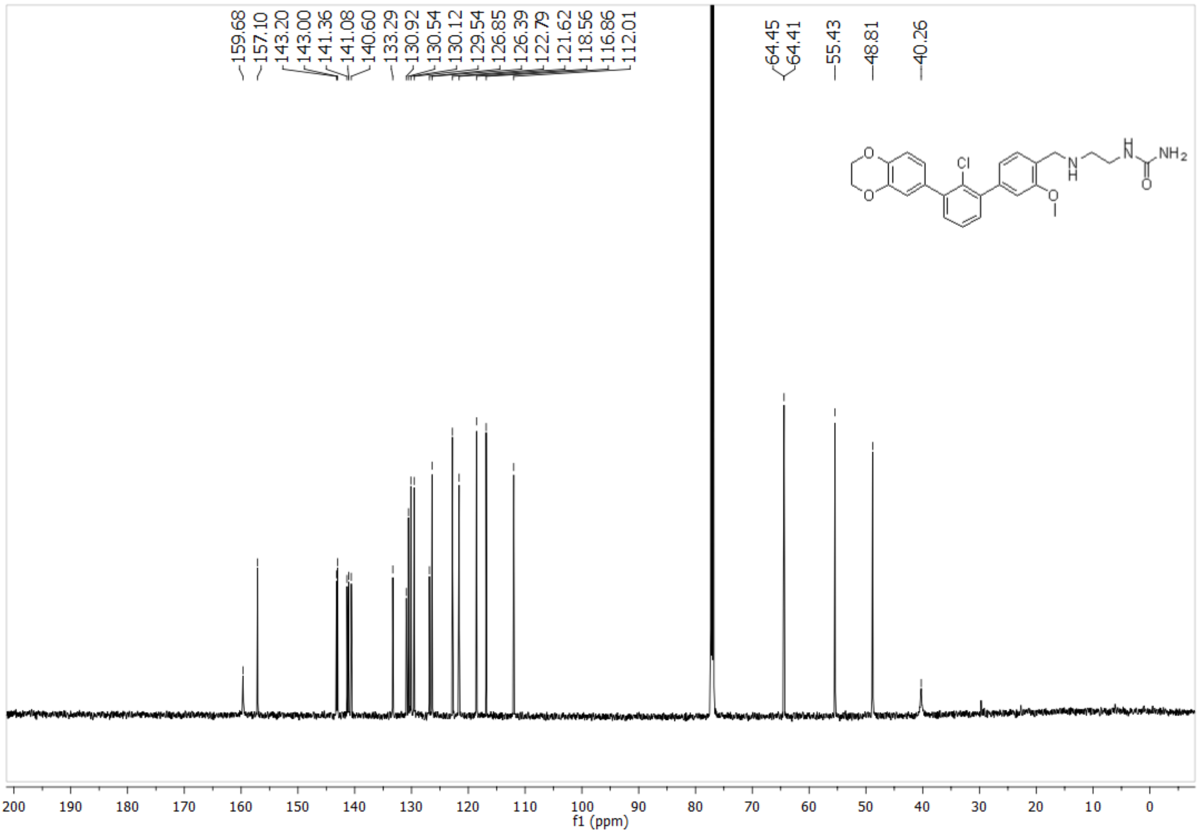

8i. 2-(((2'-chloro-3'-(benzo-1,4-dioxan-6-yl)-3-methoxy-[1,1'-biphenyl]-4-yl)methyl)amino)propane-1,3-diol

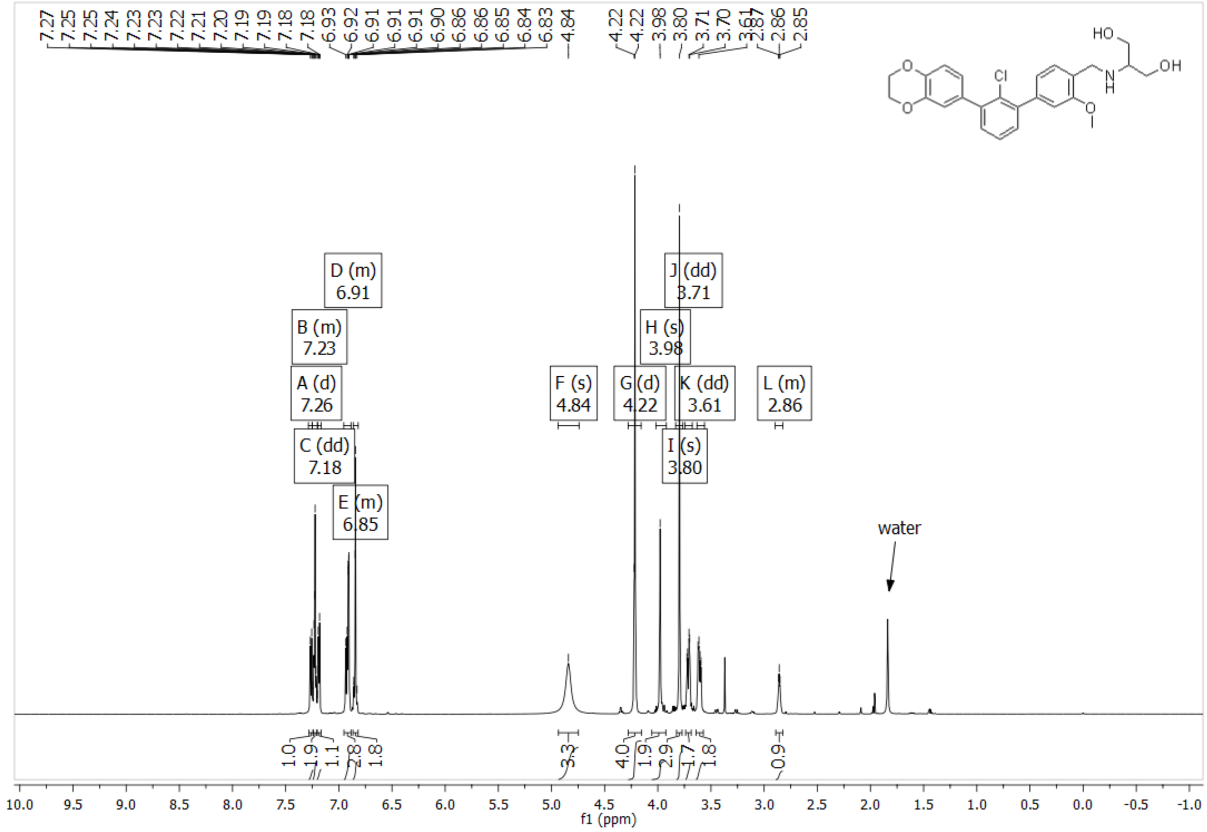

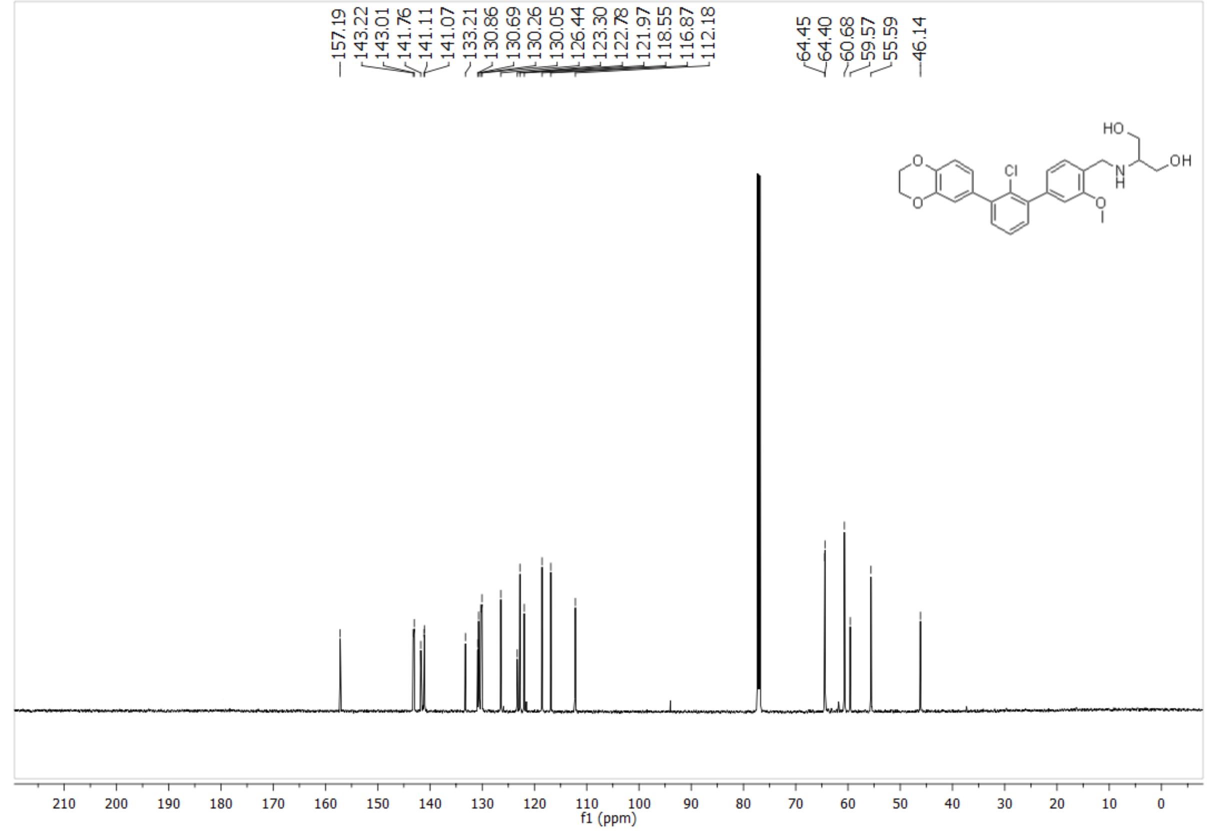

8j. 2-(((2'-chloro-3'-(benzo-1,4-dioxan-6-yl)-3-methoxy-[1,1'-biphenyl]-4-yl)methyl)amino)-2-(hydroxymethyl)propane-1,3-diol

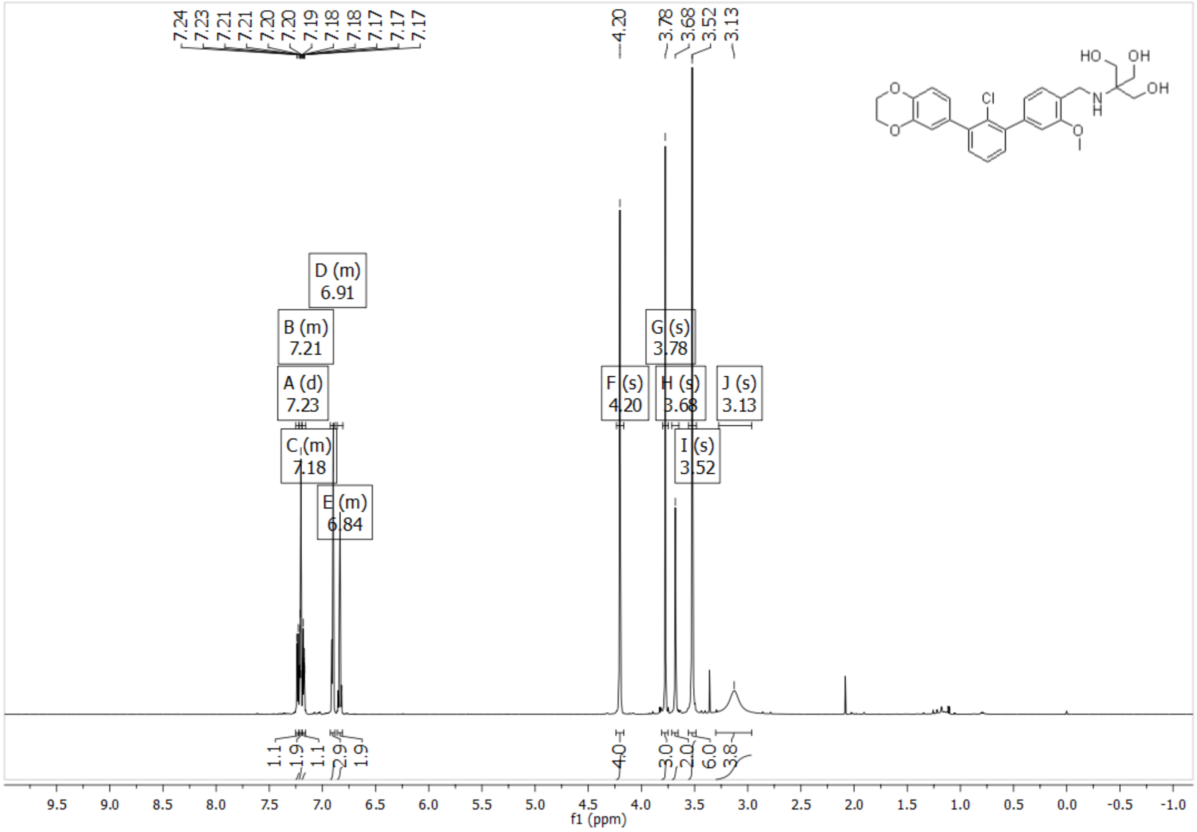

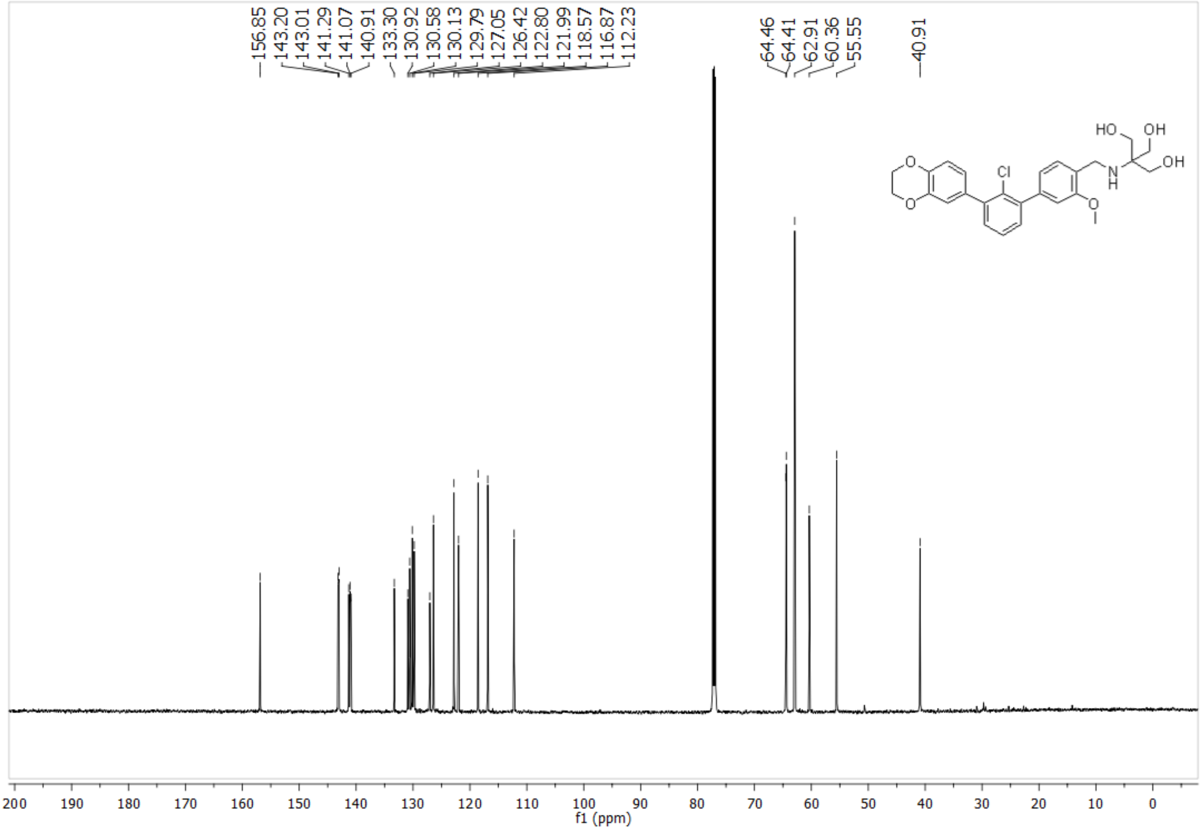

4. COPIES OF LCMS OF FINAL COMPOUNDS

4a. (3'-(Benzo-1,4-dioxan-6-yl)-2'-methyl-[1,1'-biphenyl]-4-yl)methanol

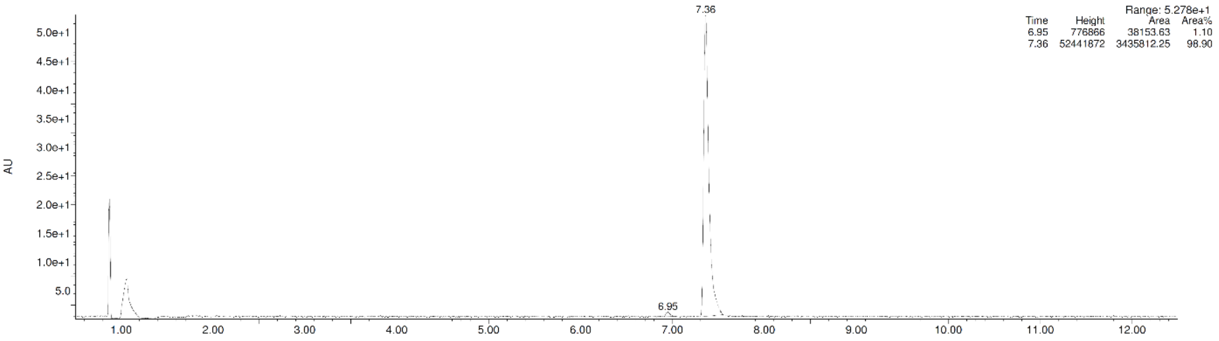

4b. (3'-(Benzo-1,4-dioxan-6-yl)-2'-methyl-[1,1'-biphenyl]-3-yl)methanol

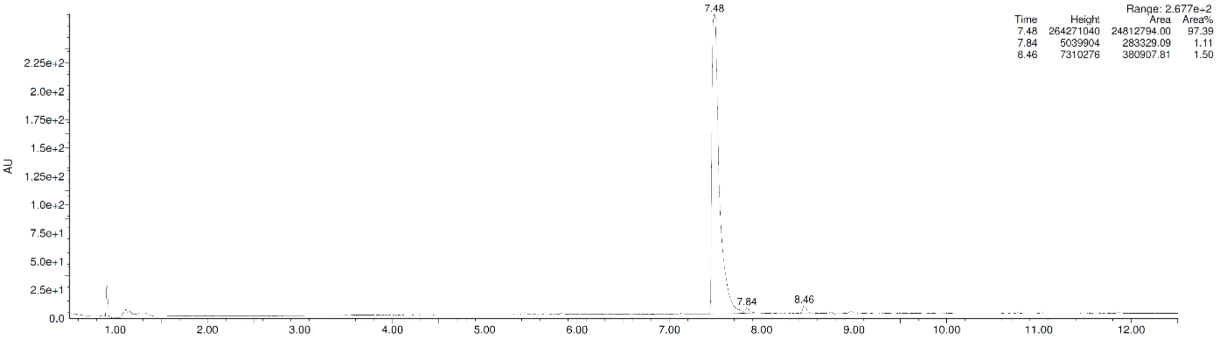

4c. (3'-(Benzo-1,4-dioxan-6-yl)-2'-methyl-[1,1'-biphenyl]-2-yl)methanol

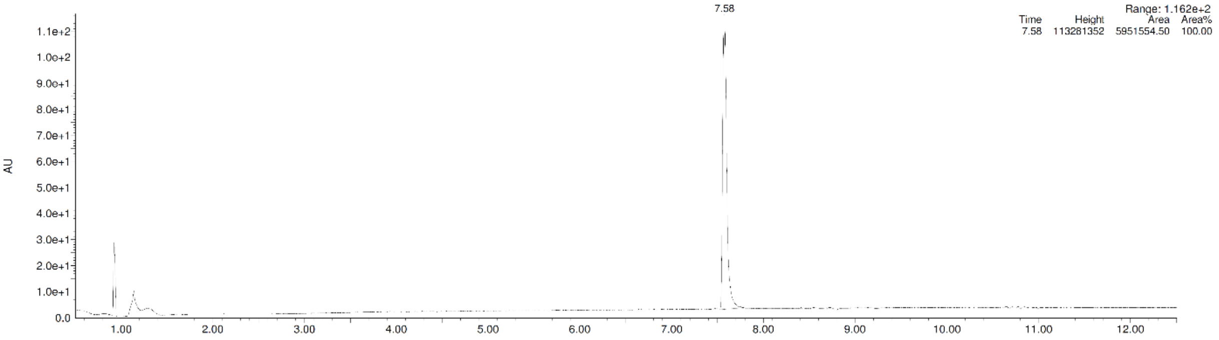

4d. (2'-Amino-3'-(benzo-1,4-dioxan-6-yl)-[1,1'-biphenyl]-4-yl)methanol

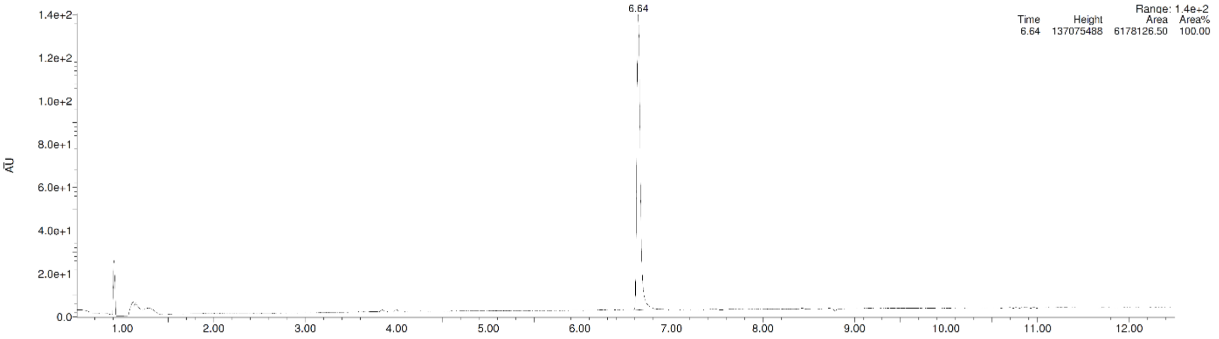

4e. (3'-(Benzo-1,4-dioxan-6-yl)-2'-chloro-[1,1'-biphenyl]-4-yl)methanol

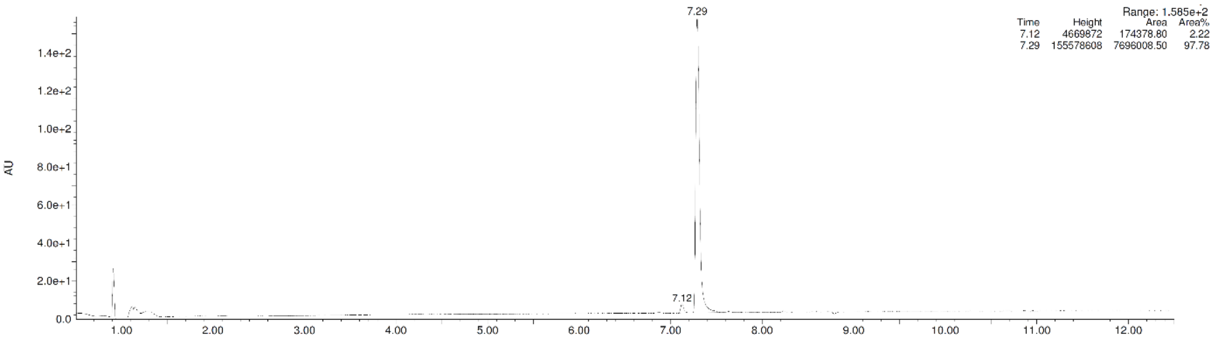

4f. (2'-Bromo-3'-(benzo-1,4-dioxan-6-yl)-[1,1'-biphenyl]-4-yl)methanol

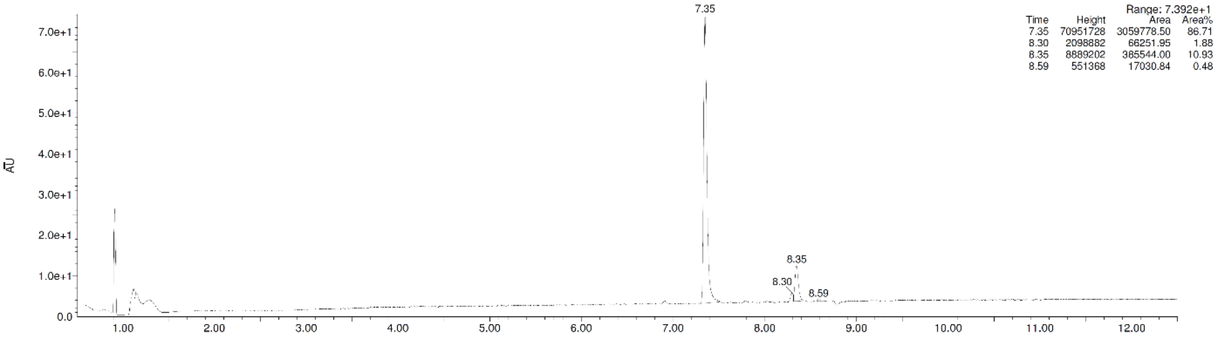

4g. (3'-(Benzo-1,4-dioxan-6-yl)-2'-iodo-[1,1'-biphenyl]-4-yl)methanol

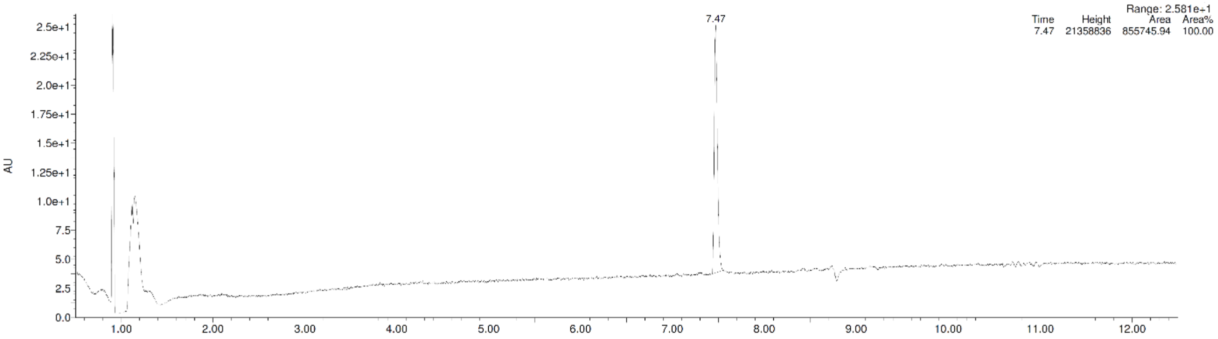

4h. 3-(3'-(Benzo-1,4-dioxan-6-yl)-4'-(hydroxymethyl)-[1,1'-biphenyl]-2-carbonitrile

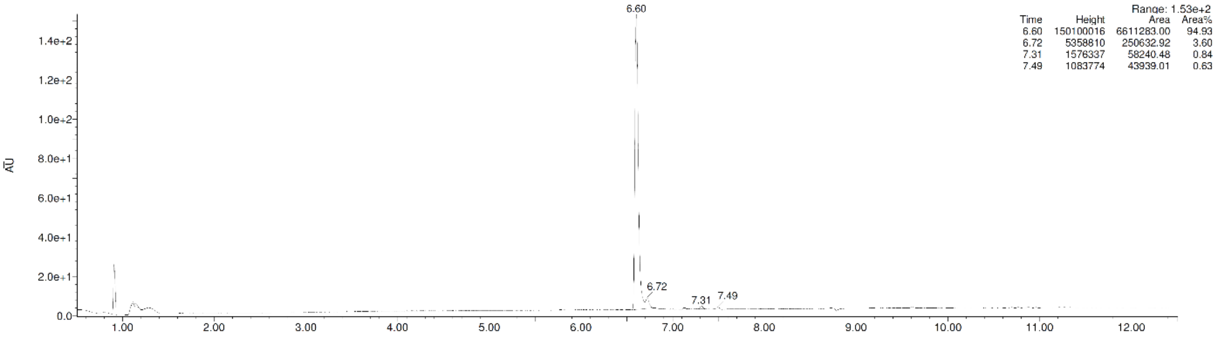

5b. 3-((3'-(benzo-1,4-dioxan-6-yl)-2'-methyl-[1,1'-biphenyl]-4-yl)methoxy)benzoic acid

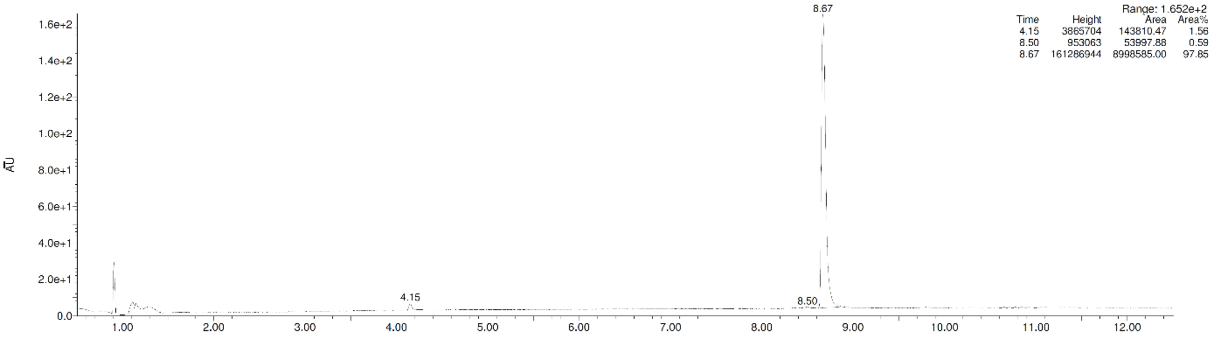

5d. 5-((3'-(benzo-1,4-dioxan-6-yl)-2'-methyl-[1,1'-biphenyl]-4-yl)methoxy)-2-(pyridin-3-yl)benzaldehyde

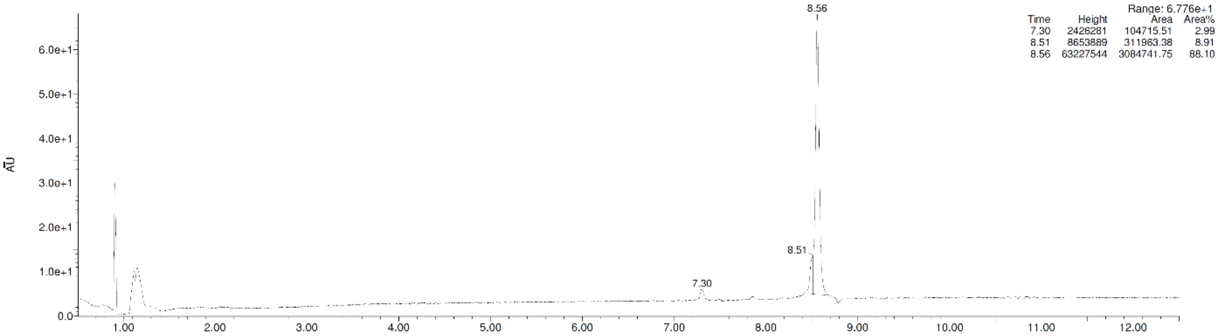

5f. 3-((3'-(benzo-1,4-dioxan-6-yl)-2'-methyl-[1,1'-biphenyl]-4-yl)methoxy)isoxazole-5-carboxylic acid

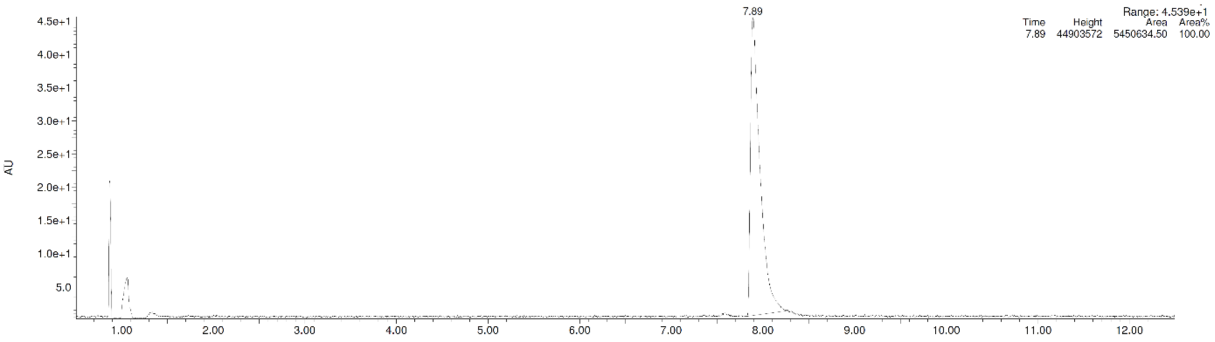

5h. 1-((3'-(benzo-1,4-dioxan-6-yl)-2'-methyl-[1,1'-biphenyl]-4-yl)methyl)-1H-1,2,3-triazole-4-carboxylic acid

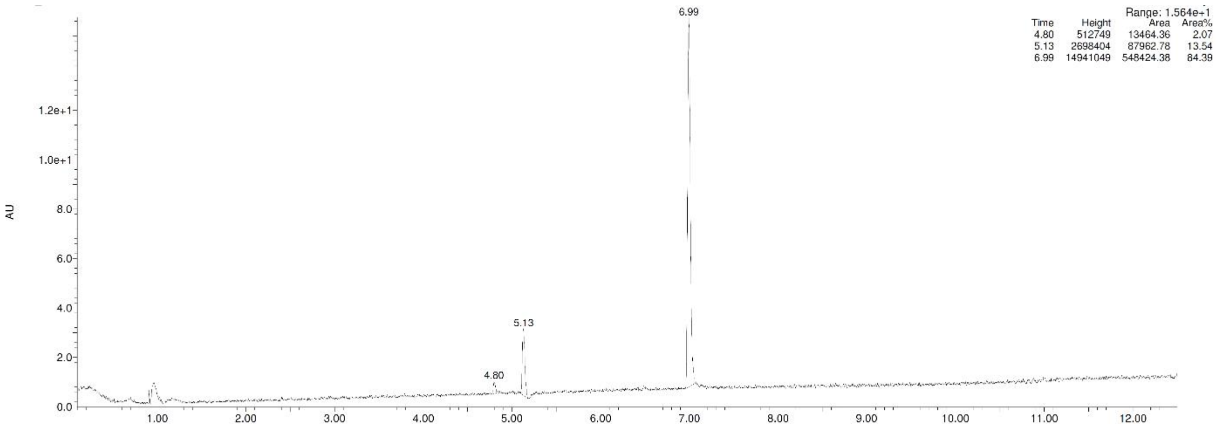

5j. 3-((3'-(benzo-1,4-dioxan-6-yl)-2'-methyl-[1,1'-biphenyl]-3-yl)methoxy)benzoic acid

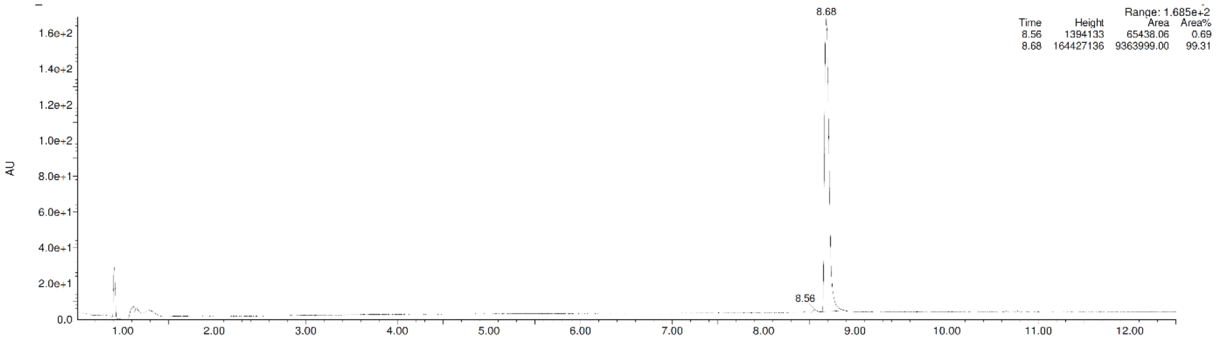

5l. 5-((3'-(benzo-1,4-dioxan-6-yl)-2'-methyl-[1,1'-biphenyl]-3-yl)methoxy)-2-(pyridin-3-yl)benzaldehyde

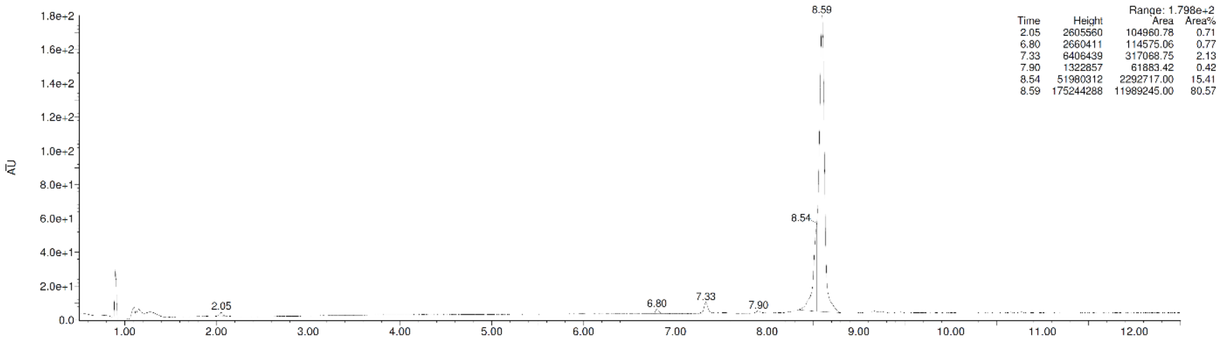

5n. 3-((3'-(benzo-1,4-dioxan-6-yl)-2'-methyl-[1,1'-biphenyl]-3-yl)methoxy)isoxazole-5-carboxylic acid

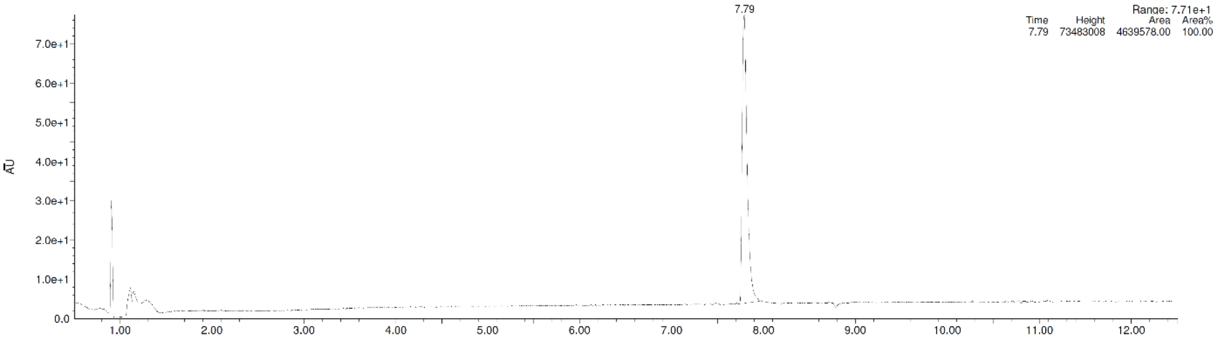

5p. 3-((3'-(benzo-1,4-dioxan-6-yl)-2'-methyl-[1,1'-biphenyl]-2-yl)methoxy)benzoic acid

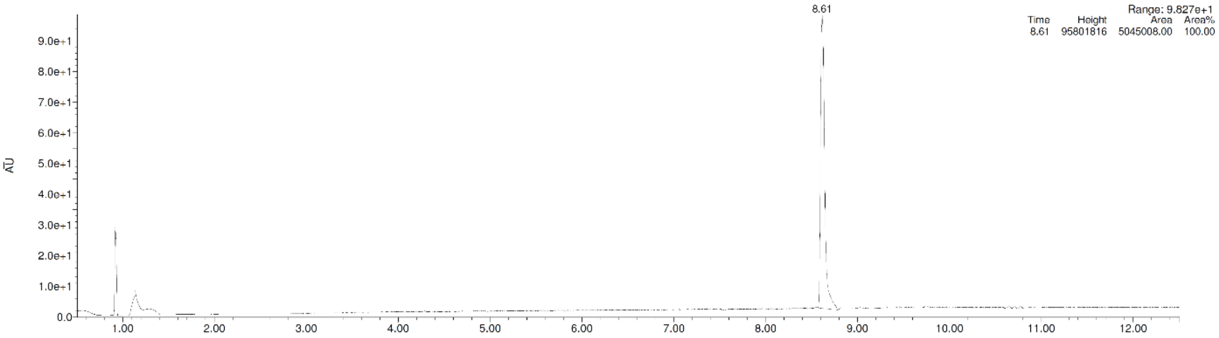

5r. 3-((3'-(benzo-1,4-dioxan-6-yl)-2'-methyl-[1,1'-biphenyl]-2-yl)methoxy)isoxazole-5-carboxylic acid

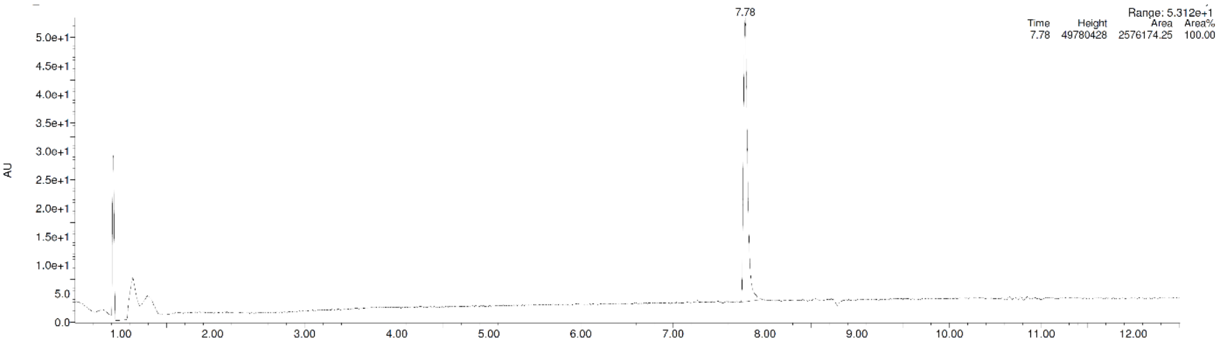

6a. 4-((3'-(benzo-1,4-dioxan-6-yl)-2'-methyl-[1,1'-biphenyl]-4-yl)methyl)morpholine

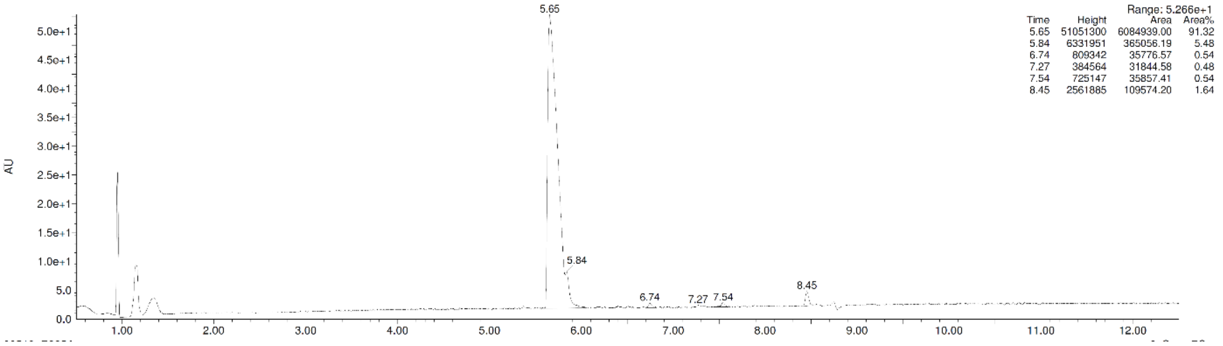

6b. 4-((3'-(benzo-1,4-dioxan-6-yl)-2'-methyl-[1,1'-biphenyl]-4-yl)methyl)piperazine

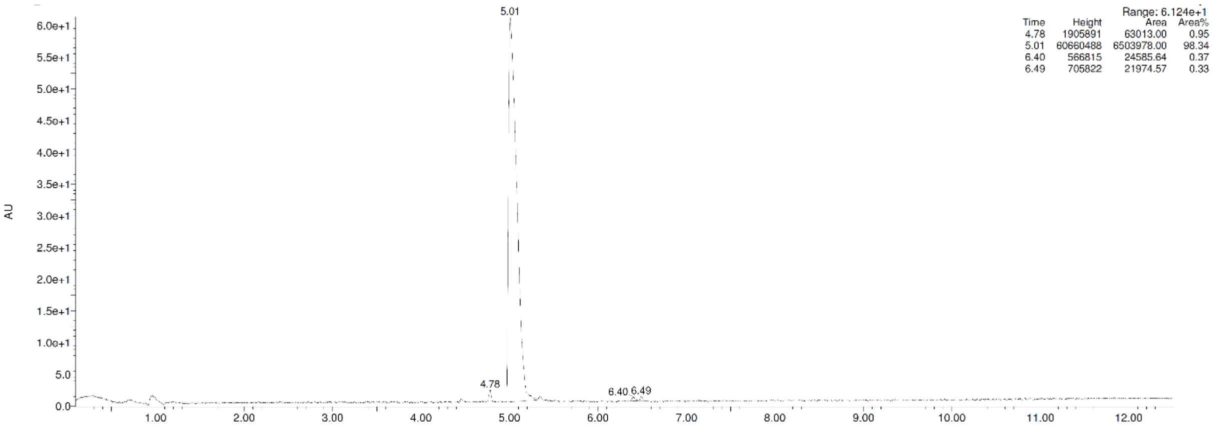

6c. 1-((3'-(benzo-1,4-dioxan-6-yl)-2'-methyl-[1,1'-biphenyl]-3-yl)methyl)piperazine

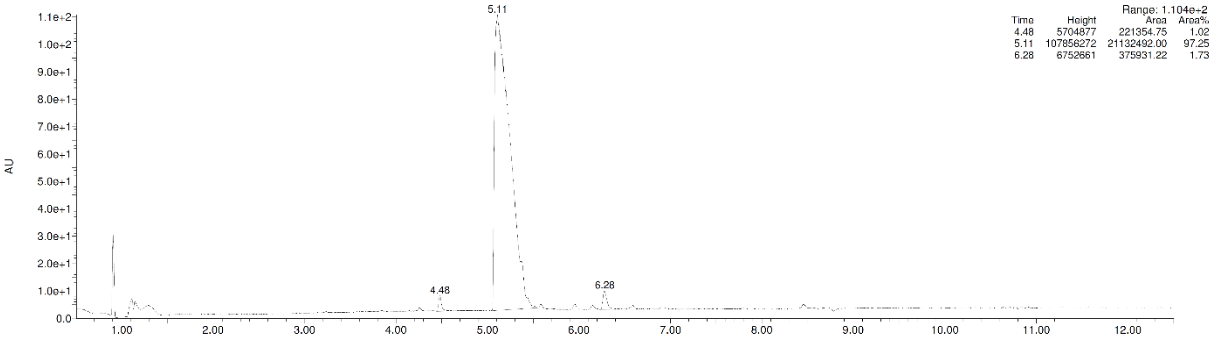

6d. 1-((3'-(benzo-1,4-dioxan-6-yl)-3-methoxy-2'-methyl-[1,1'-biphenyl]-4-yl)methyl)piperazine

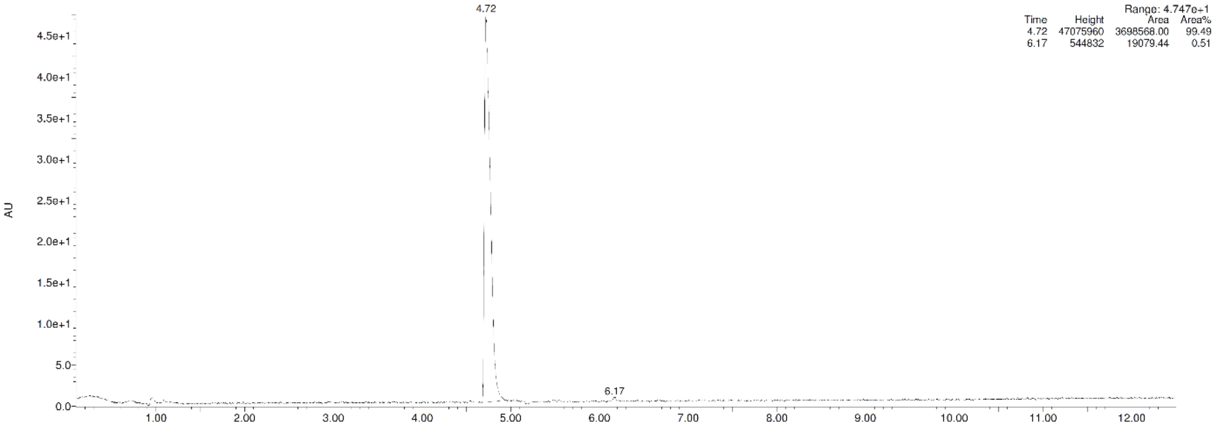

6e. 3-(((3'-(2,3-benzo-1,4-dioxan-6-yl)-2'-methyl-4-(piperazin-1-ylmethyl)-[1,1'-biphenyl]-3-yl)oxy)methyl)benzonitrile

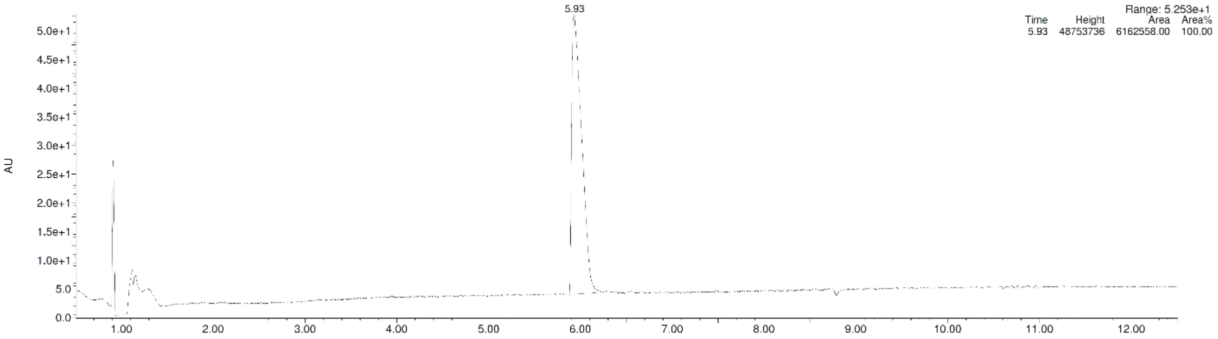

7a. N-(2-(((2'-chloro-3'-(benzo-1,4-dioxan-6-yl)-3-methoxy-[1,1'-biphenyl]-4-yl)methyl)amino)ethyl)acetamide

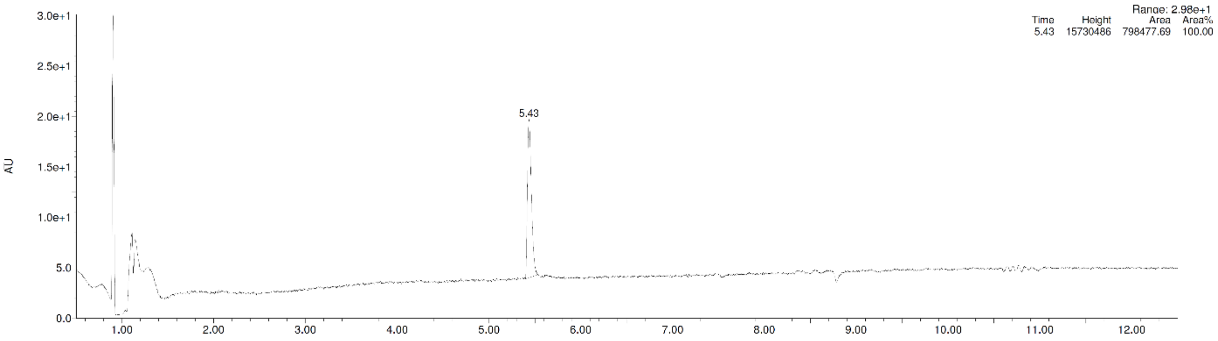

7b. N-(2-((((benzo-1,4-dioxan-6-yl)-2'-chloro-3'-3-ethoxy-[1,1'-biphenyl]-4-yl)methyl)amino)ethyl)acetamide

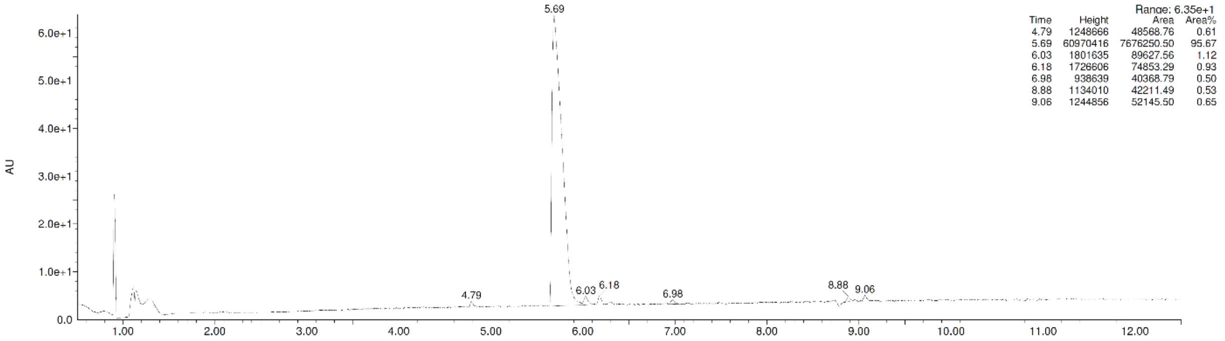

7c. N-(2-(((2'-chloro-3-((3'-cyano-[1,1'-biphenyl]-3-yl)isopropoxy)-3'-(benzo-1,4-dioxan-6-yl)-[1,1'-biphenyl]-4-yl)methyl)amino)ethyl)acetamide

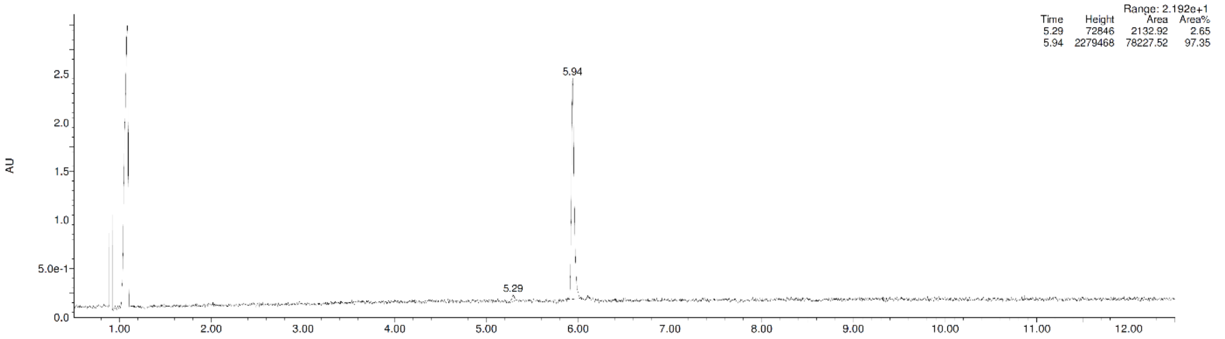

7d. N-(2-(((2'-chloro-3-((3'-cyano-[1,1'-biphenyl]-3-yl)isopentyloxy)-3'-(benzo-1,4-dioxan-6-yl)-[1,1'-biphenyl]-4-yl)methyl)amino)ethyl)acetamide

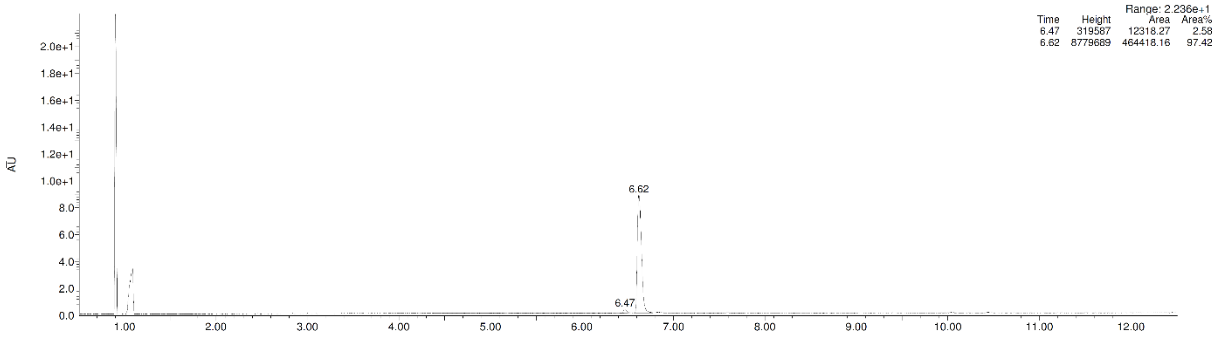

7e. N-(2-(((3'-(benzo-1,4-dioxan-6-yl)-2'-chloro-3-(cyanomethoxy)-[1,1'-biphenyl]-4-yl)methyl)amino)ethyl)acetamide

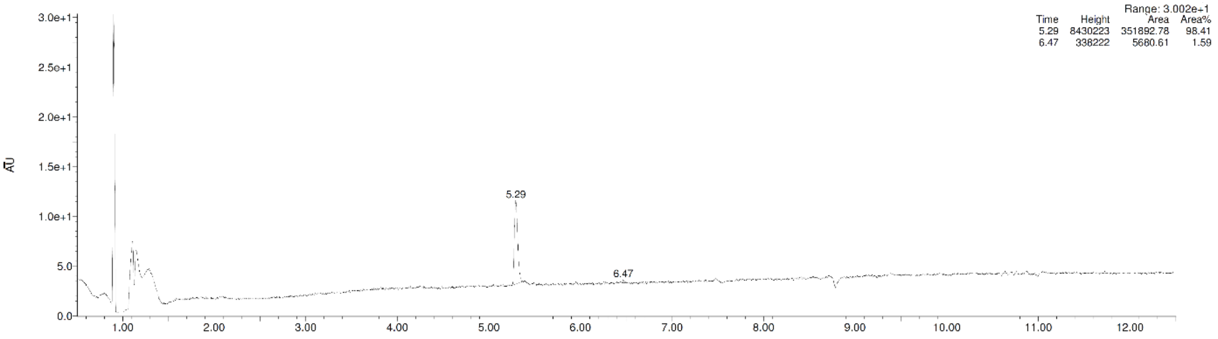

7f. N-(2-(((3'-(benzo-1,4-dioxan-6-yl)-2'-chloro-3-(cyclobutylmethoxy)-[1,1'-biphenyl]-4-yl)methyl)amino)ethyl)acetamide

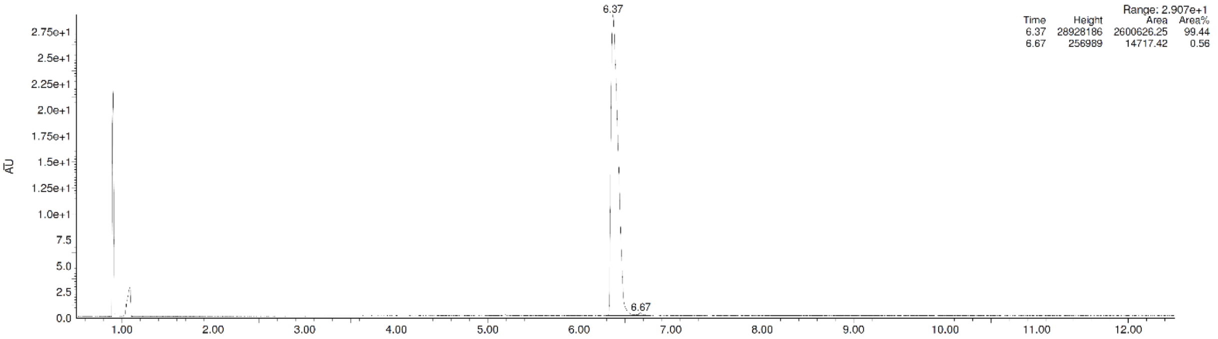

7g. N-(2-(((3'-(benzo-1,4-dioxan-6-yl)-2'-chloro-3-(cyclopentylmethoxy)-[1,1'-biphenyl]-4-yl)methyl)amino)ethyl)acetamide

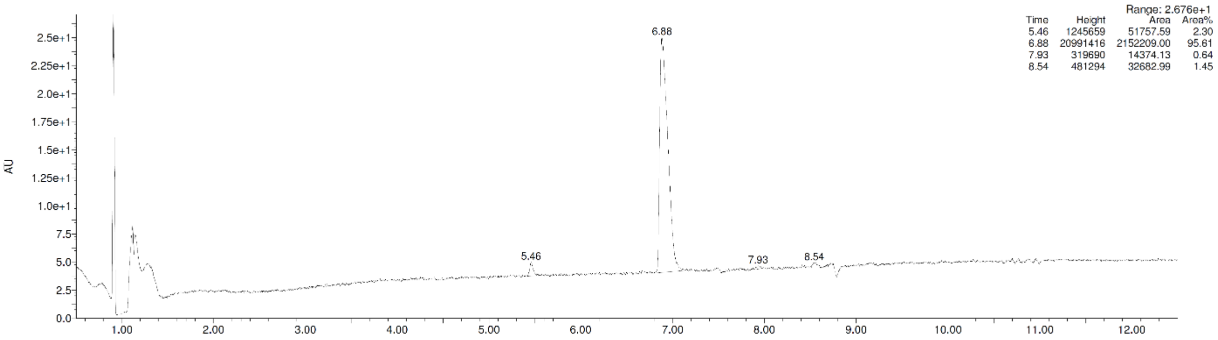

7h. N-(2-(((3'-(benzo-1,4-dioxan-6-yl)-2'-chloro-3-((3'-cyano-[1,1'-biphenyl]-3-yl)methoxy)-[1,1'-biphenyl]-4-yl)methyl)amino)ethyl)acetamide

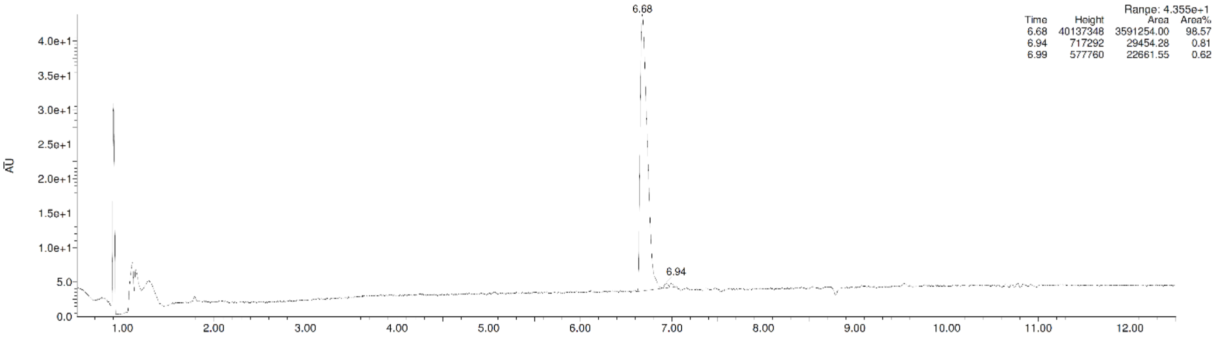

8a. 1-((2'-chloro-3'-(benzo-1,4-dioxan-6-yl)-3-methoxy-[1,1'-biphenyl]-4-yl)methyl)piperazine

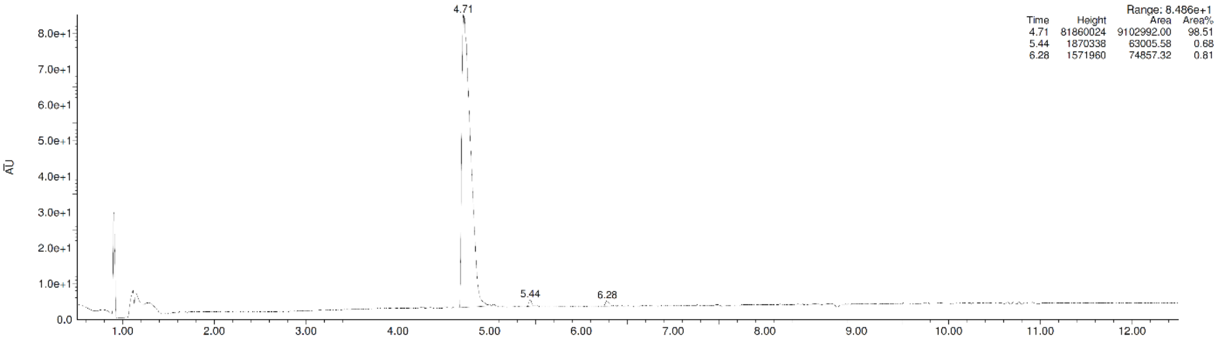

8b. 4-((2'-chloro-3'-(benzo-1,4-dioxan-6-yl)-3-methoxy-[1,1'-biphenyl]-4-yl)methyl)piperazine-1-carboxamide

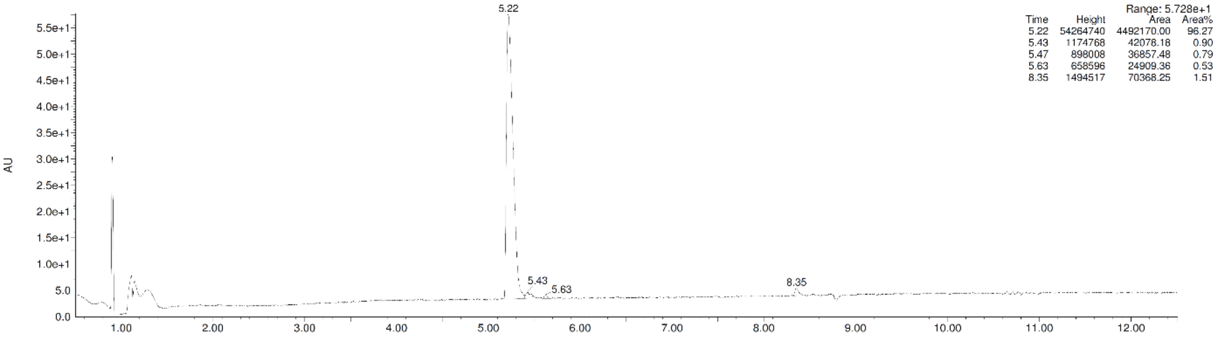

8c. 1-(4-((2'-Chloro-3'-(benzo-1,4-dioxan-6-yl)-3-methoxy-[1,1'-biphenyl]-4-yl)methyl)piperazin-1-yl)ethan-1-one

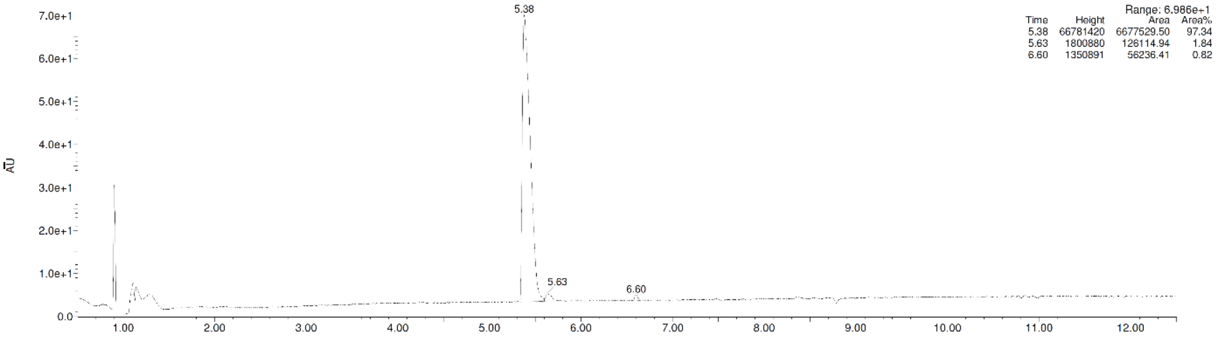

8d. 2-amino-1-(4-((2'-chloro-3'-(benzo-1,4-dioxan-6-yl)-3-methoxy-[1,1'-biphenyl]-4-yl)methyl)piperazin-1-yl)ethan-1-one

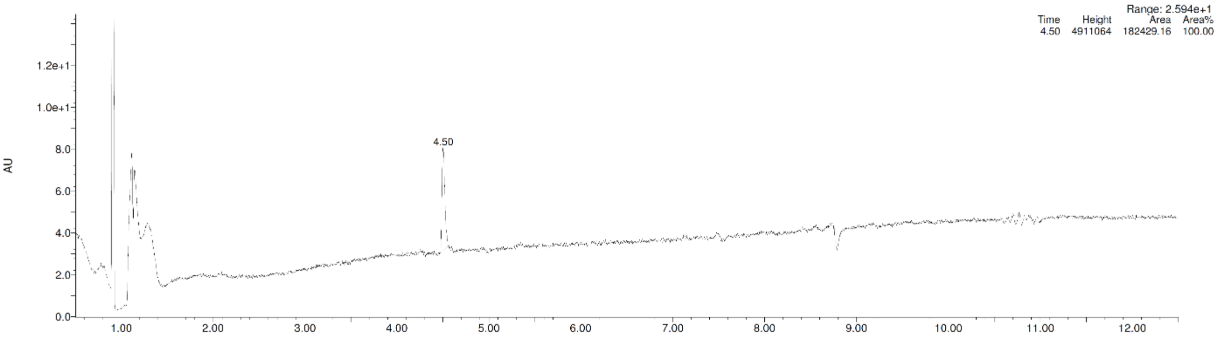

8e. 1-((2'-chloro-3'-(benzo-1,4-dioxan-6-yl)-3-methoxy-[1,1'-biphenyl]-4-yl)methyl)-4-(methylsulfonyl)piperazine

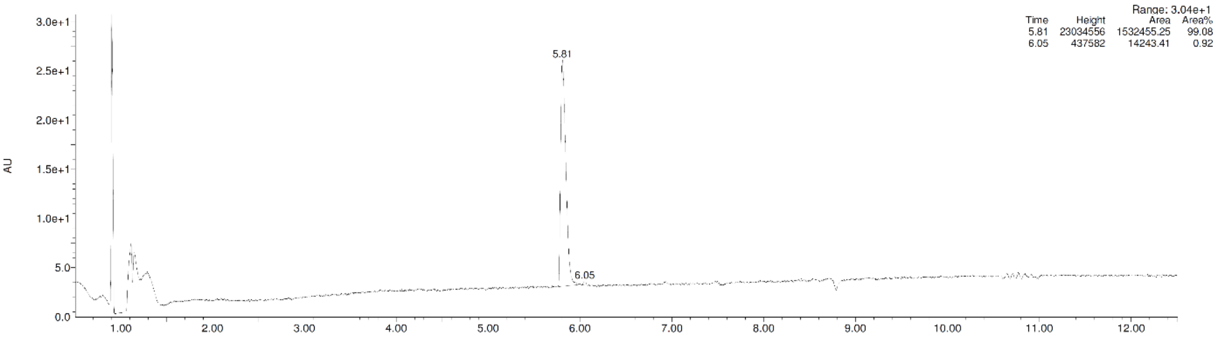

8f. 2-amino-N-(2-(((benzo-1,4-dioxan-6-yl)-3-methoxy-[1,1'-biphenyl]-4-yl)methyl)amino)ethyl)acetamide

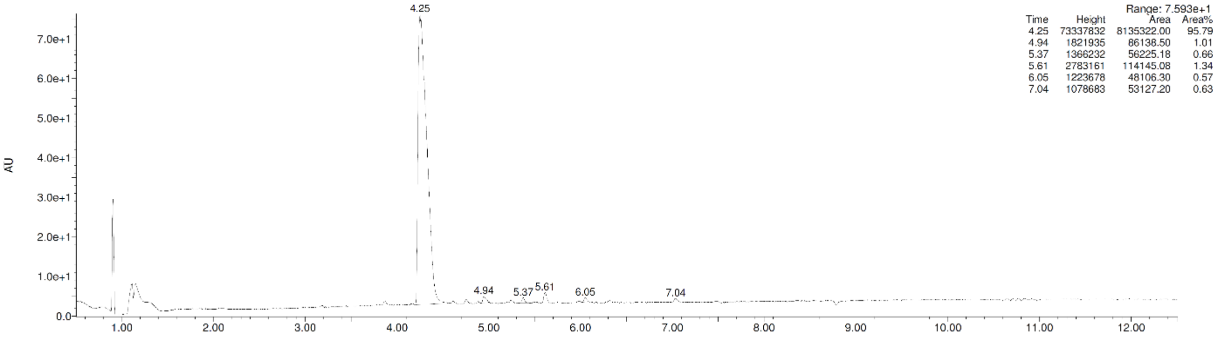

8g. N-(2-(((2'-chloro-3'-(benzo-1,4-dioxan-6-yl)-3-methoxy-[1,1'-biphenyl]-4-yl)methyl)amino)ethyl)methanesulfonamide

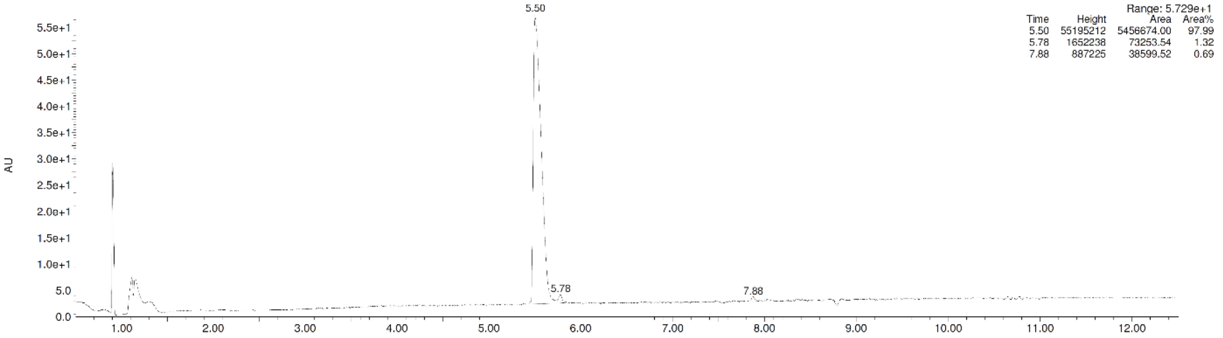

8h. 1-(2-(((2'-Chloro-3'-(2,3-dihydrobenzo[b][1,4]dioxin-6-yl)-3-methoxy-[1,1'-biphenyl]-4-yl)methyl)amino)ethyl)urea

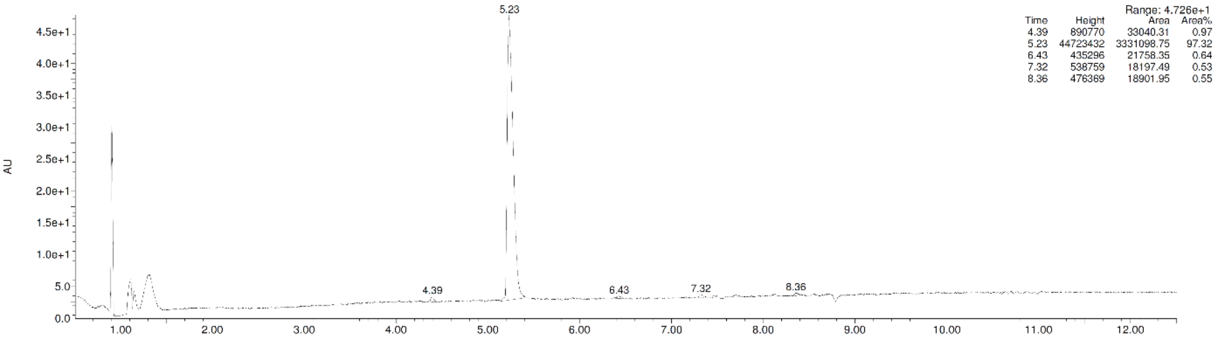

8i. 2-(((2'-chloro-3'-(benzo-1,4-dioxan-6-yl)-3-methoxy-[1,1'-biphenyl]-4-yl)methyl)amino)propane-1,3-diol

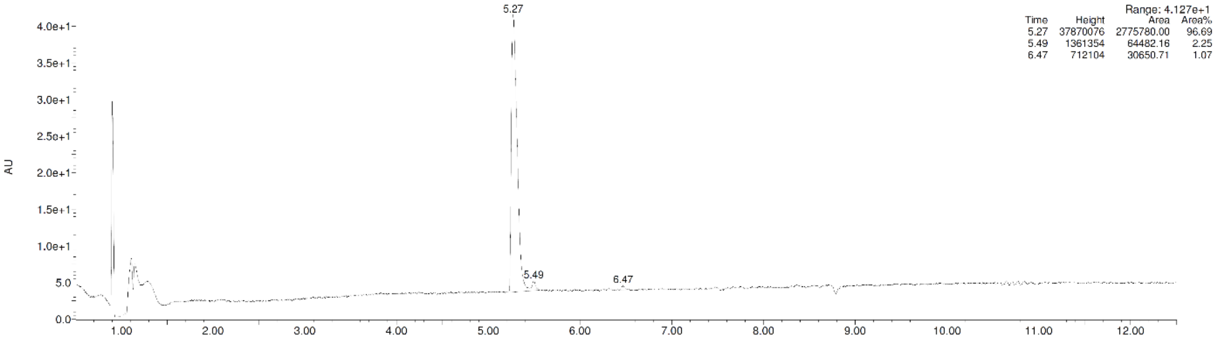

8j. 2-(((2'-chloro-3'-(benzo-1,4-dioxan-6-yl)-3-methoxy-[1,1'-biphenyl]-4-yl)methyl)amino)-2-(hydroxymethyl)propane-1,3-diol

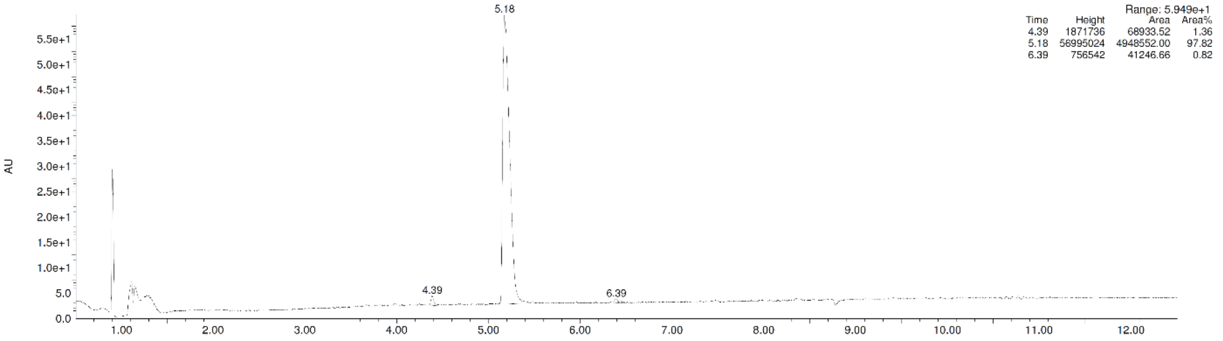

6. REFERENCES

(1) Bacon, E.; Dandu, R.; Guise-Zawacki, L.; Huang, C.-Y.; Hudkins, R.; Tao, M. Substtuted Spirocyclic Piperidine Derivatives as Histamine-3 (H3) Receptor Ligands, WO2009097567A1, Cephalon INC. and Pharmacopeia INC., 2009.

(2) Dowdell, S. E.; Eidam, H. S.; Elban, M.; Fos, R. M.; Hammond, M.; Hilfiker, M. A.; Hoang, T. H.; Kallander, L. S.; Lawhorn, B. G.; Manns, S.; Philip, J.; Washburn, D. G.; Ye, G. Hydroxyformamide Derivatives and Their Use, WO2015104684A1, GlaxoSimthKline IP, 2015.

(3) McQuaid, K. M.; Long, J. Z.; Sames, D. C-H Bond Functionalization via Hydride Transfer: Synthesis of Dihydrobenzopyrans from Ortho-Vinylaryl Akyl Ethers. *Org. Lett.* **2009**, *11* (14), 2972–2975. https://doi.org/10.1021/ol900915p.

(4) Robbins, J. S.; Schmid, K. M.; Phillips, S. T. Effects of Electronics, Aromaticity, and Solvent Polarity on the Rate of Azaquinone-Methide-Mediated Depolymerization of Aromatic Carbamate Oligomers. *J. Org. Chem.* **2013**, *78* (7), 3159–3169. https://doi.org/10.1021/jo400105m.

(5) Corminboeuf, O.; Fretz, H.; Lyothier, I.; Pothier, J.; Pozzi, D.; Richard-Bildstein, S.; Sifferlen, T.; Wyder Peters, L. N-Substituted Indole Derivatives as PGE2 Receptor Modulators, WO2017085198A1, Actelion Pharmaceuticals LTD, 2017.
